# Rediscovering an old foe: Optimised molecular methods for DNA extraction and sequencing applications for fungarium specimens of powdery mildew (Erysiphales)

**DOI:** 10.1371/journal.pone.0232535

**Published:** 2020-05-13

**Authors:** Reannon L. Smith, Tim Sawbridge, Ross Mann, Jatinder Kaur, Tom W. May, Jacqueline Edwards

**Affiliations:** 1 Department of Jobs, Agriculture Victoria Research, Regions and Precincts, Bundoora, Australia; 2 School of Applied Systems Biology, La Trobe University, Bundoora, Australia; 3 Royal Botanic Gardens Victoria, Melbourne, Australia; Universita degli Studi di Pisa, ITALY

## Abstract

The purpose of this study was to identify a reliable DNA extraction protocol to use on 25-year-old powdery mildew specimens from the reference collection VPRI in order to produce high quality sequences suitable to address taxonomic phylogenetic questions. We tested 13 extraction protocols and two library preparation kits and found the combination of the E.Z.N.A.^®^ Forensic DNA kit for DNA extraction and the NuGen Ovation^®^ Ultralow System library preparation kit was the most suitable for this purpose.

## Introduction

Since analysis of the first DNA extractions from museum specimens was made possible through the development of PCR during the mid-1980’s, the use of reference collection specimens for molecular phylogenetic studies has increased and several comparative studies have been published on ancient DNA (aDNA) and PCR amplification methods for plant and fungal specimens [1]. However, there is a knowledge gap regarding obligate biotrophic fungal plant pathogens such as powdery mildew–are we able to extract useable aDNA from powdery mildew on host leaf material for PCR and whole genome Next Generation Sequencing (NGS) applications?

aDNA from preserved specimens is subject to numerous natural processes such as degradation, fragmentation and the deamination of nucleotides, thus reducing the DNA quality and quantity that can be obtained, which reduces the efficacy of PCR [2]. There are many factors that can affect DNA in fungarium specimens such as the age and quality of the sample when collected, the preservation method used, exposure to chemicals and other mutagens, and temperature and relative humidity at which the specimens are stored [3, 4]. Accumulation of these effects results in small DNA fragments (40–400 bp) and low DNA concentration [5]. For aDNA molecular studies the use of whole genome NGS applications has been shown to be more reliable than PCR- amplified gene regions, as the DNA strands are already fragmented, which is preferable for most sequencing platforms, whereas aDNA PCR is limited to shorter targeted gene regions which can reduce the phylogenetic capabilities of these regions [6, 7].

Accessing aDNA from specimens that were preserved primarily with the aim of conserving morphology rather than DNA presents complications when developing methods for the isolation, sequencing and analysis of aDNA [8]. Molecular methodologies have been developed for specific areas of aDNA research such as palaeontology, archaeology, forensics and reference collections of animals, plants and macro-fungi [9]. To date there has been limited research into fungal plant pathogens in reference collections, in particular obligate biotrophs such as powdery mildews (Erysiphales), although studies such as Ristaino [10] and Yoshida et al. [11] investigated the oomycete *Phytophthora infestans* from reference collections, using molecular tools to trace pathogen lineages to understand pathogen evolution.

Currently there are nearly four million algal, fungal and plant specimens held within just over three thousand herbaria and fungaria around the world [12]. The original purpose of these institutions was to provide permanent conservation of plant, algae and fungi collections for morphological analysis enabling research on taxonomy, nomenclature, phylogenetics and the evolution of species [10]. Accurate species identification supports our understanding of worldwide biodiversity; however, there is major discrepancy between the number of species that have been taxonomically classified in collections compared to the estimated species numbers which are still waiting for classification [13]. With the advancement of PCR and affordable sequencing technologies, aDNA molecular phylogenetic studies have seen a surge in the utilisation of herbaria, which have been relatively untouched for molecular analysis to date [14, 15].

The Victorian Plant Pathology Herbarium (VPRI) maintained by Agriculture Victoria at Bundoora, Victoria, Australia, is an example of a reference collection rich in historical collections of fungi. The collection was established in 1890 by Daniel Mc Alpine, the first Consulting Vegetable Pathologist to the Department of Agriculture of Victoria [16]. Specimen-based records of plant pathogens have been collected across Australia and preserved as a reference collection [17]. Currently, VPRI holds ca. 43,000 dried specimens and cultures. Online portals such as the Australian Plant Pest Database [18] utilise specimen-based collections such as VPRI to provide up to date information on current plant pathogen and pest status across Australia [17]. It is therefore vital that reference collections such as VPRI are accurate and up to date with current taxonomic classification.

Powdery mildews are the most commonly occurring plant pathogens worldwide, with ca. 900 species within 16 genera infecting thousands of plant species including ornamental, economically important agricultural and horticultural plants [19, 20,21]. Taxonomic classification of powdery mildews is complex due to the asexual and sexual lifecycles of the fungi. Traditionally, powdery mildew identification was based on morphology and host plant associations with morphological classification relying on specific descriptions of sexual characters to identify to species; however, when the sexual state was absent, identification was largely based on host association [21]. Currently, the use of phylogenetic analysis of nuclear ribosomal DNA has enabled researchers to identify five major lineages of powdery mildew, resolve genera and species delineation, understand powdery mildew evolutionary history, and the evolution of phenotypic characters used for identification purposes [22, 23, 24, 25, 26].

The subject of this study is the apple powdery mildew fungus *Podosphaera leucotricha*, which causes significant yield losses of cultivated apple (*Malus* x *domestica*) around the world [26]. The aim of this study was to test 13 DNA extraction protocols, which include 4 different DNA isolation methods including modifications, for use on preserved powdery mildew specimens from the Victorian Plant Pathology Herbarium (VPRI), in order to obtain DNA suitable for use in species identification PCR and whole genome Next Generation Sequencing applications to provide molecular resolution of preserved powdery mildew specimens.

## Results

Apple (*Malus* spp.) leaves infected with *Podosphaera leucotricha* collected between 1992–1994 were selected from VPRI (Table 1). A 6 mm leaf punch was used to sub-sample from VPRI *P*. *leucotricha* specimens as it was a standardised measure that could be used to compare DNA extraction protocols effectiveness. Infected leaf material was sub-sampled from VPRI *P*. *leucotricha* specimens using a leaf punch to cut leaf sections, which were then used to test 13 DNA extraction protocols. The 13 protocols tested were Chelex^®^100 (CheX), innuPrep Plant DNA (InuP), sodium dodecyl sulphate (SDS), E.Z.N.A.^®^ SP Plant (EznS), DNAzol^™^ (DnaZ), E.Z.N.A.^®^ Forensic DNA (EznF), Qiagen DNeasy^®^ Plant (DneP), Isolate II Plant DNA Lysis buffer PA1 C (IspC), Isolate II Plant DNA Lysis buffer PA2 S (IspS), Wizard^®^ Genomic DNA Purification (WizG), E.Z.N.A.^®^ Plant (EznP), Cetyl trimethyl ammonium bromide (CTAB) and Qiagen DNeasy^®^ Plant plus PTB (DneP+). These protocols were compared on the basis of DNA concentration and quality. PCR, ITS phylogeny and whole genome NGS library preparations were also performed. The DNA samples were expected to comprise *P*. *leucotricha* DNA, host DNA from apple as well as DNA from microorganisms present on the leaf tissue prior to its collection.

**Table 1:** Victorian plant pathology herbarium (VPRI) *P*. *leucotricha* specimens investigated.

VPRI NUMBER	LOCATION	COLLECTION YEAR	HOST SPECIES
**18381**	Queensland, Aust.	1992	*Malus pumila* L.
**18536**	Tasmania, Aust.	1992	*Malus domestica* Borkh.
**18575**	Tasmania, Aust.	1992	*Malus domestica* Borkh.
**19785**	South Australia, Aust.	1994	*Malus sylvestris* Mill.
**19947**	Tasmania, Aust.	1994	*Malus* sp.

### DNA concentration

**Fig 1:**
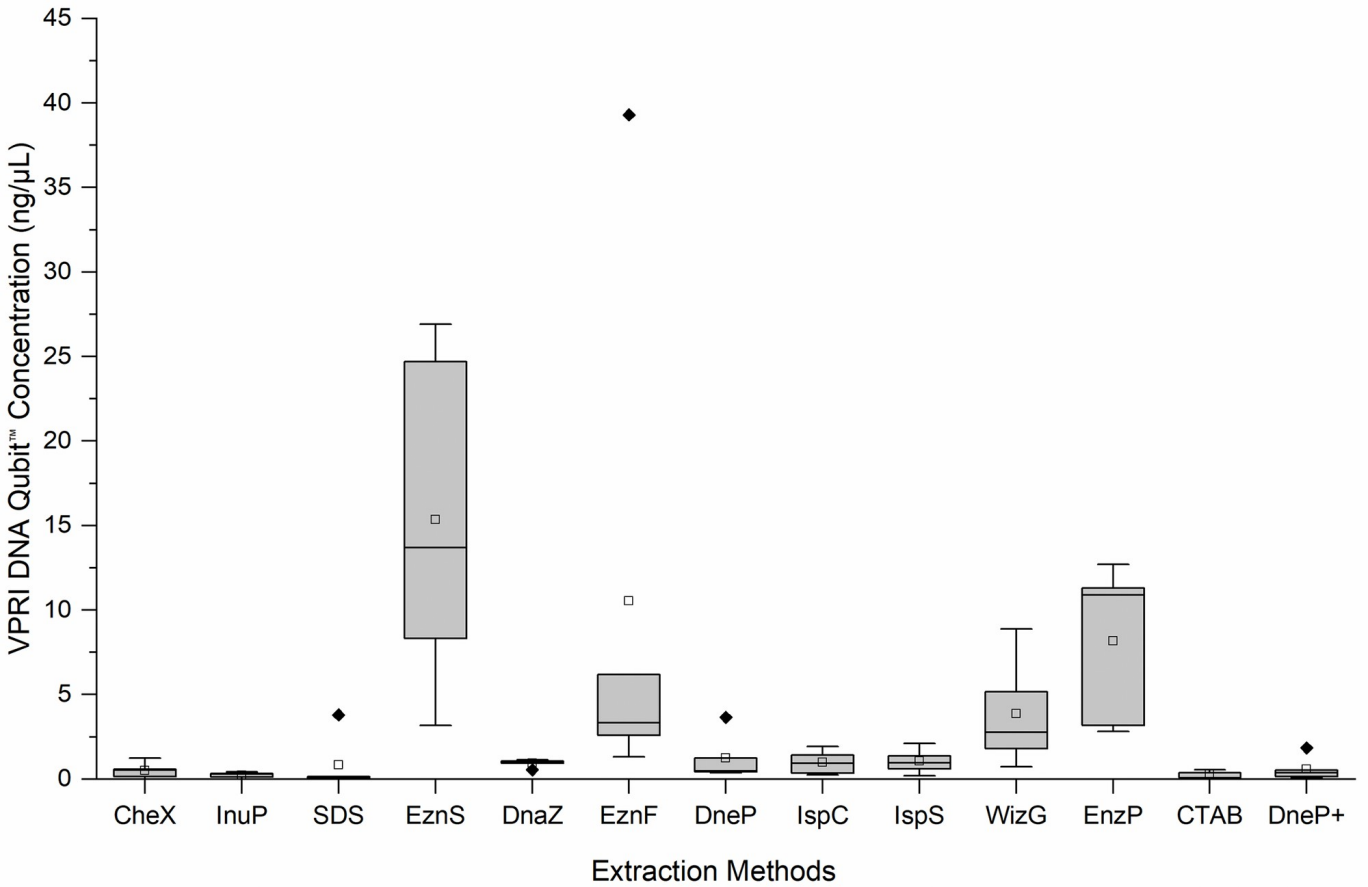
Boxplots of the DNA concentrations (ng/μL) of five victorian plant pathology herbarium (VPRI) apple powdery mildew specimens produced by 13 extraction protocols as measured by Invitrogen Qubit^™^ fluorometer. Median line ^__^; Mean □; Outlier ◆ Extraction method abbreviations: Chelex^®^100 (CheX), innuPrep Plant DNA (InuP), sodium dodecyl sulphate (SDS), E.Z.N.A.^®^ SP Plant (EznS), DNAzol^™^ (DnaZ), E.Z.N.A.^®^ Forensic DNA (EznF), Qiagen DNeasy^®^ Plant (DneP), Isolate II Plant DNA Lysis buffer PA1 C (IspC), Isolate II Plant DNA Lysis buffer PA2 S (IspS), Wizard^®^ Genomic DNA Purification (WizG), E.Z.N.A.^®^ Plant (EznP), Cetyl trimethyl ammonium bromide (CTAB) and Qiagen DNeasy^®^ Plant plus PTB (DneP+).

**Fig 2:**
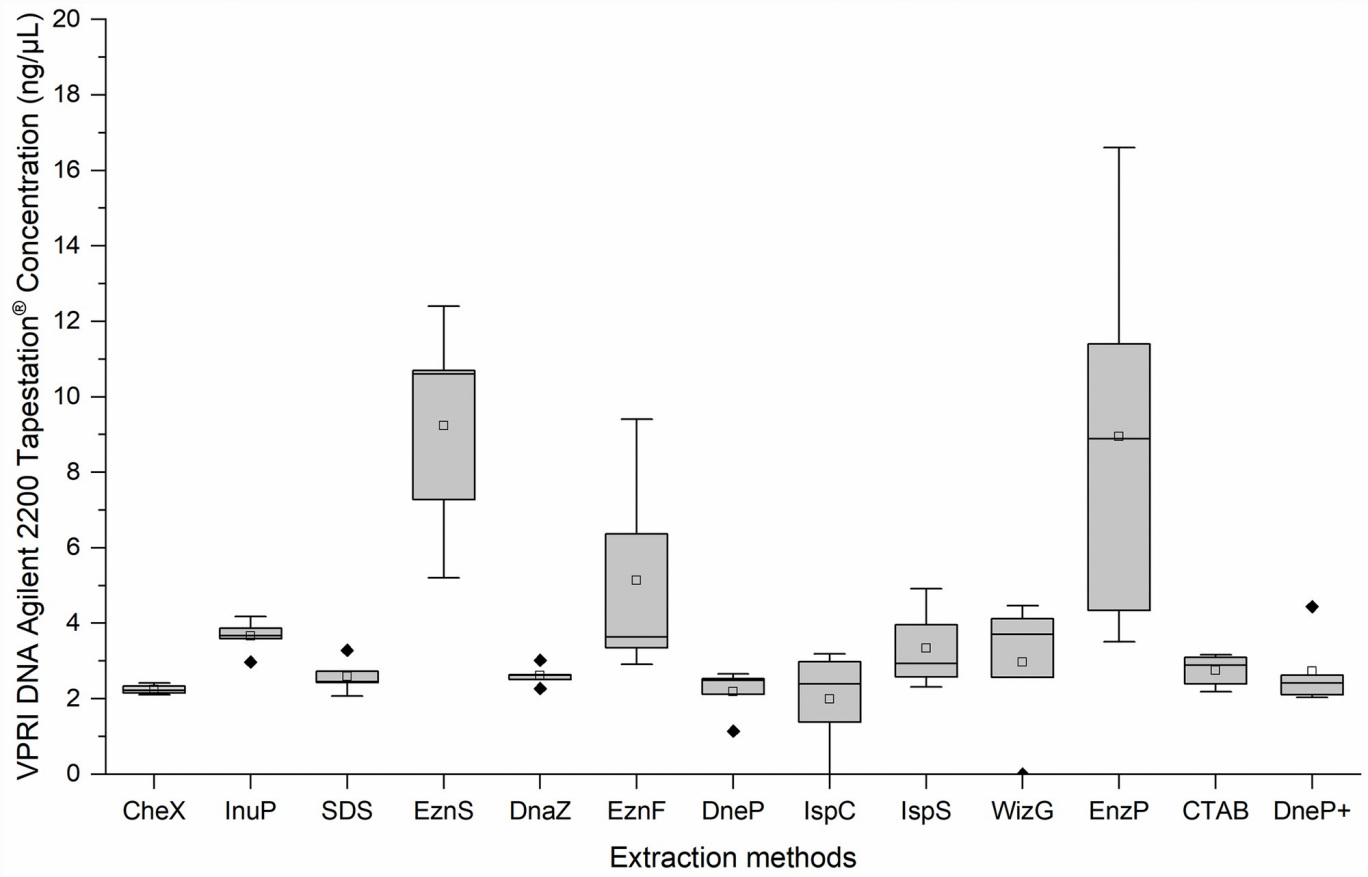
Box plots of the DNA concentrations (ng/μL) of five victorian plant pathology herbarium (VPRI) apple powdery mildew specimens produced by 13 extraction protocols, as measured by agilent 2200 Tapestation^®^ DNA. Median line ^__^; Mean □; Outlier ◆ Extraction method abbreviations: Chelex^®^100 (CheX), innuPrep Plant DNA (InuP), sodium dodecyl sulphate (SDS), E.Z.N.A.^®^ SP Plant (EznS), DNAzol^™^ (DnaZ), E.Z.N.A.^®^ Forensic DNA (EznF), Qiagen DNeasy^®^ Plant (DneP), Isolate II Plant DNA Lysis buffer PA1 C (IspC), Isolate II Plant DNA Lysis buffer PA2 S (IspS), Wizard^®^ Genomic DNA Purification (WizG), E.Z.N.A.^®^ Plant (EznP), Cetyl trimethyl ammonium bromide (CTAB) and Qiagen DNeasy^®^ Plant plus PTB (DneP+).

The 13 different DNA extraction protocols generated variable concentrations of DNA from the five VPRI *P*. *leucotricha* specimens (Table 2). DNA was quantified using two methods, Qubit^™^ fluorometer (Life Technologies, Singapore) and Agilent 2200 TapeStation^®^ (electrophoresis) (Agilent Technologies, Waldbronn, Germany) to eliminate instrument bias analysing poorer quality DNA samples. The two methods gave different estimates of DNA concentration. Qubit^™^ fluorometer consistently estimated lower concentrations than Agilent 2200 TapeStation^®^, except in two instances: EznS and EnzP. Based on Qubit^™^ fluorometer quantification the DNA extraction protocol which produced the highest DNA concentration was EznS (13.7 ng/μL), followed by EznP (10.9 ng/μL) and EznF (3.34 ng/μL) (Fig 1). WizG yielded 2.76 ng/μL; the remaining nine extraction protocols produced DNA concentrations < 1 ng/μL, with SDS producing the least DNA (0.107 ng/μL). Concentrations assessed with the Agilent 2200 TapeStation^®^ followed a similar pattern to the Qubit^™^ fluorometer results with EznS, EznP, WizG and EznF showing the highest concentrations of 10.6 ng/μL, 8.89 ng/μL, 3.71 ng/μL and 3.64 ng/μL, respectively. However, the Agilent 2200 TapeStation^®^ readings for the remaining 9 extraction methods were slightly higher with concentrations ranging between 2.22–2.93 ng/μL (Fig 2).

**Table 2:** Median and range of total DNA concentration (ng/μL) and DNA quality (A260/280) produced by 13 extraction protocols tested on five victorian plant pathology herbarium (VPRI) apple powdery mildew specimens.

Extraction Method	Median (Range) Invitrogen Qubit^™^ DNA Concentration (ng/μL)	Median (Range) Total DNA concentration Invitrogen Qubit^™^ (ng)	Median (Range) Agilent 2200 TapeStation^®^ DNA concentration (ng/μL)	Median (Range) Total DNA concentration Agilent 2200 TapeStation^®^ (ng)	Median (Range) NanoDrop 2000^™^ DNA Quality (A260/280)
CheX	0.52 (0–1.25)	104.4 (0–117.60)	2.22 (2.11–2.42)	444 (422–484)	1.36
InuP	0.30 (0–0.43)	60.4 (0–85.40)	3.67 (2.97–4.18)	734 (594–836)	1.29
SDS	0.11 (0–3.79)	5.4 (2.75–189.50)	2.45 (2.07–3.28)	122.5 (103.5–164)	2.68
EznS	13.7 (3.18–26.9)	1370 (318–2690)	10.6 (5.2–12.40)	1060 (520–1240)	2.17
DnaZ	0.99 (0.5–1.13)	49.9 (27.25–56.50)	2.62 (2.27–3.02)	131 (113.5–151)	2.06
EznF	3.34 (1.33–39.3)	334 (133–3930)	3.64 (2.91–9.41)	364 (291–941)	1.97
DneP	0.46 (0.38–3.66)	46.6 (43.9–366)	2.49 (1.14–2.66)	249 (114–266)	2.05
IspC	0.94 (0.25–1.93)	94.3 (24.6–193)	2.39 (0–3.19)	239 (0–319)	1.92
IspS	0.97 (0.21–2.12)	96.8 (21–138)	2.93 (2.31–4.91)	293 (231–491)	1.83
WizG	2.76 (0.74–8.89)	276 (74–889)	3.8 (2.82–9.71)	380 (282–971)	1.64
EznP	10.9 (2.82–12.7)	1090 (282–1270)	8.89 (3.51–16.60)	889 (351–1140)	1.87
CTAB	0.36 (0–0.55)	27.2 (0–41.25)	2.89 (2.19–3.17)	216.8 (164.3–237.75)	1.88
DneP+	0.36 (0.08–1.84)	36.3 (8.30–184)	2.42 (2.04–4.44)	242 (204–444)	1.33

Extraction method abbreviations: Chelex^®^100 (CheX), innuPrep Plant DNA (InuP), sodium dodecyl sulphate (SDS), E.Z.N.A.^®^ SP Plant (EznS), DNAzol^™^ (DnaZ), E.Z.N.A.^®^ Forensic DNA (EznF), Qiagen DNeasy^®^ Plant (DneP), Isolate II Plant DNA Lysis buffer PA1 C (IspC), Isolate II Plant DNA Lysis buffer PA2 S (IspS), Wizard^®^ Genomic DNA Purification (WizG), E.Z.N.A.^®^ Plant (EznP), Cetyl trimethyl ammonium bromide (CTAB) and Qiagen DNeasy^®^ Plant plus PTB (DneP+).

### DNA quality

**Fig 3:**
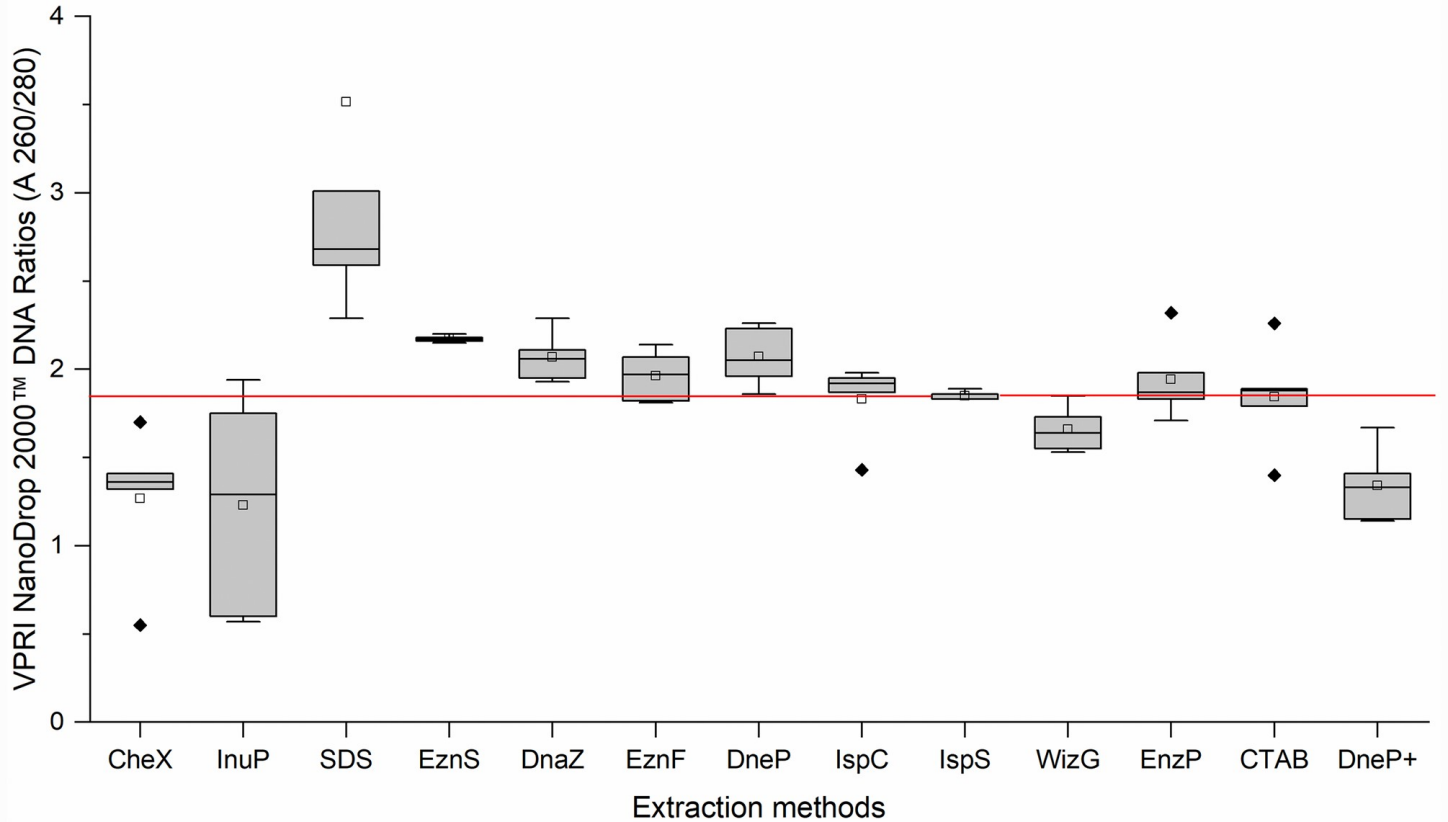
Boxplots of the DNA quality measured by thermo scientific nanodrop 2000^™^ absorbency measurement 260 nm / 280 nm ratios of five victorian plant pathology herbarium (VPRI) apple powdery mildew specimens produced by 13 DNA extraction methods. The red line indicates the desired target absorbency ratio 1.8–1.9. Median line ^__^; Mean □; Outlier ◆ Extraction method abbreviations: Chelex^®^100 (CheX), innuPrep Plant DNA (InuP), sodium dodecyl sulphate (SDS), E.Z.N.A.^®^ SP Plant (EznS), DNAzol^™^ (DnaZ), E.Z.N.A.^®^ Forensic DNA (EznF), Qiagen DNeasy^®^ Plant (DneP), Isolate II Plant DNA Lysis buffer PA1 C (IspC), Isolate II Plant DNA Lysis buffer PA2 S (IspS), Wizard^®^ Genomic DNA Purification (WizG), E.Z.N.A.^®^ Plant (EznP), Cetyl trimethyl ammonium bromide (CTAB) and Qiagen DNeasy^®^ Plant plus PTB (DneP+).

The visual appearance of the extracted DNA varied between methods from colourless to brownish. In all cases the *P*. *leucotricha* DNA was highly fragmented, as indicated by the Agilent 2200 TapeStation^®^ electrophoresis images with fragment sizes between 50 bp– 400 bp. DNA quality was measured using NanoDrop 2000^™^ spectrophotometer (Thermo Fisher Scientific, Wilmington, Delaware, USA) absorbency measurement 260 nm /280 nm ratio; the optimum range indicating high quality DNA is 1.8–1.9 [27]. In general, the silica binding column methods (IspS, IspC, EznP and EznF) produced more consistent DNA quality than the precipitation-based methods (SDS, WizG^®^ and DnaZ). The only method that consistently produced DNA quality within the 1.8–1.9 range was the IspS (Table 2 and Fig 3). The mean DNA quality produced by the EznF, EznP, IspC and CTAB protocols were within the ideal range, but the raw data included outliers either side of the required absorbency ratio. The absorbency ratio of the remaining DNA extraction protocols InuP, SDS, EznS, DnaZ, DneP and DneP+ were outside the required range. Precipitation-based methods produced less consistent DNA quality than the silica binding column methods (Fig 3).

### DNA PCR amplification

Nine published PCR primer sets used in powdery mildew phylogenetic studies were tested for their suitability to amplify powdery mildew DNA extracted by the 13 protocols for species identification (Table 3). The nested PMITS1/PMITS2 and PMITS1/ITS4 [27] was the only set which amplified DNA extracted from all the methods tested (Table 4). Sanger sequencing of the nested PMITS1/PMITS2 and PMITS1/ITS4 PCR VPRI amplicons demonstrated that 64.6% matched GenBank Accession no. KY661076.1, the target *P*. *leucotricha* ITS region, at 98% identity or higher. The remaining amplicons matched other powdery mildew species (12.3%), undetermined fungi (4.6%), the host *Malus* (6.15%) or had failed amplification sequences (12.3%) (Table 4). DNA extracted using protocols CheX, WizG and DnaZ did not amplify well during PCR (Table 4). CheX and WizG resulted in only a single *P*. *leucotricha* ITS amplicon produced and DnaZ resulted in two *P*. *leucotricha* ITS amplicons. Only five amplicons derived from VPRI specimen 19947 were *P*. *leucotricha* ITS. There is reportedly no correlation between herbarium DNA concentration and PCR amplification success [28], yet the presence of other fungi and plant host DNA resulted in preferential amplification over *P*. *leucotricha* DNA in 21.5% of the PCR reactions during this study.

**Table 3:** Published primer sets tested on all DNA extracted from five VPRI *P*. *leucotricha* specimens from 1992–1994 (total = 65 reactions per extraction method), and the percentage of *P*. *leucotricha* amplicons generated per primer set.

PRIMER	TARGET GENE REGION	EXPECTED AMPLICON SIZE (BP)	REFERENCE	SUCCESSFUL AMPLICON %
**ITS1F/ITS2**	ITS 1	230	White et al. 1990 [29]	1.5%
**PMITS1F/PMITS2**	ITS 1, 5.8S, ITS 2	700	Cunnington et al. 2003 [30]	6.2%
**PM5F/PM6**	ITS 1, 5.8S, ITS 2	400	Takamatsu and Kano 2001 [31]	10.8%
**PMITS1F/ITS4**	ITS 1, 5.8S, ITS 2	600	White et al. 1990 [29]	56.9%
**NESTED PMITS1/2 PMITS1/ITS4**	ITS 1, 5.8S, ITS 2	550	Cunnington, Lawrie and Pascoe 2004 [32]	69.2%
**PMBT1AF/BTMYCR**	β-tubulin	400	Feau et al. 2011 [33]	55.4%
**CHS79F/CHS354**	Chitin Synthase	300	Carbone and Kohn 1999 [34]	60.0%
**MCM7AF/MCM7A**	Mini chromosome Maintenance Complex Component 7	550	Ellingham, David and Culham 2019 [35]	0.0%
**MCM7SEQF/MCM7SEQ**	Mini chromosome Maintenance Complex Component 7	550	Ellingham, David and Culham 2019 [35]	0.0%

**Table 4:** Nested PMITS1/PMITS2 and PMITS1/ITS4 PCR results for VPRI apple powdery mildew *P*. *leucotricha* specimens.

VPRI #	18381	18536	18575	19785	19947
**CheX**	*Golovinomyces*	*P*. *leucotricha*		*Golovinomyces*	Undetermined fungi
**InuP**	*P*. *leucotricha*	*P*. *leucotricha*	*P*. *leucotricha*	*Erysiphe*	Undetermined fungi
**SDS**	*P*. *leucotricha*	*P*. *leucotricha*	*P*. *leucotricha*		
**EznS**	*P*. *leucotricha*	*P*. *leucotricha*	*P*. *leucotricha*	*P*. *leucotricha*	*Malus* ITS
**DnaZ**	*Podosphaera*	*Golovinomyces*	*Podosphaera*	*P*. *leucotricha*	Undetermined fungi
**EznF**	*P*. *leucotricha*	*P*. *leucotricha*	*P*. *leucotricha*	*P*. *leucotricha*	Malus ITS
**DneP**	*P*. *leucotricha*	*P*. *leucotricha*	*P*. *leucotricha*	*P*. *leucotricha*	
**IspC**	*P*. *leucotricha*	*P*. *leucotricha*	*P*. *leucotricha*	*P*. *leucotricha*	*Malus* ITS
**IspS**	*P*. *leucotricha*	*P*. *leucotricha*	*P*. *leucotricha*	*P*. *leucotricha*	
**WizG**	*P*. *leucotricha*	*P*. *leucotricha*	*P*. *leucotricha*	*P*. *leucotricha*	*Malus* ITS
**EznP**	*P*. *leucotricha*	*P*. *leucotricha*			*P*. *leucotricha*
**CTAB**	*P*. *leucotricha*	*P*. *leucotricha*	*P*. *leucotricha*	*Podosphaera*	
**DneP+**	*P*. *leucotricha*	*P*. *leucotricha*	*P*. *leucotricha*	*Podosphaera*	*P*. *leucotricha*

Light grey: *P*. *leucotricha* (matched to GenBank Accession no. KY661076.1), Dark Grey: other powdery mildew, White: *Malus*, shaded: undetermined fungi and Black: failed amplification. Extraction method abbreviations: Chelex^®^100 (CheX), innuPrep Plant DNA (InuP), sodium dodecyl sulphate (SDS), E.Z.N.A.^®^ SP Plant (EznS), DNAzol^™^ (DnaZ), E.Z.N.A.^®^ Forensic DNA (EznF), Qiagen DNeasy^®^ Plant (DneP), Isolate II Plant DNA Lysis buffer PA1 C (IspC), Isolate II Plant DNA Lysis buffer PA2 S (IspS), Wizard^®^ Genomic DNA Purification (WizG), E.Z.N.A.^®^ Plant (EznP), Cetyl trimethyl ammonium bromide (CTAB) and Qiagen DNeasy^®^ Plant plus PTB (DneP+).

### Phylogeny

**Fig 4:**
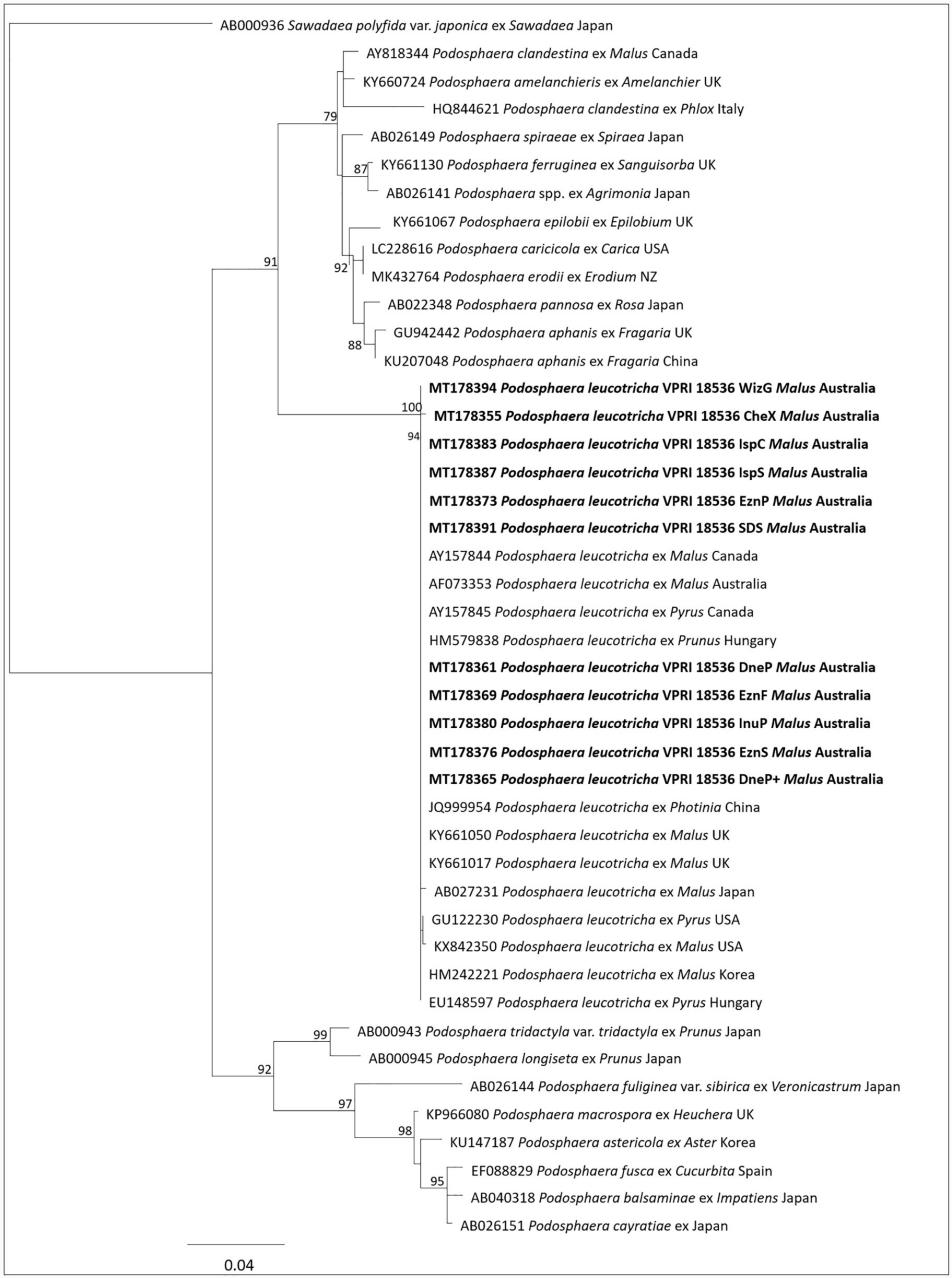
A Maximum Likelihood (ML) phylogenetic tree of the *podosphaera leucotricha* combined dataset of VPRI 18536 *podosphaera leucotricha* nested ITS PCR amplicons (bold) and other *podosphaera* species ITS sequence data taken from GenBank. This tree was generated using rDNA ITS1-5.8S-ITS2 sequences in PhyML with the GTR substitution model showing the relationship between VPRI *P*. *leucotricha* and GenBank *P*. *leucotricha* accession sequences. Bootstrap (BS) values of >70% were taken from 1000 replications and are shown on the respective branches and the scale bar equals 5 changes per 100 bases.

Thirteen sequences were derived from *P*. *leucotricha* VPRI 18536 by nested PCR and Sanger sequencing, one from each extraction method. Two sequences were excluded from the analysis: the sequence generated from the extraction method DnaZ identified a contaminant (*Golovinomyces*) and the sequence generated from the extraction method CTAB was ambiguous and could not be aligned with the others. The maximum likelihood anaylsis (PhyML) including the other 11 VPRI 18536 sequences confirmed that VPRI 18536 was *P*. *leucotricha* (Fig 4). Sequences from other *Podosphaera* species sequences downloaded from GenBank were included and clustered in two separate clades. The first clade (bootstrap support 90.9%) consisted of *P*. *clandestina*, *P*. *amelanchieris*, *P*. *leucotricha*, *P*. *ferruginea*, *P*. *pannosa*, *P*. *spiraeae*, *P*. *pannosa*, *P*. *aphanis*, *P*. *epilobii*, *P*. *erodii*, *P*. *caricicola* and an un-named *Podosphaera* collection from Japan. The second clade (bootstrap support 92.4%) consisted of sequences from *P*. *tridactyla* var. *tridactyla*, *P*. *longiseta*, *P*. *fuliginea* var. *sibirica*, *P*. *astericola*, *P*. *macrospora*, *P*. *balsaminae*, *P*. *cayratiae* and *P*. *fusca*. Within the first clade, sequences from *P*. *leucotricha* formed a tight clade (bootstrap support 99.8%) at the base of the clade. The sequences from VPRI 18536 and GenBank sequences of *P*. *leucotricha* from Australia, China, Korea, Japan, Hungary, UK and USA clustered together. Among *P*. *leucotricha* sequences, there were several base pair differences between some of the collections. Among the sequences of VPRI 18536 derived from different extraction methods, there was also a small amount of variation. In the alignment, sequences derived from extraction methods IspC, IspS and SDS had one missing base (T) at position 520 compared to sequences from the other nine extraction methods. CheX had one different base (G) at position 469 and IspC had one different base (T) at position 551.

### Next generation sequencing VPRI 18536

Two library preparation kits, Illumina Nextera XT^®^ (San Diego, California, USA) and NuGen Ovation^®^ ultralow System V2 (San Carlos, California, USA), were compared using DNA extracts from the 13 DNA extraction protocols applied to VPRI specimen 18536. The genomic libraries were quantified by Promega Quantus^™^ fluorometer and Agilent 2200 TapeStation^®^ and submitted for Illumina^®^ HiSeq 3000 sequencing, except for DneP+ Illumina Nextera XT^®^ and NuGen Ovation^®^ ultralow System V2 libraries which were sequenced using Illumina^®^ MiSeq V3 due to the Illumina^®^ HiSeq 3000 being unavailable. Gydle programs were used for sequence read processing (https://www.gydle.com/). *P*. *leucotricha* VPRI sequences were filtered for quality using nuclear filter with a minimum score of 20, minimum length was set at 50 bp, and total length of 100 bp. Mapping to reference sequences was performed by nuclear search with sequence length set at 100, sensitivity set at 25, kmer 13 and mismatches set at 0. Six reference scaffolds were used for sequence read mapping: *P*. *leucotricha* ITS (GenBank accession number KX842350.1), *P*. *leucotricha* mitochondria and rRNA (S3 File) and host DNA *Malus* chloroplast (GenBank accession number KU851961) and *Malus* mitochondria (GenBank accession number FR714868.1). The mapped reads were used for creating viewable gym files using Gydle Gym-build. These files were visualised in Vision 2.6.24 (Gydle, Canada). Raw and QC read numbers were taken from the nuclear results before and after trimming. The mapped read numbers were obtained from the gym files displayed in the Vision program. Total read number of mapped sequences reads and mapped read percentages to total QC reads are presented in Tables 5 and 6.

**Table 5:** Illumina Nextera XT^®^ VPRI *P*. *leucotricha* sequencing alignment results taken from vision alignment.

	Reads Raw	Reads Passed QC	PM ITS Raw	PM ITS	PM Mito1 Raw	PM Mito1	PM Mito2 Raw	PM Mito2	PM rRNA Raw	PM rRNA	*Malus* Mito Raw	*Malus* Mito	*Malus* Chloro Raw	*Malus* Chloro
**CheX**	3,301,690	2,125,386	142	0.01%	2,395	0.11%	1,603	0.08%	3,382	0.16%	1,915	0.09%	2,956	0.14%
**InuP**	7,172,861	5,266,236	837	0.02%	13,406	0.26%	10,011	0.19%	5,696	0.11%	4,223	0.08%	5,210	0.10%
**SDS**	6,913,188	3,950,749	477	0.01%	7,518	0.19%	5,551	0.14%	1,805	0.05%	5,479	0.14%	6,237	0.16%
**EznS**	919,376	746,040	80	0.01%	1,544	0.21%	1,120	0.15%	806	0.11%	59,470	7.97%	164,254	22.02%
**DnaZ**	6,236,108	2,948,446	21	0.00%	457	0.02%	304	0.01%	72	0.00%	3,060	0.10%	10,743	0.36%
**EznF**	5,911,120	4,799,780	408	0.01%	1,773	0.04%	1,353	0.03%	1,849	0.04%	20,072	0.42%	24,437	0.51%
**DneP**	5,999,284	3,837,027	88	0.00%	403	0.01%	350	0.01%	494	0.01%	7,692	0.20%	10,775	0.28%
**IspC**	241,089	195,337	99	0.05%	635	0.33%	412	0.21%	1,013	0.52%	23,867	12.22%	29,683	15.20%
**IspS**	140,095	110,579	16	0.01%	87	0.08%	71	0.06%	334	0.30%	5,711	5.17%	5,737	5.19%
**WizG**	4,517,829	3,631,938	72	0.00%	161	0.00%	120	0.00%	1,657	0.05%	14,064	0.39%	25,543	0.70%
**EznP**	6,256,598	5,063,754	142	0.00%	481	0.01%	360	0.01%	1,494	0.03%	35,642	0.70%	67,163	1.33%
**CTAB**	6,357,620	3,373,175	46	0.00%	692	0.02%	469	0.01%	1,230	0.04%	8,304	0.25%	5,959	0.18%
**DneP+**	640,221	572,874	9	0.00%	140	0.02%	75	0.01%	66	0.01%	1,337	0.23%	2,376	0.41%

Total raw reads, QC reads and mapped raw reads and percentage of aligned sequence reads to reference genes: *P*. *leucotricha* ITS (PM ITS), *P*. *leucotricha* mitochondria 1 and 2 scaffolds (PM Mito 1/2), *P*. *leucotricha* rRNA scaffold (PM rRNA)s, *Malus* mitochondria and *Malus* chloroplast genomes. Extraction method abbreviations: Chelex^®^100 (CheX), innuPrep Plant DNA (InuP), sodium dodecyl sulphate (SDS), E.Z.N.A.^®^ SP Plant (EznS), DNAzol^™^ (DnaZ), E.Z.N.A.^®^ Forensic DNA (EznF), Qiagen DNeasy^®^ Plant (DneP), Isolate II Plant DNA Lysis buffer PA1 C (IspC), Isolate II Plant DNA Lysis buffer PA2 S (IspS), Wizard^®^ Genomic DNA Purification (WizG), E.Z.N.A.^®^ Plant (EznP), Cetyl trimethyl ammonium bromide (CTAB) and Qiagen DNeasy^®^ Plant plus PTB (DneP+).

**Table 6:** NuGen Ovation^®^ Ultralow system V2 VPRI *P*. *leucotricha* sequencing alignment results taken from vision alignment.

	Reads Raw	Reads Passed QC	PM ITS Raw	PM ITS	PM Mito1 Raw	PM Mito1	PM Mito2 Raw	PM Mito2	PM rRNA Raw	PM rRNA	*Malus* Mito Raw	*Malus* Mito	*Malus* Chloro Raw	*Malus* Chloro
**CheX**	6,269,905	5,725,643	26	0.00%	2,038	0.04%	1,352	0.02%	856	0.02%	247	0.00%	487	0.01%
**InuP**	6,333,104	5,828,307	1471	0.03%	112,097	1.92%	79,256	1.36%	14,398	0.25%	10,338	0.18%	24,331	0.42%
**SDS**	2,011,540	1,817,078	741	0.04%	43,443	2.39%	30,016	1.65%	6,494	0.36%	7,275	0.40%	13,892	0.77%
**EznS**	4,782,426	4,283,523	80	0.00%	1,544	0.04%	1,120	0.03%	806	0.02%	59,470	1.39%	164,254	3.84%
**DnaZ**	2,587,961	2,327,882	95	0.00%	6,200	0.27%	4,067	0.18%	217	0.01%	7,911	0.34%	66,157	2.84%
**EznF**	38,206,866	34,654,454	3291	0.01%	90,080	0.26%	63,198	0.18%	15,598	0.05%	268,897	0.78%	543,306	1.57%
**DneP**	6,292,079	5,726,089	211	0.00%	5,821	0.10%	3,959	0.07%	1,086	0.02%	24,778	0.43%	62,759	1.10%
**IspC**	3,562,523	3,137,760	99	0.00%	635	0.02%	412	0.01%	1,013	0.03%	23,867	0.76%	29,683	0.75%
**IspS**	846,555	758,044	16	0.00%	87	0.01%	71	0.01%	334	0.04%	5,711	0.75%	5,737	0.76%
**WizG**	7,642,944	6,936,414	233	0.00%	2,523	0.04%	1,811	0.03%	3,818	0.06%	56,928	0.82%	142,744	2.06%
**EznP**	3,352,226	2,795,537	45	0.00%	552	0.02%	454	0.02%	185	0.01%	13,004	0.47%	38,916	1.39%
**CTAB**	2,446,075	2,211,347	101	0.01%	4,613	0.21%	3,254	0.15%	3,363	0.15%	14,643	0.66%	19,590	0.89%
**DneP+**	5,757,754	5,145,556	201	0.00%	4,499	0.09%	3,255	0.06%	1,054	0.02%	22,404	0.44%	46,878	0.91%

Total raw reads, QC reads and mapped raw and percentage of aligned sequence reads to reference genes: *P*. *leucotricha* ITS (PM ITS), *P*. *leucotricha* mitochondria 1 and 2 scaffolds (PM Mito 1/2), *P*. *leucotricha* rRNA scaffold (PM rRNA), *Malus* mitochondria and *Malus* chloroplast genomes. Extraction method abbreviations: Chelex^®^100 (CheX), innuPrep Plant DNA (InuP), sodium dodecyl sulphate (SDS), E.Z.N.A.^®^ SP Plant (EznS), DNAzol^™^ (DnaZ), E.Z.N.A.^®^ Forensic DNA (EznF), Qiagen DNeasy^®^ Plant (DneP), Isolate II Plant DNA Lysis buffer PA1 C (IspC), Isolate II Plant DNA Lysis buffer PA2 S (IspS), Wizard^®^ Genomic DNA Purification (WizG), E.Z.N.A.^®^ Plant (EznP), Cetyl trimethyl ammonium bromide (CTAB) and Qiagen DNeasy^®^ Plant plus PTB (DneP+).

There was a difference in the numbers of raw and quality-controlled (QC) sequence reads generated by each library kit. The Illumina Nextera XT^®^ libraries highest QC reads were from the extraction protocol InuP (5,266,236) followed by EznP (5,063,754) and EznF (4,799,780) (Table 5), whereas the NuGen Ovation^®^ Ultralow System V2 libraries from the extraction protocol EznF generated the highest number of QC reads (34,654,454) followed by WizG (6,936,414) and InuP (5,828,307) (Table 6). The percentage of reads aligned to *P*. *leucotricha* gene regions were < 1% in most cases for both Illumina Nextera XT^®^ and NuGen Ovation^®^ Ultralow System V2. These percentages taken from the total QC sequence reads are comparable with the number of total QC reads relative to the percentage of aligned ITS sequences and show that NuGen Ovation^®^ Ultralow System V2 libraries provided a higher percentage of *P*. *leucotricha* sequence reads.

**Fig 5:**
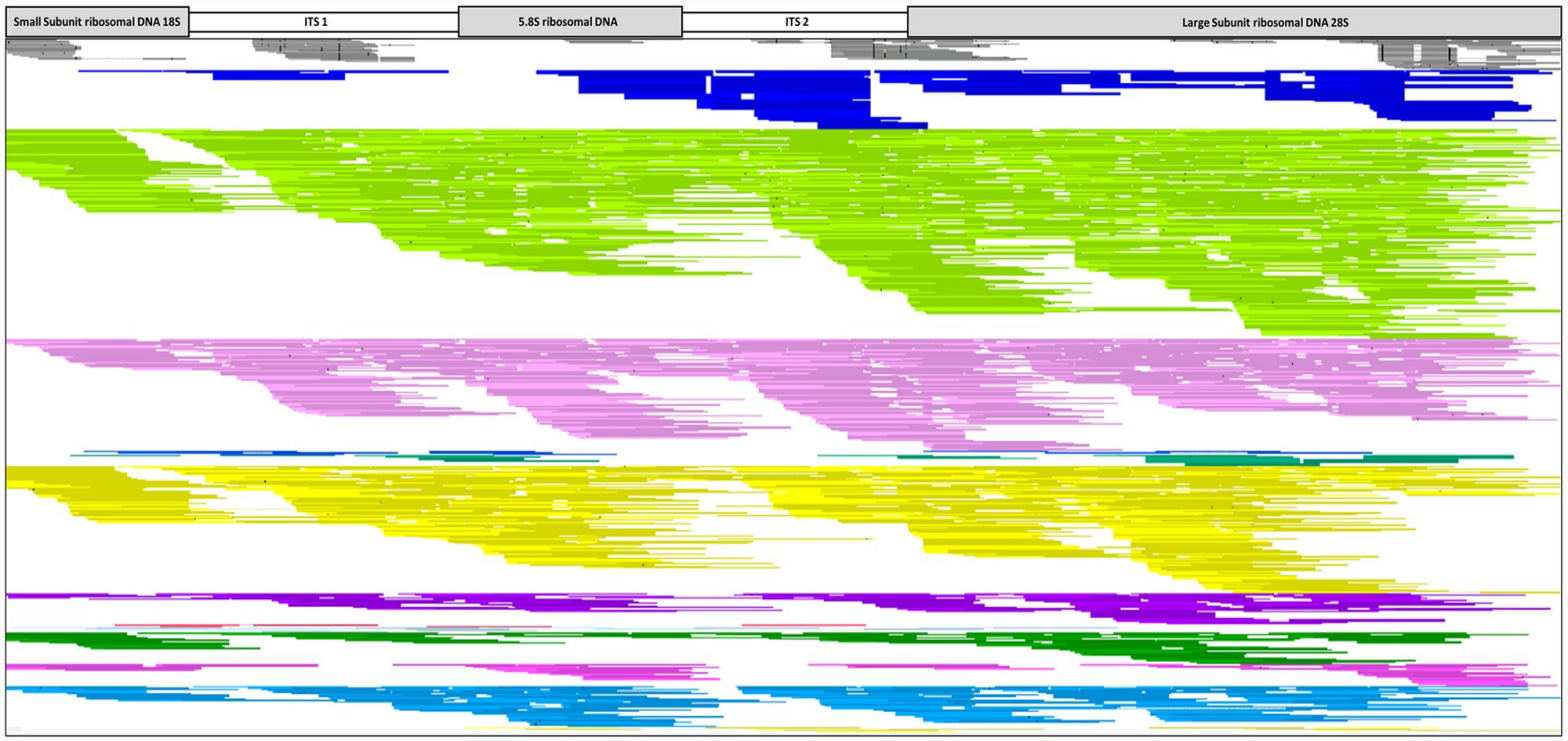
Vision 2.6.24 image of 13 illumina nextera XT^®^ VPRI 18536 DNA extraction protocol libraries mapped to *P*. *leucotricha* ITS (GenBank accession no. KX842350.1) including *P*. *tridactyla* as an outgroup for comparison. Continuous unbroken lines represent sequence reads that completely align to the reference sequence. Gaps in the alignment indicates no mapping sequence reads, and SNPs between the mapped read and the reference are represented as black bars. Colour code: Grey- *P*. *tridactyla*, Dark Blue- CheX (Chelex^®^100), Light Green- InuP (innuPrep Plant DNA), Light Pink- SDS (sodium dodecyl sulphate), Blue- EznS (E.Z.N.A.^®^ SP Plant), Green- DnaZ (DNAzol^™^), Yellow- EznF (E.Z.N.A.^®^ Forensic DNA), Purple- DneP (Qiagen DNeasy^®^ Plant), Red- IspC (Isolate II Plant DNA Lysis buffer PA1 C), Light Blue- IspS (Isolate II Plant DNA Lysis buffer PA2 S), Dark Green- WizG (Wizard^®^ Genomic DNA Purification), Light Blue- EznP (E.Z.N.A.^®^ Plant), Dark Pink- CTAB (Cetyl trimethyl ammonium bromide) and Light Yellow- DneP+ (Qiagen DNeasy^®^ Plant plus PTB).

**Fig 6:**
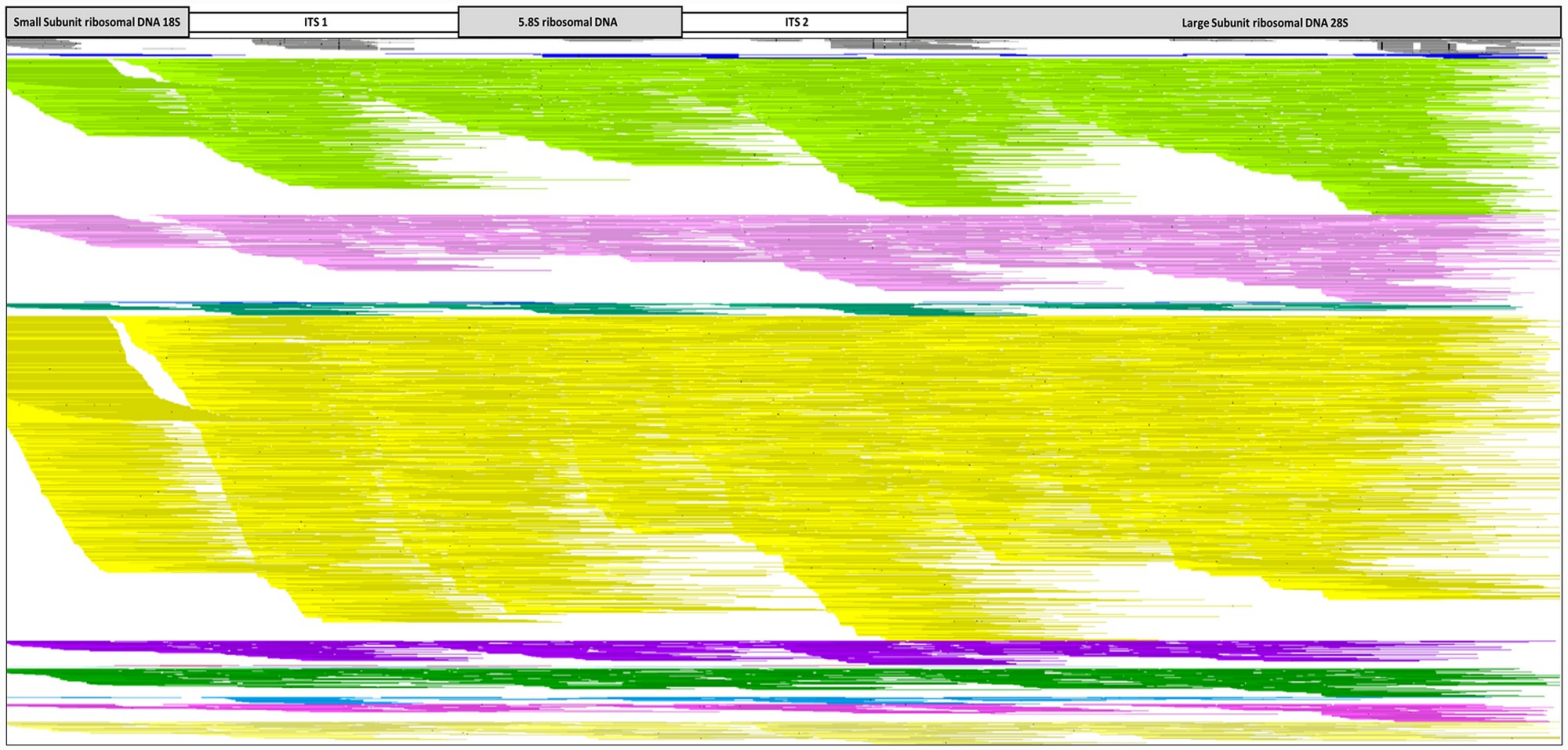
Vision 2.6.24 image of 13 NuGen Ovation^®^ ultralow system V2 VPRI 18536 DNA extraction protocol libraries mapped to *P*. *leucotricha* ITS (GenBank accession no. KX842350.1) including *P*. *tridactyla* as an outgroup for comparison. Continuous unbroken lines represent sequence reads that completely align to the reference sequence. Gaps in the alignment indicates no mapping sequence reads, and SNPs between the mapped read and the reference are represented as black bars. Colour code: Grey- *P*. *tridactyla*, Dark Blue- CheX (Chelex^®^100), Light Green- InuP (innuPrep Plant DNA), Light Pink- SDS (sodium dodecyl sulphate), Blue- EznS (E.Z.N.A.^®^ SP Plant), Green- DnaZ (DNAzol^™^), Yellow- EznF (E.Z.N.A.^®^ Forensic DNA), Purple- DneP (Qiagen DNeasy^®^ Plant), Red- IspC (Isolate II Plant DNA Lysis buffer PA1 C), Light Blue- IspS (Isolate II Plant DNA Lysis buffer PA2 S), Dark Green- WizG (Wizard^®^ Genomic DNA Purification), Light Blue- EznP (E.Z.N.A.^®^ Plant), Dark Pink- CTAB (Cetyl trimethyl ammonium bromide) and Light Yellow- DneP+ (Qiagen DNeasy^®^ Plant plus PTB).

The mapping results of the two library kits highlighted that NuGen Ovation^®^ Ultralow System V2 libraries performed better than Illumina Nextera XT^®^ libraries with higher numbers of *P*. *leucotricha* reads mapping to the references. For both libraries total mapped read numbers obtained from the six reference Gym-files highlighted the nominal amount of *P*. *leucotricha* DNA sequences which mapped to the references compared to the total QC reads, most notably the ITS gene region (Tables 5 and 6). However, the number of ITS reads which mapped to the *P*. *leucotricha* ITS reference was higher in those protocols that generated the higher number of QC reads as shown in Figs 5 and 6. The Illumina Nextera XT^®^ libraries with the highest number of mapped ITS reads were InuP (837), SDS (477) and EznF (408) (Table 5). The Illumina Nextera XT^®^ ITS Vision image shows the overall reduced number of aligned ITS reads and reduced sequencing coverage across all DNA extraction methods, indicated by gaps in the alignment (Fig 5). The Vision image shows that InuP, SDS and EznF sequentially have the most coverage of the *P*. *leucotricha* ITS regions. This differs from the percentage of ITS reads from the total QC reads which shows that IspC has the highest mapped ITS percentage compared to the remaining 12 protocols; this is due to IspC having the second lowest QC read total (Table 5).

The NuGen Ovation^®^ Ultralow System V2 libraries which produced the highest mapped ITS reads were extraction protocols EnzF (3291), InuP (1471) and SDS (741) (Table 6). The aligned ITS Vision image for the 13 NuGen Ovation^®^ Ultralow System V2 libraries shows the overall increase in aligned ITS sequence reads and increased ITS gene regions coverage, indicated by the minimal gaps across the alignment (Fig 6). The NuGen Ovation^®^ Ultralow System V2 Vision image visually highlights the superior sequencing results of EznF extraction kit compared to the 12 other protocols tested. It shows that higher total QC read numbers resulted in better coverage of the ITS region (Fig 6). The alignment image shows that EznF, InuP and SDS gave the best coverage of the ITS; this differed from the percentage of aligned ITS reads, which showed SDS as the highest, followed by InuP and EznF (Table 6).

The *P*. *leucotricha* mitochondria and rRNA mapping for both library kits tested showed an increase in the numbers of reads which mapped to these references (Tables 5 and 6). For Illumina Nextera XT^®^ libraries protocol InuP had the highest number of mapped reads for all three *P*. *leucotricha* references Mito1 (13,406), Mito2 (10,011) and rRNA (5696), closely followed by protocols SDS and EnzF (Table 5). Plant host mapping was completed using *Malus* mitochondria and chloroplast. The Illumina Nextera XT^®^ libraries extraction protocol EznS had the highest number of mapped reads to these references with 59,470 reads aligning to the mitochondria reference and 164,254 reads aligned to the chloroplast reference (Table 5). The NuGen Ovation^®^ Ultralow System V2 libraries mapping results showed InuP with the highest number of reads mapped to Mito1 (112,097) and Mito2 (79,256) but EznF had the greatest number of aligned reads for rRNA with 15,598 (Table 6). EznF also had the highest number of mapped reads for the plant host references *Malus* mitochondria and chloroplast with 268,897 and 543,306 respectively.

## Discussion

This is the first study to systematically compare different DNA extraction methods and sequencing capabilities on powdery mildew reference collection specimens and highlights the difficulties to extract, isolate and sequence powdery mildew DNA from preserved leaf material. We found that DNA concentration was more important than DNA quality for molecular applications of DNA from powdery mildew plant pathogens as the increased DNA concentration will provide greater chance of containing the target DNA. We also found that PCR barcoding and Sanger sequencing were not suitable for identifying preserved powdery mildew specimens due to the variability of correct fungal DNA amplification, and that NGS was more applicable for molecular analysis of preserved powdery mildew specimens. This result is not consistent with studies by Särkinen et al. [36] who compared DNA extraction methods on herbarium plant specimens and showed DNA purity was the most important factor for PCR amplification of barcode regions. The difference between the two studies is likely to be due to the different end points. Särkinen et al. [36] focused on PCR applications for herbarium plant DNA, in which plant DNA is by far the dominant DNA type extracted from the specimens. Therefore, DNA purity improves the success rate of subsequent PCR amplification. In our study, the target powdery mildew DNA was a tiny proportion of the total DNA extracted from the specimen and therefore higher total DNA concentrations increased the chance of sequencing powdery mildew DNA using NGS.

Although the methods tested in this study successfully obtained powdery mildew DNA from the VPRI specimens, the DNA samples were very low yielding, heavily fragmented and degraded, consistent with previous reports when using herbarium specimens [15, 28, 37, 38, 39]. More selective sampling from preserved powdery mildew specimens, by reducing the amount of leaf material sampled, could improve the DNA concentration of the target fungus. None of the 13 DNA extraction methods tested provided both high quality and high concentration. Regarding concentration, this study found that the better performing protocols were commercially available kits that used silica binding columns such as InuP, DneP, IspC and IspS rather than precipitation (WizG or CTAB) or chelating based methods (CheX). This result suggests an increased retention of fragmented DNA in column-based DNA extractions over precipitation methods. For the recovery of powdery mildew DNA from a mixed preserved DNA sample the EznF, EznP and EznS kits produced more DNA from VPRI specimens compared with the other silica binding kits. Previous studies on herbarium DNA extractions highlighted CTAB or commercially available kits such as Qiagen DNeasy^®^ Plant to be reliable for herbarium DNA extractions [15]. However, this study has shown that for older preserved powdery mildew specimens, these methods did not produce comparative concentrations of fungal DNA compared to the EznF, EznP and EznS extraction kits.

DNA quality from preserved plant and fungal specimens is often compromised due to contaminants such as plant-based PCR inhibitors and microflora present on the specimen at time of collection [40], which can confound PCR amplicon sequencing [36, 15]. The IspS extraction method produced the best quality DNA, although it produced one of the lowest DNA concentrations. Several protocols produced less than optimal DNA quality (IspC, EznF, EznP, CTAB and EznS), and it was decided that further DNA cleaning steps during extraction to improve DNA quality would be detrimental, based on the low DNA sample concentrations that were obtained from the VPRI specimens. Further cleaning would potentially reduce the DNA concentration below that required for NGS library preparation, given most of the extraction protocols yielded < 1 ng/μL of DNA [28, 41].

Most herbarium phylogenetic studies to date have relied on the analysis of PCR products for species identification but a major factor that strongly influences preserved specimen PCR success is target amplicon size. The application of PCR-based approaches for phylogenetic studies using aDNA is problematic; aDNA can be highly fragmented and there are few small loci (less than 500 bp) which are phylogenetically informative that can be used [36, 42]. This study tested nine published primer sets for potential gene regions, that could be utilised as barcodes for powdery mildew molecular species identification [29, 30, 31, 32, 33, 34, 35]. These primer sets proved to be unsuitable in most cases as the target gene regions are too long in length (greater than 550 bp) for the amplification of the fragmented aDNA. The tested primers also showed inconsistent amplification across the five VPRI samples. The poor PCR results in the present study highlight the difficult nature of working with preserved plant pathogen specimens.

For this study we found that the nested PCR primers PMITS1/PMITS2 and PMITS1/ITS4 provided the most consistent amplification results for VPRI powdery mildew DNA. Currently for the construction of powdery mildew phylogenies, ITS is the most commonly used gene region, although it does not always provide adequate resolution between closely related species [43]. However, for *P*. *leucotricha* ITS was sufficient to demonstrate that the VPRI 18536 specimens were correctly identified (Fig 4). Molecular sequence data produced for species identification must be specific and reliable for accurate identifications, but many of the common fungal ITS primers are hindered by multiple types of biases, such as length bias, taxonomic bias and primer mismatch bias [44]. Together with fungal primer bias low DNA concentrations and variable DNA quality from fungarium DNA reduces PCR capabilities for molecular identification.

An alternative method to overcome the limitations of PCR approaches with preserved fungal specimens is to use a sequencing platform that is designed for fragmented DNA [42]. Whole genome NGS requires DNA strand lengths less than 500 bp and it was hypothesised that NGS would be suitable for fungarium DNA, which is already naturally fragmented. However, library preparation kits developed for fresh DNA have a fragmentation step incorporated into the protocol to create uniform DNA fragments. In this study, we compared two different library preparation kits to investigate whether DNA from preserved specimens would generate better sequence data using a kit specifically designed for low quality and fragmented DNA (NuGen Ovation^®^ Ultralow System V2) over a kit for fresh DNA (Illumina Nextera XT^®^).

Analysis of the sequencing data for VPRI *P*. *leucotricha* DNA showed the NuGen Ovation^®^ Ultralow System V2 kit outperformed Illumina Nextera XT^®^ in library concentration, read quality and generation of reads that aligned to *P*. *leucotricha* reference sequences. The results demonstrated the ability to generate sequence data from unrepaired aDNA of VPRI *P*. *leucotricha* that could be confidently aligned to *P*. *leucotricha* reference scaffolds. However, a greater depth of sequencing is required to generate whole genome phylogenetic data.

When comparing the library kits, the NuGen Ovation^®^ Ultralow System V2 outperformed the Illumina Nextera XT^®^ consistently in both raw and QC reads except for SDS, DnaZ, EznP and CTAB, which yielded higher Illumina Nextera XT^®^ raw and QC reads (Tables 5 and 6). Illumina Nextera XT^®^ requires excellent quality DNA for library preparation whereas NuGen Ovation^®^ Ultralow System V2 has been tailored for degraded and poorer quality DNA, resulting in higher library efficiency [45]. Illumina Nextera XT^®^ has a tagmentation step to fragment the DNA and attach adapters to the DNA fragments, and aDNA which is already fragmented could pose issues during adapter and index reactions when dealing with DNA of varying lengths [46]. In comparison NuGen Ovation^®^ Ultralow System V2 library preparation uses targeted sonication to fragment the DNA sample prior to processing resulting in a higher percentage of equally fragmented DNA strands. Nascimento et al. [45] systematically compared four library preparations including Illumina Nextera XT^®^ and NuGen Ovation^®^ Ultralow System V2 and found the latter outperformed in terms of library sample concentration, library fragment length (ca. 300–500 bp), good quality sequences and produced the best assemblies from the sequence data.

From this study, we conclude that the EznF DNA extraction method (based on DNA concentration, quality, PCR and sequencing performance), together with the NuGen Ovation^®^ Ultralow System V2 library kit gave the best results for use on preserved specimens of powdery mildew, as shown by the Vision alignment image (Fig 6). DNA concentration and selection of the appropriate library preparation kit were the major contributors to successful aDNA sequencing. Higher starting amounts of aDNA requires less amplification during library preparation and results in improved DNA library complexity, as amplification can preferentially select and amplify a portion of DNA present therefore losing genetic diversity within the library [47]. This is especially important when working with an epiphytic, biotrophic fungus such as powdery mildew which constitutes only a small proportion of the extracted DNA.

In summary, our key findings when working with plant pathogenic fungi from reference collections include: (1) selective sampling from the specimens to maximise the target fungus and minimise the contribution of other phylloplane microphylla and host DNA; (2) PCR amplification success was limited due to the fragmentation of fungarium DNA and whole genome NGS overcame this limitation; (3) DNA concentration was more important than DNA quality for whole genome NGS purposes; (4) a library preparation kit designed for degraded and fragmented DNA outperformed a standard use kit to generate fungarium sequence data.

## Methods

### Sampling

Five 25-year-old *Podosphaera leucotricha* reference collection specimens were sampled from the Victorian Plant Pathology Herbarium, Agriculture Victoria (Bundoora, Victoria, Australia). Specimens sampled were VPRI 18536 (collected 1992), VPRI 19785 (1994), VPRI 18575 (1992), VPRI 19947 (1994) and VPRI 18381 (1992). For standardisation of starting material, a 6 mm leaf punch was selected to cut sections of infected leaf material to be used in the DNA extraction protocol study. Powdery mildew conidia and mycelia were collected from leaves and stems by using a 6 mm leaf punch; specimen VPRI 19785 included chasmothecia.

### DNA extraction

Thirteen DNA extraction protocols were selected to cover the main DNA extraction methods such as chelating, silica binding and precipitation outlined in Table 7. Commercial DNA extraction kits manufacturer’s instructions and DNA extractions protocols from published sources were followed as per instructed, full methods outlined in S1 File.

**Table 7:** DNA extraction protocols tested on five VPRI apple powdery mildew *P*. *leucotricha* specimens in this study.

Method or kit name	Protocol Code	Reference or supplier (catalogue no.)	Extraction Method
Chelex^®^100	CheX	Hirata & Takamatsu 1996 [48]	Chelating
innuPREP Plant DNA	InuP	Telle and Thines 2008 [15] (Analytik-jena 845-KS-10600)	Silica binding
SDS	SDS	Edwards, Johnstone and Thompson 1991 [49], Pintye et al., 2012 [50]	Precipitation
E.Z.N.A.^®^SP Plant	EznS	Omega Bio-tek (D5511-00)	Silica binding
DNAzol^™^ with MinElute^®^ PCR Purification kit	DnaZ	Richards et al. 2019 [51]	Precipitation + Silica Binding
E.Z.N.A.^®^ Forensic DNA	EznF	Telle and Thines 2008 (D3591-00) [15]	Silica binding
Qiagen DNeasy^®^ Plant	DneP	Telle and Thines 2008 (69104) [15]	Silica binding
Isolate II Plant DNA Lysis buffer PA1 C	IspC	Bioline (BIO-52070)	Silica binding
Isolate II Plant DNA Lysis Buffer PA2 S	IspS	Bioline (BIO-52070)	Silica binding
Wizard^®^ Genomic DNA Purification	WizG	Promega (A1120)	Precipitation
E.Z.N.A.^®^ Plant	EznP	Telle and Thines 2008 [15] (Omega Bio-tek D3485-00)	Silica binding
CTAB	CTAB	Särkinen et al., 2012 [36]	Precipitation
Qiagen DNeasy^®^ Plant plus PTB	DneP+	Lister et al., 2008 [52]	Silica binding

DNA was processed from VPRI powdery mildew infected plant material placed in 2 mL Eppendorf tubes containing a metal bead and was homogenized on Tissuelyser II (Qiagen) for two rounds of 30 seconds at 30 Hz or until all plant material was broken down. For all protocols, a Ribonuclease A (RNase A) treatment was included to remove RNA during processing. DNA was eluted in sterile water or the elution buffer provided by the commercial kits. NanoDrop 2000^™^ was used to assess DNA quality using the 260/280 nm absorbency ratio (1.8–1.9). DNA concentrations were quantified using two methods: Invitrogen Qubit^™^ fluorometer and Agilent Tapestation^®^ electrophoresis.

### PCR amplification and sanger sequencing

**Fig 7:**
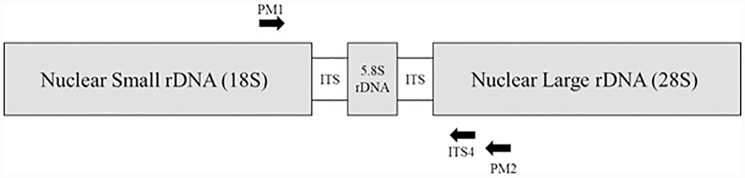
Primer map indicating nested PMITS1/PMITS2 and PMITS1/ITS4 amplified regions used in this study.

PCR amplification and Sanger sequencing were used to confirm the presence of *P*. *leucotricha* in DNA samples from the thirteen different extraction methods. A powdery mildew specific nested PCR was used spanning the ITS1, 5.8S and ITS2 (Fig 7) [27]. Primers used were PMITS1 (5'-TCG GAC TGG CCY AGG GAG A-3')/ PMITS2 (5'-TCA CTC GCC GTT ACT GAG GT-3'). The initial PMITS1 and PMITS2 PCR was performed in 20 μL reactions using the Dreamtaq 2x master mix, 500 nM forward and reverse primers, DSMO 5%, 5 μL dH_2_O and 2 μL DNA template. Thermal cycling conditions included an initial denaturing at 94°C for 10 minutes, followed by 35 cycles of denaturation at 94°C for one minute, annealing at 65°C for one minute and extension at 72°C for one minute; final extension at 72°C for 10 minutes. PCR products were confirmed on 2% agarose gel. DNA extracted from fresh *Podosphaera tridactyla* (GenBank accession MT309052) and *Podosphaera* xanthii (Genbank Accession MT309053) using the SDS method were used as positive controls for each PCR round as no fresh *Podosphaera leucotricha* was available at the time.

The nested PCR PMITS1 and ITS4 (5'-TCC TCC GCT TAT TGA TAT GC-3') reaction mix was set up as previously mentioned for ITS 1 and ITS 2 except the primer concentration was increased to 1000 nM and included 1 μL of the first round PCR product as the DNA template. Thermal cycling conditions for the nested PCR were the same as the first round except the annealing temperature was lowered to 60°C. PCR products were confirmed on 2% agarose gel.

Successful nested PCR products were sent to Macrogen (Seoul, Korea) for Sanger sequencing. All VPRI *Podosphaera leucotricha* ITS sequences generated in this study were accessioned to GenBank (Table 8).

**Table 8:** Successful nested ITS PCR *P*. *leucotricha* amplicons GenBank accession numbers generated in this study.

Protocol	18381	18536	18575	19785	19947
CheX	-	MT178355	-	-	-
InuP	MT178379	MT178380	MT178381	-	-
SDS	MT178390	MT178391	MT178392	-	-
EznS	MT178375	MT178376	MT178377	MT178378	-
DnaZ	-	-	-	MT178359	-
EznF	MT178368	MT178369	MT178370	MT178371	-
DneP	MT178360	MT178361	MT178362	MT178363	-
IspC	MT178382	MT178383	MT178384	MT178385	-
IspS	MT178386	MT178387	MT178388	MT178389	-
WizG	MT178393	MT178394	MT178395	MT178396	-
EznP	MT178372	MT178373	-	-	MT178374
CTAB	MT178356	MT178357	MT178358	-	-
DneP+	MT178364	MT178365	MT178366	-	MT178367

Extraction protocol abbreviations: Chelex^®^100 (CheX), innuPrep Plant DNA (InuP), sodium dodecyl sulphate (SDS), E.Z.N.A.^®^ SP Plant (EznS), DNAzol^™^ (DnaZ), E.Z.N.A.^®^ Forensic DNA (EznF), Qiagen DNeasy^®^ Plant (DneP), Isolate II Plant DNA Lysis buffer PA1 C (IspC), Isolate II Plant DNA Lysis buffer PA2 S (IspS), Wizard^®^ Genomic DNA Purification (WizG), E.Z.N.A.^®^ Plant (EznP), Cetyl trimethyl ammonium bromide (CTAB) and Qiagen DNeasy^®^ Plant plus PTB (DneP+).

### Phylogenetic analysis

ITS sequences from *P*. *leucotricha* VPRI 18536 from the 13 different extraction methods were aligned with sequences of *P*. *leucotricha* and *Podosphaera* species obtained from GenBank on the basis of the phylogeny published by Takamatsu, Hirata and Sato [53]. Extra *Podosphaera* species sequences were obtained using BLASTn. Initial alignment used the MUSCLE 3.8.425 package [54]. The alignment was visually refined and trimmed using Geneious 11.1.4 [55]. A maximum likelihood tree was generated from the aligned sequences using PhyML 3.3.20180621 [56] using the Hasegawa, Kishino and Yano 1985 evolutionary model with fixed proportion of invariable sites 0, number of substation rate 4 and estimated Gamma distribution parameter. Branch support was calculated with 1000 bootstrap replicates. *Sawadaea polyfida* var. *japonica* was chosen as the outgroup following the phylogeny published by Takamatsu, Hirata and Sato [49].

### Powdery mildew fungarium specimens next generation sequencing

VPRI specimen 18536 was used as a DNA representative from each of the 13 DNA extraction protocols, in a comparison study of two library preparation kits, Illumina Nextera XT^®^ (New England Biolabs) and NuGen Ovation^®^ Ultralow System V2 (NuGen).

Illumina Nextera XT^®^ double indexed and NuGen Ovation^®^ single indexed sequencing library preparations were completed for 13 VPRI 18536 DNA samples as per manufacturer’s instructions (S1 File). No DNA repair was performed on the fungarium DNA samples. The NuGen Ovation^®^ Ultralow System V2 libraries DNA samples were fragmented to 350 bp by sonication using Covaris S-Series Focused ultrasonicator. Fragmentation sonication settings are shown in S2 File. DNA library concentrations were quantified using Promega Quantus^™^ fluorometer and Agilent 2200 TapeStation^®^. The finalised Illumina Nextera XT^®^ and NuGen Ovation^®^ Ultralow System V2 libraries were paired-end sequenced on the Illumina^®^ HiSeq 3000 platform. Except for DneP+ Illumina Nextera XT^®^ and NuGen Ovation^®^ Ultralow System V2 libraries which were sequenced on Illumina^®^ MiSeq using the reagent V3 600 cycles kit due to a changeover in sequencing platforms in our facility and Illumina^®^ HiSeq 3000 is no longer available.

### Read processing and mapping

Reads were assigned to each sample based on their indices. Gydle programs were used for sequence read processing (https://www.gydle.com/). *P*. *leucotricha* VPRI sequences were filtered for quality using nuclear filter with a minimum score of 20, minimum length was set at 50 bp, and length total of 100. Mapping to reference sequence was performed by nuclear search with sequence length set at 100, sensitivity set at 25, kmer 13 and mismatches set at 0. Gym-build created files of mapped VPRI sequences reads to be visualised in Vision 2.6.24 (Gydle, Canada). References used for read mapping were a *P*. *leucotricha* series of reference scaffolds, which included *P*. *leucotricha* ITS (GenBank accession no. KX842350.1), *P*. *leucotricha* mitochondria and rRNA (generated using fresh *P*. *leucotricha* DNA, S3 File) and host DNA *Malus* chloroplast (GenBank Accession no. KU851961) and *Malus* mitochondria (GenBank Accession no. FR714868.1). Raw and QC read numbers were taken from total sequence reads before and after trimming. The mapped read numbers were displayed from the gym files by the Vision program (Figs 5 and 6). The total number of mapped sequence reads were converted to a percentage of the total QC read numbers.

## Supporting information

S1 File DNA extraction protocols and library preparation. (DOCX)

S2 File DNA extractions raw data. (DOCX)

S3 File Apple powdery mildew *podosphaera leucotricha* mitochondria genome. (DOCX)

S4 File Apple powdery mildew *podosphaera leucotricha* rRNA genome. (DOCX)
