# Supplementary material for: Rediscovering an old foe: Optimised molecular methods for DNA extraction and sequencing applications for fungarium specimens of powdery mildew (Erysiphales)
Source: PLoS One. 2020 May 13;15(5):e0232535. doi: 10.1371/journal.pone.0232535 (PMC7219758; doi:10.1371/journal.pone.0232535)
Supplement: S1 File — (DOCX) [file pone.0232535.s001.docx]

**Supplementary Information 1**

**DNA extraction protocols and Library Preparation**

**Chelex® 100 DNA extraction protocol**

Suspend 5% Chelex® 100 solution in dH^2^O 300 µL aliquots into 1.5 mL centrifuge tubes – can be stored in refrigerator 4°C for several weeks.

To be completed under dissection microscope. Place host leaf on filter paper, using a fine needle collect chasmothecia and dip into ethanol droplet on a microscope slide. Repeat for 20-30 chasmothecia. Allow ethanol to air dry, then place another clean/fresh microscope slide on top and using finger crush up chasmothecia. Then using the fine needle to collect crushed chasmothecia and place into Eppendorf tube with 5% Chelex® 100 solution. For conidia collection place host leaf on filter paper, cut a 0.5 cm strip of sticky tape (from the fresh end) using flame sterilised scissors and hold with sterilised forceps. Place cellotape sticky side down onto leaf surface in area of high conidia infection. Gently press down to collect conidia and peel off, repeat until sticky tape is sufficiently covered in conidia. Using forceps to open 1.5 mL centrifuge tube and place cellotape into Chelex® 100 5% solution.

Place Chelex® 100 tubes into a heat block at 56°C for 15 minutes. During this step set up boiling hot water bath (100°C), once ready place centrifuge tubes into bath and boil for 8 minutes. After first boiling step vortex thoroughly. Repeat boiling step. Vortex again and centrifuge at 15,000 rpm for 5-10 minutes. Label new centrifuge tubes and transfer 200 µL of the supernatant into new tubes, dispose of waste Chelex® 100 tubes.

**innuPREP Plant DNA extraction (ammonium chloride and cetrimonium bromide)**

Powdery mildew leaf and fungal tissue was homogenised using Qiagen Tissuelyser II for 30 seconds at 30htz. Powdery mildew DNA extractions were performed as per innuPREP Plant DNA isolation from plant material protocol (page 7 of innuPREP manual). No modifications were made to the protocol.

**SDS DNA extraction (sodium dodecyl sulphate)**

DNA extraction buffer: 200mM TRIS pH8, 250mM NaCl, 125mM EDTA and 0.5% SDS.

Place prepared powdery mildew infected leaf tissue in 1.5 mL centrifuge tubes and add 150 µL of DNA extraction buffer and crush samples with a sterile pellet pestle, ensure all fungal tissue has been lysed. Add again 150 µL of DNA extraction buffer to wash pellet pestle into the tube and vortex suspension for 30 seconds. Place centrifuge tubes into the -20°C freezers for 10 minutes, then place centrifuge tubes into a heat block at 70°C and incubate for 10 minutes. Vortex tubes for 5 minutes and return to heat block. Once heat incubation has concluded place centrifuge tubes into fridge 4°C for 5 minutes and then centrifuge suspension for 10 minutes at 13,000 rpm at 4°C. Transfer supernatant into a new 1.5 mL centrifuge tube containing 300 µL of ice-cold isopropanol and mix suspension by pipetting. Repeat previous centrifuge step then remove and discard supernatant carefully using a pipette not disturbing the DNA pellet at the bottom. Wash DNA pellet with 300 µL ice-cold Ethanol and mix carefully by pipetting several times. Final centrifuge step for 10 minutes set at 13,000 rpm and 4°C. Remove and discard ethanol supernatant carefully and let the samples air dry in tube rack until all traces of ethanol have evaporated. Resuspend DNA pellet in 50 µL dH^2^O and store at 4 °C or -20 °C for long term storage.

**CTAB DNA extraction for herbaria (cetyl trimethylammonium bromide)**

CTAB DNA extraction methods were obtained from Sarkinen et al 2012. All DNA extractions using this method were performed as published except an extra 70% ethanol wash step was included at step 10 as pellet was very dark.

**DNAzol^™^ with MinElute® PCR Purification kit DNA extraction for ancient DNA**

DNAzol solution is made up of Plant DNAzol^™^ and 2% PVP.

Prepared powdery mildew fungal tissue was placed in 1.5mL centrifuge tubes and lysed using Qiagen Tissuelyser II. Centrifuge tube for 5 minutes at 13,000 rpm to bring all fungal material to the together in the bottom of the tube. Add 150 µL Plant DNAzol extraction buffer to tube and incubate on a shaking rack for 15 minutes at medium speed. After initial incubation add 150 µL chloroform and incubate on shaking rack for 15 minutes at medium speed. Flash centrifuge tube to collect supernatant in bottom of tube. Transfer supernatant to new 1.5 mL centrifuge tube and centrifuge for 10 minutes at 13,000 rpm. Transfer upper aqueous layer into a fresh 1.5mL centrifuge tube and add 5x volume of buffer PB from MinElute® kit and process as per MinElute® PCR purification protocol (page 17 of MinElute® Handbook). Only modification to protocol 0.05% tween-20 was added to Buffer EB before use and then warmed to 30°C prior to elution step.

**Qiagen DNeasy® Plant**

Prepared powdery mildew fungal tissue was placed in 1.5mL centrifuge tubes and lysed using Qiagen Tissuelyser II for 30 seconds at 30htz until leaf tissue resembled powder. DNA was extracted following the DNeasy® Plant mini kit instructions. Modifications were at step 8 an extended 65°C incubation for 30 minutes, for increased cell lysis and RNase activation and final DNA extraction was eluted to 100 µL.

**Isolate II Plant DNA (PA1 & PA2)**

Prepared powdery mildew fungal tissue was placed in 1.5mL centrifuge tubes and lysed in Qiagen Tissuelyser II for intervals for 30 seconds at 30htz. Two DNA extractions were performed using the Isolate II Plant DNA kit, PA1 (CTAB) and PA2 (SDS). DNA extractions were completed using the standard protocol for purifying plant genomic DNA, except for a repeated DNA elution (step 7) to a total volume of 100 µL.

**Wizard^®^ Genomic DNA Purification**

Prepared powdery mildew fungal tissue was placed in 1.5mL centrifuge tubes and lysed in Qiagen Tissuelyser II for intervals for 30 seconds at 30htz. Add 600 µL of Nuclei Lysis Solution to centrifuge tube and vortex to mix through. Then incubate for 30 minutes at 65°C. The RNase was added to each sample. Samples were cooled to room temperature before adding 200 µL of protein precipitation solution and vortex at high speed for 20 seconds. The samples were centrifuged for 3 minutes at max speed to precipitate proteins. The supernatant was transferred to a new centrifuge tube containing 600 µL of room temperature isopropanol. This was mixed gentle by inversion for 1 hour at low speed. Centrifuged samples at max speed for 1 minute to pellet DNA and remove the supernatant. 600 µL of room temperature 70% ethanol was added and washed the DNA pellet by inversion. Centrifuge at max speed for 1 minute and removed ethanol by pipette, then air dry the DNA pellet and remove all traces of ethanol in the centrifuge tube by inverting on paper towel for 15 minutes. Elute DNA in 100 µL of DNA Rehydration solution and incubated at 65°C for 1 hour.

**Omega Bio-Tek E.Z.N.A.^®^ Plant DNA Kit**

The E.Z.N.A.^®^ Plant DNA kit for dried plant samples protocol was used for the extractions. . Prepared powdery mildew fungal tissue was placed in 1.5mL centrifuge tubes and lysed using Qiagen Tissuelyser II for 30 seconds at 30htz. Rapid protocol was followed as published except for repeated final elution step for a total of 100 µL.

**Omega Bio-Tek E.Z.N.A SP Plant DNA Kit**

The E.Z.N.A SP plant DNA mini kit for dried samples was used for the DNA extractions. . Prepared powdery mildew fungal tissue was placed in 1.5mL centrifuge tubes and lysed using Qiagen Tissuelyser II for 30 seconds at 30htz. Standard protocol was followed as published except for repeated final elution step for a total of 100 µL.

**Omega Bio-Tek E.Z.N.A.^®^ Forensic DNA Kit**

The E.Z.N.A.^®^ Forensic DNA standard protocol was followed for the herbarium DNA extractions. Prepared powdery mildew fungal tissue was placed in 1.5mL centrifuge tubes and lysed using Qiagen Tissuelyser II for 30 seconds at 30htz. Standard protocol was followed as published except for an extended initial incubation step was increased to one hour and a repeated final elution step for a total of 100 µL.

**Amended Qiagen DNeasy Plant with PBT**

Prepared powdery mildew fungal tissue was placed in 1.5mL centrifuge tubes and lysed using Qiagen Tissuelyser II for 30 seconds at 30htz until leaf tissue resembled powder. Prior to starting protocol N-phenacylthiazolium (PTB) was added to a final concentration of 5mM was added to Lysis Buffer (AP1) of the DNeasy® Plant mini kit. DNA was extracted following the DNeasy® Plant mini kit instructions. Modifications were at step 8 an extended 65°C incubation for 30 minutes, for increased cell lysis and RNase activation and final DNA extraction was eluted to 100 µL.

Library preparation

Illumina Nextera XT®

Illumina Nextera XT® libraries were generated following the published reference guide, with no modifications to workflow.

NuGen Ovation®

Following Covaris S-Series Focused ultrasonicator user manual for start-up procedures to prepare equipment for sonication. DNA shearing settings for 300 bp are as follows: Intensity 4, Duty Cycle 10%, Cycles per burst 2 and treatment time 40 seconds.

NuGen Ovation® ultralow system V2 libraries were generated following the published used guide with no modifications to the workflow.
