# Supplementary material for: Rediscovering an old foe: Optimised molecular methods for DNA extraction and sequencing applications for fungarium specimens of powdery mildew (Erysiphales)
Source: PLoS One. 2020 May 13;15(5):e0232535. doi: 10.1371/journal.pone.0232535 (PMC7219758; doi:10.1371/journal.pone.0232535)
Supplement: S2 File — (DOCX) [file pone.0232535.s002.docx]

**Supplementary Information 2**

**DNA extractions raw data for Table 2 – Nanodrop concentrations (ng/µL) and quality (260/280 nm), Qubit concentrations (ng/µL) with total DNA (ng) and Agilent TapeStation DNA concentrations (ng/µL) with total DNA and the DIN score (DNA integrity number).**

| **Samples** | **Nano Conc ng/uL** | **260/280 nm** | **Qubit ng/uL** | **Total DNA** | **Tapestation ng/uL** | **Total DNA** | **DIN** |
| --- | --- | --- | --- | --- | --- | --- | --- |
| **CheX 18536** | 157.6 | 0.55 | 0.522 | 104.4 | 2.15 | 430 | 0 |
| **CheX 19785** | 9 | 1.32 | 0 | 0 | 2.11 | 422 | 0 |
| **CheX 18381** | 132.7 | 1.41 | 1.25 | 250 | 2.33 | 466 | 0 |
| **CheX 19947** | 14 | 1.36 | 0.141 | 28.2 | 2.22 | 444 | 0 |
| **CheX 18575** | 83.4 | 1.7 | 0.588 | 117.6 | 2.42 | 484 | 0 |
| ***Median*** |  |  |  | *104.4* |  | *444* |  |
| ***Range*** |  |  |  | *104.4 (0-117.6)* |  | *(422-484)* |  |
| **InuP 18536** | 5.6 | 1.29 | 0.427 | 85.4 | 4.18 | 836 | 1.5 |
| **InuP 19785** | 4.9 | 0.6 | 0 | 0 | 3.67 | 734 | 2.8 |
| **InuP 18381** | 5.1 | 1.75 | 0.313 | 62.6 | 3.59 | 718 | 1 |
| **InuP 19947** | 3.9 | 0.57 | 0.124 | 24.8 | 3.87 | 774 | 1 |
| **InuP 18575** | 5.2 | 1.94 | 0.302 | 60.4 | 2.97 | 594 | 0 |
| ***Median*** |  |  |  | *60.4* |  | *734* |  |
| ***Range*** |  |  |  | *(0-85.4)* |  | *(594-836)* |  |
| **SDS 18536** | 7.2 | 6.99 | 0.107 | 5.35 | 2.73 | 136.5 | 0 |
| **SDS 19785** | 8.7 | 2.7 | 0.159 | 7.95 | 2.45 | 122.5 | 0 |
| **SDS 18381** | 5.1 | 5.37 | 0.055 | 2.75 | 2.43 | 121.5 | 0 |
| **SDS 19947** | 7.9 | 2.51 | 0.056 | 2.8 | 2.07 | 103.5 | 0 |
| **SDS 18575** | 84.7 | 2.21 | 3.79 | 189.5 | 3.28 | 164 | 1 |
| ***Median*** |  |  |  | *5.35* |  | *122.5* |  |
| ***Range*** |  |  |  | *(2.75-189.5)* |  | *(103.5-164)* |  |
| **EznS 18536** | 241.6 | 2.18 | 24.7 | 2470 | 10.7 | 1070 | 2.9 |
| **EznS 19785** | 66.2 | 2.17 | 3.18 | 318 | 5.2 | 520 | 1.5 |
| **EznS 18381** | 117.2 | 2.16 | 8.32 | 832 | 7.28 | 728 | 1.6 |
| **EznS 19947** | 113.5 | 2.15 | 13.7 | 1370 | 12.4 | 1240 | 3.2 |
| **EznS 18575** | 256.6 | 2.2 | 26.9 | 2690 | 10.6 | 1060 | 2.1 |
| ***Median*** |  |  |  | *1370* |  | *1060* |  |
| ***Range*** |  |  |  | *(318-2690)* |  | *(520-1240)* |  |
| **DnaZ 18536** | 39.2 | 2.11 | 0.998 | 49.9 | 2.62 | 131 | 0 |
| **DnaZ 19785** | 29.2 | 1.95 | 1.13 | 56.5 | 2.51 | 125.5 | 0 |
| **DnaZ 18381** | 47.6 | 2.06 | 0.94 | 47 | 2.27 | 113.5 | 0 |
| **DnaZ 19947** | 32.3 | 1.93 | 0.545 | 27.25 | 2.64 | 132 | 0 |
| **DnaZ 18575** | 43.9 | 2.29 | 1.06 | 53 | 3.02 | 151 | 1 |
| ***Median*** |  |  |  | *49.9* |  | *131* |  |
| ***Range*** |  |  |  | *(27.25-56.5)* |  | *(113.5-151)* |  |
| **Samples** | **Nano Conc ng/uL** | **260/280**  **nm** | **Qubit ng/uL** | **Total DNA** | **Tapestation ng/uL** | **Total DNA** | **DIN** |
| **EznF 18536** | 71.2 | 2.07 | 6.17 | 617 | 6.37 | 637 | 1.8 |
| **EznF 19785** | 23.1 | 1.82 | 1.33 | 133 | 2.91 | 291 | 0 |
| **EznF 18381** | 36.1 | 1.97 | 2.6 | 260 | 3.35 | 335 | 1 |
| **EznF 19947** | 40 | 1.81 | 3.34 | 334 | 3.64 | 364 | 1.7 |
| **EznF 18575** | 298 | 2.14 | 39.3 | 3930 | 9.41 | 941 | 1.3 |
| ***Median*** |  |  |  | *334* |  | *364* |  |
| ***Range*** |  |  |  | *334 (133-3930)* |  | *(291-941)* |  |
| **DneP 18536** | 12.3 | 1.86 | 0.466 | 46.6 | 1.14 | 114 | 0 |
| **DneP 19785** | 4.8 | 1.96 | 0.384 | 38.4 | 2.12 | 212 | 0 |
| **DneP 18381** | 9.9 | 2.05 | 0.439 | 43.9 | 2.53 | 253 | 0 |
| **DneP 19947** | 16.2 | 2.26 | 1.25 | 125 | 2.49 | 249 | 0 |
| **DneP 18575** | 40 | 2.23 | 3.66 | 366 | 2.66 | 266 | 0 |
| ***Median*** |  |  |  | *46.6* |  | *249* |  |
| ***Range*** |  |  |  | *(43.9-366)* |  | *(114-266)* |  |
| **IspC 18536** | 39.3 | 1.92 | 1.93 | 193 | 3.19 | 319 | 0 |
| **IspC 19785** | 6.4 | 1.98 | 0.246 | 24.6 | 1.38 | 138 | 0 |
| **IspC 18381** | 7.3 | 1.87 | 0.341 | 34.1 | 2.98 | 298 | 0 |
| **IspC 19947** | 22.6 | 1.43 | 0.943 | 94.3 | 2.39 | 239 | 0 |
| **IspC 18575** | 21.1 | 1.95 | 1.42 | 142 | 0 | 0 | 0 |
| ***Median*** |  |  |  | *94.3* |  | *239* |  |
| ***Range*** |  |  |  | *(24.6-193)* |  | *(0-319)* |  |
| **IspS 18536** | 77.3 | 1.83 | 2.12 | 212 | 3.96 | 396 | 0 |
| **IspS 19785** | 6 | 1.89 | 0.21 | 21 | 2.58 | 258 | 0 |
| **IspS 18381** | 18.8 | 1.86 | 0.6 | 60 | 2.31 | 231 | 0 |
| **IspS 19947** | 18.1 | 1.83 | 1.38 | 138 | 4.91 | 491 | 0 |
| **IspS 18575** | 34.6 | 1.83 | 0.968 | 96.8 | 2.93 | 293 | 0 |
| ***Median*** |  |  |  | *96.8* |  | *293* |  |
| ***Range*** |  |  |  | *(21-212)* |  | *(231-491)* |  |
| **WizG 18536** | 336 | 2.07 | 34.4 | 3440 | 9.71 | 971 | 1 |
| **WizG 19785** | 77.6 | 2.04 | 3.14 | 314 | 2.82 | 282 | 0 |
| **WizG 18381** | 67.2 | 2.18 | 3.57 | 357 | 3.41 | 341 | 1.2 |
| **WizG 19947** | 86.9 | 1.92 | 7.83 | 783 | 3.8 | 380 | 1.8 |
| **WizG 18575** | 207.1 | 2.13 | 17.6 | 1760 | 5.29 | 529 | 1.2 |
| ***Median*** |  |  |  | *783* |  | *380* |  |
| ***Range*** |  |  |  | *(314-1760)* |  | *(282-971)* |  |
| **EznP 18536** | 45.9 | 1.98 | 11.3 | 1130 | 11.4 | 1140 | 2.3 |
| **EznP 19785** | 21.1 | 2.32 | 2.82 | 282 | 3.51 | 351 | 1 |
| **EznP 18381** | 22.4 | 1.83 | 3.17 | 317 | 4.34 | 434 | 1.3 |
| **EznP 19947** | 47.4 | 1.71 | 10.9 | 1090 | 8.89 | 889 | 1.9 |
| **EznP 18575** | 28.6 | 1.87 | 12.7 | 1270 | 16.6 | 1660 | 4 |
| ***Median*** |  |  |  | *1090* |  | *889* |  |
| ***Range*** |  |  |  | *(282-1270)* |  | *(351-1140)* |  |
| **Samples** | **Nano Conc ng/uL** | **260/280**  **nm** | **Qubit ng/uL** | **Total DNA** | **Tapestation ng/uL** | **Total DNA** | **DIN** |
| **CTAB 19785** | 4.6 | 1.4 | 0.07 | 5.25 | 3.09 | 231.75 | 1.8 |
| **CTAB 18381** | 6.6 | 1.89 | 0.363 | 27.225 | 3.17 | 237.75 | 1.5 |
| **CTAB 19947** | 7.9 | 1.88 | 0 | 0 | 2.89 | 216.75 | 0 |
| **CTAB 18575** | 9.5 | 1.79 | 0.55 | 41.25 | 2.19 | 164.25 | 0 |
| ***Median*** |  |  |  | *16.2* |  | *224.25* |  |
| ***Range*** |  |  |  | *(0-41.25)* |  | *(164.25-237.75)* |  |
| **DneP+ 18536** | 6.6 | 1.67 | 1.84 | 184 | 4.44 | 444 | 2.1 |
| **DneP+ 19785** | 2.3 | 1.15 | 0.083 | 8.3 | 2.1 | 210 | 0 |
| **DneP+ 18381** | 2.3 | 1.41 | 0.144 | 14.4 | 2.62 | 262 | 0 |
| **DneP+ 19947** | 2.2 | 1.14 | 0.363 | 36.3 | 2.04 | 204 | 0 |
| **DneP+ 18575** | 3.1 | 1.33 | 0.525 | 52.5 | 2.42 | 242 | 0 |
| ***Median*** |  |  |  | *36.3* |  | *242* |  |
| ***Range*** |  |  |  | *(8.3-184)* |  | *(204-444)* |  |
