# Supplementary material for: Rediscovering an old foe: Optimised molecular methods for DNA extraction and sequencing applications for fungarium specimens of powdery mildew (Erysiphales)
Source: PLoS One. 2020 May 13;15(5):e0232535. doi: 10.1371/journal.pone.0232535 (PMC7219758; doi:10.1371/journal.pone.0232535)
Supplement: S3 File — (DOCX) [file pone.0232535.s003.docx]

Supplementary Information 3

Apple powdery mildew Podosphaera leucotricha mitochondria genomes 1 and 2

>lcl|tig00000002 len=173096 reads=24522 covStat=1.00 gappedBases=no class=contig suggestRepeat=no suggestCircular=no

Apple powdery mildew Mitochondria genome 1

TTTAACCTTATTTCAATAATTCTTATTATTGTTCAGACTATATCATGTATACAATAATTTAGCTATATGTATACCCAAGT

TTTTAAATAAATAAAAAAGCCACCCTACGGGATCCTACGGATCCTCCGTATCCTCCCGGATCCATACTTTGAAGGGCCCT

ACCCTTTTTAAAGAAAAGTAGTTCCTGAGGGACCAGGAACTGGTCCCGAGGGGAACCAGTCCCCTAGTACTTTTTAATAT

TCTATTTAGAGTCGTTGAACCTGCCTATTAAATATCATCTGTAATATTAAAAAGGATAGGCTGCATATTGTCTTGAGCTT

AATTCACAATTTACGCTAAAAGTGTATAGCTTAAGTTTTAGCGCTCAATGCAAATGGTGGTAAAACAAGGTGGTTTTCAT

GCAATTATCTTGGTTTATTTGCCTAATTACGTGCATAAATTAAATAGCTGGGGTGGGTATTGATTAATTTATGCATCTCT

CACTAGAGCAAAGGCCTATATAATATACTTTTGGGGTTTTCCTTAGCTTTCTGCAAATGAAAACACCGCCCTCGAGGGAG

CAATAATTATGTATTCTACGGCTATTCATACCTGATTTTAAATAATTTATCCTATTAATACCGGAGGGCTGCTTGCTTGG

GCTGGCTTGCCCCTGGCTTCCCCTGGCTTGCGCCTTGCTTGCTTGGCCCCTGGCTTGCTTGGCTTGCCCCTGCTTGCCCC

TGCTTGCCTTGCTTGCCCCTGCTTGCCTTGCTTGTCGAGAGTTAAATGACCTTTAACTTCTATAATCTTAGCTACCTTAA

CAAAATCTAAGTAATCCCTAGACTTTATTCCTTGTCCCTCCTAGGGAGGAGCCTAAGGAACAACTTTAGAAAAAGGAATT

ACCTTATCTTTTATATCACTAAATTTACTTACAGAAAAATTAACCAGATCTGGTCTGGTTGAGGCTCTCTCAATCCTACC

GCAATCTAGGTAGTCAATGAATTTAGTTAATAATGCCTCATCTCTTCTAATCACCTCCCAGGAGGGAATGTTGAATATAG

AGAAACCCATAGTTTTCTCACGTTAAAGCCTGTTGAATATTTTTATTCTTTAGAGATTTAACATAAAAAATCCCTCTCCA

TCTACAAAACCACCTAATCAATTCGGATCCTGTATCCCCTTGTCCACTTACTAGTGGACGAGGTAGAGGTAGGACGGTAG

GAAGTGAATTTTTAATACGTAAGATAATCCTTTATTCATGGAACCCTTAATACTTATAATCTGGCGGATTCCTTTAAAAC

TAGATCCGTGCTTGCCCCCATCTAATAATAAGTATACAGGTCTAAATAAAAGGTCCCTCCATCTCCCCTCTTTTGAGTTA

TTAAAGGATATTTATAAAAATGTGGTATAATTACATTAGCTATAGCACGTGCAGACTGAACGCTAAAATATGCCTGATTT

CTGTCATCACGTTCGCCAACTATACCTATACCGAAAAGGAATGTATTTTCTCAATAACAGTAAGTCCCTGCCTATGTAGC

ATTATTTGAAAATCTGGTGTCACATTTCACCCGGAGCGAGTGGTACTGCTTTTAGATACCTTCAGACTAAAGCTAGACTC

CGCATCACAAAACCCAGTAACTCAGTATGGAGAAAGCTTCGGTTCTTTTACATCCACTATGGTTGTGAAAAGTCTTTTAT

GCGAAGCGTAACCCTCTTCCTTTCGGTCCCCTGCTGTATAAACAATACTGGGGAAGGAAAAGCTCTTTTTGATAACAGAC

TTATGGACAGGTTCGTTTAGCTATAGTGACCGAATTATAATAATTACTTAAGGATCACTTGATAAAACCAGGCACAACCC

CCAGGGAAAAAAGTATAAAATTGTAGATAAGCCTGTGAATACAACTGCTAAAAGTACAGTAGCAACTAACCCATATAAAG

TTCCTCTTCTATTTCCTTGAATCAAAGAATGGTGCGCTCAAGTCACAGTTATCCCTGACGATAAAAGTAATACTGTGTTA

AGTAAAGGTAATTCAAAGGGATTTATAGGTAGGGTCAGTGTTGTGACTCAATGCTGCCCTCAGAACCGTACGTGATAGTT

TCCCATCATACGGCTCGCCCCCAGTCCTGCTCCAAAGTCTCCTGTATGCGGTGGAGCTTACTCATTATTTAGAATTTGGG

TTATTTCCCATGTTTGTTATAGCTATGGATACGTTTCATATCCAATCAGTTCAATTCCCCTTGTGGAACAATATATGATG

ATAAGAGCATAAAGGTACTTGTTTTCGCATTATTGCTCCATCAAATTGTGCTCTTGTAATCTTCTCACCTTTTCTATATT

TGGCTCTGATGTTTTAACTGATCGTATGTGGTGCATTTCAATAAATTGATGCGTACCACAGATCGTACAGCTTTCATTGA

AGGATGATTTCGTATCTTTACCAGCCCATGTTTGACTGATGATTTTGTCGATATCTCTGTCATTTTTCTTCCCATAGTCG

TGCATCACTTTTAAATTGTCGACTTTGAAGTATTTCGTGTCAGTTTCTGGGCAGGTTAGATCTGGTCCATACACTTTGAA

TACTTTGCTCATGGTTCCGAGTTTGTATTTCTTAGCCAAGGTTAAGGCACATGATGCCTTTAATATTCAGTTTATCGAAT

ACAAACTAGCTCTGTTTGCAGCAAATCTGTAAAAGTTTATTAGACCCTGATTTTGTCATTGTAGTGAGTCACGATTTCGT

GATGTGATAGATTCATCATGGGAGTGTAGCCTGTTGGTAAGTATTTTCCTTTATGATTTTGTCTGATAAATTTAGCTGCC

TTAAGTTTCTTTAGTATAGTCTCAACTGGAGCTGAGACTAGTGTTCTGGCAATTCCGATAGCTCTACGGGTCCGTCCCTG

TTTAATTAGAAACGAGTTGTTGACCCTAAGGTTTCGGTTTCGGCTCCCAAGAAGTTCCATTTATCTGTCATACTATTTAT

GGTAGTCTTTTCATCATTCAGGTCAAGACCACACTTACCTTTTAATACTTCCCTTATTTTATTTTTATATATTTCGCATT

TTTCAGGTCTCCCACCAGTAATACAACAAAATCATCAGCATATCTGATATATGTCAATCTCCTTAAGTTCGGGTCAAAGA

GGTCTGATCTTCTTAGGCTTCTCATTCTAGTAAGGATCTTAGTTCTCTCGGTTGGTTCAGCAGTCTTGGCTTTTTGGCTT

GTAGAGCAGCATACAGAGGGTTTTTCCTTCTCTTTGTTCCTCTATCAAATCTCTTTTCTCGTCCTCTAGGAACGTGTCTA

GTTGATATAAGACTATATTAGCAAGAAGTGGACTCAATACACCTCCTTGTGGAGTTCCCTCCTTTGGTTGGTGTAGTATT

CCATCATCCCTTTATAACCTGCGCTTATAAACTTTTGGATTAACTCTAGCAATCTAGGGTCACCGATTTCCTTGCGGATG

TATTTCATAATGATTGTGTGTGGTATATTGTCAAAACATTTCGATATATCCCCTTGTATCACTCAGTTATATTTCGATCC

TTTTAAGTATATATCCAATAATGCTGTGTGGACCGATCTTCCTCTTCTAAACCCATGCGAGGAGGCTGAGAATTTAGGTT

CAAATATAGCTTCCAGGATACCATGTATCCCTTTTGTACTATCTTCTCCCTAGGAGTTCCAATACCTAGTGGTCTTAATT

TACCATTAGCCTTGGGTATTTCAATTCTACGATTTGGGGAGAATCTAAACTTTCCTGATTTAAGTTCCTTTGCAGTCTTC

TCAAATCATTCCCAGTCTATTCCATCCAGGGTCTCCTTGGATAACCCCTTCGTCATATTTCCTGGTTTGCTCATTATCTC

ATTGTAACACTTTTCTAAGTAAAATGGATTTGCTAAAATTTCTAATAATTTGTAATGTTTTCCTGCAACATTTGATCTTT

GTTCTAAAAGTTTTGTGACTGTAGTAGTAACGTCATCTCTACCAGATACCCATAAGGGTTTGTCTGACTTTGATGACACA

ATATTGTTATTATCTATAGCGTTTTCTTTTAATGAAGCCTGGCTGTCTCCCTTTGTGTTTCAAATAGTCGCACTAATATG

GAGACTCCGTCCCGCATTAAGTACAAATCGTACTCATAGGCGGTTAGATCCCGAGGTTGTCTACTAGTGATGTTTAACGC

CTGTACTGATTTAGCAGCTTGCACATTCCTTTTCAGGTCTCCTGTAGGGAGAATCAACTAACTTTTGGCAACATCCAGAT

TTAGAAGCACAAATGGATGCAATGTTAGTATAACACCGTGATATTCTGCTTTTGATGTTACCCCTGGGTTGATGGTGAAT

ATGTGTATCAAATTTTTATCATCAGCATGTCAGTTCCCACTAATTTTGTTATCTTTTCGGGGCAGCCCAAATCATCCAAA

ACTTTTATATTCAGCTATCCGTGATCAGAGTCTTGTTAGGTTTTCTGTCCGTGAATATCCGTGGTATGTATCTATAAATG

AGGCCAAATATACATAAATTGTGAGGCTACACAATAAAACACTAGCGACCTCTCTCGGCGCACCTTCTATACCCATTGGT

GGTCAGCTAGCTCCTAACTCTACGGTTGGTGATAAAGCACTCGTTATTAAAGTAACCCGCAGTTACTTCACTTATAAAGA

AGCACAATATAAGTTTTTAATTTTATTCACTAGTCCCCGGGCGCGAACATGTTCCTCTCCCCTTTTCTTTTAATTAAAAG

AAAGGGGGACAATCCCGGATCGCAGAGGGAACTATTATGTTTATGGGCTTGGTTAAATCCCATCCATGGTTGCCTATACT

CTCTTTTTATTCATTCCTCCTTTTAAGATTTTAATTTTATCTAGCCCTTCTCTGGTTAAATGATCTTTATTTTTAATAAG

CAGAGCCGCTTTCTTGAAATCCTCAAAATCCGGAGCTTTTTACCCTGTAGTTTAAATTTTTCAAATATGGGAATTATTTT

ATCGTTAACATCTGTAAATTTTTCAACTATAAATTCACCATAACCAGATTTAGATATATATCTACCACAATTTAAAGCAG

TAATTAAACTTCTTAGTAAATATTCATCTCTAGTATGTTGAGTTAAAATAAATCTCAATCATACGCTTTCACCTAATATA

GAACCGCTTTTTAATGCCACAAAGAAACATCCTTCTGCATCGGTAAAACCTGCTAACCAATGTAAATTTGGGATTTTTCT

ATCTTTAATTTCGGGTCTTAAAACAGGTTTTAAAGGTAAAAGATTTAAATTAGGGATTTATCTCCCTTATTTAGCATAGC

TCTCTTGAACATAATTTTATTTACACCCTCTTGAGTTAAATGTTTTCCTTCTTCTATTAATTTTACTACTTGTTTAAAAG

TAGAAAACCAGATTTCTTATTAGTTATTAAAGGGTATTTATCAAAATGGTTAACTATAACTTTTAAGTGTTCAATAGCAT

ACACTCTAAATTGTACAGATTCTCCAAGCAATTTAGAAATTTGGCCTACCCAAAGAACGATTGAATTTGTTCTAATAAGG

CAAGATCCTTTATATGTAGACCGATTTGAAAACTAGCTTTTACTCTGTAACCTGTCTTGCTTTTAGAATCTCGACTAATA

CCCACAAAAACATCCTTCCCCATCCGTTAAACCTGTAACATAATAAGGATTTAACTTATTACAAGATAAAGAACTAAGTT

CGCTTGACGCTTTATCTTGTGCAAAGCAAGTCATATTATTTTCTTTGCCTTTGACGTAATCGGCAAAGAAATGACTACGT

AAAAGCCCCGCATGAAAGTAAAGCTTTGAAATAAATATATTGGGAGTTTTAAATAAGCCAGGATAAGCCTTATAGATTGA

ACCATTGTATTAACATAATTCATATGATAAATAAAACCAGGTTTCCCTGACACTTAGAGTACACCTTACGGTATTTTTTA

ATACCGAGAAACCGTCTACTCGTTGCTCTTTTACAGATATGTAGCTATCTGGTTTAGATCCGCGATTGCCTATTTCTTGA

TTTACACCAAGAATCTCTAATGATGTTACTATACCCTCTGCTATTAACGAGGCCACTTTAAAAGTTTCCTTAAAAGCTTA

GTTATTCCCTCGCACCATCATTTCGTTCGATGGTGCGGGCCGGCCCAAAGCCGAGAGCTTTAGGGTGTCCCCGAGTATGG

CTTCTTTCCACAATTTGCTTTGTTTATGGAAAAATGCTCAGAATATAGCTAAAAAATAAAGCTTCTGAAACAATAAATAA

AGCAACACCCATATTCAATCCCCTTTGAACTGCCAAAGTATGATTTCCTAAATAGGTACCTTCTGATATTACGTGTGGAG

TAAAGAGTTCGGCTTTCGCCTACTAAAGTCCTCCCCGAACCGGACAAGATGGTCACCCATCATCTCGGCTCTCCAACCCT

ACATATATTAACTGTGATTTAAGGAACTGATTTTCCTTTGATTTTAGTAGACTATTTAGTCCATTTTGTGTGCCATTTTA

TGATGTAGACGACATAGGGGACAGTCCGGTTAAGGCTCCTCCTCCTCATCATTTCGGTATGGACAGTTGTACCTTTAAGG

TATTTTAGCCCCTTTATGTGATCTCCACCTTTCGTTCTTGACCGCAGATCACACATCTGGCATCCCCGTTAGCCTGACAG

TTCTACTCATGAGTCAGTAAAATGCTCTTAGTGGGTTAGCATCCGTTCTTTTGGGGTTATCCTAATGATTTCCTTGAGTG

TTGGATTGAAGGACTTGCAAAGGGAGGATATCTGGGCTAGGATCTCGTCATATTTTGAATAGAATAGGGATACCCCCAAC

TCTTTTACCTCGCTATTCTCCCTTCTCTTAACTAGTCCCTCCCCGGAGACTTATCCCTCCCAATCCCCTATCAATCCGAT

GATCATCGATCCGGATGGGATTGGGAAGGAAGGACTCAGAGAGATTTGAGATACTCGTAGATTTTCTCCTCAGTCTTACC

AATTACAGCTTTGGCGTAAAAGGCCCGTCTGATTTAATGGGTCTACTTAGATCGTAACCCCTTGGCAAAAATCTTGGCAA

CACTTTTCATCCTGTATTTGGCGGCAAACATCTTTGCAATAGAGAATTTTATGATATAATTTCATCTAATAATCATCTGT

CTATAGTTATTGGCCAGCTTGTAGTAGTTAGCCAACCCTCTCAGAATGTATCCTACTTGGATTATCGTACCGTACTGCGT

ATTGTTCAGATATTTAAAGTTCGCTATAGGATGTCCATTACGTTGACAAAACCCTTATCGAACAGACGTTTCTTTACTTT

CTCCGAATCTGGTTTGAGGAAGATACTCCCCGTGGTCATTCTACGGACTTTTCTAATTTTATCCCCGTATCTTCGGTAGT

ATATATTTACTTTAAACGCTGCCTTTCCTATCTGATAGCCTAGAAAGCGAATCGGATCACTCTTAGGATTAGTAATCAGA

GTTTTTCTTCACTCAACGTCAATTTAAACCTTTCGAGGAACTGAGCACATTTCTGCTTAATCTCCACTGCTTCCTTCTTC

GGCCCTATGATTGTAACTAAAAATCGTCCGCATACCTAACATAATGCATACGACGATAGTTAGGGTCCATTGAGTCCGAT

TGTCCTATTTGTCGAGCTGCTGCAGCTTTAACCCCGTATTTATGATATACTCTCGAGTACTCCGGGTTAGCTGCTCTTCT

ATGCTTCCCTCGGTTATACTCTTGGATTGACTTCTCCATGTAGAGGTCAAATTCGTGCAAGAGAATGTTGGATAGCAGAG

GACTTATAATCGCACCCTGTGATCCTATACTACTGACCAGCTCGAGTTTGCCTTCCTCCCGTATAATCGTCTTAAGTAGG

CTATGAACTAGTCCGAGGAATCTGGGGTCCTGAATCTTACGCCCCAGAAGTTCCATAAGCACTCTATGGTCGAGATTATC

CAAAAGCAGCTGCAGCCTTTTATGTAACCTTCAATAGCCCAGGTAAATCCGGGACTACCTGCTCTGATATGCCTCAAAGC

GGTGTGACAGCCTCTACCTGGTCGAAAGCCATGAGAGTGAGTAGAAAAGATGGGTTCGTATATGATTTGTAATATGGTCC

GCACCACCTCCTCTTGTACAGTTCTATCATCGAACGGTGGAATACCTCACGGTCTCATTTTTCCATTTGCCTTTGGGATA

TATACGCGTTTGGTCACACCTACTTTATATTCCCCGAGATTACTTTATCTCTTAATGCTTTTAATCTGGCGAGGGTTGTT

CCGTCGATTGTGATCTTATCGAATGAAGGTGTGTTGGAACCGGCTGAGCGGGACAGTTTTATATACGCAACCTTCCACAG

ATCGAGTTCTTTCATTAGACTAAATAATCTCTCGTTTACAAAGTGAGGATCGTTCTTATTCCCGTTTCACAGTGTCTCCA

TTCTACGTGCAACCTTGGAGTCCTTCCAGGACGGCGCAGTAGCTGGAGACTTAGTAGAGTATGACCGGGATATTATCCCG

GTATTTAGCCCCTTCCATCTTACCTGATGGTACTACGGAGCCTCCGCCCCACAGGTGCCGCTGTATTAGTAGTCAGCGCG

AAAATTTCGCCGCATTCCCATCGGGATGAGAATACTCCTGTGGTTCACCGGTATCGCACAATGCAGCTGACCTGTTCTTG

GTCTGGTTAATCTAAGTAATCCTTCCGATCTTTCCGAACTTACTGTCCGTGCAGTAAGCTTAGTTTCTAGAGGGTAGCCG

ACGAGACTGACACCCCATACTTACGTGGTAGTGATAATCGAGCATAACCCACTGGTTTTGATGGCCCATACCGATCTAGG

ATTCAAGACTGACCCTTTTACTTTGATTAACGGCTTTCACTCGAGAGGGGTCCTTGGAACGCTCAACTCCAGTCCCTCCC

TTTCGACACATGCTATGGTCCCCTAGCAAAGTTCATTTACTACGGTAAGTAGTCGTGTGATTCTCGCAGGTAACTATTGC

GAGTCTCCTTATACGGAGCAGATCATTGTGCAACCTTACGGTCGCCCCACGTCATCAAAATGACATAGACAATACAAAAA

TAAAAGCGAATCCTAATAAAACAAAGGAATTATTAAAACCGTGCATAGTTAACACTCCACTTGTTGTTAAAGTTAATAAA

GAAATACAAGTATACAATGGTCAAGGAGATGGAGAGACTAAATGAAAGGGATGGTGTTGAAATATACTTCTATTAAAGGC

GTCGACAGTCATTTTCTTTCTAAGAGGATTTATAGATAAGTATAATTAATTACATCTAAAACAGAAAACTGCTGCGGGCT

CCCTCCCTCCTATCCCCCAATTGCTGCTTCGATCATCGATCGGATTAGGACCTTACCCCCTAATCCCCACTTTTTCAAGC

TCCTCCCCTACCCTCCCTCTAATCCCTCTAATCCCCTTAGCCGCCGCGCGGCTGCGGGTATCCCTGCGCTAAGCCCCCGC

AAGCGCTGCTGCTGCTGCTGCTGCAGCAGGCTAGCCATTAGGCAGCCTAGCAAAGGGAGGGAGGATACCGCGAGGAAAAG

GAGCTTACCGCACCTGAGGGAGCAAGATACTCCTTTATCTCTAAAGCCCATAGAGGAAGAAGAACCCCTCCCCCACTGCC

TCCCTCCCTCCCTCCCTCCTCCCCGCGACTGCCTTCCCTAAGGGTTTTATTAACCGAGGGAAAACAAAAAAAAACCACCC

CTCCTTAGCCCACCCTAAGGTCTTTCCCTTTCCTACAAACTACGCCGCCGGAGGGCAAGTTTTTCTACCCCCAGCGACCC

ACCACCCCTGTGCCTCCTTTTTTCTAATCCCCCTTAGGATGGGAGCTAAGCTGACGCAAAACACTCACTCGCCACGGAAG

AAGCCCCTCCTTCCCCTGGTATCCCCTAAGAGGAGCATATAATCCCCTAGCTAAAGAGGGATAAGCCCATAGGAGGGAGG

AGAAAAGCTCGTTTTCTTTATTGAACCCCCGGAAAAATTACATAAGTTACGATCCCCTCTATGACACCCGGAGTATCCCC

CTACGGATACCCCCGATTAGACGGGATAAGCCACTAGTTTCCTGGCCTTCCTCAAAACCACCTAGTACCGCTGGAACTTA

TTCCCGGAGGGCTCTCAAGGGATAAGTCCCCCTACGGCTGCTCGAGGAACCTAGTTCCTCGGCAAATTTGGCGGAGGGAA

ACTGTTGCGCGTTGGCGTCCCCCAAAACTAACTCCACGGGCACCCTCCCCTAAGGCGGGAAGCTGCAGCAAGAAGAAGTT

CCCCTCAGCCGAGGGACGGCTCCCATCCCCTGGGCGGATTACCTAGTTCCCCTGCAGCGCTTCCTAAAACTGCTGCAGGG

CGAGAGACAAAACCCCGATCCCCGTTGCGGTGCGTCCCCCGCCAAAACGAGTTTTGTGGGACCCCTGGGCGGTCCGTCCC

TCAAAGCAAGTAAATCCCCGGGATATTTTGCTGCAGCTGCTTTGCAGCAAAATTAAAAATAAAACCGAGAGATTGGGATT

TTTCTATTTTGCGGAGCCCCAAATTAAAACCCTCCCCGCCGCCGCTAAGCGGCTAATTCTCCTTGCGTAGCTGGGAGGGA

AATGAGGGAGGGATTTTTAATTACCTCATCCTAGGAGGCCTAGCTAGTTCCCCTCCGCTGGTTCCTCCTCAACGAACTTT

TCCCTCTTATCCCCGCGGGATCCCCGATTACAGCCGGAGGAACCTGTTGCGGTACGTCCCCCAAATTGTGGCCACGAACC

CCCAATCATCGGGGAGTTTGGGTCCCTCAAACCTGTTTGAGGAACGAAAAACCCCTAGTTTTGTGGGACAACGAGAATTA

ACTCTTTTGTTGTCCCTCAAAACTTTTGAGGCGGAACGAAAACCACTAGTCCCCTTGCTAAGAGGGGATAACCGGATAAG

GAGGAGGAAAAAGGAAGCTAAGCCTACCCTCTCTTCTTTAGCGGTATCCCCGAAGGGCTAGCAAGCCATACCCGGAGGAA

GGTCTTTTAAGGCTCCTTTAAGTCTTTCTAAAATGGAGGGACCTATTTTTTAGCATCTCTTGAGAATCGAATCATCTCCT

TTTTAGATATCTGTTTCGTAGGGAGAAACGGATTTCTCCCTGTTCTTGAGGTAAGAAACCTCTTCTATTTCTGTTATGCA

CGAAGTGCCCATATCATGTAATAAGAACTTATTTACCTAGTCCCCTTTCCATAGGGGTCAGCTGAGAGGAGGCGAAACCA

CCCCCACCCACCACCTGGCCGGGACCCCCTCGGGGAAAAAGGGCTAGTTTGCAGGAAACAAACTTGTTTGTTTTCAAAAC

AAGTTTTTGAAAAAACTAGTTCCTGAGGGGCCCCAGGAACTGGTCCCCGAGGTGGGAACATGAAGTCCCCGGGACTAGTT

TTGTGGGCCACGAAACCCCCGTTTTGGTCCCTCAAAACAAGTTCGTTGAGGGAGCCCACCTCGACGATGAAAAACCACTA

GTTCCCTCTACGAGGGATCTACTCCAAGAGATTCTCCAATTATCCCCGGCGATCCCCATCGCCTGAGGCCCCATGGTTTC

TTTTAATTAAAAAGGGGAGAGGGTTTTAATTAAATTAAAACCCTCCCCTTCGAATAGGGAGAGGAACAAGTTGCCCTCCT

CCGTCCGGCGGGACTAGCTGGCGAACTTGTTCCCCTAGAAAATTTAGCCGAGGGACCTCCCCGCTTTTCTTCTGTCTTTT

CATTGAAAAGAATAAAAGCTGCAGGCGGGCGGGATTACCTAGTCCCCTTAGCGCGCCCTGCGCGCCGGCGCTTGCTCACG

AAACCCCTGGGCGAGGGACCCCCGCCAGCAAACAACCTGTTGCAGGGCCCGTCCCCAAATTGTGGGCCCACGAACCCCCA

ATCGATTGGGGTTTTGGTCCCTCAAAACAAGTAAGTTTTGAGGAACGAAAACTAGTAAAGAGGGAAATATTACCCTCTAA

CTCTTAATCCCCTGCGCGAAGGCGGGATAATAAGGAAAAAGAAACCCCCAAAATTTATCTTTATAAATGCCATGCCCCAT

GCCCCACTGGCCGCGCGGTTACGAGTCAGGTCGGTCTTGATCCGACATATTCTTTCTTGACAAAAGTTCCTTACCTGTTA

GGATACTAACCCGAAATTTTGGTTTAACTAGTTTTGCGGGGCAACGAACTCTTTGGTTTTGCCCCTCCTAAAACAACAAG

TTCCAAACCCCTTTGGGGTTGCATCCTTCCTCTCCAAAGGAGAGGAAGGGATGCCAGAGGGACAAAACTAGTCCCAATCC

CCACCGATCGATTGAGGGATTGGGATAAGTCCCTCGGAGAATCGGGGACTAGTTTTGGCCACGAAACCCCCGTTTGGGTC

CCTCAAAACATGCATGTTTTGAGGAACGAAAACCCTATTCCCTCTCCTGGGTACTCCCAATCAATCAATCAATCGATCCG

ATCCGATCCGATCCGATCATCCGATCGATGATTATCCCTCCCCTTTTCTTTTCTCCCCTCGAGAGAACTTTATTTTAATC

CCCGGAATTCCTTTAGGAATCCCTCAGGGATTCCGCGAAGGGAATAAAAGTCGCCTTCGGTTTGGAATTTGTTTTGCTTT

GCAAAATATAAAAATACGACTTACGATTTCTATAAAAGTCTTTTGAATTAAAAGGGGAGGAGGGTGGGTTTTAAACCCTC

CTCCCCTCGAAGGCCGGGAGAGGTAAAACCCTCCCCTTCGATCGGGGAGAGGTTTGTTTCTATTTTGGCGGAGAAAAATT

AAAACCCTCCCCTTCAGGGGAGAGGTTGGTTTCTATTTTGGCGGAGAAAATTAAAACCCTCCCCTTCGGGGAGAGGAACA

AGTTCACCGATTGGACTAGTTTTGTGGCCCACGAAACCCCCGTTTTGGTCCCTCAAAACCTAGTTTTGAGGCGGTACGCA

AAACCACTAGTTCCCTCTCAGGTACTTATCCCCATTGACACCCCTTTTCTTTTCTCCCCTCGAGAAATAACGGAGCAAAC

TTGATTTTTAATCCCCGGATTCCTAAAAAGGGAATCCTTCATTCCTTACGATTCCCCGCAAGGGAATAAAAGTCGCATTC

GGGTTTGGAATTTTTCTTGCTTTGCAAAATATCCAAATAAAAACCCCAAACGAGGATTTTGGGTTGGGGTTTTTACTTGG

GATTTTAGAAAAATTTTGGAATTAAAAGAAAAGGGAACCCAGACAGAGGGTTTAAAACACAGAAACCCTCTCCCCTTCGG

GGAGCCCCAGAGGGTTTAAAACACAGGAAAGAAACCCTCTCCCCTTAATCGATCGGGGAGCCCTGAGGGTTTAAAACACA

GGAAAGAAACCCTCTCCCCTTCGAGTTTTGGAATTTTGGCAGAGCAAAATTCCAAAACCCCTCGGGAGCCCCGGAGGGTT

TTTAAACCCTCTCCTTCGGAGTTTTGGAATTTTGGCAGAGCAAAATTCCAAAACCTTCGGGAGTTTTAATTTTGGCAGAG

CAAAATTAAAACCCCCTTAATCGATCGGGGAGCCCTGAGGGTTTTTAACCCTCCCCGAAGGCGGGGAGAGGAACCAAGTT

CCCGCCACCCGTATCCCGCCTATCGGCGCCGGATTTATCGGGGACTAGTTTTGTGGCACAAACGGTTCTTGTCCCTATTC

AAAACAAAACCCGGATTTTTTTTAGAAAACCCTCCCCGAAGGGAGGGTTTTAATTTTGGCTCCGCCTAAATAGAAAAATC

CCTCCGAATTTTTATTTTTAATTTTGCTGCTGCAAGCTGCAGCAAAAATATCCCTCGGAGAGGGGATTTCTAGTTCGTTG

AGGAACGAAAACTACTTCTGGTTATCACTATAGAACCTGCCCATAGCTAATTACAGAATAATAGATGTATATATAAATTC

GCTTACCCATACTCTAATCTTTCTTTAAAGTTATCACTATAGAACCTACCATAGCCAATAACAGAATAATAGAAGTAATT

ATCAATCATATACTATAACTTGTATACATAATGTTACCTAAGGTTGTTATATGACTACTTTCTGATAAACAACCGTCTCA

TGATTTACCTGTTACATAAAAACATTCTCTTCTAAATCTGAGCTAATTAAAGACCCCCATTAAATGTACAATGGTAAAAA

TTATTAAAGATCGCTTGCGCATCAAAAAATTTGATATTTTTCAAGAAAAGAGATAAAGTTATCTGGTATCATAGGATAAA

AGTATAAATAAAAGATATTATTATAAATATAGCTAAAGGTATACTTTTATTAGTATTACTTAATAACTCACTTATTCTAA

CGTTTATTAACATTAATATAAATAGAAATAGTATAGAAACTGCACCTACATATACTAATAAGTATGAAATACCTATAAAA

TTTAATCCTAATAATATAAGATAACCTGCAATACTAATAAATAAAGTTATCAAAAATAAAACCGATGAAATGGGATTTTG

GCTAATTATTACAAATATACTTGATAAAACTGAAGATAAAGCTAAAAATCTAAACTATCTATTATGTAACCATTTGTATA

TGTTTCATGTATTTGATATAAAATAATAAAATAAAAAGGTACCTCCCATTATCGATCGGTGGTCCCTCGGAGAGGGAAAA

TGTATTAATTGCTATCTATTAGAGTTATTAAATTATTGAAGAAATAACCGCAAAATTATTGGTGGCTTCCTCCTCCTACC

CCCTTAGTTTTGGACGCCCTGCGGATCCCCCGCAGGGATCAAAAAGAATTTGTTTTACTGGCTTTTGTCCCTAAAACAAA

AACCTAAAAAATTCGCTCTTTTGGGATTTTTTTATTTTTCAGTCGGTTTTTATCCCCTGGAGGGAGGGAGGAACGAAAAC

CTCGCATCCCTATCCCCCGGTATCCCCCTAGGCCCCTAGGTCATAGAGGGATAGTAAAAGAGGATTGGATTCCCACCCCT

TGCCTCCTTTCTTACCGAAAGCTAAGAAAAGCTACCCCTAGTTATCCCCTGCTGGGTGCCGTAGCCCCCGATTACCCCCA

CTTGCTAATCCCCTCCCTCCAGGGATAACTAGGCTGAGGTCCCCCTGGAGCTAGCCGCACTTATCCCTCCTGGCTCCCCT

AGCTAGTAGCTCCCACTTCCTCCCCTAATCGCTGCAGGCTAGCCTCCCCCTAGCTAGGTCCCTAGATAAGCTACCCCCTT

CCGAGCTCCTACCCCCGATTAAAAAAGGCCTAAGGTCCCCGGGAGGTGTTTTTGGGAAAATTAAAAACACCACCCCACCC

CCAATGGGACGCCGATGCCGATCCCTCCGGGGTTTATTTTTCCCCGAGGTACCCCACCCCTGTTTTTATGGCTTATCCCC

TGCGGAGCTACGGCGGGCAGGATTAGCTAGCTAGTTTCCCCTGGCTTCCTAAAACTTTGAGGGACGGCCCTGCAAACCTC

CTGCCGCTGCCCCCGTCCCCCAAAAACTTGTAGCTGCTAAGTGGAGGTAGTTTTAAAACTTGCATTTCTTACCCGAGGTA

AGAAACCTACCCCCTTCCTCCTAATCCCTCCCCTAGGGATAAGCAGCAAAGGTGACCTCCCCTTGGAGCAAGCGGGAGTG

GCCATTTTTAATTGCTGTATACCTAGCGGCTGAGGCTAAACTGGCTAAAGGGATAAGGCCTTAGTCCCTTCATCCCCTGC

GCTAAGGCGGGATCCCTTCTCCCCTCCGCCTCGAGCGGAACTAGAACCCTCCTCCGGGACTAGTTGCTGGGGACGCCCTG

CAACTATTCAAAACCCCCGGTCCCCGTAGCTCCTCCCCTGGAAGTCCCTCCCCCTGGAGGTGTAAAAATCCCTTCCCCGA

GGGGACTAGGAGGTACCCATTGTAAAAAATCCTGCCCCCGGAGGTACCCACCCCTTTTTACTGGCTTTGTCCCTCAAAGC

ACCCCCTATTTATTACCAATTCACCCCCGATAGGCCCGGTGGGTGGAGGCCCTGGGATAAAGAAAAATAAAAACCCCCAA

ACCAAGCCTAGGCTCATCCCCCATGGATTTGGTTTCTATCCCCATGGAACCCCGGAACCCCCCGGAGGGAGGGACCCTCC

GGGGTTTTATATCCCCATGGTCCCTCGGGGTTCCCTCCGGTTCCATGGGATTGGGTTTATGCTCCCCGCCATTTGGTTTT

AGTGCTCGAGGTACGAAAACTAGTAGTTTCCCCCCCGGTACCTAAAACTTTGAGGGACAAAACAAACCAGCTCCCTCCCT

AAAATTAGCCGGAGAAAAGGGGCTTGCTCCCTCCACGGCTGCCTACCCCCGATCTCTACCCCCTTCTTATCCCCTGGCTA

GGCAGGCATTAGCTGCCTAGTCCTACCCCAATTCTTAGCTACGCCTCCTAGGGATGAGGATTAAAAATAAGAAATTTCCT

CATACCCTCCAGAGGGAGAATTAGAATTCCCCTTGGCTCCCCCTTACCCCTGGCCTAGCCCGCCGTAGCTAAGCCCTAGT

TTTCGTTTCTAAAACTAAAACCCCCGGAGACCCATGGGGAATAAAACAAAAAAATTTATCCCCCATGGAACCCCCTCCGG

GAACCCCCGAGGGAACCCCCGAGGGGACCCCCATTTTTATCCCCCATGGAACCCCCTCCCATGATAGGGTTTTGGCGGGA

TTTCTTTTTATCCCCCGAACCCCCGGAGGGAACCCCCGAGGGGACCGCCCATTTTGATCCCCATGGGAAACCCCCGAAGG

GGACCCCCGGAGGGATTGGGTTTTCTATCCCCATGGGAAACCCCCGAGGGGACCCCCGGAGGGATTTTTTTTTTTTTTTA

TCCCCCGAACCCCGGAGGGAACCCCCGAGGGGATCCATTATTTTTGATCCCGATCCGTTTTGAGGGACAAACCTGTTCCC

TGTGGAGTGCGGCTACGGAAAAACTTTTTTACGCAAAAACTTTTTATCCCCTGTGGGATCCCCGCAGGCGTCCCCCAAAA

ACTACAGTAGTATACCCCCTTATATATAGGAAGAAAAGGAATTGAACCTTCGACAGTTATTTACTATTAAGTTTACAGCT

TAACCCGTCATACCAACAAACGGAACCTTCCTATTAAAGAATACATTATGTTTTATCCCCTGCGAGCTACGCGGGGATAC

CCCCGCCGAGGAGGTGATAATTTAAACCAGAGCTATTAGCTTCCCTAGGGAGACAATAAATATATCTCCCAGCGCGTAGC

CCGGAAGGGTTATTTATTTACAAAGTTTAAGTCCCCCTCGGAGAGACTTACTCCTCCACCCCGCTTTCTTTTCCCACCCC

ACCTAGGAGGTTGCTGGAGCTATAAAAGATATAAAAAATAAGCCAAGAAATCTCTTTCTTTTTCACCCTCCACTCTCGTC

CCGCTGGAATCGCCGAACTGTTCCTCTCCCCTTTTCTTTTAATTAAAAAGCCAGCCGACTTCTATTAAAACCCCCAAGTT

ATTTTCCCAAGGGAAAAGAAAACCCTCCCCTTCCTTCCCGAAGGAAGGAGGGTTTTCTATTTGGAGATTTTGCAAAGCAG

CCAAAATTTAAAAGACTTTTATTAAAAATCCCCAAGTAAAGTAATTTGATATTTTGGCTGCAAAGCAGCCAAAATTTAAA

AAGACTTTTATTCCCTTCGCGGGAATCCCCTGAGGGATTCCTAAAAGGAATCCGGGGATTCAAAATAAAAGTTATTTATC

CCCGAGGGGAGAAAAGAGGGGACGATTGGAGGTCCCTCGGAGGTAGAATTTTTATACCCTCTCCCTGCGCTTCGCTTTTT

CGATCCTCACACCCTAATTCTCCCCGAGGAATATAGAAAAAGAAAACATCTAGCTAGCCAAAAGTGAAAAATTTATATCC

ACTGACTGGTCCACTTTTTTTTAAAGGAGAAACCCCCGAGAATTTTCCCCATGTACGATTAACTTCCGTAAAGCTCTATA

GAGAACCTGAGCAAGAGATTTTACAAATAAAACATATCTATAGCGTTTGCTTATTTAAAAGAAAAGTCCCTCGTCATAAA

GATATATCCCTCAGTAGTCTAGTAAGCCCTGCGGGTACCTAAAAACTAGTTACCCTCCGAGGCCTTGCGGGAACCTGTTG

CTGGGCGTCCCCCAAAAACTCCAATTGTCATCCCCCAAAGATATATCTTGGGGATAGTTTATTCCCTGCATATTCCCCTC

CGGGAGAATTGAGGGATGGCTAAGTTTTAGTTAATCCCCATGAGCAAAACCAAACCTGTTCTGAGGCCGGGAGGAACGAA

ACTGCTTTCGTTTTATCTCTAAAGGCGGCCGGCCATATGAGGAAGAACTCTGCTAACTAGCCTGAGGTGAGGTAGTTCTT

AAAAACCTGGCTCTTTCCCCTTAGTCCCGCAGGAAGCGCAACCTAGTTCCTCCTCCCCTTTTCTTTTAATTAAAAGACTT

TTGATCCAAATCCGTCGTCTTGGGATATTTTTGCAAAGCAAGAAAAATTACAAAAGAAAAGGGGACAATGATTGATTGGG

TCCCTCGGAGAGGGAACTAGTGGTTTTGGTGGCTCCCTCAAAAACCCAAATCACTTGTTCAAACCCCAAAGGTTGTTTGC

ATCCTCTCCTTCGGAGAGGACGCAGAGGGACAAAACTATCGGGGTTCGTGGCCTCAAAACTAGCAAACTTATTTTCCCCT

GAGGGATTAGGAATTTTCCCCTAGCACTGAAAAGAAGCGGGAAACCTGACCCTCCCCTGGACTTTTTAAACAAGTCCCGC

TGCAGGCCGCTTGTTCCGGCTCTGCCGAATCCCCGGATCCGCCGATTAGGGATGGCACGCCCTACGGTCCCTCCCCGGTC

GAGGAACTAGTTTCCCCTGGCTCCTCAAGAGGGACAAAACCCACCCCCACCACTTGCTGGCCGTCCCCAAAAAGTCCCTC

ATCCCCTGCAGGGATCCCCGATTTGGCTTGCGCTTTGGTTCCCTCGAGGCCTTGGCGAACCCTCCCATCCCCCGATTGCA

GCAGGCCGGGATTACCTAGTCCCCTTCATCCGCAGCAGGGGATACCCCGATTAGGCAGGGAACTTGTTCCCTCTGCCTAA

TCCCCGATGATTATAGGCGGATGCCCCATACCCCCGCACCGGAGCGCCCCCTTAGCACGCGGATTGGACTATTCTTAGTT

GTCTCCTAGTTTTCGTTCCTCAAAACTTGTTGTTTTGATGAGGGACAAAAAGTTTTGTCCCACTGGCATCCCTCTCCGAA

GGAGAGAACGAACAGGATGCAAACCCCCAAAGTTTGAACTAGTTTTGGACAAATATTTTTGTCCCTCAAAGCCACCCCCT

ATTTTATTCCCAATTCACCCCCGATCCGATCGGGGTGGAGGGCCAGGGATAAAACCAAACCCAAGCCCGTAGCTCATCCC

CCATGGATTGGGTTTTAGGGGTATCCCCATTTGGGTTTTGGATCCCCGGGATAGAAAAACAAAATCCATGGGATAAAAAT

CCGGGTCCCCTCCGGGGTTCCCTCCGGTTCCATTGGATTGGTTTTATGCTCCCCGCCGCCGCCATTTTTTTGAGTGCTGG

AGGTACGAAAAACGTACGTGTTTTGGGGACCCCCGATCCCACAGGGATAAAAAATCCCCGGAGGGAACCCCCGGAGGGAA

CCCCCGGAGGGGATGGGGTATAAAAACACCCCATCCCCCAAAAATGGGGAAACCCCCGGAGGCCGGACCCCCGGAGGGGA

CCCTCCGGTTTTGGGTTTATTTTTTATTAAAACCCGGAGCGTAGGCTCCTCCCCCTGGAGGTCCTCCCCGAGCTACGCGG

GAGGTGGTGGTGTTTTTGTAAATCTTCCCCTGGAGGGCCCTACCACCCACCCCTTTTTTACTGGCTTTTGTCCCTCAAAG

CACCCCCTATTTTATTCCCAATTCACCCCCGATCGGCCCGGTGAGGCCCTGGGGATAAAAAGCCAAAACCCACAAAACCC

AAAACCAAGCCCTAGGCTCATCCCCCATGGATTGGGTTTCTATCCCCCGAACCCCGGAGGGAACCCGGAGGGAGGGACCC

TCCGGGTTTTTATCCCCATGGGGTCCCCTCCGGGGTTCCCTCCGGTTCCCCATGGGATTGGGTTTTATGCTCCCGGCCAT

TTTTTTTAGTGCTCGAGGTACGAAAAACTAGTCCCCCTGGAGTATGCTGATCCCCTAGGGATTAGCTCCCCTGGACTGCT

TATACTGCAGGCAGGCCCTGGCTCTGCTTACCTTAGCCTCCTTAGACTCCTACTATGCCTTATCTTGGCCTAGCCTCGGT

CTTTTAAACACTCGGAGGAAGCCGCAGCCTGCTAATCCCTCTATGACCGAGGGATAAGCAGGCTTTTTTCAACAAGTCCC

CCAGGAGGCCGCTGCTTGTTACCGACTCGAGCCCGAGGGGACGCCTCCCGCATCCCCTGCGCGTAAGCCCATTCCCTCCT

GGGGATAAAAAATATAAAAATAAAAAATCTCTCGATTTTTTTTTTTTAATTTTGCAAAAGAACTTTTTACATAAAAACAC

CCCTGCCGGAGCCGGTGGTGTTTTTGTAAAACCCAAATCCTTCCCAGGGGACCCACCCTTTTTACGGACGAGTTTTCCGG

GGAAAACTTACTGGCCTCCCCTAAAAGCAAGCTCCCTCGGTTGAGGTACGAAAACTCCTGGCCCTCATCCCCTCTGGCGA

GGGCTTCCCCGATGCACGCAGGGATTCGGAAGCCGGTACTTGTTCCCTCGAGCCCGGAGGGGCACTTGTCCCCCTTACGC

TGTAATACCCCCTGCTGGCGAGCTACGCAGGGATAAGAGGGAACTTGTTTTGTGGGACAAAACTTTTGGTCCCTCAAAGC

AGCACAGCAGCCTGCCCCCAAGTGCAGGGAGGTACGAAAAAAACGGGTTTTGTGGGACGGCCTGCCGCCGATCCCACAGG

GATAAATTTTTTGTAAGTTGAAGAAGTTTAAGTTGAAGTTGAAGTGGAAGTGGAGGGATTTTTACTGGCTGCTTCCTGGC

CGTCCCCTCAAAGCCCTCCCCTATCGATTAGGGAGGGATAAAAAGAATGGCCCGTAGCTCTCCCCGGATAAAAATAAACA

ATAAAAATCCCCCAAACTAAAACACCAACCAACCTCTCCGCCGATCTCCCCTAGGGAAACCCCCGGAGGGGACCCCCGGA

GGATAGAAAACCAAATCCCCTAGGGAACCCCCGAGGGGACCCCCGAGGGGTTCCCATTGCATTGGGAAACCCCGGAGGGG

ACCCCCGGAGGGAGGGACCTCCGGGGTTTTTGTTGGTGGGTTTTGGGATTTGTTGGTTTGGAATTTTGGGTTTATTTTGG

AGGAGTTTGAGGTCAGAAAAACTAGTCCCCCTGGAGTATGCTGATCCCCTAGGGATTAGCTCCCTTGACTGCTTATACTC

GCAGGCAGGCCCTGGCTCTGCTTACCTTAGCCTCCTTAGACTCCTACTATGCCTTATGTATCCCTCTGCTGCTGGCTTAT

CCCCTAGGGCCCTAATCCCCCGATTGCTAGCAGGGTAGTCCCGCGAAGGGCCCCTAATTCTCCCCGGAGGGGGAAATATG

AGGAACCCACCCCATTGAGGGATTTTTAATCCTCATCCCTATTTTATACCCCCTCCCTCCGGGGAGGCCGTAGCTAAGAG

GGGATGGCTAAGCCCTCCGGCCCTGCTGGAGTAAATAAGGGGATCCTCCTAGCGCAATCGAAAACCTGGAGCGCTTAAAA

CACTCCATTTATTTACCCCCATTAGGCTTTCTAGCGCAAGCGAGTCACCCTCCTGGGGATAAAAAAAGAAAAATTTTTTT

AATTAGGCAAAAGAGGCAAAGCGGTTTTCTAGCTGGATACACCATAACGCTAGATCGAAAAAAAGGGAATAACCATTTTA

CTGTGAAAATGGTTATTTATTAACCATGGTTTAATTGTTAAATGTTTAATAAATCCCGTGCAACTTCCACTACACGAACC

ATATTTCGACTTAACACTAATTAAAGTCACGCATACGAAGTTTTTCCTTATAGATAACTAGAACTAATTATCTAGAATAA

AAATATAAAACCAAACTTATTGCACACACTTTTTCAGCGCCTGACGAGTAGTTAGTACCCAAATGAGATCAAGGAATTTA

TTTATCATGCAATAATATTCAAATTTATTTTCAATCCCTAGACTTGTTTAAACCTGCTTTAATCTTTAGTATTTTATCAT

TACCTTCTTTAGTTAAATGCTCCTTACGTTTCATAATTTCAGCTACAAGACATCAATCTGCGTAATCTAAGGCTTTTACC

CCTAAGATAGGATATTTTCTAAAAAGGGCATGATGATATCATAGTTGTCAGAAAATTTAGTTACCCTGTAATGACCTCAT

CCTTGTTTATCCATGTAATGTCCACAACCTAAGTAAGTTACTAAGCTTTCCATTAAGTTTTTATCTCTATCATGCTGTCC

AATTTGAAAATTTAACTTAGCATTTACTCCTAATTTATAAACTGAAGATTTACTAACATCTATATGGAATCCTCCGTCAC

CTGAAGTAAATCCAGCTATTCAATAGGGATCCGGAATAGAACTGTTAACTAAGGGTCTAGAAACAGGCTGTGTATTAGGA

AGGCTAAAATTAAACTCGGGGATAACCCTCAGTTCAACCTTGCCCTAATATTTACAACATTTTGTAAACCTTGCTCGCTT

AAATGAAGCTTTTACTTTTAATATAACAATTTGCCTAAATAATAGGTAATCTGCTTGTTTTGAGTAATCAAGCTATAATT

ATCCAAATGGGGAAGACATGACTAATTAACTGTTCCAAAGATGAGACACGTAATTCAACTTTATTTCTTGATTTATCCGT

AACTATGTTACCAATTTCTCCAAAATATGTTCTTATCATGTTTAACATAAATAAGTCTTTACAATGAACACATATAGAAA

ATCGAGGTTCTACAGCGTAACCCCTACAACGAGGATTTTTCTGATAGTAGTGCTAAATGATCCCTCCGCATCTATGTAAC

CTGTGATAAACCAAGGATTAACTGATTGATTGTTGTTTAAATGACTGGTTTCCTGCTTAACTTCTTTTGGTTTTAGATCA

ACAGGTAAAGAAGAATATGTTCTTCATTGAATATTACGTTTAGAAAGGTTTAGGGGTGTTATTTGGGGTTTTTCCCTACA

CACCATTAATACCCTCCCTAGCGTAGCAAGGGAGGGAAAGGTGTTAAATCCCTCAGAGTACACCTTAATCACATATTATT

TACATGATAACTACCGTCTACTCGTTGCTCTTTTACAGAGTATATATTTATATTGTTAGTAGGATGGTTTAATATATACT

CTGATTTAGATCCGCGATTGCTCACTTTCTTTCAAAAGTCTTCAAGCTTATTACCACACATGAGGGATTAGGTCATCCAT

ATCTTGATCAAGATCAAGTACTTGGTACTTGAAGCACTAGAGTGTCCCCGGAGTTTGATAGTTTATCCCACTGAATCGAT

TTCCTAATTCAAACAGCAAGATCCACCGTGACGTTCTTATTCACGATTACTAGTAATTCCTTATTCATATAGTCGAGTTT

CAGACTACAATCCTTGTTATATCCTACTTTAGCATGATTAGCTCAAATTCGCATTATCGCATCATTTTGTATAGGAATAT

GTGGCACGTCTATCGCCCACAACATTTCATAGCGATCAAATTTACCGTACAAAGAAATATGCATCACCTCCTACCCACCT

TACGCCTGCTTTTAGAGTAAACCTATGTTAAAAGCCGTCCTTTATAATGGGCGGGCCCTACTTATGTAATATGTTTTTCT

TCCTTTATTATACGCAAAATATTATTATAACCATCTTGAGTCAAATGCCCTTTATTTTTCATAATGATAGACACTTTCTT

GAATACGGAAAAATCTAATTCTTTACACCACTTATGCGGTATTTTTAAAAATGGTATAATTATCTCTACGATATGTGTAA

AATTAGAAACTTCCAAATGTACAGAAGAATCATTCTGAGTATAGACATATTTACCTGGATCTAAGTTAAAGAAAGCAGAC

AGACCTTGAAGAATTCCCTTATCTCTAAGGTTTAAACCCACTCCAAATCTTAATTGAATTCTCTTACCTGATCTCGTAGA

GAGAGAACTACTTATTTTGATATTAAAGCTACCATCTCCACTTATGAACCCGGCTATTCATAACGGATGAAGAGGACCTT

TAAATACGTATTCTGGTCTATTTACCACTACTACATCTGGAAAAGCTTCCCTTAGACTAAGGTTTAAACCTAAATTTAGA

GAGGCCTTTATCCCTATTATTTCCAATAAACCCTTTTATTTAAATGCTCTCCACCTTTTATCAGATTAAATGCTTTTTGA

ATAAAGCATAATCTGGTGCCCTTGGCTGTCATTAGTGGGTATTTATCAAAATGGTCGATGATAACTTGTAACTCCTCAAT

AGAGTCTACTCTATATTGGCGGCTATCTTTACTATGTTTATGTATTCTACCTACACCCATTGTAAATTTTATTTTTCTAA

TAGAGCTGTGTCTTTAATATGGAGTCCTATAGCAACGAAACCCGTTTAACTCTTCAATTGGTTTTATATTTAATATTATT

TTGAATTAAGATAGAGAAAGAACCTTCTGCATCAGCAAACCCTGTGAAAAATCCTGGGCTTATATCATTAGAAGGCATAG

ACATAGTGGTGAATTTTGCAACATCTCTTTGCTTAGAGAGGATTTTGACTTGAAAATTTCTTTCGGAACCCATTAGAGTA

CACCTTAAATGCATTGCCCTTGGCTTTTTATGCATCAATTGCCATCTACTCGTTGCTCTTTTACTGCTACACTACTTGGT

TTTTCTGATACAAAAGCGTATCGAGAATAAGTTAAACTGATTTAGATCCGCGATATCCCATTTCATTTTCAATCATCTTC

TGACTTATTACCGTACCTGAGTAATTACTTCAGCCACTTATAGTCTTTCGACCATCCGCTTGGTACCAGAAGCTTTAGGG

AGTCCCCGGAGTTTGGCGATTTTTCCCACATGAGCGAATTCCTACTACGCTCGGCAGGCCATGATGACTTGTCTTGCCCC

AGTGATCATATCCGATTTAGATGGTGAACTGCTTTATTTTCATAATTAAAGCAAGGGTTTCCGTTAATTTAAAGGAGTTA

ACCGGCATCTCACGACATTAACTGAAGACAGCCGTGCAACGCTTGTAATATCCTGATAGATAATATTCAAGTTGTGGTAA

GGTTTTCGCGTACCTACGAATTAAATAACATACTTCACTACTGGTTTCAGTAAACGGTCTAATGATTCAATTTTTGAGTA

TTCCCCGCTGGGGATTTTAAGAGTTACTATCACTGTGTAAATGACGATACACCGTGCACGGTTTTGTATCCATCCTTCCT

CTTTACGATCTTGCACTCGTCTCAAAATCCGACTGTACATTTGGACAGACATATTCAGCCTAACCCTTGTGATCCAGTCC

GTCGCGACATTTACAACTGCCGACGGTCTTTAGTTATAATGGTTCATCCATTAAAAATCTTACTTACGTATATTTTAGGA

TACTATTGACCGTTTTCACCTAATTTTTCTACAAGTATGGAAACTATTCGATTGTAACCTTCTCTTAGCTCCCTCTTTTC

TTTTCCCATATTATTGAAACACTCCTATTCTTTTAATTTTATAACCCTTAATTTCCACCCACTCTTTTAGTTCTTGTCTT

TCAATTCTGGTTTTAAAGTTCTATAACCTACATTCACCGTTGAGGCCTACTTGCTTTAAAGTTTCTAACAGTATAGTATC

TCCATCAGGCTTTTAATTTCATATACACACCCTGACAATAAGATCTCGGGTGCTATAGAATTATCTAAGTGTCTAACACG

TCCATCCTCAAGATACTCTATGTAAGGAACAGCGTTTACTATCTTATGTCTTTCTTCTAATGTAATATATTCTATTTTTC

CCTTAAAGGTGGATAGTCTAAAATTATTCATATTGTAAGATAGTTTGAGGATTAAAGATCTAATCTCTTCACTACGATGT

GAACCTTTATAGACTGCTTTACAAATATCTGTGAAATCTAAAAATCTAAGCCTTTTTGGTTCTAAATTTAACATCTTTAA

GATAAGGTATAATATAATTGTTCAATACTTTAATATTTTTAATTACTAATATTATACTCGGCTTCGGCTTAGGCTTAGCT

TTGGACGGATTTCTAGTTTCTACAAACTGGTTTTCTTCTTCACACAAGAAAGAGCCTCTAACCCTTGCATCTATACTATT

CAACGCTATAGCTTTGGAATGTTTTAATTTGAACTTGGAGTACTTATCGAAAGCTAGATCAATCTGCAAAAATTCTTTAA

TTTTCTCAAACAAAGGTCATTGTTCTGCTGTGGCTGAGATAGACAGTGTAGGCTCAATGTCCGTTCTAGAAACAAAGAAA

GACCCATCTCCTTCAATATAGCCTAGCAATCAACCTTTAGTTATTACAATATTGTGATCCAAAGGCAAAACACTATTAGT

TCTTTTAGTGTTCATTGAATTTTTGAAGTCAAATATCTGATTTGTAAGAGTTTTGGCTTTATCTTGGTTATAAAAGTCTT

TTATCTCTTCCTTGGTATAAAAGAAAGGCTCTCTTAAATAAAAGATAATCAAGGTGTTTAGTTGAATTAAGGTTGTATTC

ATCAAAAATAGAAATTAATTTATAAGTTCCTTGTGCGTTTGTTACTGAAAAGATGCATTTATCCTTATATTGTCTAACTC

TTCCTATCCCCAGAGAATTTTTATATCTTCTAAAACATTTAAATCATCTTTGTGCAATTCAATACTAAACATAAAAGTGA

TTTTTTTATGAAATTTTCAGAGTTTACTACTGGATTAATTGAGAAACTTGATTCTGCATCACAGAACCCTATAAATCATT

GTTTATTAAAATCTGCAGATTCTTCTTTCATCTTAACCCTTTCCCCACCAATGCTTGTCGAGTAATTTCGATTTCCCCTC

CACCCTAATGGCTGGGCTGGGCTGAACACATACTCGGATAGATGGAGAGTATAAACAGTCTTTCAATAATATAGGTCTGG

AATTCACCTTGCAGAGAGGGATTCGAACCCACACTGCAATTTTTCATACACAAATTTCAGTTCCTGCGTTGCCACATTAC

TCTTGAGGTGGAATGCTTACATTTTTATTTATAGACTAAATTAAAATCTAACCTATAGCATTCATAATTTTCTGCTTGGA

CTACCAGGGTATCTAGTTGAGATAGGTAGGTCCTCTCGGACTTTCCCCTCTTAGAACCGTACGTGCCACTTTCATGGCAT

ACGGCTCAAACGTTATATGCGTCTTAATCATTCTACATATTGTGTATACTTATTTCCTTGTAAGGGTATATTTCCCTTGT

CATCTAAAAACGAAACCAACCTTTGTATATCCTTTTTACTTGATATACTTAACTGATAAGTTGTTTTGCTTTCCTTTCCT

ATATCCCTAAGTCTAGGAGATCTTTCCAACACATTAGGACCAAACTCTAATCTTCGTTTAATTATTTCTAGAGCTTCCCT

ATCTGTATGCTCGATAAAAAATTACATTGACTTTTATTGATATAGAAACAACCTTCACCATTAATAAACCCTATTATTCA

GTTATCTATATCGTAGGGAGCTTTCAATAGTCTTTCGACTCTATACTTTAACTCATTATCTCTAGTTCCCTCCCGAGGGA

AGAGAGGTTGGTCCCTATAGATAATAAACGTTCAGTCTTATAATGATTATATTCTTCTAATGTTTTAAACTCCTTAACAT

TATTTACCAGTCCGTCTTTTAATAAAAGATATCTTATTAACTGATTTTTGTTACTAAAGGAAATACATCAAATACATTGT

GGATTATACTTAGTAAGCCTGCCTTATCATTAACTGCTAAACGTGAGTCTGGTTTATTCATATATTCGTAAATAGTTCCT

ACATTATTTAACTTGTCCTTAATATTTTTAGAATTTCTGTATCTCTTTGATGTAAAGACAAATGATAAGCATAACCTACA

TTATATTTACTAATCTCTCCCGACTTTAAAACTCTTTTTTAGGGTATACTTGAAAATTTCCTTCCGCATCGTTAAACCCT

ACAAACCACTTGAGCCATTTTGCATTTATTTTATTAATTATTACTTTATTTTCCATGTCCTCAATAAAAATAGCTATTAA

TAAATACAAGGTTAAGGGGTATACACAATTTAATAACACACATAACATCTTTACGCAAGTGGGCTCCCTTTCGGGTTATT

TTTTTTCACCTCAAAGCCCTCCACTCCTTCCGCCGGAGGCAAAAAAGCGGAAATATATTCCCTATCCATTCCATTACAGA

ATGGCCTTCGCTTTTACGTTATCCTTTTACCTCTAATCGATAATTTACTTTACAGTAAATCCTGCTTCATTTTAGGACCC

CCAGGTCTCCTTTACGCTGACCATAGGCATTACCCTGTTCCCTAATAAAAACGCTCTTCCTTAGGTTTCAACTTTACTCT

GTTAATTATATGTTCATCTTCGCTTAACCCAGGACTCGTTAAGTGGCTAATTTATTATATTTTATAATATAACCTAGTTA

CTGAGGGACCTGTACTGGTCCCGAGGAACCTGTCCCTCGGGACTAGTTCCCTCCTTCCCCGCAAGTCCCACGCACTTGTT

CCTCGGACCAGTACCTGGCCCTCTGGAACTAGTCCCGCAGGAGGCGGTACTTGTTCCGCTCTTCTCGGGACAAGGTCCCT

CGAAGAGGGAACTAGTTTACTATCCTAGACAGAGTTTTAATTACGTTGATTCAATTTCTCTAACCATAGAAGAAAGCCAA

TACTATAAATCTAAAAGATAAGTCTAACAAACAAAACCCTAAATCTTCCTAGATATATAATACTTTTCTCTAGAGCTTTA

TACAAAACAGTTGCCCGTAATGCATATCTAGATAGACTAATGTAAGATGAAATACAATTGATAATCAATTTAGCACCTAC

CCCTACTTTTCGATCGGCTTGAAAAGACTGGGCGGTAACCCCTTTAAATAGTTATCAAGTTTTTATTAGAGCTTTTCAGG

TCGCACATCCTGTTCGCTACCCGAGCCTTCGTCCCTCAACGTCAGTTATTACATAAAAGGTTGCTTTCGCCTTTACCAGT

CCTTATGGTATGAACAAATTTCAACTCTCGCACTCCATAAATACTCACCTCCTTATATATAACTCTAGTATAATCGTATC

TTGCTTGTCGATAAAAGACGATTTTTACCGTCTAGGTACCCTTTAACATGGCAATATACAATCTGGCTAAAATTGTATAT

CTAACAGATTTATCATAGAATAAAGAGGAAAATTCTTATTCAATTCGCCTAATCCCGGAGGGATTCCTTTTAGGAATCCT

GACGGATTCCCGCAAGGGAATAGATTATCTACCTCTATTCATACCCTCTTTTATTTTTGTATTTTGGCTACACCCTCTAT

AGTTAAATATTATTTTTTTTCATTAAACCTGCTACAATACTAAAATCATAAAAATCCTTAGCTTTCTCACCAAGGATAGG

GTATTTTTAAAAAAGCTAAAACTTTATCAGTAACATCAGAAAATTTAAACGAACTCCGAATTCATATACATCTCTTTTGC

GACTTAATTTACCACAACCTAAATAGTTTATCAAACATTTCATCAAGAACTCATCACGTATATGTTGAGTTATAATAAAA

GCTAAATAAACTTGGTATCCTGTAGAAGAAAGAGCAGACTTGGCTATCCCTACCATAAAACAACCTTCAGCTGAAGCAAA

TCCGGATAATCCTGAATCATTTGGAATACTCACTAAAAATCCCGTATCCCCTACCCTTGAAGCAGGTATAATGTAAGGAA

ATTCTGCTTTCAATTGTTTAGATAAACCTCAATTTAAAGACGCTTTAAGAGATGCTATTTTTAAAATACCTTCATTATTT

AAATGCTCCTGTCTTCTTATTAACTCATAGGCGCGGCTTCTTTAAAAGCTCATAATCTCCCTTTTTTAGTTATTAAGGGT

ACTTGTCTAAATGTTTTATAATAATTGCTAATTCATCAAAAGTTTGAATTCTATATTGAATAAGATTTTTCCAGAGTCAT

GTATTTTTCCTACACCTAGATAATATTTAATGCTTTCTAATAATGCCCTATCTTTCACATGAAGGTTGATTTGAAAAAGA

GTTGTACTCTTCAATCAACTGCTCTATTAACTACTTTAGTTAAAGAAATTCTAAAACAACCTTCACCATCTATAAATCCG

GTTAAGAATCCTGGATTTATATTTAGACAAGTTTTGGGCTCTATACAGCTAGATGTAGAATAAAATCTGATCTCTTTTTA

TGTAATACCTTAGAAAGGATTCTGATCCGATAACTTCTTTCGAAACCCATTAGAGTACACCTTAGATGCATTAATTTTTT

AAAGGTTCAATGCATCAACTACCGTCTACTCGTTGCTCTTTTACAAACACGCTACTTATTCTCGTAAACTCGTTTGACTT

AAATCCGCGATACCCCATTTCAGTTTCCATCATTCATTTACTTATTACTATACCTGAATAATTACTTCAGCCACACATAT

ATTTTCATATACCGCTTAGTACAAATGATTTTAGGGCGTCCCCGGAGTTTGGTAGTTTAAGGCACACTTGTGATTTCCTG

CATCACAAAGCACCTGGTAAAGATGAATAACACTAGTCTTTTACGTATTACCGCGACTGCTGGCACGTATTTTGGTCAAG

ACTTGTAGATAGTTAATGTCATAATCAATTCTAATTCCTATATTAGAGAAATCTTAACACTTTACATTAAGATAAAAACT

ACCTAGTATTTTATTCGAGTAAATCGATATATCAAAGTAAAGTTTATTCCAACTTATATTAGTGTTTTTGTAGGCAACGA

CTATATTAAATTGTTGTAAACACCCAGAAAAGTTTTAATACTAAGAAAAACCCCCACTATTGCCTTACGCTTAGCAAAAG

TTAAGCCGATCCTGAAAAAATTGATTTTCGTATTTCCCCACCTGCTACGCAAGCGGGAGAATAGATCATAGTAAAACAAA

CCATAAATGCTCCCCAGTTAGCCGGAGGGTTACCCCCGATTACCTGGGGAGCCTGATAAGCACCTCCTAGTCTTGTAGAG

TCATTTCTCTCTACCGTATAAATATAAATGGTTTATACACACCACTTCTACGTGATATTAGCTTAGTAAAAGCTATACAT

ACTCCCAAGTTGCTGGTTCAGCCGTTAGTAGGGTAGGAACCCAGTAAAGTGATCCCTCCGAACCGTACGTGCACCTTTCA

ATGCATACGGCTCTCCCCGATTCTCCTGCGCGAAGAGGTGCGGATATTAATTTAAATACAATGTTCCGAGAGTTTATCTC

CAGAACTTATTAGCATGTACCAACGTTATTAATATCTAGTACCCTTAACGGGTAAGACAGGATAAGTTTGCTGTGAGAAA

GTGATTCATGGTAGATAGATGAATAAAAAATATTATTCCATGCTGCAATAAATTTATCTCTAATATGTGAATCTGGCTGA

CTCCTTCCTGGCCAGGTTCCAGACTGACAGTACTATGGAGTCTCCGACCTCTTGACATTTGGTTATTGATGTTATAACCT

GTATTTCTATTATTGTTTATTTGTAATGAATTAATTTGGGTTTTATTTTGTTTTGCCTTTATTTTCTATATCTTAACTAT

TTATGTGAAACGTAATTAAAGGTGTTTCAGAGAGAGACCTCTGTATTTACCCGAATGTACTAAATTATGACATGTTCTGC

ACAAGGGAACTTGTTTACGATTAATTTTGCGAGATTTTTATCGAACGGATTAAGTTTTAAGTTAATTGTCCTGATATGTT

TAACATGGTGCATTTCAATATTTGCCTCAGAGCCACAGTTAGCGCAGGATTTTTCTAGAGGATTGATAGCATATATCTTT

CATCTGACGTATTCGAGAGGATCCGTGAATTTCGACCCCTAAGAACAGCATAGGAGTACGCTTTAGCTCTGGGAGTACAA

AACTCGTGTATTTAGTTTTTTCCTTTTGAGTACGTTTTGATGAGGATATTTTTCCGAATCTCCTATCAAATACTGCCTCG

TTTATCTGTAGTTTTCGACATATGGTTTTCTTAGGCTACGCTTCAGAATCCAATGAGCTTTAATTAAGAGAGGTTTGTTA

TCGGCAAAGCTATAATAATTTAGGAAACCCCGTTGAATGTTGCGTTGTACCTGAGTATTATATCTCTTGTAGGAAGCAAT

ACGAGTCTTTTATGCCTTGAGGGTTTCAGCGGGAGCCGGTGATTTTACAAAACCGTTCTTTTCGAGTCTCTTCACAATTT

CCAAGACTGGTGCTGACATCCAAAGATTACCGGTTGGTACTCTCCGGTTTCCCCTATCACCTTTCATCATATAGGCTGCA

CCCGTGTTTGAGACTAATCTCTTGATGTACACCCCAAGGAATTTAGCTCGACTGGATCTGGCATTAGTAATTAGCGTCTT

CTCCATAGATAATTCTAGGAAGATGAGAGATAGGAACTTTTGTATCCTATCCTTCAGGTCCGATGCGTATTTTCTGCTTC

CTCAGACCCCTATTAATCGTACCAGTCGTCCGCGAATCTAACGTATCTAATTTGAGGTGAGCTTTTGGGTTAGGAACTAA

CGACTTTAGTCTCTTTCTTTCTTTAATATTTGTAATATATTTTTGTGTAGTTCCCCGTGTAAGCAGGGAGCAGTCTACCA

CCTTTGGCTTTTATAATTTTTAGCCTACCTTTGATCCTAGCTATTCTCATAGTCAATTTGTTATACTCAGTATTGGCAAT

ATAAGGTTTAACACCTGGGTTCTTAGGCTTCATTATTCGTGAGAATTTTTCTATATATTTATCAAGTTCATGCAGGACAA

GATTCGCCGATAAGATAGGGCTTATGATACTACCTTGTGGTACTCGAATTCAGTTGCAACAAATTGTCACTTTTCGTGTC

TCATTCTACATATCCTGGCTTTGACTAGTTTTCAGTATAAATGAGTGAACCCCTCTTTAAAGTGCTTTAAAGAAGATTGG

AAAGTAATTGATGGTCTATATTATCGAAAAACCTTTAATGTCACCTTCTATAAATCATGGTACCCCTTTTCAGTCTCTAA

TACTTCTCAGAGCCGTATGGCATCCCCTTCTCGGTCTGAAACCATGTGACAGGCTAGAGAACTTGGGTTCAAGAATGGTT

TCAAGTACAAATAGGAGAGATTGCTGAACAATCTTATCTCGCGGTGAACTTATACCTAGAGGTCTAAGTTTCCCGTTCGC

TTTGGGTATAAATACCCTTCTAGCTGGATTGGGTTTGTATGATTCATTTAATAGTTTCTCCGATGTGTTATTTATCCAGT

ACCAATTAAGTCCGTCTAGGGTCTCTTCGTTGGAGCCTGGGACCATATTACCTGGTTTACTCTTGATGACGTTGTATGCT

TGGATTAGCATTTCCGGATCACAAAGGATTTTAAATGCATTCTGACATCTCCCGTCGTCGTCGAATTCCAGTTTTTCCGA

ATTAATGCTTGTAAGTCCTGTGTTTCTCTCGGGCGTTAAGTTTTATTTTGTTATTCGTCATTATTATGTTTCTAGCTTGT

TAAATTATATTATATTGAGTACTAATGAGGGAGTAAAAACGATAGGTAACTTCTATACAAGATATTCCCAATTGAGATCC

TACCGTACTGTCTAAGTTTAAATTTAGATGAGTATGAGCCCCCATGATCTGTTTATATAAGGTGCATTCGTAACGACTCG

AAGGCCTTTTATCTCCGAGTCTGTCCCTACCTCTCCAGGTCCCCCGATCGATCCGAGTTTAAATGGTTACCTTTATGCCC

ATTGTGCGGATCCTGGGGTGGAGTGTATCTTGTGAATACTTAAAATTAGGTTCAGTAGGCTTTATTGGAGAATATCTGTG

TATAGTCATATCTATGCCTTTTGCATATGCTCAAAGGACTCCCGAATCTTAGGTTGTTGAGGTAATTAACTCTCGGAGCA

GCTAGAGAAGGGCTAGGCCTCAACTATCTGTTGAATTCATGAAATTCCGCCGCCGAATTATTTACTTCACCTAGAGATCG

TTAGTATTTAGGGCGATATAGTTAGGATATATAAAATTTGTGGCCAAAGTAAAATACAAAGGATCTCCGATCATCGCTGG

AAAAATGAATAATAGTGTACATAGGGATTATGATGATCCATATGTTGTCAAGATATCCCGAATTCTAATATATACGTCCA

TGAGATTGTTAGGTTTTTACTTACTAAGATGTTTGTCCTGCTTCGCGCAGACGGGCAGTATTTATTTCCTTATTTGATCC

ATAGCCTGATGATCAATTAAAATACCAGTGTCGTGTAAATGTATCTTTATACACTCTGGTGGTCCTCCAGACCCTCATCC

TCTTCTTTAAACCATAAACCTCCTAGCTCGCGGACAGGTTGTCGTTGCTTCAGTTCCTACATTCTGGAATCCTGCCTTTC

CGCTTTTCATACATGCTACACTCCCAGTCGCCTGGTTTGCAAGGCCTATGGCGACACCCATTTAAGCCGAGGATAAGTAT

GATGCTTTACAGATATTGGAAATAATCTGGATTCGAAGCGGGTCGAATCAAGCGTATATATTATTAGACGGCGCACCGGC

TATTGACCAATATTCCCCACTGCTGTACTATACGTATTGGGGTTTTTCAGACCCAATGTGATCGATCAACCTCTCAGTTA

CGATTACGGAATTATAAGCTAGTTAAACCATTACTTTAACTACTACCTATCCGAAATATTAATTGTCCTCTTAAAGCGGA

AAAAATCACCACCCACCTTTTATCCCTATCCCTTGCCTAAGCTTAGGGATAAGGTGTGAGTGGAATATTCCTTGCAATTT

CCTTTTTTTAAAGATCTTTGACAAGCATGAGATCCCTGGGATAAGGCATTTATTAACCTCACTTTAAGGTAGCCAAAATA

TCATTTACTCACCTGTACACCACTTCTAATAAAAAATCCTCTCCTCTAAAAGATATCAAAAATATATTAGTGTAAATTAT

ATAATTTATATAATATTCTTTTCCTAGCTCCCCGTCTTTGGAGGGAGGCCACGCAGGTGAGGGAATATTTAAATTAGACG

TTCGATTAGCATGTGTCAAGCACTTGAGATAGCGTTCATTCAGAGCCATCATCAAACTCAATTAGTTGTTGTAGATATTC

CCCATAAATTTAATTGGAGTGTTTTCTACAACACTACTAATAAATATTAACCGAATATTTAAGGCTAAATTAAAACCCAT

CCCTCCCCTCAGGAGCCGTCTAATCCCCTTTTTGGTTAGTCCGCTGGGAGGCCGCCCAACTTGTTCTCCCTATCCCCCGC

AGGGATTAGGGAGGGAGGGAGGGATTCTCCAAGGGATAAGTCCCTCCGGCTCAGGAACTAGTTTGCAGGAAACAAACTTG

TTTGTTTTCAAAACAAGTTTTGAAAAACTAGTTTTGTGGGCCACGAACCCCCAATCGATAGTGTTTGTCCCTAAAAGAAG

ACAAGTTCGTTGAGGAACGAAAACCCCCTAGTTTTCGTTCCTCCCCTGCACTTGGTGAGGGACGCACCGCGCAGGCAGTG

CACCGTCCCACAAAACTAGTTACCGCCGGAGGGGAATAATCCCTCCCCGATTCCCCGATCGATTGGGACAGGAATGGAAT

CCCCTGGCGGATTCCCCGAATCTAATTTATTAATCTATAAAACCCATGGAATTTTTAGCTTGCTAGCCTCCCCTAACAAA

GAGGCCAGCCAACCCTAACCCTAAAGGTGATCCCCAGGAGGTTATCCCCTAGCCTCCCCTAGCTAGGAGCCCAGATAGCG

GTGTCCTCCTATAGAGGGGTAGCAAAGAGGGATTACCCCTAGCGAGTTTCCACCCTTTGGCTTACTAATCCCCGCCTTAC

CGCCCCTGCTGCTGCAGGGGATAAGCAGCAAGTAAGAAAACCGGATTTTCCCCTATTTTGTGGGACAAAATGGTTTGGGT

CCCTCTCATCCCTCTCCGAAGGAGAGGATGCCGCCGCCAAACCCCAATCCGATCGATCCCCGATCGATCCAAAGGGGTTT

GAAGAACAAGTTCCGTTGAGGAACGAAAACCTAGTTTTGTGGGACAAAATTGTTTTGTCCCTCTGCATCCCTCTCCGAAG

GAGAGAACAGCACAGGATGCAAACCCCCGATCCTTTGGGGTTTGAACAAGTTCGTTGAGGAACGCAAAACTAGTTCCCTC

TCCAGGGATCTACTCCAATCCGATGTCCCTCCCCTTTCTTTTCCCGAGGGAAAAGAACAACCAGAACCAGAACTGGGATT

TTAATCCCGGAGGGATTCCTTTCCTAAAAGGAATCCATACCTTAGGATTACCGCGAAGGGAATAAAAGTCGCCTTCGGGT

TTGGAATTAAAAGAAAGGGGAGAGGGTTTAAAAACCTCTGCCCCTGATCGGGAGCCCGCAGGGTTTTTACAGGAAAACCC

TCTGCCGCCCTTCATCGGGGAGCCCTGAGGGTTTAAAACACAGGAAGAAACTCCCCTTCGGCGGCCCAGAGGGTTTAAAT

ACACAGGAAGAAACCCTCTCCCTTCGGGGAGTTTTTCCTGTGAATTTTGCGGAGAAAAATTAAAAACCCCTTAATCGGGG

AGCCCCGAGGGTTTTTAAACCCTCGCCGGCCCCGATCGAAGGCGGAGCCCCTCGGGGAGAAATAACTTGATTTGGAATCC

CGGAATTCCTAAAAAGGGCGAAAGGCAATGGAATATTCACCTTACGGATTACCGCGAAGGTAATAAAAGTCTTTGGAAAT

TGGGGCTTTGCAGCAAAAATATCCAAATAAAAAGCAAAAAATTTAGGGTTTTTTACAGGATTTTAATAAAATTTGGGAAT

TAAAAGAAAAGGGGATTTGGAGAGGGAACAAGTTGCGCCTCCGGCGATCATAGGGGACTAGTTGGGATGGAGCTATACAA

GATAAAGTCTATCCCCTACTAAAAATACGTATTTGGTTGGATATAATTAAGCCGTGTTCTGTGATAATTATTATTCAATA

TTAAGGGCTAACAATAAGATTGTATTTTCATTAAAAATTTTATACTGTATATACCCTACCCCCGCTAAGGGAATGAATGA

ATCCCTGGCGGGATTCCCCGGAATAATAAAAGACATATATACATTTAGCTATAGTTCTATACTAGTTCCCCGGATCTCCA

CCGATTAATGAGGGTACTCCAATCGATCGATCGATCCGATCGATCCGATCGATCGATGATTATCCCCGGATCCCCGATCG

CCAGAGGCGATGGTTTCCTTCCCCTCGGGGAAAGACGACTGACGATTTTTAATAAAAGTCTTTTGAATTAAAAGAAAAGG

GGAGGAATAAAAATTCCAAAACCCCTTCGGGGAGTTTTTAATAAAAATTAAAAACCCCTTCGCCCGCCCTTCGGGAGTTT

TTCCTGTCAATTTTGGCGGAGAAAATTAAAACCCCTTCGATCATCGGGGAGGAGGGTTTAAAACCCTCTGCCGCCCTTCC

GGAGCCCGCGGGGTTTTTACAGGAAAAACCCTCTGCCGCCCTTCCGGAGCCCTGAGGTTTTTACAGGAAAAACCCTCTGC

CGCCCTTCAATCATCGGGGAGGAGGGTTTAAAACCCTCTGGCCGCCTTCAGCGATCGGGGAGCCTGAGGGTTTAAAACCC

CTCTGGCCGCCCTTCAGCGATCGGGGAGCCTGAGGGTTTAAAACCCCCTCTGGCCGCCCTTCAGCGATCGGGGAGCCTGA

GGGTTTAAAACCCCTCTGGCCGCCCTTCAGCGATCGGGGAGCCTGAGGGTTTAATACACAGGAGGAAGAAACCCTCTCCC

CTTCGATCATCGATCGGGAGTTTGGGTATTTTGGCAGAGAAAATTAAAAACCCCTTAATCGGGGAGCCCCGAGGGTTTTT

AAACCCTCTGCCGGGCCTTCCGGGAGTTTTTCCTGTGAATTTTGGCGGAGAAAATTAAAACCCCTTCGGCCATCGGGGAG

CCCGGAGGGTTTTACAGGAAAAAACCCTCCGCCGCCCCGAAGGCGGGGAGAGGAACAAGTTGGCCTAGGGACTAGTTTTG

TGGGACCCCCAACCCACGCCACGCCAATTGCTGCCCGTCACTCAAAGTGCTGGGCGAGGAACGAAAACTATAAAATAAAA

TTTACCTCATTTACGGACGGTTATTTTCTGTTTAATACTAAAGTTTTTACTTAACACTCTTTCCTAGGTGTTTATGCTTA

ATTAGCGACGGACTTTCCTATACTATGTTTATGGGATAGTATTTAGATTATTTATATCACAACCTCTTGTCCCAATCCCC

AATCAATCGCCGGGATTTGAGGCGAACTAAGGTTGCTCGAGCAACTAACTAGTTTTGTGGGACCCCGCTGCCCGCGCTGC

TGGCTCGGCTGCTCGCTGCTCGCTGCTCGCTGCTCGCTGCTGCTCGCTGCTCGCTGCTCGCTGCTCGCTGCTCGCTGCTC

GCTGCTCGCTGCTCGCTGCTCGCTGCTGCTGCTGCTGCGCTGCTCGCTGCTGCTAAGGGGATGCTAAGGGGATAAGCCTG

CAGGGATCGCCGCTGGGATGCTAAGGCGGGGATCCCCGAGGACTGGAAAGGAAAAAAGGGTCTACAAATATAAAGAACAA

TTATGTAAGTAAATTAAATAATTAGAGTTTGAGTAATTTTTATATAAAGTAAACATTTGTCTACTATCTATTTTAAGTGC

ATTTTTCCTAATTCAAATGCAAAACCTAAAGTTAAGGCTAAAAAATATTAACATAATAACCAAACCATATATATTATTTA

CATATGCACTAACAACATAAGGATAAACTAATAGAATTTCTAAATCAAATAATAAAACAAAAGTGCAAAAATAAAAAAGA

AATACTAAATTGTGTTCTGTTTTGACCTAAAAAGAGTGAAAACCACATTCAAATACGCTATCTTTTTCAAGTGAAGGGTT

ATGTGGAGCGAATATAAAATTAACCATTAATAAAATAATAGCTAGTAAAGGTATAAAACCAAAAAAGTAGTGGTAGTCAT

ATTTTTGGAAGGATAATCAGATAATATGTTTACCATATATAACAAATTAATGTAAATCTAAAGCATCCTTTATATAGCTA

CTTGTTAAAACAACAAAACTTGAGCTTGTATAAAGGCAATACCTAATTCTAAACCCGAAAATGCAATAATAAAACATAAT

GGAACCAACCCCAAAAAGAAATATATAAACCCTGATGTCATAATATTATAAGTAAAACCTGCTAAAATATTTAATAGCAT

GTGACCAGATAATCGCCACACAATTTCATCCTTTTTTTATTTTCTAAGAGGTGCTGCTAAGCCGTCAATCTATTTCTTAT

GTAAGAATATTAAGCTCATCTGAATAAATATAGACCTTTAAAAGGTTGGCGGGAGTTCTATTTTCTTTTAAGTATAAAGA

TATACTAGGTTGACGCACTTTTACTTATGCTAGCCCCTCCTGGGCTTACTCCCTCCCTCTAAGAGGGAGGGAGGGAGGGT

GCTAGATAACTGAAATGAATTTAAAAGGTATATACCTCTGAATGGAGTAGTACGTTTCCTACTTAAATAAGTAGAAATAC

TAGCCGAAGATATACCTAAAGCTTCACTTGCTGAACTAGTCCCTCTCCGAGGGACTTATCCCGTATCCCGCCGCGCCTAT

CGGCCGCCGGGATCCGGACTAGTTTGCAGGAAACAAACTTGTTTGTTTTCAAAACCAGAAGTTTTAAAAACTCGTTCCGG

AGGGCCCCAGGAACTGGTCCCGAGGGAACAAGTCCCCGATTGGAACTCCAATCGATGTTTGCAGGAAACAAACTTGTTTG

TTTTCAAAACCAGAAGTTTTGAAAAAACTAGTTCCTGAGGGCCCCCAGGAACTGGTCCCGAGGGAACAAGTCCCCGATTG

GAACTCCAATCGATGTTTGCAGGAAACAAACTTGTTTGTTTTCAAAACCAGAAGTTTTGAAAAACTAGTTTTCGTTCCTC

TGCATCCCTCTCCGAAGGAGAGAACAGCACTGGATGCCAAACCCCCAAAGTTTTAACTCCTGTTGTTTTGAGGGGCCAAA

ACAACAGGAGAGGAGTTTTCCCCAACTAGTCCCCGATTGCAATTGGCGTAACTTGTTCCTCTCCCCTTTTTTTCCCCCTC

GAAATAACTTTTATTTTAATCCCCGAGGGATTCCTTTAGGAATCCCTCAGGGATTCCCGAAGGGAATAAAAGTCAGTCTT

TTAATTAAAAAGCGACTTCTATGAAAAATCCCCAAGTCCTCCGATTTCCCGAGGGGAAAAGAAAAACCCTCCCCTTCCCT

TCCCCGAAGGGAAGGAGGGTTTTCTATTTGAGATTTTGCTGCAAAGCAGCAAAAATTCCCCAATTTGATTCCCTTGCGGC

GGAATCCCCTAAGGATTCCCTAAAAGGAATGGATTAAAATAAAAGTCCTTTTCCCGAGGGGAAAGAAAAACCCTTCCCTT

CCCCGAAGGAAGGGAAGGAAGTTTTGCGAAAAGGTTTGAATTTTTGCTGCAAAGAAAAGATATTTTCAAATAAATAATAA

TTTATTTGAAAAATTAAAAAAAAAAATTATTTTGCTTTGCTTTCTTTTATATTTTGATTTTTGCAAAGAATAAAAATTAA

AACCCCCAAATCATCGGGGAGCTTTGGGTTTTATATTTTGACAAAAAGAAAAAAAGGCTTTTTTATTTTTTTTTTGAGGG

ACAACAACTAAAAAAAGTTAAGTTACCGTTGTCCCCCAAAACTAGTTCCCCGAGTTGGGCCAGGAACTGGTCCCGAGGGA

AATTGGAAGTCCCCCGACTAGTCCCTTCCCAGCCCCTGGGCTGGGAGGAGCTGACCCTCTTGCGGACGAAGTAGAGGGGT

AGAAGCGCACATTAATGTAATAATGTGATAAAAATATAATTAATCATATAGCTAACGAGGCGTGCTCCCGGGTGAAACCT

CCTGCCGGACCGCCAGAGTTTCTGCGCAAGCTAGCCCCCTCTGGGCTTACACCTCCTCCTCCGGAGGAGGGTGCTACATT

AGAAAATATTCTAAGGATAGGCTATTGTGATATCTTTAATCTCAGATTACTTAATTTATTCTGGGGTGTAAGCCCCACTT

GCACCCTCCCTCGAAGGGAAGAACCGGCTTTCCCGGCGACTAGAGTACACCTTACAGTACGGCTACGCCTAAATACTGAA

GAACCATCTACTCGTTGCTCTTTTACAACTATCCCTTGCGGGATCCCCTATAAGGATCCCCAATTAAAGGATCCCCGAAG

GGATAGGTATATAGCATATAATTGACTTAGATCCGCGATCACCCATTCCTATTACTATAATCTTTAATGATGTTACTATA

CCCTCAGTAATTAGCTGGGCCGGATAAGAAGTTTCCATCTTATCTTTAGTTATTAAAGCTTTGAGGCTTCCCGAATTTGG

CTCTTAAACACATTAAGTGAATTACCTATTTTCACTTTTATATGTTGGCTGCCAGTCTTAAACCTAATGAAATATTTCTT

GCTAAGTATGAACGTCTGTACTCTTGACTTATTTTAATTATAATTTTTAATCCTAAACATTTTCTCTTCTGTTTTCGGGA

AACTAAATATGATTATAAGGTTAAACTAATTTAAAAATATACTTTTTCTGAAAGGACTACTACGCTTCTTGGCTATATAC

AATGACAAGCTTCCCTGAGATATACCTAAAGCTTTCCCAGCTAAGCTAATAGAAGGATATGTATTTATTTCATTAGTATC

TAAATCTGTAACCTCTATCTTTTTGTCGTTTTATTTTCTACATTTTCAGTATAAGATAATTTCGATATAGTGTATCCTTT

TAAAGTTTCATTTCCTGTTTTGACAGTTTTATTAAAGAAATATCACAAACGTCCATTAGATATGCCCAAATATTTAGCTG

CATCTACCATATTTCATATCCCCTTTAGGGGAGGGACAAGAAAATTCTTTTTCTCCTGTTTCTTTGTTAGTAAGTAAAAC

ACAGGAGGCTGGTGCGAGATTTTACCTGTAAAACTATTAGACCCATTTGATTCATCGAGAATTTCCTTATTACATTCCCT

CCACCCCCGATGATCGATGGGGGTGGAGGATCTGGTAAAGAGAGAGGACCTTTTGCTGAAATTGTATATTTATTGTAAGT

TAAATTATTTAATAAATATTTACTAGTTCCCTCTACGATCGGGGACCTTGTCCCCAATTGAGAGCGGAACAAGTTCCGCC

CGCGGGACTAACCGAAATCCGAGATATTCCCAAATAATTCCCTGCATCTGTAAGAGAAGCCAAATCTAGTACCTCACCAG

TCTCTGTATTAGTGATTTGAACAGGTTTTCTAAATGTATTGCTTATACGCATTTTTCTATACGCTCCTTATCAAAGACTT

TACCTAATAAAGCTTCTCTTTTTAGTTCACGGGTAGATTCGGATACTTTAATATTTTTAAAGCTAAACTTATTAGTTTTT

AGACGCTTCACTGTGTCTGTAACCTAGAGGAGATCCTGCTATTTTTAATATATTATATTCAGGGATTAGGGTATCAAAAT

AAAATTGTTCCCTTTCAATTAATAATTCCGGAGAGCAATATTCTAAAATCTCTAACCTAAACTCCGCATAACCATACTTA

AGCAACGCCTTATAAATCCTCATATTACGTTTAGGATATGTAATATGGTTATAATTAAAATATTGTTTAAATCTAATACT

AAGGTTTTCGCTGACCCGACATAAGATTTACCCTGCTCTATATGCACCCACCTGTAAATACCTGTTCGTCCTTTATTTTC

ATTCACAATTGATTCTTTTTCCTTATCAGGATTAAGATAAACTTGAACAGGTACAGGTTTAGATGAGATAGACATAGTTC

TTTGACCATTAGAGGCAGAAATCATTCGGGAAACATAAGGACGAGCTAATAAACCTATCCCTACCCGGATCCCTGAAGGG

TAGCTTGCGCACCGGATAGGATTTTGCAGGGTATAGTGTTATGCTTAGTTCCCACAGTAAAATTATTATTAGGTTTCAAT

ATTAAACTACGAAATATAAGTATAATAAATAGGCCACTTAATACTCGAAAATTACGAATATGTATTAAATACATACCGAC

AAATTTAATTTATAATTAATCAAGACTATACATGAACTTTAATTTGAGTTTACTTGATTCTACTCAAAACCCCTTTAATG

GCGACTAGAGTACACCTTACAGTATTAAAACTATACTGAAGAACCGTCTACTCGTTGCTCTTTTACAAACTTTTATAGTT

TGATTTAGATCCGCGATTACCTACTTTTGAAAAAAATTTTCTCTATCTCTAGTGATATTACCATACCTACTTCCCCTCCC

GGGATATAATGATTAGTTAAGCCAGTTATATCTTTACAATATAACCTTGGTTACTAGAGCTTTAAGGTGTCCCCGGAGTT

TGGCTCTGGTTCACATAGTAGCCTAATCTACTGTATGAATTCTATTAGGACAAGTAAAGGTAGGGTCAGCCTTGTAAGAA

CTGCCCTCAGAACCGTACGTGATAGTTTCCCATCATACGGCTCGCCACATAACAACTGGTATCCAGATACTCGACGGCTA

GTTGAAAGAGAATATTACAGAAATGGTTATGTTAGCTATTTCATTGAACTTGCAATATATACGTGTTGCTTGAAGTCTGG

TCTGGGTTTACCCATACCTTTCTACTTAAAGGAGAGGTGCTTTACCCATAACTTTAGCTGCAGCCTTTTCTCCAACTGAA

AGGTGATGAAGGTACCCCACATTGCAAGATCTTAATCACCTCCTGAGGACCCTCTATAACTCGCCAATTTCCTTAGTTCG

TACGCAGTAAGAACTCCCTTGGGATATTTTGGCTTGTGATGATATTAAAGAGGAATTGGTTTACGTTGGAGGGCAGCTCC

GAATTGAGCGTAAGAGACCTTTTCCTCCTTACGATATTTAGCTCTGACATTTGCAACTGACCTTCTGTAATGATGCATTT

CTAAGTTAGAGGTTGAACCACAAAGAGTGCAAGACAATCCGAAGCTAGATTTAGTAAGCTTTCCGCTTAAGACTGTCCGG

ATGTTTTCTTCCATGTCTGCGACAGAACCTCCCCTTGAACTCGTGGTTTGCAGATTAGGCTTGTGTAGGCAAATTCCAGT

GTCTGGACATTCAAGTTCATTACCAAACCTGCAGAAAACTTTCTTCATAGTTCCGAGTTTGAGTTTACGGGCTAATGTAA

GAGCACAAGAAGCTTTAAACATCCAGATAATAAAATGTAAACTCTTACGATTTGAAGCAAAGCTATAATAGTTTGACGTT

CCATGTATCTTGGAGTTGTAAAATCTTATAATCTCAGAATGACTTAGGTTTACCATACTGGTAAGACATTGGGGTATCAC

TTTTGACCTCGGTTATGACGTACTATCCCTACATCTTTCAAGGCCTTGATCAATTTAGAGATGGGTGCGTTAACCTGTAT

CATAGGAGTTCCCACCATTTGGCGCTTACCATGATTAACTAGTCAGCTGTTATTAGTCCTTATCTTAGATATCTCCGCTC

CAAGAAAACTCCACTTTTCAGTTGCAATGTTCGATATGACGGTCTTATCTGAATTAAGTCCTACTCCACAATTCGCCTTT

AGAAAATCTTTAACGTTGTTCTTTATGTATCTAGCGTCCATAAGACTTCCGGTTACTAGTATAATAAAATAATCAGCATA

TCTTACGTAGTCCATTCTCTTAAAACCTGGATCAAACATATCCTGACTTCTCATGGTACGCATCTGTTTAAGAAGCTCAC

GTCTCATTTCTGGAGATTTAGACCTTTCCCTCTTTCCCAAGAAACTTTTGTAGGTTGGGTTTCATCTACGCTTTCTACCT

TTAGTGAAGCTGACTTTATATTTGGCGAGATATTTGTCCAGCTCGTGCAGAACAATGTTACTAAGTAACGGGCTAAGGAT

CCCCTGAGGTGTACCTGAGTAAGAATTCACTAGTTTTCCAGTAGTAGGATCAATGAAACCTGCATTAAGAAACTTTCTTA

TGATGCTAAGGAAAGCCGGATCTCCGATTTGAGCGCGAATTCTTTTCATGATGATAGAGTGAGGAATAGAGTAAAACACT

TGGTAATGTCCCCTTGGATAACCCAATTATGCTTATTTCCAATCAAATATGTTATGAACAGAGCCGAGTGAGTTGATTTA

TTTGGTCTGAAACCATGGGATGTATCCAGGAATTGGGGTTCAAAAATTGCCTCCAAGACGGAATGCAGGGCTTTCTGCAC

GATTTTTCTCTCGGGGATCCAATCCCGTTGGGCCGAGTTCTTCCGTTGGCTTTCGGTATCTCGACTCGTCGATTGGGTTT

GAAATCAAACTTACCACTTTTAAGATCCATGGCCACCTTCTCAAACCATTTGTAATCAATCCCGTCCAGAGTGACTCTAT

CAACACCTCGAGTCATATTCCCTTGACTTTTAATTTCATCATAACAGGCTGCAAGGAAGTTAGGCTCCGCAATAACCTGA

AGAATTTTGTAATATTTCTGACCAGCCACTTTGTTCTGTTCCAAAGTTTTCTCACCATAGTAGTAACACCGACTCGCTTA

ACCACCTTGGAAGGTTTGACCTGGTTTGCCGGCTTTTCTAGGTTATTATCTAAGGTTTCTACTTCTGGAAGTTATGCTGT

TTCCCTTCGCGCAAGAGAATGCACTACTATGGAAACTCCGTCTCCGCATATTAGAAATAGAGGCGGTTAGATCCCGGGTT

GCGCTGAAGTTACGTTTATTGTCCTAGTAGTTTTAGCCGCCTGCTTATACCCGTTTAGGCTTTCTGTAGGAAAGAGCGTA

CAACCCTATGACTACTAATGAAACTATTGCCATTTCCATTAGCTATGTTGTAAAGATACCGATATATTCTGCAGATATCT

CCCGCAGCTGTTAGGCACTTATAAGTGTCAGGTTGGTTAGCCTCGACATAACTACTCCCGCTCAAACATTCTTCTTTATG

GGGCAGTCCACATAAGAATGTTCGTTTGTATTCAGCTTTCCATTCCAAACGGATTATTAGCCCGATTAGTCGTGAATACC

CGCGGCATGTATCACGCACGTGGTCGTGGTCATATATACACGAGAGCCCGGACTTGGCCTCAAGAAACTTCAGCACCTCG

CACGGCGCACGCAATAAAGCTAATGGACAACCTGCAGGAACTAATAAAGAAAAGAATTCTAAACCGTGTTTTTAAATCCT

ACAATTGTTGCACCTAGCACTACGGTAAAGCTAAGAGAAAATGTAAAAATAAAATGACTTGTTGAAGCAAAACTATATGG

AACCAATCCAACTAAATTATTTACTAATATAAATATAAATAAAGTATATACAAATGGGAAATATATTTGCCCATTTTAGA

ATTTATTTGATTTATAACTATACTATGTATAGTTGCATATAATGTTTCCTGGCTTATAGATCAATTATTACTTATTACTT

TATTGTAATTTTTAGCTAAAAGCTTAACCATTAAAACCAAAAAGCAGATATTGATAGGGTGGAATCGAACATTTGAGATT

ATCTCCTGCCTAAGCCCCTCCAAACCCAGCGAGATAGTTACCCATCACTAGGCTTTCCAACCGCTACATTTAATTCTATG

ACCCTACATCTCCATATCATTTAGGAATTTGTCTATTGATATAGGTGTATTTTTCAATTATTATTATGAATCTGGAAGTG

GTTTCTACATAAAGGAATTGCTGCTTTCTCATGATAGCTCTGTTGGTCTTTGATACTATCTTTCAATGGCTTTTCTGGTT

TTCCAAGGCTTTTAACATGATGCATTTCTACACCTACGTCTGTACCACAGACTATACAGCATTCTTATAGGACTCTTATA

CGGTTCATTCTTCACCCCATTAGTGATAATGGGTTTGAATCTATCGATTTAAGTTCATCGTTTTTCATCATTGTGACGAT

CTTATGAAAACTTTATGGATTGTTCAGTGATTTAACGAATTCTGGTTGCCAGTCTTTAGTAAGTTGGTTTTAATAGGTCT

AGGTATGTCCTTGTACTTATCATAAAGTATTGGTTCAACTATTCTTTTACTCTTCTTCCTTGGATTGTCATTCATATTAC

TTTGCTTAGCCCCTCCCTCGAAGAGAAAGGGTGTAAGGCAGAGGGCTTAGATTTGGCTTCAAGCCATTTCTCCACTCTTT

CGTCTGTAACCCCTATTACTGATTTCTTGTTAGTTGATAGTGGTTTATCCAACCCTATTCCTCCAACGGCGAAGACTTTG

GCTAGTGAACCTAGTTTAAATTTTGCTGCGTAGAATTTAGCAGCCGAGAATCTAAGTATGTAGCTAATTCTAGCTACTGC

TCTTCTTCTATTACCCGCTAGGCTCCATAAATGTGATATACCTCTTATAATGCTATTTATTATGCGATTTATCTCAGATT

GTGGTAGCATTAATAGTGTGAACTTAGTTCGAGCTTCTCCGGCTTTATTACAGAATCCGGTTTTAGTTAGACTTTCCACT

ATTTTCTGTACATTAGCGTCTAATGTAGGTATAGTCATTTTCCTATTAACTACTTTTCCTCTATATCCTTGCATTGTTAA

TATCGTTCTTCTACCGATCAAATATCCTAGGAAAGGCACCATTTTTGATATATGCGTTATATGAGTTTTCTCCATATTTA

AGGTTAATGATAGTCTTGTCTCCAAGAATGTTTTAATCTTATTTCTTATATCTACTGCCATATCTCTAGACCCTGTGATC

CCTATTAAGAAGTCATCAGCATATCTTACGTATTTTACACTTGTATATTCTTTATCGAATGGATACATTGCGGAGACTTT

TCTAGCCATTGTAGGGTTTAATCCTTTTCTTCTGTCCATGTATTTTAGATACTCCCTATTCGTTCGAGGTTTCTCTCTAG

TTCCTTGATACTCTTTCATGATCTCTTCCATAAATTGGTCCAGTTCATGTAGGTATATATTAGATAAAAGTGGAGATAAG

ATACCTCCCTGAGGAGTTCCACTTACGTGTTCAATTATCTTATTATTAAATATTATATCAGCTTTTAATGCCGATTCCAC

TAAATCCAATATTAGATTATATCGGACTTTCCTTCTCAAAAGGTTAATTAGTATGTTATGATTTATTGAATCCAAATAAC

TTTTATGTCCCCTTCTATGTATCAAGATCCTCCTTTGAATCTAGTGTGAACTTGTTTTAATGCTGTATGACAGCTTCTAC

CTGGCCTGAATCCGTGACTATTCCCATTAAATATAGGTTCGAATATAGCTTCTAGCATACTTCTAACCACCTCTTGTACT

ATTCTATCGTTTAGAGTAGGTATCCCTAGGGTCTTTTACCCTTTCCTTTCGGTATTTCCACTCTCTTCACAGGAGGTGTT

CACTCGAATTTTCTGGCCATGACTTGTTCTCTTAATTTATCTAGCTTTTCTTGCGTTGTTCCATCTAGTGTTTGTAGATC

TATTCCGGGAGTGTTACTTCCTTTACTGGTTCTCACTTTGATGTAGCTCGCATATCATAGCTCTTTAGTACTAAGCAGAG

TCTTTAAGTTCTCCTTGAAAATCTTTTCCTCCTCCTCCCTCTTCGAGGGAGGAGGAGGAGGGATTAGGGTTTAAGTAATT

GAAATGTCATAGTTTCGCCAATTTTTCAAACCCTAGATCGTTTGTATTCTGATCTTCTCTCATTTTAGATAGCAAGGAGT

TTGTATTTTGGTTCCTTGTAGTGTATGCCCGGTTTGACAATCATAATCGAGATTTAGATTCCTTCATGTTACCCATTACT

ATGAATCCTCTGACTTCCATAGAGCAGCTGTGTCTTAATAGACCGCCTGATTTATGGATCTCTCCGATACCTTTGCTAGC

AGCTGGGATTAAGGATCCCTGGTTTATTATGGTTGCGCTTTTGCATTCTTGTCCCCAATGCGGTGGGTAATTATGAAATA

ATAGTTTTACTTCTTCACATTTAACCGATGATAAGCTATTTCATGACAGTCGTGAGTTCGACACTTTATAAACTGCGGTG

GTTCTGCACCAAATGAATCTCGCGTTTCACAATTCAGTGTCAACCATATTAAACGCTTGAGTTAGAGTACTTTCTTGGGA

TATCTGAACCAGATAGTCGAAGACTCCCTCACTTACCCATTTCATGTAACAGGCTATTGTCCCTTTACCTTGCGGCTTGT

TAGGTTGTTAACCCCAACAGGGTTAGTTACACACTCTTCATGGTTACTACTACCATGGATCTTTACGATCAGTTCTAGCA

AAAGTCGAGCGGACGCCCTTAAATATAAACCTATGTTTGTTAATGATAGATGTAAATTTCCAAACAAGGGTGCATCCAAA

CTTATAAAATTTCTTATTTCAAACTGATCTAAAGGACTAGTAATTACAAAGGAATTTATATACATAACTCCTAAGATTAC

ACTTAATTCCTGTAATTAATATTTTGCCTTTTACCCTCGAGACTCCGTCTCGGAGGAATTTAGGCCTGTGGCCTCTTTTT

AGAGCGGAACCCTGCAGGTAATCCTCCCCTTGCGGCCTTATCCCCTACGGCTGCCGTAGGATTAGGTGGTAGCAAGGCTT

GCACCCTCTAAGAAATAGAAAGAGGGAGCCCCTAATCCGCGGCGATTTTTTCTCCCTCAGCAGCCTAAGCAGGAGTGTAT

TCTTAGCCCTCCCCAGGTTTTCCTCAGCCAAGCGGTATCCCTTATCCCCCTACGGCTCCCTAGGGATAAGCCTCCCCGAT

CCCCAATCAATCAATAGCAATAGCTGCAATCGCCGGGATTAGGCTAAAGAGGGGTAGCAAAACCTGAGGGATCCTCTAAG

GCATAGGAGGACCCCCTTAAATACTCCTTCGTCTATGGCCCTTATCCCCTAATCGATCGGAGGACCTCTTTGCTACCCCC

TAAGGGATACCCCAATCCAATTGCTGTCATAGAGGGATAGCTGGATGCTAAGGGATACCCCTCGAAAAGAAAGCAGAGCT

TCTTCTTCGTCCCCCTAATCCCTCTAATCCTACCCCCTTATCCCCCTAATCGCCGGATTAGTTGGGATACGCCCAGCCTG

CTGGGGATAACCTGATTAAAACCCCCAGGGACCGTACCCACCCCTTTTGGTTAGTCCCGCAGGAGGCGGAACTTCTGGTT

CCGCTCTCAATCGGGGATAAGATCCCTGAGAGGGAACCCTAGTTTTCGTTCCTCACGAAACCCCCGTTCAAACCCCCAAA

TGGGGTTGTTGGCATCCTTCCTCTCCGAAGGAGAGGAAGGGATGCTGCTGCAGAGGGACCAAAACAAGTTTGTCCTAAAA

CTAGTCCAATCGGGGTAACTTGTTACCTCGAGAGGACAAGAGGGGACCCTTCCCCTTCGGGGAAGGGATCCCCGAAGGGC

CCAGAGGGTTTTTACAGGAAAAAACCCTCTGGCTCCCCGAAGGCCCCGCCTGTTTTAATTTTGGCTCTGCCAAAATTCCA

AAACTCCCGAAGGGTTTTAATTTTATTAAAAACTCCCCCTTTTCCCCTCGGGGAAATAACGGAGCAAACTTTATTTTTAA

TCCCGGAGGGATTGATTCCTTTAGGAATCCCTCAGGGGATTACCGCGAAGGGAATAAAAGTATTTTAATTAAAAGCCCCC

GACTTCTATTAAAAATCCAGTAAAACCCCATCGATCCCCCGTTTTTGGCTTTTTGATTTGGAGATTTTGCAAAGCAAAAT

TCCCAAATTTGATTCCCTTGCGGAATCCCTAAGGATTCCCCTATTTCATTCTCCCCTTAGGGGAAAAAGGAATAGGAATG

GAATCCCTCCGGGGATTAAAATAAAAGTCCTTTTCCCGAGGGGAAAAGTCCCTTCCCTTCCCCGAAGGAAGGGAAGTTTT

ACGATTTGAGATTTTTGCAAAGCAAAATTCCAAAAGACTTTATTCCCTTGCGGAATCCCTAAGGATTCCCCTAAAAGGAA

TGGAATATGGAATCCCCGGAGGGATTAAAATAAAGTTATTTCTCCCCGAGGGGAGAAAAGAAAAGGGACGGAGATCCCCA

TCGATCGGGGAGAATCGGGGAACTTGTGGTTTTCGTCGTCGAGGTGGGCTCCCTCAACGAACTTGTTCTGGTTCAAACTT

TGGGGTTTGCATCGTCCCTCTCCGAAGGAGAGATGGACGACGCAGAGGGCCAAAACCACCCGTTTTCCCCAACTTGCTGG

CTTGTGAGTGGTTTTCGTGGCTGGCTCCCGGCTCCCTCAAAACCCAAATCACTTGTTCAAACTTTGTTTGCATCGTCCCC

TCTCCGAAGGAGAGATGGACGACGCAGAGGGCCAAACCAGTTTTCCCCAACTAGTTTTCGTTCCTAAACGAACTTTTTCC

CTACCGGGGAGAATCCCAAGATATTTTACAGAAAGTCCAAATTAAAAAGAAAAACCAATTCTGTTTTTCCTTTGAGATTT

TTTGTTCAAACCCCAAAGGTTGCATCCTTCCTCTCCAAAGGAGAGGAAGGGACGCAGAGGGACAACAAAGAAGAGTTAAT

TCCCGTTGTCCCCGCCGCCGCAAAACTAGTTCCCCGGCATAAGGCTGGACCGAACCCGGAAGAATAAAACCCTGAAGATA

TATTATAGCGATTTTTCTTTGCTTTAAGCTGGGATATCTATATCCTAAGAAGGGGATTAAATACCTAGGAAAAAAGATCC

CCTCTAAGCGAAAAAGCATTTAAAAGAAATATATTAGATATCCCCTTAGGGTAAACTACAACTTACTTATGAAAAGACGA

GAGAGAAATAAAATAACAAACCTAGGTAAAATATACTTAGATAAAACCCAAATCATAAATGGTAATAAAACAAATGCAAA

GGTAACTTGGTTAATAAAGTAAAAGGAACTAATTGTGGCATTTTTTTTTAGTTTTTAAATTATCTTACTGCACTAATACC

ATATAAGGTATAAATAATCGAGGTATATATGACACAATTACAAAAGAGTAACCCCTATTGACTAACACCAACAAACACCA

TATCTCCAAACGAGTAAAGCTCTCTGTAATCCCCGATTCCTCCTGGCTTATCCTACCCCCTAATCCCCCACCAATTCTTA

AGAGGACAGGAGAGGAGCCTAATCCCCCAATCAATCTACGGGGAGCCTTGATAAGCGGCCCGTAGGGATAACTACGATTA

CCCCCGATTAGGGTAGGGATACCTCCCTCCCCCGAGTTTCCCTCTTGTCCCAATCCCCAATTGCTCGCCCGATTAAGCGG

AGGGTTCCTGCAGCCCGGGATTAATCGGAGGCACCTGTTCCCTCGAGAAATTTCGCCGGAGGGGACAAGCCATCCCCTGG

CCGGGATTACTAATTGCTGCAGGCTTATCCCCCTGCGTAGGGATTACGCCGCCTACCTTTAAAACCTGGGTGGCTCTTAA

CCAGGAGAAGGATATTTTAATATTATTTTTATCGCCTGACCTTATGTATCCCCTTAGCATCCCTGCGGCTGCTCCTATCC

CTCAGACCGCTATCCCGAAGGCTCGGTAGCGGCTAAAGAGGCATTAGCCCCCGTACCTGGGGCATAGTTTAAGGAAGGGT

AAAAAGACCTTTGCTACAAGACAGCCCTCCTAGCCACTAGTTTTCTGGGACGCCACAGGCTTGCGGTCCCCGTCCCTCAA

AGCCAAGTTAAATCCCTCCGGGATAAAACCCCGATTTTTGCAAAAGAGATTTTTGCAAAAATTCCAAAATAAAAATTATT

TTCGTGTTATTTTTAGATTTGGTTTTGAGGGACCCAAAACGGGTGTCGTTGTCCCCATTTGCAAAACACTAGTGCCCCCG

ATTCTCCCGAGGGACTTATCCCAATCCCCTCAATCTTCGATCCGGGATCCGGACTAGTTTGCAGGAAACACCAACTTGTT

TGTTTTCAAAACCAGAAGTTTTAAAAACTCGTTCCTGAGGGCCAGGAACCTGGTCCCGAGGTGGGAACATGATTTCGTCC

CCCGATTGGACTAGTCCCTCCCCGGGAGGGACTTATCCCAATCCCCGATTTTTTAGGGGATTGGGACTAAACTAGTTTTG

TGGGACAACGAACTCTTTTTGTCCTCTGCGTCCTCTCCGAAGGAGAGGATGCTGCGAAACCCCTAAGTTTGAAAAGAGTT

CCGTTGAGGCGAACGAAAACCACTAGTTCTCTCCGAGGGACTCCAATCAATCGTCCCCTTTCTTTCTCCCTCGGGGAAAT

AACTTTCTTTGATTTTTAATCCCCGAGGGATTCCTAAAAAGGCCGAAATGGAATCATTCATTCCTTACGGATTCCCGCGA

AGGGAATAAAATTTTTAAATTTTGCAAAGCAAAAATATCCAAATAACTTCTTTTAAGCCAATTTTTAATTAAAGAAAGGG

AGGAATAAAAATTCCCCAATCGATAAACCCTTCGGGAGTTTTTAATAAAATTAAAACCCCCTTCGGCCCTTCGGGGAGTT

TTTCTGTTAATTTTGGCAGAGAAAAATTAAAACCCCCTTAGGCGGGGAGCCCCCTTTTTACAGGAAAACCCTCTCCCTTC

GGGGAGTTTTCTGTTAATTTTGGCAGAGAAAATTAAAACCCCCTTAATCGATCGGGGAGCCCTGAGGGTTTAAAACACAG

GAGGAAAGAAACCCTCTCCCCGCCGCCCTTCGAAGAATGGGGAGTTTTTTCTGTTAATTTTGGCGGAGAAAATTAAAACC

CCCTTCGGCGGGGAGCCCAGAGGGTTTAAAACCCTCTGCCCTTCGGGGAGTTTTTTCTGTTAATTTTGGCGGAGAAAATT

AAAAACCCCCTTAATCGGGGAGGAGGGTTTTTACAGGAAAAACCCTCCGCCACCTGGGGAGAGGAACCAGTTAGATTCCT

GTGGGACTAGTCCCTCCCCGGAGGATTTCCTTCCCTTCCCAATCCCCCGCGGATCGATTCTGATTGGGGATTGGGAAGGG

AAGGGACTAGAAGGATTAGCTAATACTAAAGATCAGGTATTAATGAGACATAAAAATTAAACCAATTAAAATATACAAAG

CATAACTAGTTATTACCTGTTTATCTAAACTTGCAATATTTCTACTTAAATAAATTAAAGCTTTTTCTATTCCATATGGA

CCTAATAATTCTACACTTCCTTTATCTATAACCTTAGTAGTCTGACCTCCTAACATTAAAACAAGGCCAGTTATATATTT

ATTATATAATAATTCAATGAAAAACGTTGATTAAAGAAGCCGAAAATATTATAACCTAATCTAGAGAACTTAAAATAAAT

AAGAACTTTTGACCTGCCATAAACTCTGAAAAACTATTGCTAAAACACTAAAAGATATTGTAAATATTAGAGGTAATAAT

TTAAATAAAAGAGGTACGGCGAACTCCTGATCCAACATTATTTCATGTGTAGGATGAATAAATAAACTATTATCCGCAAA

GAAACCTGAACCTAATCCAATAAAGATATCTTTAGTTATATAACCAAAGAATATGGAGAAAAGAGCTAATATAATTAACA

TGGCTATCATATTTATCATACAAATATAAGTAATACGGGACCACCCTTTAATACGAGGCGTCGAGATAGGGGTTGAATCT

AATTAAATATAGCTACAAAATAAATTTTATAAGGCCATTGACTATATAAAGAAGAAGTCAAAATGAAAACTTATGTACAT

AAAAATATTCGAGGATTACTTAATTCTTTTACTAGTTTGCAGGAAACAAACTTGTTTGTTTTCAAAACAAGTTTTGAAAA

ACTAGTTTTGTGGGCCACGAACCCATCGATAGTGTTTGTCCCTCATAAAACCACAAGTTAAAACCCCCAATGGGGTTGTT

AGCATCCTTCCTCTCCAAAGGAGAGGAAGGGATCCCAGAGGAACAGGAACGAAAACCTAGTCCCTTCCCTTCCCAATCCC

CCGATGAATCAATCACATCAATCCAATCGCCCGGGATTGGAGGGAAGGGATAAGTCCCTCCAGGATCATCGGGGACGAGT

TTTGTGGGCCACGAAACCCCCGTTTGTCCCTAAAACAAACAAGAAGTTAAAACCCCACCGATCCCTTTGTTGCATCCTTC

CTCTCAAAGGAGAGGAAGGGATCCCCAGAGGAACAGGAACAGGAACAGGAACGAAAACCTAGTCCCTTCCCTTCCCAATC

CCCCGATGAATCAATCATCATCAATCAATCGCCCGGGATTGGAGGGAAGGAATAAGTCCCTCCCGGAGATCGGGGACTAG

TTTTGTGGGCCACGAACCCCCAATCCGATCGCCGATTGATTGGGGTTTTGGTCCCTCAAACCACCCCAATCGATAGTTTG

AGGAACGCAAAACTAGTTTTGTGGGACAACGACACCCGTTTTGGTCCCTAAAACAAACAAGAAGTTAAAACCCCACCGAT

CCCTTTGGGGTTGCATCCTTCCTCTCCAAAGGAGAGGAAGGGATGCAGAGGAACGAAAACCTAGTTCCCCCGATTCTCCT

TGGGATCTCCAATTTGTCCCTTGGAGGAACCAGAACAAGTTCCCCGATCCTCCTAGGGACTAGTTTTGTGGGCCCACGAA

ACCCGTTTGTCCCTAAAATAAAAGTTTGAGGAACGAAAACAACCGAGTTCCCCGAGTTGGCCCAGGAACTGGTCCCGAGG

GAACCCCTTTCAGTCCCCCGATTGGAACTCCAATCGATGTTTGCAGGAAACACCAACTTGTTTGTTTTCAAAACAAGTTT

TGAAAAACTAGTCCCTCCCCGGAGGGACTTATCCCTTCCCTTGCCAATCCCCAATCGATAATCGGATCGCCGCCGGATTG

GGAAGGGAGGGACTAGTTTGCAGGAAACCGAAACCCGTTGGTTTTCAAAACCAGAAGTTTTGAAAAACTAGTTCCCTCGT

AGAGGGACTTGTCCCCCTTTTCCCCTTCGGGGAAAAGAACTTTATTTTAATCCCCGGAGGAATTCCTAAAAGGGGAATCA

TTCACCTTACGGATTCCCCGCAAGGGAATAAAAGTCGCCCTAGTGGTTTGGAATTTGGGCTTTGCAGCCAAAATCTTAAA

ATAAAACCCCCAAAAATTTGGGTTTTTTACTAACTTTATTTTTAATCCCGGAGGGATTCCTAAAAAGGGGAATCATTCAC

CTTACGGGATTCCCCGCGAAGGGAATAAAAGTCGCCTTCGGGTTTGGAATTAAAAGAAAGGGGAGGAATTCCCCAATCAA

TCGATAAACCCCGGGAGTTTTTAATAAAAATTAAAACCCCTTCGGGGAGTTTTTCCCTGTCAATTTTGGCAGAGAAAAAT

TCCAAAACCTTCGGAGGAGCCTGAGGGGGTTTAAACACAGGAAAGAACCCTCTCCCCTTCGAAGAATGGGGAGTTTTTCC

TGTGAATTTTGGCGGAGAAAATTCCAAAACCTTCGGGGAGGAGGGTTTAAAACCCTCTGCCGCCCTTCGGGAGTTTTTCC

TGTGAATTTTGGCGGAGAACAAATTCCAAAACCGCCCTTAATCGGGGAGCCTGAGGGTTTTTACAGGAAAACCCTCCCTC

CGATAAATGGGGAGTTTTTCCTGTTAATTTTGGCGGAGAAAAATTAAAACCTTCGGGGAGCCCGGAGGGTTTTTAAAACC

CTCCCCGCCCTTCGAATGGGGAGGAACAAGTTACGCCTCCGGCGGCGATCATCGGGGACTAGTTTTGTGGGACAAAACTT

ATGGTTTTGTCCCTCAAATCCAAGAGTTCCGCCGATTTGAGGCGGAACGAAAACCACTAGTTCCCTCTCCTGGGTACTTA

TCCCCTTTTCTTTCCCTCGGGGAAAATAACTTGGGGATTTTAATAAAATTTTGGAATTAAAGGGAGGGAGGGTTTTTTAC

AGGAAAAACCCTCCCTCCCTCCCTCCGGGAGAGGAACAAGTTACCCCGGCCTTAATCCCGCGCCTATCGGCCGGCGGATT

GGGACTATTTTTCGTTCCTCAACGAACTTGTTCTTGTTTTGAGGGACAAACACTTTTGTCCCTACAAAACTCGCGAGTTT

TGGGGACAAACGGTTTCTTGTCCCTATTCAAAACCAAAACCCCCAGATTCTTTTTTATCCCCCTCGAGGGGATTTAAGTT

TTGAGGAACGAAAACCTAATTAAAACCCGACTTAAATAACCGAAAATCGTATCATTTTTAGTAATTAGAGGGAAACTATC

AAAATGTTGTATAATGCTAACTTGTAATTCTTTAATAGAAGAAACTCTATATTGAACTTTTCTTTCCCCATGATAGCTTA

TACTTCCTATACCCCGAAGAATATTTGAATTAATTGTAAGATATAGAAATCTTTTTATGTAAACATATACCAAACTGTAA

ACTTACTATCAACTGGCCCTCCCCGGAGGGAGGGTTTTCATTTTATAAGATTTATTCAGACCTATTGAAAAGTAGCTTTC

TCCCTCCATCTGAGAATCCGGATACAAAATAAGGGTTTAACTTTAGTTGTCTTACCGGAAGAGTAGAGACATTTCTAACT

AAGTTAATATTAAATATGTTTGCTAAGAAAGGATTATGATTAGATAGTTTATTTCTAAACCCATTAGAGTACACCTTAAA

CAAAAGAGAATTTATCTATTGTCAACTACCGTATACTCGTTGCTCTTTTACAATAACAGGGTGTTTAAGGGCTGTGGATA

TAAATAGATCTGTTATTGACTTAGATCCGCGATAGCCCATTTCTCTTTCGACAGAAAAATACCATTTTTCAGCCCTGCTT

CAAGGGGTGAAAGCCCCTTGAAGCGAAGGGTCATCTTATTCTGACTTATTGCCATACATGAGTAATTAATTCATCCACCT

ATAGTCTGCGATCGACTAAGGTTTGGCACCAGAAGCTTTAGGGTGTCCCCGGAGTTTGATAGTTTTACCCGCAATAGATG

ACATCCCACGTCATCCGGCAGGTATACTCATAAATATATCCCCTTCATGTGCTAGAGATTTATAATTAACTATTGGTCCA

TTCGGATTAGTTAAAAAGTTAAATATAATACCTTAACTGAATATAAAGTAGTAAAGATTGCTCCGAGAGTTGCTATAAAG

TAAACTACAGTACCACTAAAGTAAAATTGACCGTATGCTGATTCTAAAATAAAATCTTTACTATAGAACCCCGTCAAGAA

CACCAATTCCTGCCTATAGCATAAAACCCCCTAGAGGGAATAACCTAAAAATAATTTGGGAGTTAAAGATCATCTGTTAA

AGAACGGTTAAGAACTTATTTTCTCCGATTTCGTCACCACGAGGACTACGCGTAGTACGGGCGGATTCTTTTGAATTCAA

TATCTAACTGTCTTAGGGTCTACTTAAAAATACCTCGCGAACTTCGGCTAAAGACTAAAATTCTCTAAGAACTAGTCCGT

TTTCATCTTTAAGTTGCACGGTTAAAGAATCACAGGGGAATAAACTTATTTAAAGACTGGGGAATTAAGATTTGGCCATC

TTCCCTAAATTAAAAGTTAGAGGGGAGACTAAGTAATTTATTAAGGCGTTGCATAAAACTATTCTCTACAACTTTAGAGC

TCTGGTTAGTAGATAACCTTTTACTATTAATTTTACTTATAATAAGTTTAATAACCTCTTTACCTTCCTCTGTAAAATGT

AGACCTTTTTCTTTAAGAATTGCTATAGACTTAAAATCATTTAAATCCGAAACGTTTATTACTATGTAAAGTTTGCCTTA

ATCAAAAAGGTATAAGAATATTATTTAGAAACTTGGTATTCTCAACTCATATGCTAACCTGAGGTACTCAATGTGGCCAT

ATTTTCCTTTCACTAACGGAAAGGCTTACCCAGCACCTAAAATATTTATCCGTCCCAAAATAGCAGCTACCTTATCGTTC

TACAGCTAAATTATTCAAGAAATTTTGAATTTAAACCATTAAATCTAAATCTTTAACAGACTGTGTTAAATTAAACCTTA

AGCGAAAATTACTTTTTCTAATACTGTATATGAACCCTCACCTTCTAGTAAACCCTAGCAACCAGTCAGAAGTAATAGAA

TAAGAACGAATTTGACCAGAAGGCCATTCAAAATTCGTTCGATTTTTATTCATTGAACTTATGATAAGATTGATCTGTGT

CTTTATTTCTAGACTTTTTTATTCGAGGTATAAAGTTAAAAGCTTTAGGCCTTCTTTTCGAAGAAAAGATGGTAAAAGTT

TAGTAAAGCTAAAAATCCTGTTTTACAGTATTTAAAGGCGCGAAATTAAAAATTTCGATTATTCTTTTAATATCATCTTG

CCGTGTAACGACTCAACTCTTCCCTTTGCTGTTTTAGAAATATCACCCATTTAAAGAGTTTCTTGTATAAATTCTAACGT

TTTTAAATCATCAATGTGTAAGCCGATAGAAAAAGTAAACTGAAAATGTGTCTCTCCTACAGTACCGATTGAAAAACAAC

CCTCACCATCCGTAAACCCGCGAAATCATTCATAAAAAAGTTTTTGGGGTTTTTTAAAATCCAAATTAGCTCTACCTCCT

CGATAGTACTAATCATATGGCGAAAATTTCGACCCTGAAAACCTGCCCGTCTTGCTGCTTCGCTGTATAACACTTGCTGG

ATTTACTAAAGCCAAAGTGCTTTTAAGTTACACCCTGTTATTTATCTCCCGACCTCGCTGCTTCGGAAGGAAGGAGACCA

AATTATTCGCCGATGATTAATGGCCGCCTTGACTTATTATATTGCAAATGTAAAAGTAAAGTTTCGAGGTGAGAATAATA

AGCAGTTATGAACACAGATCTCCTCCCTCATATTAAAATTACTCAAAGAAATAATAGAAACCCTATTACTTTATTCCTTC

TATTTGTGAGGGCCATTGAATGAAATTATTTAAAAGTTTATTTCTAAACCTTGTAGAATAAACCTTAGACATGGGAATAT

TTACCATGCCAACCATCATCTACTCGTTGCTCTTTTACAATAACATAATTAATTGTTATTGATTTAGATCCGCGATTGTC

CATTTGGTTTTCACCAGGCCTACGATCCGTAGCCAGCCCCAGCTCACCATTTGAACCAATGGTGAGCGAAGGGTCATCTT

ATGGCTTATTACCATACCTGAGTAATTAGTTCAGCCACTTGTAGTTTTTCAACTAAGAAAATCCAATATTTTATGAAATT

TTTGTGATCCAAGAGATCACCAAAAATCTACAGTTTGGTACCATAAACTTTAGGAGTTCCCGGAATTTGGTGGTTAACCC

TTCATTGACGGGTGAAAGGAAATGCAACTAAACTAAGACTAGCAATTAACATAACAGAATAAGTTAAAGGCAAGAATGGT

CTTAGTCCTCCATATTTTCTAAAATCCTGGTTATCCGCTACTGCATGAATTACTGCTCCAGCTCCTAAGAATAATAATGC

TTTATAGACCCGACTTTTAATAAATCGTTAAGATTTGGGGTTATCAGGTAAATTAACAGGAAGTTGTTCTTTATTGAGTT

GAAATATCGCGCCTTCTTTAATTACAGCGCTAGGAAATGATCAATAAATATATCTTCATTTATGTACTTAAAACCATCCT

TTGTAATAGTGTTATTATTTCTATTACGCCTTACTGTTCCCCGATTACCTGCTTGAGAATTATTAGAAAAATATTTAGCC

ACACCAGTCAATGTATTAACTGCAAAGAATCCTGAGGCTTTTTCGTTAACCCGGGCAGATAATCTGGATGTTTACAAAAA

TAAAAATTCCCTAACTCAATGTTAATTCCACCTGGTTTAACCAAATTAAAATATCTACCTATTCTATTATCAAGAAAATT

TCTTTGTTGCTTTAGAGGTAGTTTCTCCAAATCGGTAATACCTGAATTTGGATTCAAGTTCATTCATAATTCACGTTGAT

TATTATAAGCACCATAAATTTCCAAGGGGATTAATATTTATAGAATAAATTTTACCTGGAATTAGATCATATAAAGATTT

ATCTTTTAATTCAACTAGTTCCCTCGCCGAGGGACTTGTCCCTAGGTGAGCGGAACAAGTTCCCTAGGGACTAAAGGATA

TTTATCTTTTGGTTTTAATTGAGAATTTATTTGTTCATATCTAAAAGGTACACCTTTTTCTTTTAAATATATAACAAGGT

TTTCCCTTTATCATTTGTTATATTAATACCCTTATATCAATTCATATTATTTCTAACCGTACCTACAGATATCTTCAATT

GATCTGCTAATGAATTATAAGAGTTAGATTCTGCTAATAATATAGTAGCCTCCCCTTGTATTTGGAACTTGTTGCAAACG

CTGCTTCTTCTTTATTAAATCAAGCTTGATATACATGAGTTTCATCTAAACTTAATGATAATTCATTAGCTTCAAAGAAA

AATTAAAAAATAATATCTTTTCCCCATTTTTCTTGATCTTCCCTCCGGGAATGATATTAGCCCCCTTCCCCGGGCTAATG

TTTAAATAGGGTTTATAATTAGTATAAATACATTGTTCTATTATACGACTAACATACTGACCAAAACCTCGAAGTATTTT

TGCACCACCCTTACTTAACAGGAGGGAATGCATAATTAATACTATATCATTCTTGAACTATATTATCGTATGTAATAATA

GGTGCTCATTTAACAAACAGGACAGGATGATGATACCCCTCCCATTGGCCGCCATTACTCAATCATGCAAGTGAGATCTT

AAACGATTACCATAAAACTGTTCATATGGTCATTAAGACGATTTCGAAAAGATATAGCAGAACCTATTCCATATTTCCCA

CTTTCTTTATGCACAAAACAGTAAACACCTGAACAATCACCATCTATTTCCCTCCCTCCCCGAAGGAGGAGGGCGAGGAC

TTTTATATTCAATGGGTAAATGATACATAATATTAGGTTTTATCGTTTTAAACTTAACTGCATATTAAATCTAAATAATT

TATTAAAATTCTTACTTAAAACTAAAATAACAAATTATCTCCTTTAAATCCTGAATTATTATTTTCAGGTAAAAGATTAA

TCATATTAATTAAATCATGTGAAGCTTTTATACCTTTTGATGTTAAGAACAAAGGTAAATTTAATTTATTTTGATCATGT

ATATGATATATAACATTACTCAATAATAATCCACCTCTAGACTTGTCATTTAGTATTGTATAAAAGAAGAATGAATTTTA

TAATTGCCCCGGGTCCCGCCTTAGCCCTCCGGCAAATCCCTACGCGCTACGCACCCGTAGGGAGGGGTATGAAATAGAAT

TATTTTTTCCTTTAGCCCTCGATCTCCCCTGTATTAATCTGGGGAATAATAGGTGTAATTAATAATGAACTTGAACATAA

ATTAAATAAATATTTATTTATTATAAATTCCAACGAAAACATAGTAAAAAAATCATTTAACTTGAACATACCTCCAGCAA

CATTAGTAAAATGGGGTGATAAACAATATTCCCCTGCGGGAATCCGTCAGGGATTCCCTAAAGGGAATCCCTCTGGGGAT

AGGTGTTAATTTAATTTTAACTACATAATATTACCTTAGATTGAACTAGCTATTACTTATTTACTGGTATATGAACCAAA

AGGAAAGAGGATTATAAAAATAACCAACCTCAACCCTACCTATATAATAGCGAGTAAACAAATTTTTTGCATTTTAAACC

TCCTACGGAGTACGCCTGATTGAAAAAGGCAGGCCTCCCGATTGAAAAAGGCCTCCCCGCCCAGGAGGGAAACGCGAGGC

GAGTTTTAAACAAATTTTAAACCTCTCCGGAGGTTTAAAACGCTTTAACCCCTAGGGTCTTCCCTTGGGCGTGCAAGCCC

GCCCTGCGGGCTAGAGTAAAAGAAGAACAAATAAAATCCCCGGAGCTTTTTATAAAAGCATCCTTCTTCATCAGAAACAC

TCCCCTTAATTACTGCGCTAAGGAAAAATAATGTAATTACCTGCAACATATCACTATGTTGTTTAGGCCATACCTTAATA

TAAAAACGTTTAGCTCCCTCCCTCTTCTACATGGGAGGCAGTAAGTGGCGGACCGCCATTTGGGGTTTTAGGCTCAGCAC

GCGTAGCACGCGTTGCACGCGTAGCACGCGTTGCACCCGTTTTATGGGGACTGCTGGAAACAAACTCGTTTGTTTTCAAG

TTTTGCAAAAAACTAGCCCTCAGCAAAGCTGGCCCGCATGTTTCCCCGGGAAACTAGCCCTCCATTTTTTCCACCGTAGG

ATCCGAAGATGCTTCGCTACAAAAAAATCAGGTTTCCCTTCCTACGGCCTAGGGATTAGGGAAACTAGTATGAAAATTCG

CGCTGAATTATAATCACGCACGTCAATAACTTATACTCTAGAGGTTACTCTTACTTAGAAGAGCCCTAAGCTGTATAGAA

TAAAACGACTTTAAATATTTTTACGTCTGGTCGTTGAAGAGTTCATGTATTTTAATTATACATAACTTTCCTGCTGATTG

TCTCTCAAACCACGATTCCTCCCCTTATCCCCGCTAATCGGAGGAAAATAGGGGATTAGGAGGGAGTGGTTTTATTATAC

CTACCTTATTAGATAACTAAGATTTTGGAGATATTCCAGCAATTTGTAAAATTTTACATGCGCCCTATAATTTAACGCAT

GATTAACTAAGTGGAATAATGCTATGTTATATGAAGATAACCCTACCGCTATAACCATCATCCCCAATTGCTTCAATATT

ATCTGATAAAATTTGTTGTTTGATATATTGTTGGACCATTTCTTTACTAATTGAAGTGTTAACCTTCGTATTTATCTCAT

TTTTCTTTTCATTTTACTCATAAATTCAATTGATTTAAATCTTCACTATTTTGATATAATCTAAAATTATAAAAATCTTT

AATTAAATTTACTCTGGACGCTTTTGCTGATCTTAATGGATATTTTATGAAATATTTATCTATTAGATTAAATAGTTCTG

ATTTTCTATATATTATATATTTAAACGCTTCTATTTTAGCACCTATAGCGTCTACTCTACCTCCATATATATTAATCAGA

GGTTCTAATAAATACATATTTTTCTGAGATGCTGATATAAATATCTGACCGGATTTAACATTAACATAAACAGAACCGTC

ACTATCTATAAATCCACTTAGTCATCCGTTATTAAATGTTAAAGGTTTAGGGTAGATTAATGTTATACCATATTTAATAC

ATAATTTATTCATTTGTGTTAAACGTGTAGGATTTCTTATCAAACCATTTACGTTATTAATTAATTTAATTAAATTATTT

TTGCTAGTTAATTGATATTTCAATGCTTTTGCATTACTTATTTTATATATAGAACCTCCTAATTTTTGTTGTATATATGT

AAGTGCAGCTTTATCTCTTATATCCATTACTAAATTAAAACGTGCTATACCTTTTTTGATAAATTAAAATAACCATCTCC

ATCTATAAGACCAGCCAATCACTCGTAAAATCTAAGTTTTCTCTGGTTTTAGAGGGTAACGTTCATTGTTATTTGTAGAG

GGTAACCCTTTAATAAAAATTGATTTATTTTCCTATTTTCGTTAGAATCTAACAATGTAGTATCTATAATTACATTTAAA

TTAGATAAAGGGCAATGCCTTTTAAATATACTACCTTTAAGAAATAAACCCAAAACTGTGTTAAAAAATAATATAATACT

TTTTTAACCAACCAAGAGGCTTTTATACTTCGTTTTATTTTGGGTTTTATACTTCATAAATTCATTAGTATCAAACGTAT

GGCCTCTGAAATTCCTACTAGCTTGCTGATAATAATAATCTTTCATTTTTCTCCTTTGATAAAATAAGTATTATTTTTAT

ATTAATGTATAGGAGCCTTAGATTGGAAAATGAATTAAAAAGATTAATACTAATATAATTACAATAAATTTTAACTGTTT

TGCGTTTATAATAATTAAGAGCTTTGGTTATCTGCGAATTGTTTATTAATAAAGGCATGCCTTTAAAAATAAATTTTACG

TATATAGTTTGATGTCTGAACATATAAAAAATTTTCTATATTGTTCTCGAGCCAATTGACTCATTGTGGAATAAGCTATA

ACTTTTTAATATCTTGTTGGAATAGACCGACAAGAGAACTAAATACAGTTGTTATTGCTCCAAGTCATAGGCATATAAGT

AATACAGTACTACTATATTCTATTAAAGGTGATGAACGCATCATTAAATACACTCCCGCGGTTCATTTGTGTTATTAACA

TAGATAAACCCTATGTGTGTATTTTATTAAAAAACGACTTTACTTATTCTTTATATAATTAAGATTTTTCCTAGCACGCG

CTAACGGGTACAACACTTTAAATCTTATAGTAGTCCCTAAAGTAGATTTAAATATTCGATTATTTGGCAACTTTCTTAAG

ATAAACCAATTGTCCACCAAATGATACTGGTTGATCTTTTGATATCTTTTATATACTAAATTCCTACCTAAACCTAAATA

TTTAATACAGTCCGCAATAGAATCAAACGAAGCTATAAAATTTCCCCTTCATCCTGTAATTCAATAGCTTGAGATTTTTA

TCCGAACGAAATCTATTTAATGACTTAATAAAAATCCTTCCTTCTTTTGCCTCTAAATTAGAAGGACCCTTTAATAGTAA

ATCAATTTTGGAAAGCAGAGACACCCGATCCACTTTCGGTGTATTAGAAGTTGACAATCTCTTATTGTTCATTTGGTTAA

GTATTTCGTCTAGTACCTTTACCCCTCTTCCGTGTACTGAAGACCTAATTCTTTTAATTTTAAGATCGCTTTTCAATCCT

GATAATCCAAAAGCTTTTACTATGTCAGATCATAGAGTCAAATAAAGGAATTAACTTATTAATTATAAAATCTTGTTGAC

TAATTTGTAATAGAATTACATGATTATACTTAACCAAAACACACGGGGAGAGGTGCTCCCCGCCCCTTCATTTAAAAACT

TTTAACTTCTTCCATTAAAGGTAAATCCTTAATAGATTGACCTATAGAAAACCCTAGTCTAAAATTATCTCCTTTAGTGA

TAAAAATGAACCTTCACCCTCAACAAAGCCAAGCAATCAATTAGAAGTAATAGGCTGGCCTTTAAAGCTGTTATAAAATT

CCGATCTAAGAGTGTTCATACCAGCTTTAATGGTTTCTATTTCTTTTAGTTGCTCTATTCTATTACCTAGATCTATAGTC

TCTGTGTAAAGGTGGAAAGCTTTTTAAAATCTAAGAAATTTAAATTTTTGTGTGTTTTAAGTGGGTATTGAGTGAAAATA

TCTATTATAGTCTTTACATCCTTTTTATTCTTGACAATGAAATAAGATTTTTAAAGTATGTATAGACTTTACCAAGACCA

AGCCTTTTGGATAAACTCTAACAAACTTGTATCGTCTACGTGTAAAGAAATAACATATGTAAAACTAAAATTAATTCGCT

CCGAACTTTTTCTGCTACCAATCCCGAACGAACCTTCACCATCTGTAATACCACACAGTCATTCAAAGAAATTACTGTCA

TCCTTCTTATATGAACTGCTATAATATCTTCTTTTACCTGTATTACATAATCCCCTTACGGGTGAGTTAAATAATCTAGA

GAAGGTAGCCGAAGGAAATATAGAAGAAAAAGTCGGATTATTTTTCTGTATACTTCTAATTGAAGCTGCAGAGTATAAAC

CTTGGTTAAGTGTTGAAGAATTAATTAGTATAAGACGGCTAAATTTATTATCCTTATTACAATGGATAATCTTATTTATA

TAATAAGCGGAGAATAGAGGTAATGAAGCCAAAAATTTTAAAGTCGAGGTAAAATACACATCTGCGGTTCATTTATGTTA

GTTATTCTTTAACGAGCATTTAATATAAAATATAATTTTCATACTCTTGAATAACACCTTAAGTGTTCCTAAGGATCGGA

CTGTACCTTTAACTTTTAAAGATTTTTATTCATTTCTTTTAGGCGCAAACTCAAAGAACGAATTTTATTTAAACCTATTT

CATTAAGGTGTTCTTTTTAAGGTAAGATTATATATTTTACATCATTTAATGAAAACCAGAGATTTTTTAAAGGATACCTT

TTAAATAGTTGGGGACTATTAATTTAAGTTTATTAAAAGAAGAAATATTTAATATTCAACCTTCCTTCTCTTTATTATAT

TTTATATTTATAGGCCACCCCTATATTTAATATTACACTCCCGATAATTTAAAATATAAAATTCTTTAAAAAATAGAAAA

GGTAAAATAAAGAGTTTTTGAATTCTTTATTTTCTATTAAACCAGTAAAACTAACTTCCTCCGGACCCAATGCATCTATA

TAACCAGATAATCAACCTGTCATTAAACTAGGCTTTATAACTGTATCTATAAATAAAATTTCTGTTGAGTATTGGCTATT

AAAAATATTTAATCAATTTTAAATTCTGGTTTTATTAACTGTGCAAAAATTACCATTAAATAAAGAGACTAATCTGGAAA

AATTCTCTCTGGATGTAATAAAAAGAGACAGTTCCACCTGTACCCGAAGGATGTGAATCATTAAACCCTGATCTTTTATC

ATTACTTTACCAAAACCTAATCGCCCTCCCAGAGGGGAGGGGAATTCTGGTTTTATATAGTAAATAACTTGTATATCCTT

TATACTTACACTAATATCAAAGTACACTTTGTTATTTAGTATGATAAACGAACCTTTTCGGTAAATCCAATAAATCATAC

CAAAACCTTTTATTTTTATGTTGAACTTTTGGTTTAAATAATTATCAAATTTAAAAGTTATATCGCATGCAGTCTCTGAG

GTACCATTATTAAAACTTAACAATGTTACCTGCTGATTAACCATTCACCCGCCCTCACTAATTTTAATCGGGTGACACAA

GATTTTTGGTACATCTTATAACGTAGTGCTCAGAAAATATATACTAACTTGTTGTAAAGTTGTCCCAGCAAATAGCGATA

TGATAATTGATTGATTAACATCAATCAACGGCAAACTATTGACACTATAAGTAATAAAATACCTCGGGCGCCGAAGGCTA

CCCGGGCACCTAAATCCCAAAGAAACTTTAAAAGGTATACAATATAAAAGAAATAACCCTGAATACATAAACATTAAAAC

TTTCTGTATGTATTCATTCCATAGGCTAACCCCTTCAGTTCATTTAAACCTTCTTAAGTAAAATGCCCCTTTTCTTTTAT

AATATGTATACCTCTGCAAAAATCTAAAAATCCTGGTATTTAGCTCCATAAATAGAACATTCTTCAAATAAAGGTATTAC

AATATTAACTAAATCGGATATATTTGCGACAGATATACTATATCTGTCTCTATCCCCTGTAGGTTTTACTAAGGAACCAC

AACCTAAAGTATCTACTATTCTTTTAGAACAATTAAATCACGCTCATGTTGTGTAATACGAAACTGCGGTAAACAAGTGT

AACCTAATTTCATTCTTGCCAGCTTAGAAAAGTTTAACCCAAAAGTTCCGTCTGCCTGTGCGAACCCTGTTATTCAATAT

TTATCTAACCCTGCATCACTAGACTTCGTCCCTCCCTCCGCAGGAGGGAGGGGCTAGACTCAAATACAGGTTTAATTATA

GGTATAAATTTTTCTTTAGGGTGATGTTCTAATAACTTAGCGGATAACCCTTTAGGGAATACAGATTTTATAGATAATAT

TTTATAAAACCCTTGTTCTGTCAAATGTTCTTTTTATAAAGAATATCCATAACTTGGCATCACAGCTTGAAATGAACAAA

CTTAGTTGTTTGTAGGGAAACTCTTCAAAATGCTTCAGAACATTTATAAGTGCTTTGATAGAAGAGACCTCATAATAATA

CATCATACCTGATTTACTAATCGAGCCAACTCCTCCAAAGTAGTCTTGAACTTTCTTTAATAATTCAAGATTTAAAGGAT

TCACTTCTGCACCTATAGAATATACAATGCTTACATTATAACCAAACACACTAGAACATTTTGTCCGTAAACGAATAGAA

AAACTCCCTTCTCCATCTGTATAGCCTGTGATAAACCAAGGATGTAATTTTAATTTACCCTCTGATTTAGTACTTAAATA

GTGTCTTTTCACCATTGTGGCCGATTTCGTAACCTTTACCCTTACTAAGACAAACTAGAGTCTATAGTATGAAGGGAAAG

CTGGTTATATACAATTAAGGTAGATTTACTAAAATACCGGCTATAATCCTACGAAGAGCGATTTAGCCTCATCATTCTTT

CTACCACCCGTCTCCAGGCTGCCTAGAGTACACCTTAACCCAAGAAAACTTAGGTTCTTGGGAAACTACCATCTACTCGT

TGCTCTTTTCCCGCACCATATAATCTAATTTGGTTAATCTTTTACTACAGCCGCCTACTACGCTAGGTATCAGATGTTTA

TCAACCCTGATAAAAATTTAAAACAATGGATGGTACGAGGTTAGATCCGTGATTGTCCTTTCTTTTCATCACTCAACACT

TATTACTATACCCTGCCCATTACAGCAGGCCGCACCTAAACTTTTCATTAGGTGTTTAGTATATTGAGCCTCGGAGTTCC

CGGAGTTTGGCAGTATTATTAGCCAGTTTCTAGTAGCTCACACCAGATCCAAGCGTGTATTAATGCACTAACTGGAGTAG

GCAAAATAGCCTACCTTCGGAATAAATAAAGCTCTGGAAGGGGTACCCCTTGAGCTGTCAAACCGTACATCTTTAAATTA

GGAAGTTATTGTTTTAAAATACTCGATTATAATTCATATTGTCTACCAACATTTTTAATTTCTTCAACCCTCCAGCTGTT

AAATGTTCTTTATTTTTAAGGATTAGTACCCCTTTATTAAAGTCCTGGAAATCCAATTGTTTAGCCCCATAAACCGGGTA

ATCTTTAAAGAAACTGGTTATAGAGCTTAAGCCTTTCGTAACACTAAAGGTTCACTCCTTTCTAGAAGTATTAGTATTTC

TAACACTACCACAACCCAATAAGTCTGAGATTCTGTTTAAAACTAAGAGATCTCTTTCATGTTGAGTTACACGGAAACTA

GCTTGTCCCGTGAACCCTAGAGCCATTTTCGGAGCTTTATAGTAGCTTAAACCAAAGATCCGTCTGCTTGTGTGAAACCA

GCAACTCAATGTCCATTTAAAGGATCAGAACTGGCTACAAACTCAGGTTTCTCTATGGCTTTAACTTCAGGGAAGGCTGT

TTTCAATCTTTCACTTAAACCATTAGGGAATACGGATTTAATTGCAAGAATTTCGAATAAACCGGATTTAACTATATGTT

CCTTATTCTCAATCATTTTAAGATTTGAGCTCAAAGTTTGAAATGAACATCTTTAGTTGTTTGAAGGGGATAATTAATAA

AATGGTTATGGATTATTCTTAAATCTTTTTATTTCGAACTGTATAGGAATATGTACTATTATGTGATGTTATAGAACCAA

TTCCTCCGAAAACCTTTGAACAGCCTCTAATAACAATAAGTTAGGAGTATTAACTCCGGCAACTATTTTAAACTCTGGGC

TTACAGAAAACCCTAATAGATAAGAGGGGTCTTTACGAATAGTAATATATAAAGATCCGTCTCCGTCTGCTAACCCGGTA

ATAAACCAAGGGTCCAATAAGTTAGTGCCGGAAGTTGTTGTTGTCCGAATAGTTGAGTATTGTCTAACATTAGTATTAAA

ACGACTACCATTAAATTTAGCTGTACGATAGAATTCATTCTCCCTGGAGTAGGCTAAAATTTATGTTGATTTCCACTATA

TACGACAGCGGCTTAGAAAGCTCCTCGTCGTCATCATACAAATACGGTAGAAATTCTCAATAATTTACATTATTGTTCGG

ACTATATCTTCAATCATACTATGAATTAATACATTTAGCTATAAATTTTATAACTTAACCCTTCAACAGTAAAATGCTTT

TATTTTTAACTAATGCATGGATTTTTAATCATCTTCTATATGAAATGAGTTTTTAGTTTTAAAGGATATTTTTCTAAATA

ATTAATTATTTTTCCTAATTTACTAAAATTAACTACTGTATTATATCCATTATCACTCACTTTTAATATGAACTATATAA

CCATAAAGTAAAGTTGCTACCTCTTTACTAAATTCTATATCATCCTTTTGTGATATAAAATATCTCACAGTAACTGTTGT

TTTACCTTGTTTAGATGTTAATATAGAAGAACTGAAACAACCTTCTGCATCTGTAAAACCAGACAATCAGGCGTTATCTA

ATTTTACTTTGTGATTACAATCTAGTAGTTGAATATTCATATTATATTTGTTATTAAAACCATTAACCCACACTTTAAAT

TGATTAATTTTGTATTTAGTTAAAATATTACAATTAAATATTTGGATTAATTTTAATATGTTATTTTTATCTCTAACTCT

ATAATGATGAGTTTTATTATTTTTATCTTGCACAGAAACAGACCCAAAACCTAAATTTTTCTTTATAAAAAGAATATTTG

AGCATCTACACTAGATTGTGTTATTTTTAATTCTAAATACCCATCTTTATTTAAAATAAAAGATCCATCTCCTTCTGCAA

AACCTATAAATCATCACTTAAATGTATTATAAATTTTAATTGCTTCACGTATAGTCTCTGAGGAACCTTTATCAGACTGG

CCGTCCTCAGCCATAAAGTTTCCCATGGATTGTCCCTCTTCTCGGATTTTTCCGAAGTTGACTGATGTCACAGAGGACCA

AGAATTCAAAACAGGATATTACCGCTTATAGTGAAGTTTAAGGAAAGCCCTGTCTCCTATTTCTTTTACTGGCAAATTAT

TTTTATTAAATTGTCGGTATATTCGGGCCACCATGAAATGGCCCAACCCCTCAAAGAACTCTTGAGTGGACCAATACTTC

AATAAGGATATTTTCGCACATAGTTTAAGGAAAGCCCGTAATAACTCCATATTTACCGGCAATATTTTGATATATTTAGT

TTTCGTGAGTAGTACGTTACCCTCCAACTCCAAAACCAATATAAATCAATACTCCCTCACCCTTGAGAATGGGGAGGGTT

GTCTTATTTCCAAGACTGCCCCATACCAAGGAAACTAGAATTACTTTTTAATCCTTCCCGAAGGGACCCCTTGGGTACCT

GAGGGTCCCCTGGGAAGTTTATAATACATTTCAGCTATAATATAAGGACTCACTATGGTTATTAGCTTATCCATACTTAA

TTTACTTACTCTAATACGTCACTTAACCTTTCCATCCGAGTTAGTACGTTTATTTATAGAAGCCTTTATACCAAAATTAA

CGTCTAAAACATTTATTAATGTTAAAACTTCATCCTTAGTAAAATTGTCTGTACATATTTTAAGACAACCATCGCTAAAA

TAAGCATCACCCATAATTAAATGAGCTAAAGCTACAGGAGTTAACAGCTCTTCTATATTTAAAGGTAAAATTTTTATATA

TTTACCGTCTATTACCTTATATCAAAGTTGATGTAAATTAGAAATAGCTGGTAAACGTTTAGTACTAAATCAAAACTGTG

TAGGTTCTTTACCTGTTAACTCAGGATTAGGTCAAGGTGTAGGTCTAGAAACAGTACAAATAGAAGCTAATACATCATAT

TTTAAATAATTTACGTAATGTAAAATTTTGGCCGAAAATGTCAATTCAATTCTACCATTAACAAGTGGATTTTGAAGAGG

ATCATATTTTAGGTGACCATCCCTAATAGTTCACCTGTCACTATTTCCCAGACTTTTGAAGGAATCACTCAACGAGATAA

TTCCTCTTTAGATAATACAGTAGAACTAAATATTAATTTATTATTTAAAAGTTTTCCTTTATCTAAATAAGCTAAAACAG

TACTACGACTAACAGCAAAAATCTTAGAGCATTCCGTTTTAGTTTTAAAAGGAGCTCCTTCAACTAAGCCATATAAATCT

CCAGATAAATTATTTATATCATAAACTCATATAAGTGGTTTATCTAAACCTTTACTATCAGTATGAAAACCTATTTTTAC

ATACTAACCATCCCTACGCGACAGTAAGAAAATTTATGAAACTTATTAGAATAGCTATTACTAAATAAACCTGAAGTATT

GAAATAAATATTGCAGTAAAAGAAATTGATTAAATATAAACCTTCCCAAAGCTCAAGGTATATAGTTGAAATATATTATT

AAATATATCAATGGGAGGCTGTTTAATAAAAATAGTAGTAGGCTATTACAATGAAATGGGCGAGGACAACTAACTATAAT

AAAACAGCCTCCCGATAATTTGGAACATTAAAATAAAATAAAGCTAAGAAAGAAAAAACTAATATAGCTATAAAACTATA

ATTTTTAATTACATATCTTCCTCTAAATGGTTTTACCATTTTTAACATTATGAGCAATAGACCCTACAAGACAGTTTAGT

GCTTTAGCTCCATCCCGCATTGAAGAATAAATTGTTTCAGTACTAGTCTCCAAGTCTAAAATTGAAATCTTTTGAGTATT

GTGTTGAGAAGCCGCAATCTTGGCTTTGTGTTCATCCGCAAGCCTTTTACCATACCTAAAATGATTTTGTCCCAACTTAG

CTGCTCTCATTTTAGCTCTTATTTCTTCAGAGAGAATTCTTCCAAACATGGGATGGTTTTCACCACTAGGTAATTGTTGC

ATAGCTTTACTTATATTTAATTTAGTTTCTTCTGTATGTATTCTCCTAAAGGAGAACCAGCTACACTTAGAATATTATAA

GTAGGTTTTAAAAGATCAAAATAAAATTGTTCTCTCTCTATAGTATCCTTAATATCACAATATTCTAATATATATAAACT

AAAAGATTGATGACCATATTTCAATAAAGCAGAGTTAATCGGCCTTCTGCTCTTTTTAATAAAGAGGAAGAGTAATAGCA

ACGTAATCTAGTAGTTAAATTCACGGCACTACCTATGTAGAATTTTCCATTTATTGTATTTACCCAACAATATATACCAC

TTTTACCTTTATTTTCAGAAAAGATACTTTCTTTTCCTCTTTATCAGGATTTAAGTATTCTTTCACAAATGTGAAAGGTA

ACATACTAAAACAGTAGTATAATGATATATCAAAATTAAATAAACCCATCTCTGTTATACAAGAGGGATTTATATAAAAT

AGACATAATCATAATATTGTGAATAAAACAACTAAAAGAATAACCCCATACCATCACTCATATAGTTAGGGAATGTAGTT

TTATTTAATTTATGACCAATATATTTATTATTTCAAAGCTCAACTTTCCGAATACACCTGTCAAATAAATAGAGCTTCCG

ACTGTACATTCAGCAGCATTTCAGCCACCCCGTTGGGATCCAGTCTGTAGCGATATATTAAAATACCGACGGTCTTTACT

ACGGAACGTATTGACCGTTTTCACCATAGTAGAATCTTCATACTTACGAGATATATTTGGTTTAATCCTTGCTATACCTC

TGATACGATTCTATAGCCAAATTTACTATAAAAATAAAATTGACCATTTGCGACATTTATTTGGTTATCGCCATTGGGAG

TCAAACGTGCAACAAATTATAATTTTGGACTATATCTTTAAACGATGTTAAAACAACCGTTTACTATGAGTGTCTAAATT

AAATACGGTAGAACAATTATCTTTGTTATCTCTCTAAACAAACAAAGTAGATGATTGAGTATGCTGAGAGGGGCTTATTA

TTTTGGGCTTATTTTTACTATAACTATCTATAAATATCGATTCGATTTAATCGAATCCCTTTAGGATTCCCTTACGGGAA

TCGAATTAAATTGGATTCGATTAGTTAAAAAGAAGAAAACCTCACCTCTCCCCAAGCTTGCGGGGATAGGACCTAAATAT

ATAGCACCTCCCTGGTGGGGAACTGCTTAATCATCCTCGTAATACATATTTAATTCAGGAGTTATAAAACTCAGTCTCTG

AGGACTTTTTCTAAATTAATTAATCCATGGTATGGGTTGGATTTTAGAATGCAAAACGAATGCAAAATTAAAGAAATATT

TAAGTTTGTTTCATAATATTAGTCGGGGCCAGGTACTAACGAAGTTTGGCGATTTTAATATACCAAAGTTAGTACCTTAA

ACTCATAAGGCTATTTTATTTTTCGCATACTATTTATGAACGCAGCTTTTATTTTTAATTCACGAAGTTGTCGATCATTT

AGAACAGAATTATTCTTTCGAATGTAAAATATCTCCATCCATTTTTGAATGATTCGGCTTTAACTGAATATAGTTTACGT

GTTGATAAGTAATCTATAAGGGAACCTAAATAAGAATAATTCACAACTAAACGATCATGGCCCTTCTGATCCTTTTCTGT

ATAACCGCCCATTATACTGCTTAAATAACCTAGTTCTAGTTCTGCGTCTTTTGACTTAGAACCATACGCTGCGTAATCTT

GTTATTAGTGATTGTAACCATAAAGGATCCCTCCGCTTCAATGAAGCCGACCAATCAATTATCATGGGCTGTAGGCTTAA

ATTCACCTAAGTTAACATCCAAGGGTAAAGATAAAGCCATTCCTGACATATCCGCTAGAGAGAAATCAGTATATTTATTT

GAAAAATTATTAACTCATTTCGTAAATTGTAATTTACGTTTAGTTAAAAATAGCTTACCTTTAAAATTGAGATTAATATT

CCAATACTATGCTTATCTTTAACCTGAAAATGTACGGAATCAGATCTTGCATATGAGTAGACATTACCCATGTTTAATTG

GCTCTTAATTTCGTATAATAAAGGTAAATCAACTTTATGTAAATGAATAGAAAAACACATTTACCTTGGCTAATTACAAA

TGACCCGTCTCCTTCCGTAAAACCTCTAAATCATTCTATAAATGCTCGGTTATATTGATGATACTTACCTTGATAATGTA

GTAAAACAATTTCTTTGCTTCGTAAGCTATCCTCGGTAACCCTACCTAAATTAATTAATTTAGTACCCTGCTGATTGTTT

CCCTGTCTTGGAAATTTTTCCGGAGTGCTATATCTGTTATATAAAGACTCATAGTTTAAAATTATTCTTTCAGTGTGGGG

AGCAACTGCCCCGGTGGTTGTCGTGTTTAGTAGTACCCACTGATAACCCTCGCCTGCGAAGGGTTCTTCTTGTTGAGTAA

AAAGTCTATCTTTAAAGGTGGTAGGTTCTTTTACCAAGAGAATCAGTGTAGGATGAATTTTAGAAAAATATCTGCCAACA

GTTTTACAGACTCAGCCCTAATAAAAATAACGGGTTTCCCATTAATTCTACCTATTGTACAAGAAAATCTATATCTAATA

ATTAAAACATTTATTAATCTTGTAAGATCAATATAATTATGAGAGAAAGTAGATAATACTATACCTGAAGGTAAAAATAC

TCCACTACTCATTAATCAGAAAGCTAAAGCAACTGGTGTTAAGATATCACATAAGATATGATTCGGCATAACTTTTCATA

CTTACTATTATAGAAATAATGATATAAACGCTTAAAGCATGGTAGTGCTCTAGTGTATAAATAAATTTGAGTCTTAGAGT

AAGTAGGATAACTAGAACAAAACGCAAATAAATTAAAAATATAAACCAAATATATTTCTGTTCGCTCAAAGGTAATCCTA

CTACTAAACGAACATTTAAAGAAGTCTTACCGGTAAAATTTAATTTACCACCTGAAAGCAATAATCCCATAACAACTCCT

TCAGTCCACCCGTTTAATTTAAGTTTATCTGCCTCTTTTCTACTTATTTTTGAATTAGTGGCTGCTAAACCGCGATTAAT

CTGAAGTAAAGGGACTGAAGATATAACAGTTAAATCAGTACTATAGCGTCTAATTATACCTAAACGTGAAGTGTAACAAT

AAGATGGCTTACTTCTATTACAAGCTGGCGATCATGCACTGCAGCAAGCTGCTGCAGAATACCGCCCCTTAATAGAATTA

TCCAGCTGCTGCTTATTTTCCGGCGACTGAAAGGAGTTCCTTACGAAGCATGCTGAGCACCCCCGGAAGCTCTATTTATT

GGTAAGCTCCTCCTATATTTTTTTGCTTTATATTTACCTTCGCACAAGCCCAGTTGTGCCGCCAGCTGCCGGGTTTGCGG

CACAACTGCTGGAGCTTGGGCTACGCATCCGAAGGGAAAAAAATATTTATTTATTTTATAAAATAAGGCTGCTCCGCACC

CTAACCCTGTCAGGTTATTGCTGCTGGAAAGGTTAATTATTATGCTTCAAGTATAAAGTAAACTTAAGCTAAGTAAGTTT

AAGTAACCCTCGGATTCTCAAGCATTTGAAAATGTTCCAGCAATAATCATAGAACTATTAAAAGTAAACTCTACGTTATT

TACTTTTATGGGCCTGCAGATTTGTTTATCCCAACCCACTTGTGAATGAACTAGAGAAGCAACTCTTTTACCAAGTTACT

CCCCTTTCCCGAACCGTACGTGATAGTTTCCCATCATACGGCTCTCCGATTTTAATCTATTCCATACCCCTCCCTCCGGG

GACTGAGAGTTATGTATTATGATTATTTTCGTCTCCTTTATGGTGATATGCTAAATGGCATGTTCTACATAGAGGAATTG

GTTTGCGGTTTCTTTTGGCCATCAAAGCATCGATTTTTTCAGTTTCTCCCCTCCTCTAAGAGGAGAATTAGGATCCTTCA

TCATTCTTACATGATGCATTTCTACTCTGTAGTCTGAGTCACAAACCGCACACTTAAGATTTGATAAAGAGGCCTTGGAT

AGAGTCTCGGCATAAAGAGTTGGAATCATCTCTTTGCTGTTTACTTTGAAATCTCAAGCATCCATACCGTAGACTGCATC

CACGAAACTGATCCTGTTTTCTCCTTTCAGATTACTTCCAAACTTGGTAAAGACTTTATTCTGAGTTTCCAATTTGAACT

TAGCTGCTAGAAGTTTAACACAGGAAGTCTTTAAAATGTAATGTATCGATGAAGAAATTCTATTCCTATTTTCTACGAAA

CTATAGTAATTGATTAATCCTCTATAAACAGAATTATACTGTGCTATTATTGTGTCTTTGTCGTTAGGTAATCAAAGGAA

TCTCGGTACAGGAGTGTTGTTTTCTAGGAACCCAACACTTGTAAGTTTCTTCAAGATTCTCTGTTTAGGAGCTTCCAATC

TTACTTCTCTACCGTTTCTTTTAATGAAACCAAATTGACTAGAAGAAAATGACTGGTGTGATTTACGGAATATGTCAGTA

GCCAAGAATCTAGCTTTGCCTTCCTTGGCGTTAGTTATCAAAGTTTTGCTATCACTTAACTCTAAATTTAGTTCTTGTTT

AAGCAAGATCTTTATTTTATCCAAAATGGTTATACAATCATCCTTAGAACCTCTGATACCTATGATTCAGTCATCTGCAT

ATCTCACATATGTCAATTTCTTGAAACTAGGATCCATAGCTTTATAGTACTGGGTTTTAAGAATTAATTTATGGATCCGA

GCCAGAACTTCTGGACTAGTTTCTTTGCTTTTCAGATATCTCAGTCTATTATAAACTGGATTGATCTTGGGTTTTGAACC

TGTATCCAAGTCCAATTTTAACCTTTCCACAAATTTATCTAGTTCATTCAGATAAATATTACTCAAGATAGGACTAATAA

TAGATCCTTGCGGAGTACCTGAAATAGAATGTTTAAAGATTCTAAATTCCATGTAACCTGCTTTCAAGGCTTTTCTGATT

AAATCCGTGAATCTTCTGTCTTTTATTTTTGTTCTATGATTTTCATTAACACATCTTGATCGAAGGAATCGAAACATTTA

GATATGTCACCCTCAATATATCAGGTAGCGACTCCAAATCTCTCTTTTATCTTCTTCAAAGCTGTGTGACAACTTCTACC

TGGTCTGAACCCATGACTATTATCAGAAAAGGTAGGTTCAAAGATTGCTTCTAAAATCATTCTCATTACCTCCTGGACCA

ATTTATCCCCAATCAATTAGGGGAGCAATAGTTAAAGGTCTTGTACCACCGCTGGCTTTGGGTATTTCTACTCTTCTTCC

AGGGGTAAATTTAAAGCTATTATCCTTAAGTTTCTCTATTATCCTTTCGATAACCTCCATAGACATACCATCCAATGGCG

TAGTTGGTGTGATACCCGCGGTCATGTTACCTGGGTTACTCTTCAGCTTATTATAAGCTAGTTCGTACAATGAAGGGTCG

TACATCAATCTATAAATCTTTTCAAGATTTACAGGAGTATCTGGGTTTTCATACAATGTTCTCTCAGTTTTAGAAGTTTC

GCACAAGTGTCAGATTTGCTCTGTACTGGACTCCCAGCACTAGTGGATAACTCTCTAATATTAAAATCTGGAATCCTTCC

CTTGTTCCTATTTTCATAGGACAGTATATATTTGCATATCCGGGTACTACGATTCCTCTGTTACCATAGCCGTTTCCAGC

CTTAGGCAATCTCGAAGTTCCTATATTTTGAATTAAAATTCCCTTAGATTGTCTACTCTTTACCTTAAAATTGTTTATGC

AATTCGTTTGCGGACGGACCGTACCCCACCCAAAAGTTCAAGGTGGGAAATCGCCGCACTCAACCTATTCCATACCCCTC

CGGGAGGGATCACAGGTAAGTTGAAGTCCATGGCTTGAGACAACTGGTAAATAGACTTCAAGCAGTATAGCCTTAATCAT

AAGAATCTTGACTAGGAGGCTACTCGACCTTCCTGATTAGGTCTAATCCCCATTACTGATTATCATGGATAAAGGCGAAC

CTTTACCCCTCACGATCTTTTATTGTCCGGGGTGGGAGGGAGGTACCAACTACCTCCCTGGCGACAAGTTATTATGGATA

AATTCCTGGTTACAGATACAATTAAATATTTATTTTTAGAAATATTTTTACTTCTTCTGTACGAATTTACCGTAGAAGTA

CTACAAAGTAAACTTTGGGCGGCTTTATTTGCTGTATCATAAATTACTTTAGTATTTGTTTCTAAATCAGTTACTTGTAC

CGGACTACCTAATTTATTACTTATTTTGGATTTAGTTTCCTCAGAATGAGATTTTCCAAAGAAATAAGGCTGCTTTTCAC

CTAAATGACTATCCCTCATTTTACGACGACTTTGTTCATTATGAGTACGACCTAAAATGTTTGGTTTGCTTTTCCGTACA

TGGGGTTTTTCCCCTCTTTGGCTTTCACTCATTTTAATTTAGTTTCATCAGTCATTATTTTACCTCTACGGCTTTCACTC

ATTTTAATTTAGTTTCTTCGGAATGTTTATAACCTAGCCAAGAACCTGCAACTTTTAGTAAATTATATTGGGGATTTAAT

AAATCCATATAATATTGCTCTATCTCTATGGCTTTTCTTTGCTAACATATTCAATAATGTCCAGTTGGAAATTAGCATAA

CCATATTTAAGTAAAGCTTTATTTATTGCGGTTTTCCCAGTCTAGGGTTTTCCAAATATCCCTTGCTAAAGTAATTTCGG

AATCTTATCGACAAATCTACCCCACTCCTATGTAACAGGATTTTACCATTCTTTTATTTGTTCAACGATATATGCCTGAT

TTTCCCTTATTTTCACTCATCCCTCCTAAAGGGGAGGAATGAAATAATTTGTGATTTAGCTAATTCCACATTGTGGTAGC

TAACTTCTACTTTACAGTGCTCTGGCCATCGAAAGAGTTGGTTGAATAATAGATTCGCCCTTTTACTAGAATGAGTAACA

TTACCTAGATAGATAGGCGCCAAAGGAGAGGTAGTAAAAGTTTAACTTTTATAATATTAAAACAACCAACCCCGGGTAAA

TTGTTTAGATCTGAGTTTGTATGATGATAATCATTCGGAAATGCTATGTTCAACATTGGGTTTCCCTATGCCTAATTCCG

TTGTCAGAAACACTTTCGGTCAGTGTTTAGAGATTTCTTCTCCATTTCAAAATAAAGCACAACGACGCACACTTTTAGCC

ATAGCACCAATTAATAAACATATACCAATAATAGTTACAATGTTTTCATTAACAAAAGGTGCTAGTGAGAATACAGTACT

ATAATCGATGTTCAAATATAATGTAGCCTTTCAACCATATTTTGGACTATATCTTTACCCTACCGTAGGGTATATAACGT

GTAGTCTCTGAGGATCCTCAATATTATTAAATAATATTTAATGTTTCCTGCGGATTGTCCATTTTCACTGGTTTAATTTA

CTTAAATAATATCCCGAACCAAACAAAGCTGATTTGGAATCCAACCTTATGAAAAATTAATCCCAAAAATTGGGTTTTAT

CCCCAGATCTATTCATTTTAATAATTAAGGTCTGTGTACGCATCTCCATGGATGTTTTCACAATATTCCTATAAAACGAC

TTTAGGAGTTTACCGTCATATAGTTATATAATATGAATGATATTACTATCACTCCCGGCAATTTATTTAAACTGTAAAGA

ATCCAAATGTGAAAAGTTATAAACATTTCGCTTACTATTAAAATTATTTTTATATTTTTAATCTTATATATTTTCTCCTT

GTTTAGACTTCCTCTGTGTAGATCTTGAACAAGACATCAATCTTTATACGCGAGATATTTAGACGAATATAAAGGGAAAC

GATAAAAATACTTACGTACGATCTCGTGACTTCTAGAATTATGAGCGATAGCCATAAAAGCATAATACAATTTATCCTCT

GCTTTTCTAGTTCTAGTATATAGACTGACAGTAAAAAGCTGCAATCTCACTTAAAATGTCAAAATAACTACCCCACCTTG

GTCTTGAGAAACCTTTCTAGAAGAATTCTGTTTAACTTCTATACGAAAAGAAGTTTGAACACTTGTTCTTAAAAACTCCA

TTTTTCTTTCGGTCGTATAACGTTATATTAAAATTCCCATCAGCATCTGCGAAGCCTGCCAATCAACTATTACTATCTAA

AGAGGACAAATCTAAACCTAGACAAGGTATAGAAGTACTATCTCGTTCATTTATTCAACGAATAGCTCTATTAAGTTAAG

TTAAGTTAAGTTAAGTTAAGTTAAGTTAAGTTAAGTTAAGTTAAGTTAAGTTAAGTTGTTAAGTTAAGTTAAGTTAAGTT

AAGTTAAGTTGTTAAGTTAAGTTAAGTTAAGTAAGCTTCTATATTAGGTGTCAAAATATAATAAGCTATGGCAGACCTAT

AAAAATAAAAAGAATATTACCTGGTTTCCTTATTCTTCGCATTAGTATACGACCCTACTTAAGACCAGAATATAAATTAT

TTTCTTCTTAAATTAATTCCCTCGTTTATTTCTTTAATCTCCTCTAAACCTTTAGAGGTAAGATGAGCTTTATTTTTATT

AAGTCAGCAACTTGACATCAATCTGCGAAATCTAAAGCCTTAACTCCAATCATAGGATACTTTTAAAAATGGAATTAGTT

TATTAGTCAAATTTTCAAAGTGAGTTATTTTAAAGTCTACCCTCTGCCTCCGGATCTAATATTAAGATTACCACAACCTA

AATATTCAATTAAACTTCTCATTAAAACCTCATCTCGAGAATGTTGAGCAATTGAAAACGCAAAACAACTTTTTCTCCTA

GATCGCTTAAGTTAAAGATTTACTTATTAAAATAATAAAACTACCTTCCGCATTTGTAAACCCCGAGAATCAATTAGGAT

CCTGGATTTGTTTATCTTTAAGTAAAGGCGCCGCCTTTTGACCGGAATAATCCCAGGGAAAGCTGCAGATTTTAATTCAT

CCGATAAACCACCATTTATTCATGGTTGCTTTTATTGTAACTAGTTTTTAAACCTTCGACCGTTAAATGTTCTTTTTACT

CAATTATATAAAACGCGATTTTAAATAGTTAAAAATCTCCTCACTTTTTAGTTATTAGAGGATATTTCTCAAAATGGCCT

AAATTACTACCCTAATATTATTAATACCTGTAACAGTAAAACAAATAGCATCTAAACCTTTGTTCTTTAGGTTTCCTACT

CCCCATTTTAATTGAAATAATTCTAATATAGCTCTATCTTTTTATGGACTTTAATTTGAAAATATAACTGAGCTGAATAA

CCAGCATTACATTTATTATTTTTAACTATAGATAACTGAAAACTTCCTTCACCATCCGTAAAACCAGTAAAAATCCGGCT

ATTTTATTTTCATCATAATTTATTTTAACTATTTGTGTGCAATAAGACCTAGGTTTAAAAGTAATTTCGAAATAGTGCAA

TAATTTCTTCTATTACCTAAACCTATTTCATTCCTCCTTTACGGGGAGGAAGTGAGGTAAATAAAACTTAGATTTTACAA

ACTTCACGCGCAGTAAATAAATTTTTATTTTTATTTTAAAGAAGCTAACAAAGCTTTGAATTGAGTAGAATTTTCTGCTT

GGCTGCTTCTATTAAGAAAAATTTTTCTCAAAGCTCAAAATCTAAAGAAACTTTACCTATTAAAGGGTATTTGCTAAAAT

ACTCTATAACCTTCTTTTGACTATTAGCTCCATAAGCTAAATTTAAAAATCTATACTTTGTATTAGATAAACGTATAAAT

CTAACCTTAAAACTAGTTTCTAAGTATTCGCTTATTTTACTATAAAGTAAAAATAATAAAACAATTCCTCTGACTCCTTA

CTGGTTTTAAGAACAATATTATCTGATAGTTAAAATTTTAATATAATTTTCAGATTATTATTTTTTTATTCTGAGTAGTC

ATAGAAAAAAGCCATTATTTTGTGAAAACCCTCCCAATCAAGCATTACTATCAATACTTGACTGATCTAAAGGTAAAGAT

TTTATATTCGTATCACTATTTTCATTATATCAATGTATAACACGAGATAATGCTTTTATTTTGCCTGTTCGTAAGTGTCC

ATTAATTAAATTGATTATCTTTAAAACTTCTTCTTTGGCTAAAATTTGCAATAAAACATGACCTGCCTTTTCTCTGTCAA

CAACTTTGCCTACTTTTAATTCAGTAGACAATTTTTCAGCCAAAGGTTTATCATTAATGTTAAAACAATAAGAATTTTTG

GCCGATAAACAACTTTAGTATTAGATTTAGGGGATCTTGAACACCTATATCCATCGCCTTCGATTAATCCTGCCAAATAT

GGCCCAAGCAAACCCCAATTTTCTTTGGCTAGACTTGTAGAATATCTTCTGACTTGATTATTTAAACCCACAAAACCTCT

GCTTTGTACGGCGGACCTCTTATATTTAAAATCCCGCCACCTAAATAACTTTCTTTTCTACACTTGGAAACTTGTAAAAT

TTTTGTCTTACCAAACGATCATAGTATAGCAAACATACCTATAGTTAAAAACAATCACCCACTCTGTTAGTTAAGAAAGC

TGACATAGAACTTTGATTTGCTGCTATTCTAGTAAATCAAAACTTACTAATAGATAAGAACAAACCCCTACACCTTCTCA

ACCCACAAACATTAATAAGAAATTATTTGCAGTTACTAGCACAATCATCATGAATGTGAATAAACTTAAATAACTAAAAA

CGCTGATTATGCTTTTAAGTCAAATTTATCATACAATTTGTCTAATATTTTATAAGGTTACTACTTGAAAGGCTTTTGTG

CCTTACTTAACCTCAAATTTTCTACCTCTATTCATACCTAATTTAATTTATCGAATGATATTCATACCCTTAACGGTTAG

ATGAGAACGATTAAGCATTAATTCATGAATCTTACACCAATCAAGATAATCTGAAGATTTTATACCAACTCGTTCCTGAG

GGGCCAGGAACTGGTCCCCGAGGTGGGAACATGATTTTCGTCCCCCGATGGGACTAAAGGATTTTCCTTAATTAAAGGAA

TTATTCTATTAGTGATATCGGAAAATCCACTATGCTTAAATGAACCGCAGACTTCGAATATTTATATATTTTCCCTGATC

CTAAATATTGAACTATCTTTTCCATTAATCCTAGATCTCTTATATGTTGGGTTATTCTAAATCTTAATTGTACTCGATAA

CCAATTAAACTATTTGATTGAGGAGTACGAACATCGAAGTTCCCCTCAGCACTGATAAAACCTTAAAGTAAAGAAGGGTT

TATAATTAGCCCACTTCGTAGGGGTCACAATTAATCACTGATCTTTCCACTGCTCGATATCCAGGAAACTCTGATTTTAA

TTTATCGGATAAACCTCAATTCATATTCCATACCCCTCCCCAATTTTATTCCCCAATTCACCCCCGATCCTTTGCGGAGC

ACGCTACCGCAAGGGGTGAGGCCCGGAGGGGAGATGCTTTGAGATTTACTATTTGATTTAAACCTTCGACAGTAAGATGA

GCTTTATTATTCATTCCTCCTGCCTCTGCCGAGGCAGGAGGGTGTAAGATTTATTATTTGTTTAAATAAAAGAAATCCCC

GGCTTTTTAGTTAATAATGGATATTTTTCTAAATGAACCTGAAGTTTATTTAAATCTTTAATTGAACTAATTGAGTAATT

AACAAACTCCCTTTTCGAGCTAAATAGATAGCACCAGCACCACCTAAAAAGTTGTAACTGGGCGGGATAATACATTATAA

TCCTTTCTATGTAAACCAAGTTGAAATTTTGGTTCAACTCCCCAACCCAACTTTAGAGTTTTATTTTTAACTAATATTAT

AGAAAAGAACCTTCACCGTCAATTAAACCAGACCAAACCCTAGGATTTATCTGAGTAGAGGTAGAATAATTCATATTTCC

CACTCTCCACTTACGTCCTAAGGGGACCCCTGGATTGGAGTGGGCGGGCTTATTTCATTGCCCGAGACGGTATTGCTTAG

ACGGGATTTTGATCACAATTGAGCTTCAACAGCCAAATATGTTGAAGCTCTGGTCTATACCACGGTTTCTTTCGGAACCT

TTTAGAATACATCTTAAACCCATTAATTGTTTAATAGATCAACTGCCGTATACTCGTTGCTCTTTTACAGCCATACTTTT

AGTATTATTCACTTAGAAAATAATACTTTAGACTTAGTCTTGGCTGACTTAGATCCGCGATTCCCCATTTCGTTTTCACC

TGGCTTTAGCCCAGCCCTGGCTCTGCCTTTGAACTAAAGCAGAGCCGAAGGGTCCTCCTATGGCTTGTTACCATCTCTGA

GTAATTACTTCAGCCACTCATAGTTTTTCAACTACAGCTAGGTACCATAGGCTTTAGGGGTTCCCGGAATTTGACAGTTT

ATCCCCATAGACGCGTTTTCCCATAACGCGCCAATCACTTTTGTAGCGAAACTAGTAAAAGGGATCTTAGGTATGTATTC

AGCTATGGAAACAATAGACTAAGCTAAGCCTGATTAGATCTCGAATCATAAAAGAAAGCAATAATAAATTATAGACCCAC

CCCTCTAATTTTTGGTATAAGGTGTAGGACATAGAACCAGAGAGGACAAAGAAAGCTTTTGGCCACTATTGCTACCTCTG

GCATATAGTATATAAAGGGTGTTAAACATTGCCCCGGAGCGAAGTAAAGCCCCCTTCACGGGAGTCCCTACGCATGTTTT

TTAGTCCCGCGGGCGGAACTTGTTCCGCACTCCAAGGACGGCCCCAATTGGTCCCCAATCATTCTCGGAGAGCGGAACCT

AGTACTAAAAACAATATAGAGAACACTTGAAGTTTACCTTTATTCCCTAGCAAACCCTACTCGCCTAAAGACTTATGCGT

AGCCAGGGAGGGCCGAACCTACTACTTTCTTTTGTGTAGACTTTTATTAGCATGAGGCCAGTAGTCTACATAATAAAAAG

GAAATCTTACCTCCTGAACTTGTGTACCGGGTAGGAGAACCTGGCCGGAGCTACTCCTATACCTGAAGAGGTTAACTAAG

TATGCTCTGCTGCATATCTCGTGACTTATTGACAGAAACGTATTTATTATCAAATAAAAAGGTTTACCGCCTCTTAACCT

TCGAGTTATTGTCATTGGATCTACCTTTAAGTACTTAGCACAATTAGCCGCTGAAGCAAAGGATTTAATAATTCCACCAT

TTTCATCTATAAGTTCCAATCTAATCTTAGCTTTATTTGAGTAATATTTATTTAAAGACTTAATGAAGATTCTTCCATCT

GCTTTACTTAAAAGTTGGATGGTCCCTCCAAAAGTTTGGTTATATTACTATTTAATATTGCCCTTTCTCCTACGGATGGT

TGAGACCCGCCGCCTAGCTCTGGTTAAGCGATTATTATTCATACCTCTTAAAATAGAATTAAAGACTTTAATCCTTCATC

AGTATAATGAAGACCTAATTTGCGTAATTTATAGATTGTTTTTCAATCTGGGTAATCCAATTCTTTCTTTGATTGTCAAA

TAAGACGATCAAAAAGGAATAAGTACTTGGGTTATATAATCTAAACGGTGAATAGCTAAAGAAATCATATTACTGCTTTT

TATAAGAAAGTGATACCTGCACCATCTAACCCTCGCCCTGCTTTAAGAGAGCACCCGGCTAATTTATGTAAGGGAGGTGC

CTACACAGGTTAGCTAGCTAAGTTATTTAAATAATCTCTAATCCCGAAGGGATTTCCTTTAGGAACCCTGACGGGTTCCC

CGCAAGGGAATAGGCCTCCATTAACACTAAATCTTTAGCTGACTGAACAATATTAAACGATAAAGCAAAGTTATTTCTTG

TATAAAAGATCCATCTCCTTCTACGAATCCTACCCTCCTCCCCTCTTGAGGGGAGGGAGGATAAATCCTAGTAATCAATA

AGGTGTTATCCTTATGGGGTAAGAAGATGGAATAGTAAAATCCAACTCTTAAGCTATTCATCCCCAACCTTCTTTTTCGA

CTTTGTTGAGAATATCCTGTGTTTTTAACCTAGAGCTTGTGTAGATTTAAAAGCTTTCTTGAAATCCCTAAAGTTATATC

CCCTCCTTCGGGGAGGGAGGGAAAGAAGTTTATGAGTTTTAAAGGATATTTACTAAATCATTAAAGAACGTTTTTCTAAT

GGGGTATCATTTGGGTTTATTATTACAAACCTGGCCGCCGGATCCAGTAATATATACCTTACCCATTGCCTAATGTGCTC

CAATGTATGAAGTGCCAACATCTTTAAATCATCAATGTGTAATTGAATTTTAAACTTAAACCTGTAACCATCCTGTCCTC

GAAAAGCATAAATGAGCCTTCACTGTCAGTTAAACCAGATAACCATTCGAAAAATCTTTAAGCTTAATTCTGGTCGATTT

GAATAATAACGCCTTGGAGTAAAAGGTTTAAGGCAACCCTAGTCTATGTTTATTGAGAGTAATACGTCTAAAATCCACCA

ATTTAGTTGCTTTGTTTTGAGGATCACTCGACATGTAACCAATCGAGTAGCAATGAACTAATGAACTAACAATTAAAACT

GGTATCAACATGGCCACAGTTAAACTATCAAAGTGGAAACCTCACAATACGTTAAGTGATTCACTATCTATTCATCTAAA

CAGATTTAGATAAACTGGTATGTTGTTTAAACCAACTTCAAAAAGGCTACTATTGCTAAGAAAGTTGTGAAAATTACACA

TAGAGATGTAATTAATTGTGCACCGTTAACTCCGACTTTTCTACCAAAAAGCCGGAAACTACTGATCCTAATAATGGTAA

AGTAATTATAGCTAAATACATTATTTATATTCTATACTGATACTTCCTCTTCGTTTTCCCCTTTACCTTAGTTTTATTTA

TTCCCTTAGGTTGGCTAGGGTATTCCCATAACTTTTCGGTTATGCCTGACTATACCTTAAGCAAATAATTTTTGTCCAAT

CTTTTCTTAAAACTTTTTTAAAGTCCCCTGAGTCGTATAGAATTATTTACCAACCTACATTTAGTCGATGAACTGCGCAC

CATCCGAACGAGCCGCAGTAGTTTTGGTGTTTGGCTGCGGATGATCTATTTCATTTCTTCATACAAATCGTTTTACCGTA

CAACGAGTCATTACCTGTTCCATTAATCACATTACTGTATTAACTTGGTAGATTTGTCTTTAAGACTTTCCCGCAATTTA

AAGGTTTTGCCATATTGAAGATATGACTAGGCAGAGCTTTACTCTACCTTTTGATCTGATTTCTCTGTGCCGTCTACAAC

ACTGTGATTATTCATAGCCTTATTCGAGTAAAGTATTGTAATTCTTCTTTTAATTGTAAAATTTATTATAAACCTAGCCC

CATCTTCATTCAAGTGCTGGTTATTTATTTTTAATAATATACGCCGCACTCTTAAAATCTATCGCCCTCCCTCCTTCGGG

GAGGGAGGGAAAGAAATTTAAGTGCTTTGACCCTAATATAGGGTTAGGTTTATCCAAAAAGGAATTATATGTTCAACTAT

GTGATCAATTTTTGTAATAATAAACTAAGCCATACCTAAACGTTTTTATAATTCATTACAAAGCAGCCACTTCCAAAGAA

ATTCACCAAGCTTTCTAATAATAAGAGATCTCTGGAATGTTGAGCTATTGAAAACGTAAAGAAGTAGATAAACCCCCACT

CTTATCGCCCTCCCTCCCCTTCGGGAGGGAGGGAAGTTTGAGATTTTTAACAGCAATAAAAAGTTGGATTCTCCTGTAGA

AAACCCGGCCCTACTCATTCCGGATGTAAGTTATTATTTTGAATTATGCTTTCCTTTAGTGTAACTGGAATAGTCATAGG

AATGCTTATTTTAAATATTTAGATAAACCACCTTCCTGTAGTATTAAGAGAGGCTCTAATATGGGGTGAACTATTTTTTG

TATACCTTCTAAAGTATTATGTTCTTTATTTAACATAAGTAAAACCATTTGTTTAAATAATAGATAGTCTTAATGCTTTG

GGGTAATTAAAGGGTAGTTATCGAAATGAGGAAGAATTACGTAAACTAAATCTTTTAAAGTACTAACTCTAAACTCTACG

CCTGAACTATTATTAAGTTTAGATACCTAACCTATAGCTCCGAAAAATTATGGAATTGAACTGATTAAAACTCTATCCTT

TTCAGGCATTTATTTGGAACTATTGCTTGAACGTTCCCCATCCTGTGTAATCCCTCCCTCCCCTTCGGAGGGAGGGAATC

TAGGATTTTTTTAATTTTTAAAACAAAAGAACTATCAGCATCGAAAAGTCCTGTAATAAATCCGGGATCCCCATTGAAGA

ATTTATAGCTTGATTAGTAGTATAGTTATTCTTAGCCATTGTAGAATAGCATCTGGCTATTCTTTTAAAGCTAAACTTGT

TTACGTTTTCGAATAAGTTGTATGGCCACGGGATAGACAGGATAAAAAAAGAGTTTAATCTGTAACACATCTTATATAAA

ACTACGCATTTAAATCCTCATTATCTAGACCATTATATCTTCCTTTATTCATACCTATTTTTATAAGACGTATTCGCTCT

AAACCTTAAGGGCTTAGATGATCTGGGTTTTGAATCATATCAGGCTATAAGGCATCTCCATCTTTAGAATCCTCTAATTT

TACACCTACAATCTTGTGTTTTAGAAAAAAGGTATTATAGTGGACATATTGTCTGCAAATTTATGACATTTAAAGGTGGT

TGAACTCCGATTTACTTAAGTAAAGTTTACCACAACCAAAAGAATCCTAAAACTTTTAATAATAGCTCATCTCTAATATG

TTTACTTATACTAAATTAGAATATTACTTGAAAACCTACTTTATAAGCAGAGTAGGATTTTTAATACCAACATAAAAACA

CCCCTTCACCAGATACAAATCCAGCCAGCCCCCACTCCGGATTAGGTCTTTTTATTGATAAGATAAGGTCTAATATAAGG

AACAATCTAGGGAAAGGCTCCCCTTGAAATTATCAGATAAACCTTTATTTAAAGAGGTTTTAATTGCTACAATATCTTTC

ACAAGCACCTTTTAGGGTTAGATGTTCTTTTCCCTTATATCCATAATAGCCGCTCTAAATAAAAGATCTATCCGGATCGC

TTGTTGAAAAATCCGTAATTAAAGGGTAACTGTAAAATGTGGTATTATTACATCCACAATATGTGCGAAGGAACCCTATC

TGTATGTAAGTTGTCCGAGGGTTTTTAAAAATATTACCATGACCTAAAAATACCCTGGGATGATCGGGGATAACTTGTAG

TAATTCTATATCCCTCTCATGAAGATCGATCTGAAAAGAGGTGGAATTAATCAGCCTGTACGATTTTTATCGTCTTTTAG

TATGTTAATCCTAAAACAACCCCCAATTCACCATCTGTAAATCCTGTAATGAACCAAGGGTCTAACCCTTGTTTTGTGGA

ATAGAGTCGAACATGCTTATTAGTTTGTGTATTTATAAATAAAGTTCTCCCCTTGTAATGGCCTAACTACGTTCCGTCCG

AACACGCCAGTAATCGAATAAAAATGACTTTTTCACAGGTGCAATAAGAGAATCTCGTGTTAATCTATAAACTAACTTAG

TTTTATTAGATAAGTTATCGTTACAATTTTACATTTACGTATTTGTCCCCTTCCGATACTTAAAACGTAGAAGTACTGCA

TGGTTACACAGCAGGACATTTAAGGGATCTGTGTTATATATAGGTCGCAATAGTAGAAGAGCTAGCTCACTTATACGTAT

GCTTCTAATTAAATTATATATACGCAGGGATTTGTAAAAGAAGTGTAAACCTTGTAACATTTACCTCCTAGGTGTTTGCA

TATATTCCTACACACCTCTTAGCCTATTCGCATAGGCTAATACTAAGTTGCGACTGTACATTCGCGAGTATTTAAGCTCT

ACGTAGGTGAACCAGTCTGTAGCGATGTTTATAGCCACCGACGGTCTTGACCTGGAAAGTTGTTAACCGTTTTCACCATG

TCCGGTCTTTAGATATTGGTTTTCCGCTATAACGTTAGAACAATTAAAGACTAGGTGGGATTCGCGCCCACTGCTCCTTA

AAATAAAATGCAACTAGAATACCTAAACCAATAGCAGACTCTGCTCCTGCAATAGCTATAATGTAGATAGCAAATGTTTG

GCCTAATACATCATCAAAGCTAAGTGAACTTACCAATATTAAGAATGTAATAGCTAATAGCATTATTTCAATCGAAATAA

GCATTAATATTATATTTTTCTATTTAATACAAATCCCAAGGTTCCGATTAAAAATAGTATTAAGGTTAAATTCATTGTAT

ATTTGTTTTTATAATAAGACCCTCCCGGATGAGGAGGGATTACCTTTTAGCTTGGGAGCTAGTTGAGGCATTCCGTATCC

CCCGGCGGCTACGCAGCCCCTAAAGCGCTATCCCTAGGGATAAGGATTAGAGCCGCCGGATTAGAGGGGATAGAGGCTTA

AGGCCTGGTGCCACCACCTTCTCTCATCCGCCCCTGAGGAAAAAAGAGTTATCTATGCCCTTATCCCTACCCCTAGCTAG

GCGCAATCACGTTGAGCCACGAACGAAAGTTGTGAAATAAGGCGGCTTGATGTGTAAAGACACTTATTAATTTCCCTACC

GACCTTTTGCCTTAGCTTTCCATATATGCTATATACAACAAGGAAAGTCTTTTGGAGGTTTCCCTCTGTTGTTTACTTAT

CCCCCTGCGGCTAGCTAGCTAGCTAGCAAAACCTGAGCCTCCCCTTAAAGGAAAAGGAGAGGAACCCTACCCTCCCTCCT

AAGCAGAGAAGGAGTCTGCTTAGCCTCCCCCAGGTTTCCTCACTCCTCCACCCCTTGCGGTATCCCTTATCCCTCCCCTC

TAGGACAGGACTCGACTCCCGGTATCCTGCGCTAAGGCCGGGATAACCGCTAAACAGGCCAGGCCCTAGTTTCCCCTGCG

TTGCTAAAACCCAACCCACCCCACTTTGAGGGACCCTGCAAACTTGTTGCTGGGCGTCCCCCAAGATCCCCATCGATCTC

CTCCGATCGGCGCAGGAGCCCTGTTATGACTCCTGCTTCGTCTATCCTCCCCAGCTTACGCTTAGCGAAAACCTGGACTA

CCCCTTTTTGCCTTGCATGGTCTAAGCAGTAAACCCCTGCCTTAAAACACCATGCTCCACAGGCAATAAACCTAAGGTCC

CCCTTTTTCGAGGCTGCGTAGCTAGGAGGCAGCATTTAGAAAAACCCTCCCTACCCTGCACCTCTGGATTCCCCCATAGC

TGCTGGAAGGAAAACTATCGCCACGGAGGGAGGAAGAAGTCCCAATCCTATCCCGCCTCTTGACGCCTGCTGCAATCCCC

TCTTTACCCTCCCCTCTTTACGCTATCCAAGGGGACCCCCTCTTTACCCGCCGCTCTAATCCCCTCAAATCCCCGATCGA

TCGATGATCCCCAATCAATCCAATCGATGAGCATCCCCGCCGGAGGAGAATCCCCCGATCGATTAAGGTAAGCATCCCCG

CGCTACGCAGCCCTCCCGGGTAAAGGGATAGGCGGCTGCTGCGTAAACCACCGATTGCAGCTGGCGTAAACGCCATAGGC

GCTCTCCCCGTAGAAGAGCCCGATTGCTGCTGGCGTAAAGAGGGATGGGTAAAGCTAAAGGAGAGGGATTAGAGCCGGGA

TAAGGGGTAGCTCCTAGGGAGCTACCCCCTTATCTAGGGTCCCCAGCCCTAGGCTTATCCCCTAGCCCCCTAGCCCCCGG

ATTAGCGTAGCGGGAGGAAGTCCCCTAAGCGATTAAGCATAAATATATAAGGATCCTCCTCGAAAGATGAGAATTTAAGC

ATTGGCTTTTTCTTCCTTGAAAAAGTCCCCTTAGCCACCACCTCCTAGTAATCCCCTGCTGCTAAGGGCGGATCGCTCCT

CCCCCTCGGCTAAATTTACCGAGGAACTAGTTTCCCCTGGCCGGTTCCTCCTCACGAAACCCCTGGGAGGACCCCCAGCA

AACAACCTGTTGCTCCGTCCCCCAAAACTAGTCCCCGATTCCCCACCGATCGATTCGGGAGGGACTTATCCCTTCCCTTC

CCAATCCCCGATCGAGAAATCCCCGATGATAATCGGCGGGATTGGGAAGGAGGGACTAGTTTGCAGGAAACCGAAACCAC

CCGTTTGTTTTCAAAACCAGAAGTTTTGAAAAACTCGTTCCTGAGGGCCCCAGGAACTGGTCCCGAGGGAAATTGGAAGT

CCCCCGACTAGTTCAAACCCCAAAGTTGCATCCTTCCTCTCAAAGGAGAGGAAGGGAGCCACGAAACCCGTTTTGTCCCT

CAAACCCCCGATAAATCGAGAGTTTGAGGAACGCAAAACTTTGTGGGACAACGACACCCCCGTTTGTTTTGGTCCCTAAA

ACTTTTGAGGGAGCCCACCCCGGCGGCGAAATGAAAACCACTCGTTCCTGAGGGACCTGGAACTGGTCCCGAGGGAAAAG

TCCCCCGATGATTATCGGGGACTAGTTTGCAGGAAACAAACTTGTTTGTTTTCAAAACCAGAAGTTTTAAAAACTCGTTC

CTGAGGGCCAGGAACCTGGTCCCGAGGTGGGAACATGATTTTCGTTCCCCGGGACTAGTTCCTGAGGGCCCTGAACTGGT

CCCGAGGTGGGAACATGATTTTCGTCCCCGATTGGACTAGTCCCTCCCCGGGAGGGACTTATCCCTTCCTTGCCAATCCC

CGAGAAATCGCAAAATCATCGGCGATCTAGCGCGGATTGGGAAGGGAGGGACTAGTTTGCAGGAAACACCAACTTGTTTG

TTTTCAAAACCAGAAGTTTTGAAAAACTAGTTCCTGAGGGCCAGGAACTGGTCCCGAGGAAAAGTCCCCGATTGGACTCC

AATAAATAGTTTGGCGTTCCTCTGCATCCCTCTCCGCAGCAGGAGAGGATGCCAAACCCCTTTGGATTTTAACTTCTGGT

TTTAAGGGCCAAAACAACAGGAGAGGAGTTTTCCCCGCAAAACTAAACTAGTCCCTCGGAGAGGACTTATCCCAATCCCT

CATTCTTCGTTCTTCGATCGGGGATACTAGTTTTCGTTCCTCTGCATCCCTCTCCGAAGGAGAGAACAGCACAGGATGCC

AAACCCCCGATCGAAAGGGGTGGGTTTTAACTCCTGTTGTTTTGAGGCCCCAAAAACCCCGATGAGAACACACCAATCAA

TAGAAGGTTTCCCCATTAGCCAAAATAACTAGTCCCCGATTCGGAGAGGGACTTATCCCAATCCCCGATGAATCGATCCC

CGATTCGAATCGCCCCGGATCCGGACTAGTTTGCAGGAAACAAACTTGTTTGTTTTAAAAACAAGTTTTAAAAACTCGGT

TTTCGTTCCTCACGAAACCCGTTTTGAATAGAATAGGGACAAAACCCACCTGTGTTTGTCCCCATATTGTAAAACACTAG

TCCTCTCCGAGGGACTTATCCCGCCGAATCCCCTGAATCGATCGGCGGGGATTGGGACTAGGTTCCCCCGATCGAATATG

GCGTACCCTTGGAGAGCGGAACAAGTTCCGCTTCCCTAATCCCCGATTGATTGGATTGATTGCGGGGATAGGGACTATTT

AATTTGGCAAGGGAATAAAAGATTTGAACTTTTATATACTTTCTTTTAATGTAAACATTAAAAATTAACCGAAAATCCCT

TATATTAAATATTTGAAAGATTTAAGAATATATCCTGCGGGTATCCCTATCTGGGGATTCCCCTTAGGGAATCCCCGAAG

ATAAGTATTATTACTTTACCAAAGGGGATATTAATTAACAGTAGTATCCCCTGCAGAGCGCCTAAGGCGGCCGGGATTAA

TTAAAAACGCCCTCCTAGGACCCTCCGATCGATCCACGGCGCTACGCACCCCGTCTTTTTCTAGGGCTATCCCCTTAGCT

GCACCTAATCCCCAATTGCTGCTAAGGCGGCCGGCGGGATAACCCCTAAAGCGCTATCCCCTTTGGCTGCGTAGCGGGCA

TTAGAGGATTAGAGGGGATACCGCTCCGCAGCCAAGCGGTATCCTTATCCCTCTAATCCCTCTGGCTTGCTTATCCCCTA

CGCGTAGGCTGCGTAGGGATTAGCTACCAGGCTAGGCCTAAACATGGATTGGATTACCCCTACCTGGCATAGTAGGTAGA

AAATCCGGGAGCCAGAGGGATTGGCTTTTCTAAGCTGTATCCTATGCCCTCTAATCCCTCTGGCTTATCCCCTACGCTGC

GCCTACGCAGCCGTAGGGATAAGCCTTCTTATCTAGCCTCCTAGCTAGCCTAATCCCCTTAGCAGCGCCCTGCGGGCAGC

CTAGGGATAAGCGCCCCTATGGCAGCGTAAGGTAGGGATTAGGGAGCCAAGTGCCCCGGCCGCATTATCCCCAATCGCTG

CAGGCTTATCCCCTGCTGGAGCTAGCTGCTTATCCCCTGCGCGTAAGGCTTAGGATTACCCTGCTGCGATCGGGGATTAG

CTAGCTCCTGCAGGGCCCGTACCCGAGCTCACCCTTCTAGGGATTAGAAGGCAAGGACAAAAAGAGAATCTTTATTGCAA

AACAGTCATGAGACAAATTTTTCAATAGTCACGGATTCTATAGCTATTGGCATAGAGCTGTGAAGAATTCCACATATTTC

GGAGCATTGTCCGAAAAATATTCCAGGTCTATTTATATATACTGATGCTTGATTTAACCTCAAATCTACCCTTTGCCCTG

TCTACTTATAGGGACGAGTTAGGGTACTTACCCCTGAATAAAGCTAATCTTTAAAACGTTACATTATCCATTTAACAGAT

AACTTCTATCTACACCTTGTGTATTTATTATATAGAGGGGCTTGCTATAGCCTCCCTCCCTAAAGGAGAGGGAGGGCTAG

ATCCTTATTAATTCGTAAATGATGATGATTATGATGACGATGATGGACCCTTACCAGTAATAGTCCCTGTAAATTATTCT

GGGTAATGAAACTCATCACGACCTTTATATAACTTAGGTCGTCGCTTCATGTATTTTTACATACCGGTTATCCGTAAGAG

CCGTTTCTACCTAAATACCTTGACATATCGGATACTGTATTAAATACTAAAGCACTATGATTTAATACATCTACTAAAAC

AATAGATCTAGATCTACTAGATTTTTTCGTTCACTTCCCCTTATGGGACCCCGACGGGTCCCATAGGGTCCCAAAGGGGA

CGGTAGAAACCCCAGCTATCCGTCCTATTATATCCGATTTTAAATCTGGGTGGTTCATGACAAAATAAACAGGTGTTTTT

CTTCCCCACTGCCACAGGCCTTAAAGATTAATATATCTACTTATATACCTCCTACTAGTTTTGTGGGCCCACGAAACCCG

TTGTCCCTCTGCGTCCTCTCCGAAGGAGAGAACAGCACAGGATGCCAAACCCCCGATCAATCCAAAGTTTGAACAAGTGA

TTTGAGGGAGCCACGAAATGAAAACCACTAGTTCCCTCTCCGGGGTCCCCGATTATCCCCGATTGAGAGCGGCGGAACAA

GTTCCGCCTAATCCCCAGAGGGCCGGGAGGACTGTGGGACAAACCTAGGTGGTTTCTTGTCCCTATTAAAACCAGAACCA

AACCCAAATCTCAAAATAAAAGCTATCGATTTTTATTTTTAATTTTGCTGCAAAGCTGCAGCAAAAGATTATTTTTATCC

CCCTCGGAGGGCCCCGATGGAGTTCGTTGAGGAACGCAAAACTATCCGATTTTCCATCCAGACTCTTGGCAGCTTGACTC

TGGATTATCGTACCACACCCAAAAGAGACTTTTGTCTAGTTTTCGTTCCTCACGAAACCCCGTTCAAACCCTAAGTTTTC

CTCTCCTTCGGAGAGGGAGGGATGCTGGCAGAGGGACAAAACCACCCGTTGGTCCCCACAAAACTCCAATAGTCCCCGAT

TCTCCCCGAGGGACTTATCCCAATCCCTCATTCGATTCGGGATCCGACTAGGTTTTCGTTCCTCTGCATCCCTCTCCGCT

GCTGGAGAGGATGCCGCCAAACCCCAATCGATCATCGATCCCAAAGGGATTTTAACTTCTGGTTTTGAGGCCCCAAAAAC

CCGAAGGTTTCCCCGCAAAACTAGGTTTTCGTTCCTCTGCATCCCTCTCCGAAGGAGAGGATGCCGCCAAACCCCAATCA

ATCGATCGATCCGATAAAAGGGGTTTGAACTGAACTTCTGGTTTTGAGGGCCCCCAAAACCCCCGTTTTCCCCATATTGC

AAAACACTAGTCCCTCGGAGAGGACTTATCCCAATCCCCAATCGATCTACATCTCCGATGATAATCGGCGGGATCGGACT

AGGTTTTCGTTCCTAAAACTTCTGGTTTTGAGGGGCAAAACCCCAATAAAAGGAAAGGTAGGGAAGTTTTCCCCATATTG

CTGCAAAACACTAGTAAAGAGAAAAAGTTTACCTTTTGCCAAAGCATGTAAATAAACTCCTGTTATTCCCATCACTCCTT

CTGTAGTAGTATAACTTTAAGATTTGAGGATAAAGGCTTAGAATGATCTATTAAGTAAACATCCATCTCTAAAACAGGGC

TATAAATTGTGTAATTTATCAAGTTAATGTACCTATCTAAAGTAGTTTTAGGGAATTACCTAATGAAACTGCCGCTCTGT

TCTTTGATGAAAATTTAATTAAAAATTGGGTATTATCTCCCACTCTTACTTCTATCCCTCACGGAATCCCCGTATCCCGG

AGGATCCGGAGGATAGATTTAGAACCATCATTCATTAGATAAGTACTTTTTCCCAATTACCTAGTCCCTCTCCGAGGGAC

TTATCCCAATCCCAATCGATGATCCCGATTGGGATTGGGACTAAAAGGAAATACAACAGTATAGCTCTAATTTAATTTGG

GTCTAAATTGAGTAAACAAAATTGTTCGTATAATCTAGCCTCCAATTGTGTCTGCTTATCGTAATATAAACAATGATTCT

AAGCTTAATGCGCATGTTAGGGATTTTCCCTTAGTCAAGTTGTTTATATAGTTATTAGTAATTATAAGAGGTCTTCAAAT

AAAATTATGCCTGCCGTGTTTTCGAACTGAAAGGTATAAAGAATTAAACCACCCTTTCAGGTCTGCTACTGTTAACCTTA

TGCTTTCAATCTCCCTTTATTCATACCCTCCTTAATTTAGCGAATCTTACTAATACCTTCATCAGTAAGATATCCTTTAA

TTTAACCCCCATTTCACATGCTTTACATCAATCCTTAAAATCTAGAGCTTTCCACTCCTATAATAAGATACTTAGAAAAG

AATGGAACGATCTTTTCTGTATATTAAGGAAATTTATGTACTTGATAACTAAAAGTTTCTCCATCTTTATAAACTTTACC

ACAATCTAAATAATCCCACCAGACACCCCTTCGGGGTCCCCTTCGGGGTGGACTTATTAATAACTGCTCATCACCCCGTG

TTGAGTAAGAGTAAATTTTAAATGTACTGTGTATCCAGATGAGTGTGCTTGTCTGAGATTTCCTGCCTAATGCTAATATT

AAAGGATCCTTCACCGGACGTTAAACCAGCGGCTGCTGCTCGCTCGCGGCTCGCGGCTCCGCTCGCTCGCTCGCGGCTCG

CGGCTCCGCTCGCGGCTCGCTGCGCTGCTCGCTCGCTCGCTCGCGCTGCTCGCTCGCGGCTGCTCGCTGCAGCTAGTAAA

GCCTGATCATATAGGGCCAGGGATTTTCTATTAGGAGTTGAGGTGCAGGGATAATATAAGGAAACGGCTATTTTGAACTC

ATCGGATAGACCTTTGTTTAAACTAGCTTTTAATGCTACAATCTGTGCCATTCCCTGAGTGGTAAAATGTTACCTGCTTT

CTATCATAAAAATTACTTCCCTGAACAAAAGATAATCGCTTGCTTCTGAGTGATTAAAGGGTACTTATCAAAATGATTTA

TAATCGACGAGAATTCCTTGAAGGATCTCCCTACCCCATTGAACACCAATCAATGTGAACCTTGGCTATAAATACTTCCT

ACACCGAAGAAAATTTTTATCTGTTCTAACACACCGATATCTTTCTTATAAACTTCCCCTTTTAAAGGGGAAGTATAATT

GAAAAAGGCTCAATCCTTAAACCTAAACGATTACTAGTGGATCTCCTGATATTAATCATGAATGATCCCTCCGCATCAGT

GAATCCAGTCAATAACCAGGGGTTAATAAGGCAAGACTTATTACTACTTACCGACGGCAGCCGGTACCTTTGTACAGGAG

TCTCCGGTAGGAAGTACTAAAAGGTCGGTCTAATGACCCCTGGTACATTTTGTGTTGATAATGAAGTAAGAGTATTTAAA

CAAACAGCCGATCCTACATAGTAATCCCAAGTCTTTAAACACAAAAGCATCCGCAACCTTACTTTCCTGTATACAAACTA

GGATGTCCTGGTTTATCCTGACCATCTACCCCAGAATCTCGAAAATCTCTTAGAGTTGTTCCCTTAGTATATTTATCTAA

GTACATTAAATGTAAGCAATTATTTTGTATATATAGTTTTACAACTGGATCGGTAAGACTGTTATACATTCTTAATAATA

CTTGAGAACATTCTTTGATATTAATAGTAGTGTACAAGTGTAGATTTTAAGACAATGAAGGAAAAGAATCCAAAAGACTA

TCCAAATTAGACCTCTTCTGTTGTTTAAGGGTCCCCCTTTTTGATTAAAAAAGGGCGATGATGAAGATGTACTAAACTCC

CTGCCCGTAGCCTAGAAAACCCAAGCCAGGCAGGGAACACACCTCCTATTAAAGGTAAAATACCCCCTTGTAATATTAGA

GTTTATCTAAGACTTAAACTCCTTCTCTGTAAGCTTAAAACGGAGACTTATTTATTTAATCTAAAATATATCCTTTTGCC

CTATAAAAGTGAATATATTTATAGGGACGAGTAAGGGATACCTTACCCCTAAATAAAGAGTGCATAAAAGAAGGTAGCAC

AAATTATCCATTTAACTAATAAACTAGACTTTTTTCTAAGTCTTATCTCAGGTGGATTTTTTTTTCACTTAATATCCCCT

GCCGCCTAAAGGCAGGGGATAGGGATGGGAAAGGTATTTAGTTCCTTCCTAAAAACCCAAACTAGCCCCCGATCGGCGCC

GGAAGGTTTCCCCACCGCGTGGGGAAGGAAGAGAGTCTAATATAATACATATCTTTTACGAATTTAAAGCTCCGCCACAG

GCTAAGCTTAGCGAGATAGAAATATTATCCTGTATCCTTTCGGATAGGGTTTGACTATATCTTAAGTTAATTAGCACCCC

CGCAAGCTTGCGGGAAGAGGTGTAAGCCCTCCGGGCTAGCAGATATTTAGTATATAATGCTAAAACCAACCGCCGTTTAG

TCGATGAACTGCCCACCTTTTGCGCAAGCGGCGCAAGCAACCTATCTTCCTTCCCCGGGAAGGAGCCAACCTATTTGCGG

CTTTTTGCAGCAAAATTAAAAATCCCTAATTTTGCGGCGGCTTATTTTGAGATTTTTACAAAAAGCAAAAAAGGATTTTT

GGGGATTTTTTATTTATTTATTAGTCCCTCCCTACGTAAGTTAAATTAGAGCTTTAATTTAGAGGGAAAGAAAGAGGAGC

CCCTTCGTGCTCCGGCCCTCCGGACCCTAAGTTGGTGCTTGCTTATCCGGTCCCTTTTATATAAAGAGGGACGGATCGAA

GGTGGTTGGCTGCGGATTACCTATTTCCTTTCGTGCATCTATTTCGATTTTACCATACCTCAATTCATTACGTGAGCCAT

CAGATATGTTAACACTCTTACTTGGTTGAAACAGCTTTAAGGCTTTCCCGCTATTTGACGGTTTAGTGTGAGGAGATCTT

ATTATTCTCTCCCACAACCAGGCGGCATATTAATATAATGGGGTTCTCAGCCCCACTTCTACCTGGATAGGCGTCACACT

TTATTCCTAAAGAAGGACAAGCGAAAGACGTACACAATTTTATCCTATTATTAAATTAACTCGACCTTACTCTATGTAGC

ATACTTTTAACACTCCTATTAACAAACTTAAAGAGACATAACCAACGGAATCACCATAATAGTTTATCATTACTAATACA

CTAGCCTAAAGGGAAGGTTTTATAAGATGGCCCTACCTATTCCACCCCAATCAATAAACCAACCCGATCAATAAACAGCT

TTTACTCTATTTTGTAAAAATATAACCCTTATAAGATATACCTGTATTCAGTATTTTAGCTATTTTATTTCTGTCCATTG

TAATGTTTTTAATTTTAAAATTTTATTCTGGAGATAATGCTGGAGAATTACAGAGTTTCTCCTGTTTCAACTTATTTTAT

GATAACCGAATGGCGAAATTTTTCTCTTGAAGAATTACTCAAATACTGCGTTTTCTTCTCAGCTATAAGACTAGCCCTAA

CTCTAAAGTCGTGAAATTAGCTAGACTTGCACCCTCTATAATCGGAGAGTATTTGTGATCTTAAATAAATTTAAGTAATT

TTTCCTTATTTAAGGCAATTATTACAAGTATTAGGGTGTATACCAAGATCTCCTGGAATTTTATTTAAAGATTTGGATGC

ATAGTATAGAACTTTTCCTTCTTCTAAATCATATAAATAAATAACTGTTCCTTGGTTAACCCTAAAATTGACTATTATTT

GAGTATTTAAGTTAAAACTTTCATGTAGGATAAGTTAGGGGTGCAAAGCCAAATTGATTCGGAATAAGAGTAAAGAAATT

AATTAGCTTATACCTGCGGCTAAACTCGGAATAACTTTATAACCCCTATAATTTGTAAAAGATTGTTAAACAATCCCCAC

ATTGAAGTGGAAAAGTTCAGGTAAACAATAATAAAAACTCTTTTAGTCAGATAAATCTAACCTAAAAGTCAACTAAGGTT

TAGATACCACTTTAACCAGAAGTTAATCTTCACCTTACGTATCTTCTACTTTTGGATATAAACTAATTTATTTTAAAATA

AAACCGCTTTCCCGATTTAAAATACCTCGAAGTAGTACTACTAACCAGACCTAAAAGGCCGCGGGCAGGATCTAGTTGAT

TTGAAACTATAAAGTACACAACCGGTATCTGCATCTATAACTTGAACAGCAAGGAGAAATTTTAAGAACGTAGGTAGCTA

GCGGGTAGATATTTGTTTAAATTTTTATATATTTTTACCATCCTTTATTTCAATATTAGAGGGTTCACTCAAAAGTAGGT

TAATTTAGCTATTCAACTGTACCCCTATTATTGACAGCAGTTGGTTGAGAAGTTTAAAGTCTATTCCGATTCATTTGATT

TAAGATGAGATTTAATATTTTTATCCCCTTCCGAATAATGATGGCCTCTATCCCTAAGATTAAGTACAGTTTTTCAATCA

ACATAATCCCTCTCTTTTTACTTTTTCAAGAAAGATTATCAAAGAATGGGATTATTACCTTTTAATTAACTCTTGCTGAG

TGATAACTAGTCTAATAATTGAAGATTTTCCAGGTATATCTTGCGTTTCTAATCTAATCGAATTTTTACTTATATTATTA

TGACCTATCTGAGAGAAATCCTAAAGAAAATCTCTTATTGATGCAATTAAATTAAAATCTTTAGAAGATTGAGTTATACT

AAAGATTGTCATATAATCTTTCTTTCTTACGGAAAAGCCAACCTTCTCCTTCCACAAAGCCTAACAATAAACTAGGAGTT

ATGCGAGGTTTATGCCCAGAAGGCATACTAAAGTTAGTTCTTTTAGAGTTCATTCCCTGTTTAATATTGATAATTTATCT

AAGACTGTGTTCACTTTTTATCTTACAAGTCTAAATTTAAAAGGCTTTTGGAAATATGGCAAATTAAGAAGTTTATGAGT

GTTTAGACCACAGTTATTAAATATCTCGAGAATTTTACTTTCTTCTGATATTTTATTTACTATAAAGTTCGCTGCCACAT

TCTTTTGGTTTCTACTCGACCTATAGCTAAAGTTTTTGTATAAACACTCGTTCCTGAGGGACCAGGAACTGGTCCCGAGG

GAACCCCTTTCAGTCCCCTCGGGACTAGTTTGCAGGAAACAAACTTGTTTGTTTTCAAAACAAGTTTTGAAAAACTAGTC

CCTCCCCGGAGGGACTTATCCCTTCCTTCCCTATCCGCGCCGATGAGCCGCGGATTGGGAAGGAGGACCTAGTTTGCAGG

AAACACCCCAACTTGTTGGTTTTCAAAACCAGAAGTTTTGAAAAACTAGTTCCCTCTCCTTGGGATCTCCGTCCCCCTTT

TCTTTTCTCGGGGAGAAATAACGGAGCAAACTTGATTTTTAATCCCGAATTCCTAAAAAGGCCCGAAAGGCAATGGAATA

TTCACCTTACGGATTCCCCGGAATAAAAGTATTTTTAAATTTTGGCTTTGCAAAATATAAAAATAAAAAGCCAAAACCCC

GAGGATTTGGTTTTACTTGATTTTTAATAAAATTTTTGAATTAAAAGAAAGGGGAGGAATAAAAATTCCCCAATCAATCG

ATGAACCCCCTTCGGGGAGTTTTTAATAAAAATTAAAACCCCTTCGGGAGTTTTTCCTGTCAATTTTGGCGGAGAAAAAT

TAAAAACCCCTTCGATCATCGGGGAGCCCTGAGGGTTTAAATACACAGGAAGAAACCCTCTCCCCTTCAATCATCGGGGA

GGAGGGTTTAAAACACAGGAAAGAAACCCTCTCCCGCCCTTCCGGGGAGCCCCAGAGGGTTTTTTACAGGAAAAACCCTC

TGCCGGCCCTGATCGGGGAGTTTCTTCCTGTGTATTTTGGCAGGCAGAGAAAAATTAAAACCCCTTAATCGGGGAGCCCG

AGGGTTTTAAACACAGGAAAGAAACCCTCTCCCCTTCATCGATCGGGGATCATCGGGGAGTTTGGGTATTTTGGCAGAGA

AAATTAAAACCCCCTTAATCGGGGAGCCCCGAGGGTTTTTACAGGAAAAACCCTCTGCCGCCCCTGATCGGGGAGCCGAG

GTTTTTTTAAAACCCTCCGGCCCGGGCCCGAAGGCGGGGAGAGGAACAAGTTACGCCCGGGACTAGCATATTTAAATCAT

CAATGTGTAGACCAATGGTAAAATTAAAAGAGTAATTATGGCCAGCCTTACTAATATAAAGCCAACCCTCCCCGTCAGTA

AGACCGGAGAATCACTCAATAAATTATTTACTGTAAAGATTACCCCTAAGGAGATGATCTTTAACTAGCTAAAGCTAGAC

GACATTGGAGAACCTTCTGAAGAAAAACATCTAAAGATTCAATTAGATTTAGAACAAATACCACGGATAGGCTTTACTTC

TAGAGAGGTGGAATTTTTAACAAGATAATTAGTTTTAACAGTTAGTCGTGCTAAAGCAAAAGACGTTCCCCACGTTGCAC

AAATTTATCCTATAATTAAAACATTCCTCCCTAAACTCATAAATAGTATTGCTCTAAAAATAAAAGTAATAAACCTCTCC

CCTGAAGGGAGGGTTTTAACCTGAAGAAAAATTTTAAGGCATTACAAAACCTCAAGGTTAAATTTATCAAATCCTTCCTT

TTTATTAAAGGTCGGAGTAGGAGTTTCCCTGAGTGAGAGATCTGGATTTAAATGCTTGAAAGTAAAGTACTGGTCTAGTC

TTCTAGAAAGACTATTACTAGAACCCACGTATTTTCTCCGGTTAACCTTATGGGTGAAAATATAAACACCTTAAGCCTGC

TGCTTTTAATCCTCTAGTCCCGCAGGAATCGCCGAACTGGTTCCGCCGCCGGCTCTCCAAGGGACTCCACCAATTATTGT

CCCCCGATCGGAGAGGGAACTTTTCGTTCCTCACGAAACCCCCGTTCAAACCCCAAAGGTTGCATCCTTCCTCTCCAAAG

GAGAGGAAGGGATGCCAGAGGGACCAAAACAAGTTTGTCCTAAAACTAGTGGTTTTCGTTCCGCCTCAAAACTAAATCCC

CTATTTGACAGGACAGGACAGGAGGGATTCCCAATTCACCCCTATCCCCATCGGGGGTGAGGCCCGGGGATAAAAAGAGA

TTTTTAAAATAAATAAGAATTTATTTGAAAATTAAAAAGAAAAAAATTGGATTTTTCTTCTGATTTCTTTTAGATTTATA

TTTTGGCTGCGAAAGCAGCCAAAATTAAAAGCCCCAAAATAAAAAAAATTTTTTTTTATTTGGCTTTTTTATATTTTTGC

AAAAGATATTTTTGCAAAAATTAAAAAATAAAAAATTATTTTCGTGTTATTTTGAGATTTTTTGTTCAAACTTTGTTTGT

CCTTCCTCTCCAAAGGAGAGGAAGGGACGCCAGAGGGACAAAAAGTGAAGTTCCGTTGTCCCACAAAACTCCAATAGTCC

CCAATGATCGGAGGCGAACTGGTTCCTCCCCGTCAATCCGGGGATAAGATCCCCTCGGAGAGGGAACCTAGTTTGCGTTC

CTCTGCATCCCTCTCCGAAGGAGAGAACAGCAGCAGGATGCCAAACCCCCGATCCTTTGGGGTTTGAACTTATTGTTCAA

ACTTTGTTTGCATCGTCCTCTGCCTTCGGAGAGGACGACGCCAGAGGGACAAAAGTTTAGTGGCCGCAAAACTAGTCCCC

CAATTATCCCCAATCTCAATCGGGGATTGAAAAGAAAAAGGAGGCCCAACTTGTTCCGCCGGCTCTCAATCGGGGATAAG

ATCCCTGAGAGGGAACCCTAGTGTTCCTCAAAACTTATGGTTTTGAGGGACAACAAAAGAGTTAATTTCCGTTGTCCTCC

TCCATTTGCAAAACCCAAAACACTAGTCCCCAATTATCCCCAATTATTGCCTCCGGATTTAGGAGGCGCAACTTATTCTT

CACTTTTTTTAATTAAAAAAAAGGGGCGGGGATAAGATCCCTGAGAGGGAACTATAAAAGTAATTGTTTAGAAAAATGAA

TAAACATTTTATTAATGAAATCCTTATTGGTAATGCTTAGGATAATCATATCTAATTGATTACCCCTTCTCTACTTTTAA

AATAGTAGTTTTCTTGACGATATTTTTATGCCTTTCCACCTGATAAATTCTTAAAAAGTTTATTATTTCTTCCATTAATT

ATTGATCTCTATTAGCTTGACTAAGTCTAGAAATTAAAGTGTAATTTACTGATCTATCTATAACAAAGGAACCGTCACCT

TCAGTAAAACCTAGTAATCATGTCTAGTAATTTTTTGTATAGCCTGGTACCGGATGATAATCCGTTCTTTTGGAATTCAT

GCTATTTCTTATAATTTAAATTTTTGGCTTTTCACTCACTAGTCCCCGCAGGAATCGCCGGCAACTTGTTCCCCTCTCAA

TCGGGGATAAGATCCCCTATCGGGATCGGGGAGAATCGGGGAACTTGTGGTTTTCGTGGCTCCCTCACGAAACCCCGTTC

AAACCCCAAGGGTTTGCATCCTTCCTCTCCAAAGGAGAGGAAGGGATGCCCCAGAGGGACCAAAACAAGTTTGTCCTCAA

AACTAGGGTTTTGGTGGCTCCCTCACGAAACCCTCTGACTGACTGGTTTTGAGGGACAAAACCAGTTTGTCCTCAAAACT

AGGGTTTTGGTGGCTCCCTCACGAAACCCTCTGACTGACTGGTTTTGAGGGACAAAACGGGTTTCGTGGCCTCAAAACTA

GGGTTTTGGTGGCTCCCTCACGAAACCCTCTGACTGACTGGTTTTGAGGGACAAAACCACCAAAAGTTGTTGTCCTCAAA

ACTAGTTTTTCGTTCCTCAACGAACGGGTTTCTTCTGGTTTTGAGGGACAAACACGGGTTTCGTGGCCTCAAAACTAGTC

CCCAATTATCCCCAATTATTGCCTCCGGATTTAGGAAGAAACTTGTTCCGGCTCTCCCCTTTCTTTTCCCTCGGGAAATA

ACTTTTATTTGATTTTTAATCCCCGGAGGGATTCCTTTAGGAATCCCTCAGGGATTCCCCGAAGGGAATAAAAGTATTTT

AATTAAAAAGCCCGACTTCTATGAAAAATCCCGTCCCAAAAAAGCCAATTTTTGGCTTTTTGATTTGGAGAGTTTGAACT

TGTGGTTTTGATGAGGGACCAAAACAAGTTTGTCCTCAAAACGAGTTTTACAGAGATTTAGTAACCTTATTTAAATTTTA

GAAAGTTCCAGATGTTTTGTCGAATTTAAGGGGTATCTATCAAATATGTTTATAATTAGTTAAACTTATTTTCGTTTTAC

CACTCTAAATTCTGCACTATTTTTAGTAGTTGTTACAGATCCTATGGTTAAAATTTTTTAATCCGATGTAACACTTCAAT

GTCATCCAAATGCAACTTAATTTTAAAGCAAAAAGATTTTACTTATGTAAAACAACCTGATGCATCTTAAAATCCTCGAA

ATAAATCCATAAAACCTCTGGATCTTTATAACCTTTACTAAAAGTACTATGAGGGAGTACTGAGCAATAAGGAAAGAGAA

TTGTAAAAGTATGGCCGAGAGACAATTTATTTTGGAATTACCTCCACCCCGTAGGTTTAGTTATTAGTGGTAAATCAAAC

TTAACACCAGGTATATCTTTTAATCTATCAAGTTATAGCTGCGTAATTTAAACTTGCGGATTTGCTAAGAATTTATTAAG

GATTTCACTAGTTATATTATTATATTCATCATAAAGTCTAATACTTCCCTTATTCGATATAGTGGACCCCATAGCTCCCC

GACCCTCTTTCGGATTGGGTAGAATAATACCTAAAAGACTTATTTAAATTAATAGAAGTCTTGTAAAGCTTTCTTTCTAT

AGAGGTAGGTCTGAGGGATGTGAAATAAAAAGAATAATTGCGGCTCTTTACACTTGGGTATCATGAAAGACGAACCCAAC

ACAATTTTATATTATTTTTAACTTCAACCATAAAAATCCATCTTTAAATAACTCAAGTTAAAGCCTGCACTTCTAGACTT

CTTATTTCTATCCTAGGTTACATCACAAAAATATGCGTATATGTGAGTATGCAAATCTAGAGAAAGTTTTAATAACTTAG

ATTAGGCTATTGTGTCAACTTTAATCTGGGGTTATTTAACATGAATTTCCCCTGAACCGGTTTACCGGCGACTAGAGTAT

ACCTTACAGTATTGGATAATAAAATATCAAACTGAATAACCATCTACTCGTTGCTCTTTTACACATGCAAAAGAATTGTT

AAGCAATTTAAACCTTTTCACAGAGGTGATTTAGATCCGCGATTACCCATTCTCTTTTCAGATAATCATTAGTATTCTTA

CTATACCTGAGTAATTAGTTCAGCCGCACCGTGACCCTTTTAGGTCTAATGTTTAGTAACTAATGTTTTAGGGGCTTCCC

GGAGTTTGGTTATTGAACACTAAAGTGAGGGCCTAATCTCACTTATTATGTATAACATCCGCTGCAGTAACAATAAATCT

AACATGAGTTTGTTCAGGAAGTATTACTCGATTATCTACTTCTAGTAATCTTAATGAACCATTCTCTAGATCTGATTCTG

GAACTAAATATGAATCATAATCTATAAAGTCATCCTCCTTGTTTAAGAAATCTGGATAACTGTAACTTCAGTATCACTGG

TGACCCTCTGCTAAAACTGACATTGATGGATCACTCACTTCCAATTATATTAATTATAAAAGTAAATATGTTCACCGATA

TCTATAAGTTAATCAAATTCATATATCGTTTTATTGTACTAACCCTTTTCTTTTTAAAAAACCCACCCGAATTATGCAGT

GCTCCACTAAGATACTTCTAGCCCCTTTTTAAACATGAACATGAAGTAAATACCCCTAAGCTTGCCGCTGGTTAGCAAGT

AGAAAAAAGAAACCCAAAAGTTTGGTTTATATTTTTCCCTTTTGGTGCATTTTACCCTTTTGGCTAAAATAAAACCCCCA

AAACCAGAAAAACATAGTAAAATTTTTATCTCTTCGGCTTTTTAGCCGAAGGGTAAACACCCTGAGAGTGCAGCACTTAA

AGGGGCCCTTGGTCCTGGTTGAAGGGCAGGTAGATAAAAAATTTTTTATCCCATCCCACCCTCTGCACCACTGCAGAGGG

TCAGGATAGAATCTTACGTGGAGCCGCTAAAATTCGCCATAATCCACCAAAGAAAGGGGATAGGCTTTAAAAAGGTACAA

AAGTATATATTAAGACATAACTTCTTATATTGCCTAATTTTCATGAAGACACTTTTTATATAAACTTTTTATTATAAAAT

AAAAGTATATTACCCTTGCCTTTATTATTCTTTTAGAAACTGTACTAGGTTTCACATTTAAAAATTTTGCACAGTCCGCA

AGAGAATCGAATGAATGTAAATTATTACCTTTTTCATCCACTATACCCACACAAATGTTTTACGAGAAGAATTATAATAT

TTATTTAAAGATACTACCCATTTTCTGCCATTTCTTAACTCGAAGTTTGAAGGCCTAGAAGTAATAGGTTAACTTGATCT

AGTACTTGTGCTCTATCTGCAATAGGTTGCTCGAACTTGTAGAAAGCCTATTCCTATTCATTTTACTTAAAATTAAATCT

ATTACTTTAGTACCTTTAATCGAGAGGTGATGACCTGGTTCTCTCAACCTTAGTATATTCTTTCAATCTTGGAAATCTAA

CTGTTTTTACTCAGTCAATTTAGACTATCCAAGAAAGGAATAAATATATCCGTAATAAATGCAATACGCTAGTCTCAATT

CTAATAACAGATTGTTGATTAGGGTTCTTAGATACAACCGAATAAATTCCAATAGCTCCTGCATAATTACCATTTGTGTT

TGGCAGATTTTCTAGATAAACCTGAATTTTTGATTAATTCAAGATTAGAATAAGTCTGAGTCAAACTGAAGTCTAATCTA

TAATTATTTCCTCTATTTATACTAAAACAACCTACTCCTTCTACAAACCCTAATCCCTCCTCCTCCTCCCTCGAAGAGGA

GGAGGAGGAGGAAATAGTCAATAAGCAGTAATATTAATAGAATTATCTTTAGCCATTATATAATCTAATCTTGAATGATT

CATACCTTGTTTAATTTTTAAAATTTATTTAAAAGTATCTGCCCCTCTATCCGGTTTAGTATAAAGCAGCGCATAAGCTT

TAGAAAAATCTTTATAGTTGAGTCACTTTGAACCTTTAAGAGGATAGTGCGAAGAAATTTAAAGCAGTTGAGCTATATCT

TTAAGTTTGGTTACAGTATAAGATGCAAAATTCTTGTATGATCTAACTTCCCCAAAACCTAGCCGATTTCTGAATATAAT

GTAACACATTGATATCATCTTTGTGTAAGTTCATCTGGAATCTAAAAGCACAAATTGTACCAATATGTATATAAAAATTC

CCCTCAGCGTCCGTAAAACCACTAAATCATTCATAAAAATTATTCAGGTTATCCGTATTAATAATCCCTCTTTCCCTGCT

TGCAGGGTATCTCTCATTGCGGCGGATAATGTGCTAAATCCACCTTTTTCCAATAAAATAATAGGCTTAAATGTAGTAAA

ATGACTCTTACCACGAATTAACCCTGATTGTACAATATATGTTATGGACCTTGTATAAACAACAGGAGTTGTGACCCTAA

AACTGCCTTTTTTTATAAATAGGAAAAAAAGGAGAATTTGAATAATTGTCTTATAAATAAACAAGTGTAATTTTTATTTT

TGGATTACCCTTCAAGCATTCCATAAGATATGCTGACACTGTAAAGCTCTCATTGCTATTAAATATATTTAAGAATTATT

CTTCCATATCTCACGATATGGTTTAGACTATGTCATCATTAATAAGATTTATAATCAGCCTCCTCCCCTCAGATGGAAAG

GAAAGCTTTGCGACCCGTCCTAGGTATCTACCTATAAGTGAGAACTTGCCTAACATTATTCATTAGGCGTTCGCTGTAAA

GGGAGACAAAAATTTTAGGGATATTTAAATAAATCCCTCCACTTCGTATGGCCCAGCTCCAGCAGATTAAAAAAATAAGC

GATGGGCGCCCGGCTACTAATCTGATTAACGGAGGATACTCTATACAACTTATTATTATTTTAGTGCTAGGAAGATATTT

ATCGTAGATAATTTTATCTTCTAGTCGTTGAACGCTCTTGTTTATCATTGATATTAACCAAAGGCCCAGCTTTTTTGACC

CTAGGTAAAGACCGGGCTCTTTTTACAGGAAAAACCCAATTTTTTATTAATCTACGATACCCGACAGGGTATCCTAATTA

ATTACAATAAAATAAGGCCGAGGATCATCCCTCCTCTTATTTATATAGGAGGGACGATCTACTTTAAACAAGCTCCGCTT

CAAGTAAATTTAAATTATATTTAATTCTTCTTGAAATTCATCCTCTTTAACCTAACAATCTTAAGTTGTTAGCTGGACTT

AAGTGAGACTTAATCCATAAGGTACAACAATAGGGTCAGCCGAAACTGCCCTCCGAACCGTACGTGATAGTTTCCCATCA

TACGGCTCTCCACCTAATTACATAGACTCCATGTTTGAAACCATTTGAGATAAATATACTAATCTTTGAAATCAATAATA

CGTCTTAGATCTTGATAGTTTAATGCCCCGCCGTTATGGAAGCATTTGTGATGATATGCACATAGTGGAACTTGTTTTCT

ATTTACGCTACCTTTCCACTCTTCAAAGGTTCGGTTCCCTCCCCGTCATTTGGCTCTCGCATCATCTTTAACAGCTCTGA

TGTGATGCATTTCGATGTTAGTTTTAGATCCACAAATTAGGCAAGATTTAAATAAGTTAGAAGCGGTGGTTTTCGCTCCT

CATGACCCTGGGGATAATTTCGGTGATATCGTTCAAGTTAGATTTCTGGTTAAAATCTAGTGTCTGTTTAAAAGTATTGG

GGATCTTTAATCCGAAATTATCTTCCGATGCGAGGTCTTTACCGAATTTTGTAAATGTAGCTCTAACGGTTCTTAGTTTG

TATTTCAAGGCAAAGGTTGCCGCACAAGATGCCCTTAAATATCAGCAGATTCTATGTAATTCGTATCTATTTCTTGCGAA

CCCGTAATGCGCAATTATACCATTAATTTTGGAGTTATAAAATCAGATGATGTCGTTGTGATCTAGGTTAACTAAGTCTC

GTCTTGCTGTAAATCCTATGACGCCTAATTTGCTTCTTTTATAAACCCACCTGAGAGTAATTTCTGGATAAGTACGTCCA

TAGGTAGATCTACTCTCATTCTCGGGACGGTTCTTTTTCTCATATTGGTTCCCACGATACGGGACAACTTCTTACTGTTT

TAATTCTCCTACATTTTGCTCCCAGAAATATAAACGGTTCCGTAGTGGGTGAGATGAGGGATTTCTCCTCATTTAATTCT

AGACCTGTGTGTTTGATTAGATAGTCTTTCACGTGTCTTCTTATTCGCAAGGTGTCTTGGTAAGATCCTGTCACTAATAT

TACGAAGTCGTCGGCGTATCTAAGATATAATAATCTTTTGTACGCAGGATCTGACCTGTTTACTGTTTCGGTAATAGTCA

TCTCCTTAAATAGCGCGATCTTCTCTTGAGGGTCCTTAGTATAACTACGTCTGTTTCTAAGAGATATATATTTAGGATTT

TTCGCTCTGTTCTTACCGCTGTCGAATTTTATTTTGTATGTTGCCATGAATTTGTCCAAATCGTCTAGGACAACGTTTGC

TAAAACGGGGCTGGCAACATTTCCTTGAGGAGTTCCTGTTTCTTTATGGGTAACTATTTTTGTGGATACATCGACCGTTC

CACATTTTAGCATCTTATTTATTAGTCTAAGGAATGGTTCGCAGTCGATCTCTTTCTTTAGTGCCTTTATTATATTTGTA

TGAGGGATCATATCGAAACATTTAGATATGTCCCCTTGGATGACTCAGGCGTAGTCCCCACCTTGCATCATTAATACCTG

TAAGGCGGATTTTTCCGACCTATTCGGTCTGAAACCGTGACTGGTATTGCTGAACTTTGGCTCTCAGATTGCTCTGACGA

TTAACTCGAGGGCTTTTGGACGATCTTCTCCCTCGGCCCATTGATTCCCAACATTCTTACTTTACCATTAGGTTTGGGGA

TTTCTTGAGTTCTACTTGGGTTAAATTGATATTTCCCATTTCTCAATTCATCGGCTGTTTTGTAGAACCATTCCTTACTT

ATACCATCTAAGGTTAATTGATTGATATCTGCACCCGGAGTCATATTTCCTTTACCTTTTATAATTTCATAACACGCTAT

CAAGAAGAGTGGATTGGAAAGAATATTGTGAATTTTAGTATATTTACCTCTCATATTTCTACAAGAGTCTAACTCCCTCT

TATTCAAGCTCCCACCGAGATAGCTTTACGTACTTTGGATTTCGGTTCCACTAAGTCTTTAGTGTGAGATGAATGAATAG

GACATGAGTGAATTAAGCTACCTCCCTTCGCTTTGTTTGTAGCTACTATGGAGATTCTGTCTCCGCATACTTTCGCATAG

GCGGTTCGATCCCGTGGTGACTTATTAGATGACGAACAACAACAAGATGTCTCGTTGTCGTCTTTGAATGAGTTTAGCCC

CTCGCTCATACTACTTGCGTAACTGTCATAACAGCGACCATATCCTTCGGGGTACCATTATAGCTTAAACTATCTATAAC

AGCTAATTGGATATCATAGACCGACTTATTCTGCTTAGGATCTATGGACCTTCTATATTCGGTCAATGAGTAAATCGAGT

TCGTTATCCTAGGCATGCTCCTCCCACTGATCCCTTCAGTCTGGGGCAGCCCAGATCAAGTTGGACTTTTAATATCTGCT

ATACTGCCTATAATCTTGTTAGGTTTTACAGGAGCGATATATAGTGGTCAACTACGTTGTATTACAGTCGATAATATTGA

TAATAAAACGCCCCTTGTAGTTGAGAGGATTGTTCCTCGCATCATAATGGTCTCTCACGGCGCACGTTTAAAAGAAGGGA

AAGCAATTAAGATTAAAATTAAAGCTGGTGTAATTGTCCAGATTAATTCGATAGTTGTTCCGTGGTTTAAATATTTGTGA

GAGATTGGTGATTTTGGTTACTATAGTTTCTTATTATGGATAACATAATTCAAGCTACACCAAACAATATTATAACCAAA

TAAAACATAATATTATCATGTAATTCTACTAATCCTTCCATTTGCAAAAAAATCAAATTCAACGTTCAATTAGTTAAAAT

AATGTTATTTAATAATAGCCTGCCTATTCCTAGGGCTAATACATAGCGGGCTTGTATTAAACTCTACCTGTATTCATACT

AGTTTTGCGGGGCACGAAACCCGTGTTTTGCCCCTCAAAACAAGTTTTGAGGGACGAAAACTAGCTTTAATATGAATAAT

TTGGTCTAATCCTTCTTTATTATAATAAGCTTTACTTTTAATTAATTCTGATATAGAGCAGAAGTCTGGAAAATCTCAAG

CTTTTATTCCTAAGATTCCTCCCTCCCCGAAGGAGGCCCGATAGGATATTTAATAAAAAGGGATAATTCTTTCAGTTAAG

TAAGATCTTTTGAATACTTTGTACACTACTGCATTTTAAGAGTGTTTATATACTTTACCACATCCTAAATATTCAGCTAT

GTAAGCTATTAATTGTTTATCTCGGACATGTTGATTGATTTGGAAGATCAATTCGATAAAAGCTTTAGAGTTACCATCAC

GATTTCTAACCTTAACACCAAATGATCCCTCTCCACTAGTAAACCCTGCCAGTCATTGAGGATTGCTAATATAAATCTTT

TCTTTTCTTGGTCTTATAGCAAATGTAACATCAGAGAAAGCTTTTTAAAGGTTCAGATAAACCATTATTTATAGATGCTT

TTATTGATATAATTTTACGTAAACCTTAAGGTGTGAAATGTTATCGGTTAATTAGTAATTGGTAAGCTGGTTTAAATAAA

AATTATTTCATCCCCGTATTTTTGTGAAATAAGAGGAAATTTATCAAAGTGATCTAAAACTACTTTTAACCCTTTGATAG

AGAATACTCTGTATTCTACTGAACCAACATTTGTAACATATATTTTACCAACACCAAGAAAATTTTGAATAGCCTCCAAG

ACAAAGAAATCTTTATTATGTAAATGTATTTGAAATCTTGGTTTGATTACTCAACCTGATTTAACATTATTACTTTTGTA

ATACTTAAAACAAATGAACTTTCTGCATCTGCAAACCCTGTTAAGAAATGAGGATCTATTATTAAATCATGATTATTATT

AGCTCTACTATTCTTAGTGAACGATGTAGAATAACTTCTTTTATTAACTAATTGAGTAGTTAGGATTTTGTTCTGATAAT

TTCTTTCGAAACCCATTAGAGTACATCTTAAATGTGTGGGGTAACAGCCCTAAATTATAGCTTCCCCTGCACACCAACTA

CCATTTACTCGTTGCTCTTTTACAGCAATATAGAACTTTAGCCCACATATTACTGACTTAGATCCACGATTACCCACGTT

CTTTTCAGAAGCCTTCAAGCTTGTTACCTTACATGAGTAATTAGTTCATCCACTTATACCCTTTCGAGTATAGCTTGGTA

CTTTAAGTTCTAGGGATTTTCCGGAGTTTGGTTGTTTAATCCCCATTAATGATTTCCCAGATCATTTAGGAAGAGGAGTA

GCACTATCCTGAAAATATAGTCCTCAAGGTCTTGGTGTATCGCAGATATTAAAGCTAATCAGGTGGTATAGAAGGTTCAT

AAAATAACTCATATATTTATTTTCTTGGATTTACATTATTTTTATCCCTCTTTCCCAAACATCCCTCTTTTATATTGCAT

GTATATATATATTATTCCCCTAAAAAATCTCAAAATAAAAGCTATCGATTTTTATTTTTAATTTTGCTGCAAAGCTGCTG

CAGCAAAAGATATTTCCCTACTAATAGTCCCCGATAAATCCCCGATTGATACCCCCGATTAGGGAAGCGGCAACTAAGGG

TTCCCTCGAGGGAGGGAGCCCTCCCTCGAGGGAATTTTAGTTCCTAAAACTGCAGGGCGAGGGACAAAACGAGTGCTGGT

TCCACAAAACCCCCGTCCCTATCCCCGCGTAAGGCCGGATTCGGAAGCCGCGGAACTAAGGGTTCCTCGAGCCGGAGGGG

ACCCTCCCTCCCTCGAGCAACCGGGGTTTTGGGGACCCCCTGCACTGGCGCTGCGTCCCTCAAAGTGCTCGGAGGAACGA

AAACTAGTCTTTAGTTCCTCCCCTGCTACGGGCTTTGAGGGAAAAGTTGCGGGTTCCACAAAACCCCCGTCCCTATCCCC

GCCGGCTGCTGCTAAGCCCGGAGGAGCCTGAAGCAGCCGCCAACTTGTTCCGCTCGAGGGAGGCACCCTCCCCTCCGGCT

CGGAACTAGTTTTGTGGGACAAAACTATTTTGTCCCTCACGGAGTTCGTGAGGAACGAAAACACTAGTTCCCTCTCTGGG

ACTTATCCCCATTGAGAGCGGAAGAAGTTCCTCCCCTTAATCCCCAGAATCAATCGGGGATAGGAGGAGTATCCCCGCGC

GCCAGGGCGCGGAGCCGACTAGTTTTTCGTTCCTCTGCATCCCTCTCCGAAGGAGAGAACAGCACTGGATGCCAAACCCC

CGGATCGATCAATCCAAAGTTTGAACTATGGTTTTGATGAGGGACAAAAAGAGTTAATTTCCGTTGTCCTCCTCCATTTG

CAAAACCCAAAACACTAGTCCCCAATTGAGGCCGTAACTTGTTCCTCTCCCCTTTTCTTTTAATTCAAAAGCCGACTTCT

ATTAAAATCCAAGTCAAAAACCCCCAAAAATCCTCGTTTTTGGCTTTTTATTTGGAGATTTTGCAAAGCAAAATTAAAAA

ATTTGATTAAAATCCCCAAGTTCTCCGCCGATTGATTGATTTCCCAAGGGAAAAGAAAACCCTCCCTTCCCTTCCCAGGG

CATCACCGATCGGAATCGGAGGGTTTTGGCTATTTTGAGATTTTGCAAAGCAGCCAAAATTTCAAAATTTGATTCCCTTG

CGGAATCCCTAAGGAATGAACCAGAATGATTCCCTTTTTAGGAATCCCTCCGGGATTAAAAAGAAAGTTTGGCTCCGTTA

TTTCTCCCCGAGGGGAGAAGGTCCCTGGAGAGGGACTAGTGGTTTTCGTACCTCCTCACGAAACCCGTTCAAACCCCAAA

GGTTGTTTGCATCCTTCCTCTCCAAAGGAGAGGAAGGGATGCAGAGGGACCAACACGGGTTTCGTGGCCTCAAAACTAGT

CCCCGATTTCCCGAGGGACTTATCCCTTCCCTTTCAATCCCCAATCGATGAATCGATCCCCTATCATCGATGGGATTGGA

AGGAAGACTAGTTTTCGTTCCTCAACGAACTCCCCGCCGTTTTGAGGGACAAAGGTTTCCGTCCCCCAAAACTAGTCCCC

GATTCTCCGAGGGACTTATCCCAATCCCCAATAGATGAATCGATCCGATCGATCCTTCGGGGATCCGGACTAGTTTGCAG

GAAACAAACTTGTTTGTTTTCAAAACCAGAAGTTTTAAAAACTCGTTCCTGAGGGCCAGGAACTGGTCCCGAGGGAAATT

GGAAGTCCCCGACTAGTTCCTGAGGGCCAGGAACTGGTCCCGAGGAAAAGTCCCCGATTGGACTCAATAAATAGTTTGGC

GTCCCTCTGCATCCCTCTCCGCTGCAGGAGAGGATGCCGCCATAAACCCCAATCGATAAATCGATCGATCGATCCAATAA

GGTTTGGAACTTGTTGTTTTGAGGAGGGAAAACCCACTGCCCCGCCCAAAGTAAAGTGAATTCCGTTCCCCATTTGCAAA

ACACTAGTCCCCGATTCGGAGAGGGACTTATCCCGAATCCCCTATCAATCGCGGATTGGGACTAGTCCCTCCCCGGGGAC

TTATCCCAATCCCCGATGAATCGAGGATCTCCGATGGGATTGGGACTAGTCTAGTTTTGTGGGACAACGACACCCGTTTT

GGTCCCTAAAACAAACAAGAAGTTAAAACCCTGGGTTTGGCATCCTTCCTCTCCAAAGGAGAAAGGGATGCCAGAGGAAC

GAAAACCACTAGTTACCTCTCCGAATCTCCGATCAATGATTATCCCCGATTTCCCCTTTTCTTTTCTCCCCTCGGAACTT

TATTTTAATCCCCGAATTCCTTTAGGAATCCCTCAGGGGATTCCCGAAGGGAATAAAGTCTTTTAAATTTTGGCTGCTTT

GCAGCCAAAATATCAAATAAAAAGCCAAAACCCCGGATCGATGATGTGGGTTTTTACTTGGATTTTTAATAAAATTTTGG

AATTAAAGAAAGGGAGCCCGGAGGGTTTTTACAGGAAAAACCCTCCCTCCCCGGGGAGAGGAATAAGTTGCGCCTCCTGC

GATCATCGGGGACTATTGGAGTTTTGTGGGACCCCCAGCACAGGCTGCGGTCCCGTCCCTCAAAGGGAGTTAGTGAGGAA

CGAAAACTTCCGGATTCTGGATTACTCTCTACCCCTCCTTTAATTCTAATTCCCTGGGGTGGGTTTTACAAAGTTTTCTG

CACGAACTATTTTTCGAGCTAGGCTGACTAGTAGTTCCCTTGGATTGCAGACAAGGCAAAACCCCACCGATCGATGATGG

GAAGGAATAAAAATTTTATTCCTCCCCTCGTTTTGCGATGATGGGGAATTATTATTCCCCATCCTTGACAAAGCAGCAGC

AGAAAGGGATTTCCCCAGCAGGGATTAGATTTTTAAAGACTTTTCCCCTAAGAGGAGCCGGGAATCCCGGAGGGTAGGGA

TGAAGCAAACAAATTTGTTTTAGCCATCCCGGCCCATTTAGAGCGAACCGAACCGAACCTGGCCAGGTTTTAAATATCCA

TTCCCATTCAACCCGGGAGGGTTACCCCTCTTTTCTCCGAAAAGAGGGTTCTTTCTCCGAAAGAATCGGAGGATCTTGGT

AAAAAGATCCTTGCCAATCGGCTGCTATGCAGTAGAGCAAATGACTCTTCTTAATCCCCTTTAGGGGATAGTATGGTTAT

CTTAACATCTTTGCCTAATCCGTCTTTTTAAGGAGTCATTCTCTAAGCTCCTCCCCTTATTGATCGGCGTGTTTTGGAGG

AGCTTATACTCCTTCGTCTATCCCCTAATCCCTGCGTAGCTTCCGTATTTAAAGGGGGATACCCCGTGTCATAAGGAGGA

GGGAGCCCTCAAACCTGACCCCCGCTCAAATACTCCTTCCGAAGCGCGCACCTTAGGCCTAAACTATCCAAGAAAACGAA

CTTAAGCCTTCTTTTTCCTCTTTTGGCGTATCCCCTAGCCCGGCCTTATCCCCTAGCTGCCTTCCTCCGGCTGTAATCCC

CTTTAGGGGATAAGAGGAAAAAGACCAACCTCTTAGAAGGTTAGCACCTCCTGCCTAGTATTCTTAAACTAAAAAGATTT

ATTTTTAATCAGGTATCCTTCGGGATAGGGTATCCCAGATACCCCTGCCGCTATCGATCGGGGGATAGGGTATCCCAGAT

ACCCCCATCGATCCGCAGGGGATATAAAATATGTAAAGTTGAAAACAGTTTGAAGTATTATCTCTGATATATATAAAATA

ATATATTTTAAGAAATATCTAGAATATATTTGGGATTGCTATCGCCTATTTGCTATTGGTATTTGGTATTGGTATTGCTA

TTGGTATTGGGGATTGGTTATATAAAAATAGCCGCCAAAGAAATTCCCCGATGATCGATGGCTCCCTGGGGGTTTGTATA

TATGCTGAAACTCCACTATTAAATACTGATTTAGTCTGCCTTAACTTAGTACTTAGTACTTAGTACAAGGTACTATTTAC

TATTTACTATTTACTACTCCCTGCCCTACTTAGTACGGATCCCTGCTAAGGCGCGGGATACACACAATACACCCCGGGCG

AAATTAAACTGGAGGCAAGTATTTCCTCCCCTTTAACCTGTCTGCTTAGCCTCCCCAGCCTTTCTCCCTCCCTCCCTCCC

TCCGCAGCCTGCCCACCCAGGGACCCCGATCGATCGATCGATCGATCCGATCGATGATTCGAGGGGTCCCTGGGAGCTGG

GATAAGGCCCATAGGCTCTGGCTGCTTGCTGCAGCTAGCCCTAGGGCCTAGCCTAGCTAGCCTAGCTAGCTAGGGCTAGC

TAGCTAGCTAGCTAGCTGGGGACTCCAAATGCTCCCCTAGCTTACTTGGCCGCCGCCAGCTTAGACTCGTATGCCTAATC

CCTCCAGGCTTATCCCCCGCTAGCCTCCCCTAGCAGCCCTACCCTGCCTACTAGCCTGCCTACCCCCGATCGGGGCAAGC

AAGCCTGCCCTAAGGCTTATCCCCCTAGCCAGGCCAACTCCCCGTCTTTAGAGGGTGGAGGATTAGGGGTAGGAAAGGGA

GGTAGCCTCTATCTTTTCCTACCCCCTACGCAGCCTACCGGATAACCGAGGGAGCCAAAACTACCCCTATGGATGGATGA

TGGATGGCCAATCGATGGCCCCTGGCTGCGGAGGCCAAAACCTGGCTCCCTAATGACCAGTTTTAAAGCCATAGGAGGAA

GGAAAAATATGGAGTGAGCCTCGGCTAGCTCTACCTCTTTAAGAGCCAGAGGGCAAAAGGTATGCCTTCTCCTTAAGAAT

CACTATAAACATAGTTGTTTAGAACAAATGGGTCATCCATTAAAACTTTGTAAAGGTAAAGATACAAAAGGATGCGGCTT

AGGTGGACTATTTAAAGCTCACTCTATACTAATACTTGTTCTGTTTAATAATGTTTGTAAAATATCTATATAAAACTCTG

AGTTAACTCAAGGATATCTATTTACTGCATTACCCAGAACTAACTGTAAATAAACTAAATGTAAGAATAAGAAAGTAGCT

ACCACACTAATAATACTTCCAAAACTACTAATTAGATTTCAACCTGCAAAAGCATCGGGGTAATCACTTATACGACGTGG

CATCCCTTGTAACCCTAAGAAATGTTGCATTCTATAGCAACGTCAGCACTGTAAAACTATAATTTATCTTTCCCCGAGGG

AACCCCCAATCGAAGGGGACCCAAGGGGTCCCCAGGGACATAACTATATATTTTTCCTTCATAAGAAATACCTTTATCTA

TTTCATACCCCTCCCAATTTTATTCCCCAATTCACCCCCGATCCTTTGCGAGCACGGCTACGCAAGGGGTGGAGGGCCGG

GGAGGGATCAAACTTTTAGTGTATAATAATTCACTTTCCCGGAGGGTTGCATATTTAAAAGCTTTAGTTATACTTTTCAA

TTCAACTATTAATTCATGTGAATTAATATCAAATACATAAACTGTTTTACGATTAGATACTTCTTTATCTCGGAGATTAT

GGGAACCTCCCTGGTTCCTTAGGAACCCGTGAGGGTTAGATATGTTTACTGGAAATAATAATAAACCATTATCTAATCCA

CCACCCTTATACTTGAGGTATTAGGCGAACCATTATCCGATGGTCAAGGAGATCGATCCTACTAACAACATTTAATCACG

TATTCATAGTCTTGCTTATTCGTATGTAGACTATTTTATAGTGGTAGAATAATAGTTTATATTTCTAAGTATACATAATT

GATTATTGTAATTGACTCCATCTCCCTTCTGTATTTTACTAGAGATGATTCGCTTATCTAATACTAAAAACTTAGCACAG

TCTGCCTGTGATTTAAACGTTTTAATAAGAGTACCGTCAGGATAAATAAGTTCTACTCCTTTTTAGTACCCCGCCTTGAG

GCTGGAATCTTTGCTCAGAAATAATAAAAATTTACCATCGTTTCTATACTCATAATTAGATGGTTTAGATAAAAGCTCTT

CTATCTTAGTTAATAATTCTGTTCTGTCATGAACATTTCCACGGGTAGATAACCGATTGTTGTTCATTTGATTTAAAATT

AGTTTTATTATATGCTTTCCTTCTGGAAGATAATGAAGACCTTTCTCATGAATGTTAAAATTGCAACTCAATCCATGAAG

TCTAAAACTTCTTGGTACGGAAATTTACAGAACTAAATAAAGGTATTATTACCAACTTAATCAAGTCCCTTCTAGTTATT

TTTATATTATAAACCGTAGCTATAGAATAAATTTGGATTTCGTCAAGATTAAGTGACAATAATTCGGGCTTTATTTGCTC

TGAGGCATAATTACACAGGAAATTTACGATGGCTTCCATTAAAGCTGCTGCTTTATTACCTTCTTGAGATATAGAAAAGC

CTAGACTTTTATTACCCTTCTCCTCCCTCCTCCTGGAGGAGGGGAGAAAAGTATAGAAAAATGATCCATCACCCTCAACA

AAACCTCCTTCTCCTCCCTCCTCCCTGGAGGAGGGAAAAGTAAATTATTTGTAATGACTATCCGATGATCAGAAGGCATA

ACGAAATCTTTTCTCTTACTATTCATACTTTCAGCTATTTTATCTAAGATTCCCTCCTCCCCGAAGGGAGGAGGGCGATA

GGATTTATTTCTGCACGGAGGCTATGCTTACTACTTTTATTATAAAGCATGTAAGCTTTTGCAAAAGCTAAGAAATTTAA

ATGTTTTGAAGAATTTAAACTATACTTAGAAAAATAGCCAGTATAATGGAAATATCTTCTCGTGAAAATATGGTAAATAT

AGCGGATTTTGTAGCCTAGCCCCTATTGCTAGAAAAGTTATAAATAGATCCCAAACCCATTCCCTCCTTAGGGGAGGAAA

GTAGTTTTAATATACTCTAAAGCAGGCCGCATCATCAATATGAAGATCCATTCTAAACATGAACTTATAACTGTTTCCTG

AACCCTTAGTAATTAAAAAATAACCTTCACTATCAACTATACCACGAAATCATTCAATAAACTATCGAGAAATACGTAAA

TTTCCCCATTATCTAATTGTAGTCTTACCTTCTTTCCATCTTCAGTCTCAGTTAAACGACTAGCTAATTCTTTTAGTTCA

GAAGAATTGGCTAGTAACTGAATTTTTCCCCACTTTCAGCTAATTTATTAGAAGATGTATAAGAAGGATTGGAAAGCTTG

AAATTAACCTTAAATAAATCTTTCATTGAATAAACAGAGATACCAAAATTAAAATAAAGAATATAAAGCTTTATGTAGTT

AATAAAAATAAAATTTAAAGCCTTAATATTATCATAATAAAGTAATATTACATTTAATACTTTATCCAAATAAATTGTGC

GAATCCGTTGCAGACTGGTGACAAATAAGACTGGATTCATCAATAAAAATTGGATGAATTTTTATCTAACCTTTTTTTTC

ATCTATTTATTTTTCACTAAGTTCCTACATCGTAAAAAGGGTGGTAGCCTCGTCTCACGAAGTACGCAGATTGCTTGCGC

AATATGCATAGTCCTTCGGACTCAGCGAAGAAAAAGTCTTGGTACTCACCCTGGGCTATAGCTGCTCTAGCTACTTTACC

ATAAATTTGGAAATATCTAGCCATTTCCTATCGCCCTCCTCCCCTTCGGGGAGGGAGGGAACCAGATTTAAATTCTATTA

TCACCTCATTGTCTTCATTATAAACAACAATGTGCTTTCTAAGTTGATTGTCTCCAGTAGGTTTTTCTTAATATTCCGCA

AAATTATCTACTATACTAAGTATAGGTTCAAAAGATAATATAAGATTTTATTTATAAAGGTATTTATTGTTAACATAATT

TAAAAGTATACTATAACTAATTTGTAATCCTTTCAAAGCCCTGTTTATTGAAGAATAAACGATAGGATCTTTCGACGGTG

AAAGGACATCATACACAAATACTAAAACCAGAATATTTATTATCTATATCAATAGATGTATAACTCAAAGAATTAGCGGT

TTTAAAAATTTTAAGCCAAATAGCAAATCTATTATAACCTTAAGAACCTTTAGAGAATAATGATAATAATTTTTCAATTG

TTAAAATAGCATTATCTAAATTACCCCATTGAGGATTTACTCTGGGGTAACTTTATAATAACAATTCCCGAAGGGCATAG

TTGGTTTAAATTTAATAATAGCATATTGTTCAAATACTAAGGAAGTTTGAGGAGTAGTAAAATACAGAAACTCTAAGCTT

CAATATAAAGCTCTGCTTTTGCATATTTCCAACTCAGAAGCAGAGTGGGGATTACGTAAACCTTTAGTGGGGCAATTTAA

TATATTCCTCCATTCTTCTATCGCCCTCCTCCTTCGGAGGGAGGGAAGCTAAATTATTAGAACTACCTGCCGCCTCTCGG

AAGTTACCGGTATAAACCTATTATGATGAAATGGTACCAAGATTTAGTGAATATCTTCGTTATGCACGGGTAAGTTTAAG

TCGCCTTTTAATAAAGCTGCTTTCTGATAGACAGACGGCTTTTTAAAAATCTCTTTTCTGACTTGATTCATATTTTTCCA

TCCCTTAGTTCGGCATTTCCCGCTATCGCCCTCCTCCCCTTCGGGGAGGGAGAAGTTTGAGAAAGTCCGCCGACTTAAGA

TAAGAGAAGCTCCTTTACCGTGATATTATTCCCGGAAGTAGACAATCGATTATTATTCATTTGGGCTAATAATTTATTAA

TTAAATCTTTTCCTTTGGGAGTATAATGAAGAAATCTTTGTTTAATACAAAGAATAGCTTTTCAATCTGGATAATCCTCT

GATTTCTTACTTCGTCATATTACCCCATCAAATAGAGGAATAATCACTAAGTGTTTAATCCTCCTAGAAATTCTAAGTTT

ACACACGGCCACCTTCCGAATATAGGTTACAAAAGCTTTTACACCCTTATGAGTACCCAAGTTATTTAAATAAATTTCTA

ACCCTTCCATTAAAAGTTTATTCCCCTTTTGAGTAATCCCTCCCCTAAGGGAGAATGTAATAGAATAACCAAGTTGATAA

TTTCTTCTCTCCACGAAAAGAACCATCCCCTTCGGTGAATCCTAAAAATCACCCGTCTGTAATTCTTAGTTCAGATGAAT

CTGGCATTTAACAAATTCTTCTATCGCCCTCCCTCCCCGAAGGAGGAGGCCCAACTTAGAATTAATGCTATCTCTAATAT

TTTCTACTAGGTGAACTACATCCTTTTTAGCTATAGCCGCCCTTGTACAGCTTAAAAGCGGTAGCCAACGCTAGAAGATT

TAGCTGTTTAGTTGTATTTAAGGGATGATGTGTGAATATATCAATTATTGTCTGAATCTATTGTTGACTTCTATCGCCCT

CCTCCTTCGGAGGGAGAAAACTTTAAAAATAGCTTGATTAACATTAGTATATACGAAGACGGATCCTATACCTAAGGTTT

CATGAATGAAATTTAAAACCTTAACGTCATCAATGTGCAACGTTATTGCAAAGGTAAAACTATAATAAAGCTTTTCTTTA

CATTCGATTAGAAAAGCTCCTTCCGCGTCTGTAAACCCTCTAAATCATTCGATAAAATAAGTATTAAAAGAATTAAGTTA

AAGAGAATCCAACTTAATAGATTCATTATTATCTATATTGCTATCGGCCCTCCTCCTTCGGGGAGGGAGGGCAAACTTAA

GATAAGACTTTATAAACAGAACCGGTAGAGTAAGAACGTTTTAGTATCGTAGTAGCCAAATTTGGTACCTGAAGACTCAA

GTTATAAGAACTAGACGTAAATTTTTGGTGAATTTAGATTAGATAAGTTATCAGTGCTTATTTGTCACCCGTAGATGTAG

ACCATATCACTACCCTTCCTAGCTTGCCCCCGGATGGCAACTTAACTGGTTGTTTGTCATCCCTCAAAGTTTTGAGGGAC

GAAAACAACTAAATAGATAATTAATTATCCCTGCGGGTATCCCGAAAGGATCACATCCATCAGATACCTTCGGGGTATCC

CCGAAGGGATAAAGGTTAAGGTTAGGGTACTTGGCGTATGGTCGTTGAAGTGGCTTTACATAGAGATGTAATCTCTAAGA

AAGATTACCTGCTGATTGGACGTAATAACCTAGGGCAAAATTAAACGTCTTTCCAGCAATCTGCCAAGTTTTAGGGGTTT

AATACCCAGCTGCTGGCACAGCCATATCTCCCCTTTACACTACATAATTTCTAATGTAGGCCCCAACATTAGAGGGAAGA

AAGTTAAATTACACAGTAACCGTAACTGTGGACTATATCATAAGATAACATTATGTTATCTTTCTTTCGTCTAGTCTCTG

AGGATCCCCTACGAGCGATAGTGATCCGGCGATCCTACTACCTACTAAGACAGATATCGTTGCGTGGGTTTCCTGCGGAT

TGTTTAGATACTCTTAACATATTTACTGGTTAATTACCCAAATTTTTGGCAGGTAGTCCCCTATAAAAGTGGGTGGGGAT

AATTATATGCAGTAGTTAAGCCTTATGAAACTTCTCTGCATATAGAAAGATGGACGGGGCTAATTAAAGAGGGACGTTAC

GAGGTTTAGATTGAAAGATTAAATATTCATCTTTAATATTTATGTATTGATTATTATCTATCCCTCCGGGGATTCCCCTT

AGGAATCCATCCCTGGCGGGATCCCGGGGAATAGTGTCTTTATAGTTGTTCATCTTATTTTAAAATATTGTCTAGCCGCG

TTTATAGAAGGAAAAATTAATTCATTATTATTTTTATCATAACAATAAAAAATTTACCTCCTATACCATATTGGGCTGAG

AGTTTTCCTTTTAAACCTTTTTAGTCCCAATCCCCGATTGGATTGGGATAAGTCCTCCCGGAGGGACTAGTATGAGAGTT

ATTGATATAAAATTGTTTAATACCTTCGCTAATAGGGTTTTTCGTATCCGAACTAGTCTTATATCCATATTTAGGATGAT

TGGCTTTTCTGAAAAGTTATTTTAGTTTTACTATTGTTTCAATTTTATGAGTAAAACCTTGACAGCTACCTGCTATTTTA

AATATATTATACTTCCGCAGGCCGGTTTATAATGATCTATTCATTTTGGTTCCAACTTTATAGAAGTAATTAGATCTTTT

CACAAAATTCCAGTATTCCTCCCTCCCCGAAGGGGAGGAGGCCGATAGCTAAAGAGAAGTTCTGTAATTAAGATTTTCTC

ATCGCTCTAGCCAATATGGTTTGCATTTTTCCGTACTCGCATAATAAAATGGCTAGACATTCTTTTAGTTAAATTTAGAC

TACTACCTACATATAAATCATTCGTTAAATTATTAATAAACATGTAAACTCCAGATTTAGACCTTAGTTTACTGTATATA

TCCCTTTTACTTTGTTTTATATCATTAAAATATAATACATAATTATTTCATACCCTCCCAATTTTATTCCCCAATTCACC

CCGATCCTTTGCGGAGCACGCTACCGCAAGGGGTGAGGCCCGGAGGGATTAGGTGAATTATTGTTAGGGCTAATATATTC

ATCTAAACCATTTAATGCATTCCCTCCCTTGTCTCTAACCCCGATAAATAAAACTCAGAAATGAGCTTTTCCTAACATAA

TACTATAATCTAGACCTAATATTTTAGGTATTCAGAAATATCATGCACTATATAATGCAAACACTGCTCCCATACTTAAT

ACGTAATGAAAATGCAACTTATTTTCACCGGCCTTATATAAGCCGGCCGGCTAGTATAAATTTTGGACTGTCTCTTTACC

CGCAATAATAATACTATTTATCCTCATATAACAGGAGGGTATTTACTTACACAAGGAATTTTATATCATAAAGGATTTTT

ATATTTAAGTAAATCAATTACAAAGTTTGAAGAAATTCTGTGTTCTAAGCTATGTTTTTAGATCTTTGATATTTTCTAAT

CTCAAAAAGATTTAATTTTCATAACCCTATCCCTCCGGGGATTCCTTCAGGGAATCCCTGGCGGGATTCCGGGAATAGAA

TTTAATATCACAATATAATCGGGTGATTATGAATAAAATAATCATATATAAATAGCATATTATCGACGTTTTTAAATTTG

AAAGCAATAGAAATGTAGTCTTTAGGACCAGCAGATTTAGAAGATTTGGCACGTTTAATAATAAAAGGAGGTTTACTATG

TAATATAGTATTATCAAAGTTTAATTTAGATGTGTATTCATTATATTTGAATTCTAAATTACACTCAATTCTATTTCCCA

ATCGCCCTCCTCCTTCGGGGAGGGAGGGAAGCATGATTAAATACTATACTACCATCTGTGTCAACTAAACCCGCAAAATA

GGGATCATATAAGGCTATATTATAATTTGCTTCAATATAGTCTATCGGCCCTCCCTCCTTCGGGGAGGGAAGATTGTATA

AAATACAAGCCTCTTTAAATCCAGGTATTTTTAGTCTAATTAAACCCTTAATACGCTTAAGAATATACATCATCCCGGAG

GGCCCGTTTCTTTGCTGGAGACAATATAGATAGAATATGGCTTATTCTTATCCGCTCTAATTCGACCCATATGCAAGTAA

TCTTGTATACGTGTTAATATTCTAACATCTCTATTGTGCAGTTTAATTCTAATCCCTCCGAAAGGGATTCCCTTCAGGAA

TCGGGATTCCCCGGAATTGGTTTTCCCGAGGGGACCCCAATCGATGGAAGGGGACCCTGGGGTCCCCGAGAACTATTTTT

ACTAGTGGGATCCGTTCTAATATAAAAATTACCGTCCCCGTCTAATATACCTGCAAATCAACTTAAAACTTCTAATCTTC

GGTTTTCCTTCCCTCTGAGGGGAAGGAAAATGTAAGATAACTGACGTACAGTCTCTGGGAATCCTTTACAGTTTTCTGCT

GATTGTGTACTAGGAAGTTCTTTATTTGAACTTGTTGTATCGTTATTTTCACGGTTTAAAAGAAGAATATTCCTACTAAC

ATATTTTTTATGCCCGGCCAGATAAAACGTATTAATAAACCGTAAACAATACACATATAATACAGTTTCCAGCATATAGT

CAGTTGCAGAAGAATACGTTGTAGAAGACGTATTATTCTGGCCCATTAGAGCTACGACGTAATAACTTTGTAAAACCTTA

GGTTTTACAGGACTAGCTCTTCATTACCAAATAGGTTTAAATAATTAATTATATTTGGTAACGCGCTGCGTATAGTCTCT

ACAGAACTTTAGTGATAATATTACTAAAGCACCACGGCATAGCTTATAATAATGTCGCAATGTATAAGCATCATTATACT

TATTATAAATTCCACCGTTAGCAAACTAAACTGTTAGTTTACCGAACAAACAAGTTTGTTTATTCAAGCAGCGTGACAAC

ACAACAGATAACAACAAATTGTAGCTCCTTTTAAACAGATTTAGGTTTGTTATGATATAAATAAAACTTATTGTAAGAAA

TTAATTTATGGCCTAATAACGGATAACTTTCAAAATGGTTGAAAATATTTTTCTAGTCTTTTCATTATAAAGATGTAATC

TATAATATGAAAAATTTCCGTCTTTTGGTTTGTCTTTAAGTATTTTAGTTTCACCTTTAAAATAAGTTTTTATCCACTCT

AAAATATGTATTTCATCGTTTTGACCTATAGTGAAAGCACTTTTTCTTAAAGAACCTTTATCGTTAAAAACTAAAGAAAA

ATTTCCCTCACCTTCTATAAATCCGCTCAGTCAAGCTGGAAAATAGTTTGGTATTTTTTATTTGCTAGTATATTTAATTG

TTCTAGTTTATTCTCGTACTTTTCCCTTCTCGATTGAATAAACAAGTCATATTTAAATTTTCTATTCAAACAAGCGGTAG

CAAAATTAAACTGGCTTTGTTTTCTAGCAGTTAGTAGAGGGTACTTTGCTAAAATTGCAAATAATTTGACTAAATCTCGT

TCTTTTGACAAATTCACGTTACATATTCATAATCTGGCTTTTTCAAGTGCAACATGTCCACATATATATTTAGCCATTAT

ATTAAGCATATTTATATTTTCCTCGTTTCTCTTTAAAGCTATAAAACCCTAGTTCGAGGTTTGGGTTCTATTGTTTTATT

AGATCTAATATTAGTAGTAATAGTACCATCTGCTTCTAATAGACCAACAAAAAGGATAAATATAGTCTAAATGGGTGCGA

AGCTGCGAAGCTGCGCAGCTCTTATTAATACCATTACCTGTTTCCTGGGGTTTACCTAAAGCGCATATTGCACTATCGCC

CTCCCTCGGGGAGGGAGGGAAGGAGAAATTAATTTGTTAACGTTATTATCGTGGAATGCAATATCCAATGAGTAGGGTCG

AATGCCGCACCCTGATTATACATGTGTATAACTGGCAACGAACACCCTCCCTCCGAACCGTACGTGCAACTTTCACCGCA

TACGGCTCTCCGGAACTCTCACTAAATTATTCAGACTATACCCCCCCGAAGGGAAGAGGCTTGATTCCCCAACTCTTTTA

GACTCATTCCATCATATTTACCGCCATGCACTTTTCCGTTACATAGACGGCAAAGAGCTATAGCTTTTCTACGTATCTTA

CTCATAACTTTTAAGGTTTCCTTATCTTTAGTGTCCTTAAAATGTTTTAGATTATGCATCTCCCTTCTCTTTGGACTCAC

ATATTAGACAAGGTTCATCTAATAGGGGACTATTATATACTCTCAGGTAGGTCATACTTAAGACTGCTAAAATCGCCCGG

CTCTAGGACTTAAGGAATAAAGGCATACACATCCTTTACTTACTACCCTCCCCTAATCGGCCTCCCCGATCCCTGCAGGG

GATTAGACGCTGGGATGATTAAAACCAGGGATGTATACTACCCTCCTAATCCCTCCCCGCCCGATGCCCTGCAGGATTAG

ACGCTGGATTACAAGGGGAGAAATAAAAGAGGGATTCTCGACTTTCCTCCGAGACGGAGTCTCGCAGGGTAAATTAAGGT

ATTACGCAGATAATGATTTAGCTTAAGGGGATTAAGGCAATCGCCGACCATTTACCATACATTTAACTGTCTTGCCATAT

TTTTGAAAATTTTACTTGTAGTTGATCTATGTTTAGCGGCTAGTGTATGCACTAGGGAATATTCTAAAATATACAGTGCT

TCATTAAGATTACTTCTGTTTTCTGCCATATTATAGAAGTTCATTATACCTCTCACAACGTAATTATATCTGGTGATAAT

TTCATGGTCTGGTAAGAATATGAACTTATTACAACTTTTGGGGAATCCTTTTTCATTTGCAAATCCCTTCTGGATTAAAC

GCTCTTTCAGTAGTTCTTTCGGAACCAGAATCTTAGGTTTCCCAGTTGATTTTCGTATGTTCAAGCTAAGTCCAGTTTGT

GCGCTGCAGCTGCTGCTGCTGCTGCTGCTGCTGCTGCTGCTGCTGCTGCTGCTGCTGCTGCTGCTGCTGCTGCTGCTGCT

GCTGCGGCTTCTTCAGGTAGGTAAGGAAGGATTTTGGCCTAACGTTTGTCCCTTAATATAGTGTCCGAGGAACGTGGCGT

ATTCGGACCCCAGATGGGTTATTTTAGTTTTATCCATGTTCAACTCTAGTTTTAATTCTTTGCTTAGGAATTCTTTAACC

TGGTCTCTAATTTCCATTGCCAGAGATTTAGGGCCAGATACTCCAATTAGCCAATCATCTGCGTATCTAACATAATGAAT

TTCCCCTCTTCCAGTCCTTATTGCTGAGGGCGCTTCTGGTGTTATAAGTTTTAGACCTCCTGCTTTAGCAATAGTATTTT

TAATGTCTAAGATTTCGGGTTTTTTCTTTAGATACCCCGGGGTATCCCAAGGGATGATATCCTGACTTTTCTTCAAGGAT

TTACCATAATCGATTCATACTTAATCCCCGCCTTAGCGCCGCCCTGCGCTAAGGGGATACGAAGGTCAAATTACGGATTT

CTTTTCGAGATTGGTACCTTAGGGGATTATATTTCTCCTTCAAGGTATCTACATATTCATCAAACGGTGTAAGATAAAGG

TTTGATAGTATTGGAGACAGTATTCCTCCTTGCGCAAGTCCAATTCTCCCCGATCCCGCCGATTGGCCGAGGGGCAAATT

AGAGATTCCAGATATGCCTTGTCTACTTCCATATATCCTGCTCTGAAGAATTTCCAGATTAAATTCATTATGGTTTGATC

CGGTTTAATTCTATCTTGAATTAGTTTGGCCAAAATATGATGGTCTATATTGTAAAAATATCCAGTTATATCCCCTTCTA

TTATTCACGTAATTCCTGTCATTCTCTGTATTTCTAACAGTGGTCCCCCGATGAAGTGGGGTACAGACTTTTGGTCGGTC

TAAAAGCAAAACTCTTCGGATGGAATATTTTCTCCAATTCTGGTTCCATTAGGTCCTTTAATACCGTTTGTATCACTTTG

TCAATTGTACTTGGTATCCCTAGGGTCTCATTTTCCCATTCGCTTTTGGTATGAAGATTCTCCTTGTCGGTTTAAACTCA

AATGTTCAATCAGTTATGCTATTACGTAGTTTTTCTAACTTTATTAATGATATGCCATCCAGAGTATAAGCGTCTTCTGA

ACCTTCTTCAGTCATATTTACCGATTTCGATTTAATTTTTTCGTACGCTTTTTATACGTAGCGATGTCATTTAGTGCCTT

ACTAGAGATTCCTCTAGTTTGGTGGGCCACGAAACCCGTTTGGGGTCCCTCATCAAAACAACAAGAAGTTAAAACCCTTT

GGGTTGCATCCTTCCTCTCAAAGGAGAGGAAGGGATGCAGAGGTACGAAAACTAGTTCCCTCGAGGAGGCAGCTTGTCCC

CTGCGCTCGAGCGGAACCCTGAAGCGCTCCCCGCGGAACTAGTCTTCTGAACGTGTAAAGGATAATAGGCCTTTACCTTA

GTATTCCACAGAGGATAAGCACGTGAATTTACACTTGTGGCCATCTTGTCGAAGAGAGATTCCTGTCCCCTTCCTTATTA

AGTACTACGGACACTCCGTCGCCGCACAAGATTGCTGTGCTGTGGTTTTCAAGTCACCACATAGGAATTGCTTCCCTTAG

GCGATCCCCAGTTCCTATTATAAAGTCTGTTATTCCATCTTTAGATCCCATCGTTATGCATGTTGCATATGTCCTATCCT

TTGAATTTTTGTTAGTATAATGCTTCTCTACTACCAAAAGGGATATACTTGTGTCAGTTACTTTCAAGTAAAATTTAGCG

TCTTTGGACGGATTAATTATTGGACCTTGGATGAAAATTATCTTGCCACTAAGATTGTCGTACCTACTTTCTTTTGTGAC

TTCACTGACATGCTCCACTCCCTCCTCATTTCGATTTTAGGTAAGTCAGTAGATTTACATAACCTAAATTCAGTTATGGC

CTTTAGTGAGGCGGGCTTCATAATAGTTAAAGGGCGCACTGCATTTGCTAAAATAACCCCACTTACCATTGTGTAAACAC

ATTAGATTTCCCCTAAACTCTAAGATAATCGGGGCTAAATCTGTAACCTTTCTTGTGGTATTATTTACAAGACTTTCCTT

TATTTAGTCCCAAAACCCCGCTAATCCCCGCCTATTTTCCTTAGCCTTAGCCTTCGGCGGGGATAAGGGGTTTAGTGACT

TGCCACTGCCATTACACCGTCATATCTGCATATAAATTCCCTGCCATATTTAAACACCCCTAAAGCGCGCAGGCCTTACC

CCTTGCTTATCCCCTGCGATCATCGCCGGCGAATTTCGGGGATTAGGCAAGGGATACCTTTTCTTATTTCATACCCCCCT

AATCCCCGATTTGCTGCCCAATTGCAAACGGGCTCCCCTGCGTAGCTAGGGAAAGGGGAATCCCCTTGGGAGCCGTAAGG

CAGGGACCTCCTTTGAAAGATCCTTAGACAGGGGGCAAGCCAGGATTTTAAACCTCCGGTCAGAAAAGATTGTATGTTTT

GGGGGTTACTAAATACTGGGCTCAGAAAGACATAGACAACATATCTAAACCCCTTGGGTTTTGGGACTAAATAATGTGGA

CAGCCAGAGTATAGACCACAACCCCTGGGGACCAAAAGGATGTGTAAAAAATTCATTAATACGCCGTAACCAATCTTTCA

TAACTAAATATATACCCTTTATTACCACCACCTATCTTAGCATAATTTTTAATTTGACAATGTGAGATATTTAAGGCTCT

TTGAGCCTCCATTACTCCGTCATATTTAGCAATAAAATTTCTATCTATATCATATACAAACACTGCTTTTCTAATATGAC

TATTATTGTTCATATTTAAGACTAATTCCTCACATTCTTTTCGATATCAATCAGCTATTATAGGAGTGTCATTTATATTA

TAAGGTATATTGGTTAAATATCATTCTCCTCTAAATATTATTTGTTCTTTTATAATATTAACTAATGTAGAATGATTTGA

TTTAATTAACTTAGCTAAGGTTAAAACGGAAGGAAAAATAACCAATAACTCTTTAAATGAGTTATAAATATAAACAGAGT

AGGCTGAATTAGCTTCAATCATTCTTATCTGACTTTCAAGAGAATGACTTTTATTATAAAAGGATTATTTTCACCAGTTA

CAGCCCTTGAAATTAAACCTTTAGTCTTATCAGAATGTGTTCTATTCTTAGCTAATTCAGATAGTAACTTCTTAGTTTCT

TCTGTATGTTTATAACCTAAAGAAGAATAACCTTCTTTTAAAACATTGTAATAAGGTAACATATGGGTTATATAAAAGTT

TCTCTTGCAGTTAAAAGAACTTTTCAACATATTCTAAAATTAAAAGAGAAATTTGAATGATCGTATTTAAGTAAAGCTTT

GGTAATAGGCATATTAGCATTTGGTTTACTTTTAAAAACTCTTATTAAGATAATTTCTCATTCTAGAAGCTAAATTAATA

GAACTACCCACGTAAGCATGCCCATTTACCTTGTTTATTAAACAGTAAACACCTGATTTATCTCTTTGTTCTTTTAGAAT

ATTGACTCTATCTTCCTTCATGCTATCATAAACTTTAACTCCCTTAAAATTTGGGATGTTATCTTGGTTATAAGGCTGTT

TATTACTAAAATTAGAATAAGACCGAGTTATTCCGTATCCTCCCTGCAGCCCTAGTAGAATGGCTACACCGAGGGGAATT

AAAAATTCCCCATGAATTTTTCTCACCTGGACTAAATCTTCCCGGAGTAAACACCGAGGACTACGTATAGTCTCTGAGGA

TCCTACTATAATATTACTAAACCCCCGCCTTAGCGGTATCCCCAGCTACGCTGGGGATTAGAGGGTTTTGGAACTAAATA

TTAACCCCAGGTTTCCTGCTGATAATTCAAAATTATGCTTTGTAACTGAGATTAAATAAAACTCTGGTTCCCTCGAGGCC

TTCCGGGGACTTCTCCCCTCCGGCTTATCCCCTGCGTAGCAGGGATTAAACGGAAAAGTTCCGCTTCCATAATCCCCGGA

GGATAAGGCACTAGCTCCCTCGAGGCGGCGAAGCGGCGACCCTGCGATTCTCCCTCCGCCGATTTGGGCTGCGCTGCAAG

TGCCGCCTGCCGGACTAGTATCATAATTGTCAGAAGTTCCCAGCATATAGTAGTCAAATCTAAAATCCCTCTTCTTGGTA

CCGTAAAGCGCACCATCGCCCCTTGCCCCCAATGACGGTATGAGGGCTTAAAGCAGACAAGAGGGACTGACTTAGAGGGA

GAGCTAAGTGGTTGTTTATTAACCCTCCGATTGTAAACATAAATACAAACCCTAATGCAAATAACATTGAAGGTGTAAAT

GTTAAAGATCCACCGTAACACGTTGCTCGGGGTAGAGCCTTCGATCATTTCAGATCTAGTTGGCTTTGTGTTTCACAAAA

CAGTGATAGATATCTATATATTTTGCTTAAGTACTTGTTTAAGGCACTGTCTATTACTAGTAACCCGGACCATTCCTTCA

AACTTCATATGAAATATTCTACTAATATATCACTCAGAAGTAAGCATGGCGTCTGGCCTCTACGCTTTTAGATAAAATAC

ATATATATATATTTTACTCAATCCCTCCGAAGGTGGGGAATCCCGCCAGGGATTCGAAGGAATCCCGGAGGGATAGACCT

TTAATCTACCGAATTTCACTTTCGTGTAACCACTTAGTTCGACGATAATCCTTAGTAATTTATATTATTATTTCAGGATG

TCCCTCGAGTTTGCACATGTTAACAATATTAATCTCGGCTACCTCCAATAGTAGTATACTTATATTTTAATTTATACTTA

GGACCCTGAACTTATAAAGCATGGTAGGATGAATGTATGGTAGCATATTATGTAAATTCTTTTTAATGGATTTACTTTTA

ATATATATAACATAATAATTAGCTTGTTTATGGATATTACATTCTAAACCGAATTTAATAATTAATACATTAATAATGAG

TACTAATTCTTTTACTGTAAAACATTGAGTTTGTAATGTTATACCTGAATTATAAGCCCCAACTCCACAGATCCACTGAA

CTAAAGCTTCTCAAGTTAAGAGATCAAATAAATTTTGAGGAATAATTTTTTGCCTTCAATATAAAATAAATGATATAATT

CTGTTATACAGGGTAGACTTCTGGTAGTAAAAGCTAAGCCATAATGTACCTTTTATGAAGAATAGCCTTGGTAACATAAG

GACCTTTAGAACAATAATGGCTTAATTGAAAAATACTGAATAAAAATATTCAAATTGATTGTATTTTTGTTTAAATTGCA

ACCTACCATCTCCTCTTCCTTTTGAAGAGAAGCATCCGAAAGAATAATACCTATAAATACAGACATTTTATTAGTAGGTA

TTTTTATTAAAGCTCGTTCAATAGAAGTATATCTAAGAAAACCTACAGTAGAAGAAAGGTTTGAGCCGTACAATACTAAA

CTAAACCCTACTCTAGCGAAGCAAAACGCTAGTGTTCTCTGTTGGCTAGGGATTGCCCCAACTCTGAAGGTGTGCGAAGC

GATAAACACTTGGCTTCGCACAATTCATGGGGGCAAGCAAGCTCTCCGCCTGTGAAGAAAGACATATAGATCGTTTAATT

TGTTTTATCCTATTCAATCCTTCAAGTGTAGTATGAGCACCCTCGCTCTTTAAATATGCTACTTTTTTCAACATTCAAAT

GCTACAAGCTTTATTCCTTGCAAGGCGCAAGCTCCGGATTTATTAAAAATGGAATTATTTTCTCATTAATACTTAAGGAG

TCTTGTACTCTAAAGATAAAGGAATCATTTCTTTTAATATTTGTCCACAACCACCTAAAGCCAGTTGTAACCTTTGGATT

AATTTTTCATCTTTTAAACTAATATAAAAACTAAAGTTACACCTTCACCCAATTGAAGTTTATTACTCTTATAAATTGAG

ATATAAAACCATTAGAGTTATACTCATTAACGACCTCACTAACTCAGTCTAGATTTACTTGGCAGGGTCTTGTAGTATAA

TACCTATCCCTCCGGGGATTCCCTTCAGGGAATCCCTGGCGGGATTCCTCCGGGAATAAGGGCTAAAATCTCTTCCATTC

GGTAGAAAACCTTTGCCTTGCACAGAAGATGACACCAAAGCTCTCTTATCGCCCTCCCTCCTTCGGGGAGGGATTTAGAA

TATTAAGATAGCTTTTATAACTACTATTGTTAAGATTACCCCGTCAATAGGGATAGGTAGCTGCTAACTTACCATTAATA

TTGTATTTGGTCATTATATACCTCTTACTAAAGGAGGTTGACAACCAACTAAATTGGGTCGAACGAAACTGCAGCTACCA

CTAACACTTGGTTAGTCTTGATGGCTGAGCTCGAGCTCCCACCGAACCGTGCGAAATAGTTGCCCATTACACGGCTCTCC

ACCGCTACTTGACTTCATTGATTAACCGAGTCGGTTTGTTAGACCAACTGCCCCCAATTTATGAGCCGCTAGATGATGCT

TTCTACACAACGGTATTTGTTGTCTCCGAACTGCTCTAACGTGTCTCTGTATAGCATTCGTGGTTTTGGCCATATTTTTC

AAGGGGTTGATATGATGCATTTGTACGTCGTCGAATGATCCGCAGATTTCACACGGTGCTCCCCTTGACTGGATATTACT

TTCTCCAACCTTCAAGCCATTGCAGCTAGAGGATTCTTTGCTGCCGACTTTCTCTCATCCCAAACTACTTTTAGGAGGCT

GTCCTCCCCTCGGCTCCTTGCAAGGACTGCATGTACTCAGGTACTCAGTTAGGCTTTAGTTTATTTCCTTGTGGTTTAGG

TATGGTATTATATCGGTCAAACAGCACTCCCTTTAGTTCTGCCCTTTTCCTTTGGGCGTGTCGGCTTCATCCGCTCCTAC

TACCGATTTCGCCCTGCCTCCTATAGGTTTGTTCAGAGCATTTCCTGCGATTTTAAACACCGCAGCCACCGTAGGTAGCT

TGAATTTTGCGGCGTACACCTTGGCAATCGAATACCGGATTATATAGGCGGTTCGGGCTACAGCCTGTCTCCGGTTTCCG

GCGATCGATCATCATTCACTTAGCCCCTAAGAATGCGATTCACTTTTGGTTCACTTCAGATTGTGGCAATCGTAGAAACC

GGAAAGCAGGTCTAGGTGTTCCGTCTCCAGTACAAAACTGACCTGAGTCAGCCGGGCTATCACTCTCTTCATATTTACGT

CTAGAGTAGGTATGGTCATTTTTCTCAACACCTCCCTACCACTGTAAGATTGTCGTACGAAGATGGTTCTCCGGCTAAAT

TTATACCCTAAGAATTCAACACCCTTTGTGATGTGGGTGATTTTAGTTTTCTCCAGACTGAGTTCCACCTTTAATCTTTC

AGCAAGAAAATTCTTAACCTTGTCCCTTATCTCCATCGCCATAGAGTTAGGCCCTAGCACCGCTATAACAAAGTCGTCAG

CATACCGTATGTATTTACAGTTTCTGTATCCTGCCTCATTATGTATTCTGCTTGGTATCCTTAGTCTATAGCACTCACTT

TTTCTGCCATTCCTTAGTAATTTACGGGCCTCAGGGTTTGGCTTCCTACTCCCCGCCTTCACGTCACCCTGGTATTGTGT

ACAAAGTTCTTCCATATACTTGTCAAACTCGTCCAAGTATATGTTGGACAGCAATGGACTTAGTATTCCACCTTGTGGAG

TTACTAATTCGGGGATATAGACCGTTTTATCTTTTGGAAGACCTTCGCTTTAAGCCCCGTTCGGATTAGCTTTAGAATAG

TGGGATCCAGGACTTTCCTTTCCAGAATGCTCATTAGAACTTTGTGATTAATTGTCGGGAAATAATTTTTGATGTCACCT

TCAATAAATCATATAGAGTCTTTCATATTTGTGTTCATCCACTTCAAAGCCGTGTGACAGCTTCGATTTGGCCTAAATCC

ATGTGAAAGATTACTAAATTTAAGTTCGAAGATGGGTTCTATGATCGTACGCACGACCTCCTGAACCAATCTATCGTTAA

TGGCTGGAATACCTAGGGGCGAGATTTACCAGGTTTCCCTGGTTTGGGAATGATAATCTCCCTTACCCCAGTTCATGAGA

ACTCATTCTTCAAGACGGCTTCCCGCAGTTCGAGGATTCTCTTTCTCGTTAACGAGTCAAGCGTTTCTCCATCTGGTCCA

GGTGTTTTAGACCCTTGGTTACCTCTTAGTCTGAGGTACGCGCTGTATCATATCGACTCCTGTTTTAGTAGCCCTTTTAA

ATCATGAAAGATCCTCTCGGGTGCTTGGTAGCTGATTAACCAATGTTTGGCCAGAAGTCGTAATCCTTTCGGTATTTCTA

CCGGTTGCTCCTGTACAGTCGCTAGATTCTTCGTTCTGCTTCTAGTAACGTATGACCTCACTTGACCCTTGGTGAGTCCA

GTAAAGTGTTTAGGTCCCTTTCCTGCGAGAAGCGCAGGTACTACGGACCCTCTGCCATCTCAATCTGTATTCAACAGCGG

TCCATTGATTGTCCCGTTGTATCCTGCGGCTGAGACTTCACCGGTAGCATATGTGACTCTTAATTTGATGAATAAGGAGA

CTCTTTTGTAATCTGTCTTAAATGCGCTTAATAACGTCTTAACGGAGGAATGGCCCTGCGAGTAAATTTTCACCTCCAGG

GTGCTAAGATAACCATGTAACTGCTGCAGCCATGGTAAGGCCGAATTATGCCCGAAATTACTTGAGTTTTGGTAATTCAA

CCTCATCCTTACTCATCCGATGACTGTCAGACCTATCTTGAGTCCACTTAAGTATTTAGTGTCAGCTATGACTCTCATGA

TCCTTTTAGTGCAGGCTGTTGTCCTCCTATCATCGCTGATTCGGAGTAAGCCCTATAGTCTTTAACGAATTCCACTTCGT

TATTTGACCCGGAGAAAGTTTCCCGGGATACAACATTTTCACATATACTCGAACGGTCGCCCAGATTTTAATTCCTGTGG

GTCAAAATTATGAATTGTTACGTTTCCTAATACGATCTAATTCAAGATCTAAATCAATAAAATGTTGCATATAGCGGCTT

AAAGAAATTTTGGAATTTCTTACTTGTAACTTTTCCTTATCTTTTACAATCTTAGCCCAGACATTAAACTCATCTTGTTT

ACAAGAGAGTATAGGGAAAGACTAAAATGATAAACAACTTCCTACCGCCCTCCTCCCCTTCGGAGGAACAATTTTATCGC

ATATATGTTTACGTCGGCACGAGCGCCGTCAGAACGTTGATTACCGTCGCCTAAAAATATGAGGCCAGTAGAGAAATAAT

AGGAAGTTCGCCAGTGCTACGTGAAAAGTTAAAACTTGGCACCACTCGATCATAATAAGAATTTTTAAACCATGCGGGTC

CATTTTAACATTAAAATTACAATAGATTGTAAAGTAACCTGAAATTAATCATCCTGATAACTCATCAGGTGAAATACTTA

ATGAATAAGAAGGTAATAACGCAGCCTTCAGCTCGGGAAAATGAGTTGAAACTGTGGTAGAAATACCTCGGTTAAGCGCC

GCATAGATAGATAAAATCTCACCAAAACCCTCTTTAGTTTTATGATCACCAGAATACATCTTATTAATAGCTTTAGATCA

TAATTTAAATGTACAAACTTTATTAAAGTGCAAGGGGTTATTATTATAATTTTTAGACTCTCCCTGTATTCATACCGCCT

TTAATTTTTCGAATTTCATCTAACCCTTTCACGGTTAAATGATCCCTATTAGCCATTAAACGTGACACTAGGCAAAAATC

CCTATAATCCAGGGATTTTGCCCCAAAATAGGGTATTTTTCGAAAAACGGTGTAATCCGGCAATTAATATCTAATAATCT

ATTTACACGATAATAAGCAATAGAATTATTCTTATAACTATGAATAACCCGCAGCCTAAGTATTCCTTAAAACTTTCCAG

CAATAGTTTATCCCTACTATGTTGAGTAATATTAAAATTTATTTTAACCCCAAAACCGGATTTGGTATTGGATTTAAAAA

TATCAACTCCAAAGCTACCCTCCGCAGACGCAAACCCTGCCAATCAATTAGGATCTAATATTTTTTATTTGTAACCATAT

CTCTTGGCCACGGAGTTACATTAGGGAAAGCCTCTGGAGTTGATTAGGTAAACCCCTGTTGACACTAGCTTTTATAGCCA

CTATATTATCCAATCCTTCACGAGTTAAATGTTCTTTATTTGAAACTAGCTCAACGGCTCTTTTCATAGTAGGTAATCTG

CTAGCTTTTGGGTAATCAAAGGATACTTATCAAAATGATCTATTAAAACTTTTAAGTCTTTCTTGGATCCAACTTTATAT

TCTACCGCCTCATTAGCTTTTTAATGTAGATGTTTCCTACCCCTGAAAATGATTCTTAATCTGCTCTAATAATTTAAAAT

CTTTTTATGAAGAGTAATTTGAAACACAAGTTCGACTTTTCAACCGTTGCTGTAACTAACCGATTTTTCTACTCGAACCC

TGAATGAGCACTCCCCGTCACAAAATCCTGTTATAAAATAAGGATTTAGTTGAAATGGACGGTAAGCGGTCGTCCGGAAT

ATTATTCGATAATTCGCTGACAGAGTACACCGTGAATAATATCTTGGTCGTGCTTATGAATTATTTTATGACTTATTTTA

GTAGAGTATAATCTAAGTCTGCCAGATGAAAATAGCTAACCGACTTAAGTAAAACTATAGTAAATAAAAAATTTGTTTTG

AGCCAAAGCATTAATAAAATATAAAAGTAAAATAGGATATTTATTAAAATGTGGGATAATTTTAGAGATAAGATCTTTTA

CAGAACCTACTCGATATCCTACATTTTACCAGAATAGAAAATAGTTCCCACCTCAAAATAATCTTGGATATACTCAAGTA

CATTCTTATAACGAACATTAACTAAAATACGTAACTCAGCCTTGATCTCCCAACGTCCCGTGAGGGATTACGAAGTGTAA

ACTGTAAACCAAAACACGCGGAACGGTCACAAAAGCCGGTGATTCATTCTGGATTAACTTTATCACTTTTACACTTTTCT

ATAACTTTAAATTTACCTTTTGCCTTATCTCCTAAATCCCCTGCGGGGATAAAAGGAGATTTTGGGTGCGAACTATAATC

TCTACTAAAAATACTAGGTCGTTAACCTCATAAATGGACTATATCTTAAGCTTTATTAAAGCCCCACTCACGTATAGTCT

CTGAGGATCCCTCCCCACCTACCTATTTTTAGGTTTTTATATAAAAAAATTTAGGATCGATCCTAAATTTTTATCCAAAA

CTCGGCGGAGGGAGGCCCGACGTATTTATTAAAAAGGCAGGTTATTTGTTGACGGGTTTCCTGCTGATTGTCTCATCTTA

ATTAAGGATTTATTAAATTATCACATAGCCTTTATACTAAGTACTAATAAATATAGAGATTTTACAGCATACAGTGAGCT

TTTCCTTTCACTAATTCCCTTTTTTTAGAAAAAGGAGAATTGCGCTTTTATATATTTATATATAAATATATAAAGGGGGC

AATAGGTTAGGCTGATAGCCAACAGCTATAGCTTTGCAATAAGCAGTATAACCTAATAATGATAAGGCCGCACCTAGAAA

AATACGGCTATTATTAATGTGAAATAGAAGATTGACCCCAACATTCAAGATGTCATTAATCAAAATCCTTTCCCGAACCG

TACGTGATAGTTTCCCATCATACGGCTCTCCAGAGAGAAATCTGTAGCCTTCTCTCCTGTTTTCCTTCGCATAACCACAC

CATTACAGCGGGATAACTACTATGAAAACTCCGTCACCTCGGTCTTTCGGACTTCAGGTGATCCTAAATTCTTTAATAAA

ACCCATTAAATATTCCCTTAGGCCAAAACAGTCTAACCATGGTTTTTTATTAAAGTCCGCAACTACTAAAATACATGTTT

AGCCTCCTTGGGCAACCCTTCGAGGGGTAGGCCCTGGGAGGTAAAACCCTCGGAGGGTTTAGTCAGATATGTTGTGGTTT

TCGTTATCCTAGCCATAGGAATCTAGCTTGGGAGTGAACCTTTATAAGTAATCTCCCTTTTAGGCATACTGTTTTCAGTG

TATAGTTGGCCTATACTACCTTAACCTAATGCTTTACACCCTTGGGCTGAATTTTATATAAGAAATGATCTGAAAAATAC

GGCAACAACTTAGGTTTAATCTTATTTAAATCCTTCGCATTAATTTGGATTTGATGCTGCACCACTTTAAGCTTTGGTTT

TGCACCTCTATAAATAGCAGTCCCCTCCTCTCCTGCGGAGGGGCAGTATCTCTAGGAATCTCATATGAATATATAGTAGG

TTGAATATCAAACTTAATTATTAAAATATTCATTAGTAAAATAACTTCTTTTAAGCTGAATCCGTACGTTATTAAACTAA

CACCTTTATTTCGGCTAGAGCCGTCCCCTGGATCAGATGAGCCAGAGCTATATAATCCAGGTAAAAAATAAATCTGGTTT

AATGGTTTTGATGATTTTGCCCTTTTCTTGAACATAAAAAGGTTATAAATTTCGATTAAACAAGGTAATTGTCTAGTCTG

AAAAGATCAGTTATAGAAGGTTTTACCTCTAATTTTGGCTTTACCTTGATATAAAGGACCTGAGCATAAATAAGATAGCT

CATTATACACTTGTCAGAGATACGGTAAATTTTTACAGATTGTTTTAATCCTATCCGAGGATTTCAATGGCCATACTTAT

TCATTCAACCATCCGACAGTAATAAACCTACTATTATACTTTTAGCTCTAGAAGTTAATTGTATGGTATTTCTCTCTAAG

TTAGTTATTTTGGATTTTCGATAATGTGAAGATAAACCTAAAGGTTTATTTCACAGAGTTAATCTATTATTTACCAAATT

ACTTTGTTCTGCCGCACTGGTAGAATACTTCTTATATTTTTCGCCATTAAAAACGAGGGTGGAGTATTTACACTCAAGGT

TTTATTAAATGAGATAGCGTACGTTGCGGCGGTGAAATAAGCACGTGTGTCCACCTATTAAGATGAAAAGTTATTAAAAG

AAATTTTTAATCTTAATTTGGACTATTTCTTAACCCTAAAGGTAGGGTCTTTCCCGTGTAGTCTCTGAGGAGCCTGGTCA

GGTTATAAAATAAATATAAATATAAATATACCCGGGGACCCCTTGGGTCCCCTTCCATCGGGGTCCCCTCGGGAATTTGT

ATTTTATGTCATAACCTACAAAAGGGTCCTGCGAGTACTATAAGATTAGTGATTATTACTAAGCCCCTCCAGCAATTTTA

TACCCCCTCCTCGCGTAGGCCTCCTGCCTTATCCCCCGTTATTCCTCCGATTAGCGGCGGGATTATGGCAGGGGAGGACC

CTAAAATTTTATTTTAACCCTCCTCCCCTTATCCCCGCCAAAATTGCCTCCTGCTTATCCCCTGAAGGGGATTAGGCATG

TAGGGAGGGAGGGCTAGCAAGGCAGGGGAATAGCCTTTTATCTCGGTATCTCTCACAAGAAGAATAGGAGATCACCTAAC

TAAGAGAGCGCCTGATACAAGATAAAAAAGGTATAGTACACTAATTTAAAATAGTTTCTTGCATATAGGAAATTATCCTC

CATATTCTTTCAAATATGGACGACGTGGATGAGTTTAGCTAGATTCCCAACTTATAAGTCATAGAAGGGTGCATATAAGG

TAAAATAAGGTTTTAATTTTTCTGCAGACTTAACTAAAATATAAATACGATAGTTTTTCCTGCTTTATGTATTGAGCATT

TAATCTTATATCTATCGATTAAATATTGAGTAAGCCGTTGAACATCTGCATGTGTGAAACCATCTGTACAAAGATATAAC

CCTTTACTTGAACCCCCGTGAGCCATCCTGCTCGCACCAACGGGCCTAGAGCTAAAGGAGTTAACAATGATAGGTCTCTT

GGAACTATTTTTACTTTACCATTATAAAATTTAGTATAAAACTCATTTAACTTAGGTAAAGCTTTACTTAAAAGTTTAGA

CTTTTATAGGTTTTATTTGTTCTTTTGTCAATATAAGAATACTCTCTATACTTTCCGGAGGATATACTAGATAGAGAATT

AAGTACACTAATAAAATAATCCCTTTTATCTTCAGCTTGAATGAAACCGAATCTGGCATTCTTACTAGATGGACCCATTT

CTAATTTACCATCACCTAAAAGACTACCAAATATAATCTCTTTAGTCTCCGAACTAAATACTTGAACTTTACCTACACAA

GCTAGCCCGCTGCTTACCCTCCCCTAATCCCCGCCTTAGCGCTTTGCGTAGCACCTGCGGCGGGGATAGGGCAACACTTT

TTTCCCCTACGGCCTCCTTTCTCGATTTTACTCTTGAGAGGTAAGGCTATCCTCTTCTGGCGACTCTCTGTTGAAAATAA

AGAATTAAACTATCTAATTTATCTAACCCAACTGTATACATGTACCGGGTAGGTCCGGCTCTCACGAGCCTTTCCCCTCG

GAACCGTACGTGCGCCTTTCAACGCATACGGCTCGAGCCTCCTGTCTATGTTTTACTTCGCTATCCCCACCTGTCCGGGG

AGTCGTGCTTTTCATTCATAGGGATTCCAGAGAATCACCTTCTTAATATCATTAGGTCTCATTCAAGACTCCCGCGGTAT

ATTACCGTACTGCAGGGTTTCCTTATATGCGGTGAGCATATTTGAACCGGGGTTTGTTCTTGAGTTCCTCCAATAATCAA

GTCAGGACACTGATGACCAAGTCTGCCACCTTTCGGTACGAATCATATGATTCTATCCTCATCATTACAACAAGGCGTTC

GCTTTTGGCCTCTCTCATACCCACTAGTCGATATCTTCGTTTAAGGAGAGTCACTAGGCTCTTTATTCCTTTATATCCGA

CCTCTCAAAGTTAGTGAATTTGGTCTATGCAAATTATTTCCCCTCTTTCTATTTTTGAGAAACTATTGGGCTTACCATGT

TCCATTATTAACACAATTTATATTAAACCTTAGGTAGATACTATATTCCGTAGATCGTATTGACTCTGAATTGCCTGCAT

AGACGGCGTATCCCTGATCTAAATAATATCCGAAGATATTACACCTTTTACGGGACCTCACCCATATCTTCACTTACGTT

TACCATAGCTTACATTTACCCTAAGCATCTGCCATTGTCTCTAGAGCTTCAGACCCCGAATTACTTCAGGCGCATGTCTA

GATAGGGTCAGAACAGAGTAGTTCTGGAGGTATTTCACCTCATTGTTAATAATGACTTCATTGTCGCAAGATGCGAAGGC

GATTCTTAATTTCTATCTAGATTAAGGAAACCCTGGACTATATCTTATACTTAAGATTTAGAAGCATAACCTATTGGATT

ATTTTCTATAGTAAAGTAATTCATATTATGTCTTAATTTTCTAACAACCTTTAAATCTTCTGGGGATAATGGTTGCTTGT

TAACAACAATTTCAAGTATTTCAGTTCATAATTTAAAAGATTTATTTTTACTAGTTTTCAAAGGATATTTATTAAAATAA

TTACATATAATAGAAATAGTAGTTTTTTCTGTAATTACAAGAAATAGATAGTCTAAACATACTATTAGTGTAATTAATTA

AATTTTTAATTTTTATACATTTATTAATTGTTTTAAGCTTTGCTTTAGTTACTGTATTAAATAGTAAAGCTATCTTATTT

AAAACCATTTCTGCGTTTTTGATCTAAAATAAAAAGAAGTTGTATATAAAACGCTTTTTTCATTACCTATTTTGACAGAA

AAACAACCCTCAGCATCCGTAAAACCAGATAATCATGCGTCATCTAATGAAGGTTCTTTATAACTATAAATAAGTTCAGG

TATTTTATTAGTAGTAAATAAAGAATAATTAAATCTCGATGCATTATTTAAAGCAATATTTCATTTTTGAGTTGATTTAC

TCTAGCTTGTAATACAAGATTACCATTCAGTAATAAATATAATAGAAAATACCTTTGTTTTCAGATACTATGTATCTAGA

AAACTTTCTATTACCATTATTATCATAAAAATCTTTAACTATACCTATTTTAAAAGTTTCATGTATTTCATGTAGAACCT

TACTATCTTTTGAGTAATTACTAAAGAAGATCTACCTTTGTGCTCTAATATAGCACCATCACCTTCTAAAACCCTATAAA

TCAAGATAATCAATCATTATCCTCTTGACCATCAGGTACAGAAAATTCTTGTTTAAAGAAATAAGCAAAATTTTTTAAAC

AAATCATAAGTATTTCCACGTATAGTCTCTGAGGATCCTATTTTCTCCATGCTTAGCGAAGAGAAGTTTCCTGCGGATTG

GGCATTTCTATTCCCCGCCGCGGGGAATCCCCGAGGGGATTAGCATTTAAACTATAAAAGTATCTAATAAATTTCCTAAT

TTTGTATTGCATATAAAGCAAATACTAATAATAGTACATAGAAACAAAAAGGTAGCCGTTCCAGCCTACAGTGGAATTTA

TTACCATCTCCTCACGGAGAAGGAAAGCCACCGATTGACTTCATACTACAAACCCACTAGAGATGGAACCCTCACGGGTG

GATCCCTCAAAGAACCGTACGTGTCAGTCACCTGACATACGGCTCAATCTGACTACATCATGTAGGGTAATCTTGAAATT

GTTTTACTATCCAGTTCTTCCTTTTTATGATGGCTCTCGACCTTGTGTACTTTCCTATGACATCATTTGTGGATGAGCAT

CATATTCTGCATTTTATGTCTTTCACCTTTCTGGCTGATTGGTTGAATGTGGTGTATTTCTATCTCTTCAATGTTAGGCA

TTTCGTTGCCCCGTCAAAGCAGGCTCTCATTACAGATAGGACAAGTTCCCCTGTTTTTCATTAATTTTTCTTTGAATGAT

CCATAAAATCCTTGACTTAACAGACTTGCTCTTACATTGATTTGTACCACCCTTTTCCGCTCCCTCTCAGAGTGAAAGGC

GTGGATCGATTTGAGAGATTTAGGTATTAGGTATTCCATGGCAGACTGCGTGCTGACAATGTTAGTTGGATCGACCAAGT

AGATGGTTTTCCCTTCCAAGTTTTTCTTGTATCTGGATGCGGATTTAGTTTCTCCTCGAAATACCCATTTTCGATTCTTG

AATAACCCTTCGTTTTGAAGTAGAATAAGGCTATTTTCTTCTTGCCTCATCTAGGATGTTTCTTGAGCGCTCATTTTCAG

GCTAGTCTATATAAGGCTTGCCTTACATGGTCTCGGTAATTAGAAGAGTTTCCCAGGTTGAAGTAATTGGATCATCCTCT

GATAACAGGGTTTAGCTTGGCTATCAAATTGTAGCTGTCCAAATTGGTACTTTCTCGAAAGATTTCTCTCAGCTGGCTGA

TGACTCCTTTCACCTTTGCGGGGTTAGGATACATGGCGATCCCTTTTCTTCCTACACGATTCCTAAAGAATCCATACTTG

GGTTTTCAAAACTCCCTGTACCTGAAGGTATAACCTAGAAAGTTTAGAGGATCTGTTCGAAGGTTAAGAATTTTCGTCTT

CTCCTCGGACAGTCACACTCCTCTCTCATCTAGGAATTTGTCTATGCCTTTTTACTATCATAAGCATCCTTCTACTTCTT

CCAAATATTACGAAATCATCCGCAAAGCGTACGACTTTCAGCTTGGTAGATATCAATCTCCTTCGAGTCCTTCCATCGCT

GTCCTGGTATCTTACGGTCTTCTCCCCTCGCTGACTTTGTATAACTTTCTACCGCTCTCTTTGCGGCCTCTTCTAGTCCG

TCCAGCACGTAGTTACTTATAACCGGGGATATGATACCTCCTTGCGGTGTACCCGTTGTAGTGGCTTCATATTTATTAGC

ATGAATAAATCCTGCTTTAAGTCATTTGTGTAGGAAAGATTTGAATATCGGCGGCAAAGGTATGTGAGTCATAAGTCATT

CATGCGATATGTTATCGAAGTATCCCTTGATATCTGCGTCAAGCACAAATTTGCTTTCGTAGCCGCCTCTGAGAGTGTAT

CGCAGTTCCGCCAGGGCATTCTTTGTGGATCTCCCTTTTCGGAACCCGTAACTATTAGGGTCACTAGTTGTCTCCGTTAC

TGGCTCCAGTATCAGCTTTATCAGCCCTTGCATGCATCTATCTTGTATAGTAGGGATACCCTGGGGTCTTTCCCTCCACT

CGCTTTAGGTATATATACCCGTCGAACTGGTGAGACTTTATATTCGTTAGGTCGATTTAACAGTTGTAATAGTTTGTTGG

CCATCATTTCCATGTCCTTTTCGTCCTTCAGAATTATATTGTCCACTCCGGGTGTTTTTCCTCCTTTATTGCCTACTGCT

TTTGTACTGCCAGGTATCGGAAATCGTAGGCTCGCGCCAGCTTTTCCTGTAACTTATACATTTCTCTCTTATCGGATCCT

TGAGCTAATAGCGCGAGCTTAGTCTGCCTCTTGAATACCTTTTCTTCTATCGTCCCTTGGGGTTCAATAGGGTTCATCCT

TCTGCCCGTTCCTAGTGTAGACTTACATCTTATCACTACTTTCGATCCGTAGCAGGGAGTATTCCAGTTTCCTGAGCCGC

GGTATCCGTCAAACAATGTCGCATTCAGCTTATCCCTTCCATTACGAAGGGCCTTTGCTTGGGCGACTATCTTTCCCCGT

TATCGTCCAATATATTCTGTTCTTTCTTTGAATATATTGGGTCTATCATGACCCTCTTCTTAGTAAGAAGAGCCCGATCA

CCTTTGCGGTGTTTTACGAGGTGCCCGCATTTTACAATATTTACAATCCGGTCAAGTATATTATCCGGCTTACCTGTGTA

GTTTGATTCTGTAATGGGATAGACTCTGGGAAGTTCACAGCGGAGGCCGGTATTAGGCGGCTTCCTTCTCCCACTTTATT

GTCTATGTACAGAGCCCGCTTGGCAAATGACTCTGTTATTGCTGACCCACTTTTCAGATGAACCTTTGCTCTCGCTAAAC

TAACCCTAGGTAAGTTTACAATTCAATTGTAAAATTCTACGGAACGTCCAGGGGTTCCATAAAGAGTATCTCTACCCTCT

TTGCCAGCTGGTCCCCTTGTCCTATTCACTAGTCCACCGACCATCCCCTCGTTGGAGGATAACCTACTAGTTGATCCGCC

ACCTTTTATGTGAACGAGTGTGAGATCTTTCGATCTGTAAATATTGCTGTTGCGTCGTAATAACACTCCAATTGACATCA

TGGCATAGCAATATTTTGCATATAAGTTTCCTCCGGGAAGTAAATATATTTAAATTTTATCCATTGTACCGATTCATAAA

AACCTGGGAGCAATCCAATCCTACAACCTATTAGAATTCATTTCTAAATTTAAAGATTTTAATTTAATTAAACCTTCTTT

GGTTAAATGTTTTTAATTTAACCATAAAATTGCAGCCTTTTAAAACAAATATAATCTTTTGGTTTTATATTTAAAAGAGG

GTATAAATCAAAATGAGGTATTATTTTAAGTAAATAAGTAATATCCTGAACTATGAAATCACATCTTGGTGTAGATAAGT

TTGATCTTAAATTTACCACACCACAACCCCAAAATTTTATAAATAATCTCATTTGCTACGCAGAGGGGAGAGCCGGGCAA

TGTCTTTACTATGTTGAGCTATATGAAATCTACAATATACTTTTTCAAGTAAAAGATAATCTTTAGCAGGTCTTACGTAA

ATAGAAAACCCTCCGTCCCCTGCTACAAACCCTGACACTCAGTGCGGGTTTAAATTATCTGGTAAATTTAGATTTGGTTT

AGGGTACGGTAAAATATCAGTATAATACTTTGAAACTTTTTAGATATCCCTCTATTAATGGAACCATAATAAGAAAGAAT

TTGTAAAAGCCTGACTTACTTAAATGGTCTTTATTTAATATAATTTTGATAACCTTCGATCATAAAATAAAATCTAAACC

CCCTTTTGCTTAAAAGAGGGTAGTTTGTAAAATGAGGTATAATAACATCATTTAAATAGTTCACATTTGTTACTCTGTAA

ACAGATATTTTTTATCTGACCTATTATAGATAGCTCCTACACCAAAAAGACTGAATTTTGTATAAAATCTCTGCGTCTTT

TCATGTAAATTTATTTCAAATGAACCACGAACTTTTCTTAAAGAATCCTTACCCCGGACGGGAATTTAAATAATGACTGA

AAAGCAACCTTCTGCATCTACAAATCCAGTTACTCACATAGGGTCGAGTAAGACCTCTTTTTATAAAACAGTTATCGGGG

CTTTTAAGTTTTTTCTTCCGCCGGGCAAACTAGTCCCCGGACTTGTTCCCTCGGACTAGTTCCTCGGTAACTTCCTCCCT

CTAATCCCCTTTGGGGATAGGGTGGGATGGAACTAAACCCAAATTTCTTTTAAATAATTTATTTAAACTTACCACCGGAG

GGCACATCCCTTTTTTTAGGTAGTCCAAAAAACGGGGAAAAACCTGGGGAGGGACTTATATTTATTAACAAGGCTACCCC

TTGTTAATATATAAACAAAAATAGGACTATATCTTAATCATTCTATATGAAGGACCCTCCGCGTGTAGTCTATGAGGAGC

CCCTAATAAATAAAAATCTAGGTATACCCTAATAAAAGGCTTTAAAAATCTCATTCCCCTACATTAGGACTTATTACGGT

TTCCTGCAGATTGCTCATTGTAATATCCTTTAAATTTCGACTCTTATTTCCTCCTCCTCCTCCCTCTTAGAGGGAGGAGG

GAGGAGGGACAAGTATTAAGGGTTTTAGAGGTTTCCTGCATATAGCGAACATACACATAAATATTATTTAATGCGGGCCT

AATACCGACCATACCTAAATACTTTTCAATTTAATGAAAATGGACCATCTCTTTGCCAACCAAACAATTAAATATTTTAA

ACCTCTCCCCGTTCCATCCGCAAACCACCACTATATACACAAGGTGTAGACAAAGTGTATACAGGTAGGTGTAGACAAAA

AGCAGTACACAAAGTGTATTGTTGCTTAGGAAGAGATATGTGATATTTAATTATTTATCTTTAATTACAACACTATTTCA

TTTTACCAAATAATTTTTCCAAGCTTTACCCAGTACGCTATCAGCTTTAGCCGAATGGGCATGTAATTTTCTTAATTGGT

AAAATTTTTCTACTATATTTAACCTTACTAGTTTTGGGGATCTGCAAGGATTAATTTTAAAGTAATCATTAACTAAGGAA

ATAATTTCATTTTTTCTAAAGCAAGTTCATTTAAATGCACCTTGTTTAGCCATTACATATATTTTCCCACCATAAAGTTT

AACTAAGTCATCTAATAGGAAACGGTTTTTGACTAGCAGTTATATATAATTGACCACTTGCTTCGTTCATATATATACTT

CCGTCTGCGTCAAAACCCGGCTAATCAACCACTATAATAGGTTAGAGGTTCGCTATCTTTTAAGTTTATATTATATTTGA

GACATATTTTACTTAGTTGAATTACTCTTATAGGGTTACGAATTAAGCCATTTATAGCATTTATTAAATTTAGTAAACCG

GTTTTATGATGTAATCTATATCTTAAATGGTTATCTCCTGCTCGCAATTTTACTGAACCACCATATTTTTGTTTAATTTG

ATATAAACAATGTTTATCTCTTAGCTCCATAACTATCTCTAAACTAGCAAACCCTTTTTGGATAGTTGAAAACAACCGTC

CCCGTCAATTAACCCGGCTAGTCATTCGTCAAACTTATCCTGGGGATTTGGGGTTTGGGGTTTTATTAGTCCCTCCCGGA

AGTATGATTACTTTTATATTACCGTAATTATTAAGGTTTTAGATGATGATATAAGATCCTTGCAGTAAGCTTTTGTAAAA

GCAGTTAAATAGTGTTTGTCTATTGTATTTGGGAAATTTTACATATTTTGATAAAACCCAAATGTTGGCAACAAACGTAT

GGCCTCTGAAATTTCTACTAACATGCATAATCAAAAATTCATAATTTGTATTTTCGATTTAAAGTTTTCACTTTGTGTTT

TGGTTATCTGCGGATTACTTATTAATATTATAAAAATTCTTACTAGAAAGGAATACATAGTATTGCTTATATTCTTAGTA

TTAAATAATAGTGAATTGCTTATATAATTCATTCAATGCTCTTTTGAATGAAGCATTCATAAGTTCCCTGCATATAGTTT

GTTGCGTTAATTGTAATAATACAATCAAAGAGCCATTATGACCAAATACTGATTTATTAGAGCTTGCGGATATTGTAGTA

CTTATTATACCAAATCCGGGATTATTAATATATAAACCTCTGATGCTACATTTGCTGAAGCAAATGTGTGGACCATATCT

TTAAACAGATTGCCCTGCCTAATCCCTCTAATCCCCTAATCCCCTGCTGGGACGGATACGGGGATAAGAGGAGGAATGCC

CTTATCCCGTTGGGCAGGGCATAGTTAATCGGATTCATAATTATTTCATTTATTCATAAAGGTATCTCAGCTTTTGGCTA

ATAAAGATTCTCGAGGTGCAATATGAGCTTTGAGATTATTCAAAAGATAAAATTTTGGCGTTAAATGCAATCTAGCCTTT

TACCTGATCTTGAAGGGTATTTTTAAAATATTCAATTAAATTAATGATATCTTCTTTCTTAGTAAGATATCATTTAAAAC

AACCATTCCCACCTCTATCGATATAAACATAACCTCCAAACAATTCAACTAAAGGTGTAAGCATTTCACTTGTCTTTTGA

CTAGCTGAAATAGATAGTTGTCAATTAGAACCTCCATTTATAGCAATTGATCCATCTGCATCCCAAAATCCGAGAACCAA

CCGTTATTTAGAGTTAATTTTGACGGATATTTTAAGCTTATGTTATATTTAACACAAATATAATTTAATTGTATTAATCT

AATAGGGTTTCTTATCTCCCCATTTACATCATTAATAAGATTTAATAAACCTTCTTTATTATGTAATCTATACCTTAATG

CCCTAACACCTGATCTTAATTTGATTGAACCACCGTAAACATTTTTACTGCGGTTAGAGCTCGTTCATCTCTAATATCCA

TTGTAATTTCTAAGCTTGCATAACCTTTTTAGATAATAAAAGAACCATCACCGTCAATTAGTCCGGCTAATCATTGTTTA

AATCTCAAATGTGAGGGATATGAATTATTTAAATTATGAATTGATCTAATGTTTAACAAACGTATGGCCTCTGAAGTTCC

TACTAGCATGCTAAGTGCGTTGGTTACTTGCGAATTATCGTAAATTTTGGGGTTTTACTATACCGGTATACAATAAAGTA

CAACTTATAACTATAGTACCTAATAAATATAAAACGAACTCCTCGCTTACAGTCTGTTTCGAAAATTTTTCATTTAAGGG

CCACCTTTGACCGAAGAATCCAATTACTTATCAAAGAACAATATTTCACCTGTCTGTTTTTGACCTAATATTATAACTTA

AATATTAGCCTTTATGGTCTTAAAGAGTAATCCGACTGTACATTAAGCAGCATAGTTTTTAAGGAATTTCTTAAAAGCTG

CCACCTACCGGTGGGCAGTCTGTAGCGATCATTTATCTGACCGACGGTCTTGTTGAAGGACAATTTTGACCGTTTTCACC

AGTATTGCCTTCATTATTAATTGTTTATCGAATAAAGATTGCACAATAATATTTTAACTGAAGACTAGGGTTTGGTATGT

ATGACATTTGTCCATATGGGTCTTAACATTTTTTACATCTGCCCACACAAACTTCACAGCTTTGGCTGAACCGTTCCCTG

TTTTCCTTTATTTATGCTTTAATAAAAAGAGCTATCATCCCTTTTATTAGGTATAGCGCCGCTATAGTTATACTAAATTA

GAACCATTTATTTGTTTACATAAATTAGTAATTTCAACCCTTTTAACGTTATCTAAATGTTCTTTTGCTTTGATTTTAAA

AGACACTTCTTTTCATTTACGGTAACTGTTTGCTTTATTAGTTTTAAGAGTGTATTCATCAAAATATTTATATAAATTAC

TACAGTTCTTGATACCATTAACTCTAATTTCTAATACTGTTTCAACAGAATGGTTTAATACTACACCAGAGGCATTTATT

TGATTAAACAAATTGACAATATGTTGTAACACATACCTATTAGCCTCATGCTTTTGAGTTAGTATGTATCTAAACCTATA

AGCACTAGATGAATTACTTAATATGGAGCTAGTAAATGAACCCTCAGCGTCTGTAATACCTACTAATCAATAATCCGTTA

AAGTTGGTAAAACCGTAGCATAAATAGGAACAATTATGCTCTCCCCATGTTTTAACAGTTTTCGTTAAGAGCTGATAAAA

ACGTGTTGAATCTGTTAGCTCTCGTAGGAAATACCATATTACCATTAAATATAAATGAAATAAGTACTAAATTTGCTCTA

TCCTGTACGATAAACCTATGAGTGTTAGATTTAATAGATTGTTTAATCACGTTACCAAAACCTAAACTGTTCTTTATATA

ATTAAGAACATTCACATCTTCTGTAGATTGAGTTACAACAAATTGTAAGTCTCCTCTTTTAGCTACAGTAAAACAACCTT

CACCTTCTGCAAATCCTATAAATCATTGTAAAAATTCAATATTAGGTAATTTATGATTAGGGTACATTCTGGCATATTTT

GTAAAAATGGTTTAAATTCATAATCTTTTATAACAAAAGCTGATTGAGATAGTAAAAAGCTGATTGTAGTACAAATTGTA

TTACAATATATAAAAAGCATAAATAGTTTTTGTCAAGAAAAGATGCTGGTATAATATTGGGTCACCACCACCACTAGAGA

TGGGAAAGAACCTCACGGTTCAACCCCTCAAAGAACCGTATGTACAAGTCACCCTGTGTACGGCTCAATCAGGAAGAATA

ATTTAGATTTTGCAATTATTATGCATGCCAATAATTACTAATCTGCCTCACGGCTGAAGGAAGTATTCTTCGTTAGTTTT

CGATGACACACCAAATGTATAAGCCTTAGATTCTGAAGGGCGGTACGAGATCCTCCTTTCGAAATGGGTTTGACGTGATC

GACTTCTAAGTTATTAAAGTTAAGTACATCAGTGTTACCAAGCTCATCCCTAAATAATGAGTTTCCGCATACTGGGCACA

TACCCTTCTGTTTTAGACTTAGCTTACCTTTCATCCCTGCCGATTTACCGAGGGACTTGAAGTTCACTTTGGTCAGGAAT

TCGATTACTTTGGGGATGTCCTCATGATAGCCATGTATATGGAGTAGATCTTTTGGTATGGCAAAGTTCGTCCCGCCACT

GTCTCAACCACAGTTGTGGGGTCGACTAAGACCCTCTCTTTTCCGGTGTTTAGTTTACTATACCGAGAGGTAGTCTTGGT

TACTCCATGGAATGCCCATTTATGAGATCCTGGAAGCCCCATCTCGAGACCTGCTTTCATGGTTTGGGAGCCCATGAAGT

ATGTGGTGGCTATAGATTTCTTCCCTCATTTAGGATGCTTCTTGAAGGCTCATCTTCAACATTCTTTGTATAAGAAGTAT

CTAAGATAATCCCTGTATCGTACGCTCTCTCCTAAGTTGAAGTAGTTCGATCAGCCTCTGATAATAGGGTTGACTTTGGA

TACTAGTTCATAAGCGGATATATTGGAATTAGATCTAAAAATATTTCGTAGTCTAGTTTTGATAGCTTTCACCTTCTCCC

GGTTCGGGTAGAGCGCTACGCCCGGTTTGCCCAGCCTTTCCTTAAAGAAGTTGTATCTAAATTTCCAGATTTCGTGGTAT

TTTAAAGTGTAACCCAGGAAATTTAGTTCCTTACCGGAGGAGATCGAAAAGAGTCTGGTCTTCTTCTGACAATCACACAC

CTCTTTCCTTCAGGAACTGCACAATTGCAGGTTTGATCCTAAGTTCAAGGATTCTACGGCTGCTAGCTATAACTACGAAG

TCATCGCCGTACCGAACCGTCTTTACGTGGAACGTAAGCATCTTCGTTCTTCTACCGTGTTTGTAGATGTTTTACGGAAG

GCCTTTCCCCGTTATACCTGCGATTGATTGTCGAATATGTTCTTCCAAACCATCTAGGACAAAGTTGGATATGGTAGGGC

TGATGATACTTCCTTGGGGTGTACCGCTAGCCGTCTCTATGAATTCCTCCTCGAACTTACTTACCCTCCGTATAGCCCCG

CTCAAGAGTCATCCGTCAAGAATTGTTTTGTGTGTAGAAGGTAATGGGAGGTTTTTCCTAATTCAATCGTGGTTTATGTT

GTAAAAAGCCTTTTATGTCCGCGTCTAGTACTCATTTCCACTCTCGCCCCGATTGTAGATCAGTTCTTACAGCCGCCACC

GCATTTTTCGCTCCCTGTAGGGACGGAAGCCAAAACTGTTCGGATCGCTATACATTTCGACAATAGGAGCTAATACTAAG

TTCAGTAGCTGTTGTAGGCAACGGTCCTTTATTGTGGGTATACCCAAGGGACGTAGTTTTCCATTTGCCTTTGGGATCAT

CACTCTTCTTACCGGTGCCGCTCGATATTTTCCCAGTTTTTCCACATTGATAAGGATATCTCGCAGGCTGTTTACCATTT

CGGCTTTCTGATCATGGTTTTCTAGTATTATTCCATCGTAACCTGGGGTTTGGCTACCACTATTAGATGTTACTTTCCGT

ACAGCCATTGCTCGGAATGGTAAGGTCTTGGCTAATTTGGTTTGCAATTTGGCTACTTCCGAAGAATATTTTCCTTTCAT

GCCCACTAGTTTGACAAGTTTGATTTGTGCCTTTTAACCTCCTCTTCGAATGTATTTCAGTCTAGATTACTCCAGTCTAA

TGTCTTCGGTTTGGGGTTGTTAGGGACGAAGACGGTAAGTCCTGCCTTAGGCCTGTGGATAGTTGGCGTCTCCAACTTTG

TCCTTTACTAGTGTTTCCTAACAATATTGAATTCTGCCCAATTGAACGAACTAAGGACTCTCGTATTGGGTGAGTATTTC

TACAGAGTGTGTTATACCGTCTGTAGATCCCACTATCCTTGGTATTATTCAAGGCGTTCGCTTTTTCAATTATCTTCCCC

GTCTGAGTTGCATATGGCTAGATTTAACTTCGACCATATGTAAGTTGAAACCAACGTAATACCCCACAGAACATAAATGT

CCTTTGCAATCATAATGACTCTCCTCTCTCTACTTCCTAGTCCACCTTTAGGTCAGAGGTAAATCCCTAATGAGGATCCC

GAGACTGTGTGTCTATTACCATAGCGGGCGATGCATTATCTTTGTCTCCTTCCTTTAAGGGACGGGGTATTATCTCAGTA

AACGTTTTCGTATATCTACGTTGTTTTCCGAGGTTCCCATTATAGGTATTGTGTGTGTTTACAAGTAGGTTGGCCTGATT

CGCATAGCTTTCTTCGCGAGTTATTTGAAGGGCTTCTCCTTGTAGGATCGGGTTATGAGTTGGAATCTGTGCAGGTATTG

AACAAGAAGGACCCAATATAAAAGATGCAGCTGAATCTTTATGCTTCTTATATGTCCCGTCGTTATGGTATAATCATAAG

GGGAGGGAAATAGGGCTAGACTTTTCGCTGGACCCGTTTTAAGATGATTCTTAACTTTCGTTCAACTCATATCGATCACG

AAACTGTGGAAACGCAATCTGACCGAATTGTACCCGGCCATCCCGCTATTCATAGGTCCTAAGAACCCCGTCTCGTTGGA

CGGCATCCCACCTAATAATTCCTCCGCCTTCTTATCCGGGGATAAATTTGGGTCTGCGGAAATGTTTGGGTAGAGCACTA

ATATCGCACTACCTTTCATGCACACAATACTGGAATGTCGTAACGCTGCTTCGAAGAATGATGTATTAAAATTACGGTCT

GTTAAAACCATTGTTATTGCCAATTATAGGCAAACTGGCTACACTGTTTATAAAACTTGTATTAATTCTATTTAATAGAG

GCCGCCCTAACCTACGCAGAAGTATTCTCAATATTGAATAAAAGAAGGCTAACCGAGCTCTAGCTAGAGCTCTAAATTAA

ACCATATATTTCTTGGCGATGATGGCTTCAATCAAAGAAAGTCCTATTTGAATTCATATTAGTTTTAATATTCTAATCTT

TTGGTACTCTGCCAGGTCTTTATGTAATTTATTTACATAAATATTATTCGCAGATTCTCAATCCTTATAATCCAAATATT

TTGAACTCAATATAGGAAAAATATTCAAATAGTCAATTAGAATTTTATTACTGATTTTAGCGTTAGAAGTATTAACAATA

AACTGTTCATACCTATGAGATATTACTCTCTGGCCTAATTTAGTAGATAAAAATTCAGCTATTTGTAACATAACTTTCTC

AAGACTAACTCCACTTCTATCAGTTCGCCGTTGCGATAAATAAAATTTAATAGCTAAGTGTGATTTGGGATTTTCAGTAA

ACCCTTTTATAGCAAAATGCCCTTCAGCGTCGATAAAGCCAGCTAATCAAGCATTGCTACTTAGAGGACTAGTATCTAAA

GGAAGTTTATCAATATAGTAACCTTGCCTCTGAAGTTTAAAAAATCCACCATATCATAAAAGCCACCAATTTTAGGTGTT

CTTAATTTACCGTTTATCGCCTTCAATATATTGTAAACAGACTTGGTATCTTGAATCATTCACCGTACTAAGTTCTTATT

TGGACGTAAATACAAATTACCACCGAATTTATTTTTAATAACTCTGCAGATGGTCTATCTTTTAATGCAAATATAACCTC

TATTCCTGCCGCGGGAGATCTTCCTTTGAAATCTTTTAGAGCTTTAGGTGTATTAAAATGTCCATCGCCTTCAATTAATC

CCGCTAAATAAGATTCTAAATAATAGTCTGGATTTTGCAGATGATTAATCTTACTTGTCATCTCTAATCCCCAGCAAAGG

GAGATAAAGAGTGACTTTCTTCGAGTATAAATTTGGTATATTTAGTCATACTCATGCTAATTGAGGTAAAGAATCTTAAA

GAATTTGATGCATAAAAGCTTACCAAGCGGTTACGGTAGACAACTACCATTAAATAGATATATATGTGTATAAACAGCAC

CATTGTAATTGCCAAATTATTTCTTAGTTTGCGAGACGTAATCTCATGATAGTGTTACTACCTTAAAGACTTAATTAAGC

CTAGAGCAAACACTCATATATTCACATATATGTTGGGACCATATCTTATTTACTTAACCGAGGGTAACTCCCTCGTCGTA

GAGGAGCTGTAAAAATTTTGTAAGTGATCTCAGACGAATATTGTTCTTTATCATTCATTCCGGATTTAATGGATTTGACA

TAAACCATGTTAGTCTTATGGTTAAGTCTGTTTTCTTTGAACAATTGTACAACCTTTACTCAATCTTTATAATCACAATA

TTTGGTTCCGAATAAGGGATATATCGTAAGATAATTTACAACTGCTAAATTTCCTTTTAAATTCGTAGTTCTAACTCTAT

ATTGAGGATTCTCACTATCGTTTCTAATAAGTTTAACTAAAGATTCAAAACATATAGCAATTTCATTTAAAAGAAGAAAT

TATCTTTTTATTTTGGTCTTTTGTCTTTGACTTAATTCAAATTTACACTCTATTTTGGGATATTTACCAGATTCAGTACT

TCTAAGAGAAAAATGACCATCCGCTTCTATAAACCCGGATAATCATGCATTAGATAATAGAGAATCCGTATTAAATCCTT

TTTTCGAATGTGGTGTTTCTGTATTTATTATACCAATCTATTAATTTATGTAAAACCTCAATTTTATTAGTTTTCATATT

GCCATTAAGTAAATTAATTAGCAATATTAAACCTTCTGAATTATTCACGGTAAAAATATAGGCATTTGCACCTTTCTTTT

AGAAAGAGAACCATGGCCTAACTCTCTTTGAATTATAAGAGCTAAAGGTAAGTCTTTCAAATGAAAAACTATTTGAATAG

AAGGATAGTTGAGAGTACCTTTAACAGATCTTAAGGTTTTGGTACATGTATTGTACCATCACCCTCAAGGAGACCTGTTA

TATATTGAGAAAAACGTGTATTATAATTACCACTTTCGGGTATTTTAATACTAGTTCCCTCTACGATCGGGGACCTTGTC

CCCAATTGAGAGCGGAACCAGACGTTCCGCCTCCCCGGACTATTATTCGTGTGATAAAAGTCATGGCATATCTTCTTATT

GTAACTGAAGGGATAAAGCAATTATTTTCACTAAACCCGGTTTGACCTCCCTCCCTCCGGGCAAGGAGGCTAAAATTTTA

CGAGTAAACTCTGGCGTATAGCCTCAGAAGAGCCCTATTTCGTGTAAACTGGATAGGTTTCCTGCTGATTGTGTGTTATT

ATAACACAGTTCCCAGCAAACAGCCAGATTTAAAGCCGGCACAATAAAAATGAGTTCCGCAGGGATTAGTGGATAGTTAG

CTAAAACAGAACTACTTAACTCTATTTTTGTAAGTAATATACCGGCTAAGACCGGAAGCGATAATAATAATAAAACTGCA

GTAACAACCACAGCTCATCCAAATAAAGCTAACTTGTGTAATCTGATACCGGGACTTCTCATATTTAATATGGTCGTAAT

AAAATTTATTGCTCCCAATAAACTACTAATACCAGATAAGTGTAGAGCAAATATTATTTGGATAGGCGTCTCACATTATT

AGTCAACAGTGAGACCCCCAACAGAACCGGGCATGCACCTCTCGATGCACCTATTAACATCTCGTTAGAGATTCGGCTCT

TCGTTAAAAGCTCAGCTTGTTTACTGAGAGCAGTAGATTTAGATTTCTCCTTGGCCGCCGGAGCGTTGCTGAAGAATTCT

TTTGGATACCTCGATTAATTTGTTGATCGTCTTGCTATCATACTGTTCCAACAACTCGTTTCTTGACTTGTCCATCTCGA

GAAGGATTTTACATGTTGCTACGGTTAATTCTTCTTTAGTGTACGTCTTCAGTTTATTCGGTAGATACGTTTTACGCATT

TTACGATAAAGGCTGATAGAAGATGCTTTCTCCTCCTTTGTCTTTATGGCATGACATGTCACGTGGACTAGCTGTAGGTT

GTCGTGGTTATTCAAGATTTTTATTAACTCCGGAACATTACCCGCAAAAATTTTGGGGATTCTGTGATCCACGTGGACTC

CCTCCAACCAGTTTCTTTGTCGACTGTTGATAATGCTCCCGATCGTTTTAATGTTTAGTTGCTCTTTGGCTATCTGGTGG

TTGGCTTTGGTTTCATTATCGACAGTCTTATCTGTCTTAGAAAACGTGCAGCAGCTATTGTGAGCCAGGGCACTATCGAA

AGATAGGTTATCCATGATGGTTTCGATGTTGCAGTTTCAGTTTAATAGCGAGTCTCAAGTGATGAGAGTTTTCTCACATA

ACGGACAGTTATGTTTTGTTTGAACAATAGTGTACTTTGGGTATCTTTGCGATATTTACCAATTAGTAAGGCTCTTTTAC

ATACGCAGTAGGATTCACCAACATCGAGTTCAAGGTTAGTTCCTTGACCGGAACTAGATACCTTCACATGCTAGGTATAT

TCAGTTTAGTTAGGCTAAGGATGTCCAGGGACGATTTCCTCTTCGTTCCTTTCGCTGCAGTATTATCGTTAGATATTGAG

GGTGATTCTAGTCATACTCTATGCGTGTCAGACTTTTGGTGATAGAAAGATCTTGTACTGGGCTTATCTACGACCATCGT

GAAATGATTTATGAAAGTATCATGGAAGCTATGTTGGTATTTGTTCATCACAAATTTACGTAGCCGTCTTCAGATGTACA

CGTCGAGCTGACGACGTAATCGCTGCTGGTGAGGAGCTGGAGAGAAGTAGTTTGATCATCCCCTAATCAGTAAGTTGATC

TCATTGAATACCTCGTATAATGACTTATAGTTTGAGGTATTTGCCGTGATATTTTGATTTTCTCTCTGAATTTCTTAGTT

GATTCCGCTGAAGGATAAGTGTATGTTCCTATTCAATCAAGTATCTTACCTGCCCTGTGTTTGGATGTTCCAATCAACCA

GTTAACTTTTGGGGTAGATTAGGTGATGAGTCCATCCAAGGAAGTCTACAGATTCGGACATTTTTCATGGAATGATCTGG

GTCTTCTGGGATAGGCTTAACCCACGCGGTAAGAGGAATTCCGAGATGGAGTGTTTTAGATGTTCTGCCATTATCTCGGA

CTTCACACCTACCAGAAAGTCATCGCCAAACCTTACGAACCAAGTCGTGTTGTACCATTCAATTCTAGCTTGGTTTCTAT

AAGCGGCATCAGTCTTGATGGGTTTTCCTTCCAATAGATCCGGGTCTTTCCGTAAGGCACGCTCAAGACTATGAATTCCA

TCTTCGCTCCCTTTGGTTTGGGCCGCGGACTTTACATGATGTTGTAATCCGTCTAGAGTCCAGTTCATTAATAGAGGAGA

TATAATTCCTCCTTGGGGTACTCCCATCTGGTTATCCGATTTGTCTAGAACCACTTTGTAGTGTTTTACATTCTCTACTG

TGTAGTTACTAAAAGGATTCTTCTCAGTGTCTAATTCTAGTATGTTGGTTTGAAGTATCCGTTTTAATAAGTATTCGTAG

CCAATGGGCATTGGAACATTATCCATAAGTCAGTTATGATCTATGTTGTCAAAACATCCTTTGATGTCGCCATCGATGAT

GTATTGAGTGTCGTATACTATCTCCTTACTAGTCCCGCTGGAAGCCGCGGAACTTATTGTTCCGCTCCCGTCAATTGGGG

ATAAGATCCTGAGAGGGAACTAGTTTTGTGGGGCCACGAAACCCCCGTTTTGGTCCCTCAAAACCAGAAGAAGAAGTTTT

GAGGAGGTACGAAAACCACTAGTCCCGGAGGCGCCCCAACTTGTTCCGCTCTCCCTGGGGACGAAAAAGGTCCCCGATCG

ATCGCAGAGGGAACTTTTGTGGGCCACGAAACCCCCGTGTTTGTCCTCTGCATCCCTCTCCGAAGGAGAGGATGCTGCTG

CAAACCCTTTGGGGTTTGAACTTTGAGGGAACCCACCTCGATGAAAAACTCGTTCCTGAGGGCAGCACAACTACTGGTCC

CGAGGGAAAAGTCCCCGATTATCGGGGACTAGTTTTGTGGGCCACGAAACCCCCGCCCGTTAGGGGTCCTTCAAAACGGG

TTTCGTGAGGGACGAAAACCTAGTCCCTATCCCCGCGCGGGGATTAGGGAAGCGGAACTTCTGGTTCCGCTCTCAATCGG

GGATAAGCACCCCTCCGGCTGCGCCGCCTCGAGGGAACTAGTATTAGTGGCTCCCCTTTCGTATAGTCAGTCCGGCACTA

ATATCTGTTTTCTTCGCACAACGTTATCACCGTATCCTGGTATGGTTATTTTTATGTTATTGCTCGGGTCGATTTCATTT

AGTGGGATCTTCTTTCTGTCTACTATAGAGGGTATATCCTTAAAGAAGAGGGAGGATCTCATTTGGGTCTCAAGGTAACC

TTTGTCATTTAGACTGAGTTGTTTATTGGATTTCTTGTATTGGAGTCTATTATTTATGTACGCTGTGGCTTGGTGACAAT

TTCTTCCGGGTCTAAACCCGAATGAGAATTCGTCTCCTAAGGGTTCTAAGTATGGTTCAATGACTAATTTTACTAGCATT

TGTAGGTTTCTATCAAATATGGATGGTATTCCCAAAGGTCTCAGCTGCTTACCATTCGCTTTAGGGATATATACTCTTAG

AACATCTTTAGGTTTGTAATTAAGCAGACTATTGTTCTTAATGTAATTACACAATCCGAACTTTAGCTTGTTGTTATGAG

CTTTGACCAAGTGGTATTCATTACTTATGAATAGTATTGGGCTATCCTTCATTGCTTTCATCTCTTGCCTGATCATCTCG

ACATATAATCTACCTTCAGCGGTTTGTAGGTGTCTACGTAGTCTTTCCGAAAAGTTAAGCTGGTTCACCCCTTTCTTTGG

ATTGCCTGATCCGTACGTCCCTTCTGGTTTGATATTATCTCCCCAGTCTGTTATATCTCTCGGATAGGTATTGCCTTGCT

TCTTCTTCTTGGTCCCCGAGAATAGTCTAGGGAGTTTCTTGAAGGCTATTCCGTCTGCTGATGGGGTTTTCGCCCCGTCC

GTCTTCTCGGTCAACTCAGTCGCTCATACTTTAAATATGATATTCTCCATATTCGCTGCACTGGCTGTAAGAATCTCTTT

TCAGCATTCGGGTTCATTCAATTTTTCTCGGATTGTGTTGGAGAATCTGCTGTTTCTGGCATTGTATGCAGTGAACTCCT

TAGATCTGACTATGGTTTTATCGAGGTGCAATGCCCTTTCTTGTAGGTTCTCTAGCTTGGCGATTGTATCGTCTGCGCTG

AGTACTTCTTCAGGACCTCCCACCTCTTTGATCTGTAGATCAGATAAGTCAACCTTGATATCTAGGTATCTTGTGAATTC

TTTGGAAGATTCTATTGCTGTGATGAATGTGCAAATGGTGATGTAAAATTTCACTGCTTGTTTGATAAGAGATGCATTCT

TATTAGGCTCATCAAGTAACCAGACAGTATTGGGTTCCTTTTGAAATCCTTCACATTCTTTTCTAAATGGGGCACCAGAA

TAGCTTCCAGTTCTATTTTTATACTCATAAGTTCCCTCATGGCATCTGGTGTACTAGAGTATAAACGTCTTTGATCCGGA

GAGCGGTGCATTCCCAATAGATTGTCTTTTAACGTAGTGTTACGTCTAATAATTGGATTATTAGATTTAGCACTATCTAA

TCTCTGTATCAACACACTCCCGTGTTGGTAGCGAACCTTACCAGCCCTCAAGGATGAAAAGGTCCAACCCTCTGAATTGA

GGCTTAGAGAATGGTCCCTTGCTATGTAATTCTGCTTCTGGCCATTTCAGCTTATAAGGGCAGTTATGGACGTGGTTGTT

TGCCAACTTGATTTCAGTCTCTTGTTTACTTCGTGCCTGTTTGTCTCAGTTAACCCATCGTTCTTTAAGTACAACCTTTC

GCTGAGGTATGTACCATGGTCGGTTTTATTAGTCGGGTATCACCCTACTGTGTCGGTATAGACAGTCAACATAACTCAAC

TTTCTGAAATCGTAAGCTCATCTTTTGCTTGACTATTTGTCGATCCGTAAAGACTCGGACTAAACAAAGGAATTACTAGC

AGTAACCCGCACGCTAAATCTACACTAGGTCCACTGTGACTTTGTACTCCACTTAAAGGAGGATACATTGTTCATCCTGT

CGGATTAAATAAACCTTTCCAGTTTCATTGTTATTATCCCCTAATCCTCCTTTTAGGAATCCTCCCGCAAGGGAATTACT

TTAAATAGTCCGGGGTAGTGTAAAATCTATTTAAAGAATCTCAATTATATTGAGTTCTTAAGGTATTCATGCTATTTTTA

GTAAAATTAATTGATTAGTTCCCTCTAGAGTTTTATATGATTTAGATAATCTAATTTTATGTATTTCACTTCAAGATAAA

AATCTTGATGTTTAGAACTAAATAAGGGGTATTTAGACAAGTAAGTAATTAGTTTTTCACTAGAGCTCTTTTACTTGTTC

TAACTTCATAATTAATCTCCACAAAACTTTCTCTAATTCTTTTTATTTCATTCACAGATTTTACATCTAGAAATTCTCTA

ATATTTTCCATTATATGTTTATTGGAATGTTCTTCATTTGACGACTTTGGCTTGGCCACTGTCGAAAGATTAGAACGTTT

ACCATAAATGGCTTTTGAGATATTCTCATGTAATGTCTTATTTGTTCTGCTATACCTTGTGAATTTACACTAAAAGACTA

TAAAGTTACCATCTGCTTCAATAAACCCTGTCAATCAAGGATTATCCCCTAAATTACTAGTATCTTTTCCTAACTTAGTC

AAAAATTTAGGTTTATTTGAAGAAAACTTTAATGAATTTGACCTAGCATTTAACCAGTCAATCATTCTATGTAGAGCTTC

TATTTTGGGAGTTCTCATTTTACCGTTAAGAAGTACAGCAATCTTTTCTAGTGAAGTTACGTCTTGAAACAATAGGTTTA

CGTATTTGGTGTTTTGGGATACTCCAAAGTACCACTACCAAATATTACTTGCAATTTTATTGCTAAAGGCGCATCTTTGT

CTACAAAAGTGATTTTTACCTTGGGGTATAATAACTTACCCTTTTGATTCCTGTTAGTTACAGGTACAATTATTGAGCCG

TCTCCTTCTATCAATCCTGCGAAATAATGACCTAACATGACCAGGTTTTATTTTCTGGTTTATTAAAATCTCTCATGTCA

TTATTGTTGTGTGGATTTATATGAAATAAACGCCTCTTAAAGGAAAACTTTGTTATATTATACGATTTAAACTTCAATAG

TTATAAAATAAAGTCTATTTTGGATTTATATTTACATATAAATATGGACTATATCTTAATCCATTTCAATAAATGGATGT

ATCACGTATAGTCTCTGAGGGTCCTACTTAGACTAAGTCTATGAGTTTCCTGCTTATTTTCCCTAAACTTGCAAAGTTTT

CCGCCACTCCTCCCCTCGAAGAGGGCTAACACCGGGTTCTTGTCGTCATACCAAGATGGTGGTGAAGGGTTTTCCAGCAT

ACAGTGATATTCTACATAGCAATTACTTACTATTCGGGCACAGTTAAATACCCGCACCATTTTCAATACCACTAGCAAAT

AAAAATAATATTAAACTAGGAGGTAATAGTAGGGTCGACAGCCCGCCCCATCGCTGGTGACGACTTGCCTCCCTCCGAAC

CGTACGTGCAAGTTTCCCGCATACGGCTCTCCCTTCTCGAGATTAGAACTCTAAGAGGACTCACTTGCGAAAGTAGTAGA

GTGTTATTTTATCGTTTAATCCAATTTTCCTCCTATGGCTGAGATTCCAAAAGAATTTTTAATATTTCCCCGAGAAATTA

ATTAACCCCCTAAACCCCCGAATTTGTTAATATTTCCCCGGAGAAATTAATTAACCCCCTAAACCCCTAAATTTCCCTCC

ATATTTCCGCATTGCTCCCGATTTAAGCGGGGAGAAGGGATTAAAAATTGGATCCCTCCCCTCATTTTCCCCATATTTCC

CGGATTGCTCCCGATTTAAGCGGGGAGAATTAAGGCTCGAGATAAGTCTAAGGTTTGTTGGGTTACCTTCCTAAGATGTA

AGACATGCCCGTTAAGGGAGCCCCCGATGGGTTCTCATTTTCGAGGGGTTCCACATATTCAACCCCTTCAAACCATCGGG

TCGGGTCTGCTTTTAGTTTTGGCGGGACCCCCAGCAACTGGTTTGCGCGTCCCTCAAAAAGTTCCGTTGAGTTGAGTTGA

GTTGAGTTGAGTTGAGGAGGAACCCCCTTATCCCCCGTAGATCCCCGATTAAAGCAGACCTCTTTTTAGCGCCGGAACCT

AGAGGGAGGGTTTTAAATGGAGATCTGGTTTATATAGTCCCATGGCTTGAAGATTTCCATCCGCAGGAGCCTCTAATTTT

CTCCCAAACTTCTTGAAGGCTTTAGCTCGATTACCTAAGTTGAACTTACGGGCTAAAGTTTTAGCACAAGAGTGATGTAA

AAGTAAATTATAGAGTGGAAAGAATAAAGGTTGTCCACGAAACCGTAATAATTAAGTAGCCCTCTAATAACCGCATTATA

CCTTAGAATTATAGATCTATGATCTAAGAATATTCATTTGGTTATAGCATTAGGAACTAGTTTACTAATACCGTCCTTAC

TAATGTAAGATTTTATGTATCCTGCGCTAGTGAGTTTAGCGAGGATAGTTTTACTGGAAGGTAGAATAACAGTCTCTTAT

TAATTCTGGGGGTAGGAGGATTCTAGATTTAACTACTCTTTAACTACCCTAGAGTAAGAAACTCTTGGGGATTCTCCCAT

TGGGGGTCTTTTAATATCAACACCTAAGAATCTTACAGAATTTTTAGTTACATTAGTAATTTGGGTTTTGTCATCACTAA

GTTGCACATCTAATTTCTCTGCAAGGAACACTTTACATAAGTCTGAAATTTTTGAGCCATCAGTCTATCCCCTACAAGAC

CTAACATTCAATCATCCGCATATCTAACATACCTTATTCCAGGGTAATCATAGGAATTCCCCAAGCCTGAAGGGCTTGGG

AATCATCCCCTGCGTAGATCGGGCGGGGATAGGTGGAGATGGCAATTCCTTTCTCTTGCTTAGTTCTTGGAGAATGTCTG

GGTTTTATGATACTCGTGAAGTTTATACACAAGTGGTTCCTTTGAAGAATATATCATTTGGGGGTTCCTAACCTGATCGG

ATCGGAGATATATTTGGAGGAGAATTCTTGGATAAGAAGATGTACAAACACATCAAATTCATTTAGATAAATATTAGAGA

GGATTGGTGATACTATACCTCCCTGGGCTACTCCCCGTAAACGTGTTATGTTTTATTCCACCATCTACATAGCCGGCTCT

GGCAAGTTTTCAATATAGATCTATAAATCTTTGGTCTTTGATATGTTTTCAAGGATATTCGCCAGAATTGTATGATTTAC

ATTGTCAAAGAAGCCTTTATATCTCCTTGCACCATTCATGTAATTCCATTCCAGTTAGAGACCTCTTTTAGCGCAGTGTG

ACAACTTCTACCTAGTGGCCTCGTCTTCCCCACAAAGGGGAGATACCATCTGACGACTTCTTACCTTCGAATATTACTTC

AAGAAGTATTCTCATAGCTTCTTGAATAATTAGGTCTTTGTAGGTATTGCCCCGGTAAAACCAAATGACCATTTTCGCTG

CAGCAGCAGGCTGGCGAAATTTTTTGGTTTACTCTGGTGAATTTGAATCTCTGACTTAAGCTCTTGGATTATTACATAAA

TAATCTCTTGATTAAAACCATCTCCCCGCGGGGTCATGTTAGACCATCTCCGCTTGTCATACTAGACTTAGCCCCTTAGA

CAAAAGATTTTTATAAGATCTAATTAAAAGCGACTCTGTACAGATTAATTTATAAAGATTAATCTCTTTGTCCGAAAAGG

GATTTCTAATTTCATTTCATTAGTCTCACTGTTACCAGCGGAGACCATAAATGATCTCGAGAACTGGGTCACTTTAGTAC

TTGCTTGCATTCTAGGGCAAGTACCTAATATTAACCCTCCGTCACCATAGGAATTGCTTCCCTTAGGCAATCTCGTAGTT

GATTCCTGATAGTACATGATATTTTTAGGTGTGCTGCCTACATCTTGTTGAATATGCTTATTACTGCTTCCTTTAACTAG

TCTCTGATCTGAACTAGTTAACAATAATATATGGACTTGTTCCATAGGGTTCAACTGGCCCCAACTGACAGTATTAATCA

CCATGAATATCTTATCTAGCTGACCTTCAGAATGGTCGAGGTCCGCCTTTGTAGCATGCTCTTGTCCCTCAAGGATTTCC

CTTTTGGTAAGCTACATGATTTACTGCCTATAATCCCTCCATCCATCGGGGAGAATTGGAAGAGGGCAGACAATAATATC

TCATTCTATTCCCTTAGGATGAGGGTGAATTAGCCCTAAGACCACCACCGTTCCCGCCGATAGATGGTCATAAATAAAAT

GAAACTACTGTAGCCTATCAGTATCCACAACGACGCACATCAGAAACTAATATTATTAAGTCTAGGAATCTTTTACCGAT

TAAAAGCCAAATTTACTTTTAACCCTACAAAGTTATTTAACTCCATTGTAGAAGTAGTTTTCTACCTGGACGGGCTATGG

CTTCATCCAGTACTAAGACTGAGCTTCGCGTGTAGTCTCTGAGGATCCTACTAATAATTAATTACTTTGGTTTCCTGCAT

ATTATTCCCTTGTATACATAAGTGGTACGACTAATTCAGTTATATTTTAACTTTATTAATTCCCTTGCGGAATCCGTCAG

GATTCCTAAAGGAATCGGATTAGATTTACCCCAATTCTTCAAGGTGTCGTCTGTATCTGTTAACTGGAAGCTAGCTTCCA

TAATTCGAGAGATTCTATGCATATTGCGTAGTCATAATAAAGATCTTTACAAATCTCCACGGCATTCCCCTTTTTATCAG

AAGAGAAAAAATATATTTACAATGAGCCTTTTCCCCAGGGGAAAGAGTAAAACCCCCTGGGTTTAGCCTTTTCTTCCCAG

AAGAACAGAAAAGGAGGAGGCCTAAACCTTTAGCCTCCTTTTCAAGGAGCCTTTTTCCCCTCCCGGGTTTTCCTTTTCCT

CCTTCCCTGGCGAGAAGGAGGCTTTTCAACAAAGCCTCCCCTCCAGAGGGAGGAGGAAACTTACAGAGGAAGGGACTTAT

AAAAAGGCAAGGTCAGGGTAAAGGGGTTTAGATAAGTAATTTATCTAAATGGTCTCAACTAAATTCTGTTCTAGCTCTAT

TCATTCTACTTTTCAATTGGTCTATTATTAAGATATTAGCATCTGTGTAATGCTGATTACTTAACATTACCGCTTTACTT

CAATCAATATAATCTAGATGTTTAGAAGTGAATAATGGAAAAACTTCGAAATAATTAGTAATAATTCTTAAAGAGTTACG

ACTTGATGCAGCTAAAGTGAAATATTCATTACCTGTAGATATCTGTTTTCTAGTTAAAGATTACAATTTAAGAAACCTGC

TATTTCATGCATAATACTGAAGTAGCTTGCATTAGTTTGAGGTTCTATCATTTTGTCTATTCTTAATCTACAAGACACTT

TTCTTTTAGAGCCCCATTTTCTTTTTAGTATGTTGTATAGAAAAACTCCCATCTGCATCCACAAAACCTGCTAATCAACT

ATCATTACTTAAACCTCCTTTTTAGGCAGGTTTAGGGATATTTGATCCATGATTTTGTTTAACCAGTCTATTAAAAGATA

AAGCTGGTGAATTTTTGGTGTTCTTAATTCCCCATTAAGTAAACTAACTAATCTTTTAAGCCCTTTTACCGGAGAAACAG

TTAATACACAGGCATTATCTTTAGGTTTATATCTAATATGTCCATATTTTATTATATTAAGTAAATATAATGCTAAAGGT

TCATCTTTTAAATTAAAAGTAATACAAAATCTTGGATTATGTTTTTTTTTGGGATATTCAAATATGTCCATCACCTTCAA

ATAAGCCGGCTAGATAACTACTTTGTGTAAAGCAAACTTGTTTTCGTAAATATATTTCTCTTCTTTTACAATGGGTCCAT

TTTTTGCCATATCTGGACCCCAATCAATAAAAGGTAATAAAAGTTCATTTAACGTATAATCTTTCGAATAAACAATGGAC

TATATCTTGAACTTAAATAGATACTATTTAATGTTCCACGTATAGTCTCTGAAGATCCTACGTTATCTTATTAAGTATAT

TTATCTTCGAGGCCTAAGGCCCCTTCCTACTGCCTTAAAGACAATCCTGCCCCGTATTATACAGGTCATTGAATAAAATA

ATTTGGTTTCTGGCGGATTATCTATTATTTTAGCCCTATTTACATGGGCTAAAGTT

>lcl|tig00000003 len=155673 reads=22064 covStat=1.00 gappedBases=no class=contig suggestRepeat=no suggestCircular=no

Apple powdery mildew Mitochondria genome 2

TGGGAGGCTTCCTCCCTCTTTACCAGTTATCCCGGCCTTTGGATCGGCGGGGGATCCGGGGTGTCATAGAGGGGATCCCT

CCGGCTAAATTTACCGAGGGGAGGAAGTTTCCAGCGTGGCCGCAGCTCAACGGAACTTTTAAATTCTTGAGGGATCTGGG

GTAAAAGAGAAGGGGTGAGGCCATCCCTTAGCTTATCCCAGGAGGGCAGCGTAGGGGAGCCTAGATCGGATCGATCGATC

GGGATCGATCGATCGATCGATCGATCGATCGATTGATTGATTGATTGGGGATTAGGAGGGCCTTCAACGCAGGGGTAATC

CCGGGCCTGCCGGGGATGCCAGCAGGGGGTCCCTCCGGGCTCGAGGCGGAACCAGGCCTCCTCTAATCCCGCCTTGGGGG

CGGGATAGGGACAAGAGGGAAATGTAATATAAAGGAATTAGCTCAATGGTAGAGCAACCGTTTTACACACGGCAGGTTAG

GTGTTCAAATCACCTATTCCTTACATGTTAATATTATCTATAATTTTTATTATTATCTAATGCCGTTACTTTAAGACGAG

ACAAATCAATATTATACAGTAGAGTTGCTATTATAGTTTTACTCTATTCATCTATTATTGCTATAAATAGTTTATTTATA

TCTAGTTTATATAAAGGTATTGGTTTGTATGGTGGTTTATTTCATGCAACAGCTATTACACAAAATTTTCATATCTTCAT

TTTATTGTTAGTGCAATTATTATACAATTAACTGCTTTTATCCTAGAAGAGTTTTATTAGATCTTTATTCCTCTTTAACT

TCTATTCTGCCGGCGGGGAGGCGGGGAAATAGGAAGATTTAAGTTAGAATACAAGAATGATAAATTAATAGACAAAGACG

GAGAACAATTTAAAATAATAGAATACCCTGGATATAGTTAATATTATGGGGAAAACTGCCAAACTCGGGAACCTCCCAAA

GCTTTTGATACCAAAACAGGTTTATTAATTATTTTCCCAGGGTGAACTAATTATTCACGTATGGTAATAAGTCAAAAGAT

GGCGCTTCACTTACACTGATTAAAGTGCACTAATGGTGATATATGGGGACCCCTAATGCTGTCGAGCTACAAGCTCGAAA

AATGGACAATCGCGGATCTAAGTCAAACACTATAATGTTTGTAAAAGAGCAACGAGTAGACGGTAGTTACACTGCGCTTC

GCGCAGTGTTAAGGTGTACTCTAAGAGGGTTCGAAAGAATTACCTGAGCCGGAGTTCCTTCTAATAAAGGATTATATTGA

ACTACTGTGCGTAACACGGATTCGATAAATTTTCAACCTAAATTATATAAATACGGAGATAGTTTAAACCCTTGATTCAT

AACAGGGTTTACAGACGGGAGATGGTTCATTTGCTGTTTCTATTGCGAAGAAAAGTCAGGTATAGGCTGAAAAATTCAAC

CAATATTTACCATAGGATTAGACCAAAAGACTTAGGATCTATTGGGGCAAATTAAAGCTTACTTTAAAGTAGGTAATATC

TATACTAGTAGAAGAGGGATAGTTTACTACACTGTGGGTTCTACAAAAGATATAATAAAATATATCTTACCTCATTTTAC

AAATACACTTTAGCTACTCTAAAATTAAAAGATTATTTAGTATTCAAGGAGATAGTTCTTCATATGGAAAAGGGTAACAT

AAATCCTTACCAGGTTTATTAAAAATCTTTTCACTAAGAGCTATTTTGAATAAAGGGTTACCTGCCTTTATAAAAGATGA

ATACCCAGATATTATCCCAGCTATTGAACCAGAATTTAAGATACCCACTAAGTTAAATCCTTTTTATTATCGGGATTTAT

AACAGCAGAGGGTCTTTTTATTTCTCTTTATTCTAATGAGAAAAGAAAAGCTGGATATGCAGTGAGTCTAGTATTTAGTT

TAAGCCAACATACTAGAGATATAGAGTTACTAGAGAAACTTGTTAAATACTTAGAGTGCGGTATAGTCCGAGAAGTGCCT

AATCGTGATACAGCTGAATGAGTTATAGGAAATGCGATGATATTAATCTAAAACTAATTCCATTTTAACAAAATATACTC

TTTCAGGTGTAAAACTTTTAGATTTCGATAGATTTAAAAAGTATCTTATTTAATAGAAAATAAAACGCATTTAACTTCAG

AAGGAGTCGTCGCAATAAAAGCTATTAAAGACGCAATGTATACTCGGTAGTTGTGTTAATATAAGGGTTAACCAGGTTAA

GTAAAATTAGTTATAGCTCGGCTCGGCTCGGGTCGGCTCGGCTCGGGTCGGCTCGGCTCGGGTCGGCTCGGCTCGGGTCG

GCTCGGCTCGGGTCGGCTCGGCTCGGCTCGGGTCGGCTCGGCTCGGGTCGGCTCGGCTCGGGTCGGCTCGGCTCGGGTCG

GCTCGGCTCGGGTCGGCTCGGGTCGGCTCGGCTCGGCTCGGCTCGGCTCGGCTCGCTCAAAGTCTAGTGCCTTCCCTTGC

TTTGCAAGGGGAGGAGCTGAGCCCTAGCAAAGCAGGGGTAGTCCCCAGGGGACCCCCTGGCTGACCCCTACTTCGTCCGC

AAAGCAAAGGGGGTAGTTATATTCATTCCTAATCCCTTAGGAGGTACGCATGGGCAGCTTCCCTGTCTGTTTATTTTTAC

CAATTAATAGGACCTTGGGGACTATTAAAATATACAAAAGATAGCTTAAAAGCTACTCATTATTTATTTATTTAGAATTA

GATAAGATCCGGTTCTCAACGTTAATTTTATTATTTATTGTCTCGGGTGCGATCTTTTAATTTCAACTAGTGATTTAGTA

TGGGCGACCGTTCGAGTGTATTTGGAAATATTAGTTTTACTAATTAACTCAGAAGAAAGAGTTAAAGACTATCGATCTTA

CTCCGAGTTGGCAACAATAGGAGGACAGTCAGCCTGATCGAAAAGGGACTATGAGGTGAGATGTTCCTTGAAACCAAGAG

TTTTAACTTGAGATTTTAAACCAAGAAGAGTCCCGAAGTCATACATGGAAAGATGGATAAGACTGCATTGTCGAAGACTG

AGTAATTTTGGGTTGATCACGACCTATTCTAGAACGGAACATTTACTAGAATACCTCATTAGCCGATTGATGGATACTAG

CAACACGGCCCTTTCTTGAAAGGAGGCATTACGTAACGCATTACGAACAGATTACCAAAGGCTATCCAATTCCACATGAG

CCAAATATGCTACCGGTGAAGTCTCGAAGGGAGAATTCCTCCGAGATGGCAGAGGGTCCGTAGTACCAATGTACATTGGA

AAGGGACCTAAACACTTTATCTGACACCAAGGGACACAGGTAAGGTCATACGCTACAAGAAGCGGAACGAAGAAATTGAC

TCCCGTAGTAGAGTCTAAAGATTTCCAAATTCTAGCTAAACATTGATGAATATGTTACCATAACAGGGAGAAGGTTTTCA

AAGACCTCAGAGGAATTCTTAAGTTGGAGAGTATATGATTTGCGGCTTACAACAAGCTGAAAAGAACAAGGGATCCAAAA

CCCTGGACTAGACTCAGTAACGATAGAATCCATAACCAAGGAACGTATCTTAGAACTGCGCGAAGCGGTATTACGGCGAA

AATTTAACTGACAAGGGATAAAACAAGTTATGATCCCAAAACCAGGTAATCCGGGGAAGACGAGACCATTAGGGATACCA

ACGATCAATGACAGACTGGTACAGGAAGTAATCAGATCCATCATAGAACCGATCTTTGAACTAAATTTCAGCGGTCAATC

CCTGGATTCCTACCTAACAGAAGCTGTCATACGGCTTTAAAATGGATGTACACACAGATGAAAGATTCTACGTGATTCGT

AGAAGGAGATATTAAAAGTTACTTCCTACGATTAGCCACCCAAAACTTATGGAGCTAATTCAAAGAAGAGTCTCCGATCC

CATCATACTAGAGCTAATTCGGAAGGGTTTGAAAGCGAAGGTCTTCCCCTATGGTGGTGAGGGGTATATACCAGAGCTTG

GAACTCCTCAAGGAGGGATACTTAGTCCTCTTCTCTCGAACATATATCTACACGAATTGGACAAGCGTATGGAAACGCTT

GGGAAGGAGTATCAAGGAACAAAGACGTCTAAGAATCGTAAGAGAAACCCCTAGCCATGAAACTGTTAAAAGCGGGAAAA

AAGCTCATACTACGGAAGGAACATATCCTACTACGATCCTAACGACGGAGAATATATAAACTGTAAATACGTTCGCTATG

CTGACGATTTCATAATAGGAATAGCAGGAAATCGAAAGATGGCCGAGGAAATCCGGGATAAAGTCAAGGCGTTCCTGGAG

GAAGAACTAAAGGTGGAGTTGAGTTTGGAAAAACCCACATAACGCACATAAGCAAGGGAATCCCTTTCCTAGGGTATAAA

TTTAGCAGAAGAACAATATTCATAAGACAAAGGTATTGAGATAAAATACGGAAACGTCGAATGAAAATACCGCTACTGTC

GGTTGATATGCAAAGAGTTGTGAAGAGATTGGCCCTAGCCGGATATTGTACAAAGGGGCGAACCTCGCCCAAATTTTAAG

TTCCTACAACTCCCGCAATCAGAGACAAATCTGAAGATGAATCACATATTGAGAGGGTTGGCAGAATGATGAAAAATAGC

TGGGAACAGGAAACGTGCTATCAGCTGACTATCCTATATTCTTAAAACTTCTACAGCAAAATGTACGCGGCAAAATTCAA

ACTAAAACCACGGCAGCAGTTTTCAAAATTGCAGGTAAAGACCTAGGAAGACCTATCGGAGTTCGAGCAAAATCAGTTAT

AGGGGTTGATGAGAACAAAGTACCGAAAAAGGGTACTTTAAAGGGTTTACTGTACTCAGAATATCATACGATACCGCCAA

CTATGAGTAATATGCTTAAGGCAACGTGGAAACCGGAATACCTGAAACTAATTGAAAAAGTAAAGACTCGTTAGAGTTGA

TTGAGTATCTCCGGAAAGAGGAGCGGAACAAGGAAAAAGCCCCTTAATTCAATTAGGAATAAGATTACGGAACACGATCA

GCTCCCAAGGTGCTGCTTGCTTGGTGTGTGGGACTACGGAAGATGTGGAAATGCATCATATTAAACCTCTTAAATACCTT

AAGGAGATCAACAAGTTGAAGAGGCATATCGCGGCAGTAAATATACCACAGATTCCGCTCTGTCGAAAACATCACCTAGA

GGCCCACAAGGGTAACTGAAGAAATAATCCTCACCCTATCACAGGGCAAAAAATTCTCCAGCAATGAAAACATGTAGACG

GTGGAGAGCCTCGTGATGGGTAACCATCTCGCGGGGTTCGGAGGGACTCGTGGCTGATGGTCTAGACCGTATAATACCCG

GTTACTTTTAGCTTTCGTTTCACCCTATCAATATTTCTAAGTATTGAATTACAAAGTTATGGCTTGTATTTACTTAGTAC

AATATATAGAAATTCGGAATTAGCCACAAATGGTGGTCTTACTTATTTTTATTAGGTGGAGCAGAGTGACGTAGGAAGTT

ACTTCTATACTGCCTACAACTATCAAACTCCGGGGACGCCTTAAAGCTTAAGGTACCAAACTGTATCTGAAAAGGTATAT

GTGGATTAGCTAATAACTTATGTAAGGTAATAATTCTTAAGATGGGGAAACTCCAAGAGGTTATCGCGGATCTAAACCAA

TAATACCTAAATAAGGTATTATTGCAAAAGAGCAAAGAGTAGATGGTAGTTTATCTGATTCATCGTGATCAGGTATAAGG

TGTACTCTAAAGTATTTCGAAAGAAATACTCAAGTCAGAATCCTTTCTAACCATTTAAATCTAATTAACAATCGTTTATA

TTCAACCAAAGCAACTCACTAATATTTATCCTTTAGATTTAACCAGTACAGAACTAGATGTTGTAGCGCCGATTAATCCT

TGATTAGTAACCGGATTTCTTGATGGTGAAGGTAGTTTTATTATAAGTATTGTAAAGAATAATAATAAGGTAGGATGACA

AGTAAAATTCTAATTTTTACTTTCATTACATGATGGAGATAAAGTTCTTTTAGAAAGTATTCCAAAATATTTTAAAGTTG

GTTACATATTTAAAGATAAAACCCAAAAGATTCTCCACTACCGTATTACTTCAACTCAAGATTTAATAAAAATTTGGGGA

TCATTTGGATCAATTTCCTTTAATTACTCCAAAATACGCAGACTATCTCCTCTTTAAACAAGGGTATGATCTGCTAGCTA

ATAAACAACATTTAACTATTTCTGGTTTACATCAAATAGTAGGATTTAAATCTTCAATGAATTTAGGTTTATCTGATCAT

TTGAAGGAATCTTTTCCAAATATAGTTCCTGCAATTAGACCTTTAGTTGTAAATCAACTTATAGCTAATCCTCAATGATT

AGCAGGATTTGCATCAGCAGAAGGTTGTTTTTATTGGTATAAACAAGTCTTCAACTACTAAAACCAAGGTTAATATACAA

CTTTAATTTCAACTTACTCAGCATATTAGAGATGAATTTTTAATTAAAAGTCTAGTTGAATATTGAAACTGTGGAAGTGC

TCATCAAAGTAATAATGTGTTTAGGTATCGAGTAAGTAAATTCAGGGATCTTTCTGAAAAAATAATACCGTTCTTTGAAG

AGCACCCTATTGTTGGTATAAAGTCTAAAGATTTTAAGGATTTTTGTACTGTGGTTGATATGATGAAAGAAAAAACATTT

ATCTTTTGAGGGAATTGAACAGATAAGAATAATAAAATCTGGTACAAATACTGGAAGAGATAAAAGTATTTAAAGTTTAA

CTTATTTAAACTTATTTGTATGATAAATCTGACTTCAAGCCACACTCAATTATGGGTTTAAGCTCCTGTTTTATTGTGCG

CCTTTGAGTTAATTGTCGAAATGGATCCTCGGGATCTAGACATTATCCGAATATGTCTCCAGCAACGGGATTAGGTTCAG

GGAAAGCCCAGGGCTAAACATAGCATTCCCGGTTAAAGGTGGGACAGGTCGAAACCGAATTAGACTAGTAGCCCATCGAG

CTGAATCTTCTATGGACAAAGCTATACTGCCTAAATCTTATCTACCAGGGGCTCGTCAGGAGGCCGGCATCTCGTTCGGG

ACAGGATGCGTAAAGTGAATTGGGTTACGAAATGAATAACCAGATTTCGGATCGTCTTTAACCAGGGCAGAGTATCTGCT

CGTAACGTAGAGAATAAGAGTCCTAAGGAAGAACGCAATTTGATGATAGATACTAAGGGACTGCCTAAGGTTCCAAAGAA

CTATGGCAGCAGAGGAATCGTAGTACCAGGAACCCTAACACTCCAGACAGTGAGTTTCAAGATAATGGGGGTATGGGGAA

GGATTCCAGGAATTAACGTCCGTTTCGTATCCACATCCGCTGAAGGTTTCAGTACAGTAACAACTGACGGAGTGGGGAAG

TTACGGAAGATTGCCAAATTGTGTAAAGATAATCCCAAATTTATTGTTACAGATAAATTGTATAGATTATTATATGACAA

GGATCTGTTCTGGATCGCTTACAACAAACTTAAAAGTAACCCAGGTAATATGACTCCAGGAATTATGCCAACTACTCTTG

ACGGAATGTCTAATGAGGTGATTGATGATTTAATTTCCAAACTTAGGGAAGGAACTTTCAACTTTCAACCAGGGAGAAGG

GTAAATATTCCGAAAGCCAATGGAAAGACACGTCCGCTAACCATAGCGCCTCCTAGGGACAAGCTGGTTCAAGAATGTAT

AAGAATGATCTTGGAAGCGATTTATGAACCTAGTTTCTCTGATTGTAGTCACGGATTTCGTAACGGTAAAAGTTGCCATA

CTGCGTTGAAAATGATCAACCAAAAATTCGGTATGGCGACCTGATTCATAGAAGGAGATATTTCCAATTGCTTCCCTTCT

ATTGATCATCGTTTTCTCCTGAATATACTGAGAATTAGAATTAAAGATGAAAGATTCATTGAAATAATTCGTAAATCATT

AAAAGCTGGTTACTTTGAATTTAATAAATATTCGCATAGTGTCGTGGGTACACCGCAAGGTTCTATTATCAGCCCAATAC

TAGCAAATATCTTCTTAGACAAGCTAGACAAATTTATCTTAAATCTAAAAGCCGATTTCGACATAGGTACTAAAGCAACG

ATCAATCCAGAGTACAAGAGATTAGAGAAAATAAATATTCAGCAAAGACAGTTGGTGAGAAAATGGAAATACACAAAGAA

TGAATAAAGGTACCTTCAAAGTTGGCTATAGATCCCAAATTTCAGAAGTTAGAATATGTGAGATACGCTGATGATTGAAT

TATAGGAGTTAGAGGATCAAAACTAGATTGTAGGTTGTTGTTACAGAAGATAAGTGACTTCCTGGAATCAGAGATGAATT

TGGAACTATCAAAGAGAAAACTTTGATAACCAATAGTAAAGAAGAAGCCGCGATGTTCTTATCCACAAGGGTCAAGAAAG

GTGCTCACACTACGTATCGTCGATCTCAGGGAAGATTGAGAAGAAATGTGAGAAACATAAGATTAACAGCACCAATAGAT

AGAGTCACCAAAAACTGAAAGCGGGGGCTTCATTAAGGGTAATGTATCCACACCCAAATTTATATGGATGCATAACGATA

AGGAGGCGATAATCGTGCTGTACAATTCCGTCTATCGAGGTATTATGAATTATTATCGTTTTGCCTGCAATTTCAATGAC

CTATCAGCCAAAGTGCATTATATCCTGAAAGAATCATGTGCCAAACTTCTGGCCGCCAAATTTAATTTGAATAGTCAAGC

AAAGGTTTTGCTCGTTTTGGTAAGGATCTAAGGGGTGACGGCAAACATAAATTTGTTGAGGTAATTTTAGGGATTAACAC

AGCAGCATTCATGGTGAGCGTAGATGACATCAATCTTAAGATCTTCGCTGAAAATATTTCGAAAGCATCTTTAGATAATC

TGCGATGTACAATCTGTGATTCAGATTATAGAGTGGAGATGCACCATGTTAGAATGATGAAAGACCTAAACCCCCAAATT

AGTCTCGTGGATAGACTAATGGCTAAAAGAAGACGGAAACAGATTCCACTTTGCAGAAATTGTCACATGAAACATCACAA

TAAAGTGGGAAAGAATACACAACAATCAAAAGTTAGTTCTGGAGAGCCGTATGATGGGAAACTATCACGTACGGTTCGGG

AAAGGGTAGTGTAATTTCGCTATAAGCATAGTAAAGGTTGGGAAATCGTGAATGTTTCGGGAACCCGGGGCCTGCCCGGA

TAGTTCTCGGATTAAAAGCGTAAAAGACCCTGCTGATCTATAAGTTCAGAGACCGATACATCTTATCTCTGAGTGTTACA

TTACTCTAGTTCATTTATTTGGAATAAGTTTAATATATGCAAATTCAGGTACAACAAATTTAGACGGGTTATATATAATA

ACTAGTTTGAGTGATTTGAGTAGTAGTGGAAATGGTTTAACTAACTACCCTGATGTTTCATACTGGTATAAATCAGATTA

TATAAACTATTCTTTATTAATATTAAGTGTAGGTTTTTATTTAAAATTAGTGCCGCACCTTTTCATTTTGATCTCCTCTT

GTTTATGATGCAATACCTACAATTGTTACGACTTTTGTAGCTATTATTGCTAAAATATCTATACTAATTTTTTATTAGAG

TTAGTACATTATACAAGTAATTCCTTATATTTTTCTTTCAAATATACTTGAACTACTAGCCTATTAGTTAGTTCATTACT

TTCATTAATAATTGGTTCAATACTAGGTTTAAGAGAAAAGAGAATAAAAAGATTGTTAGCTTTTAGTACTATTTCACATT

TAGGTTTCTTATTATTAGCTTTAAGTATAAATAGTAACTCCCCTTTCTATCTATGCTTTTATATTTTACTTATTGCAATA

TTCTATAAGTAACCTAAACGTTTTATAATACTTATTAGCATTGGTTATTCTTTTATAGTTATGTTAACAATAGTGATAAC

GAGGCTAAACAATTAAAAGATAAAAATAATTCGCCTATACAGCTAATTAGCCAATTGAAGGGTTATTTTAATATAAACCC

ATTATTATCCTTAAGTTTAGCTATTACAATATTTTCTTTTGTTGGTATACCCCTTATGGGTTTTTACGACGTAACAGTTC

TCTTGTGGTCATAACCATGAACATCTTAGGCGACAACCAGAAAGGATTATTTGAGCTGGCTATAGCCAACGAGCTTCGGT

CAGAAGGCTCAATGAATAGCGGAAAGTATGAAAGTGAAGGTTTAAATACTAGTAGGCGTTCCTTAGACTGCGACATTATT

AGGGAACAACGAGAGTGAATTACTGTGTGAAAGCTGGTCCAACCATTCCTGGTCAACCTACACTTAACCAGGGGAATAAT

AGTATGAATTCCAATATAAAAGTTGAGAACGTCGCCCATACTCCCAATACCAGTACAGGTAAGCTCCATAATAATGCTTA

AAGAGGCATTCATAACAATCAGAAATATGAAAATCTTTGACAGTCACAAGAGAATCAAATGCGGTAACCCCGCTATGAGA

TATCAATCTTTGGACACTATAAGAAATTACAGTGAGGATTGAAAATGTGCAAATGCACTAGTGACGAGCAAAGGCGGAAT

GTCAGTTCGTCATTACTCATGTGGGGCAAGATAGCAGAGAAAGCAAATGGCTCACGTAATACTGAGCATAACGAGATAAC

TCGTGATATTAATGCAAAAATATCGCCCACTTCTGCAAAGCTCTTAGGGACTCAAACAAACAAACTGAGTACTGTGAAGT

GGAAGAATCAGGATCCCAGTAGGGGAGTTCAGAAGCTGCAGAACCCAAATGACCAAAGGGAAGACAACTCTCAGAGATTT

TGGAGAAAGTACATATAGAACAGATGGAAATTGTTGAGCAAGCTGAGAGATTTGGACTACATAGCAAACAAGTACATAGA

ATACAGGAGATAAAAGCAAAGTCTTATAAGTTTAGAATAGCTGCAGTACATCACATATTGACACAATCAGGGAGTGACAC

AGCAGGGGTAGATGGAATCACAATAAACACCAAATCGGATGATGTAGACAAGTTCGAACTGGTCGAATGATTAAAAAGTA

AAATAGATAATCCAAAACGTATAAAGCAAAACCAGTACGGAGGGTGTACATTCCCAAATCCAATGGAAAGCTAAGACCGT

TAGGTATCCCGACCATAAAGGATAGAACCCTGCAATCTTTAATAAATTCTATATTATTACCCCTAGTTGAGCTAACGTCT

GATAAACAAAGCTACGGATACAGACCCTACAGATCGGCAAAGAATGCTTTAGGAACAATTAGACAGTGCTTAATGACGGG

ATCGGAATATAAATGAATTCTAGATGCAGATATTAAAGGTTTTTGATAATATAAATCATGAATGGATAATGAACAACGTA

ATGATACCTCCAAAATTAAAAATAATTTTAGAAAACTGACTAAAATCCGGTACAATATATGAAAATAAATTTAGAGAAAC

CAATACGGGAACACCACAAGGAGGTATCATTAGCCCTACGTTAGCTAATATGACTCTTAATGGATTAGAAAAGGTAGTTC

TGGAGGCAATCTGACCTCTAACGAGAAGCACAGAACAAAGGATGGTAAGTAAACCCACAAAAGTCCAGACCTCAGAAGGA

GAAATGTCAATACCCAACAAGAGAAGAGCCCAGGGAGTACAAGTAGTAAGATATGCGGACGACTTTGTAATAATAAGCAG

AAGCCAACACATAATATCACGGTACATAAAGCCCAGAGTGACTGACTTCTTACTAGAAAGGGATTGACACTATCATCAGA

GAAAACCAGAATGTTTACCCTCCAGGATGAGAAGTCTAAATTAAATTTCTTAGGTTATACATTTCAATACAAAACAAATG

AAAAGCCAATAGATCATTAGTTAAGGACCACATAGATCAATCGGCAGTAGCTTTATTGCCGCAAAAGAGAAGGTAATAGC

ATTTAACAGATCGTTGAGAGAAGTATTCAGCCGAAACACCAATAAGACTGCATATGAACTCATCGCTTTATTGAATCCCA

AAATCCGAGGGTTTGCCAATTACTATAACATTGGTAACTCATCCAGATATCTAGACTATGTAAGACAAGCACTGTATCAC

CTCAGTTGAAAATGAGCTCATAAGAAACATCCCAAATGAGGAAGGAAATCCATTGCTAGAATGTACTTCCTGAAGCTAGA

TAAAGAAAGAATGGGTAAAAGAAAGTATGAAACAAGTTCATCAACATTAATACACAACGTGAGATGAGCGTTCAGAGGAA

TCACGAGAAAAACTTCACATTACAAAGAAGACAAGAAGAGTGCGGGATATTATAACTATTTACTATCACCGTCTGTAACT

AGTAACATTTTATCCGCACTAAAATACAGAATGCCCGATACACTACAAAGTGTACATGCATACCACAAAGATTATATGGA

ACTTGTGGAATTTAGTAGTAAGACCAACTTTTAGCTATGGGAAAGACATCTTCGTTTAAAGAAAAACTTATGAAGAATCA

GGAGGGTAAGTGTATAATATGTGAAGACGCTATAACCGTAGAACAAATCGCGAATGGGGCAATTCATATACATCATATTG

TACCTGTGTACAAGAAAGGAGCCAAATCCGATGTAAAAACATGCAACTTTTACATTCATGGTGTCATAAAGAAGTGAAAC

ATTAGCACAATCAGCCAGGGCAGATGGGAAATATTCAGGTAGAACGTTGAATGGCTCCAAGCTGAACTGCCTACAGGGTT

GAGCCGTATGCTGAGCAATTGGCACGTACGGTTCTTTGAGGGGATCTTATTGTGAAATGGGTTCCATCTCTAGTGCTAAA

CAGATGGTATTAAGTGCTGCATTAGATAGTGGTTATGTTTTTAGTTTTAATTGCCATATTAACTAGTGTAATAAGTGCAG

TATATTATTTAAATATAATAAAAGAAGTTTTTTATAAAACTGATTACAAAATTAATTCTCGGAGCCATCATCTTGCATTT

AACTCTAAACCATTAGAAGCAGCCGCAGCAGCTGCAGCAGCAGCCGCAGCAGCAGCAGCAGCAGCCGCAGCAGCAGCGCT

AAATAGTGATTTTACTTGCCAGTGAGATGCCAGTCAAGTAGTATGCAATGCAAGCAAGGGGAATTTAGTAAAAATGAGAG

TTATCTATACAATAACTATATCTATATTAACATTATTAATACTATTGTTTATATGCTTCTCCATTAACTTGTTAGATTTG

GTAAATATATTATCTTTCCTCTAAGTTGGGTGTTTATAAATTTTGCCATCCGGCGATTAGAGGAGAGGAATTTTTTGCTT

AGCCCAGGGCAGCTGCAGGGCTAAAAGGGTGCCCGGCGGCGGATCCTTATAAAGGCAAATCTAATTTTCTTAGTGCGCAC

TAAGCGCACTAAGCATACTTTACCTTACTGATGCAAAAAATAATGGGGAGGAATCTTTTATTGGGTTTTACAAAGGGCGG

TTTTATATTAAGCTGGGGGTGGGAGGGTCTTTAGGCGGCTTTTTACAAATCTAAACCTTTTGGAAGATGTTTTATAATTT

TCTTCCCCTTTAGGGGATGCTAAGGGGAGAATAGCAGAAGATGGATCCCTCAAACCGAAACTTCTTTATTTTTGCAGCCA

AAATTCCAAAATAAAAATCGATAGATTTTTATTTGGATATTTTGCAAAATTAAAAAATAAAAATCGAGAGCTTTTTATTT

TGATATTTTTTTCGTCCCTCAAAACTTTTGAGGGATGGACAAACCACCAGCGAGTTTGCCATCGAAGAATGGGGAAGCGC

AAAATAATTAACCCCTCTCTTGAACCCCTAAATTTTAATATTTCCCCTCGCTACGCAGGGGAGAATTAATCCTTCTCCCA

TCGGGGATTAGGATCTCTACAAGCTCCCTCCCTGCGTCTCGAGGGAAAGAATAATTATTATATTACTTTAAAATCCCGCT

TAGCAGCAGGAGGGGATAAAGTTTTATTATTCTTTCCCTCGAGATGATATTTTTGTGGCTAACCCATCGCATCGGGGAAG

GATCCGGCGATTGCTGGGCTATCTGGGGGTTTAATAATTAAGTGCCTCAGATAGGCAAGTCTTGCAATAGTAGATTTGGC

TAGTTTTAAAGGGGTAGGATGTCAAAACTTTGAGGGACGGCACCGCGGCAAACCGCCGCAGGGCGTCCCAAAACCGGCGA

TGATCGGTGGCCAGCTATACCCAGCAGGGATCGGCGGGATTAGGAGGCGCTTCTAGTTTTGCGGGGACAAACACAGGTTT

TTCTTGTCCTGATTTCTTATTTCGGATTTTATTTCTTATTTCTACACCCTCCTGCCTCGAAGAGGCAGGAGGAATTAAAA

ATCTAAAAATCCCAAATCCCATTTGGGATTTTTTAATTTTGCAAAAATATATTTTGCAAAAATATTCTTCCAAAACCCCA

ATCAAAGGGGCCATGCTTTTTATTCTTTTTTATCCCCGGGCCCTCCACCCCCGATCGATCGGGGGTGAGGGAATCCCTCC

TGTCCTGTCAAATAGGGGTTGAGTTCCCGTTGAGGAACGAAAACCTAGTCCCATCGGGGGACGAAAATCATGTTCCCACC

TCGGGACCAGTTCCTGGGGCCCTCAGGAACTAGTTTTTCAAAACTTCTGGTTTTGAAAACAAACCAGTTGGTGTTTCCTG

CAAACTAGTCCCTCCCTTCCCAATCGCTAGATCGATGATTTTGCGATTTTTCGATTTATCGGGGATTGGCAAGGGAAGGG

ATAAGTCCCTCCGGGAGGGACTAGTTTTTCAAAACTTCTGGTTTTGAAAACAAACAAGTTTGTTTCCTGCAAACTAGTCC

CGGGGACGAAAATCATGTTCCCACCTCGGGACCAGTTCCTGGGGGCCGGAACGAGTTTTTTAAAACTTCTGGTTTTGAAA

ACAAACAAGTTTGTTTCCTGCAAACTAGTCCGGATCCCCGAAGATTGAGGATTGATCGATTGGGGATTGGGATAAGTCCT

CTCGAGGGACTAGTCGGGGACTTCCAATTTCCCTCGGGACCAGTTCCTGGCCCCTCAGGAACTAGTGTGTTTTTGCGGGG

AAAACGGGGGTTTTGCCCCTCTGGCGTCCTCTCCGAAGGAGAGGATGCAAACAACCTTTGGGGTTTGAACAAGTGGATTT

TCGGCTTTTTAACTTTTGAGGGAGCACCTCTAGATGAAAACCACTAGTTCCTAATCCGGCCAGCAGGGGGATAAGCGAGG

GGAGGGAGGGGTCCCGGGGAAATTTCTCGAGGGAACGGAGTTAGTTTTATCTTCTTGAAAACTCATGTAGAAGCCTTATC

TCTAAAGCCCGCAGGGGTAGTATAAGGTTCTTTTGCCTAGCAACGGCTGCTAATGGCTAGGGCAGGTAGGGGAAGGGAAG

GATTAGAAAGACCCACCCCTTTAAAAGGAGGGATAGGGCTAATCCCCGCTACGGCTGGCCAGGTTCATGGATCCCGTTAA

GGGGGATAAGGGGATAGGAGAGGGAATTGTCCCGGATTTTTCTAATCCTTATCCCCTAGGCAGCCTAGGGGCTGGCGGAT

GCTAAGGCCGCCAGGGGATCGATTGGGAGCTTACCGGGGATAAGCCGCCGCCGATCGATGATGGATGATGATTGGTGATC

ATCGGGGATTAGAGGGATTAGGACGCCTCTGGGGATAAGCCGCAAGCACCCCGATCCCTCCTGGCGCAAGTTGTTCGGAA

GGAGTCTTTTTCTAAACCCTCCTATTTTTGTGGTACAAAAATGGTTTTGTACCTAAAAATAAGTTTTAGGCACGAAAAGT

TTTAGGACAAAACTTTTGTCCCTCTGCATCCATCTCTCCTTCGGAGAGGGGATGATGCAAACCCTTTCTTTGATCGGTCT

CGGGGTTTGAACGGGGGTTTCGTGAGGAACGAAAACTAGGTCCCTCTTATCCCTCTAGCTACGCCTAAAGAGGCTAGGGA

GGAGGCTAGGGGATAAGGGGGCTAGGGATAGGTAAAGCCTACCTACAGGGGAGTTTCTCCTCCTGTCTGTTTAAATGGGG

AGCTCAGCAGGAATATTTTTCTTTAATCCTATCCCGCCTTAGGGCAGGGATTATTTTAAAGATATATATTTAATTTTTCC

CTTCCTGGGGGTATAAATATATATATATATTATATTCCTACACCTACCGCTCCAATCCAAGTCCTGCTCCTCCGGGGAAA

ATAAAGTTTTTAGCCAGATATTGGGTTCCTTAGCTTAATTGGTAAAGCGTATTCTTGATAAGGATGTGTTTAGAGTTCAA

GTCTCTGAGGAATCAAATTTCACGCCAGGTATTTGGGGGCTCCTCTTTATGGCTTTTATATTTTCTATAACGAGCTACTC

GTTACTCTTACTCCGTTACTCTTACTCCGTTACTCCGTTACTCTTACTCCGTGACTCTTACTCCGTTACTCCGTTACTCC

GTTAGTCTTACTCCGTTACTCCGTGACTCTTACTCCGTTACTCCGTTACTCCTGACTTCGGGGGCAGGCTGCAGGGGTTA

CACTTCGAACTTCTTCGTTCTTCGAAGTGTAACGATATCTAAAAGCGAGCCGAAATTAATCGGGGGTTTCTCTGCGGGGG

TAGCCGCAGAGGCGGGCAGCTCAGGGGAAAAATGCTAAGGGGCCTGCCATAAGCTAACCCTGCTTAGGGGATGGAAGCTT

GCAGGGGAGGCTTGAGCCTAGTGGTTTTCGTGGCTCTCCTCAAAAAAATCTCAAAATCCCAAATCTCTCGATTTTTATTT

TTAATTTTTGCAAAAGATCGATCTTTTGCAAAATATTCTTCCAAAACCACCCCAATCAAAAGCCGCCATTCATCATCGGG

GGTTTTTGGATTCTTTTTATCCCCTGGTCCCTCCACCCGCCCCGATCAGGGGAGAATTGGGAATCCCTCCTGTCAAATAA

TCGGGGGTTAGGTGGTGAGGGACAAAAGAGTGAGTTTTGTCCCACAAAACTAGTTCCTCTCGAGGGATCTCATCTCCGAT

TATCCCCAATCAATCGACGTATGGAGAGCCGGGGAATAAGTTCCTCCAGCGATCATCGGGGACTAGTGGTTTTCGTTCCG

CCTCAAAACTTGTTGTTTTGATGAGGGACCAAAACGGGTGTCGTTGTCCCACAAAGGTTTTCGTTCCTCAAACTGGTTTG

AGGGACCAAAACCCCAATCATCCGGGGTTCGTGGCCCACAAGTTGGGGAAATTCTTGCGCCTAAAAAGATAGGAGAGAGT

TGGCTGAGTGGTTTAAAGCGACTAGCTTGAAACTAGTTAAAATTAATAATTTTCATGGGTTCGAATCCCTTACTCTCTGT

ATATTTTTGTCCTTCGGGGACCAGGGATATTATACAAGGGGATATCTAATTATTGTTGCTTTATGGAAAGCTATGCTATA

CCTTTGATAAAAATAGTACCTCTAGGGACCGCAGGCCGTCCTCCGCCTCGAGCGGGAACCCGAAGGGCTTCCTAATCCAG

GGGTATGGTATCCCGCCTTAGATCGATCGGCGATTGATTGATGTGATTGATTCATCGGGGATTGGACGCCCAGGGGATAG

GGCTTTATTATTCTCCCTCCCTAAAGGGGATTACAGGGGAGGGAAATTAGAGGATAACTATTTATCTGCTTTTTTCTTAT

TTAATTATTTGTCGGCAGGGGTTCCGAAAAAGTCTGGGCCTTGGGGGTCGGAATAATAAATTCTCCTACCGGGGATAGGG

AAGAGGAGTAAAATACCCCAACGGATTAGAGCCAGGTTGGTTAGGCGCCTCATTTGGGTTGAGGAATTGTTATGTTCGAA

TCATAATAATCCGAATTAAAACCCTTCCTCTCAACAACCGGGAACAACCGGGAACAACCCCTTTTTTAGTTTTCGTTCCT

AAAAAATATATATAAAACCCAAACCCAAAAGAAAAAGTCTTTTTTATTTGGGTTTAATTTTGCTGCAGCTTGCTTGCTTG

CTTGCTTTGCAGCAAAATATATCTTTGCTGCAAAGCCCCGGTATTATAGTGAAGTTTAAATTATAAGACATCGTGTACCC

TTTTTATTAGGATGTTTAGGCTTAAATATCCATTATATTTAAATTTAAATTTACTATTTTAATAGATATTAGTAGTATAC

ATCGTTAGGCGCGATGTTGCAATTAGATTAAGTTAAGGTGGATGTTAGTCTAATTCTCATCCTATCCACCTTATCCAGAG

TATCTCCCATCATATCTTACCTATAGAGGACGAGACCTAGTTAATATACTATAGTTGTCGATTTTGTCCAAGCCTAGGTC

TTTTGCCTTCTCCTTTACTTATTAGCAGCACGATTTACTTACTCGCTCTTATGTTTCATCGTTTTAGCGTCATTATAAGG

GATTGGTAGTCAAGGTTCAGCGTAATAACGCCAGGCTCCGTTTTAGTTTGCGACTCATTCTATTCCTTCTATCCATATTT

CGACAATCGAAATGCGCTTAAAGCTTTATTTAAGTAGCTTTACCCTATAGGATTACTTTGTGAGTCGCATGGTTTTCTAT

CTCTAGCGATAACTAATCAAATCCTAATATAAGTGATTGTATCTCGTCGTCACATTAGGATCCTCCTTATATAGACGACT

ATTGTATATGTTTATTAATAATTTAATGATGAATAACCCTAACCCTAACCCTAACCCTAACCCTAACCCTAACCTAACCT

AACCCTTACCATAAAAATTTGTTTTCACGGTTTTGGATAGGTGTTAAAAAGGTCTTTTCACTCCAACTCTCCCTAATCAT

ATTCTCCTGCTTAATAAGAATCCTATCATTAGAATTTTTAGAGTACTTGCAGGAATAAGTATTTTACTTATTTAACTCAT

AGATTAGATTCTTTAGGTGAGGGATTATTATACCTTTCTAGTTTAGTTCTTTGTACGGGTTTAGCTTTGCTCTTTAGTAT

ATATCATATGGTTTAACTTATCATAGAATTAAGCATATGATTAAAGTTTTGAAGAGTGATGAGTTAGATGTTAGAAACTC

ACCTTTGGATAGATTAGCTAGTATAGCTGCTAGAAGAATTTCTGTAGTAAGGTTGTGTGAAAGCAGCAGCAGCAGCAGCA

GCGAGCAGCAGCAGCGAGCGAGCGAGCAGCGAGCGAGCGAGCGAGCAGCGAGCGCAGCGCGGCAGCTCCGTTGGTGTGGT

TTTGTGGAATGGCGGGTATTGATGAACTTAGAAAAGCCCACGGTTTAGAACCTATCTTTCTACCTCATTTAGCGCCCAGA

ATATTTTCGGATAGTGAGTCGACTAAAGAAATGAAACTAATGCGTTATAACGAAGCCACTTTAGAACGTAATAGTCAATA

ACTTAATGCGTATAAACACGAGCATGCTATTGTGGGGATACATTTGAACCAAAACCCTGATTAGTCAGCAGGTGGATAAT

ACCTGAAGAGATAAATAAAGCGTAATGAATCCTTATGCAAAAGCAATAGTAAAGAGATTAAATCTAAAATCTTAAGTTCT

TTAGATAAATTAAATGAAATACGGAATAATAGAAAATAAGCGTTGTTCTAATCCTACTTCGTACAAGTGGGCCTCTCGTT

TTGGGGTTTTGGAATAAATATATATAATTACCTGACCCTTAGGTTCCTAGTTAATTATTAGTAGGAAGGAAGGATATATA

TTTATATAGGAGTTATCTAGTCTTCTAACCAGCTTTTCCTTTCACCCTCCTTGAAGAGAAGAGAAAGTGGCAAATATTAA

TTGGAGTTAGGTATGGGGATAATTCTTTAGCACATATTATTAGCGTATAATTCTTTTCGGGGTAAGGCGCAATTGAAAAA

CGAAATAAAGTTTCGTTAATAGAGGAGACAGCCGCCTCGGACTTCTACGACGATAGAAGGACAAATTGGCTCAATAGCGG

GAGCACCGTGTTAATTCCAGCTAATTCGACCAGCAGGTACGAAATGGAATGGAATAAAAGAAGAGATTAAACCATAGGAC

AACCATTTTGTACTTTTTATTTCCCACAGAGATGAAGAAAAGAGAGAATTAGAGTAATTAACCAAAAAAGATCAGTATTT

GGGGGACTCGATATACAGCGGCTGTATCGAGTCACCCACAGGATTATTCAGGAACCACAATAGGCCAATATTTTACGGGG

TTTAAATTTTGCTAAAGTGGACCACCCTATGGTTATAAGACCTATTTAACGATAGAGGAAGGAACCAACACCGGTCCCAG

CGAAGCAGTTGCTAAAACGTCAGGGGTTTTTCAGCAACCACCCCGAGAATTATAGTAATGAACCCAAACAAAACAAATAT

CATAAAAGGATTTTTGGGGGACTCGATATACAGCGCTGCAGTATCGAGTCCACCAACCACAATAGGCCAATATTTTACGG

TTAAGGAAGTTTAATCTTAAATAAACTTCCCTCTTGTTAATAGAGGGAGAACCTCGACTTCTACGACCGCGCAGCTAGCT

AAAAGAAACGCAGCATTAGCGGAGCAGAGATTTTATTCCTGCAGATTTCGACGATCTGAAAAGGAATAAAATCGATTAAA

CAATAGGACAACCAATTGTTATAAAACCAACAAACTGTTTAGAATACAGCTAGATATCTAATACTTAAATTCCCAAACCA

ACTAGGATTATGGAAGGGAGGAGGAGGAGGAGGAGTAGGAGTAGGAGTAGGAGTAGGAGTAATATTATTAAAAGTCTTTA

AAACTTTATTATATATATTTATCAAGAGTATTTATAAATATGATATTTACACTTTTATCTTGTATTGTATTTTGTTATTT

TTTATTGATATGAAATTAAATTTGAATTCTATTTGGAAATATGTTGCAGGAGGGTACTGTTCTTGGTTATCAAGCCTTTT

ATGAAAGAATGGTTAATAAAAATCAAACAACTGAATTTAAGGATGCTGTTAACAGTATTAATAATAAACTAGATTTAATA

TACGAGGATGCTTGTAAAAATACTGCAGATAAGAGCGAGATTTTAGAAATGAAGAGGTCTTTTAAGGAATTTAAGGGTTC

TATGAAGGAATTATCAGATATACAGAATAATTATTGTAAAAGCATGCAGAAATTACGGATAATGAGAATTCTAAGAGTTT

ATTTGAGTCTTATAAAGAGGAATTTGGGAATGCTTTTAATAAAGCTAAGGAGGTTTGTGATAATATAGACAAAAATACAA

TAATTCAGATAATTCTATTAATAAATTAAGCAATGATGATAATTATATTATAAAGTTAATTAACGAATTTAAGGATTATT

TATCAAATTTATCTATTACTGAAATTTGTTTAGTTATTAACATAAGTAGTTGTTTGTTTATATTAACTTGTCTTGTTACT

ATATTATTAGCAGTATATGGTAATTTTATTATTAATAAATTCTCTTTAGAAGAAAAATACCCTAAAATAGCCAAATTAGT

TAAATTAAGAGTTAAACTACAACATACTTATATATTAGTTAACACATTGCTTATTTTGGTTGTTTTAATTTTAATGATTA

TAATTAATTTTATAACATTAACAAATGGTTAATACTATCCTGCTACTGAAAGTGTTAATTTAATATTAGTCCGGAAATTT

CGAATATTTATTATGTTAATATTAAGATTAGTAGTAACCATAATAAATATAATTTACTTATATTATTATGATCCTCTAAA

TATAGAGAAATAGGGTGAAGATATACTATCAATTGATTTGTAAATCATAAAAGAAAGGTTTTAAAGCCTAAGAATAAGTT

AATAAAGTGGATCTGTATTATCTCTATATTTAATTTTAATCTGTTTGTAGTTGTATATTATAGCAGAAATATTGTTTTAT

TTAGCCACCTTTTCTTCCCCACCCGGAAGAAAGGGAGGGAAACTTGGCTACGGGCAGGTCTAGCCAAGGCTTAGGCCGAA

GGGAGAGAGGTCACCCTCGCTAGCCTCTCCGGAGGGAGGGTAAGCCACCGTCCTGCAAGCCCTCCGTGGGAGGGTAACCC

TCGGACCCATCGGCTAAGCTAGCCTACTAGCGTCAGCCTCCCCGTAGGGGGTAAGCCTCCGCTAGCCTCACCCGAGGGTA

CCTGCAGGCTAGCCTCGGAGGTAAGCCTCCTCCTACCCTCTCCGATCTAGTTGCTTAGCCATACGGCTGGTCATGCAACT

TAGCCTGCGAGGATGGTCAAGCTACTTAGGCCTGCGGTTAGCCGCAGGCTTACTCTCCTCTAGTAAGCTCGCAAGCCTCT

CGCAGGGTGGTTTTAGGTTTTATCTAACTTTAAAGGGGTGTTTAATTTATCTTAATAACCATCCCTTAATGAAGAATTAA

AATATAAAAGGCGATATTATATTTACTTAAATAAGTTTTATTAAGGATTTATATCTAGTATTATGGAAACTCCTTATGTA

GGTAGTATAATAAATGGAGGAGGCTATTTCTTAGGTTAAAGCTATGAGGTTGCGTAGTCGAATTATTTATGTGTGTATGC

GCAACTTCCGATCTAAGGGGTATATTTAGGAGGCGGGTTACTTAAAGGTCTTTGTGCAGGTTGTGGATCATGATATAGTA

TTTATCTAGTGTACTATATCGAAAATACACTTATTGTGTATTAAACTAGTATTGTATTATATTAAATAAACATCAAATAA

TGAATAACTTTGACACGGATTTCCTGCACTTACTTGAAATGAATGAACGAATAAAGAATGAAAAAGAGGCAGTGGTTAAT

TTAGAAGACGAATTAAAGAGTCAATGTAGTAATTTTCTAAACAAGCATTCTATCCAGAATAAACTAGGTAGACTAGGCTC

TATTGAATCTTTATTAGATAGACGGTTATCTGCTAAACAATTACTAGAATTTATAGAAAGATCTGATAGAAATGAATGGG

GTCTTTTTCTAAATTACCACCTTATATTAAAGACCATATCAGAGGAGTTAAATTAGATAATGAAATACTTAATTATAATA

CAGACGTTATAACTCGATTAGAACATAAGTATAAAACATGGGATTCTTTAGATGATGAAAATTTAAATAATCTTTTGTGA

CTCCCGACTATGAGGACTATTTTATAGATAGGGTGACGGAATATTTATTAAAGGGGTTTTGAACTAGAATACAAGAGTAG

AGAAGTGAAAAACACGCCGAGGGTTATGTGAATCTCGTAGTATTTAATACTATTAAACTGAGAGATGAAAAGGTTTTAGT

TTATAATTATATAACATATCCCATGGGATTATTTAAATTATTGTATCTTTATGCAATATTAGAACAAAAATTCTGCCTAT

GTAGGGGAAAAAAGGGTATGTTGCCTCTGACTTTTACTTTGTTCTAGATAAGTCTTTAAGGATACCTGAGCTGGAACCTT

TAGACGCTAAGGTTAATAAGGCTTATCCAGGGTTTTTCTCCAGATAAGGACTATCTAGATATATTAGATAGTGAGGCATC

TAATTATATAACCCTATTTAAGTGGGTATCTTATGGACGCATTATGGATATAAATTTTGAAGAACTAGAGGACTTGGAAT

TAATAAAAGATCTTATTGGATTCGAATTCGCCAAGATATTAAGGAAGTTAAATAAGGATATACAGGTAGAACAAATTAGG

CAGTTTGTTAAAGAATTGAAAGGTGAAAATATATATAATATATTATCTAGATTTATATTTCTAGTAGAGGATTTAGATAA

GATGACTTATTACATTGAAAATAAACTAAAATTAAAGGTAAATCAAGGTCCTCAGAAATTTCGAGGACAATGTAGTTATA

TTTCACATATTTTATTTTCTATTGATAAAGCTTTTAGAAATAGTATGTATAATCATAATAGAATTTTTCTAAAGCCGGCG

GCGCCCTTTATGAACGTAAAAACACGATAGATATATTCCTAAAGGTTGTTTCTCTTTTAAAACATACATATAAATCTAGG

GAAGGTTAGATGGTAATCTCCTCCTTAGAAGAATCTTCTATTGCTTTAGCTATCCTGATAGGCGGCGGCGGCTTTGCAGG

GGATAGTAGGTAAAGAAGACCCCGTAAATTCCGCGACTTGGTTCTTCCCGTGGCATTTTTGGAAAAAGGGACTTCTGATT

AAGATATTTTACGAAGTAATTAATCTATAAATCCAAGCTCCGCAGGAGGTGGAGGGTATAGAATTTATCTAGTATTCTTA

TCCCCTCGGGATTCCCTAAGGGCGGTAATCCCTTAGGGGATTCCTAGCCCTCTGGGGGCTAGTACGAATTTTCCCTGTAC

CTTTTACAGTGCATGTATTCCCTTGATTTGTTAAAGGAATTTTCTCCTAAAGCGGTACCTTCTAGCGCACAAATCAAAGT

CCTTTCCGACGCATTAGGCGTTGTTTCAAGAAGGTGCTAATACTCGTAGCAGTTTTCTTCACCTTGAGGTATCGTAAGGA

TCGGTTATCTGTAAAATACGTTTTCTCATCTCGTAACCAGAAGGCGTCGTCTGACCTCGGGGTCAAATCGTACTAGCTAG

CAAGTCACCAGCCATCGTTACCGAAGAACTGGAAGAGAGATCCGCCAGAATCTCTAACCTCCGTGAGGGACTAGGTCCCG

AGCCTAGTTTGTGTCCCAGTTAGAGGGAATAACATGTCATAACATCTCTCTAATAATGTGTGGGTTTAAATGGGTAGTAG

TTATGGGGGACCCCACATAATTTAAGCTTTAAATATATTGGCAACTTCCGTTTTAAAATTAAATTTAAATTAAAGAATTA

AAATTGAGAAAATCCTCGGGAAAATGATTTATATTTTCCGGGAGGGAGAATCGCAATAAGTCTTTTTCTATTTAGAGGGA

TAAGTATAAAGAATCTTTAAATTAGGGTGATTCTTTAAATTAATTAAAATCTTTTAATTTTTGCACGACGAGGTTGATCT

CGTGGTTAATAATGGTCGAAATCAATCGGAGGATTTTCTGAGAGTCGAGTGCTTTGGAGCAATAACTAAACTTTTGGCTG

GCCTTTAGGTCCAACTTTTAACCAAATCTTAATTGTTTAAATTGGAAGGCAGGATCTCGTGCGTATTAAAATCGAAATTT

CGATTCTTGTGGCACCAAGGGTGTTTAGTAGTAGAGTTAATTATCCGTTAATTAATTCTCTATTAGTGTATGCTTCGTGT

GTTAGGGAGGATTTGGATGCTGGAGGTGGGGACCTAAACACGAGGCCCTAGGAGTATTAGAGGGTCATATTATATGAGGT

CATATTATATGAGTTCCTCTTATTTAAATAATACTCGGGTAAGCCCATGTTACATACACGATTTTATACCACCTTCACAT

AAAGACTATGTGAAGCTAATATATTTTATAATATAGGAATTTATAAACTTTGATATTATTACTTGATGGATTTGTCGGCT

AAGGTTCGTGACCTTCAATAGTCTGTAGATGTGCTAACACCTCATCCAGATATATTTCCCAGGGTCTATCAGGTCTATCT

CAAAGTTAGTCCTGGAAATTTACCATCCCATGAGATATGATTACTTGTTTTATTTTAAAAGATCGGTTCTTTAATTTCCT

GTAGTTGGGTTCACAAGATTCAACTATAATACTAATGGGTGTTGCATGCAAGGGGTAGTAGAGATGTAATACGAGGATCT

TGTATGGGCTAATCCATAATGCCGGAACACCTCATTAAATATTTGAAAGGTTGCAGGAGGAAAAAATCCCTACTAACAGA

CACAACAGGGAATTATCGAAAATAACTTATATAAGGACAATCTAGGAGGTTCAAGTTCCTCTAGGCTAAGTTAGGCTTTA

ATTTTTATTATACGGGCAACTAGAGTTACCTCATTAAATTGGACCTTTTCCAGTGCTACCTTATATTTAAAACTACCAAA

TAATAAAGTTACAAACACATTTTATACTACCTTTTATATCTTTAAGAAATTTATCACATTACTCTCGGGTGCGTCGCATA

AATGGGGTTGGAAATATTTCTCTAGGAAAAATCCCTCAAGATCATATAATTCGGACGAATGGGGGATAGTGCTACATATG

AGGCACCATCATACGATCTTCAGGACCGGAAAGTTGTGAATAAGAGTGACTCTTTTATACCCCTATATCTTCAGGATATC

AATATTAATACCTATAAACGATACACCAATTTACCCCGTTTGAACACGACAAAAAAATAAAGAATTATGTGTACGGTTTA

AGCAAGTGATATTACATTTACGATTTCGATATAGAGAGATGTTAACCCTAATGGTGAGGCAATACCTACATATAACAAAG

TATGTAACCATATTAACTCTATTTATGAATGGCCTCGAGGATAACACCATTTATAAATGTATGGTTGTAGGTGTATGTAG

TGAGGGTATTAAAACCAGTGCTACGAGCATATATCTCTCAAAAGAAACGAACCGTGAGTTTCTTGCAGACCAAATAGTAA

CCTATTGTACAGGAATACAGATGAAATACATGACAGACTTTACCCGTTGAAAACTTTGTGTAAGGAAATGATTATGTAAA

GCTGAGCAGGATATAAATTTTTCAGCGCTTCTTACTGCTGTTGAAAGCGGCCAACCTAAAGAGTGCGAATTTTATGGGAT

CTCAAAAGGCACTTATTCTTTCCAGGAAGCGAGGGGGTTTTACGTTAAAGATCGTCTCAATTGATTATCTGATTCAACTA

AAAGATGTGTAACCCTTACCCTTACCCTTACCCTTACCCTTACCTTACCCTTACCCTTACCCTTACCCTTACTTTAGGAG

ATATGACTACATAAGCATTGGAGATTACGGTATCCCTCAGGGGTAACGGTTTATATAAACACCTAAATCACGATTTAAAT

GTAAAACATATAGGGAGTGATTATCACGTATGCGTATACCAAGGCTCGAGATTGATAGCCAAATGAAGAGATAAAATTGC

GAGGGGAAGGGGTTACATCGTCGTTAATTCCCGGCTTTAGGTCTAAGGGTGTTTTATGAGGGTGCTCGTATCAACATGCT

GAAATGAGATACAAAACGCGAGATTGAGGGGTTAGGAGGCAGAGGTGAATATGATAACAAGATAGGGTCATTAGACCTTG

AGACCTATACTGAGCACGACGGGTTAGGCCTTGCTATACCTTAGCAGGTTTCCGTAAGATTACAGGTGAAGAGGAATTGT

TTTATCTAAAGGGTGAGGAAGACCCGATAGATATGCTTTGTCGTATGATTAACAGACTCCTACAGCAGGATAATGACGGA

TGATTGTTCTATGTGCACAATCTTTCAAGATTTTACAGTAGGTTTATCCTAGCTGCGTTAGGACGAATGGGGGTTAAAAC

CTAAAAACTTTTAGGAAGAGCTATTAACGAGATCTTTTCATCCAAATAAGCCCAATCAAAAAGGTAGGTAAAGAATATAT

TACAGTATCGTTGACTGACAGTATGTATATCTGTCAGTTCGAGTTTAGATAACTTAGCTAAAAGTTTGGTTCGGTAAACC

AGGGGTCATTTTCCTATACCTTTGTGCGAGCGGACAACCTGGAGTATAAGGGTTAAATCCCTTAAATAAACACTATAATA

AATTAGATAAAGAGGCATAGAGAAAATAGCATGCCAATATGGGGATAAAACCCCAGATGTATGAAAAGGAGACCTTGGCA

TATTTATCGCTAGATCTTATTAGTTTAATCAATGTAATGAGTACATTTAATAAATCTATCTTTGATAAATATAAGGTTAA

CACCACCAAGTTGTTCCTATTCGGCTTTATCCAAACTGGTTTATACAACAAAGTTCTACAAGGATAGCGGAGTAAAAATA

CCTGTGATATCGGGTTATATAGAGAAAATTATCCGGAAGGTATATTACGGGGTGGTTTAGTGGATGTTGATGAGCATATA

GTACAAAACGCATATAAATACGATTCTAACAGCCACTATCCAGCGGCTATGCTTAATGATATGCCTGTAGGAACCCTATT

ATATCCGATGAAAAGATATAAACAAGCTTTTGGGATTTTCCTACGCGAAAGTTAAAGCCTAGTGAGGGTAACTACGGCCA

CGGCTACGCTCCCTTACGAGATAGCAGCGGAGTAATAACATGTCCTCGAGGTCAATTTGAGGGTGTTTGGTTTACGGAGG

AATTAAAGGATAACATGTCAAGGGGTATGAAGTTGAGATAGTATCCAGCGTAGTTTTCAAACGAGGTAGAGGTCTTTTGA

CTCATTTATTAACACCCAGTTTACTTCGAAAGCCCAAGCCAAGGCCGAGGATTTGGTCGGTGAATTAATAGAGAAACTTC

TTATGAACTCTTTCTATGGTAAAAGTAAACTAGAGGTAGAAAACACCTATGCTATGATCGAAAGTGATCACCTTGAAGAG

TATGGTAAAAGCACGAGTATGACTTAAGCCAGGATTTCGATAAACTAACCCTAATCCGTGAGAAGGCAGGTTCAATGGAC

CCTACGATCCTTAAACTATTAAATCCAGAAAAGCAGTCGAAAAGGATAGCGTTAGAGGAGGCTCACCTAAGAGCTTAAGT

CTAACTCACGACTCCCAAGCCAACAGTCCAAGGGTGTCAAATCGTCTGTTGGTATAGCCGCGGCTATTACAGCCTATGCG

GAATTGGCCCTTAATAGGTTTCAAAATATTCCAGATAACAAATATTTAGGAGGTGATACGGACAGTGCCTATAATGCAAC

ACCCTTTACCCACCGAATATGTAGGTAAAGGTCTAGGGATGATTAAATTTGAGGACGATATTAAATTAGGATTTTTGCCG

ACAAGAAATTGTATTATGGGCCGTTAATAGCGAAGGTAAAGAGAACATTAAATCCCGAGGGGTAGGAAAGACTTTAATCG

TCAGGATATCTTAAAACTCCCTCACTTTTGACTAATGTTAGCAGGCGCCATGTGGTTAGCGTAAATAAGACCAAATTTGT

AATAACAAAAAGGGTGCGGTAGAAATTAAAAACGTGAGTCTAGACACCAAGATCCCAAAACTAACGTATGAAGATGTGCT

TAGTGAATTGTCAGGGTATCTAACAAACAGGAGACAAGCACCCTAAATATCATATCGCAAGATATATAAGTTCATTAAGA

AAGCTAAGTAAAAGTCAGGTTCTCGATTTTTATCGTGTGACCCTAAAGCGATACTAAATTCCTAGAGAATGCAGACAGTC

AAATAGAAGAGGGTATAAAGGAGTCAGAGGGTATGAGTAAAGAAGATAAAAAGAATCGTTATCCATAACCAAATATCTGG

CGAAAGAAACGAGACTAAGCTTAATGTGCGTCTTGCGTAAATCGCAGGCTCCCGAGCGTAAGCGCCAGAGTCAAAGCCTC

CTGACAATAAAGCCTTTTTTTCTCTCGTTGATTTTGAGGCAACCCTAATCAATAGTTGAAACTAAGCTTCGTTTCTCTCC

CTCATTAAGGAGTCCGCGGATATTGAAAAGAAGAAAGAAGAACGGCTCATACTTTCACCATCTCAGTTTTTCTATGAAGT

CGTAGGATAGAGGAATCAGAAAGAGGAGTTCAAAGTAAGTTAAAGACTAAGTTTAAATTAAAATATAACTCTCTATATAT

CTATATATACTTAGTTAGGTAGCCATCCGGAGTAATCAAGATATCTCTAGAAATAGTAACCGAATATCAGTCATATCGGA

TAGTGCACCAGCGGCGAATCTTTTCACGTAATTTTAGGTATTTCATCCCTTCACTAGGAGTGTATATACTGTTAAATTGC

GTTCGTAATCGTATGGATAGTTATTAGAGAATTAACGTCAACTTCCCAGTTTTTTCCACTCGCAATGCTCGGTAGGTCCA

ACCTGGAGCTTTAGAAGTCTCCTCGTAGTTCCGCCAGTTTTAGATATAAAGGTCATTAAAGTGATGCTTTATATCAGACT

CTGCAGCTCCGAGATATGCTATCTCGCGTAGTTTTAGTGCCCAGTTTTCCGTATATTTTTAAAAAGACTTTTTAATTTCT

TTAAAACATAAGGGAAAATCTGGGTAAGATTTTAACCGAATCTAAGCATAGGGGTAAAAAGGGTGCAAAAGTTTTTTCAG

ACAATAATCACGCAGCAGGGGAGTCTCACTCCAGAGTGTGTTTTGTGTGTTCAGGAATTTTCTAGGATATATTTAAATTG

TCTTGTAAAACCTAAGCTATTGGGGGACCCAAATCCGCTAGAAAATAGGTCAATAGGGTGCCCAAAAGTTTTTCAGACAA

TCAGAACGCTGCACGGAGTCTCAGTCCACACTTTTTCTGGCAAACTCGGGGAGCACCACCAATATATTTTTTCCCGTATC

CCGTATTTACCCTTGTAATCCCTTTAGGGGATGCAGCCTAAGGCCCAGGGGAGGGCCAGGTTCTGGTCTTTTGGGTAGCT

AGGGGATTAGCGGGGGATAAGCCTGCAGGGGAGGGAGAAGGAGCCGTAGCCCGGCGGGGGATACCGAACCAAAAGGGAAA

TTATATAGTAATCCCAACCTATTCTTAGGCAAGCTCACTTTATATTTCTCAATAGTATATAGTAGTATGTATGGATATTT

ACAATAAATTTTTATTACTATAAATGTAATGTATATAAAGAGACTATTAAGGAGGGCTAGCTATATATTAAAGAGATTAA

TTTGTGTTTTTTTACACATGCACTCTTAGAGAAATTCAATAATAAACGAAGTGAATTGAAATATCTTAGTAACTTTAGGA

AAAGAAATCAAACGAGATTCTATGATTAGTGTGAATGAAAATAGAAAAGCCGAAAAATTTATATTTAAATAAGGGGTCCC

CAGCTACGCAAGGGCGGGGAGAATAATAAAGATCCCTCTTATTGTATGGTCCCTCGGAGAGGGAATATGTATATGTAATC

TTGAGGGGATATTAAAAAGTAAAACGCTTGCTCCAGTTTGAATACGTCAAATAAGATATTCTTTTCCTGGTTTAAAAAAT

TCGGGAAACAATTATTTGACAAAGGGAACCTTCCTCTAAGGCTAAATATAATATATAAGCGATAGCGTATAGTACTGTGA

AGGAAAGGTTTGAATTAGTAGTCTTATAAGCAGCTTTAGCTTTATTTAATTAAAAGTGATAGCGTACCTTTTGCATAATG

GGTCAGTAAGTTAATATTAGATGCGAGCATACGTGCCCAGATAAACCAATTATCTTTTAATGAATTAGTATCTAAAATTA

GACCCGAAGGCTAGTGATCTTACCATGGTCAGGATTGTGCGACAAGAAGTTGTATATACTAATGTGGCGTTAATTCCACC

AGGACTGTATCTAGTCCTCGAGCCTGCTACGGGTGCTGAATAGAAGTAAATCTAGTTAGATATCTAATAAATATCCTTAG

ATAAGCGAATTGAAAGTGCTAAGCCGTATGTATAAGTGTCACTGTAAATAAATTTTTATTTCTTTGCAATGTGAGATTTA

GGCTGAAATTATCCCAACTATGGGGAGCGAAAGCAAAGTATGCGAAGAGACGATGAGTAACAAAGTTATATATTATCTTG

GAATGTTAAATTTTTAACTGAAATTGATAGTTAGTAGGGGTTAACTACGATCCTTAGTTAATTCTAGATACCAATCTACA

CCATCGTATAGCATGCCCCTAAGGTTGAAGATCAAAGTGGTATATATAGAACTTGGTAAGCCCCAAGTCCACTGATATTA

TGACGCTAATAATTTTACGTCGTAATTTATCCAGAGGCATTGTATATTAATAAAGCAATGTAACGGACTGGGGTAGAGGA

GACTTATAAAAGCGAATGCGCGAGAGTAATGATCGCGATAGAGTACAGATTAATATAGATTATAAATTATGATTGATTCA

TGCATAATTCTTTTGGTTTCTTTATGATATTCATGCTTTATTACCATACTAACTTAACTAAGTTTGTATAAATAAAAGAT

AAACTGTTTCAAGTAAATACCCGTATGAGCGTAGTAGCAAAATACAAGTAGAAAGGTATCGGGTCATTGGAAGGCTAGCA

CCCTGACCCAATGATAGAGTTGAAAGGACAGAGTCCAAAGTAATTGAATAATCAACGTTGGTAAAATAAATATAATAAAA

AAGCTAATAAAAACACAGTCTTTCTACAAAATAAAATAAACGAGCGTAAATATAGTACACAAGCAATATCTTTTTAGGAG

GAGTACACTCTTTAAGTGAGCACACCTATTTAACTAAACTGAGTCCTTGATTTATAACAGGGTTCTCTGATGCTGAAAGT

TGTTTTTATATAGGTATACAAAAAGCTCCAAAGTTAAAACAAACTGGGAAGTTCAACCTTGTTTTAAAATAGAGTTACAT

AAAAAGATATAGATTTATTAATACTAATTCAAGAATTCTTTAATGTAGGTAAAATAAAACATATGAATGATAAAGTTGTC

TTTTCTGTTAAATCTCTTAAAGATATTATGGTAGTTATTGATCATTTTGATAGTTACCCCTTATTTCTAAGAAACAAGCA

GATTTTAAATTATTTAAAGCCGTAATTCAATTAATGTGCTCGAAAGAACATTTAGTCGAAGAGGGGTTACATAAGATAAT

TAGCATCAGAGCTTCTTTAAACAATGGTTTATCTGATAAGTTAAACACAAGTTTCCCTAACACTGTCCCAGTAAATAAAC

CTATAGTCAAAGATACAGAAATCAAAGATGTTCAGTGGTTAATAGGATTTACTACTGGAGAAGGATGCTTCCTTATAGAT

ACATACAAAGCTAAAACTAAAGTAGGGGTTGGAGTTACTTTACGGTTTAAAATAGCTCAGCATTCACGGGATGCTGATTT

ACTTAAATCTTTTGTGACGTTTTTAGGTTGCGGTTCTTATGTCCCGAAATCAAAGTATGATATTGGAGAGTTTGTTGTTT

CTAAATTTGAAGATAATATTTTCAAGATAATTCCTTTATTTGAAAAATACCCTGTCTTAGGTATAAAATCCCAGGATTTT

CTAGATTTCAAAAAGGCAGCCGAGTTAATAAAGAATAAGGCACACCTAGAGGAATCAGGATTAGCGAAGATTCTATTAAT

AAAATCTGGTATGAATAGAGGTAGATAGATAATTTTACTTTTGGCACGTAGCGGGAACAGAAACACCAAATTACTTGGTT

TTTCATCTTTTCCTAATTTTTAGTCCTTCGGAGGAGCACTCTTTTTTTAGAAACAATTTCTATGTTATGTTATATAGTTG

GCCTTGCTACGCCTCCAAAGGATGGGGCTTCTATAAAAAAAGAGAAAAAAAATGTCCAAGCAAGGGGAACAATCAATTAT

AATTTATAATCTAAAGAATTAATCTAATTACTTAGGTCGAAAGGCCGCTGACTTAAAGTATGGTGACACCGTTTTACTTA

GGTTAAATATTCCTTTATACTCAGGACTAAACATTACCTGCTGTGATAAGGCCCTGTCGCAGCAGGCAAAGGTATATTAG

TCTAAGTAAGATTGCCTTTTCAAGGCTAGAAATACCAGCGGTGGGTGGGATACCTATTTAGCCGAAGTATTTCGAGAGTG

ATTGCTTGAGCGGTGTGAGCTGAAAGGTTCACGCACCGTTCTTATGGGGAAAATCCGAGAGGATCTACCTATCCAAACAT

AAAAGTCCGAACGGGTTATCGTTGTATAGATATCCGAAGAACTGTGGTAAGTTAGTGAAAGACAACACTGACTAGTATAG

CTGGTTTTCTGCGAAACCTATAAAAGTAGGTAATTTAAGTAACATCATAGCAGGTACAGAACTGTGTTCTCAGGCAACTT

ATCTTCAAATCCCCGCCGATAGGGGATAAATAAATTAAAAGGGATTTTTGCATATATCGGGCAATCGTGAAGATTTTATC

GGTGAGTAAAGGCACTCGGAATGGTTATGATCTTTATTAAATAATCGGACATAGTACGCTAAGGTTGTATGTCTAAAGGG

AAACAGCCCAGAACAAGAGTTAAGGTTCCAAATTTATTGTTAAGTGAAATTAAGGATGTTTCTGTAAAATACGGCTAGGA

AATTGGCTTAGAAGCGGCTCATTTTTAAAGACCTACCGGATGATGTGGGAAATCATCTATGTGGGATCAACTATCAAACT

CCGGGGACTTCCTAAAGCTTCAGATACCAACCTATGGAGGCGGAAAAGCCCTAAGTGGATGAACTAATTGCTCATGTAAG

GTAATAAGTCTGAAGATATCTGAAAAGATAATGGAATATCGCGGATCTAAATCAGATTTTATCAACAATAAAACCATGGT

TAATTTCTGTAAAAGAGCAACGGGTAGACGGTAGTTGACACAAGATAAATTATCTTCTTGTGTTTAAGGTATACTCTAAT

GGATTTCTAAAGAAATTATCGAGTCAGAATCCCTTCTTGGTTAATAATAAACATGTAGATATTACATGATATAAGGCAAG

TGCACCTTTATGTCTAACCAGAACTTTAAAAAGAGTGAATATTAAATTTTTAGTTAATACTTTTAATTCCAATTTTAGTA

CTTCGACTATAGGTACCAAAAATTATCTGGTTGATGGGTAGCAGGGTTTGTAGATGCTGAAGGTTGTTTCCGTATTTCTA

TAATTAAAAATAAAAATTATAAGGGGAATCCTTGGTCTACATCTCTTTATAGTACTGAAAAACCCGATGAATTAGGTAAC

ACTATGCCTCTATCTGTTAGACTATATTTCCAAATAGGGTTACACTTAAAAGATGAAAAGATTTTAAAATTAATTCAATC

TTCACTAGGGGCGGAAAAATATATAGATCTAATACTCGACCTGATTACGTTGAACTACAGGTATCTTCTTTTAAGGATAT

GTATGCTATTATAAATTTTTTAATAATTACCCTCTAATTACTCAAAATTGGCGGATTATTTACTTTTCAAAAAGCGTATG

AATTAATTCTAAATAAACAACATCTTACCATTGAAGGTTTAAAACATTAGTTGCAATTAAAGCTTTAGTTAATAAAGGTC

TACCTGAGCAATTAAAACTAGCATTTCCTAAACTTGAACGGCTTAATCAAAGATGTCTTGTTATTAAAGAAATACCCGAT

GGTTATTGAATTGCCGGATTTGCAAGTGGTGAGGGAATTTCATGGTTAGAATCTTTAATTCAGCTAGTCATGCTATTGGT

TGTCAGGTACAATTAAGATTTCAAATAACTCAACAATCAAGAGATAAATTTTTAATGGAGAGATTAGTTAGTTATTTAGG

TTGTGGTTTTATAAGTAAAGGGGATATACTTGATTTTCAAGTAACCAAATTTGTGGACATAACTGATAAGATAATTCCGT

TTTTGAAAAGTATCCCATAATAGGGGTTAAATTTAATGATTTTAAGGATTTTTGTATAGTAGCAAACCTTATTAGGCATA

AGGAACATTTAACAGTAGAAGGGTTAGAAAAGATTAGAAAACTAAAATCTAAAATGAACACGCTTAGAGATATTAAGAAA

TCTTAATATTTTTTTACGGCCTGCGGTTTACACGTCGACAAGGTAAACAAGCCTAAAATAAAGTGTGCTTCCCTCCTCCC

CTTCAAGAGGGAGGGAGGGCCTCAGGTAGTAGTTAGTAGGTCTTAGTAATCGATATCTAAATATATGTAAGTTTTCGCCC

AGCCCTATTTTATCCCAAACCTTCGGATCCGCCTTAGCAGATCGGGGATCTGCGGTTTTTCTTATAAGAGAGATGAGAAA

AAGAACCAGAACCATAAGGGGAGGGATGATAAAAACTATTTTTCCATGATAAATCTGATAGTTGTGCGTAACAGAGCACT

GGTTTAGTATATTCAATCCCCTCTGCTACGCCGCCATAATCGGCGGGGATACGGCGGGGAGTTACGTGAATAGTAGCACC

GAAGATTTAACGGATCTAAACAATATACCGACACCTGCGCTGAGTGATCTAGGAAATCACTTGTGTGGGTATAAACTATC

AAACTCCGGGAACCTCCTAAACCTTCTGGTACCAGCCCACAATTGAAAAATTGTCGGTGGATGAATTAATTGCTCATGTA

TGGTAATAATCCAGAATGTGGTTGAAAACCAATGGAATATCGCGGATCTAAATCAGTAACAGGGTGCTGCTTCTTCGCTC

TAAATAAACCTACAAGCCACAAACCTGTTACTGAAAAGAGCAACGAGTAGACGGTAGTTGGTACTTTTGCAACTCTTTTA

CTTCAATCCAATGAATTAGAAAAGTAAATAAAAGTATTTGAGGTGTACTCTAAGGAGCTTCGAAATAAATAATCAAGTAA

AAATCCTTTCTAACCAAATAAGACAATGCTCAACTCAAACTTTGGCAACTGTGCGCTCAAATGATCTTTTTAACCATTAT

GATCCGTGACTTATAACAGGATTCACGGATGCTGAGGGTCATTTGTTGTTTCAGTTTCTAAATACCCTGGCGCACGTTCA

GGTTGAAATATCCAAGTAAATTTTAAAATAAGCCTGCATACGAAAGATTTACCTGTCTTGAAAGAGATTCAACGCTCTTT

AGGAGGGTAGGTAAAATAGCTAAAGCAGGGAAAGACAGGGATAGTTATTCGTTTGTTGTAAGTTCTAGAAAACAAATAAC

AACTGTAATCTTACCTCATTTCGATACTTATCCTTTGATTACCCAGAAGAAAGCTGACTATGAATTATTTAAGCGTATTA

TTGAAACAATGAATAACCAGGAACATTTAACTGATCTTGGTCTTCAAAAATAATAAATCTTAGAGCTTCTCTCAATTTGG

GTTTATCAGAGGGATTAAAGTTAGCTTACCTAATACTATACCAGTAGCCAAACCTTTAGTAATCTTTAAGGGAATCCCAT

CTCCTCAATGGGTCGCAGGATTCGTAAGTGGTGAGGGTACTTTTATGTTGTCACTAGCGAATCTAAGACCGAAGGGTTAA

ACGTAAGATTAAGATTTATTATATCTCAAGACTCTAGAGATGAGCAGCTTATGAAAAGTTTAATTACTTTCTTTGATTGT

GGTAGATGTGAAATAGCTAATGACGCAGGGATGGTGTATTTTCGTGTTACTAATTTTTAGATAATTACGTAAAAATCCTA

CCCTTCTTTAGTAAATATCCGTTAGTTGGTATAAAAGCCAAGGACTTTGAAGATTGGTACAAAATAGCTTCTATAGTTAA

AACCAAGGCTCATCTAACTAAACCAGGGTCAGACCAGATTATTAAAATAAAACACAAATGAATAAAGGTAGGGTAAATTA

CTTATCCCCGTCTTTCTTACTACCTAAGGTTGGATAAACATTTGTTAAATAGTTATGCAGCAAAAAATTTACTGTAGGCC

TGGGGATGGCCTTTCAATGAGTTTTAGTACCATGGAATATTTGTATGACAAATTCGATCGCCTTGTGTCCATAGGTATAA

ATAATTAAAACCAGTTCTAGATTTAGTAATTATTTTTCTCTAGGTTCTTGTTTAATTAATCAAAGATTAATTAAAAAATG

AACGGAGCGATTAAAAATAAAAATTCACTTTTATATTATTAATTAACGAATCTTTAGCCAGGGGTCCATACCAAAATATA

ATATCTTATTTATATTTATGGGGTAGCAGAACGTTGGGTAAATCCTGATGTTTATTGTTTTAATAATAAATATATGATTA

TGATAACCCAAGTGATAATGCTGACATGAGTAACGAAAAGGGGTTATACCCTCGCCTAAAGCTTATGGTATTTTTAAGTA

ACGGCCTCTAAGTTTACCAACCTAAAGGATTAAACGATGAGTAAATCTTTTAGTTACTAGTTAACAACCTTCCTTCTTTT

CCTCTCAGCTGGGTTTTGCAAGTCGTCTGGGCTTGAAAACTCGTCGGAGGTTTCAAGCCAGATCTCCGTTCTTGTAAACT

CTAGGGGTCTATAAGAAAAGTTGAGGGAGGTTCTGGAAAAGCGTGACGGCTAAATAAATAACCATGCTCCAAATAAGATA

GCAGCCAAATATTACTTTGGCTGCAAGGGGTAAACCCACTTTTATTTTTCCATTTTTTTATTTAGACAAAACCGTACCTA

AATACCACCTCAGGTAAGCTAGTAGAGAATACGAAGGCGTTTGAGTGAACAATCATTAAGGAACTCGGCAAACTAACTAC

CGTAACTTCGGAATAAGGAGTGCCATTCCTCTTCATTAATATGAGGTAAAGAATAGGAAGCATGGAATAGTGTTGTACGA

CTGTTTAATTAAAACAAAGCACTTCGCAATAAGATAATAAATCAAAGTATTGAGTGTGATTTCTGCCCGATGTCGGCCGG

TTAACGGATTTACTTAGTTGACTAATATAAGCTAGGTTTTGAAGGAAACCCCGATGAATGGCGGCCTGCTTTGAGATGTA

GGATATCTCATGTGTGGGTAAAACTATCAAACTCCGGGAACCCCTAAAGCTTCTGGAACCAAGCAATAATCGAAGGGTTA

TTCGTGGCTGAACTAATAACTCAGATAAGGTAATAAGTCAGAAGATGATTGAAAATGAAAAGGGATATCGCGGATCTAAG

TCAGCGACAGGTTTAAAAATACCCACAAGCCAAATAATCCAACCTGGAACTGTAAAAGAGCAACGAGTAGACGATAGTTG

ATACATTAAGCCTTCTTCTTCTTATTTAAGAAAAAGTTAATGTATTTAAGGTGTACTCTAATGGGCTTCGAAAGAAGTTA

TCAAATAAAAGTCCTTTCTAAGCAAAAAGATTTTATACAAACTATGGTTCTGAGGAGTTAAATCCTTGATTTATCACAGG

ATTTGCTGATGCAGAATCAACTTTTAATATTTTAGTTCAACCGCGTTCTGATAGTAAAACCAAGTGAAGAGTAAAAGCTA

TATTTGCTATTGGTCTTAATAAAAAGATAGAGTAATATTAGAAAATATTCAATCTTGATTTGGAGTTGGGCGTATATATA

GTTCAGGTACAAAAGTTTATTATAGAGTAGAATCATTTAAAGACTTACTTGTTATAATAGAACATTTTGATAAATATCCT

TTAGTAACAGCTAAGAAATTAGATTATGCTTTATTCAAAAAGTTTTTGTATAATAAAACTTAATGAACATTTGACTGAAC

AAGGTATATCTAAATTAATTGAATATAAAAGTTCACTTAATAAGGGGTTATCTCTTAACCTAAGAGATAGTTTCAGTGAT

ATAGATTTACTTGAAAGAGTAAAATTTAAGTTTGATGGAATACCTAGTCCTTATTGAATAGCAGGATTTGTGGGAGTGGG

GATGGTAGTTTTAATATAAAGACTACAAAAGTTCGAACAGGTAAAGTACAATTAAGATTTGCGGTTCATTTACATAATAG

AGAAGAAGAGGTAATCAAAGGATTAGCTCAATTCTGGAATTTAGAGGATAATAAGTATATTTATGGGCAACTGATACTTC

AGTAACCGTTCAAATTGTAAATACCTCGGATATCTTAAATATTATAATACCATTCTTTGATGAATACCAAATCAAAGGAG

TAAAAGAATTAGATTTCATTGATTTTAAAAAGTAGCTGAAATTGTTAAATCTAAAGACCATTTAACAGAAAATGGTTTTA

GTGAAATCTTAGTTATTAAAGATAACATGAACTTAAAAAGAAAATAAAGTTACTGCTTCTTGTTTACACTGCAGTTCCCC

GGGAAGTAAGTATAAATATTTTATGTACGAAAAATTTTATCAGCTATGTACCTATGAGGGTCCTAAGGTAGCGGAATACC

TTGGCCGTTAAATGCGGTCTTGCATGAATAGAGAGCTATTCCTAATTTAACCGTGGCGCCTTGACGGCAAACGATATTAA

TAATCCCTCACTTAAGCAAGCCCTGCTTAGTAACCGGTACACGTGCGACTTCATACCTATAGATGTATGAGATTTGGTTA

TCATCTTAGAAAAGGGGAATTATTCCTAAAAGCTACTTAGAAATCCTATATTGAAACATATAGGAGAGGAAACTTGGTGC

TAAAACATGACGCATCTGAACTAAGTAGAAAACAACCATGTACCTAAACGTCAAAAGCGTTTAATTTTTTAAGTAAGACG

CACACGAGCGAACTCAGAGGTAACCCAAGTTGAGGGATTATTATTATTCTCCTCTTATTATAACCCCGTGGAAGCAGAGG

AGGGGAATGTAGGTAAAAATATAAACCAAACCAAATAAAAACATGAATAATCTCAAATTGATAACCAAATTAAATAGATG

CTTCTCCAGTTGACCCTCAATATTAGGCAGAGACGGACCTATGTCTAATGCGTATTGTTTAGCTATTCAATATATTAAAG

AAAGTAGACCTATAACTGCGGTTGAAGTTAATAAAGTTTTAGCTTTTCTGGTATTAGTATTAGTCAGGACATGTTAAATA

AAATACTTAGTAGACCTAGATTAGATTTTGGCGATCTAGACTCAAATACTATAAAACCGCAAAATTCTTACAAACAATAG

GTACAGTTAGAGGTAAGGTTCAAGTACCAGGTGTTTACATTTGGATTCATCTAGTTACAGGCGACATGTATGTAGGATCA

TCTTCTAAATTAGCTCGTAGATTAATAGGTTATTTTAATAATCACCATAAAGATACTGGTAAGTTAATACCTTTAATTAA

AAAGAAGGTGTGGGTGCTTTTAAATTACAAGTTATACCTTTAACAGAGTCTTATCAAGTGAATCAAGAACTTTGTTTAGA

ACAATACTTCTTGTTGCAATCTAAGTTTAATCTTAACACACTTAGAATAGCTAACGACTATTCTGGTGCAAGAGCCATAC

CTTTATACATGTACACTAAGGACTTGTCAGAGTTGATTTACTCTTCAAACATCCAAGAGGATTTTATATTTAAATTAAAA

GTGCATCATAGTATATTTAGCAACAGCTTAAAACCGGAGCAGTTTACTTAGGTAAATATGTATTCACAGATAAGCCTATT

ATTGGAGCTAAAGAAAGCAATATGTCAGAAACAGATGTAAATTTAATGTTGGATAAAGATAGATTGGAAATACAAAAAGT

AATCCCTCTCGTAGGAAAGTAATTATAAAAGATATTGAAGGAAGTAAGCAAACTATGCAGTTTGATAGTATAAGTAGTTG

TATAACTTTTCTTAACCGAATTGCACCCTCTAATAAAACAACATTACGTAGACACATTGAATTAGGAAAACCTTATAACG

GTTACCTGTGTCAATGAGATACTGTAATTAATGTTACTTCTATTATGGACAAAAGTATAGAAGTCCTAGTCACTCATGTT

CCATCAGGTTCAACTGCTATATACCCTTCTTTTAGAAAGGCAGCTTTATCTTTTGCACCGGATTTTATAACTACTGGACA

AACTCTTAAAGCTTTTGCTGAAAACGGTAAGCTTTTAAGGGAGAGTTTAAAATAAGTCCTCTCTTAAAGGATAAGCAGTA

GCGCGGGGCCCTTCTTGCTTGCTGCTCCTTTTATTAAGCTTTATGGGGTTTATATTTTATCATTAAGGGATACGTCCCTT

TTAATGATGACCTTATCACACTGGGCGCCTAAATAGGAATAGAAAAATCGAGCCTAGGAAACTCGATTTTTGTGCAATAA

AGAGGCAAACTCTGGGGACACCCTAAAGCTTCTGGTACCAAGCTATAGTTGAAAAACTATATGTGGCTGAATTAATTCTT

CAGATTTTTGTTAGGGTTTGTCTTAATGAATAACCTAAACAATAAGGTAACAAGTCAGAAGATGAGTGAAAACGAAATGG

GTTATCGCGGATCTAAGTCAGATAATATATCTGTAAAAGAGCAACGAGCGGATGCTTCTTCGTAGATTATACTAAGATAC

TTAAAAATAAGAAGTCTTTGCCCATCTACGTAAGGTGCGCTCTAGTCGCCACGAAAGTGGTTCTTAAGCCAAATATAAGT

AATTTAAGATTAAAGATATCATAATAACCTTGCCGGGCCCTCCTTCCTTATCCCCTTAGCGGGGATTAGGGGAAGGGAGG

AAAGATAACATCAACGGAAAAATTATGAAACGGAATGATTTAACGATACAACAGCTGTCTCAATGATTGGCTCAGTGAAA

TTGGAATAACAGTGAAAATAAAAATGTTTTCGCTGCGTAGGTGAAAATCCTACTCTACAGAATAAAAGTAGTTTGACAAA

TCATCTAACTTTTAAAAGAACGTTTCTCGGTGGCGGTCCAAAACCAACTGCGGATCGCAACCTTAGAAAAAATCCCGAGT

GCGAGCTACCTTTATTTTAGTCCTAAAACTAGAGATTCCTGGCGAATAACTAAATATTGTTCCAAAAGTTCTTTAACCAA

AGAGGTAGCCTTACCTGAAAAGGGGTGTGCCTCCTTTTATCAATGATTCGTGGGGTTCTCTGATGCTGAAAGTAGTTTTA

GTATAGTACCTAAATCAAATCCCCTCGAGAAAAGTAATATTAACAGGTTCTCTTTATGTTTAAAATAGCTCTTCACAAAG

ACGATATAGATGCTTTGATTAGAATTCAAACTAATTTAAATTTTGGAAAGGTTAGTTTAGATAAAGATGAATGTAAATTT

GTTGTAACTAAGCAGGAAGAGATTAATAAATTGATTTGTATTTTTGATAGATATAATCTTAATACAACTAAATATTTAGA

TTATATTGATTTTAAGAAAGCTTTTCTCATATATCAAGGTAGAGATGGTTTAGTTGCACAAGAATTAAAAGAACAAATAC

TTGCATTAAAAACGGTATGAACACACAACGTTCAGACTTCAATATGCCTCTAAACCATCGGATTAGTATTAATCAAAATT

GGTTACTCGGACTAATCGAAGGTGAGGGATCTTTCCAACTATGAAGGAAGGATTTAGTTCCTGTGTTTGGTCTTGTGATG

ACTGAAAAACAACTACCTGTATTAGAGAAAGTAAAAGAGTATCTGATAAATAACTTAGGTTTTGATTCATATTCCATATA

TAAATTAAAAATTCCTCAGCTATTACTATAAACCATCAAAAGCTAGAAATAACTGTAAAGCAAGTATAGTACTTATTATT

AAGAATATTCATGTTTTACATAATCACTTTTACCCTTTTAGGTAGGGAGATTTAAACTTTATTACCAAAAGTATCAAGAT

TATAAGGATTTTAAAATTATATGTAAAGCAGTATATTTTGGTGCACATAAAATATCCAAAATTAAATCCTTAGTTTTAAA

ACTATCCTTAACCATGAATAATTACAGATTATCCACAAATACAGATCCAGATAAGCAATTTATTGATAAATTGGAAATGG

ATACACTTGTTAATATATCTCCGACTATAGAACATCTTAGTGATGGTAGGCAAAGAGATATTACAACTAGAAAAATAGTG

CACCAACATACGAGTTGTGTTTATGAGATAATCCATGAGTCAAGGGGTCTAATAAAGCTTACATTATCTGATGCAGCTCA

AGTAATAGGGGTAGATATTAAAACTTTAAGTAAACATTTGGATAAAGGAGTTAATACTGTAGTGGACGTAAAAGGTTCCC

GGGTTAAAAGAGTAAGGGTTTTCAGCGGGATTGCAGCTTCTAAATAAAGTTTCTTACATGTGTCTTACGAAATAAGGTTA

TAACCGAATAGTTCTAGTTGTCAAGCCAAGCGATAGGTGAGACAACTAGTTATTTTGTAGAAAAATTAGGTGAAAACGGT

TAATGAGATCCAACTTAAAGACCGTCGGCAGTTATAAATGTCGCTACAGACTGGTTCACCGGGGCTAGACGGAGTTGTCT

GTCCAAATGTACAGTCGGCTTCTGTAACGAGAATAGGTAAGCTCGTAAGGAGAAAGGATATACCTTTGAAAGGGTAGGCC

ATAACACAGTGGTAGTAACTCCTAGTTAATCAGAATTGGCAGATACTGTTTACCTCTAGATAGACGAGAGTGCGACTAGA

AAAGTGCTTAACAACAATGTGGTGAGATCCCACCAGATAACCCATCATCTGAGCTCAAACGGTATGCATTAAAAGTAATT

TTTTGTGTAAAGCCCGTTAATAAGTATTTAATACGTCCTTCCAAATCCCCTTTAGGGGATTTGGATGGTGAGCTATATAG

CTAGGGATAAATTTTTGAATTCACGTTTGAAGCGTAGTCTAACATCAAAACCTAATGGTTTGATGCCATTGATTGTATAA

ATAGTTTTTCGTCTATTTATTAATGGGTCTAATGTCAATGTGCGTATAGTGAGATCCTAAGGCTATAATTAAAGTGTTAT

TAAGTAAACTAGGTAAGCCCAATAATGCCCTTTTTATTTTTATCTTTAACTGAGGACAAAGATAAAAATACTAAGGAAGT

TTAATTGTGAAATTAAATATACATTAGTGGGTAAAGGATGTTCTAAAAGCGAATGTTATGTTGTAATGACGTAAATAGGC

TTTAAAAATGCCGGATGGAATTTTTTTAAGCTAACCTGAAAAGATGCAGACTTAGAACAGGTTTTATGTTATATATATTA

TATAGTTTTAAAACCACAATTTAGATATTATGAAAATGGCAAATAACACATATACAGATAATAAAATAAACGCATCAGTC

GCAATAAATGATATCCCTAAAGCCTTGGTTAATGATTGGTATGTAACAGGTCTTACGGATAGTGAAGGAAATTTTAGCAT

AAATTACAACAAGAATACAAATAAAGTAACTTTTTCTTATAAAATCACACAAAAGTACATTCTATAAGTGTCTTACATGA

TTTAAAAGCATTTTTAATTGTGGTAATGTAGTGATAGATAATAAAGATTCTAAAGGTTATAAGTTTCAAGTTTCAAGCTT

GGATAGCATTATAAATATTATAATCCCTCACTTTGAGAATTACCCTTTACAAGGATCTAAAAGGCTAGATTATTTAACTT

GAAAAAATAGTATAATTGATTATAGAAAATCCCAAAATATTGATTCAGTTTTGGAATGTAAAGACAATATTAATTCTAAA

AGATTATTTGATGAAAGATGGCATTATTTCAATAAAGTTAATATAAACTTAGATCCAGAGTGGGTAAGAGGATTTGTAGA

TGGAGAAGGTTCATTTCAAATCAACATAGGTAATTATAATAACAGAGGTAAACCTTTATTAAGGGTTGCAGCTACATTTG

AAATAGCTCAAAACAGTCATGAGATTAAATTATTAGAAGCTATAAAATTATTTTTAATAGTGGTTATTTAAAACCTAAAT

ATAATATCGAATCTTTGGAAGATGCCAAAAGAGTTCGTTCTGTTTCAAGATATGTTTCAAATGATAATAGGGTTGTAACA

GAATTCTTTGATAAATATCCTTTAAAAACTATTAAAAATTTCGATTTTTAGACTGAAAGAAAATAATCAAATTGAAGTCT

GAGGATGCTCATAAAAAGGTAAACTAGATCTCATGATTAGTATTAAAAATGCAATGAATAGGAATAGATTTAAATAGTAT

ATCTTATAAGGCCGCGGCGCTTGTGTAAAACCTGGGGAACTATATATATATAACATATTGCTTGAGCCGTATGCGATGAA

AGTCGCACGTACGGTTCTTAGAGGGGAAATTTTAGTAATAAAACGACCTATCTCGCCAGACCCTATGCAGCTTTACTGTT

ACTAGTTATTAGATATGATAAACAGCATTTTTAGTGCATAAGGTAGGTGAACGGGTTTAACTATTCTGTCACCAAAATTG

AAACACCTTTATTTAGTTTTTCATATTGGTAAATATATTTTACGTGATTTTTTGAATCATTTCCAAAAGAGGAACGATGA

CTAGGAGGCAGTTTATGCGGGGCACAGATCCCATAAAAGTACCTGGGTGTATCCAAAGTTAATTTTGTAAATTAGTTATA

ATTTAGTTATACTTTTTTAGTTTGTTATTAGAGATAGCTCTACCATTGGAACTATATCCAATCCACCTATATATCTATTG

AGTTAGAACGAATTTTTAATCAAGTAAGATTTTATCTATATTGAAAATAGATGTAAACTATGTTCCCGGGCGATTTATAT

TTTATATTTAGCGCCCCTTGGAGAGGGAGGTAACGCACCATCCGCTCATATAAAAAGGGATTTATATATATCATAGTTTT

CGTTCCTCAAACTTATTTTGAGGGACAAACGGGTTTCGTGCCCGGCAAACTATTGGAGTCCCCAATAGGGAGCGGAACTT

GTTCCGCTCCCCGGAGATGGATGATGGATGTCAATCATCGGGGGATAAGGACCCTAGAGGGAACTAGTTATATTATTGGT

CCTTAAGTTTTTAAAAGCCTTATTCTTGTGCTGCAAGCAGCTTAGGAATAATGAGAATAATATAATAATTACTTGTCAAG

TTTAATGGCTTAATCTTGCTTTACTGTTTGATTTACACGTCTATCAGTCGCGTAAGCGGGCATATGATCACAAGATGCAG

AAAGGAAAGGTCTTGGGTTTTGAAAAGCTACGCTAGGGATAGGTTTTGTCCCTACCTAATAATAATACAATTGGCTACCC

CGCACCCGAAGGGGAGGTACCTTTAGTATTTAAAGGGGTTTAATTGAGTTAATTTCAAAAGACTAACTAGCCATCCCATC

CCATCTTAACATCTACCTTTAGGCTTCCTAGGGGAGGAAGCGCGGCTCTACTACTACGCGTAATCCCGCGCTAGGGGAGC

TCCATAGGGGAGCCAGGGGATAAGCCTCCCGCCTTAGCCGCGGGGAGGGCTATCCCTGGGGGATACGGGATAAGGCCTTC

GTTTAGCGTAAGCAGGGGCTTAATTGGGGAGAGCAGGGGTACCTTACGCCTAAATAGATCGGGGATCGATCGGGGATCGA

TCGGGGATCGATGGGGATCGATCGGGGATCGGATCGATTGGGGATCGATGGGGATCGGATCGATTGATTGATTGATTGAT

TGGTGGTAGTGGGCTATCCCCGGCTACGGGGCAGGATTAATTGGTTTAAGCGTAGGGGATAAGCGTCGCTGCCCTGATAA

AGCAGCCTAAAGATAAAGCGGTGCTGACCTCGCTCCTAGGGGAGGTAGGATATGATGGCTTTTAGTTTATTTGAAGCGAA

GTTTACTTTATCAAAAAATCCCGTTTTGGCAATCCAATCCCGCCGATTTTAGCCGCAAGCCAAAAAGTTTTTAGGGAGGG

CTTTGCAGGGGTCCCTCAAATTTAGAGCCCAGGGGCTTACTAGCCACGGGGGCTAATCCCTAGGGAGGGTAGGCCTGCCG

TAGGGGATAAACCATAGGCTGCGTAGCCCGCGTAGTGGGGTAACGGATAAGGGGGACTTGCTTTTCTTGCCTCGGGGAAG

AAGCCCTTTCAGGGCTCCCTGCCAAGGGAAGGTGCCTAGGGAGGAAGATAAAAATTAAGAACGTTCAACGACTATTAAAG

ATTGCTTAGCTTTTTGGGATTCTGGCTAGCCTACCAGCATACAAATTTTAAACCTTTCTTTTATAGGACCACTAGTTCTT

TGTACGGTTCTATAAAAACTCGGCTGGCTCGCGAGCAGCGAGCGCAAGGTAGGCTAAAGGTTTAGCTGGGGCCCTCTATG

ACGCGAGAAGCCCCTCTAGCTACGGCGGTATCCCTATGGTGGGGAGCCTAAGGAGGGAGGCTATCCTAGCTACCCCGATC

TAAGCTGGGAGGGTAGGAGGTAGGATAAGGGTTTAAGCGTAGGGGAAAGGGCGTGGGTAGATACCAATAAGGGGACAGCT

TTCGTTTTGCTTTAGTAGGCAAAATTTAATGGTTCTCAACATCCAACCCCTCCGATGATTCGAGGGGTAAAGGGACTCCC

CGTAATCCCCGGTTTTAATTCCCCTCCTCTTAGAGGAGGGAGGGCTTATCCCCTGTTTTCATCCATTGGGGTTTTGGCAG

GGGATAAGAGAGGGGGATAAGAGGGGAGCTCAATGTTTATTAAATATAATATAAATTTTATCATCTGTAATCATTAAATA

TTAATGTAATAGACTTAATTAAGCTCAGGTAAAATTAAATAATGCCCACCTATAAAAGAATAAATTTATAAAACGAACTC

TTTCTAATCAAATCTCCAAAGAAAACGACAACTTAAACTTACCAGTTTTATTTAGTAAAAGAAGGGGAAAAAATTCATGA

CGATGACGTTGACGTTGACGTTGACAAAAATTCATGACCTCCCATATTCTAAAAAAGACTCTTTTAAAGAGACGTCACTT

TGTACCTGCATCACAAGAATGGTATAATAGTATTTACACTTATAATAAAATTACCCTAAAACAATACCAGTCGCAGATAT

TAGTCTTATGAAGTTGTTTAAAGGTTATTTTAACTATGGTATTAGATTGGGGAAATTATTAAAAAGCCGAAAAAGCCAAA

ACCAAATTAGAGCCTTACCTATGAGATATAGATTATTATCTACCAAAAAGCTTTTGTTGGTAAAGGTGATTTAAAACATA

CTAATAACCAAGTTATTATTACTTTTATTTATATAATGCACAAGAGATGTTTATATCAGATAATTTTAATAGTAGAGTAA

AAGCATTACTTTTACCGAATAAGTCTTTAAAATCCTAAGTAATGAGCAACCTCCGGTGGGGAAAAATTATCTTATACAAC

AGAATGTTTAATATATATGAATTTTTAGCTTTGGATGAACATTATGAAGCTTATTATAATCAAATGGCTTCTATCATTAA

AAAGAAATATTGTTTTAGCAAATATAAATTATTTATTAGGAATTTATATTTATATTCTTAAAACGAAGTTATTTAATGAT

AATTACAATAGTAAAATATTCTCTAAAATTAATTCTTTAGTTAATATTTATAATAACTTACTTAAAACTAGTTCCCTCGA

GGCGGAGGGAGGTGCCCCAGCGGGTCGAGCCGAACCTTAGTTCCCGCCTCCTAATCCCCGCCTTAGCGGGGGATAATAGG

GGACTAGCGTTTTGCAAATGGGGGCCACTAAACATCGCGTTTTGGGTCCCTCAAAACCAGAAGAAGTTTTGAGGAACGAA

AAACTAGTTCCCTCTCCGGAGGGATCTTATCCCGCCCCTTTTCTTTTTAATTAAAAGAAAAGGGGAGAGCGGAATAAGTT

CCAGCGGGACTAAGGGATTAAAGGATCCCCTCATTTAGTTAATAATTTATTCAATAGATTCTTTAATAAAAGGCTAGCTT

TTATAGACTAAAATTAAAGACTAAATTAGCTCTACAGAGAAGGGGTTTTAATTAGCCCTCCGGCGGCTACGCCCCAATCA

GTAGTGGCTTATCCCCGGGCCAGGTAGGCAGGGGATACCTGGATTAGGGGAGGGCAGGTTGTAGCCCTCTTAATCCCGGC

GGCGGGGCGCAGGGGATGGGACAAGCTCCCTCTACGAGGGAAGAAATGATATATTAAAGATTGCTGCTCAAAGCAAGGCA

GGAGGGGAAAATAATAACTAGTTGCCTCGAGGCCTGCGTAGCGGGGAGCTTCAGGTGCCCCTCCTCCGGGTCGAGCGGAA

CCCTTCCCCATTGCGGGGATAGGGACTAGTTCCCTCGAGGCCAGCGTAGCGGGGAGGTGCCCCAGCGGCAGCTCGAGCGG

AACAGGCTTAGGGACTAATAATAAATGGGGCGGGGAAAATTCTTTCCCCATAAAAATGAGCAAGCAACGGACCCAACCCC

AATTTTTATAACTCTAACCCACCCTTACGCTAATCCTAACCCGGGGATATATTTTTAATTTTGAATATTATTTGTATAAA

ATGTCTTTATCTTACTTAGAAAAGGTTTTTATATTGAAAGTTAATAAGATTAAATAAAGTTAAAAATACCGTTTTATATA

TATCTAAATTTATTCATTTAATTGAACAAATATATCACAAAAATTAGATTGAATATTGTTAATTTAAAAAATGCATCTAA

ATAGTCATATTTATACGCAAATTGTGTCTTTAAAACTAAGAAATAGAGATAATAAGTTATATAGAATTTTAAAAGCATCT

TTAAGAAAGGTTAAAATAGAGGATTTTTCAAAAGATATTACAAAAAATCTCTTTTCGGGTGGTTCGCAATAAAAATTTAA

ATCCTTTTTTAATGAGTTCTTCTAGGCGGTTTAATGTAAAAAGAGACTTTAAGCGATTTAGTTTTCAAATTTTTCCCAGA

TATAAACGACGAAAGCAGCTCTTTACTCTCTTCTTATCCCGCTAAGGCGGGGATCCATCGTGAGCCAGGAGCATCTGGGC

GGGATAGAGATAATGATATAACAAAATATGTTCTTAAATTTATAAAACATATTAAATTAAGAGGTATAAGAGTAGAAGCT

AAAGGTAGATTAACTAGACCTCGAACTGCAGCAAGATCTGTTTTAAGATGAGATATTTGGGAGGTTTAAAAAATTGAATC

TTCATTTCTCGGATTATCGACGATAATGTTAAGAGGAGACAAGAAATCGAATGTTAGTTATTCAATAATAAACTCTAAAG

GTCGTAATGGATCTTTTGGTGTTAAAGGCTGAGTAAGTAGCTTGTAAAGCTGCGGGCTTGCTTTAAGCTAGTTCCCTCTT

ATCCCCAGGGATTACAGCCGGCGGAGGGACTTATCCCCTCCGGGCTTCGAGCGGAATAAGTTCGCCTCTAATCCCAGGAG

CCGGACTAGTGATTTGCGTCCTGTTCTGTTCCTCAACGGGGAACTCCTCCTGTCGTTTTAGGGACCAAAACGGATCCCAT

CGGGGTTTCGTGGCCCGCCCGCAAAACTAGTTTTGCGGGGAAACTTTTTCCCTCAAAACGGGTTTCGTGAGGAACGCAAA

ACTAGTTTTGCGGGGGTAAGGGTTGTAAAGCGGCTAAGGGGAGGCAAGTAAAGATGCTTGTGGTTTAATTGCTAGTTACC

CCTTTTAATAGGTTTTCAAGGAGTCTAAGGGGAGGAGGTTAGGCTCCTCCCATCGGGGATGGTATCCCTTAAGATACCGG

GAATCCTTAAACCCCGGAGGCTGCTAAGGAGACAAGTGGCTCCTATGACTCGGTTTTAAAAAGACGGAGGGAGGAGCTCA

AAGGTTTTTATCTTCTTGCGGCTATCCCCAGCTATCCCTCTATTCTCCCTCGGGCGTAAATGAGATTGAAGGTCATTTTT

TAAGGAGGAAAAAAGGGCTCCCTAGGGCTCCCTCAGCCGCCTTTCCTCCGCAGCGGTGGGAGGCCCTCTTAAGAGGTGAG

GGTCTTTTTTCGGTGTTTACTTTTCCTGGGGTGGCTAGCAAGGGGCCGCCTGCAGTGTTAAAAGGAAAAAGACGATAAAT

AAGGGGGATAGGAGGAAGAACTCTGGGGTTGGGTTCTTCTTTTCAGTGCAGAGAGGGGAAAAGACGGGGGTAAATAAATG

GAGTGGTCAAGAGAGGGGGTTAAAAAGCCAAATCCCACCAATCCATAGGGTTGTCTTCCAAGAGCAAAGTTTCGTGGCTT

GAGCTTGGAAAGCCCGGGGAATTTCTCTGCCTTGCTCCAGCTTGAGCTCATGGGGAGGGTCAGCAAGAGAAAAATGGGCA

AGGTTTTTACGCCTTGGCTCATCGGGGCCCTTTGTGTAAAGATACAAAATAAGATAAAAATCCTTATTACAAAAGATCAC

GAGTTGCATATTATATTTATTTAAAGGATGATTACATAGTCTGAGCCATTTTGTAAAAATGGAACCATTTGGTGGTAAAA

ATTAACTAAATTGCCTCCACTACCTACCTGATTCGATCGGGGATTAGGCACTTCCTAATCCTGCGGCGCTAAGGCGGCGG

GCTCCCTTAGGCATCATCCCGCGCAATGCATGCGCGATACGGGAGGAGGGATACCTCCTCCGATGGGGATTAGCCTTACC

TTAGCGCGCTAAGGTAAGGATGCTGCTGCCTGCTGCTGCTAAGGATCGATGGGGATTAGGGGTAAGTTCATGTTCCCACC

CACCAGGGGAGCATTAGTTGGCTTTTTCGTAACAGAGTTTGAACAGGCTAATTGCGCCAGGAAGTACAAATTGAGTGCGC

AGTTTGGCACCTCGATGTCGGCTTAACTTATCCACATGAACGCAGGAATCATGAAGGGTTCGACTGTTCGTCGATTAATA

AGTTACACGAGCTGGGTTAAATACGTCGTAAGACAGTATGGTTTCTATCTTCTAGAGGAAATTAGAATAAAATAAAGATA

TATCCCTCGTACGAAAGGAGCTGGGTATATTAACCTATGGTTTACCTGTTGTTTATGTGGCCAATATTAAAGTAAAATTT

AAAGATAGCCATTACATTGGTTGGTCTTTTTTACTAGTTTTGGGACACGAAACCGTTTTGTCCCTCAAAACAACTTGTTT

TGAGGCGGTACGAAAACCACTAGTTCCCTCTCCGAGGGACCGCGTCCCTTGGAGAGCCGGGTACCTAGTTCCGGGCCTCG

CGCGCGGGACTAACCTGGAAAGCACTAGGATGTTACTAACACTTTAGTAGATATATAACATAGGCACGGCAGGAACGCTA

TGTTAGTTAAAATAAGTGCTGAAAGCATATAGGCACGAAGTTTACTTTAAAATATTCTTAAATATACGTCAGAGAATACT

ACGAAAAATGGGGGTTTTCATTAATAAATATTTCCCTGCGATCGATAAAAACTAATGTGGTTGTTAAAACTAACCTTTAG

AAAAGCAGAGGGTTGACTTCACCCCCAGATCGGGGGTAGGTTCTTCTTTACTGAATCAGTGCTTGGGGAAAAAGACGGGG

ATTGGATTGAGCATTTGCCAAGTGCATGTGACTAAGGGCCGCCTTGCGAAATAGCCAGGTCAAGCCTAGTTCCCTCTCCA

AGGGACAAGTAAGTCCCTCGGAGAGGGATTGTGGGACACGAAACCCGTTTGGGGGTCCCTCAAAACAAGTTCAAACTTGG

GGTTTGGCATCCAGTGCTGTTCTCTCCTTCGGAGAGGGATGCAGAGGTCAGAAAAACTAGTCGTCCTCTTTTTCTCTAAT

CGCTTAGGGGAAAATATCTTTCTTTAGGTCGATTTAGCCAGGCCTGCCTGCAGTGAGGGGTTTTATATTATTAATCCTTC

AAATTAGATAGGCTTTAGCTGAAAGAACTTAAATGTTTTAGGTATAAAGTACTAAATTTATATTTAACTAAATTATTAGA

ATATCGACATATAAATACTAGGTTTTGTGGGGGACAAACTCCTGTGGTTTTGTCCCTCTGCATCCTCTCCGAAGGAGGAT

GCAAACCCCAAAGGGTTTGAAAAAGTTCGTTGAGGAACGAAAACTAGTCCGGCGGCTCCGCGCTCAAATTAAGGGGATAC

GGGAAGCGCAACTTATTCCTTCACCCCTTTTTAATTCAAAAAGGGGCCGGGGACAAGGTAAGTCCCTCGGAGAATCGGGG

AGGGACAAAACTCCTCCGTCCCTCAAAGTTAAATCCCTCCGGATCCCGAAGGAGGGGATTGAAGTGCAGGGGAGGAACGA

AAACTAGTCCAATCGGGGGACGAAAATCATGTTCCACCTCGGGGACCAGTTCCTGGGCCCCTCAGGAACTAGTTTTTTCA

AAACTTGTTTTGAAAACAAACAAGTTTGTTTCCTGCAAACTAGTCCCCAATTGATCGGGGGACTTTACCCTCGGGACCAG

TTCCAGGGGCCCTCCGGAACGAGTTTTTAAAACTTCTGGTTTTGAAAACAAACAAGTTGGTGTTTCCTGCAAACTAGTCC

GGATCGGGTCGAGGGAGGGCCCTCCGCAGCTAAATTTCCGAGGGGAGGGACAAAACGTTTTGTCCCTCAAAAAATCTCCA

AATCAAAAGCTCTCATTTGGGATTTTTAATTTTTGCAAAAATATCTTTTGCAAAAATATTCTTCCAAAACCCCAAAGAAA

TTGGCTTTTTGGGGATTCTTTTTATCCCCTGGCTCTCCAGCCCCGATTGATTGGGGATAGGGGGTGAATTGGGAATCCCT

CCTGTCAAATAATCGGGGATTGATTGGAGTTCGTTTAGGAGAACCACGAAAACCACAAGTCCCTTCCTTCCCAATCCCAA

TCGATGATCCCAATCGATTGGAGGGGATTGAAAGGGAAGGGATAAGTCCCTCCCGGGAAATCGGGGACTATTGGAGTTTT

AGGACAAACTTGTTTTGTCCCTCTGGGCATCCCTTCCTCTCCTTTGGAGAGGAAGGATGCAACCTTTGGGGTTTGAACGG

GGTTTCGTGAGGAACGAAAACTAGTTTCCCTAATCCGTAGGGGATAAGAGGGGTTCCTCAAAACAAGTGGAGTGGGTTTT

AGGGACGGGCAGCAAACCAGTTGCTGGGGGTCCCAAAACTAACTAGTCCTTCCTTCCCAATCCCCGATTTCAATCGGCGG

GGATTGGGAAGGGAAGGGATAAGTCCTCCCGGGGAGGGACTAGTTTTTCAAAACTTGTTTTGAAAACAAACAAGTTTGTT

TCCTGCAAACTAGTCCCGGATCGGGGATACCCACCCCGATCGGATCGGGGATTACGGCAGGCGCTTCTGGTTCCGGGCTC

TGCTAATCCCCTCTAATCCTGCAGCTTGCTGCAGCGGCAGAGCTCTGCAGCAGCAGCAGCTGGCCTTTAGCGGCGGGGCC

TAAGCGCCGCGCGGGATACCCTCCCCTGGGGATAGGGGATGGGACGGGGTGCATCCTGCCTTAGCGCAGGGGGATGAGGG

ACTAGTGGTGGTGGTTTTTACCCCTTCTTCGTTCTTCGGGGCTTGCGGCTACGCAGCCTGCTAGGTGCCGTAGGGGATTA

CGCGTAGGGGAGGGTTGAGTTTCTGTTCTGTTTTTAAATACCGTAGTCCCTTCCCGATCCGGGCCAGCGGGCGATGGGGG

TATTCGAAGGGATAAGTCCCTCGGAGAATCGGGGACTAGTTTTGCGGGGACGGCCTGCGGTTTGCAGGGGTCCCTCCCAG

GGTTTCGTGAGGAAGCGGCCAGCCTGCTTATGCTTATCCCTAGGGGGATTAGGGAAACTAATCCCTATCTTTTCCTCTTC

TGGGGGTTCCTCACGAAAGACCGACCTCCCCTCAAGAAGAGGGGAGGGAAAATAAAATCCATCTGGGAAACATAAAGAAA

AAAATAGATATTTTTTTCTGTTTTCTTCATTATCCACATTAGTTAACGCTTGTCCGGTTAGCATAAAAGTAATGTGGCTG

TTTTGTAACCAGCTGAAGCAAGTGCGATACTTGCACTGGACTGTTAAACTCTTTAGGGGTAAAATATGCATTAAATATTT

TTGTCTCCAGGTTTATGCCCCTTAGGCATAGGGTGCACCAAAAAGTCTATACTACCTTCCTATAAGCTGCTCTCTTTAGA

TCTGGCAGGGGTTGAGGCTAGCATGGGGAGGGTTTTTCTTCCTTGATTGGCAGGTGGGCATTTTCTGCCTACCTTACCTT

ACCTTACCTTACCTTACCTTACCTTACCTTAAGCTTCCTAGGGGAAGCCTTCTGGTTTCTTGTTCTTGCTCCAAGGGGTT

AGCTCCTCCCTCCTTTGGGGTTAAGCCTTACTCCCGCCTTAGCCGCAGCCAGCAGAGCGAGCAGCCAGCAGCGAGCAGCC

AGCAGCGAGCAGCGAGCCGCAGCGAGCAGCCGCAGCGAGCAGCCGCAGCAGCCGCAGCAGCCGCAGCGAGCAGCGAGCAG

CGAGCGAGCAGCGAGCGAGCCGCGAGCCAGCAGCGAGCCAGCAGCGAGCCAGCAGCGAGCAGCGAGCAGCGCGCTAAGGG

GATATGCCGCTTAGGATCGGGGATCTCGGCATACTTGTCTAAGGGGAGGCTCACCCTGTTTCTAAAGGGGGCTATCCCCT

TAGGCATCCGCTAGGTTATCCCGCCGCCTGCAGGGCCTAAGGGGATGCGCATAGGGATTTGCCGCCGCCTTCGCGGGGAT

AAGGCATACTAGTAATCCCGCCCTGCGCTGATCGGGGATGGGGCATGTGCATCCCCTTAGCCGGCGCATCCCTAAGAGGA

GGGCCTGCCTTTAGGGCGCCCGCTGCTCCTCTTAGGGGATACTCCTCAATCGATCGGGGGATTAGGCAGAGGGAAGGTGG

GCCTCCTGCAGCGGGACGGGCCTGCCCGTAGTTCCCTCTCAGGATCGATCCCCTTTTGAATTAAAAAAGGGGCGGGGTGA

ATTGGGGAATAAGTTGGCGCCTCCTGCGGGACGGGGGACGGGGACCAGCGGTTGGGTTGGTGTGTGTTGGTTTGCAGGGG

GTCCCTCGCCCAGGGGTTTCGTGAGCAAGGGGTGGCTTACTTTCCCTCGAGCCGGCGCGGAGGGGCCCAGCGGCTCGAGC

CGGAACAGGTGGCCTGCGGGACGGGCCCTGCGTTGGAGTTTTAGGGGGACAAAACTAGTAATCCCGCCCAGCGGCGGCGC

GCCTAAGGGGATGCCGCGTCCCTCGGGTCGAGCGGAACCAGAAGGGCTTCCTAATCGGGGAGGGATAGGGATTGGGACGC

CAGCAGCGTTTGCTGGGGGTCCTCGCCCAGCAGGGGGTTTCGTGAGGAGGAACCCTAAGGGGAAAGGAGTATCCCGGCCA

GCTGCTCCTCCTCGTCCAATTAAGGGGAGGATAGGAGACAATAAGTCTGCTTAGGGGTTAGAAAAAAGGAGGCTTATTAG

GGGAGGTCGAAAAATAATAAGTGTTTGGCCTTAGGGGAAACTCAATCGGCTCTCCTGCTGCTCATGCTCATGGGGGTATC

CTCCTCCGGCGGCGGGGGTTAATCCCCTTAATCGGGGGTTAATGGCTATCCCTCAGGCTATCCCTTTACCGTACCCTTTA

GGCTATCCCTTTAGCCCTATCCTTAGCCTATCCCTGGGATTAGAGGGGTGGTAGCGGGGATTCGAGAGCTAGTAAGCCGC

AGGGGTTAAAACTTTGAGGGAGGCCAGCAAAGCCTCCCGGCCAAAACTCGTTTTGCTTACCTCGGCGAGCACAGGTGAGG

GACGGGGACCGCTAAGGGGGTCCCACAAAACTCGTTTTTCGTTCCTCACGGGGAACTCCGTGAGGGACAAAGCGGGTGGT

TTCGTGGCCCACAAAACTAGTCCGCAGCGCAGGAGGCGGGAACTGGGGGTTCCTCCCCGGATGATGGATGGATGATGGAT

GATGGATGATGGATGATGGATGATGTCAATCATCGGGGATAAGGACCAGAGAGGGAACTTTGCGGGGGACAACGACACCC

GTTTTCTTGTCCACAATAAATTTCGGATTTTTATTTCTTATTTCTATTCCAAAACCCCCAAAATATTCTTCCAAAACCAA

AAAGCCATTGGCTTTTTGGGGATTCTTTTTTATCCCTCCTCCACCCCTAGGGGATAGGGGTGAATTGGGAATCCCTCCTG

TCCTGTCAAATAGGGGATTTAGTTGTTGAGGAACGAAAAACTAGTCCGGCTCCCGCAATCGAAGGGGATTAGGAGGCGGG

TAACTTCTTCCGGGTCGAGGGAGGGGATAAGTCCGGCCCTCCGGCGAAATTTCCCGAGGCAACTAGTTTTCTTTCGTTCC

TCAAACTTCAATCCCCTCGGGGATAAAACCCAAATCTAAAAATAAAAAATCTCTCGATTTTTATTTTTAATTTTGCAAAA

ATATCTTTTGCAAAATATAAAAGAAAGCCAAATCTCTCTCATTTGGTTTCTTTTAATTTTGCTGCAGCGAAGCAGCAAAA

ATATCCCCAATTGGGGTTTTTGAGGGACAAACCACCAGTCAGTGTTTGTCCCACAAAACTAGCTAGTCGGGTCCAGGGGA

TTAGGGGAGGAACTAACTAGTTGCCGGGCTCGGACCCGGAGGGGACAAGCAGCTCCCTCCCCTCTAGGTAACAAGTTTGT

TTTCTACAAACCAGCCGCGGCTAGCGCAGGGGAAAGGGGCGTCTTTTCTTGCCAGACTAGGGGCAAGCCAGGTTTAGGCT

TCGCGCGGCGGGCACAGTTCTGGCGACTTGTCCAAGTAAAAGTTTAACAGGGGAAAATACTCCTCTTCCTAGGGGGATTA

AGGGTTAAACCCTCAGTTTTCCTGCCATGGGGATGGCAGGAGTAGGAGTCGGATGAGGAGTAGGGAGGGAGGAAGGGGGT

CCGTCTTTTCCCTTGGAAAAGAACCGGGGAGTGGGGTTTTGCACCCTTTCTTGCACGGCTTAATAAAAATAAAAATAAAA

AGGGTTGGGGGTTTATTAAAGGGGAGCCCTCCGGCCGCCTCTTTACAAGAAGTGAAGGATTGTTTTGGGGAAAGAGGTCG

AATAAGTAATATTTTTCCCTCTTCTTGAGGGGAGGTCGGTCTTTTCGTGAGGGCCTCAAGCAGAGGAAAGACGGGGAGGC

TTCATAGTCAAGAGGTTAAGACATAATGTTTTCACCATTATATGCGAGGGTTCGAGTCCCTCTGGGGCCTAGCTTTTCTT

GCCTGGGGAAGAAAGGCAACATAAGGGGAGGGTTTTAGAAAAAGCCGGCGGCTCCTAGGTGGGGCTAGCGGGGGCAGCCA

GCTGGCCTAGGGAGGGAGGGATTAGGCAGCAAGAAGTGGCAGTTACTAGCCAGGCAGCGCAGCAATTATCGAGCAGGGGA

TTAGGCTAGCCACCAAGGATAATCGATGATCGATCGGGGATCTCGGCTACGCCTAATCCTAGGCTGCTAGGGGATAAGGC

TTGGGGAGGAAGTGGGCCTTTCCCTTAAATAAGTGCGGCTAGGGCTCCCGCGCAGATCGGGGAGGTAGCCTTATCCAGGC

TCCTAGCTAGGCGAGCTAGGGGAAGGGGATTAGGCAGCCAAGTGGCAGCTACTAGCTAGGGCAGCAGCGCTTTGGTCGGC

GCTAGTAGCTTAGGGGATAAGTGGGGCTAGCTCCCTCTGCTGATCGGAGGCTTGGCTTGCTTGTCGGTATTTTTCCCCTC

TCAGCACTGAAAAAGAGGAACTAGCCTGCAGAGGGACCTCGCTTGCTCCAGCGCGTCAGGGGTTTTCCGACTTTTGGGGG

TTTGGGGGTTTTGCCTGGCAGCTTGGTAGGGGAGGAGCCAAAAGAGGGCAAAAGCCTAGGAAAAAGGCAAGAGGCAGTTA

ACAGGGAAAAAGACCTGCGGCGATAGGGGTGCGCAGGGGGTTAATTTAAACTAATATCTAGATTAGTTTTTCGTTCCTCA

AAACAGGAGGAATCGGGGAAGTTTAGAGGGACGGCACCGCAAACCCCAGTTGGCTGGGGGCGTCCCCAAAAGCCCGATAT

ATTCAGGGGGTTTATCTAAGGGGAAAACTAATGCTCTCCCGGGTTTAGGTGGGGAAAATTTAATAAAACTATACTTCCCT

CGACCCGGAGGGGGACCATATAAACTATTACTCCAAACTCTTTTTATATAAAATTTTCCCTCGGGGAGAATAGTAATGCA

AGGATATTTAAGCCATCCCTCTAAGGTGCGTCATTTAAGGGTCCTCAAGTATGGCCTCCCTCTTTCCCGTAGGGGAGGCC

TAGGATTGGGGATACCGGTTAGAAAAAGGAGAGGGAGCTAGCAGCAGCCCCTCTTATCCCCTCTAATGCTATCCCTCTAA

TCCTATCCCAGCGAATGGCGGCCTGTTTAATCGGCCTATCCCAGCCTAATCCCTGCCTAATCGAATCGGGGAATGGCCAG

GGGATAGGGATTAGAGGGATAGGGATAGGGATTAGAGGGATAGGGGATAGGGATTAGAGGGATTAAGGGGATAGGATTAT

GAGGGAGTAACCCTCTTCTTTGGGGGAGGATGCTCTTGTGTTTTAAGGGGAGCTTTACTGCTTAAAGCCAAAAAGACGAG

CACTTAGGGGGTTGCGATTGAAAAACTAATTTCCCTGCTACGCAAGCGGGGAGAATATTCTTTGCGGGTTGATGTAAAGT

AACATAATTGGCTCATGACCAATCTATTTAGGTGCAAGTCCTTTGTCCGCATTATTTTTCTTTAAACGTAGTGTGCGGCG

GCCATCTCTTGAGGTTATAGCTTAATTGGTAAAGCAAGCTGCTCATGACAGCTGAGTTGAGTGTTCAACTCACTCTAGCC

TTAGTAAGAAAAGTTCTTTTATCCGAGTGCTGAAATTGGTAGACAGGACGAGCTTAAGTTTCGTTGACGCTAGTCGTAGA

GGTTCAAGTCCTCTTTCGGATATATTTGTAATCCCATCTATAATATTATCCGCTTTCTATTATTCAGGGGTTTTAGATCT

TTTACTAGTTCCCTCCCGCGACGGGTAAGCCCTCGGGGAGGCCTAGGATCGAGGTTTTGGATATTTGTTGTAATATATCG

AAGATGGGGTGGAATCCGCGGATTTTTTTAGATAAACTAACCATCCTATTTAAGATCTTACCACCGTGCGTCGTGTATTT

GTACTTTTAAGTTAGCCCGTACCCCATTTTATAATTCCTCTTCTTTAGCCGACTGTCATTTTCATGGTTAGATATCTAAG

TTAGTCCCTAGGGGAACACGTTCCCTCCAAGGGACAAGTCCCTCTACGAGGGAACTAGCTTTTATAAAGAAAGACCTTTA

TTAATTAAAATCCATAATATTTTATTCCCTCCCTAAATAATTGGTTTCCTCCTCCTTTATGTTCTAGTTTTCGTTCCTCA

CGAACTCACCCGTGAGGGACAAAGCGGGGGTGGTTTCGTGGCCCACAAAACTAGTCCCTAGGTAACTTATTCCGCTCTCA

ATCGGGGATAATTGAGTCCCTCGGAGAGGCGCGCAACCTAGTTTTCGTTCCTCACGGGGAACTCCGTGAGGGACAAAGCG

GGTGGTGGTTTCGTGCCCCACAAAGTCCCCGATCTCATCCGGGGTGGTGATCGGGGGTATCCATCGGGGGATTAGGGAGG

GAACTTGGGGGTTCCTCTCCCTAATCCCTCTAATCCCCCTTTAGGGGTAAGATACCTCCTCCGGCGGGGGATGGGACGCC

CAGCAGCGAGCGGTCCCTCGTAGAGGGAAGGCGTCCCGCAGGAGGCGGAACATGGTTCCGGCTCGAGCCCGGAGGGACAT

GATGATGGTCAGCGGCTCGAGGGGAACCCAGCTTAAATAAACCTATCTAGGGCACTAGTTTCCCGGCCAGGGTTCCTCAA

AACAAGTAGGGTTTTGAGGGACGCAAACCAGTTCGCAGGGGGTCCCCCAAATTGTGGGCCACCGCAGCTAAAGTTAGGGG

GTCCCTCAAAACGGGTTTCGTGAGGAACGAAAACTCGCAGCTCCCTCCCTAGGGGTAGCCGAGCTCCTAGGGGAGGGAGG

CTAGCCTACGGGTGGCGTAAGGTGGGCTTCCCTTTCTTCCCTCCGGCTTGAAAAGCGGAAATCCTTTTTCAAGTAGGGCG

CCCGTATGGGGAGGTAGGGTGGGTAATCCAGTTTGCAAGGGGTTTAAGTTATCCCCGATCGATTAAGGGGATAAAGGATT

AGAGGGAGCTTCTCAAGGCTCCGGGCGCCAGCCGCGCTAAGGGGTTATCCTGCAGGGGTAAGGATGCAGCTAAGGATTGG

GGGATTAGGGGATACCCTGGGGAAAAAGGGGAGGAATTCGTGGCTAGGGGATATTGCCCCGGTATCCTAGGGGATAAGCC

AGGGGTAAATTAAAATAACTATTGTGGGCCACGAAACCCGTTTTGTCCCTCTGCGTCGTCCATCTCTCCTTCGGAGAGGG

GACGATGGATGATGGATGATGCAAACCATCGGGGTTTGAACAAGTGGATTTTCGGCTTTTTAACTTTTAGGAGTACCTCG

ACGATGAAAACCACTAGTCATCCCCGATCGAAGAACGAAGAATGATCGGGGATTGGGATAAGTCCCTCCCGAGGGACTAG

TGTTTTGCAATATGGGGAAAGGGGGTTTTGGTTTTCCCTCCTCAAAACAACAAGTTAAACCCCTTTGGATCGGGGGTTTG

GCATCCAGTGCTGTTCTCTCCTTCGGAGAGGGATGCAGAGGGACGAAAACTCGTTTTGCGGGGAAACCGCAGCAGCGGGT

TTTCCCTCTGGCGTCGTCCATCTCTCCTTCGGAGAGGGGACGATGGATGATGGATGATGCAAACCCTCGGGGGTTTGAAC

AAGTGGATTTTCGGCTTTTTGAACTTTTGAGGGAGCCCTCGACGATGAAAAACCACTAGTCCCTATCCCCTCGGGGATTT

CGGAAGCGGCGGAACTTGTTCCGGCTCTCAATGGGGACAAGTCCCTGAGAGGGAACTTCAGGTTTTCTGGGGGATGAACT

AGACCAGTATTTGCATGGCTATGCAGGGGAGGAATAAACCCAATCGGGTTTAGAAAAGGAAACACTTTTGCAGAGGAAAA

CTAGACTAGTCCCGCAGGAATCGGCGGCAACTTGTTCCGGCTCTCAATCGGGGATAATATCCCTGAGAGGGAACTAGGTG

GTTTTTCGTTCCTCAAAAGTTAAAACTTTGGGGTTTGGCATCCAGTGCTGTTCTCTCCTTCGGAGAGGGATGCAGAGGGA

CAAAACTCCCCAATGAATGGGGGTTCGTGCCCACAATTTGCGGGGGAACTAACTTTTGTGGTTTTCCCTCATCAAAACAA

CAAGTTAAACCCCTTTGGATCGGGGTTTGGCATCCAGTGCTGCTGTTCTCTCCTTCGGAGAGGGATGCAGCAGAGGGACG

AAAACCTAGTCCGGATCCGATCGATCGATTGAGGGGATTGGGATAAGTCCCTCGGGAGAATCGGGGGTACTTAGTTTTGC

GGGGGACAACGGAAAATCACTATTTTTTGTCCCTCATCAAACGAAAAAAGTTCGTTTAGGAACGAAAACCTAGTCCGGAT

CCCGCCGATCGATTGAGGGGATTGGGATAAGTCCCTCGGAGAGGGACTAGGAGTTTTTCCTCCTATGGCTAGAAAGGGGA

TCCCGATCCCTCTTTAGGGGTATCCCTCCTTTTCGTCTTTGCCCAACCTACGGCAGTAGCCCTGCGTAGGGGATAAGCCC

TGCCAGCAGCGCAGCAGCGCTTGCCGGGGGATAGCCGCAGAGGGCGAAAAAAAAAAAGGGGGTGGAGCCAGGAGGGATCA

GAGGGTCCTGCAGCTGCCGTAGGGGAGGGCCAAAAGGACGAAAAATAAGGTCATTTCTCGGGAGAATAGAGGGAGCAGGG

GGGTAGGGATACCGCAAGGCAAAAAGGAAGCTTATCCGCCGGGCGCCCTCTAATTCTCCCGCTTAAATCGGAGCAATGCG

GGGAAATATGGGGAAAAATGAGGGGGAGGATTTAGGGGGTTAATTAATTTCTCCGGGAAATATGGGGAAATGAGGGGGAG

GGATTTTAATTAATCGGAGTAATGGCACTTTAATGGGGTGGTCTTTAGCGCAATTTACCCCTCTAATCATCGGGCTCTTT

GCAAGGGGTATTGGTGTAATCCTAGGGGATAGGGGGTATCTCCTCCTAGGGGGAAAATCCGCTCTAATGCCGGCCATCCT

TCGGGGATGGGATTAGAGCGGATGAGGAACTAGGGATAACTCAAATATTTTCACTCTTTTATTAAAGAGATCTTTTTAAA

TCCCCATCTTTAGTCACGAAAAAGCTGCGCCATTAGCTTGCATAGAAGGGACAAGGGGAGTGGAGTGGAAATAAATCTTT

TTTATGAGTCTATCTTTCTCGTTTATAACTCATCTTTATAAAGTATCTTTTGCCAGATGAAAAGATATGGAAATATCTCA

TATACACTTTTTGCCTTTATAGTTTAAAGGTAGAACGGAATACTGTTAATATTCTCGTAGATGTTCAATTCATCTTAAAG

GCTTTAGAGAAGGGGGTTTTTCCTGAAAAAGGTATGTCATAGGGGTTTCGTTTTGATTGCGCCGCCTTAGGCATCCCGCC

TTAGGTATCCTATCCCCGATCTATGACGCGCCGTGCAGCAGCTAGGGCTCCATCGATCGGGGGATACTCCGGGGTAGGCA

AAGAGGGATGCTAAGGCGGCATAGGAGGAATAACTCTTTTCGTTTAGCAAACCAGTAGGGATGGGGTATTTTCCCTCCTA

CTATGAGCGAACTAAATAGGTAGGTTTAATTCCAGGCTTACTCGGGGGTAGGAAGTTAGGGGAAGCCTTACCTTTGGCAG

CGTATGGGGTGGTAGGTTAAGTTTACTCTATGGGGATCTTTATCTAATAAAATAAAAATTTTTCCACTAATCCTCACCCC

CACTAATGGTATCTCAAGAGTGGGGTTATGAAAAGGGGGACATAGTTTAACTGGTAAAACTACAATTTTGCATATTGTTA

TTTCAGGCTCGAGTCCTGATGTCTCCAAAATGGGGTTTCATTACCCTGCTATTGTATCTTTTATGATTATACACTAGCCG

GAGGGATCCCTTTCCTTTAAGAGCCTTGTCTAAGGTGGAGTTTTCGATAGCAGCGCTATCGATTGGGGAAGTGCCCTTAG

GGTACCTAGAGGGATTATTATTAGAGGGGATTAGGGGATAGACGAAGGAGTCATAGCGCTAAGGCCTTACTTGAGGGGGA

TAGCAGACTGCTTAAGAGAAGAGGTAAAAAGCCAGACGGGGGATAAGGGGAGGAAGCTCGGCTGTCTTTCCAGCAAAGCG

TTTAAAAGTGTAGTCCCTATCCCCGCAAGGCGGGGATTAGGAAGCAGCGGCAGCTGCAGCGGGAACTTGTTCCGCTCGGA

AAATTTCGCGGAGGGGACAGAAGTGCCACGTAGAGGGAACTGTTTTTCAAATTCAATTCAAATTGGGGACGAACTCTTTT

GTGGTTTTTCCCTCCTCAAAACAACAAGTTAAAACCCCTTTGGATCGGGGTTTGGCATCCAGTGCTGCTGTTCTCTCCTT

CGGAGAGGGATGCAGCAGAGGGACGAAAACTAGTTTTCGGTTCGGGGAAACTGGTTTTGCCCCTCTGCGTCCTCTCCGAA

GGAGAGGATGCAGGATGATGGATGATGGATGATGGATGATGCAAACCCTCGGGGTTTGAACAAGTGATTTGGGGGTTTTA

ACTTTGAGGGAGCCCTCGACGATGAAAACCACTAGTCCCTATCCCCTCGGGGATTTAGGAGGCGGTAACTTATTCCGCTC

TCTAGGGCTACGCTAATCGAGGAACTAGTGTTTTTTTCTTTCGATTGCGCGCTATCGCGCTTATCCCTATCCCTCTCTCT

CTCTCTCTTCATTTCCCAGGAAAGACCGAGGGGAGGGGTAAAGAGGGGGATTAGGTTGGGGGATAAGGCATAGTAATCCC

GCCTGCAGGGGAGGTCCCCTCCGGGCTCGAGCCCGGGAACCAGTAGCCTCCTGCGATCATCGGGGACTAGTAGGTAGAAA

AAGCTGGGGAATTGGGTGCCCTCTTATCCCTCTCCGGCTATCGGGTGTAATCCCTCTTTAGGGCCTATCCATCCAGGGGA

TTATAGGCGGATAGCTTTTCTTCCCTTGAAACACCTTTTAAAAAAAAAAAAATTTAAACCCAATTTTTAGACCCAATTTT

TAGAAACCAATTTTTTAGACCCTTTTAGAAAAAGCGGGGCACTGGCTTGGGGGAGGATTGATTGGGGGCTAGCTTGGGGA

GGAAGTCCCGGGGGTAGGGCTAAAGAGGGATTAGAGGGCAGCGGCGGTAGGGGTTGGGGATTCTTAGAGGTTTCCCTCCT

CAAAAGTTGGGGAAAAAGTATGTTGGGGTTGCCCTAGAAACCATAATAAAATTTCAGGGGAATTAATTTTGCCTAATCCC

GCAGCAGCAGGAGGCTACAGGTTCCGGGGGTCGGAGCCCGGAGGGGGACAAGAAGAAGGAAGGTCCCCTCCGGGCTCGAG

GGAACTAATGGTTATATTAGCCTTTAATAAATTTAAACTCATCAGAGCTTTTCCTAATGGTTATTATATTACATTAACTG

GTTAGCTCACTCTCCTATTCCCCGATGAATTGGGGAATCCTATCTTCGAAGAGGATACCATTCCTTATAGGGAATAATCC

CTTTGGGGATTATATATTATATATTATATATTATATATTATATATTTTATATTAGATATTCTCCCTCCCTAAAGGGGATT

ACAGGGGAGGAAAATTCTAAATTATATATTGCTAAATAACTACCTATTTAGGGGCAAGACGGGGGAATAAGGGAGATAAA

TTAGTTTAGAGTAGTCTAAATTTGTTTAGATAAACTTTTTGTCTAGCATGCTTGCTATGCGGCACTATCCTTTGGCATGC

GCTAGTTTTGGGGACTGACAGGTTTGCTGGGGGTCCCTCCCAGGGTTTCGTGAGGAACCCGGCGGCCAGGGGAGGGACTA

GTTTCCCCAGGCAGCGGGGGTTCCTCAAAACTAGTAAGGTAAGTAGTTTTTAGGGACGCAGCAAACCAGTTGGGGGTCCC

CAAAACTAGTCCTCAACTCCTTTTTCGAGGGTGAGGGTAGAAAAATCACCAATAAGATACATAGTATCCCTCAAATTTTA

GGGATAAAATTTTAGTAAGCCCCTCTTATCCCCTAAAGGGGATTACAGCGGAGGGAGCTTGTCCCCTACGGCTCCGAGCC

CGGAACCAGTAGCCTCCTGCGGGGGATCCCGCAGGAGGAAATTTTATCAGCTCGAGAAGCTCAATTGGTCGAGCGGGGTC

TTGAAGCGACCTAGGCTGTAAGTTCAAATCTTATCTCGAGCAAGAATACTAAGGTAATGCTGGAATTGGTAGACAGGAAT

AGCTTAGGCCTATTTGATTTTTAATCTTGTCCGTTCAATTCGGATTTACCTTATTTAGGATCGTTATCACTTGCTTACTA

ATCAGTCCTTTGTTGTCCTTACTACTTACTCCTTACTCCAACTACTCAGTGCTGAGTAACTACTACTTACTGCTTACTCC

AATAACTCAGTCCTTAGAGGAGAGGAGAAAAATATGTTGTAGACTAATCGGTATGTCATAAATTTTTGGTATTTAATATT

GGGTGTTCGAATCACTCCAACATAAAAGAGAAAGTAATCGGACCCTTTAGGAGTTTAGGGATTTAAAATAATCCCTCATT

TGCCCAATATATGGTGGATATAGTTCAATGGTAGAACAACTGTATGTGGCACAGTATGTTCCCTGTTCAAGTCAGGGTAT

ACACCCTTCTTTAGTTTTCGGCTAGCCAGGGTAGTTTAATTAGGTTAAAACATTAATTTCATGCATTAATAATGAGAATT

CAAATTTCTCTTCTGGTCAACTATCTATTGTTGCTATTTAGCATGTTGGCTTAATGGTAAAGCATTATATTACGGCTATA

ATCATAGGAGTTCAAGTCTCTTACATGTTTATAAAAACTTTCAGGGCCGGATTTTCTCCAGCAGCTAGGTCAGTAACTTG

TTCCGGCGGGCTCGAGGCGGAGGGACGCAGGGCATCCGGGCCTGCCCTTAGCCGCATGGGGATTACTATGCCTTATCCTA

ATCCCTCTAGCTACCTAATCCTACGCAGCTTATCCCGGCCTTAGCGGCGGGGGATTAGGCGTAGGGGGATAAGGTAGGAG

GTATCCTAGCTAGGCCTAATCCTAGGGCAGCCAAGGGGATAAGGGTGGCCGTAGCCTTACGGGGATTAGGGGATACAGCA

ATGAAAAAAATCCGGGGGCACTCTTAACTTTTTCTACCTACGGGCCAGCAGGGGATAAAAAGTCTAGGGGGTAAGCTCAA

GTAAGCAGGGGAAAGGGTCAGCAGAAAGGGGTCGAGCAGGGGAAAGGCAGCCAGGAGGGAAAGGGTCAGTAGGGAAAGGG

TCAGGAGGGCTCCCCTGCCTGCAGCTTAGACGACTCCCAAGTATGCAGCAGGGAATGGAAAAAGACCAGAGCCTTAGACA

AGAGGGGAGACTAAAGACTCTTCGTCTATGCCTTATCCCTGCAGCTAATCCCTCGGGGGTATCCCGCCTTAGAATTGGGG

GTAGGAGGAGGGCGTCATAGGGGAGGGTAGGCCCTCCTCCGGCGGGGATACCGCAAGGCAAAAAGGGAAGCAGCTTCTCG

GGGGCTCTTCCCCGCTTAAATCGGAGCAATGCGGGGAAATATGGGGAAAATTAGGGGAGGGATTTTTAATTCATCGATCG

GGAGTAATCGGTCTTTCCTTCCCCTCTAATGGGGGCTCTTAGCAAGGGGTGTAATCCTAGCAATTGGGGGATAGGATACC

CTTAGAAAAAGGAGAGGGAGCCGCCAGCTGAGTGGTTTTTTCCCGGAAGAAAAAGGCTGTCCCCTCCCTCGCATGTAAGC

TGAGTGGTTTCTCCTGCTATGGGTTTAAAAGAGGGGACTAGCTAGTTAATATTTCCACCACAAGTCCCGATCCCGCCGGA

GGAGGTATCCCGCTTCGCGCGCCAAGGCAGGGGATTAGACTAGTAGGGGATTAGGGAGGGGAACCTTGCCTCAGCCTAAT

GGGCCTCGGGCGGCTACGCTAATCCCGCCTTAGGGCGGGTATCGATGAAGGGGATACCCATTGATTGGTGTCATCGGAGG

GATTAGGCGGCTAAGGGGATCCGGGAGCCGCGTCGTAGCGGGGAATCGCAAAAAGAGGGAGATAAAGAGTGATCCCTAAA

TTCCTCTGCTTACCTACTAACTATTATGAGTATAGATTAATAAATATTAATTTATTAACCCCTTCCCTCCCCTCAGAGCC

GGGGGTAACCCTTTTTTTAGTTTTCGTTCCTCGGGCCAGCACTTTTAGGGACGGAGCGCGGCCAGTGCAGGGCGTCTCAC

AAAACTCGTTTTTCGTTCCTCAAACTTGGGGGTTTGAGGGACCCAAAACTCTCGATTGGGGTTCGTGGGGGCCACAATTT

TGCGTTCCTCAAACTTGGGGGTTTGAGGGACCAAAACCCAATCAATTCGGGGGTTCGTGGCCCACAAAACTAGTTGGGGA

AAGGCTCTCCTCTTAGGTATTAACTTAGGCTCTCTAAGCTGCTATAGTTAGGGGAAAACGTTCACTAAGTGGGCTCCAAT

AACTCTTCTTAATCCCTATCCCCGCCTTAGCGCAGGGGATTAGAGGCAGGGAACTAGTTCCGCTCGAGCCGTAGGGGACA

ATAAGTCCCTCGTAGAGGGAACTAGGTGGTTTTTCGTTCCTCAAAAGTTAAAACTTTGGGGTTTGGCATCCAGTGCTGTT

CTCTCCTTCGGAGAGGGATGCAGAGGGACAAAAAGAGTTCGTTGTCCCACAAAACTAGTTTTGGGGGACGAAACCCGTTT

TCCCTCAAAACCAGAAAGAACCAGTTCGTTGAGGAACGAAAACTAGTCCAATCGGGGGACTTCATGTTCCACCTCGGGGA

CCAGTTGCAGGCCTCAGGAACGAGTTTTTAAAACTTCTGGTTTTGAAAACAAACCAGTTGGTGTTTCCTGCAAACTAGTC

CCAATCCCGCGCCAGGGGATACGGAAGCGGCAACTTGTTCTCCAAGGGGACAATAATTGGAGTCCCTCGGAGAACTAGGG

GTTTGGCTTTTTAACCCCCAACCACTGCCCTCACCACCATCTAATATTAGGGTTATGCTGTAGATGGTTCTACGTTTTGA

TTGCAGATCTTAAATAAGAAGTTCGATTCTTCCATATCTCTTTAGTTCCCTCTACGATCATCGATCCGATCGGGGGACCA

ATTGGAGTGAGTGCCTTGGAGAACTAGTTCCAGGGGGTAATCCCGCCTAGGGGATAAGGCACTAGTAATCCCGCCCAGGG

GATGCGGCAGGGCCGTCCCCATCGGGGGTATCCCGCCGCCTAAGAGGAGCGAGCGCCTAAAGGCAGGGGATTTCTCGGGG

ATTAGGCAGAGCCGGGGAACCCTGCGCCTCCTAATCCCGATGGGGATAGGGACTAGTAAGCCCAGGCCCTCCCTCAAAAC

TTTGAGGGACGCACCGCAACCGGGGTCCGTCCCCAAAACTCGTTTTGCTTACCTCGGCGAGCACAGGTGAGGGACGCACC

GCAAGCGTGCAGGGGTCCCACAAAACCTCCAATCCAATAGTAGTCCCAATCCCAATCCCCTAAAAATCGGGATTGGGATA

AGTCCCTCCCGGGGTGGGAGGGACTAGTGTTTTGCAAATGGGGGCACGAAACCCGTTTTTGCCCTCTGGCGTCGTCCATC

TCTCCTTCGGAGAGGGACGATGGATGATGGATGATGCAAACCATCGGGGGTTTGAACAATAAGTTTTAGGAACGGCCAGA

CTAGTTTTGCGGGGAACTCCTCTCCTGTTGTTTTGGCCCCTCAAAACAACAGGAGTTAAAACTTTGGGGGTTTGGCATCC

AGTGCTGTTCTCTCCTTCGGAGAGGGATGCAGAGGAACGCAAACTAGTCCCTTCCCTTCCCTTCCCAATCCCGCCGTTCG

ATGATCTCAGGGGATTGGGGAAGGGAGGGAAGGGATGTCCCTCCGGAAATCGGGGACTTGTGTTTTGGCTAATGGGAAAA

CTTCGGGTTTTCCCCTCTGGCATCGTCCATCTCTCCTTCGGAGAGGGGACGATAAACCTTTGGGGTTTGAACAAGAAGAA

AAGTTCGTTGAGGGAGCCACTAAATGAAAAACCACTAGTCCCAATCCCGATTGAGATCGATGGGGATTGGTGGTATAAGT

CCCTCGGAGAATCGGGGGACTAGTTTTGGGGGACGCAGGTTTTGCTGGCGTCCCTCAAAACCCTAAGTTTTGAGGAGGAA

GGCGCCTTGCCAGGGGAAACTTGTTCCCCTCTTATCCCCTAAAGGGGATTAAGGAGGCCAGCGTGCCCGGCTCCGGGGGT

CGAGCGGAACCTTGGGGGTCCCCTGCGGGGGATAGGGACTAAGAGGGACCCCTTGGAGAGGGAGGTAGTGCCTCTACCCC

TAATCCGCGGCAGCGGGGGATGCTAAGGCGGCGGGATAAGAGGGCGGCTAGCACCCTCTTCTTAGAGGGTGCCAAGCTAG

GGGGTAATCCGTTAAGGCTGCTTGCAGCGTAGGGGATAAGCAGCCCCTAGGGCGATTCGGGGGTAGTTTGGGGGATGGCA

TCTCACGTTCTCTTTTAGCCTGGGGATCCCTAAAAATCCCTCCCTCCTCCTTTTTAGGTACCGTAGGGGAAGGAGCTAGG

GGAGGGAGTCTAAGAGGAGGCTCCTCCAAGTATGCAGCAGCAGGGTTAAAGGGGAGCTCAAAGCTTTTGTTTTCTAGCTA

TGCCAGGTCCCTAATCCCCTCTTTAGCGCGTAGCCTGTATCGAAGGGGGATAAAGGATTAGAGGGCTGCGTAGGGGGATA

GCGCTTTGCTTAATTTCCCTCGGGGAAGAATGGAATGGGGGATACTAAGGGGGTTTTTCTTATTAGCTTAATGGTAGAGC

AGGATACTTCTAATATCTGGGTCTAAGTTCAAGTCTTAGATAAGAGTATTTCTTTTTCAGGGCGCGAGGCGCCATAAATA

GAAATATATCTATTTCTTTTAATATTTTAATTTATAATAATAAATATCCCAGGGGATTCCGGGGAATCTTCAAAATACTC

TCGCTTGCGCAACCATAGGCTTGCGCAGGCTGCGAGTAGTCAAGGTCTCTCCTCATATCCCCATCATGGGAGGCAAAACC

ACCTACTTGAAGGGGGTGTTTAACCTCGTAGCAGGGTGCCGGAGGGTGTTTTAAAGAGGGGAATCCCTTAGAGGAGCAAA

GTTCTCTCCTAGTTTTCGTTCCTCCCGAGCACTTTGAGGGACGCACCGCGCGCCAGTGGGGGTCCCGCAAAACTAGTTTT

TCAAAACTTCTTCTGGTTTTGAAAACAAACAAGTTGGTGTTTCCTGCAAACTAGTCCCTCCCTTCCCAATCGCTAGATCG

ATTATTTTCGATTTATCGGGGATTGGCAAGGGGAAGGGATAAGTCCCTCCGGGAGGGACTAGTTTTTCAAAACTTCTGGT

TTTGAAAACAAACAAGTTTGTTTCCTGCAAACTAGTCATGAGTGGGGACAAAAGTTTTGTCCCTCTTGGACTAGCTGGCG

CTAAATACTTTAGTTTTTCTAAGCACCTCCCTGTTTTAAGTGGCGTTTTTAGGGAGGTTTGTCCACAAAACTAGGGGTCC

TCTCCTAATCCCTCTTTACCGTTTTTCCTCTTATGGCGCCGGGGGCCTTTAGATAAAAAGGGGAGGAAGTCCGCCAAGGG

GTAGGCTCCCAGGCGGATCTTACCGGCGCCGCCAGGCTTATCCCTAGCGGCGGCCGATGGGGGATTACGGGGAGGAGGGT

AATCCCGGGATAAGAAGAAGCAGCTCCCTGCGCAGGAGCTAGCTTATCCTAGGGGAGGGATTAGGGGAGGAGGGGTAATC

GCCGCCTTAGCAGGGGATAAGTGGCTGCGGCTAGCTCCCTCCTGCAGGGAGCTCACCACCCCAGGAGGGGAGGAAGTCCC

AGGGTAGGCTTTCTTCCCTCCCGCTGAAAGGGGGAACGATTTAGAAAAAGGGGAGGCTCCCCGCTACGCAGGGGAGGAGG

GAGCTCCCTAGGGGAGGGGTAAGGTTGAAACCTGCGGTAAGCCGCCAAGTTTTAAAAACTTGCTCTTTTAGGGGAAATAC

GCCAGAGGGGATGGCTAAAGAGGGAGGGATTAGGGGGCTAGTTTGCAGCATAAACTCCTTTTTGGGGGTTTTAAGGAATC

CCCAAAAAGCAGCCGATCGATAAAGGGGGAAAGTCGGTTTCCTCCCCTGCTTTTCAAGGAGTCGTCTAAGGAGCGGAAAG

CGTCCGCGCCGGGAGGGCCGGCAGCTTTTCAAGGAGTCTAAGGAGCGGTCCCAGGTAAGGAGCGGTAAGCGTAAGCAGGG

GAGATACTAGATAAGGGGAGCTCCTAGTATGCCTGCTACGCCAAATGTGTATTCCCTCGCTGGGGGTATCCTCCTCCTTT

TCAAGGGTGGGAGGGGGAAAAGACATCCCTCAGGTCATTTCCCAGGGGAGAATAGAGGGGATAGGGATGCCTAAGGCGGG

GATAGCGCTTTCCTTAACCAGGGGTACGCTAAAATCGTGGGGGATAGGAGACTCCGGAAAAGAAGGGGAGAAGGAGACAA

GAGGTATGAGTCGGTCGGTCTGACTAGGGGAGGCATAGCGGCGCAGACCAGGGGGAAAACTCGCCGCAAGATATGAGGAG

GAATCCTCTAAAAATAAGTATGGAATTAGGGTATTTTTAGTTTAAAAATTTAACACAATAATAATAAATTTCATTTTTAT

TTTATTTTATTTTATCTTTTATAAAAAAAATTTTTTATGATATATCTTCCAACTTTAATGTCTATAATAGAAGTATTATT

AGTAACTATACCTGTATTATTAACGGTAGCCTATGTAACTGTAGCGGAAAGAAAACCATGGCTAGTATGCAACGAAGATT

AGGTCCAAATATCGTAGGTCAGCTTAAATTTCAGACCTCTTTTAAAAGGTCATATCATGTCTCAAATAATAATAATATAA

TAAGCGAATTATATCTGAACGGCCCTGGGAAAGCTTTAAGCCGGCCTTATCCAGGAGAAATTGTAAGTGTTTGTAAAGAT

TTACTATCTTCAACTGCTCTTAATACATTCTTTAAAGGTTTAAAAGGTAAAGGTGGTGGTATCTATATCTTTACTTATAA

AAGTGATCAGGCTATTTATTACATAGGTAGAGCTAAGGATTTCCAAAACCCTCCCGATTTAAAGCTCACCTGAATATTAA

TTTAAAAGATAGATTCCATGTATTTGCTAACGCAGTAGGTTGAGATAAATTATAATTATCAGTTATTTAGTTTTGTAACT

TAGATATGCAGCGTGAAAGAGAGAACTATTATTTACAAAAATCTTTCCCGTTATTAAAGACTAGTTTTGTGGGACAACGG

TAACTTAACCTTTTTTTGTTGTCCCTCAAAACAAAAAAATCTAAAAATCCAAAATCTCTCGATTTGGGATTTTTAATTTT

GCAAAAATATCTTTTGCAAAATATAAAGAAAGAAAAATAAAAAAAAAAATTTTTTTTATTTTTGCTTTTAATTTTGCCTG

CTTTCGCAGCCAAAATATAAATCTAAAAGAAATCAGAAGAAAAATAAATTTTTTTCTTTTAATTTTTCAAATAAATTCTT

ATTTATTTTAAAATCTCTTTTTATCCCTGGCCTCCACCCCTAGGGGGTGAGGGAATAAAATAGGGATGGGGAGTTTTGAG

GGAGCCACCTCGGCGATTAAAACCACTAGTCCCCATCCCGCGCGCAGGGGAGGGATACGGGAGGCGTAACTTGTTCCCCG

CCTTTGGGGAGGGTTTTTCCTGTAAAACCCTCCGGGGCTGGGCTCCCGATGATCGAAGGGTTTTTAATTTTGGCTCCGCC

AAAATTAAAAACTCGCCTTTGGGGAGGGTTAAAAACCCTACGGGCTCCCCGATGATCGAAGGGGTTTTAATTAGGGCTCC

GCCAAAATTCACAGGAAAACTCCCCATTCGAAGGGGAGAGGGAAAACCCTAGGGCTCCTGATGATCGAAGGGGGTTTTTA

ATTTTTCTCTGCCAAAATTCACAGGAAAACTCCCCATTCTTCGAAGGGGATAGGGGTTTCTTCCTGTGTATTTAAACCCT

CTGGGGGTGGGCTCCCTGCCGAAGGGGGTTTTAAAATTTTCTCTGCCAAAATTCCAAAACTCCGAAGGGGAGAGGGTTTA

AAAAACCCTACAGGGCTCCCCGATCGAAGGGGAGAGGGAAACCCTACGGGGCTCCCCGATCGAAGGGGAGAGGGTTTAAA

AACCCTCCTCCCCTTTCTTTTAATTAAAAAATTTTCTTAAAATCCAAGTTATTTTGGCGAAGCTCAAATTAAAACCCTCC

CTTCCCTTCCCGAAGGGGAGGGTTTTTCTAAAACCCTCCCTTCCCTTCCCCGAAGGGAAGGGAGGGTTTAGAAAAGGGGA

TAATCATCGGGGACCCTGAGAGGCAACTATTTTAAAAGTAATTTAAGTGATACCCCAACTTTTATTCCCTTTATTAAATA

TTAAAACTTAGACAATTAGAAGAAGAGTCTGGTTTCGATAATAAGTATAAAGGTATATTTATTCATCTTTATGAATATAT

TAATGGACAAGTTAGTACTAAGTATACTACATTTCGCAGTATAAATCCGCTATCTAAACATTCAAGTATAAGTCGATAAA

CTATAAGTATATATTTAAATACATATGTACCATTTAGAGGTTATTTCTTTTAACTGATGTGATAGAGTATCCTTAAATGA

TCGCAAAATTAGTTAGTGATGCTACCAAGGGGTTAGATTTAGACCGTACTATAGCTAAAAAGTCTTAATGTATTATGTAA

AAGCAGATGGTACTGTAGTTAAAACTATATATGAGTCAAAAGGTGCAGTAGGCTAAAGCATTAGATGTACGTCATACTGT

TATAAATATTCATCTCGACAAATGAATTAAGGGTGGTGGTATTAAAGATAATTATCTATTTAGTAGTCTTTGGATAGTTT

AGAGTTAGACAAATTAATGGAAATATCTTTATTAAGAAAACATAATAATTTAAAAGTATGAGTTTAGGATGCTTTAACTT

TAGAATTAATATCAGAGATGTTTGGTAGTTCCCTCTCAGGATCTTATCCCGGGCCCTTTTTAATTAAAAAAGGGGATGAG

AGCGGAATAAGTTGCGCCGGGCGGCGTATTGCCGGGCCTTCCTTCGCGCGGGAGCCGGACTAGGTTTTCGTTCCTCTGCA

TCCCTTCCTCTCCTTTGGAGAGGAAGGATGCAACCCCATAGGGGTTTTAACTCTTGTTGTTTTAGGGACCAAAACGGGGG

TTTCGTGTCCTCAAAACTCGTCCCTAGGGGAACTTGTTCCGCCGCCGGCTCTCCAGGGACTCCACCAATCATCATCGGAT

CGATTGGTGATTCTCGGAGATGATTGGTGGATCCCTCGAGAAACTAGTTTTGCGTTCCTCTGGCATCCCTTCCTCTCCTT

TGGAGAGGAAGGATGCAACCCCAAAGGATAGGGTTTTAACTCTTGTGGTTTTAGGGACAAAAACGGGTTTCGTGGCCTAA

AACTAGTCCCTCCTGGAGGGACTTATCCCTTCCTCCTTCCAATCCCAATCGGATCGGGGATTGGGAAGGGAAGGGAAGGG

ACTAGTTAAATAATAGTTTTGATGGAGAGTAAGAAGTAAGTGCTCTTATTAGGCCTCAATTAGAGTTATATACTTTAATT

AAAACCATCAAATTGACAGGAAAGCCTTAAAGCTATTTCGACTAAGTAAGTTAAGTAATTAGCTTATGGCTCGGTTAACA

ACTAGAGGTATAGTGAAACAGAAATAGATGCACGAAAGTAAATAGGTAATCTGCAGCCAAGTGCCTAGTTCGCTAACTTT

AAGATTATTCTAAATGATTAAAGGGGGCTCTTTTCTTTATTCGTAAAAAAATCCCAGCGAAGTAATTAGGTATGCAGTTC

ATCGACTAAACGGTGGTTTATATTAATTCTTAATAAATATTAATGATTAATATTTAAGATATAGTCAGACCCAACTTGAA

AGAGTACAGGTATATTATTCTTATAAAGATACAATAAAAATCTTTTGTAAGGAAATAACCGTTAAATGGATAATATTTCT

TATTATTTAGGGAGAAGGCCCCTAACTTTACCTGTATTATAGGAAACTTTTCCTATAAATGGTTAAAAAGGGGAGATTTG

TATTACGGACTTCTTCAAGCATTTGCTGATGCCTTAAAACTTCTTTAAAAGAATATGTTTCTCCTACACAAGCGAACTTA

GGTTTATTTTTAGTGGTCCAATTATTACTCTAATATTTTCATTATTAGGTTATTTAGTTGTACCTTAGGGATTATAGGGT

AATATAGTCAAACTCCGGGGAGGTCCTAAAGCTTATTTTACCAAGCTTTAATCGAAAGGTTAAAAGTGGATGAATTAATT

ACTCATGTAATGTAACAAGAAATAAGATACTCTTTAGAGTAATGGATGATCGCGGATCTAAGTCAGCAATATCTCCGGGT

ATTGCAGTAAAAGAGCAACGAGTAAACGGCTATAAATATTTACCCTCTCCATAATGGGGAATATATTTAAGATGTATTCT

AATGAGCTTAGAAATAAGCTATGGAAAAACTTTTATACTAAAAATTTCTACGGATTAAGCGTTAAATGGTTCTTCTAATT

CTTTAAATGTTAATCCCTCGTCGTTATTATGTTACATTAAGTAATAATAAACATAAGATTTACGATTTACATATTGGTCC

TTTATTTATCACCCTTTGGGGTTTAGTGATGCCGCCGCCCTTGGATTGGGATTGGGATTGGGATTGGATTGGATTGGATT

GGTGGTTTTCTGGTAGTCCCTATCCCTCTGGGGATTAAGGGGAGGAACTTGTTCGGCTCCCGTCAATCGGGGATAAGATC

CCTGAGAGGGAACTTGTGGTTTTGTGGCTCCCTCACGAAACCCGTTCAAACCCCAGGGGTTTGCAGCATCCTCTCCTTAG

GAGAGGGATGCCAGAGGGACAAAAGAGTTGTTGTCCTCAAACTCGTGGTTTTCGTACCGCCTCCAAACTTTTTAGGGACA

AACGGGTGTCGTTGTCCCACAAAACTAGTCCCTCCTGGAGGGACTTATCCCTTCCTCCTTCCCCAATCCCAATCGGAGAT

CATCGATCGATGGGGATTGGGAAGGGAAGGGAAGGGACTAGTGGTTTTCATCGTCGAGGTGGGCTCCCTCAAAAATTTTT

TAGGGACAAACGGGGTGTCGTTGTCCCACAAAACTAGTCCCGATCCCTCCGGGACTTATCCTTTCCTCCCTTCCTTCCTT

CCCCAATCCAATCGGATCGATCGGAGGGGATTGGGAAGGAAGGGAAGGGAAGGGACTAGTGTTATAAAAGTTCCTCTTAT

AAATTAGGGTTTACTTGTTCAATTAAATTTAAAAATTAAACTACATGTTAGGAGATTTTTCAATTTTATTAGGAAAACCA

CATCCCTTTAGCTCGTTTAGGGGAATAGGGGTATGGTATTCCCCGGCCAGCAGGGGAACTAAAAAGGGATTAGACGGGGC

TCCCTGGGGGTTTTTAATCCTTTCCTTTAAGTAGTAACAATCTTCTTGTGCCTTTAGAGTAAGAAAACTAAATGAGCTAA

TAGAATTAGTCCAATCCCGCGCAGCAGCAGGCGCGGGATACGGGAAGCGGAACTTGTTCCGCTCGCTCTCAATTGGGGAC

AAGTCCCTGAGAGGGAACTAGTCCTCAATTGGGGCCCCTCTGGGGATTCAGGAGGCGGCGTAACTTCTGGTTCCGGGAGG

GGCTCTCCAAGGGGATAAGTCCCTCCCGGAGAGGGAACTAGGTTTTTCGTTCCTCAAAAGTTTTGAGGGACCAAAACCCT

ATCATCATCGGGGTTCGTGGCCCACAAAACTCGTTTTGCGGGGAACCGAGGGGTGGTGGTTTTTCCCTCATCAAAACAAC

AAGTTAAAACTTGGGGGTTTGGCATCCAGTGCTGTTCTCTCCTTCGGAGAGGGATGCAGCAGAGGGACGAAAAACTAGTC

CAATCGGGGGACGAAAATCATGTTCCACCTCGGGGACCAGTTCCTGGGGCCCCTCAGGAACTAGTTTTTTCAAAACTTGT

TTTGAAAACAAACAAGTTTGTTTCCTGCAAACTAGTCCTAGGGGGAACATGTTCCGCATTGGTCTCCATTGATTATTGGG

GAGAACATTGGGGGACAAGGTCCCGAACGATCCGCAGAGGTAACTAGTTAAATTTTTTTATAAATATCCTTTAATTTCTA

AAAAAAGGGGATTCTTTATTCTTTAAGCAAATGGTGTGTCTATTATGCAATTGAAAGAACACATTCTCTAAATCTAAACT

TTAGAAGGGGTTTACAAAAGATTGTAAACATAAGAGGAACCTTAAAAATTTTTTTATCAAAAGAATTACAGAAGAAGTAG

TTTTAACTATGTTGCCTTAAACTCTGCAATTTTGGCCGTCCGTCCGTCCTTTAAGAGAAACCGTGTAATCCCACATTCAG

ATTGAATAGGCGCAGGATTTACAAGTGGGGAGGGAGGCTAATTTCTCTGTTTCCTTAGACAAAGGTATCTTAAAATCTCT

ATTGTTTAAAATTACTCAGCATGAAAAGATGAGGTATTGTTAACTGCAATTAAAGAATATTTTCCCTCATTGTGGATATT

GTTATTTAAGAAAACCCTAAAATACAATAGATTTTTGCCCTCAAGTTAGGGGACCAAATTTTCTTATGTTAATTAAATAA

TTATACCTTTCTTTATTTATAGTCCGATATTAGGTGTTAAATCTTTATATTTAAAAGATTGATGTTTAGTTCCCTCTCCG

GGAGGGACAATAATTGGTGGGGAGTCCCTTGGAGAGCCCTCCCGTATCCCGCGGCAGCAGCGAGCAGCGAGCCGCCGCAG

CAGCGAGCCGCAGCGAGCAGCGAGCCGCGAGCGCGAGCGCGAGCAGCGAGCAGCGAGCCAGCAGCGAGCGCCGCAGCGAG

CGAGCGGAGCGAGCGAGCCGCGGCGGGATTGGGAACAAGTTCGCTTCCTAATCCAGGGGTATAGGGACTAGGTTTTCGTT

CCTCAACGAACTTTTTGAGGGACAAGAAGAAAACCGTTTGTCCCACAAAACTAGTTCCCTCGAGGGGGACTTATCCCTTG

AGAGGCAATAAGTTCCCCTCCAGCGGGGAGGACTAGGTTTTGCGTTCCTCAAAAAATCTAAAAATAACACTAAAATAATT

TTTTATTTTTAATTTTGGCAAAAATATCTTTTGCAAAAATATAAAGAAAGCCAAATAAAAAAAATTTTTTTATTTTTGGG

GCTTTTAATTTTGGCTGCTTTCGCAGCCAAAATATCAAATCTAAAAGAAAGAAGAAAAGAAAAATTTTTTTTTTAATTTT

TCAAATAAATTCTTATTTCTTTGAAAAATTATTTTTTATCCCGGGCCTCACCCCTAGGGGTGAGGGAATAAAATAGGGAT

CCGGCACTTTGAGGGACAAAACGGGGGTTTTCGTGTCCCCAATTTGCAAAACACTAGGTTCCTCTCCGTGGCACTTCTTG

TCCCCTGCGGCTCGGAGCGGAACAAGTTCGGCGGCAGCTTCCTAATCCCTGCGGGGGATAGGGACTAGGTTCCTCTCCGG

GAGGGACTTTTGTCCCTTGGAGAGCCCTCCGGCGGTAACAAGTTCGCCGGCAGCTTCCTAATCCCGCCGATTGGGGGATA

GGGACTAGGTTCCTCTCCGGGAGGGACAATAATTGGTGGAGTCCCTTGGAGAGCCCTCCCGGCGGGAACAAGTTCGCTTC

GGATCCGCGCCAGCAGCAATCGGGGATTTACTAGTGATTTTCCGTTCGTTCGTTCGTTCGTTCGTTCGTTCGTTGTGGTT

CGTTCGTTCGTTGTGGGGGTTCGTTCGTTCGTTCGTTGTGGGGGTTCGTTCGTTCGTTCGTTCGTTCGTTCGTTCGTTCG

TTGTGGGGGTTCGTTCGTTCGTTCGTTCGTTCGTTCGTTCCTCAACTAACCATAAGAAGTAGTAGTTTTGAGGGACAAAA

CCCGCGGGGCCGGCCGCCGTTTGTCCCCACAAAACTAGTTTCGGGAAATGGCAAAAGGGGCAACCTAAATTAGAAGGTGC

TATTAAAATAAGAGAGATTAAAAGGGGAATGAATAGGGGAGAAGTAAGAAAATTTATACCGTGTATTTAAGGATAAAGTT

TTTTTGTATGGTCCTGGTTTAGCTTTATCAGATATAAATATAGGTATATTATATATGTTAGCGGTTTCTTCTTTATCAAC

ATATGGTATACTATTAGCCGGATGAAGTGCTAATAGCAAATATGCTTTTAGGGTCAAAGTGGCCCAATAAGGATGTAAAT

CCTTTTTAAAACGAACTATAAGCGAAAACCCTTAAAAAATTCTTTTACTTTATTAGGTACTTTATCCTGTTTATTGAATT

TTTCCCCGCCCTTTTATTATTAAATATTGAATTATTTCAAGTACTAGTTTTGTGGGACAAAACTTCTGCAGCGGGTTTTG

TCCCTCAAAACCAGTTCAAACCCCAAAGGATCGGGGTTTGGCCTCTCCCTTCGGAGAGGGATGCAGAGGAACGAAAACTA

GCCCCTTTTTTCAAAATAGCTCCCTCTTTTATGCAAAAATAGGCTTAAAGCTAAGGGAGGCCCTTATTGAAAAGACGGGA

AGTTTCGGTTTAAATCAAATTAAAGTAATCGTTGGTATGAATAAAGGTAGAAAGTGAGTATATTCTTCTGGTTTTAATAA

TAAAACACAAAAATAGGATTCCATACTAACACTACATCTATTGGTTGTGACACAACAATTAATCTACCTTTAATATTAAA

TAAACAGCTACGTGTAACACCATATATAAGGGGCTCTAAAGTAAAACCCTCTTAAAGAAGGGTAATTCGCAAGTAACCAA

AGCGTTTAACTCGTTAGTAGGAACTTCAGAGGCCATACGTTTGTTATCTATAAAAAATCTGGAAATTACAGATCTTATAT

AAAAATAAATCATCCAGAAAAACCTCAAGGCAAAAGTAAATCATGAAATCAATGATTAGCTGGTTTAATCGACGGTGATG

GTAGTTTTATTTAACTAAAAAGGTTATGCTAGTTTAGAAATTACTATGGATATAAGAGACGAACATGCATTACATATAAT

TAAAATGTTTATGGTGGATCTGTAAAACTTGTATCAGGTGCAAAAGCATTAAGATATTGTTTACGGCACAAAGAGGGATT

CCTAGCTTTAGTTAAAGATGTCAACGGAGAAATAAGAAATTCTTATAGATTAATACAATTAAATAAAATATGTTTAAAAT

ATGAGATTTATTTAATTTATCCGTCAAAGTTAACATACGATAATGGGTGATTATCTGGTTTTGATGCAGATGGTTCTGTA

ACTTTAAATAAATCTAATGGGGACTAGCTATAACATTAACTCAAAAACTAGTGAAATTCTACAACCTTTGATAGATATAT

ATGGTGGTTCTATTTACATAGACAGAACATCTAATAGTTTTAAATGATATATTTCTAATAAAGAGTCTATTACTAATACT

ATTGAATACTTTAAAAATATCCGGCAAGGTCTTTAAAGAAAAACAGATTGCACCTAATACCTAGATGTTATGAGCTAAAA

GAAATAGCAGCTCATAAAGCTTCATCTGTTACAAGTCCTTTATTAGCTAAAAGTTGAGAACTTTTTAAAAATAAATGAGA

TAAATATGAAATTTAAATTAATCCATTTATAATTATAGATAAAGATATGGTCCAAATTATATATAAATATATGTTTATGT

ATATTGCACTAAGAAGCACCGCACAATTAATAAGTTATGAATTAATCTTAAGTTCTGCTATATTACTTGTTGTTATGTTA

ACAGGTAGTTTAAATCTTACTGTTAATATAGAAGCTCAAAGAGCTATATGATTTATAGTGCCTTTATTACCTATATTTAT

AATCTTTTTATAGGATCTATAGCGGAAACCAACAGAGCTCCTTTTGATTTGGCAGAGGCTGAGTCTGAGCTTGTTAGTGG

TTTTATGACAGAACACGCGGCAGTAATTTTGTTTTTTCTTTTTAGCGGAATATGCTAGTATTGTTTTAATTTGTATATTA

ACAAGTATATTATTTTTAGGAGGTTATTTATACAATTATATCTCTTTATTTTGATTAACTCAATATTTTGATATAGATTA

TTATATCGATAATTATAATCATCAAAGAATAATAAAAGATCCTTTATTAGAAGGATTAGTATATGGGTTTACTTTAGGTC

TAAAATCTTGTATTATGATTTTTGTTTTATCTGGGCTAGAGCATCTTTATTTCGTATAAAATTATACCTGTTAATGTCCA

TATGTTGAACTGTTTTATTACCTTTACTTTTGGTTTTATTATCTTAATACCTTGTTATTTATCTTCTTATATAAATACTG

TCTGTCAGTTTTATTTAATGACAAGCAAGGCAAGCAGGGCAAGCAGGGGCAAGCCAGGGGCAAGCAGGGCAAGCAGGGCA

AGCCAGGGGCAAGCCAAGGCAAGGCGCAAGCCAGGGGCCCAGGGGCCAGGGGGCAAGCAGGGCAAGCAGGGCAAGCCAGG

GGAAGCAAGGCAAGCAAGGCAAGGGCAAGGCCAGGGAAGCCAGGGGAAGCCAGGGGCCAAGCCAGGGGAAGCCAGGGGCC

AAGCAAGGGAAACAAGGCAAGCAAGCAAGAAGCCAGCAGCAGCCTTTCCCATTTTAGTATTACAAGTTAATAAGAATGAT

GTTAATCTTAACACTACCTTGCCTATAAAGAGGAAGGTTCTTTAATTACTATTCCTGTAAGAAAACATTATACTAATATT

TTTAAAAGTCTACTTTATCTTGCATAAAATAAGATCTAAAGTTAGTGAGTGGTATTTATGCCGCCTTTGTGCATAATGAT

TCTAAAAACTATATATTGGTAGTCCCGCCGGAAATCATCGGGGAACTTGGGGGTTCCGCTCTCCAAGGGACAAGATCCCT

CGGAGAGGAACCTAGTCCCTTTCTTCCCAATCCCCTCCGGATCATCATCGTTCGATCGATCGGGGATTGGGATGGGAAGG

ATAAGTCCCTCCCGGGGAGGGACTAGTTTTTCAAAACTTGTTTTGAAAACAAACAAGTTTGTTTCCTGCAAACATCGATT

GGGGTCCCTAGGAGGCGCAACTTGTTCCCTCTGCCCGGCGAAGGGGTGGTTTTAATTTTTCTCTGCCAAAATACCCAAAC

TCCCGATCGATGATCGAAGGGAGAGGGTTTTTCCTGTAAAAACCCTCTGGGGCAGGGGCTCCCGAAGGGAGAGGGTTTCT

TCCTGTGTATTTAAACCCTCAGCAGGGCTCCCCGATGATCGAAGGGGTTTTAATTTTCTCCGCCAAAATTCACAGGAAAA

ACTCCCGAAGGGGTTTTGGAATTTTTATTAAAAACTCCCCTTTTCTTTTAATTCCAAACCCGAAGGCGACTTTTATTCCC

TAGCGGGAATCCGTAAGGTGAAGGATTCCCTTTTCTTTTTAGGAATGGAATCCCTCCGGGTTAAAATAAAGTTATTTCTC

CCGAGGGGAGAAAAGGGGGACAAGGTCCCGATCGATCGAAGAACGAAGAGGGAACTAGTTTTTCAAAACTTGTTTTGAAA

ACAAACAAGTTTGTTTCCTGCAAACTAGTCCCGGGGAACTCGTTCCCTCTCCCGGAGGGAGGGAGGGTTTAAAAACCCTC

CTCCCCTTTCTTTAATTAAAAAGAAGGCGACTTTTATTCCCTTCGCGGGGAATCCCTGAGGGATTCCTAAAGGAATCCCT

CGGGGATTAAAATAAAGTTCTTTCTCCCCGAGGGGAGAAAGGGGGACAAGGTCCCGGATCGATCGCAGAGGGAACGAGTT

CATTTAATTTAACTAAAAGAATTAATGATCATTTAAATAATAGAAGTTCTAATATTCTTTTACAACGTGCTTTTCCAAGC

CCCTCCCGGAGTATGGAATATGGTTTAAATAAATTTTCTTTATATATTATGGATATATTAAATATATCTACAGAAGCTGA

TTTATCTACAACAGAAGGATCTGTCCAAACTATGCAATAATTTATGCTTGAATTAGTACAATTAGAACAAATGTATCTTT

ATCTTTTTTTAATAAATACAACATTAATCCAGCTGCTGGTAGTAGATTAGGAGCCTAAGCATACCGAGGAGACTAAAGAA

TTGTTTAGTCAAATTAATAAATAAAATCCGCCTTTCTTAAATAAAACGTTTCTCCTCCCTTCGGGAAGGAGGTAAAACCC

CAGGGGTTTAGTGATGAAGTTTTGGAGGGTATGCGTAAGCGTATGTCTGGTTCTTTAAATCCTATGTATGGAAAACCTGT

GACGGATGCGAATAAAAATTAATTTCGGATTTCTTTAGAAAAGATGTATATTTATATGATGCGGAAGACTCTTACATTAA

TTAGTAAATATAATAGCCACAAGGATTTAATTAAAGAACTTAAAATTACTAGTAAAACTATAGTTCCCCAGTTTAAAGAT

TCAGGTATGGTGTATAAAGATCAATATATAATCTCGTAAAAAGTCCAGAGGAAATAACAGGAAGAAATAGTAGCATTAAT

TTTTAAGGTATAATTTTGTTTTGGTGCTGTGGGTAAAGTAATCCCACCTAGTTGTTATTATTTGTGGCAACGGTTAAGCG

AAACACAATATATATTCTTATTTGTTTTAATTAACTACTGTACTATGGTACGTTAGCTAGGGGTTACGCAAACTAGAATA

TAATAACAACCGGACAAGGTGAAAACGGTTAACGACACCCTAGTTGAAGACCGTCGCTGGCTTAATTACATCGCTACAGA

CTGGTTCACCTACGTAGAGCTTAAATGCTCGCGAATGTACAGTCGAAACTTAGAATCAGTATGGACTTACCAGGCTGTTA

AGCCTTAAAGCCGGGGCCTGCTGATTGGTGTGCAGGGTTATAAATATACACAGTGATATAAAACACCTGCGGGCAAGCCC

GCTTTAGATAACTAAGTTTGCATTTCCTAGAATACGTTTTGATCAATTAATGTCTTTTGTTGAACCGTAACTTTACCTAT

AGTTATTGCTTTTGTTATTTTAGTACCATGTATCTTGTATAGTTTTGAAATTATACCTAGTAATATATATTTATTATAAA

TTAATCTGGGGGTTTTAATAATCCCCAATTTATTAGTCCCCTTAGCCCGGGGATACCTCCTCGTTCGATCTAAGGGGATT

AGGCAGGCCGCTTCTGGTTCCGGGCTCTGCTAATCCCCTTTTATTCATCCGGGGATGGGACGCAGGCGTCCAGGAGAGGG

AATTAGTCCCGCCGGCAGGCGCTTCTTCTAGTTCGGGGGCTCGAGGCGGAGGGGACAAGTCCCAGGAGAGGGAATTAGCG

CGCGCATTATTTAAAGGCATAAATAATTCTTGTCCTTGTGCGAAGGGGAAATAGTTTTCGTTCCTCAAAACTTGTGGTTT

TATGAGGGACAAACACTATCGATGGGTTCGTGGCCCACAAAACTAGTCCAATCGGGGACAGCTTGGCGTTTCTTTTGCGG

TATCCGTTAGCATCCCAGCTATCCCTCATAGAGGGAGGAGGTACCTAATCCCTACGCAGCAGGGTGCGTAGGGGGATAAG

CCAGCAGCAGCAGGGGAGGCTAAGGGGAGGATACCGATTGGGTAAATTGGGGATACTACTCTTGTGCTCCAAAGAGGGGA

CAAGCCTAGCAACTTAGCTACGGGGAGGCCTCCATTTAGCAGCATTATTTTGTGGAGTAGGCCTGGCTCAAAATTCGGTC

TTTGCTAGCTCGAGGAAGGAAAACTCTAGGGGTCGGAGCTGCTTCTAAGACATGATGCTGCAACCAGCGTTCCGCTTTAT

CTTAGCGGACCAGAGATGAGAGATGTCACTTTTGGGGACCCTGGTCTTTCGTATCCCCTCTAGGGGATAGCGATGATCGA

TCGGGGATCCGGGGGATTAGGGGAGGGTATCGCTAGCTAGGGGGTAATCCCTCATCCGGCTAGGGTGGCAGCAGGGGGAT

AAGCCAGCAGGGCCAGCGTAGCGGCATCCTGCCTTTAGGCAGGTGGCCGTAGCCTTAGGGGATAAGGGGGTGCTCATCTA

TTTATTGGGGATTGATTGGGGGATTAGGGGATACCTGGGAAAAGCCAAACCCTGAATAGTGATCTATTCTTGCCTAAGCG

CGGCTTTATCATTTATGTTTATAGCAGCATCTCTATAAATCAGCCTTAGGGGAGGAGGCTAGAAAAAGCTGGAGGGGCCT

AAATAAAGGGATAAAACCCCATTCGGGGAATCCCAGGGATTTAGACATATAAATGATTTAATTTAACTAGAGATGGGGAT

TATATTATTCTAAAATTTGCCCAGCGGGTGGGGAGAATAGGAAGATTTTCATTATCATTATCATTATCATTATCATTATA

ACATCTAGGATAAGCAAAAAATATAATATTAAAATTTAAACATGAGAATTTTCAAGAGCCATCCACTATTAAAATTGGTT

AATTCCTATATCGTTGACGCACCACAACCCTCTAACATAAGCTATTTATGAAATTTTGGTTCATTATTAGCTGTATCTTT

AGTTATACAAATAATTACAGGTGTAACTTTAGCAATGCATTACAACCCTAGCGTTTTAGAAGCGTTTAATTCAATAGAGC

ATATATCATAATGTGCTCTTTAAATCAGGCTGTATGCTGGAAGATCTTCAGATTAACTAATCTTCGTTTAATTTAAGAAA

ATCAGCAGGAAACCTTTTAATTTGGATAGTTAATAGGAATCCTCAGAGACTAATACGCTTTACTACATAGCTATAATTAG

GTTATGATAGATGATATAGTCCAATCTTTTTGTGAAAAAGGATAATAAAATATGATTATCTTTTACCCTTTATTAGACAG

GGGTTCTAAAAATAAAATGGAGTCTAAGAGTTTTTCAACTATTTCAAATTCCTTTTCTAAAAAGTAAGTACTTTTACCTT

TAGGTTTTATCTTTAGGTAGTACTAACTTCGAGCGTAACTCCTTTAGTTTTCGGAAAAATAAAATTTTCAACTAATCAAG

TGAAAATAAGTTTAAGTTTAATATTCACGAAGACTTTTATTCTTATTCTCAGGATTTACAGATGGAGAAGGAAATTTTTC

TATTACATTTGATCGTGGTTATATAAGATTTAGATTTAAAATAAATTTACACATTGATGATTTATAAGTTATTAATATTA

TTAAATCAAAATTGAATATAGGTAGAATAATTATAGAAGAAAATCAGAGTAGGTTGTGCTTTTGTAGTACAAAGTTTTTA

AGAATTAAGAGAGATAAAACGGAAATTTAAATATAAAAGAGTTTAGTGTTTACTTGAGCACATTGTCTACTCTTGCTGAT

CCGTGTTACTTGGAGAGGCCTTGTAATGGTTTTACTCCTGGCCTTCTAGCTTTTCAAGGTTTAATAAATAGAGGGTTTTA

TCTTTAAGTAGGCCCGTCCCCAGTCAAAAATATTTTTAAGCAATCAGGTCAAAGAAGATTTTATTCTTCTTTCGGAGAAT

TGCATCTTACAGGACAATCTTTAAAAAATTAGACCCGTGATTCGTTACTGGTTTTACAGATGGTGAAGGAAGCTTTAGTT

TTAGTATTACCCCAAAATACAAGTAAAGAGGGTATGGTGTAAAGATTTTTTCTGATAAATCTTCATTCAAAAGATCTGAT

TCTTTTAAAAGTATAGAAAGTTTTTGGTGTTGGTCGTGTATATAAAAACCGGAAACATAGCTATATACATGGTACAGTCT

AAAGATGAATTAGAAATTATAATTAAACATTTTAATAATTATCCTCTAATTAGTAGTAAATTCGGGGACTTTTTCCTCCT

GTTATTTAAACAAGCGTTTTAGGTATTTAAAAATAAATAACATTTAACGCTGGCTGGTTTAATAAATATTGTGGCAATTA

AAGCCTCCGCTAATCGTGGACTTTCCGAAAAGATACCCGGGCTTTTCCTAATATTGTTCCGGTAGATAGACCGAGAGTCT

TAGACCAAGTTATTAAAGATCCTAATTGGATTGCAGGATTTTCTAGTGCTGGAGGGTAGTTTTATATTAGTTTAATGAAA

TCTAAAACTCTCTTGACCGGGGGCTCAGGTTTGGTTAACATTTAGTTTAACTCAACACAGCAGCAGATGCGGGATTAATG

AAAATATAATTTCTTACTTAGAATGTGGCGCTATTCTTTACGGAAAAAGCGTTGGCTGGAGATATTTAAGTTACAAAATT

CTCTGATCTTTCCTTAAAAATTCTACCTTTTTTCTAAATCTCACCCCTAGGGGTGAGGGATTTACCCTATTGAAGGTGAA

AAATCTCAGGATTTCTTTGATTTCTGTTCTGTAGCCGAATTAATTAAAAATAAAGCTCATCTTACTAAAGAGGGGGTTTA

GAGGCTATACGTAAAATTAAAGAGGGAATGAATAAATATCGATCATCTCCTTCTCAGGATTTATAAGAGGAAGTACTTAA

ACGGCAAGGATTTATAAGAAAGAAGTAATCAGAATTAGAAGAGGGCAAGTAGTAATTTTATATTTAGTATGCCCTATATT

CCTAAATTTTGCCTTTACATACTAGTAAAAATACGGATTTCCATGATTTTTATGCGGCTATTGTTATTAAAGCTAAAAGT

AAAATGGAAATCTTTCTGATTCAGATAAAGGGGAAAAATTTTATCTCTTAAAAATGGAATGAATTCAGGTAGAACGGTCT

TTAAATATGAAACTACTGGACCTCAAATTACAATAAACCCTAATGGATTAATTGGTTTTATAGAAGCAGAAGGTACTTTT

GGTATAAAAACTGGGATCTTCTTTGTATTTTCAAGTAGCTCAAAAATATACTAGTCAAGATAGTTTAAATTCGATAGGGG

TAACTTTTTAACTGGATTGTCTAATTTAGAGATACCTAAAGATAGCAAAATATTACCTGTAAATGTTACAAGTGCTACTA

ATATTAGAACAAATGTGGTATCTATAGTAGTTAGTAGTGTAGATGCTTTATATTATTATATTTTACCTTGATTAGATTCT

TCAAGATTTTATTCACTTTACCTTTCTTGCTTCTATACTTCGGATTAACCTTTTTAAGGATTTAAAAGAATCATTTTTGA

GGGATTTATAATAAAAAATTAATTATCCTATTATTTAATCCCTATAATCTTCAGTCTTATAACTCGGCTCGTATTACTAC

TATGAATAAATTCGTACTTTGCGGCAAATGAATGCTTATAATTTTATAAATAAAAGATGTTTTTCATCCGGGCAACCCTC

AACTCCTATTGTTTATACTAATGCGGACACGCAAAAGTTAAGTATATTAAAAGATAATAAGGGGAAAGAGGGGTATATTT

ATGAATAAATAATACAAATGGTAAGAAATATGTTGGAAGTTCAATCCGACTAGGTATAAGACTTACTCAGTATTTTAATA

TAAATTACCTAAAAATAAATTTATCTACTATGATAATATGTCAAGCACTGTTAAAATATGGTTATTCCAACTTTAGTCTA

TAAATTTTGGAATATTGTGATTCATCTGAGTGTATAAAAAGGAAACTCATTATATTGAAAAACTTAAACCGGAATACAAT

ATCCAATTATTCCCTAGTACACCTATGCTTGGAGTAAAACGTCGAGCAGATTCTATTGCTAAATTGAAAAAAGTTTAACT

GCTGGCTTGTCTAAATCTGATCAACATAAATTAAACCTGTCTTTAGGCGGATCAGGTTCTATTCAAATAGAAGCAGTAGA

CTTAGTTGAAAATAAAACGACAGTTCATCATTCTATGAGATCCGGCAGCTAGAGAGTTAGGAATCCAACCTATTACTATT

ATTAAATTTCTTTCAAACAATCAAGTAAAACCTTATAAAGGGAGATATATTTTTAAGATAAAATAGTCAGGGGCTGGTTA

AATATACCGCAGGTCGAGGATTTTAAGTTCTTAAGATTAGCTCTATTATTAAAGATAAATGGATACTATTATCTAACTGA

AGGTAAAAGTTCTTTTGGGATATTTCAGATATTATTAATAATTCAGTTGATATAGCACAAGTAATAATTCAGTTGATATA

AATCAATCTATTGAGAATATTTACGAAAGATTTTAAAATATTACCAAATTAGAACCTGTTTTAATGTAAAACTTTTATTC

CTCATGTGGATAATGCGCTTAAATTTATAATAGCAAATAAATCAGATAAACCTCGAATTGTTTATATATACACAAAGGAA

GGTTTAATGGAAGGGTTCTCCTTGGCTTCTTTTAGTTTGGCTCATAAGGCGGCGGCGGCATTAGGATTCAAATCAAGTAG

TAATACATGTAATCGTTATATAGATACTAATCCCGGAGGGATTCCTTTTAGGAATCCTGACGGATTCCCGGGGAATAGAT

TATATAAGGGAAAAATTTCTCATCTAAACCCATAGATAGTACGTCTAGGGATTAATGAAAATAGATAGTTTTGAGTGATT

TTTTCACTAAAAATAACCAACAAGGGTAATTAGATAATCAAATTTTATTAAATAATAATAAGTAGATTCACGAGCGGGCC

GAAACTTTAGTTGCATTTTGTACACCCATACAACTTAAAAATAATTTAGTTGAAAAATTACGATAAAATTGACGACTTTC

CTTACCAAACAATATATCTAAGGGTCTACCTGCCCATCTTAAAAGGTTTTACCTTCAGCATCCAAATAATTTTTACAATG

ATTAGTAGGATTTACAGATGGAGAGGGTACTTTTGCAATTGTACCTCAAAGTAATTGGAACTTATGTAAGTTTAAATTTT

ACAATAGATCTTCACATAGACGATGTCGAAATTTTACATAAAATAGTTAAAAATTTAGGTTTCAGGGGTGCAGTATCTAT

TTCATTTCATTTCATTTCTCAGTAAAACACGTTCTTCTGCTAGATTTGTTGTAAATAACTTTTAAGATATAAATTCAGTA

ATTATACCTATTTTTAAATAATTTCCTTTACAGACTACAAAATATTTAGATTTCATTAGCTTCAAGGATGCAGCATTAAT

AAAATCAAATTCCAAGGGTATTTATAAAGGCCGTATGATGTATAATAATTTTTCTCAAGCTGATTTGGTTAAATTAAAAA

ATTAAAAGAATATATGAATAGTGGAAGATCGGTTATAAAACCCAAACCAAAATAATTATTAAAAGACAAAGTTTCTATTA

ACAAGTGATGATTATTAGGTTTTGTGGAAGGTGAAGGAACTTTTGGATATAAGCATTTGGTCCCTTATTTTCAAATAGCC

CAAAATAAAAAGAAATTTCTGGGTTTTAGAGGCTATTGAAGGCTATTTTTAAATACTTTTAAATTATCCAGGGATCAATA

AGTATTTAAGTATTCTTTAAATAAAGGTACCAGGTCTATTCTTTGGTAATTTATACAGTAGATATAAATTTTTATTATAT

TTTACCTTTTTGAATCCATGACATTTTTAGTAGAAAAATATAGATTACCTTTATTGAGTTATTATGGTAATAATACATAA

ACTAGGTTATTATTATTTACCAGAAGGTAAAAAATAGCTTTAGATATATCGTCAGCGACAAATAAATATCGTTATACTAC

TAATAACTCAAGTAAAGTAGAACTACCTCGTTGGGAGTTAATATCTAAACTATTAGCTCAAACACCTCCTTTTATATTTC

AAGTGGTAGTCATTTTTAGTTGGTTAGAAAATTCATTATAGCTAAAGGGGACGAAAAGGTTTTATTGTCTATATTTATGA

ATCAAGTGGGGCTATCCTAATAAGTTAAAAGAATTAAATGGGGGTTCACCTTTTTCTACTTATGGTGATGGACATGCATT

CATAGGTTTAAATAGAGCAAGTAGAGTAATAGGGAGATATATCGATACAGGTAAAAATACCAAGATAAATATATAATTTC

GTCTATTCCTCTCCCAAAGGGGAAGGGTGTTAGATAAATAGTCATTTAGTTTCTTTTATTAAAGGTTAAAAGCAGGAACT

GGCTTCATATTTAGTTTAGGTAAAGGGTAAGTAAGGAAGGAAATAAATAAAATCTCGTGAGTGTTACATAAATTATATAA

ACTAGATCGGCACAGACTCAATCTAAATCTTCGATTATGAGAGATGTAAACAATTTACGACATGACAGCAAATGTATTTT

AAATATAAGTAATATATTTAATGCCCACTCAGTAAAGCCAGGGCTAGATTATCCAAACAAGTTGGCACCAACGAGTGCCA

GGTTGAAGTTTTGGGGAATAGGTGAAAATTACTGGTGTTCCTTTATATTGAAGTAATAAGTAGTCGTTCGACAGGCGAGT

TAGAAGGATGAGCGAGAGCTAAGAACCATTTGAAAACAGGTCCAACATCAGCGATACCAGCAGGAGCATTTGGTGAGCGG

AAAATAAGATGTGGAATGTTTGGTTGTTGACATTCCATTACTGAGATCTTTTCCAACACCTGAACAGGTTTAGTCCTAAC

TAATCAATGCCGTACGTTTGTGAAGCATGGGAACCCCGGAAAGCTTCTAAAAGAAGTCCTGTACGACTTAACTTACGCAT

GGGGAAAAGAACCAAATTATTCACGGATCTTCTACCTTCAAAGAAGATAACCTTATATCCCGTAATAAAGGGGTTTTTCC

CTGTTTAGCTGCAATAGTTAATACGATCGGTCGTGTCAAGTACCTCTATTTTTGGGAAGGTTATAATACAGACTGAAGTA

GAGACATGGACGGGGCAAGATAACTGAAAAGCCAATGCTCAAGGTAATTCTTGAGATACTCGAAAAGAGATATTCAGGGA

ATTTTATTCCGGGTCGACAGAATATGCCCAATTCAGTAATGTCATGAGGTAACATTGATTCAAAATGTAAGTACACGAGA

AAATTTTCTACATCTACCCAAGATAGCCCTTCCCCTAAGGGGAAACAGAAAGGTCACCCCTAGAAGGGGTAGGCCCAATA

GGGAAGTAGGCAGAACGAAAACCCAACCGGATGACCAGATTCAAAAGAATGAAAAGTCATAATGGAGGACGTATACCAAA

ACAAGTTGAATTAGTGAATATCGCGGTAAAATGTGGATTCCACAGTAAAGAAGTACAAAGGAAACAAATCCTTCTTAGTC

GATCTCTAAACTTTGCCCTATTAGCTGTAAGGAAAACAATCCAAAACACCGGGAGTCGTACACCAGGAGTTGACAATAAA

ATTCTAACAAGCAGCTCCCCGGAGCTAGAAAAACTAAATATGGTGAACGAAATCCAAAACTAGTAGTAAGTGCCAACTAC

CGAAGCGATCCAGTAAAAGGTGTTAAAATACCCCAAGCTAATGGGAAAACCAGAACACTAGGTATACCCACCATTAGAGA

TCGATGCCTGCAAGGTCTGATGAACTTAGTTCTTGAACCCTTAGTAGAATGCAGAAGTGATCATCATAGTTATGGATATA

GAAAGCATAGATCTGCACAACATGCCTTGGGTTATCTCAGAAATCTGTTCTATTCCAGGTTTAATCTCGAAGATAAGTAT

GTATTGGACGCAGACATAAAAGGATTCTTTGATAATATCTCACACGAATGATTACTAGAGAATCTACCATTGCCCAATAT

TCATAAAAGGTAGTAGATAAATGGCTAAAAGCCAAAATGTTCAGCGGAAAGGAAATACTTAACAGAGAAGAGGGTACCCC

GCCGCAAGGAGGTATAATATCACCAACTCTAGCCAACTTTACGTTAAATGGGCTTGAGAAAGTGATAGACAACTCAATTA

CATCGATAACGAAAAGTAAGCAGAAAACGCCAGCATTAAGGTCAGGACTTAAGTTAAATCTAGCTATAGTATCCGTGAGA

TATGCGGATGATTTGGTTGTAACTGCGAGAAGTAAATACTTGATAAAACCTTTATCGTACCCGAAATAGAAAAGTTTCTT

AAAATAAGAGGGCTAGAGCTCTCTAAAGAAAAACCCAAATAGTTACGGTAAAAGAGGGGTAGATTTCCTTGGATACACTC

TCAAATTCAGAGAATACTGGAAAGTGAAAGATCACTTTTAAGGAGCGAATTGGAAATGAGGGAATAGCACTCTATCCTCA

GAAAAATAAAGTATATGCCATAATAGCTAAACTCAGGGAAATATTTAGGAAAAGTTCTAATGAATCGGCCTATACACTTA

TATCCAAGATAAATCCCATATTACGCGGGTGATATGGTTACTATAACTTGTCACAATCTACGAAGTTCAGGGATATGTCA

GACAAGCAGTGTTTAGTTTTGCGTTAAATGAGCTATGAAGAAACACCCCAAATGAGGATGGAGAAGAATAGTAAATACAT

ACTTTCTAGGTATCAAACACGAGAGAGGGCTGAGAAAAAGTTCAGACCTAATTGAGTTTTCCGAGGTATAACCAGAGTGG

ACTCAAGGTTCACCGAGAAGACAGGAAAAGGAAAGACTAATTATTTAGTAAGTCCCACAGATGTACCAATAATAAACGGG

GCATCTATGTCAATACCCGTCAATCTGAGGGAAGTTCATGCTTATCACTCGGAAAGCAATAAACTTAAAGAATTCAACGC

AAAAATTAGCGAAAGAACAAAGGGAAGGAATCTAAAATCCCAAATTTGGAAATCTCCAGAGGGAAATGTACATACTGTGG

AGGCAGAAATTCAACTTAACACACAAATAATGGAACTAAGGCCAGAGTTCGCCGCATCACATTGTACCAATATCCCGCGC

TTATGGATGGATTAATTGGGGAGGTTCGAAAAGTAGTGTGGAAAATCTGGAATTAATACATAACGAGTGTCACAAATTGA

AACATAAAACCGCCGGGTAAATCTCTAAGATAGTCTACACCACGGCAGACAACTCAGAAGAGACAACTCGCATCATGATT

GAGCCGTGTGCAGTGAGAGTTGCACGCACGGTTCTTTGAGGGGATCTTGCCGCGAGGCGGGTTCCATCTCTAGTGGGTGA

TTAATACGTTACATACATAGTGCGCCGTGGGACGGCTTTTCAGGGAAAAATTGGATCACCGGTAACCATCCGATCCTCTT

ACTGATACTTCATTATTCGCTAATAACCATAATGACAGGTGAGCGATCAGTAGCCTTTTGGAAGTTAGGGCTGCCACTAA

GGACCAAACTAAGAGGAAGAGTATATGATGAGGCTAACAAACTTGATACATCACTTCGGGCACAGTATTCCCTATCAGTC

CTAAGCAGAATACGTCGGCTGATATCACCTCTATACGTCCTGGTCTATGTAACAGGACAAAACCCGGTAGAGATGACGAA

CATCCTATAGGAATGAAAAATTGGTGCAAGCATCTAAGTACTCACAACGCCTGAATACGCAAACCATGGGAACGAACCGC

CTATGCTACAGGTCTATTTAGATCTGACAGCAAGCGGAGTCGGAGTCCTCGTAGTAGTTTAACGAAGGAGGACAGTTCAA

ATAGACTATACTCTTCAACAGCTCTGAAACGTACAAAGGGTTCTGTAGAACCGGACTCAAAAAGAATGAACGTTCAAAAG

TGCCTGTTTCTAGCTGACTGAAAGAACGTATAGACAGTTTACCCAAAACTAATGAGAGACTAACCAAAGTAATAGATATT

ATCTCGGACCCGGAGTTCCTATATGCAGCATATAGTTTAATCAAACACAAACCAGGTAACATGACTCCGGCGGGAGTTGA

TAAAAGACCCTTGACGGAATTGATTGGGACTTCTTTACAAACCTAGGTGAAAAGCTGAAACAGGGAAAATTCAATTTTGC

CACATCCAGAAAGGTCAATATCCCCAAAGCCCCCAATGAATGGGGACGCCTTGAGTGTAGGCCTCCTAGAGATAAGATTA

TACAAAAGCTCTCGCCCTCGTGTTAGAAACTATCTTCGAACCTAACTTCTTAGATAGCTCCTTTGGTTTCAGAGCAAAAG

AGGGGTTCATATGGGGCTAAAAGAGCTATACCTAAAAGGAGGGAACTACAGTTGAGTGATAAATGGAGATATATCCCAGT

GTTTGCCGTCTATTCCTCATCAAGTTATCATGGGAATATTAAGGAAACACATTTATTGTGCTAGAACGTTAGAGCTTATA

CCAAAATCTCTGGGGCCAAGGTATTGGATACAAATGGAAAGACCATTAAAACTACCATTGGAACTCCACAGGGTAGCGTA

GTAAGCCCTATTTTATCCAACATAGTATTACACGAATTAGACAAATTCATACATAGATACAAGGAGAATTTTGAAGTAGG

CAGAAGCAGAGCGGTTAATCCGAAATACCACTCTCTTAACAGCGTGCGTAATAAGACGAAGAATGCAGAATTAAGAGCCA

ATAACTTAGCCTTAATGATGGATATGAATCCAAAAGACACGCAAGACCCCAACTTCAAAAGGCTACTCTATGTCAGATAC

GCGGACGATTTTGTAATACTTTTGATCTGCAGTTTACAGGAAGCGTTTACAGTAAAAGGAGCCTGAAGGATTTTCTCAAA

ATGAAATTAGGACTAGAACTTAATCTCGACAAAACTACTATTGAGAGTACCAGAAAAGGGTTCAACTTCTTAGGGGCAAC

CCTTCGTAAAGTAGATCAAGTAGTGGTAAAACGAGCGGGCGTAACCAGAGTAATCACGTGGTGAGAAGAAGATCTAATCG

AAGGTTAGTCATTAACGCACCACTGAGAAAACTGGTAGACAAACTCATTAAAACAAGTTAGCTAAAAGAATCATTTAAAT

CAAGTGATTGCAACTTCTAGAAAAGATCTAGTTAATCACTCACACTTTGATATACTAAATTTTTATAACTGACGTATAAG

AGGGATTCTTAATTTTTACTCTTTTGCCGCAAACTACTCATCTCTAAGCAGAATAGTCTGACTGTTTACTCATTCATGTG

CATTAACGTTAGCCCTTAAACATAAACTTAGAACAGCTAGTAAAGCCTTCGAAAGATTTGGAGAAAGACTGAGGGACCCC

GCAACCGATACCCTAATTTACAAGGAGGAGAGCATGAAAGTTAAACACGACTATAAAGTTGCGGTAGATGAGCTACCCGA

TCTAGATAAACTACTGCGGGCATCCCCTCACGGGAACTCTTACGAAAAAATTCGTGAAACTGTATGTGCAATATGCGACT

CAAGCACTAAAATTTAAATGCATCATGTACGGAAGGCCTCAGATATTAGGAATCCCTCAGATACGTCAGGGTAATTCCAC

TTACGCACAATGAGTGGGGTTTCCTTAGAAAACAGGTCCCTTTATGTCAATATCACCATCAGATGTTACACAAAGGTCAA

CTAAACCATGGCGATTTCAAAAGGGTTAGTAATTGAAGGGAAACGGAGGCCTAGGGTCAGACTCCGAGAAAGAAAAACTT

AAGAGATAATAGATATATCAAATTTGGGGCCAGGCTCGAATCTCCCTCTATAAGGGAATACGAGCTAAGGTTAAGAAACT

AACAGCCAGGGGACCAGGGCTGCGGGCGGTAGGGGCCCCTCTGGGCAATCATTAGTAATTAAGGAAATTGTATTAGTTGA

ATGGAGAGCCGTATGATGGGAAACTATCACGTACGGTTCGGGAGGCAGGGTAACCAATCCACTGACCTCACGTAACACAG

CTTCAGCTTTCTTTTATAGTTTATTTACACAGTGCGACAAGAAGCTGATAATTGCAATGAGGTGGGATTCCTCCGCGAAT

GACCCTTCGTGTTCCAACTCAGACATGCGCCGGGAGTAATTCTGGGGTATGAAGCTCTGAATATTGGTAAAAAGTTATGG

TAAGCGAGAGCGAAGATATCGAGACGGTTCGTTATTCCCTTCTACCTTTACTATATAAAAATAATTAATCTAGACTAGCC

AAGAGATAGTTACTTTTATCTAGTATAACACGCCAAACGTTGTTTCATAACTTTTATAATATCTAACTATTTAGTTGGCG

AGGTTTTATCCTCAGCATTATATATTAAAGGTGAAGAGGAATTGTGTAAGAGTGTGAGGTGGTGTGTAGCCGAAGCCGGG

TCTCCCTTATTTCAAAAGAAAGATATCTAGGTTAGATTTAATACACTATGAAGCAGTGCTCTGCTCTAGATAGAAATAAT

TTATTATGAAGTAAGGAAAATGCAACGAATTAAAGTATCTACCTAAAACTTTGTTAAATTTGGTGCAATAATTGGAACTT

GGTAAACCCAATGGTCTCCTTTTGCCATTTAAGTAAGGTCAGAGGGAGGTACTCTATAAAATTAAATTTGAAGTGAGTAT

ATCGACCAGTGGGCATGAGACAGTCAAAAGCTAATGCCACTTTGTAATGAAGTGGATAAAGGGAGAGCTACCTACAATGC

GATAGATTCTAATATCTGCAGATAGATAATGGTATTTATTTAGCTCATACCTTAACCTGAAAGGGTGCAGACTTTGACAT

GGGCAAATCGAAATAAAGAAGTCGAGCTTCGTGCTTCTAAATAAATTACGAAGGATTAAATAAAGCGTCAGAGGTTACAT

AGCAAGGGGCTAACGCTTCGCTAGGCAAAGCTTTGCTTATTAGCTAGCAAGCTCGTTCTAACTTCGTAACTTAAGGCTGC

TTGCAAAGCAGGGCTGTAGTTAGCAATATGCCTAGTAGTAAGCAGCCTAAGGTTTTACACATAAGTATACTACCTATGCT

TTATTTTTCCAGCTATTAGTGTTATAGCTAGCCATTAAGATTATAAAATACTTTTGTGCGCTTAGTTTGTGCAGTAAAAG

AAAGTTATTAGCTATACGAAGAAGCGAGTTTAAGATATTAGCTTGTTATTTGCTCTTTTTGTTTAATTTGTAAACATACA

AAATACTAATATATATATTTTTATTTTTAATTAGATTGCTTGAGCCGTATGCGGTGAAAGTCGCACGTACGGGGTTCTTA

GAGGGGAAAATCCGTAAGGATCTACCTATCTCAATTAGGATCAAGCTCGATAGTTACAGTTGTACAGATAGATTCAAAAG

AATACGTGGGGTCCACTAAAGTAAGTAATAGTAATATATTTAATTCCGTCCTTTCTTGTGGTTCTTTTCCTTTAATAAAT

TAAAGTTTTCACCTGTTAGAGGGTATTCTACAGGTACGGCTGGAGAAACTTCTAGTCCAGAGTCTACATTTATTACATTA

AATGGTCGTACACTTTCTCCAGAATTTCTTGAGTGATTTAGAGGTTTTACAGATGGGGAGGCTTCTTTCTATATCCAGAC

TACAAGAGAGAACTCCAAGATTCAATTTTGTTTTGAAATTAGCTTGCACGTGGACGATACTAATCTTCTAAACTATATTC

ATAACTCTTTAGGTTTAGGTGTGATTTATATCAAGAATAAAAGATCTACATTTATTGTGCGTAATCTTAATGACGTTGCG

GTAATTATTGACATTTTTCTCGTAATACACTTAATACTACTTCCCGGGGACCTTGGGTCCCCTTCGGGTCCCTCGGGGAA

AACATTTAAACTTTCTTGATTTTAAATTAGCTTATGATCTTTATAGAAATAAAGATCAAGGAGGAGATTCTAACATTATT

GCAAGAATCTTGGCAATAAAAGGCCAAATGAATAGGAGTAGAACTAATTTTGACTTAGGTCAAAATCACGTAATTAATAT

AACTTTGGACTGGTTCATTGGTTTCTTAGAGGGTGAGGGTTGCTTTAATTACTCTGCCAGTAAAAACTTTTCAGTTTTAT

TGTTGTTCAGAAGGATAATCTTCCTTTAATGGAAGGAATTAGAGACTTTATTAATAATCAGGGCGATCTTATATTTCACT

ACTAGTTGATAGTACTGAGAAGTCTGCAGCTAGAGTTTACAGTGGTAAAGCTGACTCTAAGGGTCAAATTGTTAATGAGG

TAGTGGTAAGACAGTTAGATTTCATTACCAAGGCTCTTATTCCTTTATTAGATTCTGCTAACTGATATAGCAAGAAGGAA

AAGGATTATAAGGACTGGAAGGCCCTTATTAATCTTGTAGAATTAGGCCTTCATTATACAGATGAGGGTAAATTAATAAT

TGAGCGTATTTTGGCACAAATGAACAACAATCGTTTATCTACTAACAATACTTACTCAGATGAGGATAGAGCTCTTCTAC

TTTCAGATATATCTCTTTTAATTAGCAAGGGTTCTAATTATATTAATAAGGATGGTATGATCTTTATTAAGTCGTTAAAT

AGACCCCTTAGTACTAATAAGAAAGTGGCTGTTGAAGTGGTAGAAGTTTCAAGTGGGGAAGTTATAGACACATTTAACTC

AGTGAGTGAGTTAGCTAATAAGTTAGAGATTAAAGTACAAACTGCTCATTATAGAGTTAATAATATATCTAAATTTACTT

GCAGTAGATACAATGAGAAATTGGTTTATATAAGAAAATTGTTAATTAGCTAGTTATTACAATTAAGGCGGAGTAAATTT

TATGATTGCTTGAGCCGTATGCGGTGAAAGTCGCACGTACGGTTCTTAGAGGGGAAAGCTCGAGAGGGCCTACCTATCTC

GATTAGGTCGTGGGCTGTACTACGGATCATACAGAGCACCTAGAACATTAGTTTGAACATTAGGTGTAGTTATATTCATA

TTAATGATCGTAACTGCATTCCTGGGTTTTTATATAGCCCAATATGGTTAAAATTAAACCAGAATTTCTACAATAATAAT

AACAACAAAAATAATACTACGTCCTTCGGGATATATATAAGGAAAATTCTAAATCATTCTAACTCCAACTCCTACTCCCC

GTCTTTTCATCACGCCTTGAATAAAACACGGGGAGGGTGCTTAGATTTGGTTTAAATAACAAAACGTGGAACAAAAGATC

CTTTTCTAACTACAGCTATAACAGTTTAATAGTAAGATCTACACAAAAGAATAAAGAGAATATGTCCCTAACAAAGAGTT

TAGATCTGTTTCCAGACCCTACTCAAAAGTCTAGAGCACCCAAGGGGTAGTAAACCCTTGGGATGGGGGCAAGTTAGAAA

AATTTCTTTTAGAAAAAATCTTAATCCAGTTTTTATATATGAAAATTTATCAAATAATACTATTAAGACCTTATTAAAAA

TGAAACACGAAATTTATCTGGTATATATTTGATTTTAAATAAATTTACTCTGGATTATTATATAGGATCAGCTTCTACTG

ATAAATTATATGCAAGATTTATTAATCATTTATTTAATTTTAATGGTAGTAAAGTAGTGAAAAATGCAGTAAAAAATACA

AGATATCTTCATTTGCATTTCTAGTTTTAGAATTATTTCCTGAAATAGTCAATAAAGAAAACAATAAGAAATTATTAGAT

TTGGAAGACTTTTATTTAAAACCTTATTACCCAATTATAATATTTTAACTGAAGCTGGTTCAAGCTTTGGCTATAAACAT

TCTGAAATAACTAGAATAAAAATGAAAGAGAATTATAGTGATGAGCGTAGAAAAGCTATTGGTAATTTGAATATAAATCA

AAAGTACGACGCTAAGGCTATTGAATTAATGAGACAGTCAGCTGCTTTAAATAGGGATACCGGAGGGAAAACCTATTTAT

TCTAAACGGCCTCCCTCACTCCCCACCACCCCGAGGGGTGGGAGGAGGGTATAAAACAGGCTAAAGAAAACATGAAAAAA

AGTATAAAGGCATAATAGTATATAACTTTGATTACACAGTATATGGGGAGTTTAGTAGTATAGTAATTGGCGCAAAATCT

TTAGGTTGTAGTGAGAGGACTATAAGGCGGGCTTTAAAAATCCCAAAAATATACTAAGAAGACGTTGAATTGTCAAATAT

GTTTAATTATTACTTGTTGAAGCAAATCTGTTTAAACATTACCCTCTTCAGCGGCCAAAAGGAGGGGTTATTGTTATGTC

TTTAAGTATTAATTGTTGTTATTAAATTTGTTTACCCTCTTAACATTCGTTCTGGATTTTTTACCTCTCTCTCACTACAA

GGGGTTTTACCCTCTTAGGGTAAGGCTGCAGTCCGTCCGCAGTAAAAACCCCAGATTAAACGGGGAAATTTTAATTATAG

TCCCACAAGTAAAATTTTTATGCAATCTATGAAGAAAAATAAAAGTAAAGTGGAATAGCCCGACGGGGTATTGAAGAAAC

ATTTGTTTCTTTGGCAATGTTAGTGGTTTATATGCTCCAAATAACTTTTATTTTATATAAATCCTTTTACGACCGGGCTG

ATATAATAAAAATAATTTTTGAGTGTATAATTACGATCAAAGAATTGTTATTTCATATTATTAAGAAAAATAATAAGCTA

GCCTTCGGGCCTTAAGGGGTACGCCCTACGGATTAGGGATTCCAGGATTAGAGGCGCGGGCTTGCAGCCGAAGGCTCCGC

TCGCTAGGAAGTGCCCTCCTACGGGGCCTTAAGGGCGTAGGATTAGCTGAGGAGCTTGATATAAAATAATATATTAGAGA

TCGTCGGTTATTCATATGATCGCGACAGACTGGGTCACTAATGGGTGTCTGAAATGATGCTTAATGCACAGTCGAAACTT

TTAGTTAAATATTTAATTAATAGAATACACGAAATCAAAGGTATTCTAGCTTTTATAAATGGCGCATTTAAAAAAAAAAA

AAAAGTAAGTTTGTATGTGCTACCGTTCGGTCAAATGAGCCTCTGGGGTGGAGGGAGATTATTGTCATCATGTGATTTAA

CCCTCCTTGGGCGGAAGTGGAGGGAGTTTAAAGCAAATAAAAAATTCATGAGTATGTTTGTTGGGTTAGTTGATGGTGAT

GGTTACATTGAAATAGGACCTCAAAACAATATAATAAATCTCCAGATTTTACACCCAAAAGTACAATTAGAGCCCGTTTG

GTTATTAGATTACATAATAGAGATACAGCTTTTTGACTAATCTAACCAAAATACTAGGGTAGGTTCTATTTCTAACTTAA

CTTCAGTTAATCAAACTAGATTAATTTTCAGCAAAAGAGATTTAGTGGGAGTAATAATACCTCTAATGAAACAATACAAT

TTACAGTTTTAACCGGAAATAGATCAAGACAATTCGCCTTACTTAACCACATAATCGACAATAAAATTATACATTGAGTT

GACGTTAGGTTTAATCCTTCTTTTAAATTAGCACCTGTGTACAGCTTAAATGATCTGGTTAATTTATCTTTTTAAAGATT

GAGTAGTAGGTTTCACTATTGCAGAAGGGTCCTTTGGATATAAATGCGAGGGTTCGGCTTTCTACCAAATTAAACAAAAG

GGTTGGAAAACTATGTTTTAATTAAAGCCATATGCTTGACTATTGCCGGTAGAGAAGCGAAAGCCATAAAGCCTGACGTT

GCAGATTGCTATCAATTAACGTTGTCATCTAAATTAGATGTGCAAAAGTTGTGGATTTTTCTCATCCGGCGTAAATTATC

CATTGTCTGGTTATAAACTTAAACAATATAATGTATGAATTAGAGGGCTCAGGGAAAGTGTTCGCTATCAGAGCCTTATT

AACCTTAAGGAGATTCCTGGTTTTACAGCTAGCTGCAGCATTTAAGACTTTTCCCAAGTGGTTTTTATATTACGTGATAA

TAATATATTGCCCCAAATGCCCGCTAGTTAGCTTTTATACCATTTATCCCCGATTGCTTCTGGACAGTTATAAGTTGGTT

TTAGTCCTTACTACAATTAAATATCCTTGCGGCGTCTTCTTTCTGAGGGGACTAAGCGTTTTCTTACCTCGGGGCCTGCA

CTTTGAGGCACGCCAGCAAGCCAGGGTTGACTGGGGTACCTCGCGAGCAACTAGTTTTGTGGGACACGAAACCCGCCCGT

GTTTGTCCCTCTCGTGAGGAACGCAAAACTAGTTTTAAAATCCGGATCCTAACCTAGGGGTTCCTCCCAGCACGCAGGGG

AGGAGGCCAGCCAAGCGTTCGCGAGCAGGGGCACAAACCAGTCCAGGAGGCCAGGTTCGGGGGCTCTCGCGCGCCTTCTT

CGAAGAAGGGCCTCATCCCTCTAATCCGGCGATTCTTCGAGGCCCAGGGTTTTCAGCGTAGGCCTCGCGTGCGTAGCCCG

CGTGCGTGGTAGGGGAGGGAAAAGGCAGACCTCCTAGAAGGGGATACCTCGAGAATAGGCGGGATGGGACGGGCAGGGGT

GCCTTGCCAGCGTCGCGGGAACGGGGAGTGGCTAATCCCGGGGGTATCCTCCAGTTTCAGTTTCTCGGGATATGGACTAC

GATAAAGTAAACTTTCACGAAGAGATTAGTGGAGTGGAAATTTATAAAAAGTCAAATTAGCTTGGTATAGTCATGTATTA

AATAGCCGCTTTACTTTTACTAAGAATCGGCTTAGTGCTTTCCAGACGGTGGCGCTTTTCTCCAGGGTGCGCGGGCCGGC

ATGAAAAGACGGGGATTAAGTAAATAAATAGATTTTACTTCTCTTTATACATCGGCGATACTGATGAAAACAGGTCAAAA

ATATTGGTTTAATATTTAGACCGTCGGTAGAGTAAACTATCGCTACAGACTGGTTCATCGGTGGGTGGCACTTATGCTGC

TTAATGTACAGTCGGAATCTTTTTCATAAACAAATTTTTTATTGTGAAAGACCAGGGCTTTATGCTGTTTTAAATTACAA

TCATCGCCTTCGGGGATACCACCAGCCTGAGGGTATATCTATTGCAAAAGGTAAGGTAAGGTAAGGTAAGGTAAGGGAAG

GGAGGCCTTGTTTTAAAGCTCCCTCCCTACCTTTTGCTTTTAGTCCCTCCGCGATCCCGAGGGGTTTAGTGGAGTGGAAA

AAAGGATTAGTGCCTTGGGGACTGTAGAGGGGGACTACTTGGGTAGGTAGTAGAAGGCAAGAAAGTCTTCATGATGAGCA

TACTGTTCTGGGTTTGCTTGTTTTAAACATCCGGGGATTCCCTGAGGGAATCCCGGAAGCATCTATAAAATTGATAATTT

GCCCAGTAATCCCTCTTCGAGAGGGATTTTCCCTGCAGTAATCCCTCTGTCAGAGGGGATAAGAAGGGATTTTTATAATG

CTCCCTAGGGAGGATAAGCAAATTAAAGATTTGGCAACCGTTAAGTAATAGCGGTTGTAAAATATCACTATATGCTGGAA

ACTCTTATAACTAAAATACTCTGTCGTAAAAATTTTTTAGTAAAATAGGTTAGTTTAAGACAATCAGCCGGAAACCAACG

GATAACATTTATCTTAGTAAGATCTTCAGAGACGACACGTGATACATCTCCCTTCTTTTCCTCAATCCTCTTTCCACTAA

CCCAGGAAACCTGAGGGAGGGTAATATAAGAAGAGAGTAAAGATATAGTCCATAATTAAAGAAATTTAATGTTGAAACCA

CCTTTTGTAATTTAATTAGTTTATGCAGTGTAAATTTTTCAGATTCCCACTATTAAAATAAACCGCGTCCTATTATCTTG

TATTTATTCTTTTATACAATTTTAGGACGTTTGTGGGACAGGGTTGATTTAAAAGGTTTAAGTATTACTTCTTTAATTGT

TCTTTTAAAATTATTTTCGTATAACTCCCGATTAGCTATAGAAGATACAGCATATTCAGGTGAGCTGGAAAATAACAATA

ATGGATCCCATGAAGTAATGCGCAGCAAGTCTGAACTTAAACCTTCAATAAGTTTCTAATTTATTGAATGATTTGTAGGT

ATCAGTGAAGCAGAGTCTGGTTTTATGCCGAGCGCGTAAAAATAAAGAAGGACTAGTAGTAGGATTTTAGTTTGTGTTTA

GAATAGCATTGCATCGTGACGACCGAGGTGCTTTAGAACACATTTTCTCTTTTCCTCTGTTTCATATGACCCAACCCTGT

AGACGCAAGGGAAATTTTTACATAGGTTGTTATTGATGCATTACTTATGTCTTGTCGGCAAGAGACCTTCATACTCTCTC

AGTAGAGAGTGCCTCATTAGAGGCCAGAATATCTCCACAAGAAGCGAATGAAAATCAAAAGCAGGTGGCAAACTTGACCC

TTACTATATAAGTGGGTTAACGGATGCCTGTGGTAGTTACTCAGTTATATGTACTAGACATAAAAATTGGAGTTAGGCTG

AAAAGTACAAGTTAGTTTTCAACTAAACATGCATATAAAGGAGCAAATAGTTTTAGAGAAGATTAAATATACTTTAGGCG

GAGTAGGTGAAATTCACAAGCAGGGTAATCAGGGCTGTGTGTTTATAGTAAGTTCTGCTTTACAGTTAGCCAACACAATA

ATTCCCCATTATGAGAATTACCCTTTAATGACTAAAAAAGAGGAGAATTTTAACTTTTAAAGAGGTTGTCCGAATGATGC

ATAATAAAGAGCATCTAACTACGGAAGGTTTTCAGAGAGTTTTGTCTATAAAGGCCTATATGCGTAACGGTTTAACCGAA

GCATTAGCTGAAACTTTCTCAGATGTCGTACCAGTATCTAACCCTTGCCTAATAAATGCTCCTACGAAGAGTAAGGGGTG

GAAACTATTAAATATATTGACCCTAATTGATTGGTTGGCTTCACTGAAGGTGAAGGAAGGTTTAGAGTAGATATCAAAAA

TCTACTTCCTGTAAACTAGGTAAAACCGTTAGACTAAATTTTCAAATTACACAAAACGAAAGTGATAAAGAGCTCTTAAC

TTTAATTATTTTATATTTAAAATGTGGTACACTCCAAAGCCATGGGACAGGGAAAACGATAACTGTTACAAGGTTTTTAC

ATATATAATATCATTATACCGTTCTATAAAAATATCCGTTACAAGGGACTAAAAGATTAGATTTAGCGCTTTTCATTGAA

ATTGCGGAGTTAATTCAAACCAAAACCCACCAGACTAGCGAAGGATTGAATAAAATTTTGCTTCTTAAATCCGATATGTC

TTTGTACTTAAAAGTCCCTTATCAGAGGCGGAGCTGACCACGGATGTTTACCCATTCCCCGAGGGGACCCCGATGATCGA

AGGGGACCCATTCCCCGGGGAATATTATTACTTATACTAACGCAGAGGCACAGAAAACCAAATTTTAGCTGAAAATAGAA

ATAAGTCTGGGGTTTATCGTTGAAAAATATAATATCTGGTAAAACCTATATTGGAAGTAGTAAAATTTCTGCACCTGATT

TATCCAGTATTTTAGCCTTAAAACCCTAGAAAGAGGTCAGGGAAAAATACCCTTATAAACAAAGCCCTCCTAAAATATGG

CTATTCTAATTTTAGCTTAGAAATTTTAGAGTACTGTGATCCCCAGATCTCTATTGAAAGAGAACAATATTATTTGGATC

TTTGTATTGGTGAGTATAATATCTTAACTATAGCTGGTTCAATTAAAGGCTTTAAGTTTTCTGCGGAAGCCAGGTTAAGG

ATGTCCGGTAGAAGGCACACTGAGGAATCTAAAGCTATAATGAGGGAGGCTAAATTAGGTAAAAATCATCCGATGTTTGG

TAAAACGGTACCGCTTCAAATCAAACCAAAATTGAGTATAGCGAACGGTACAGCTATAAAAGTTACAGATTTAGAGTCAA

ATCTTGAAGAAACTTGTTTTCAATTAGAAAAGCTGCGGTGTTATTATGTGTAGATCATTCAACCCTTTCAAAACGTCTTA

AGGGCTTCTAACTGCTTTATATTAAATGGTAGATATAAGGTAGAAAAGTTTTAATTGGTTTTGAGCCGATCGGCGCGCAA

GATTAGGCTGTGGGAGGCTTAACACCGAACGAGATACATTAGTATTTACAATATCTCAATTAAGTGATATTGGAAAGATC

TTGATCCCTTTGTTTGAACAATTTCCCTTAAACTCTACTAAACTTCTAGATTATTTAGCATTTAAAAAGCATTTTTATGT

TTTAAATCGAAAAGTTGTGAACTAAATAAACCAGATTTATATCAAAAGATTCTGGAATTAAAAGATAGTATGAATGCTAA

AAGAGTATCATATGTTTTACCTCTAGGTCATGAGATAAGAATTACAGGTAATTATTTAGTGGGTTTACTAGAGGGGACGG

TTCATTTTATTTTAATAAACAAGATCCTACTGTTCGTGTCACCTTGGTTACTACCACGCCTAATAGAGTACTTTTAGAAA

AGATACGAGAGTTTCTTATAAGTCATTTAGACGAATATTCTGGTATGTTGGCTAGTTGCACGAAATTAATTAATATTCCA

TTCCTCCCTTCCTAAGGAAGGGGAGGATCCGCGATAAAAAATAAAAGGGGAGAATAAACCTATTTCATACCTCCCTCCGG

GAGGGGATCTCTGTTTTAGAAATTTATCAACTGGATTATATATGTAACATCCTAATACCTTATTTTGATAAAATACAATT

TAGAACTAAAAATCTCTGGACTATGTAGATTTTAGAACTTTAGCTTTTAACCTTACCCTCCCGGAGGGTATGGAATTGAG

GGTAAATATCTCACAGAAAAGGGTAAAGAATTAATGATTAAATTAGCAGATACTATGAATAATAACAGATTATCTACTAA

TTCTAAGCCCTTATCTTTAGATATGGCAACTAAGTCAGAATTAGATCTATTGATTAAATTAAAACCTTTAATAAATATAG

ATTCTGAAGGTAGAGCTATGATAAACATGAAAAGAAATATATAAGATCTTCCCTGGGGACCCCTTGGGTCCCCTTCGATC

ATCGGGGTCCCCTCGGGGACTTATATTATTAAAGCTATTTTTATAATGGTTCTATTTCTTATTTTACTAATGGGGTATCT

TGTGCTAAAGCTCTACATGTGAGTAACAACACTATAACACAGCGATTAAATGATGGTAAACCTATAAAAATAAAGAAGGT

TTAATATCAGCTCTAAGCGTAAAAGAATTAAAGTCTACTCTCCTCCTAAGTCTTCTGGCTAGAACTACAATACAACCGCC

TTAGTATAAACTATTAGCATATAGGTTTATACGTATTACTAACCTTATGAGCGCTATACCATGAATAGGACAAGATATTG

TGGAGTTTCTCTGAGGGGTTTCTCAGTTAACAACGCAACGTTAAACAGATTCTTTGCTTTACACTTTGTCTTGCCGTTCG

TATTAGCTGCTTTAGCTTTAATGCACTTAATCGCCTTACATGACACCGCGGGGTGGTTATTTACTGGCCCTAAATTTTTA

TATATGAGATCTACATCTACCTTACTTAAAGCAAGCTTAAGTTTTATATTACCTAATATTAAAGCCATAAAAGAATAGGT

CCACATAATCTAGATGTGATCTCGGTATTGGTTGGCTCATGGTTAGGTGATGGCTTTGGAGAGCGTGAAAAGAGTGGAGG

TGTAAGATTTAGATTTAGACAAAGTGTAGTACGTAAGGATTATATATTTTGGTTACAGAATTTTTACATACAAGAGGTTA

TTGTTCTAACCTTTTACCAGTTATTTACACACAAAAACAGGGGATAAAGTATTAGAGTATTATCGTTTTGGTACGTATAA

ATTTACAAGTTTATTGTGATTATATAAAACTTTTATAATCACAATAAGAAAAGGTAATACCCGCAAATATTGCGGATTTA

TTGACTCCTTTGGCCTTAGCTATTTTAATTATGGATGATGGTACCTGAAAAATCCAGGTGTTAGAATTGCAACTAATAGT

TTTACAAAAGAAGAGGTAGAATTATTATCATCAGCTTTATATACTAAATTTAATTTATATTGTTCATTACATAAAAATAA

TAGTAATTATCAATTATATATTAAACAGGAATCTATTACTTTATTAAAGGAATTGGTACTACCATATATGATTCCAAGTA

TGCACTACAAACTAGGAATATAAATATATAAAAGTAGTCGAGGGATTAAGTATTTAATCTCTAAGTCTCCGACGGAGACT

TAAATTATTATACCTCTCCTTTAGGTATAATGGTTTGGCAATGCTAGTGTAAACGGTTAATTTTTAATTAATTAATTAAG

GCGCCTTAATTAATGCAAGACCGTCGGTTATATTAATGTTTAAAATATAATCGCTACAGACTGCGTCACTGGTGGGTGTC

GATTTATTTGATGCTTAATGTACAGTCGAAATTTTACGCAGGTAATCCTTTAGGAGTATCAGGTAATTACGATAGATTAC

CTATGGCTCCATATTTCCTGCGAGCTTTATTGGGAAAATAGCTCATTTGTGGGTATAATTGCCAAACTCCGGGGACCCCT

TAAAGCTTTGGATACCAAGCTAGATTACTTACCTTGTTTATACCAGGCTTTGGTTATAGCCTGTAAGGTAGCCGAAAGGG

TCTAAGTGGACGGAGTAATTAGCCGTGTATGGTAATAAGTTTGAAGATTATTCAAAAATCTTTTGAAGAATAGGCTATCG

CGGATCTAAATCAGTATTTGCTTAAGCTTAGACTCTTTTGGGGGCTTGCTCCGCTAGCATCAATAATAAGTTTGTAGCTA

GTAGATTGTTAACCAAGTTGGCCTAATTTTTCATTTAGGTTAACGGGTAAATACTGTAAAAGAGCAACGAGTATACGGTG

GTTAATACAAACAGTACCCCAGGGTTATATTTTGCAATATAAGCAAAATAATGTGTTGTATTTAAGATGTATTCTACAGG

TTACACCTTTCTTGTCTAATGTTATGACAAGGAAGTTCGAAAGAAGCCTATAACCAGAATCCCTTCTTTAAATAAAAACT

TTAAACTCGGAGGGACACCCGGGGTGCTTTCTTTATTCCTTGTGGGAATCCCCGGATTCCTAAAAGGAATCCCTTGGGAT

TAAGTTAGTATATAGCCCTGGTTCAGAGGCTCCTTATTTGACATCAATGCCGACCAGGTACTCACCGTATCTTATTCCTC

TTTCCCCATGGGGAAGGAAGGATGAAAAAATTCATAGGTGTATGAGTTTATAGTAAGAAACGGGTGCCCGGGTGCGGGTG

CAGGTGCAGGTGCCGGGTGATACACATAATTTTTTTCACCCTGGAGCCTCTTTTCCGGAAAAGAGGCTCCGGAGGGAGGA

AAGCCAAATTAAATCATAAGACGGAGTCCGGAGTCGGGTTTTATACTTTTATTTAAACGATAAATTTGGTGGCCGTGATT

TAAAGATTTAATAACTATATTTTTATTTATTATTATTTTAAGTATCTTTGTATTCTTTATGTCTAATGTTTTAGGTGATT

CGGAGAATTACGTAATTAAGAGTGGTGTTTACTTTAGTTACTGTCAGAATGAACTTCTGGCAACAAACCATCAAATTGTC

GGGAAGTCCCTAAAGCTGTTTCAACCAAATAAGAATGGTAACATATCTTAAGGCACAGGTAATGACTCGTGGTTATTTTT

GGTACGAAGGTTTTATTTTTAACTTTAAATATTTCAAATAAGCAATATCCGGGGACCCCTAGGGCCTTCGGGGTCCCTCT

CCTCCGGGGAATACAAATAAAACCCCCAAATAATTAGGTAAAATCGAGACAGATTATCATAATATATGATAAATGGGTAA

TCCGCAGCCAAGTACCTCCAATCATTTTAGGTATGCAGTTCATCGACTAGACGTTGGTTGGCTTTTTTTAATTAGCTTAA

GATATAGTCAAGCCCCTCTCGAAAGAGGGCAGGAAATATTTATGATTTCCGTTAAATGGATAGTTTAAACCTATTCAGGG

ATAAACCCTTAGGGGTATCTGTTATTTATTTACTTTCTATACTTGTTTAAAGACTGAAAGTAATGACCTCTCTATATCTC

AGAAGGAATCTACACTGATTGAACCTGATAGGGTTAAACCGTTGCGCTCTCCCTCTACGAGGGAGGGAGTACTAAAGGTA

AATTAGGTAATAAAGAAATAAGCCTTTCGCTTGTAAAGGCTTGATTCTTTCCATGAAAAGAATTTTTCTCAAAATTGTAC

AGGTAAAGGTACAGATAAAGAATTCTGTATGCTTGCAAACGGCTTTTGACAAGCGGAAGGTTACATAGGTGGTATTTTTA

GACAAGGGTTAAATTTTTATCCTCTTTGTACAGCTACTCAGTTGTTATCGGAACAAAGTGTACTTTTTCTCCTCAGATTA

GATAAGTCTTTATCTAATAAAGGTACTTTTAATATATCTTTGAATAGTATGGGAAAATTTGTTATAATGTATAGATTGTC

AGGTTGAGATACATTTTTTCTATATTTGTTCCATACTTTTATATGCTTTATGGAGCAAAGTATCAAGCGATATATAAGTT

AAAAAAATTTATCAATTAAAGAATTTAATAAAAAAATTCAGATGATATCTATAAAGTCTTGTTGATAAGCATTGCTTATT

CCTTGACTGCTCATTCTTCTCGTTATAAATTAAGTATTCAAGATAAATTAGTTTCCCTTAACTTAGATCCAGTTTTATTG

AAAAAGTACCTTTGCGGGGATCCTGGAAGGTCCCTTCAGGGACTTTAAAGGGAGATGGCTAGCCGCCCCAGGGGCGGCAG

GTTCCCTCGGACCAGTTCCTGGTCCCCAAAGGAACTAGTATTAATATTCCTGAAAATAATATTTCTCCTTCTTTTCTCTT

TATTTTGGGATTCTTTTAGGTGACGGTACTTTACATTTAAAATTGGAGTGAAAACCCCAAAACAGTACTGTAGTTATTAT

TCCTTTATTTAATATTCTACAATTAAATGTTGAATCTAATAAGCAAATAATGGAAATTATGACTAACACTTTAAATACTA

TGGGTATAAAGACTTCTTTAGTTAAAAGTACTAAAACTTTAACTTTAACTGTTAAAGGTATAGATAATGTTTTTAAATCT

TTATTACCATTATTGAAAAATACTCGCACTTTTATATTGAAAAGCGATAGTTTTAACTTATTAGTCTGAGTAAAACAGTT

GGTTAAGTCAGGAGGCCACCATACTTATTTTGGACTTAATGCTCTAATTGATAAAATATATAGTAGTGTTAATAAGCGGT

TTACAGACAAGGAAGTTTGAATTAGTCGTTTAAATATTTGACTAAAGGCAGTTTCCGACCGAAGAGATTGAGGAGAGTAT

TATATCTGCCCCATATATACAAATCTAAGGGGAGGGAATGAAATAAAGTTATTAGAGGTTGACAGGTGCGTTTTCCTTCT

ACATTAAAACTACCGAAGTCCAATAAGGCGTTTATATGTTCAACTTTAGGAGGACAAGATAAAGCTCTTCAATTAGCTGT

AGAATATAGGGATAAAATTCTATCAGATTGAATTAATACTTTTAATCTAGAACCTTTGCAATTATTTCCTTCCGTCTTTT

AGGATTTTAAGAGAGCTCGGCCTAAGGGCTAGCCAGCCCGAAAGGGGGTAGAGGTAGAGGTTACAATAATTATATATTTC

CCTTGGGGATAACTGGGCAAATCCAATGCAAACACCACCAGCTATTGTACCTGAGTGATAGAAAAAGATGTCACTTAATC

TAGCTATATGCAGGAAACTCCTAAAACTTTAAGTACTTCCCTCTTCGAGGGAGGGTAGCCCCAGGGCTTACACCCATTGG

TGGAAGAAAACCAGAGTAAAAATCTTAAAGATATTACAATGGACAATCCGCAGGAAATCTACCGGTATATCTGTTTAGCC

ATGACTACGTAAAGCTTTTCCTCCTACCTCGTTCCTACAGCCTCTTCGAGGCAGGAGGGTGTAAATGAGGCAGGAGGGTG

TAACATGGTATGCCTTAATAGTATCCTTAGAGGCTATACGTGAGACATTATAAGGTAAACATTAATATCTTATAAATGAA

GATTTAGTCCGATTATTATAGTAATATAATGTTTAGAAACATCTATTAGTACCTTTACCGGGGAAGCCAGGTATTGTAAC

GTTTGGATAAAAGCATGTCCAGAAAATCACACCTTAAATATGTTTCTACAATTAACACGATTTTATATTTACAATACTTT

GAGTGTCGGCTAGCTTAGTTTTATATGCAGCCCGCTCTACCAAAGGTATTTTTCGTTTATCTTCCGCTGAAAGAGCTTTA

ATGATAATATCTGATGATTTAAAAGAAGTATTAATAGGTATACTACTCGGTGATGGCCACATAGTAAAAGATCCTCTACA

GCTAATTCTAGATTGGTGTATGCTCAAACAGCAGTAGCACATAAAGAGTATTTTGATTATGTATATAGTTTTTAAAGACT

TATGTGTTAATGATTACATACCCCAATTAAGAATAGTTAGAGATAATAGAACTAATAAAATATATAGTGCTATATCTTTC

ACCACTATGCAACTTCCCTGTTTTAATGTATTTAAAGAAATGTTTTATCAAAATAATGTAAAAATAGTTCCAGATAATAT

TCACCAGCTTTTAACTCCTAAAGGATTAGCTTTTGAATTATGGACGATGGCAGTCGTCATGGTACGGGATTACATATAAG

TGTTTATGCCTTTTCCAATGAAGACGTAGATAAACTAATGTTTACATTACAAGATAAATTTGCCCTTAGATGTTCAATAC

ATTACAACAGAGATAAAACCTAGAATATACGTTTTAAAGAATCTATGGATAGATTAAGATCCTTAGTAAGTCCATATTTT

ATTAAAGAAATGTTATATAAACTAGGTTTATAAAGCTGTAACTACATAAGAGCAATTATGGTCCCCCTTTTTTAGTTCCC

TACCGAGGGCGGTAGAAAAAGAATTTTTATTCATCCCCTTTTGGGGTAGTTCCTCCGGAAGGATAAAAAAGAACTTCCGA

GGGTCCCTCCCGGTGGAAGGGATTAGTATTAACTTGTCTCTCCCAATTATGTTTGTTAATTTTTTGATCTTTTACCTTTC

TATGCTATTTTAAGAAGTATACCTAACAAACTATTAGGTGTTATTGCAATGTTTAGTGCGATTTTAGCTTTATTGGCTAT

GCCTTTTACAGATTTAAGTAGAAGTAGAGGTATACAATTCAAACCTTTAAATAAAGCAGCTTTCTATATATTTATAGGTA

ATTTTGTTATATTAATGGTTTTAGGTGCAAAACATGTTGAATCGCCCTATATTGAGTTTGGACAAATTAGTACACTTTTA

TATTTTTCACATTTTTATTAATAGTTCCTTTAGTTAATTTTTAGAAAATACTTTAATTGTATTATCTTTGAGATCTAGCA

ATAAAGAGGGTCCTATTTATAAAGAAGGTGGGGAAAAATAAATTCCTCGGCGAAGCAACCAGCTGGCCTTTAGCGAATGC

CTAGGGATTCCTTCCTGTGCTGTGCTGTGCTGTGCTGTGACTTGAGGGGGATAAGGGCTTTTAGCCTAAAGCCTTCTTAT

ATAAGCCTTTAACGTGGCTTACGTTCCACTCGTATATTATGCTCGCGAGTTTGGGGACGGCCAGCAACTTGTTTTGGGGG

TCTTTAGGAACAGGCGCCTTACTAGTCCCCTCATCCCGGGGATACCTCCTGTCCTAATCGGGGATTACGGGAGGCGCTTC

TGGTTCCGGCCTCTGCTAATCCCTTAGCATCCTTTAAGCCTTTACTTTAGGCGGCTCCTCTTAGGGGATACCCCCACGGA

TGGCACAAGGCTAAGGCTACGGCAGCAGGGGATTCCGTACGACGACGCCCTGTTTCCCAGCCTGGGGGTGGTAGGGATGT

AAAACTTTGGAGGGACGGCCAGGAAACCGCAGGCGTGCCCAAAACAAGTCCCCAGCGCGCCTGCGGCTGCTAGGGGATCA

ACCCCTTAGTATCCGCGCCCATCGGCTGCTATCGATCGATCGATCGGGATCGATCGGATCGATCGGATCGATCGATCGAT

CGATCGATCGGATCGATCGGGGATTGGATTGATCGGGGGATTAGGGGAAGTTCTGGTTCCGGGCTCGAGGCGGAGGGACA

AGGCATCCCCGCTACGCAGCGGGGATTACGCCCTTTCCTAATCCCTTAGCACGGGGGATAAGCAAGCGGTGAGCTCAAAA

CTTTGGAGGGACGGCCAGCAAACCGCAGGCGTCCCCAAAACAAGTCCATGCTCAATCCGCGCAGGGGATACGGGAGGAGT

ATACCCCTGCAGGGGATTAGAGGAAGCCGCTAGAGGTTAGGGCTCGAGGCGGAGGGACGGGAGGGATCCGCCGCCTTAGG

GGAGGGACCCTGCAAGCAAGCGGGGGTTCCTCCTCAAAACTTTGGAGGGACGCCCAGCCAAACCAGTTGCTGGGGGTCCC

CACCAAAACTACTCCAATTAGTCCCGCCGCAACTTGTTCCTCTCCCTGAAGGGGAGGGTTTTAATTTATTAAAACCCTCT

CCCCGGAGGAGCCCGGAGGGTTTTTTCCTGTAAAAAACCCTCCTCCCCTCTTTTCCGGGGAAATAACTTTTATTTTATTT

TTAATCCCCGGAGGGATTCCTAAAAGGAATCCCTCAGGGGATTCCGCAAGGGAATAAAAGTCGCCTTCGGATTAATTAAA

AATTTTATGAAAAATCTCGTGATTTGGAGATTTTGCAAAGCCAAAATTAAAAAGAAAAGGGGGATAATCATCGTTCTTCG

ATAGCCGATCGGGGGTAGATCCTAGGAGAGGGAACTAGTTTAGCTAGGGGCAAAACTTTTTGTCCCTCAGGTCCCTTCCT

CTCCTTTGAGAGGAAGGAAAAGCCCGGGGGTTTGAACCAAAAATCTAAAAGAAAGAAGCAAAATAAAATTTGGTTTTGCT

TTTAATTTTTCAAATAAATTATTATTTCTTTTAAAATCTCTTTTTTATCCCCGGCCTCCACCCCCGATCGATCGGGGGTG

AGGGAATAAAATAGGGATTTCAATAAGTTCCAAACCCCTTTGGGGTTTGGCATCCTGTGCTGTTCTCTCCTTCGGAGAGG

GATGCCCAGAGGAACGAAACCTAGTCCCTTCCTTCCAATCCCCGATGATGATCGGAGGGGATTGGAAAGGGAAGGGATAA

GTCCCTCCCGGGAAATCGGGGACTAGTTTTGAGGACAACGGGAATTAACTCTTTTTGTCCCTCAAAACCAGTGGGTTTCG

TGAGGGAGCCACAAAACCACAAGTTGCCCCGATTCTCCTTGGGGATCTCCGTCCCCTCGGGAGAAAGAACTTTATTTTAA

TCCCTCCGGGATTCCATTCCTATTCCTCCTAAGGGGAGGAATGAAATAGGGGAATCCTTAGGGGCTTGATTCCGCAAGGG

AATAAAGTCTTTTGGAATTTTGCTTTTGCAAAAATCTCCAAATAGCCAAACTTCCCGATCGATTTCGGGGAAGGAAGGGG

AGGGTTTTCTTTTCCCTCGGGAAAAGGACTTTTATTTTAATCCATTCCTTTTAGGGGAATCCTTAGGGGATTCCGCCGCA

AGGAATCAAATTTGGGAATTTTGCTTTGCAAAAATCTCCAAATAAAAGCCAAAACCCCGATTATTTTGGGGGTTTTTTAC

GGGGATTTTTCATAGAAGTCGGGCTTTTTAATTAAAATACTTTTATTCCCTTCGGGGAATCCCTGAGGGATTCCTAAAGG

AATTCGGGGATTAAAAATCAAATAAAAGTTATTTCCCGAGGGGAAAAGAAAAGGGGAGAGGAACAAGTTACGGGCCGGGA

CTAGTTTTGTGGGACAAAACTTGTTTTGTCCCTCAAAACCATAAGTTTTGAGGGAGCCACCTCGGCGATGAAAACACGAG

TCCGATCAATGAGGGATTGGGATAAGTCCTCTCCGAGGGACTAGTGTTTTGCAATATGGGGAAGGTTTTCCCTCCTCAAA

ACAACAAGAAGTTCAAACCCCTTTGGATCGATCGATCGATTGGTTTATGGCATCCTCTCCTGCTGCGGAGAGGGATGCAG

AGGAACGAAAAACTAGTCGGGGACTTCCATTTCCCTCGGGACCAGTTCCTGGCCCTCAGGAACTAGTTTTTTCAAAACTT

CTGGTTTTGAAAACAAACAAGTTGGGGTGTTTCCTGCAACTAGGTCCTCCCTTCCCAATCCGCGGCTCATCGGCGCGGAT

AGGGAAGGGAAGGGATGTCCTGGACTAGTCCCCCAATTGGGGACGAAAATCATGTTCCCACCTCGGGACCAGTTCCTGGG

GGCCGGAACGAGTTTTTTAAAACTTCTGGTTTTGAAAACAAACAAGTTGGTGTTTCCTGCAAACTAGTCCGGATCCGCCA

CCATTCTTCGATTGATCAGGGGATTGGGAGAAGTCCTCTCCGAGGGACTAGTTTTTGTGGGACAAGTAACTTAACTTTTT

TAGTTAGTTGTTGTCCCTCAAAACCATAAGTTAAAATCCCAAAGGGGTTTGGCATCCTCTCCTGCTGCGGAGAGGGATGC

AGAGGAACGAAAACTAGTCGGCTCCCGCAATCGAAGGGGATTAGGAGGAGGGATACCTCCTCCGATAGGGGATCGGGATT

AGGAGGCGGCAGCTTCTTCCGGGCTCTGCAGCTAATCCCCTCTAATCCCTGCCTTTAGGCTGGGGATACCGAGGGATGGG

ACGCCCAGAAGGTCCCTCGGCTAAATTTCCCGAGGAAGCGCCCTAGAGGTCCTCCTTCATGTTCATGTTTTTAAGCAGGG

AGCCATAGGGAGGAGTGGAAAGGGGAAATACGGGGGACAAGCCTAGCTAGGCGGGTACCCCTCTAACTTAAGTGCATAAA

GGGGAAAGGAGAACCGCTGCCAGCAGGGGGTTTCTCCGGGTTTCCTTTTCCTATCCCCTTCTAGGTAGGCGTATCCCGGC

CTTAGGGATCATCCTTAGCGCCTGCGGCGCTAAGGGGATAAGCAGGGTCCCTTTACTTGTTAAGAAGGGGTCCCTTTACT

TCTTCGAAGAAGAGGGCAAGGGGATTAGAGGAAGGTTTTAAGGCTCTAATCCGTGGCTGCCCTAGCAAAGCCTGCGGAAG

GAAGGGGAATTTCGCTCTTATTCCCTCCCTTCCTTCCGCAGGCTTTTCCCTGGAGGTTTTGGGGAGGTCGGGATTGATGA

ACTTCAGGGGGTTAGAACCTATCTTTCTAGTCCCTCGCCTTAGCCCGGGGATACCGGGGATTAGGCTGAGGCGCTTCTGG

TTACGCTCGAGGGCTTCTTGGGGACAATTGGAGTCCCTCGGAGAGGGAATAGCGAAAACCTTTTTAGGGGGACTTAATTT

GCTTGGACTCTTACAAGGATAAATAAATGAAACAAATGCCGCTTATGACGAAGGTGGCTCTATAACGTCATTGCTTAGCC

TGAGTAATGCTTATCTCTTCTTCGAATAAAAGTGGAGCCAGCAAGGGGATTGTGGGATTCATTTTAAAATCGATCCTGGG

TGTGATTAGGAGGGAGATACTTGTCGAATAAAGAAGTGTAATGAATCAATGTGCCAAAAATACTGAGAAAATAAGTTCTT

CTGCCCAGGGGAAATTAGGATCCTATCTTAAATGAAATACGTAATCCCATAGCAAATTAGGATCCCTCAATTACCTAATT

CCACCTTGTTTTCTACATCGACTAAAGAGGTATTTACTAGTATTTCTCATTTAGGCCTTAATACATAAGAGGAATGGACA

GGGGTAATAAAGACCGTATCTAGGGTTGGGGAAAATAAAGGGGATAATTCTCTAACGGGTAAGGAACCAGGGGAATTTTG

CGTTATCACTCGGTTATCTAGCACTCTCTTCGCGATCCTTTACTTGTTTCCCTGGGGGTAGCTCAAAAGTTTGAGGGACG

CACCGCAAACCGCAGGCAGGCGTCCCCAAAACGCAGGCGTCCCTCATCCCAGATAGTTTTTCAAAACTTGTTTTGAAAAC

AAACAAGTTTGTTTCCTGCAAACTAGCGCGCCCGGATTAGGGGAGGAACTAGTTCCCTCGGGAAAAAGCTTTTTAGCCGA

GGGGACCTCCCTTAGGCAGGGATTAGGCGCCTTCCCTGCAGGCTTCCTCAAAACTTTGAGGGACCCTGCAAACCAGGGGT

GTCCCAAAACAAGTCCCTCGCGGCAGCTGCAGCCCGGTAGGGTTCCGCCTTAGGGTGGGGATTAGGAAGCGGAACTTCTT

CCTAATCCCTAGGGGATAAGCGGAGGGAGGGAGAAGGATCCCTTAGCAGGGGACTAGTGATTTTCGTTCCTCAACGTAAC

TTTTTGAGGGACGGCCAAACCAGTCAGTGCAGGGGGTCCCACCAAGTCCCTCCAATTAGCCCGGGGATACCCCGGGATTA

GGGAAGCGGGAACTAAGGGGTTCGGTCGAGCGAGGGGGACGGCCTGCTTGCTAGGCGGGCTGCAGCTGCTGCTGCTGCAG

CTGCTGCAGCTGCAGCAGCTGCTGCTGCAGCAGCTGCTGCTAAGGATCCCTGGGATTACTAGTCCCAATCGCGGGGATAC

CCCGGATTAGGGGCCAGGCCTTACTTGTTCCCTCGGTAAATTTAGCCGGAGGGGACAAGCTCCCTCCGGCGAAAAAATTT

TTTCCCGAGGGGAACTATCCTTTAAATTTATTAACCGAGATGAATAGTTTAATTATTAGTGCCTATCCCGCCTTCGCGGC

GCGGGGATTAGGAACTTAAAGCTCTGCCTAATCCCGCCTTAGCATCCCGCTAGGCTTATCCCAGCCTGGAGGGGCCTAAG

GGGATGCATAGGGGATTTGCCGCCTTAGCCTGGGATGGGACGGGCAGCGCGGCTCCCTCGTAGAAGTGCTCTTATATTTA

TATATATTATCCGGAGGGGATTTTAATATATAACTAAATATTCCCGGGGATACCGGGGATAATTAGTAAAGATTTCGTTA

AATATATTCAATCTTCTATACAAAATCAGATGATCGCTAAATTATTTCTTTTTTATTAAATCCTCCAAGTTGCAATGCAA

TTATAGAGGGATTAGCCCTTTGGTTTATTTAGCTAAAGTAATGGGGATTTTTTTTTTTTATGTGTAAAGTGTTATCCCAT

ATTGTCTAAGGGGAAAGAAGTAGGGGTAAGATCTAAAATTCTCTGCCAGCTAATTCCCGTGCTCGTGGGGTTTGGGTAAA

GACTTGTTCCTCTTATCCCCTAAAGGGGATTACAGGGAGGGACCGGCGTCCCTAGGAGAGGGAACCACTTGCGCTTCCTA

ATCCGGAGGGGTATCCCGGGATGAGGGGACTAGTTTTAGGACAAAATTCCAAAATCTCGTTATTTTGCTGAGGGGAAGGA

AACCATCCCTCTGGCGATCGGGGATCCGGGGACAAGTCCCTCTACGAGGGAACTAGTGTTTTGCAAATTGGGGCAAAACT

TGGGGGTTTTGCCCTCTGCCTGCGTCGTCCATCTCTCCTTCGGAGAGGGACGATGCAAACCCCAAAGGGGTTTGAAAAGA

GTTCGTTGAGGCACGAAAACTAGTCCCGCAGGAATCGGCGAACTTGTTCCTCTCCCCGAAGGGGAGGGTTTTAATTTTGC

TGCTTTGCAGCAAAATATCCAAACCTCTCCCGAGGGGAGGGTTTAACCTCTCCCCGGGGAGGGTTTTACCTCTCCCTTCG

GGGAGGAGGGTTTAAAAACCCTCGGGCTCCCTTTCTTTTAATTCCAAAATTTTCTTAAAATCAAGTAAAAAACCCTAATT

TTTTGCTTTTTATTTCCCCGGGAAGGAAACCATCCCTCTGGCGATCGGGGATCCGGGGACAATAATTGGAGTCCCTCGGA

GAGGGAACGAGTTTTGCAGGGACAACGGTAATTAACTCTTTTCTTGTCCCTCATCAAAACAAGAAAAGTTCGTTGAGGGA

GCACCTCGACGACGAAAAACCACTAGTCCTATCCTCTGGGGATTAAGGGGAGGAACTTGTTCCGGCCTCCAAATTGGATC

GGGGGATAATCATCGTTCTTCGATCTAAGGGGGTAGATGGAGAGGGAACTAGGTGGTGGTGGTTTCGTTCCTTGCATCCC

TTCCTCTCCTTTGGAGAGGAAGGATGCTAACAACCCCATAGGGGTTTTAACTCTTGTTGTTTTAGGGACAAAGTTTTGTC

CCACAAAACTAGTTTTGCGTTCCTCAAACTCTCGATTTATCGGGGGTGGTTTGAGGGACAAAACTCTCGATTTATCGGGG

GTTCGTGGCCCACAATTTTCGTTCCTCAAACTTAAATCCCTCCGGGGATATTTTTCTGCTTTGCAGCAAAAATTAAAAAA

TAAAAATTCTTTTATTTTTCTATTTTGGCGGAGCCCAAATTAAAACCCTTCCTTCCCTTCCCCGAAGGGAAGGGAAGGAT

TTTCTACAAAAATCTCTTTTGCAAAATCCCCAAAAAAGAATTTTTTATATTTTGCAAAAATCCCAAAAATCTTTTTTATA

TTTTTTTGAGGGACAAAAGAGTGAGTTTTGTCCCACAAAACCACCAAGTCGAGGAGGGAGCCTTGTTCTTGTTCCGCTCA

GGGGTTTTGCGGGTAGGGGTAATCCTTGGTTTATCCTGGCTTCCCTTTCGGGGAAGGGAAGGGAGGGGGGGGGTATCCGT

ATCCCTTATCCCGTATCCGTATCCGTATCCCTATGGTGGCGTAGCTTAGGGGAGGGATGGCTAAGGGGATGCTAAGGGGG

ATAAGGGATACGGGGATGGCTAGGCTTATCCTCTGCAGGGCCGCCTAAGGCGGCCGGGATTAGGGGATCCTCTAAGAGGG

GATTAGGCGTAGCGGGGAAATTAAAAAATCCCAATTAAATGGCTGCAGCAGGGGTTCGCTAGGGTAAAGGGGTATCCCTA

CTTTCTTTGCCAAATTAAAACCCTAATTTTTTAATATTTGCCGGGGAGAAATTAATTAATACCCCTAATTTTGGAATATT

TGCCCGGGGATAAATTCATTTAAATGCTGCTGCTGCAGCATCCCAGGGGATTGGCTAATCGGGGGTAGGATCGTTTTGGC

TCTTTATAAGATTTAGACAATTTAATTTAAAAATTATTTCCCAGTTTTATGTTACTAAAAATGTTATTATTAACACCTTT

ATTAGGAATATTTTTAATAACTATTAGCAGATATTTTGCCTTAAAGTTTGAAAAATAATTGCCTTAATTACAACTATTAC

TAATTTGCTAGTTTCATTGGTTATTTTTATTTTATTTGATTTTAGCATTAATGAATTCGCCTTTGTTCAAGAGTACCATA

ATCTTAGTTCTTATGATTTATATTTAGGTATAGATGGAATATCTATATATTTTGTTGTGCGCCGTTTAATTAATGTGAAG

CCCACCCCACTAAGGGTCATAACTAGGTCCAACGTTATGTAAACCGACTGACTTACCTGAAATGGAAACACGTAGGGGAC

CAGAGCATGTCAGTAAAGCTCTCAAAAGGACTAACTGCAAGTTCTTAGAGAGCAAGGTAATGTCTATTCAGGGTATATCT

GTAGTGTTATCGCAGGACTATATACTGGTTTCGAAAGCAAGTGGAACGAAATGTGACAATAATTTCATAAAAAGCGAAGT

ATTAAATTAGACCTACTGTCATCGATATGGAGAACATCTCTAAAACACACACCTAAAAATAGATTAACAGACTTCGTATT

AAGAACTCGGGATCTCCTAAGTTTAGGAATAAATATCCATATCTTAAGACTATGGACCCCAAAGGGGAGACGGAGTGATC

GTAGTACCAGAATACTGGGAAGGGTCACAGGATTCAGTAAGTAATATGACAAGAACAGGTAAGGTAGAAACGACCTTACA

TTCCTGTCCGCTATTAATCGCAAGAAAACTTAGAGACAGACTAAGCTACAATTACTGCTCAAGATAACTTAACTCCGTAG

GGGTCTCTAGGCAAAGCTTTAACTAAAATTTACATTTATAAAACCCTATTACATAATGAAGTCCTCACCCAGGAAATATG

AGGGAGGTTAAGAAGACAGATTAACGCTTGATGAGATGTCCACAAAACTAAATCCCTAAACTTATGACAAGCAAGCAAGC

CAGGGCAAGCAAGCCAGGGAAAGCAGCCAGGGGAAGCCAGGCAAGCCAAGCAGCCAGGGAAGCCAGGGGAAGCCAGGGGA

AGCCAGGGGAAGCCAGGGGAAGCCAGGGGAAGCCAGGGGAAGCAAGCTAGGGGCCAAGCAAGCCAGGCAAGCCAAGGAAG

CAAAGGGGAAGCAAGCAAGCCCTCAGTGTTATTAGTTGAGAGTATCAATGTAAACCAGTCAGAAGAGTCTACATTCCAAA

ACCTCAAGGTAAAATGAGAGTGGGTATACCTTGTCCCCTTCGGGGATACCGGGGTATATCAATGATAAAATACTTGTTCC

CTCCCTCGAGCCGGAGGGACCTAGTCCCTCCCTCGCTTATCCCTAGAGGATTCGGAAGAAGCCGCGGGCTTCCTAATCGG

GGGTATCCCCGATTGGGACTACAAACTGCTTGCAAACTTAATTTCTCGGGGAGAATAGAACCGGTATGTCTTAGATTATT

CCATAAGAATAGCTTCGGATTAAGACCCTATAAATCTGTTCATCATGCGCTTGCGGTCAGAGGGGATGGGTAGGTATAAC

TTGAATGATAGAAGGTGATATCAAGGGGATACTTCGATCCCACATAGATCATAAAATCTTAAATCCCTCACCAAACGGGG

GTTTCGTGAGGAATAAATAAGATTGTCGATAAAAGTTGAGCCTTATCGTACTCTTAAAGGGGTATTCAATAAATGAATTA

CTTTTAAGCAGTATACATGGAAGACAGGTAGGTTTGTACACTCTATTCTGGGAGTAGCACAAGGGGAATTATTTCCCTAT

CCAGTCGAATTTCTATTTTACAGCTTTTACTAATTTCTGGATGATTATGGAGTTAATGAGGTACTACCGTGTCTACTATA

AATCTCTAATATTTGTTGATTGAAGCTAGAATATGGACTTTAAAACTTAAGTGATGAAAAACTAAAAATGTAGAGCCGCT

TGATAAATCGAGGCAGGGTTAATAGAGGAATTCCAGGGACTAAAAACTTGTTCCCTCTCCAATCGGAGGGACCTTGTCCC

ATCCCTATCCCAATGGGGATTCGGGAGCCCGCGGTATCCCCTTTGGGGTCTTTTCAGCGCAGAGAGGAGGCGAAACCTGC

CCGGTCACCTGGCGGGGAAAGGGGATCCCCTTTTGTGTAATTTTTTTCGGGGAAAAAGGCGGGGATTAGACTAGTTTTCG

TTCCTCAATCCCTTCGGGGATAAAAAATATCTTTATTTTAGGGACAAAAAGTTCGTTGTCCCACAAAACTAGTCCCCGAT

GATCGCCGCCGGAGGCGCAACTTGTTCCTCTCCCCGCCTTAGGCCGGAGGGTTTTTCCTGTAAAAACCCTCGGGGCCCCG

ATTAAGGGGTTTTAATTTTCTCCGCCAAAATTAACAGGAAAACTCCGGAAGGGGAGAGGGTTTTTCCTGTTCCTGTAAAA

ACCACCCTCAGGCTCCCCGATCGATTAAGGGGTTTTAACTTTGCTCCGCCAAAATTAACAGGAAAAACTCCGGAAGGGCG

GGAGAGGGTTTAAAAACCACCCGGGCTCCCCGATGGCCGAAGGGGTTTTAATTTTCTCTGCCAAAATTCACAGGAAAAAC

TCCGTAAGGGCGGCAGAGGGTTAAAAAACCCTCCTCCCCTTTCTTTTAATTCAAAAGACTTCTGATTAAAAATCGTAAGT

CGTATTTTATATTTTGCAAAGCAAAAATTAAAAGATTTTATTCCCTTGCGGGGTTGAATCCTAAGGTTAATATTCCATTG

CCTTCCGGGCCTTTTTAGGAATGGAATTCGGGGATTAAAAAGAAAGTTTGCTCCGTTATTTTCCCGAAGGGAAAAGAAAA

GGGGATGGGATCGGGGATAATCTATTGGAGTAGATCCCTCGGAGAGGAACTAGTTCCCTCCCTCCTATTATCCCAGGGGA

TTACAGGGAGGGACTTATCCCTTGGAGAGCCCTCCGGTGGGAATAAGTCTGGCGCTTAAGGCTCCGAGTTCGCGCGGGCG

GGACTAGGTTTTGCGTTCCTCAAAAAAATCTAAACAAAAATTTTTTTTTATTTTGGGCAAAAATATATTGGTTTATCCCG

GAGGGATTTAACTTTGAGGGACGGGGAGTAGTTTTGTCCCTCCCTCGGTAAATTTAGCCGGCGGAGGGGACTTATCCCTC

CGCTGTAATCCCTTTAGGGGATAAGAGCGGAAGAAGTTCCCTCCCCAGGGAGCCGGACTAGGTTTTCGTTCCTCAAACTT

AAATCCTCCGGGGATAAAAAAGAGATTTTTTGAGGGACCAAAGGCGGGTGTTTCGTGGCCCACAAAACTCGAAATAAGGG

GAGGACTTAAAAATAATTCTCCTCCTGGTTAAAGCAATCCACAGGGCCAAAGCTAAACTATCGGTTCCTATAAAGGATCT

TAAAGAAAAGTAGATAAAAAGTTCTTTTGCTGCAGACTCAAACGGAAACCTAAATCCGAGGGAAATTTCGGTTTCTTAGT

TCTCCGAGGGACTCCAATCGTCCCCTTTTCTTTCTCCCCTGGGGAGAAATAACTTTTATTTTGAATCCCCGGATTCCTTT

TAGGAATCCCTCAGGGGATTCCCGCGAAGGAATAAAAGTCTTTTAATTTGGTTTTTTGCTTTGCAAAATCTAAAATAGCC

GCCAAAACCCTCCCTTCGGGGAAGGGAAGGGGAGGGTTTTAATTAGGGGCTTCGCCAAAATACTAAGGGGATTTTTAATC

AAAAGTCTTTGGGGAATTTGGTGGTTTTTGCTTTGCAAAAATCTCAAAAGACGATTTTTGAATAAAAGTCTTTTTAATTA

AAAGAAAGGGGAGAGCCGGAACAAGTTGCGGCTTCCTAATCCCATAGGGGATAGGGACTAGTGGTTTTCGTTCCGCCTCA

ACGAACTTGTTCTTGTTTTAGGGGGCCAAAACGGGTTTCGTGCCCCAATTTGCAAAACACTAGTTCCCTCTCCCGAGGGA

CTCCGATCGATGATTGTCCCCTCTTTTCTCCCCTCGAGAAATAACTTTATTTTTAATCCCCGTAGGGATTCCTAAAAAGG

GGAATCATTCACCTTACGGGATTCCGCAAGGGAATAAAAGTCTTTTGAATTTTTGCTTTTGCAAAAATCTAAAAATACGA

CTTAGATTTTTAATCGTAAAAGGCTTTTTAATTAAAACCCCCAAAAAGGGGAGAGGGAACAAGTTGGGGGCTTCCTGCAG

CGATCGGGGACTAGTTTTAGGAGGACAACGAACTCTTTTCTTGTCCCTCATCAAAACAACGGGTTTCGTGAGGAACGCAA

ACTAGTCCCTTCCTTCCCAATCCCCGATGTCAATCGGGGATTGGAAAGGGGAAGGGATAAGTCCCTCCGGGAAATCGAAA

TCGGGGGACTAGTTTTGAGGACAACGGGAATTAACTCTTTTTGTCCCTCAAAACCAGAGGGGTTTCGTGAGGAACGAAAA

CTAGGGGGTTCCCTCTCCTTGGGGATCTCCGTCCCCTTTTCTCCCCTCGGGGAGAAAGAACTTTATTTTAATCCCCGGAG

GGATTCCTAAAAAGGGGAATCCTTCACCTTACGGGATTCCGCAAGGGAATAAAGTCTTTTGGAATTTTGCTTTTGCAAAA

ATCTCCAAATAGCCAAACTTCCCGATCGATTTCGGGGAAGGAAGGGGAGGGTTTTTCTTTTCCCTCGGGAAAAGGACTTT

TATTTTAATCCATTCCTTTTAGGGGAATCCTTAGGGGATTCCGCCGCAAGGGAATCAAATTTGGTAATTTTGCTTTGCAA

AAATCTCCAAATAAAAGCCAAAACCCCGATTATTTTGGGGGTTTTACGGGGATTTTTCATAGAAGTCGGGCTTTTTAATT

AAAATACTTTTATTCCCTTCGGGAATCCCTGAGGGATTCCTAAAGGAATTCGGGGATTAAAAATCAAATAAAAGTTATTT

CCCGAGGGGAAAAGAAAAGGGGAGAGGAACAAGTTACGCCCGCGGGACTAGTTTTAGGACAAAACTTGGGGGTTTTGTCC

CTCAAAACAAGAAATAAGTTCAAACCTTTGGGGGTTTGGCATCCAGTGCTGTTCTCTCCTTCGGAGAGGGATGCAGAGGA

ACGCAACTAGTCCCTTCCTTCCCAATCCCCGATTGATTCGGGGATAATCGGGGATTGGCGAGGGAGGGATAAGTCCCTCC

CGGGAGGGACTAGTTTTGTGGGACACGAAACCCGTTTTGGTCCCTCAAAACAATAAATAAGTTCAAACCCAAAGGGGTTT

GGCATCCAGTGCTGTTCTCTCCTTCGGAGAGGGATGCAGAGGAACGAAAACCTAGTCCCTCCGGGACTTATCCCGATCGC

GCCAGATCGGATCGGGGCGGCTATCGGATCGATCGGATCGATCCGATCGGATCGATCGGATCGATCGGGGATTGGGACTA

CTATGAAATAGTAAATCCTTGAAAGAACAGTGTAATGCATCATCCCTTAGCAGGGAACTAACTAAAAGAGGAATTCTTAA

CTTCTATAATATGGCGTAAATTCGTTATTTTTAATATCTAGCTGAGACAGTAGTAGTTTATATACTATAATCCTCCTTAG

CTCACACATAGGGAGCTAACCTAGAATGAGCCTAGCCAAAGTCTGAAGCAAATTCGGAAACCGGTTAAAGTCACCATCTC

TAAGGATAAGAAGATAAAAGAAGGGGAAATTTTATCCCCTAAAGCCGACCTACAGCAGTCTTGCAGCCTACCAAGATACA

AAATATGCTAAGTATAGCTGTCCGAATGAGTTAAAAGACCCTTTACAACCTTTAATTGAGGAGATATTAAGGAAGTGAAT

ATACTGGATCAACATGTCTAATATGTGAATCTAAAGAGAATGTAGAAATGCACATTACACCTTATCCCAAAGGGGATTTA

TTATTCCCTATCCCTTTGGGAATCCTCTTCGAAGATAGGATTCCCCGAAGGGAAAGTTTGAGCAAAGATAAATCAACTCT

CCCTCACTATCCCCTTTAGGGGGCTTATCATTAAGATTCTGAGTAAGATCCACTGAAAAGTCGTAGCACTCTGTAAATCA

TGTCCCATCGGGTTTAGTTCAAGCTAAATATGGGATGGTCTTAATTTGAGGGAGATTCAGAGCAGTAATTAAGTCTTGTT

AAATCTGATCTGGAGAGCCGTATGCGGAGAAATGTGCACGTACGGTTCGGAGGGAGACTGATCGTTGCCTGTCGAAAGAT

CACGGGGAGTCGACCCTACTTATTAACTACAATAATTTCACCTATAGCTTTATTAAGTAATTGATATTCTATAGATGAAA

ATGTTTTATCTTTGTTATAATAATACTAATATTAGAAAGTTTGTTATTAGCGGTTTTTAGTTTTAGATCTTTTAATGTTT

TATATTTTTTGAAAGTATATTGCCACCATTGTTTATATTAATAGGCTTATATGGCTCGAATAATAAAGTAAGAGCAAGTT

TTATTTATTTTTGTATACATTGTTCTCTAAAGTAAGACAAGGGGATTATAGCCCTATCTTATATCAGTGTGTAATAATCA

AATCCGGGAAGTCCTAAAGCTCGGCTTTGCCGCCCGGCACCATCGAAGAATGACTATCCCCTCTAATCCCTGGAGGGTTT

TCTTATTCGAAGACAGAAAAGGGGATACCTCCGACGATGATCGGGGGATCATATTGGTTTGGTGCGAGGGAGTAATCAAG

GATTTAAAGGAAACTTTAAATCTGGCCTGATTAATGACTCAGGGTATGATAATATCACTAGAGATATCAGCAATGAAATG

GGCAATCGCGGATCTAAGTCAGGAATGGGCCTGTAAAAGAGCAACGAGTAGATGGTTATTCGGTATTTAGCAATACTGTA

AGGTGTACTCTAGTCGCCGGTAAACCGGTTTTGGGGAGAAAATTTATAAAAATATCTCCAAATTTCAGTTTTATTCTGCA

GTAGTTTCATTCTTCCACATTAAAACCCACCCTCAGGGGAGGTAGGCCACCCCAGGGGTTTAGTTTTGCGGACCTAACTA

ACTCCCTTTGAGGGACGGAGGCACCGGGGGTCCCACAAAACTCGTTTGGCTTACCTCCGTAACTCCCCTTTGAGGGACGG

AGGCACCGGGGGTCCCACAAAGTTTTTCTGACCTCGTCGAGCACAGGTGGAGGGACGGGCACCGGGGAGGGTCCCACAAA

AGTTTTTCTGACCTCGGCGAGCACAGGTGAGGGACGGGGCAGCAAGCGGGGGTCCCACAAAACTAGTCCCGCCGGAGATA

ATAGGGGAACTTACGGGGTGGGTTCCTTCTCCAAATTGGATCGGGGGATAATCATCGTTCTTCGATCTAAGGGGGTAGAT

GGAGAGGGAACTAGGTTTTGCGTTCCTCTGGGCATCCCTTCCTCTCCTTTGAGAGGAAGGATGCAACCCCGATCCTTTGG

GGTTTTAACTTGTTGTTTTATGAGGGACAAACCATAAGTTTTGTCCCACAAAACTAGTTTTGCGTTCCTCAAACTCTCGG

GGGTTTGAGGGACCAAAACCAGTTGTTGTCCCACAAAACTAGTTTTGCGTTCCTCAAACTCTCGATTGATTGGGGGTTGG

TTGGTGGTGGTTTGAGGGACCAAAACTCCCCGATCAATTGGGGGTTCGTGGCCCACAATTTGCGGGGGACTAAACCCGGC

CCTCAAAACCAGAAAGAACCAGTTCCGTTTAGGGACGCAAAACCGAGGAGTCCCAATCGATCGGGCGATCCTATCAATCG

GGGATTGGGATAAGTCCCTCATCCATCGATCGGGGAGAATCGGGGGACTAGTCCAATCGGGGGACGAAAATCATGTACCA

CCTCGGGGACCAGTTCCTGGCCCCTCAGGAACGAGTTTTTCAAAACTTCTGGTTTTGAAAACCAACGGGTTTGTTTCCTG

CAAACTAGTCCCTCCCTTCCCAATCCGGCGGCGATCCGATGATCCTCGATTTATCGGGGATTGGCAAGGGGAAGGGATAA

GTCCCTCCGGGGAGGGACTAGTTTTTCAAAACTTGTTTTGAAAACAAACAAGTTGGTGTTTCCTGCAAACATCGATTGGA

GTTCCAATCGGGGGACTGAAAAGGGGTTCCCTCGGGACCAGTTCCTGGCCCTCAGGAACTAGTGTTTTAAAATTGGGGGA

CAACGGGAACTTAAGGTGGTGGTTCTTTTTAGTGAGTAGGGTTGTCCCTAAAAAGGCCAAATCTCAAATAAAGCCAAATA

AAAAGTTTTTTTCTTTTAATTTTTCAAATAAATTAGGATTTCTTTTAAAATCTCTTTTTATCCTGGCCTCCACCCCTGAT

CGGGTGAGGGAATCCCTCCTGTCCTGTCAAATAGGAGGGATTTAGTTTTGAGGAACGAAAAGCTAGTCCGGATCCCGAAT

CGAATGAGGGATTGGGATAAGTCCCTCGGAGAGGGACTAGTCCGGGGAAGGGAACTAAGGGGTTGGGCTCGGCTTAGCCC

GGAGGGACGCAGGGCGCATCCGGCTTAGCCGCAGGGGATTACTAGTCCCCTTAGCCCAGGGGGTACCTAAAACTTAAGGT

TTTAGGGACAAAACCACCACCGCCAGTTTGTCGCCAAAACCTGTGCCGAGGAAGCAGTTCAGGGTTCCCTCTGCCTAATC

CCGCCTTAGCATCCATCCCCGCTAAGAGGAGCCGCCGCCTTTAGCTGGGGATGGGACAAGGTCCCTCCCCGATTGGAGAG

GGAAGAAGTAAAACCGAATAAAACTGAAATATAAAACCCTTCCGAAGGGGAGGCCTCCAGGGGAACCCAGGGGTTTTAAA

ATTTTGCAAAAATCTCTTTTGCAAAAATATATTGGTAGATAAGTATACGTTGGCACTTAGCGTATACGGATAAGTAACCT

CAAATTAAAGATACCTCACTACCTAAAGCGCCGCCTAAATGGTCCCGGAAGGCCTCTAGGAATGTAAATATTCGAATTCG

CTTGCTAAGGGTTATTCCCTCGAGGCATACCTTTTGGGGGTTATTTCTTTAATAAAGGAGGGATTTTTATAACTCCACTC

CCAAAAGGTGCGTGCGTAGCCAGCCCTTTTTAGTTCCGGGGAACTATCGCCTTTAGGGGAGGGATAATATTTTTTATTCT

GGCCCCGCGAGGGGGTAAATTGGAATAATTCATGGGTTAAGAAGGGTGTGAGGGCCGTGTTTGGATCATTATTCTTATTA

TTATCAATATTAGTTATGTCTTCTATAATGGGTACAACGGATTTCGATGCCTTATATAAAACTAATTTTAATTATTCAAC

AGAATTCTTTTATTTTTGGGTATCTTTATAGCATTTGCCATAAAACACCCACAATATTCCTAAATAGTTGATTGTTAAAA

GCTCATGTTGAATCTCCTTTAAGCGGAAGTATAATACTAGCTGCTATATCTATGGCAGCTCTAAATCTAGCTAATTGCTG

GAAACACCTTGCACATATTTTTACAAGGTCGATCAGCAGGAAACTATACGCCTTTACAGAAATTCCAGGCGGCTATTTAA

CATATATTTTTTAGATAAAATTGCGTTAAATTTAAAATATGTTAATTTATGAAAAGGCGGGATTGCTTAAAACTTTGTTT

TATAAATAAGGACGATGGTTATAACTCAGAGTGCGAGTTGCATGACCTTAAATTACTCTTTAACATGATTAATATAAAAT

TAAATAGGATCTCCAGAGGCCACACGCTAGAAGTAATTAACGCAAATTTAAATAAAGACTCTGTGGGTTCCTCCGATACA

TGAGTCTAACACAAATACAACTGGTGAGTATTTAGAGAAAAAGCCACTCACCCCGAAATACCAATTTTCTACTTACTTCG

CAGGATTCATTGAAGGTGGATGGTGCAAAATATATACCTAAAAATAAGGTGCAGCTGCAATAGTAATTGCTTTAAAAAAA

GACGGAAAATCTTTTCCTTTGGCATTATTATTGCAAAAACTTAAATGGCTTGCTTGGTTGGCTTCTTGGGGCCCTTGCCC

TGCTTGCCTTGGCTTGCTTGGCTTGGCTTCGGTTGGCGGCTTCTTGGCTTGCCTTGCTTGCCTTGGGGCTTGCTTGCTTG

GCTTCTCTTGGGGGCTTGGGCCTTGCTTGCTTGCCTTGGGGCTTGCTTGCTTGCTTGCCTTGGCCTTGGGCCCTTGCTTG

CTTGCCTTGGGGCTTGCTTGCTTGTCGGTAATATTTCTAAACCTAAAGGTAAAGATGCTTGTACTTATGTGATCCGCGAT

CTAGAAGGTTTAAATAAAGTAGTCAATTTAATAAATGGATACATGAGAACACCAAAATAGTCCCGATAAATCCCGCGGCG

CCAGGCGGCCGCGGGATACGGGGAGGAACTTCTTCTTCTTCTGGTTCCTCCCCGGATGTCAATCATCGGGGATAAGGACC

CTCGTAGAGGGAACTAGGGTTTTTCGTCGTCGAGGTGGGCTCCCTCAACGAACTTTGGGTTTTATGAGGGACAAAACAAG

TTTTGTCCTCAAAACTAGTGCCTCCCCGGGAGGGACTTATCCCTTCCTTTCCAATCCCTCCCGATCGGTGATCATCGATT

CATCAGGGGATTGGGAAGGGGAAGGGACTAGTGGTTTTGTGGCTCCCTCAAAAAATCACTTGTTCAAACCCCTTTGGATC

GGGGGTTTGGCATCCTGTGCTGTTCTCTCCTTCGGAGAGGACGCAGAGGGACAAAACCATAAGTTTTGTCCCACAAAAAC

TAGTGCCGGCCGTAACTTGTTCCTCTCCCGCCTTCGGGGGCCGGAGGTTTAAAACCCTCGGGCTCCCCGAGTAAGGCCGG

TTTTAATTTAGGCTCCGCCAAAATTCACAGGAAAACTCCCGGCCCTTCGGGGAGGGTAAAACCCTCGGGCTCCCGAGTAA

GGGCCGGTTTTAATTTAGGCTCCGCCAAAATTCACAGGAAAAACTCCCCTTTCTTTTAATTCCAAACCCGAAGGCGACTT

TTATTCCCTTGCGGGGAATCCGTAAGGTGAATTATTCCATCCATTTCCCCTTTTAGGAATCCCTCCGGGGATTCAAAATA

AAAGAAAACCCTAATTTTGGGGTTTTTTATTTTAAGATTTTGGCTGCAAAGCCAAAATTCCCCAAATTTATTCCCTAGGG

GAATCCTAAGGTGAATATTCCATTGCCTTCCGGGCCTTTTTAGGAATGGAATTCGGGGATTAAAAATAAAGTTAGTTCTT

TTCCCGAAGGGGAAAAGAAAAGGGGGACAAGTCCCTCTACGAGGGAACTAGTTTTTCAAAACTTCTGGTTTTGAAAACCA

ACAAGTTTGTTTCCTGCAAACTAGTCCCTCCCTTCCCAATCCGGCGGCGATCCGATTATCATCGATTGGGGATTGGCAAG

GGGAAGGGATAAGTCCCTCCGGGGAGGGACTAGTTTTTCAAAACTTGTTTTGAAAACAAACAAGTTTGTTTCCTGCAAAC

TAGTCCCGGGGACAGGGTTCCCTCGGGACCAGTTCCTGGGCCAACTCGGGGAACTAGGTTGTTTTTCGTTCCTCAACGGG

GAACTCCGGATTTTAGGGACAAACCATAAGTTTTGTCCCACAAAACTAGTCCCTAGGAAGATCGGGGAACTCTTGTTCCC

TCCGATGATCACCGATCGATCGGGGGACAATTGGAGATTCGGAGAGGGAACTAGTTTTCGTTCCTCAACGAACTCTTTTT

CTTTGAGGGACCAAACGGGGGTTTCGTGGCCCACAAAAATAGTCCCTAGGAAGCGAACTTCTTCTGGTTCCCTCCAGGGA

CTCCACCAATCATCATCGGATCGATTGGTGATTATCGGAGATGATTGGTGGATCGAGAGGGAACTAGTTTTCGTTCCTCA

CGAAACCCGAGGTGGTAGGAGGTTTTAGGGACAAAACGGGTTTCGTGGCCTAAAACTAGTCCCTCCCGGGAGGGACTTAT

CCTTCCTCCTTCCAATCCCAATCAATCGGGAGGGATTGGGAAGGGAAGGAAGGGGACTAGGTTTTGCGTTCCTCTGCATC

CCTCTCCGAAGGGGAGAGGATGCAAACCCCTTTGGGGTTTTAACTCTTGTTGTTTTAGGGACAACGGGGGTGGTGGTTTC

GTGGCCCACAAAACTCGTCCCCGGATGATTCCGAGGGACTTATCCTTTCTCCCAATCCCTCCCGATCGGTGATCATCGGG

GATTGGGAAGGAAGGGACTAGGTTTTTCGTTCCTCTGGGATCCCTTCCTCTCCTTTGGAGAGGAAGGATGCTACCAACCC

CATTGGGGGTTTTAACTCTTGTTGTTTTAGGGACAACGGGGGTGGTGGTTTCGTGGCCCACAAAACTCGTCCCCGGATGA

TTCCGGAGGGACTTATCCTTCCCTCCCAATCCCTCCCGATTGATTGGGGATTGGGAAGGGAAGGGACTAGTAAGTATTTC

GATTTTTTGAGAAAAAGTCTTAAAGATGATGGGGTAAACCAAGAGCCATAAAAGAATGGCGCTTAAGTTATTCTTAAGTT

GAAAAAGTCTTAATTCTAGGCGGCGGCGAACAGACTTTAATTTAAATCATTTAAACCCAATTTGATCCTTATTCTTAAAT

CCCTCCTCCTCCTCCCTCGAAGAGGGAGGAGGAGGAGGAAATAGAATAGATTTTATCCATTTACATGTAGCCTTCCCTTC

CTTCTTCGTGTAACAGATATTTAATATCTTGTGTTTTGCTTTTATTTGTATACAGGAGACTTACAGAGTCCCTATCCATG

ACAGGGGCATGGCAGGGTTTATTTAATTACTAAGATATGGTCCCATCCAATATGTAAATATTGGCGTACCTACCTAATTA

TAGTCCCAATCCGCGCAGGCGCGGGATACTCCTCCTATCCCTCTGGGGATTAAGGGGAGGAACTTGTTCCGCTCCCGTCA

ATGATAGGTGGTGATCGGGGGATAATCATTGATCGATCGATCGGATCGGATCGGGATCGATCGATCGATTGGAGTACCCT

CATTAATCGGTGGAGATCCGGGGAACTAGTTAAATTTCTCAATGAACTAAAAAGTTATACTAATGATGCCTTGTGTAGCA

CTAGTTTATATTTACTTGTAATTAGAGATTATTAATCATACCCTTGTTTGGGGGTAGGTCAAGATTTTTTGATAGTTTTA

AAATTAAGTTTATATGGTATTTTTAGACTAATATTACCAATATTACCTAAAGCTTCTTTAGACTATACTTATATAATATA

TCTTATAGGTGTTATAACAATAATATATGCAAGTTTTAGTACTTTAAGAACAATAGATGTTAAAGAACTTATAGCATATA

GTTCTGTATCCCACGCAGCGGTTTATCTTATAAGTGTTTTAGTAATACAATACAAGGAATAGAAGGTGGAATAGCTTTAG

GTTTAGCACACGGATTTGTTTCAAGTGGTTTATTCATTTGTGCAGGAGGTGTATTATATGATAGATCGGGTACAAGGTCT

ATTAGTTATTATAAAGGTCTTGCTCAAATAATGCCTTTATTTTCTATATTTTTTGTTTTATCGTTAGGTAATTGTGGTGT

ACCTCTTACTTTAAATTTTGTAGGTGAATTTATGTCTCTTTATGGTGTATTTGAAAGATTACCTTTATTAGGTATTTTGG

CTAGTTCATCTATAGTTTTAGTGCAGCATATACTATATATATGTTTAATAGAATCGCTTTTGCCGGGTCATTTAGTAAAT

TTTTTATGCAAATATAAGTGATCTTAATATACGTGAATTTGTTATATTACTTACATTAGTTTTATTTACAATTGTATTAG

GTATATACCCTGCCCCAATATTAGACGGTTTACATTATTCAGTATCTTCTTTAATTTATAATAGTTGATTACTCTAGCCG

GTGCCCTCTTTGGTTTTCGAGCTCCCAGCAGCTTATCCGCTAAGGTACGGGCGCCGCCTTAGCATCCCATCGGGATTAGT

AGAGTAGGATAGGGGATAAGTAAACAGAGGGATTCGGGGACGAAAATAGGGGTAAGCCCTTATCTATGACTCTATGACAA

GCACCGGGGGTGGGGTATCCTAGGATACCGGCAAAATCTAGTCTGGGGTGGGGTATCCTAGGATACCGGGAAAATCCTAG

GCACCGGGGTATCCTAGTATACCGGAAATTGAGGGGATTAGGCGCGCAGGCTTGCTACCTAATCCTACGCCTAATCCGGC

CTTAGCAGGGGATAAAAGGGGAGGGATAAGCAAGGGAGCTAGACAAATATTAACTAGTCCCTTCCAATCCCCGATGATCC

CAATCGATTGAGGGGATTGGAAAGGGAAGGGATAAGTCCCTCGCGGGAGGGACTAGTCCCGCAGGAAGCGGGACTAGTTC

GGCTCTCCGGGACATCGATCGGAGTCCCTCGGGAGAGGGAACTAGTTTTGCGTTCCTCACGAAACCCGAGGTGGTTCAAA

CTTTGGGGGTTTGGCATCCAGTGCTGTTCTCTCCTTCGGAGAGGGATGCAGAGGGACAAAAAGAGTTCGTTGTCCCTCAA

AATTAGTCCTTCCCAATCCGCGGCTAGGCCGGGAAGGGGAGTTCTCCCGGGGAGGGACTAGTCCAATCGGGGACGAAAAT

CATGTTCCCACCTCGGGGACCAGTTCCAGGCCCTCAGGAACTAGTTTTTCAAAACTTGTTTTGAAAACAAACAAGTTTGT

TTCCTGCAAACATCGATTGGAGTCCCTAGGGGAACACGTTCGGGCTCTCCCCGCCTTCGGGGAGGGTTTAAAACCCTCAG

GCTCCCCGATTAAGGGTAAGGGTTTTAATTTTCTCCGCCAAAATTAAAAACTGGGGGTTTTGGAATTTTTATTAAAAACT

GGGGTTTATCGATTGGGGAATTTTATTCCTCCCCTTTCTTTAATTCCAAACCCGAAGCTACTTTTATTCCCTTGCGGGGA

ATCCGTAAGGTGAATGGATTCCCCTTTCTTTTTAGGAATTCGGGATTAAAAGAAAGTTAGTAAAAACCCAAATTTAGGGG

GTTTTTATTTTAATATTTTGGCTGCTTTGCAGCCAAAATTCCAAAAGACTACTTTTATTCCCTTGCGGGGAATCCGTAAG

GTGAAGGATTCCCCTTTCTTTTTAGGAATGGAATTCGGGATTAAAAATAAAGTTAGTTCTTTTTCCCGAAGGGGAAAAGA

AAAGGGGCCCTCTACTAGGGAACTAGTTTTTTCAAAACTTCTGGTTTTGAAAACAAACAAGTTGGGGTGTTTCCTGCAAA

CTAGTTCCTCCCCTTCCCAATCCGCGGCTCATCGGCGCGGATAGGGAAGGGAAGGGATAAGTCCCTCCGGGGAGGGACTA

GTTTTTCAAAACTTGTTTTGAAAACAAACAAGTTTGTTTCCTGCAAACTAGTCCCGGGGACAGGGTTCCCTCGGGACCAG

TTCCTGGCCCTCAGGAACTAGGTTTTTCGTTCCTCAACGAACTTGTTTCCTTTCTTGTTTTAGGGACAAAACCCACAAGT

TTTGTCCACAAAACAATAAGTTTTGCGGGGAAACTGGGGTGGATTGGGGGTTTTGGGGGCCCTCATCAAAACAACAAGTT

CAAACTTTGGATCGGATCGATCGATTGTTTGGCGGCATCCCCTTCGGAGAGGGATGCAGCAGAGGACGAAAAACTAGTCC

CAATCAATCGATCGCCGATCAATCGGGGATTGGGATAAGTCCCTCATCGATCGATCGGGGAGAATCGGGGACTAGTTTTG

CGGGGGACAACGTAACTTTTTTGTCCCTCAAAAGCACAAAATCTAAAATAAATAAAAAATAAAAAAAAAAATCCAAATTA

AAAATTTTCTTTCTTTTATATTTTGCAAAAGATATTTTGCAAAATTAAAACCAAAATAAAACCTAATTTTTTTTTGGGGT

TTTATATTTTATCCCTCACCCCTAGGGGTGAGGGAATAAAATAGGGATTGGGAGCTCGTGAGGAACGCACGCAAAATAGT

CCCTATCCCAAGGGGATTAGGAGGGGCATCTTGTTCCCGGGGCTCTGCCTAATCCCCTTTTTGGTCTTTTCGCCCCTCTT

AGCTGAAAACCAGGGGAAAAGGGGGATACCTCCTGTTTCCTGTCCTCATCGGGGATGGGCCGCACGCTGGCTGGGGGCAT

CCGGGCCTGCGGGGGATTACGGGCCTTTCCCCAGCAGGCTTCCTCAAACTTAAATCCCTCTGGGCTGGGGAAATCTCTTT

TGGGGGTTACTAGGTTTGGCTTTTCAGGTTTTTGCAATTTTTAGCTCTTATTAGATAGTATCTTCAGAGCCAACACAGTT

TAGTAGTGCTGCTTAGGGGCAGCCGCGGTTTTGTTCCTTTTCTTCCTATTATATCTTTAGGGGGTTTGTCTAAATAAAAA

TCCCCTCATTTTGATGTTACATAAGATATTTGAATAAATATACATGTTATAGCGTAAACATATAGTTCCCTAATATTACG

GGCTTGCTCCTAGGGGAGTTGCGGGGATCTTACCCTGGCTTATCCCCTCCTGGCTTGGGCTGCGTAGGGGATTAGGCAGA

AGTGGCCTTATCCTAGGGGATTAGGCCTTGGCAGGCAGCAGCGGGGCGCGCCGCCAATGGGGACCTTGCTGCAGCCTCTA

CCCCTACCTTTGGCTGCGTAGGGGATAGCGCAAAAAATACCAGGGGTAAGGCAGTAGTATAAACTTTAAGGGATTTGGCT

TAGGGGATTTAAGATCGGGGTATCCCAAGGGGATAATCTCAAATTACAAGCTCTATAGCATTGTTAGAATATTAGGGTTT

TATTTTGTCTGCCCTTTTTCGACTCCCCAAGAGATTTCTCATAATGATGTAAAACCAAATCATATATGACATCTAAGCTT

ATGATCTCTTTGCTAAAGAATAATATTTCTTTATGGAGAGAACGTTGATTATTTTCTTCAAATGCTAAAGATATTTTACG

ACGCAGAGAACTAGCATGATACCATAAAGTATTTCACGGATATGTCCAAGACTGTGACAGCCTATTCAGAGTGGCAATGA

CCAACGAGACATTGTTGCGAGTTCTGATGGATAGCCTTATAGTTGTAGACGCCAGTCTAAGTACAATAGATAAGGCGTGC

TGAATCCAAAATTCTGAAACCTGAACGAGAGTGAACCATCATATTAAAACGGGTCTACTAGCTACATATGACGGGCGTCC

TATCTATGGGAGAATCATGATAGCCAGATACTTTGATATCATGTACCTGGGGACATACTATCTTCTCACACCTGAACAGA

TGGCAGATTTCAGAGTTACACCAATGACAATTTACCAGCCATTAGGTAAGGACATACTAAATTCAAAGCGGGAACCTCGG

ACAAGAGTTGAAACATCATCTCCTCGGGACATCAATCGAAGGTTAATACTTGGGATATTAACGGATGAATCGTTACACAC

CTGAAATACTCCAAACCTATAGGGATAGAGCATTCCAGCCGAGACGGGGTAAGATATTCGAAAAGCGAATGCCGAGAGTA

ATTGCTCGGATACCGGGTACAGTCAGCTTTCCTGACTAAATCTCGGACACACTACAGTGGGCAGAATTCGAAAGTGCTAT

TGATGACCTACTGTCGTCCAACCTTACCAGCTGGGACGTGTTACGCCAGAGATAGATACTTCGGTAGCTATAGGAAGGAC

ATTACCAGTAAACAATGGAAACCTTCAACTTCTTGAAAGACCATTGAACTAAAGGTTCGTAAGGAACAAATGAAATTAGT

AGAGTTAGCGCAAGAATCTGGGATTAGTAATGAAAAGGTCCTAAAATTCCAAAGAACTATGGCCCTTCAAACAGATTTGC

GAAGATGAGCAGTGCAGATAGTACTGACCAATAAAGGAAAAGCCACCAGAGGGACGGATAATATAGTGATCGAAAGTGAC

GAAGACAAATGGAAAACAGTTGAATGGTTAAAAGCTATGATTCTAGATCCAAAAGGCTATGAAGCAAAACCGGTCAAGAG

AGTATATATCCCTAAGCCCAACGGTAAAGAAAGACCTCAAGGTATTCCTACTATGAACGACCGATGTCTACAGGCTCTTA

TTAACCTAACACTGGAACCATTAGTGGAAATGACCAGCGACAGACATAGTTATGGGTTCAGGAAATTGCGATCCACAAAG

ATGGCCTTAGGAGCTCTCAGAGTTAATCTAAGAAGTGCAGAAGGGTTCTACGATAAATATGTTCTGGATGCGGACATTAA

GGGATTCTTTGATAACATCTCTCATCAATGACTGATGGATAACATCCCCTTAGAGTGTACCTTAAAACATATACTAGCTG

CGTGATTAAAAGCGGGATATATTCATAAAGGTGAATTTACTGAAGCTACGGATAGCGGAACTCCGCAAGGAGGTATTATT

AGTCCTACTTTAGCGAACTTTACACTGAATGGACTTGAAACCGCAATTGAGGAGGTGGTAAAGAATAAATACGATGTTAA

AACGAAGGGAATTTATATAGGGAAACTCCAGAACCCAAGAGGGAAAGTTAAATATGGATTCTTGGCTACTAATGTATTTA

CAGTAAGATTTGCAGACGACGTAGTCGTACTTGCTAGATCTAAACGCATGATTGAGGAAACAGTTAAACCATGTATTGGC

TCATTCCTTAAAGAGAGGGACTATGGTTATCTGATGAGAAAACCCAAATCTTATCTATAAGAAAAGGGGACAAACTTAAT

TTCCTAGGGTATACATTCCAATACTTCAACAAGGTAGCCCACAGAGCTAAGATGTTTAATGACCGTCAAGGGAGAGAAGC

GATTGTTTGTTATCCTCAGAAGGGTAAGTATGAAGGTATAATTGAGAAAACCAGAACCTTATTTGAAACTAGTTATAACC

TAACGGCATACACTCTTATAGCCAAATTAAACCCCATAATAAGGGGTTGATCGCAATACTTTAACTTAGGACAGAGCTTT

AAGTTCAGAAATACCTTAAGTTATAATCTATACAAGTTCACATGGAAGTGAGCCTTGCACAAACATCCGAGATGAGGAAA

AATAAGAATCGCGAGACATTATTTTCTGAATCCAGAGAAAAAGGGAAACAGTTCACCATTGGAAGGAAAGTCTCTGAAGA

ATGCCACGAAATGGATATTCAGGGTCGTACACACAAGGACTCTATTTTCAAAGAGAACGAAGGGGAAATATATTGAATTA

GTTAACCCTACTCAAATAGTATCTACTATGAGTTCGAAACTATACCGTATACCCAAAGATCTCGAACTAATACACGCGTA

TCACTCTGACTACGAAAAGCTAATAGAATTCAACAACAAAATGAGTATTGAAAGCTTGAAACTAAATAAAACTACCAAAC

TAAAGTTATTTATTTCACAGAAAGGAAAATGTACAATGTGTAGCCAATCTCTATTAAACGAACAAGGGAATTTCATTATG

ATGGATCGACACATATTCACCACGATCTGGCTCGTAGCCAAGGAGGAAGTAAAAGTAGACTGTCTAACTTGTCTCTTATA

CACAGATCATGTCACATAGAACATCATAAGAAAGGATCTAACTTGACGAATATACGTTAGAGAAATTGACAAACAGAAAT

AAGCGCTCAATTGTAGGAACTATAACTGGTATACATAGGAAGTGGTTGCGAAAATTGATCGATGGTTGAGCCGTGTGCAG

GGTGACTTGCACGCACGGTTCTTTGAGGGGCATTTCGGGCGACCGAGGTCCCATCTCTAGTGGTACTTTGTACTTAATAT

TTGCATTATTTGCAGGTTTATTGGGTACAGCATTTTCTGTTCTAGTGCGCCCGTGCCCTATGTTTACGGTAGAGAGTAAA

GCTCTCCATCATTTAATCTACATGGTGACGTTATCGGGTTTAGGTTTAGGGAAAGCCCGAGGCCTAACTTAGCATATCCG

CTAAAAGCGAATGAAAAGGTAATTCATGTATCCTGTAAGACGCGAGACGTCGTCCCCATGGTCAAGGTTAGACTACTTGA

ATCCCATCCACTAGTCACCTCCGGAAAAGGGTTAGTACATGCATGAAGTTGGTACTATGGTTTGATCAATTATAAGTTAG

TACAATGATTGATAGATTACAACACCAGACGAGCCGTAAGAAACGGTTACAAATCGGAGAGTGGGAGACCTAAAGGGACT

ACCGCCGTAAACATGGAACGCGGGATTGCCTAAGTGTAGGTGCTTATCCTACATATGGTAACGGAGCCTCCGTAGTAGGA

TCATATCCCAAGGGAGGCAGAAAATGACGTTTCAAGTCAAATTCGCAGCATAACGGGTTCCGTTGTTACTCCAAAGCGGT

GACTCCACTCCAAGATAACTTAGAGATGGACACAAACACAGCTAAAGGGGATCTGCCTAGAAGATTTGAAAAGTTAACTC

AAATATGTGCAACTCATAAAGACGGTCTCAAAGCGAATGATATATACAAACTAATGTTCAACGTTAGAATGTATGAAGTC

GCATACCATAAATTAAGATCCAACCCGGGTAACATGACTCCAGGTATAACCCCGTCACATTAGACGGGATATCCCTAGAA

TGGATAAAAGAGACCATTAACCAAATGAGAGATGGAACCTTTCAATTCAAACCTGGTAGAAGAGTACAAATACCGAAACC

AGGTAGTTCAAAGACAAGACCACTAACTATTGCACCACCGAGAGATAAGATTGTTCAAGAGGTTATGAGAATGATATTAG

AAGTAATATTCGAACCAACATTTTCAAACAACAGTCACGGGTTTAGACCCAACAGAGGTTGTCATACTGCTTTAAGACAA

GTTAAAACTCAGTTTGGTGCAGCCACAACCATCATCGAAGGAGACATATCAAAATGTTTTATAGCTTTGATCATGAAATA

TTAATAGGACTGGTAAAAGAAGGGTGAATGATACAAGATTTATTCAATTGATATGAAAGGCCTTGAGAGCGGGATATATG

GAATTCCATACTACCCAACATTCAATTATTGGTACCCCACAGGGATCTATAATTAGCCCACTACTAGCCAATATATACCT

CCACGAGCTAGATATATATATTGAAAAATTAAAATCTAACTACGAAAAGGTGTAGTTGCTAGTAGAAATCCAGAATATCG

GAGACTAGAATATCTAAGATCAAAGGCGAATAAAGCGAAGGATTTCGAATTAGGTGTAAAATACCTAAAAGAAATGCAAC

AAATTAAATCGCGCCTCCCTAGTGACTCAAAATTCCGAAGAATTTACTACGTAAGATACGCGGATGATTGAATGATGGCA

GTTAGAGGACCTAGATCTGACGCAGTAGATATGCTACAATCAATAAGAGACATGTTGGGTAACTCCTAAAACTTGACTTA

AGTGTCGAGAAATCCAAAATCACTAGTCCAAGGGTTGAGCCTGCTCTATTCGTAGGTACATTGATCAGTATTTCGAACCA

CGTAAATTCTACAACAGGGAAGAACCACCAAAGACTAAGAGTAGCAAGTCAATTACGTATGTTAGCTCCGATGGACAGAA

TCTTTAAGAAGCTAACTATAGCAGGATTTATGAGTGCGAAGTATAAAAGTGGAATACCCAAATTTATCTGACTTGCTAAT

AACAAGGAAAGTATCATTAAACTATATAATAGTGTATTGAGAGGGTACCTAAATTATTATAGCTTTACTCATAATTATTC

GAGAGTAGCTAGTTCACTGGAGTTCATCCTGAAGACCTCGTGTGCTAAATTACTGGCAGCCAAATTTAAATTAGGTTCAG

TAACAGAAGTGATTAAAAGATATGGAGGGAATTTAAAAGGAGAAGATAAAATCGGATTCCTCAAACCATCATACAAAATA

AACGTGTGGGATTTTAAATCTAACCCTAAGGATAGGATTAAAACACTATTCGCGTCATATTTGTCAGCCGCTATACTTGA

CTCTCTAGAATGTGTAAAATGTGGATCTAGCTACAGAGTGGAAATGCATCATGTTCGTTTACTAAGTGACCTAAACCCTA

AATTATCTGAGGTGGATAGAATCATGGCGAAACGAAGAAGAAAACAAATCCCATTATGTAGAGCTTGCCACTTGGAGCAT

CACAAGAATTCAAAACCATGGGGTAAAAGTCTCACAAAAGAACTAAAATACCTATCTTTACAAAGGTTACATCACATTGA

GTTAATAGTGCATTTCTGGAGAGCCGTATGATGGGAAACTATCACGTACGGTTCGGGAAAGGGTGATCGTATGTTTAAGC

AAATCTCAATGAGATACAAAAGACTGCGGTTATCTAGTTCACATAAGGTTAGAATTAAGTGGACCAGGTGTTCAATACAT

TGCGGATAATCAATTAGTGCGCCGAACGTCCGTAACTGCTTACACAGATTATTTCCTTTATATAATCACGGCATCAAAGT

TAGGTAAAGGGAAAGCCCTGAGCTGAACACAGCATCTTTGCGTCACCCCTTCTTCCATTATATAAGCCAAATTTCCTTCA

TAGCGCCACGTAACAAAGTTGCCTGCGGAGTTAGAACATACTCTTCTTTTAATAATTCAAAAGATTACCCTTGTATAAAA

GGTTATCCAGATATGGAATCTGCTAAACTCCTTATTTTAGAAGATAATAAGGAAAAATGTGGAGTATATTTACTAACTAA

CAAATTAACAGGAAAGATTTATGTAGGAAGCTCAAAAATCTTTCTAGAAGATTTAAAATTATTTTAGTACAGGCTATTTA

AAACATATTAGTAGGCGAATGATGGTAATTAATAAAGCATTACTTAAATATGGTTATTCTAACTTTAAATTGGAAATTTT

AGAGTACACAATGCCTGATAAAGTGTTTGAGAGGGAACAGCACTTTTAGATTTATTAGAGCCAAAGTACAACATGTTAAA

GTATGCGGGAACCTGGTTAGGATATAAACATTCAGAAGCTAGCAAGTTAGCTATGGGTGTTGACCGTAAAGGTGAAAATA

ATCCCATGTGAGGTAAACCCAGTGCATTTAGAGGTAAAACCCATTCAGATGAAACCAAGGCTAAAATAAGACTAGCTAAT

AAGGATGTTAATCTAGGTAGATCCCTTTCTCAAGAAACTAAAGATAAAATGAGTCTAGCTAAAACAGGAGCAAAACACCC

TTTTTGGAAAGAGTCTTCCCGATGAAACCAAATCAAAGATCAGTAAAGCACATAAGGGGAAAACCATTATTTGTTTGGTA

AATCTCAAACGGAAGAAACTAAATCTAAAATCAGTTCAAAATTAGGGCATGCTATCTTAGTGTATGATATAGAAACCGAG

GATAAAACGTATTATACTTCGATAAATCAAGCTGCAGAAGGTCTTTCTTGTAGTAGTTGAACAATTAGAAAATATATAAA

CAGCCAAGATCTTTATAAGGGTAAATATAGAATTAGTAAAGCTCCAAAGCTTTTAAAAATGGAGACAAAACGGAGGAAAT

AGCTCCGGGTTAAGTGAAGGTAAATCTGGGAATATGGAAAGGAAGAATGAAAAGGGTAGTGGTGAATTAGATATGTTCTC

CGCTACTGTTTTAGGGTGCTAGAAGGAGTTTTATGACTAACTTAGGTGTTCTAAATTACCTCCGAGCCGCTTTATCTAAG

GTAAAGGTTATACTACTTGAAGTCTACTTAAAAGATGCCCATTTTATCGGGCCTAGTTTAGCTAAGGATCGGACTAGACA

AAATGAGTTAGGATTAGATAATTGGATTCTAACTAAAGGACATTTTTATCGGGGTGTAAAATACACTCATAATTTAAGGA

ACGGGCTACCTAAGGATAAATTAGAAGGAGCAGTTTTTCTCTTTATGTATTCTTTTTCCTTTTTCCCTATTTATTAAACA

TTAAAGTATTATCCATTTTATTCACCGAACAACCCGTTGTGTTTACTAAAAGACAGATTACTTCTTGTCCCCTATCTAGA

GGTGAGATTTATAAAATAGGGCGTGCTTATTATTCAACTCGTACTGATACCTCTGAACAGGGTCTAGACACGCCAGAAGT

ATGCTACGACAATCCTGAGGTTGATAAATCACGGATCCTTAATGAGAATCGGGGTAAATCTGGTGTCTATCGTTGGACCA

ATAAAATAAATGGCAAATGGTACGTTGGGAGTTCAGTAAATTTGTCTAAAAGATTTTCGGTTTACTTTAATAAAGCTTAC

ATGGATAAGAATAAAGACAATATGGCCATAAATAGAGCTTTACTTAAATATGGTTATTCGAATTTTCGGTTGGAAATTCT

TGAGTATACTACTGCTGAAATGGCTATTAAATTAGAGCAACATTATTTAGATTTGTTTAACGAGCTGCCAGCATCCCTTA

GTTATAATATTTTGACTATAGCAGGTTCACGATTGGGTTATAAAGTCCTTGATGCAACCAAAGAAAAGTTAAGGTTAGCA

AACTTGGGTGAGAATAACCCTAATTTCGGTAAATCTCATTCAAAGGAAGTACGGGATAAAATAAGTGAAACTCTACTTAA

TAGTGTAAATCACCCTAGTAGAGGTAAACCAGGTAATATAACATTTAAAGATAAAACTCATACCAGTGAAACCAAGGATA

AAATGAGTATAGCTAAATTGGGTGCTAATAACCCCAACTTTGGTAAATCTTTGGACGAAGCAGGGAGACTTAAACTAAGT

ATTATCCGGGGAGTGCAGTTGATGTGTATGAAATAGAAACCAAAATAACCTTAACTTATCCTTCGTTAGCTAAAGCAGGT

GAAGCTCTTTCATGTAGTAAAACAACAATCAGTAAGTATATAGAAAGTAAAGAAATCTTTCGAGATAAATTTGTTTTATC

TAAAGCATCAGGTAATAATAAAGAACAAGGAGAGGGTGGTGAAAGCCTTACTGAGTCTAATGCCTTCACATCGAACCAAG

TGAAGACTGCTAAATTATCAAAACAGAAAACCAATTTAAATTCATGTGTTTTGGATTGGGATGGTTTAGTGGCCGGTTTT

AAGTCTCTCCTAAGATTCAACCATGTTATTATTAAGAATATAGCCGTTATATTGTCGGGTAAATTGCTTGAAATTTATAT

ATGTTGGTGGTGTAGTATTACTATGGATTTTACCATTAATGGCTACTTATCTTTAAATAAAGCTTTTTATTTAAACAGGG

TAGTGATAGATCCCAAGGGTATGGTGTGAATACACATCTTAAAAAGGATTTGAAGATGCAAAAGGGTGGAACTTCGGGAT

TGCCTGTAGTCAGTGATGACCAGGGCAACGGAAGGATCGTAGTACAATATATTTTTCTCTCTTGTAAAGCATTCGGCATT

TATATGTAATGAGAATAAATTATTTGGAAGGGTCCTAGGAAAACACGCGTCTACTTATACTAGGGTTGCTGCTTTTGACA

GTACAATTAAATTTAACAGCCAAAGTAAGGTAGATAAACTAAGTGCTTATAATAAAAACAAATGTTACAACTTACAAATG

TAGCCTTTAAAAATAAAATATATACTAAACTTTATAACCAAAATCTTTATCAACTAGCTTATGATATTTTGTACTATAGA

CCTTGTAAAGTAGTAATTACAGGTGTGGATATATTATCGTTGAATGACATATTCGAATGCTCGCTATTGCATCCGAGTAT

TTTGGGAGAGAGATTAAACTCTTGGATATTTTGGATATAATATTTAAAATAAAAGAAGGGAACTATGTGTTCACAGACTT

AAGTCTAAAAGGGGACCTAAAAGTTCACTATTCTAAACAATTTTTCTGTAGCTAAAGATATTTTGTTAATTAAAGCTATA

GTCATTTTATTAGAAGCTTTTATGAACCCTTATTTAACTCTCTACCTCAAAGCTTTCGTCCTATTACCTATAAAGGCACA

CCGCGCCCTTCTGGAGGACTCATTCGGAACGATAAATGAGCTCATTTACCTTTCCATTCAGCAAATACCCATGAACTTGC

TTTAAGGGAGCTAAGGTTTAAATTAAGAGGTGTCAAGTGAATTATAAATGTAGATTTATCTAAATATTTCGAGCATATCG

ACTTCAATGTATTAATGTCTATTTTAGAATTAAATTTAAAAGACAAGAGATTTTCAGATTTAATAAGAAAGGCCTTAAAT

GCAGGCCATTTTAACTATTTCGGTGTAAAATCTGAAGCATCTAATGTCCCTCTTAAGAACTTAAGATCTTCGTTATATCC

TATACTTATTGACATCTTCTTAGAACGTGTAGATAATTATATTTCTTCTTTGGAATATCAATACACCAAGATGTCATCTA

AAGAGATTAAATTATCTTATGTAAGAGCAGGGGATCAGTTAATTTTAGGTGTGATAGGTTCTTATTCGGATTGTGTAAAT

ATCATAAATATGCTAATAGATTTCTTTAAACAAACTTTGTTCTTAGAGGTGTCCAAGGAAGCTTTTAAGATTATTAATTT

ATCTAATACAAAGGAAGCTTCATCAATAGTTTTATTGGGGTTCGTTTATCTATTGTAGACCTCAAACTCTCCTCTTTCAA

TAATACTATAACGTTGACAGCTCCTATCAATAGAATTATATCCAAATTAACTAAAGCAGGTTTCTTGAAGGATAGAAAAT

CTATACCTAAGTTAACTTGAACTCCCTTTAATAAGGATGTGATTATCTTTCTTTATCAGTCTTGGTTCCAAAGTATAATG

GATTACTACGGTAAGGTAATAAATAAACGTCAATTAGCCTCTTATTTAAATAGGGTGTTAAGTTCTTCTTGCGCTAAACT

CTTAGCTTCCAAGTATACACTTTCTTCACAAAGAAATGCGGAGAAAAACACGGTAGTACATTAAAACTATTGGGTGCTAC

GTCTACTTCAAATAGTAGCCTTATAATAAATAATAATTTAGTTCGGGTTGCCAATAGACTAGATAAAGTAAGTAAAAGAA

GATTAAATACTATGTTGTATTTAGTGGAAATTAATAGATTTAGTTCAAGCAAAGCTAACGGGCGTAACGAATCAGCCTTA

CCTAAAGAAGGATTAGGAAAATAAGTAAAAAGCTTTTAATGTGAAAGCTTATATAGCAGGGTTTATCGATGCTGAGGGAA

ACTTTTCATAAAAGTAGTTAAAAGTTCTACTATAAGAACAGGGTACTCAGTCCAACTTACATTTGGTTTAATACTACACG

ATAGAGAGTTATATTTACTTAAACTAATTCAAGCGGAATTATCAGGAGTAGGTTATATTTCAGAAGCTATATCTGGGAGA

GTTCATTATCAAATTTCAAATATGAAAGATCTCGAGGTATTATTTGCTATTTTAGATGAATTTCCTTTGTTAACTCGGAA

AATGAAAAACTACCGGTTATTCAAGGAAGCGTGGCGCATAATATCTAATAAGGAACATTTAACTAAAGAGGGTTTAATTA

AAATAGTATCTCTTAAAGCCGCTTTTAATGATTCAGGTTTATCCGAAAAACTGCAGAAAGAATTTCCTAATTTAGTGTCT

TTAGCTGAAATTTGCTCACCAGAAGAACAAAACATGGAACATAGTATCAAAGATCCTAGTTGATTAGTTGGTTTTGCAGA

TGGAGAGGGTTGCTTTTGTTCAATTAAGAAAAGCTGCTGGTTATAAAGTAGGTTACCAAATATCTCTAAAATTCCAAATA

ACTCAGGATACTTTGGATAAGAAGCTTTTACAAAGTATAGTGGAATACTTAGGTTGTGGTAATTATAGAGAAGTAGCCAA

AAATAATGATGGTAGATTTGAGGTAGAAAGCGCCAAAGATATATTAGCTAAAATTATTCCTTTTTGATCAATATCCTTTA

TTAGGTTCAAAAGCCAAGGATTGTGCTGATTTTAAACAAGTAGCTTTACTTATAGAAAAGAAGGCACACCTAACTCCAGA

AGGTTTAGAAGAAATTAGAAAAATTAAAGCCGGGATGAATAAAGCTAGATGTGAAGATTAATTTGTAGTTTAAATTAGTA

TTTTATTTAGATTTGTTAAAGTAGAGTTGTAGATGTTGTAACTTTTTGTCGATATTATTATTTTTTTTTTTTTATTTTTT

TTTTTTAATTCTGTCCTTCTATCCCCGTAGGAACGGAGACTAAATTAGTTAGTCAGCGGGTGAACTTGCTAAAGATAAGT

AGGCTTTATTCTCGTGTCGAGGAAGGAGCAAATTTAACAAGTATTGCCTTTTATAGGGGAACGGAGGCATAAAATATATA

ACTGATTAATAGGTAATTAATAATAAGATGTAATTACTTTAAGCACGAAGTTAGTGTTTTCTGGAGAGCCGTATGAAGGG

AAACTTTCACGTACGGTTCGGGAAAGGGTAGATACTTTGCTTACCCAATCTATTGCGAGTTAAGTGGTTTCTACTCTAGT

TCACTACAATAGTATCATAACAGCTCATGCAATATTAATGAGTGCGCCACTGAGCTTTGAAGAATGGTCGGTTCTTTTAG

AACCCGCAAACGAAAGTAGTTGTGTAAAGAGCTATCAGGATGGTGAAGCCAAACTTCATGGGGAAACTCCAACATCTAAG

CAGATAGAAAAGGGGTAAACGAGATAAATATCCGTATCTTAGGACTTATCCCTCATCTAATTCATGCAACGTCTAGGCTA

ACGAACTTGAACCAACGCAATATCCTTTGCCATTGGAACTCTGCTGAATGGATAGATAGGGCAGATGCGTTATTTCCTTT

GATTTTATCATTAGTTTCAAAGCTACTTTCTATAAATAACGGGATACCTCGCAAAGAGGAAAAACAATGCGCAAGAGACC

TAGCATGAAACGCCATCTCAAGGAACTGTGGGATCACCTACGGGGACCTAATGAAGGTACCTAAGAACCAAACTAATAAT

TTGGGACCCCTCAAGGGTGGTGACGGAGTCGTCATAGTAGGATGAAAAGTCCAAAGGGCGACAGTTTCAAATTTACAAGT

GAGAACATTGACATCAAAGCCGGCAGCAATGCGACACGTGGCAGCGATGTCAATAATACACTTGATTCTAACGAAGTGAA

TATTAAGGCTATAAGTAACTACAAGAACTTAGTGACAGCATATGAATTAATTAAAAGTAATCCAGGAAACATGACGAAGG

GAGTAGGTAAAGAAACTCTCGATGGTATGGATCAGAAATACCTCGAAAACGTGCAACTAAAGTTGAAAGCTGGAAAATTC

CAATTTAACCCGGCCAGAAGGATCCAAATCCCCAAACCCGGTAAAACGAAACCAGACCTCTAACTATAGCCGCCCCTAGA

GAAAAATAGTACAAAAAGCGATCCAACTTATCATGGAGAGATTATATGAACCTAAATTCCTACCCTCTTCACATGGATTT

AGACCAGCGAAAGGCACACATACCGCTATGAAACAATTGGAGTCCAACTTTCAAAGCGTTAGATATGTGATTGAAGCCGA

CTTCTCTAAAGCGTTCGATTCAATTCAACATGACGCCCTAATGAGCATAATCAAAGAAGACATCAAATGTGAGAAAACGC

TCAAACTCGTTGAGAGTGGTTTAAAGGCCGGATTTCTCGAATTTGGTGAATTACACAATAATTTGAGTGACGGAACACCT

CAAGGATCAATACTAAGCCCCTACTATGTAACATCTTCTTGCACAAATTAGATGTTGTCATGGAAGAGTTAAAAGCTGAA

TACCAAAAGGGAACCCCAAGACGAAGAAGTGTGGAAAACATGAAATTACAAAATCAGGCAAAATACTGAAGAATGAAGGG

TTATGATAAAATTAAACCCCAATTGTACAGAGCCATTATTAAACGATTATTGAGGACAGATAGTATCATGAGAGATGACT

CATATGTAAGAATACACTATATTAGATACGCTGACGATTTTGTAGTGGGAGTTATAGGTAGCCATGCACTAACCAAGACT

GTACTCGGAAAAATTCAAGACTTTGTAAACAGCAACCTAAAGCTAACATTCAACGAAGATAAAACAGGAATAACCGATTT

TTCCAAAACGATTTCAACTTCCTAGGGTTCAAGATCAAGGCACCATTGTCGAAAAAGGGTACAAAACCGTTGGAAACCAT

ATGCCTTAATGGGAAAACCATAACCAGAAGAAAGAAAACCCGTATCATCATCGATATGGACACCGAAAAGTTTTGAAAAA

GTTGGCTAGTAATGGTTTTATACGAAAAGAATTTGCCATACCCAACACAAGGAATTAGAATATAGAGGAGGGTTCAAGGG

TAATTTAATAAACTTAGATCACCCAGACATAATAAAATACTATAATTCCGTTGTAAGAGGAATACAAAATTATTACAGCT

TTGCTAAGAATAGAATTTCTATCTCGAGAATAGGATGATTGGTGAAAGAATCGTGCGCCTTAACCTTGGCCAGAAAGTTC

AAACTTAAAACACTAGCCAAAACTTTCGAAAAATTTGGGAAAGACCTCGGGTACAATGTGGACAAAGAGAGAAGAATCTC

TTTATCAACATTTCATACACGAGAGCCACTAATATAGCCAAAGTTGTGGGTATAAGATTGGACCCCTTAAAGAGTATCGA

AAAAGTTTGAAATGCCAAGTTTACAAAGTCTAAGTTAGGCGCTGCATGTGTAATATGTGGTATTTCCGAAGATGTAGAGA

TGCACCATGTAAGACAGATCAGGGACATGAAAAATCCCAACAGCAAACTGGACTTCTACACTAGACAGATGGCCGCCATC

AATAGAAAGCAAGTTCCGTTATGCAGATACCACCACAACGGACTGCACAACAACACTTGAACTAATGATGAAAAGCCATC

TTTAGCTATGAAGCAAAAGAAAAGCCAAAATAATGAAAGGTAGAATTACTTAATCCTTAATACACTTCGAAGAAGATCGG

AACGATTAAATATGAAGCGGAGAGCCGTATGATTGGAGACAATCACGTACGGTTCGGAGGGCGGCGGAGAGTGTAAACGA

TTCAGGCCGACCCTACTTTTCTTTATGGTTATGCCAGCTTTAATCGGAGGATTTTTCGACACGGACAAACTTTAAACGTG

CATCATGCGCATATCTGTTCTGATCATTCACCAAGCTGTGGTGTAAACGGATTAGGGTTAAACAATGCAGTTAAGCCCAA

CGAGGTATGGCTGTGAGGAATTGCTAAATGGGAAAAGGATAGAGGCAGCATATATTCAAGTACCCTACCTGAATGTCAAG

AGGTTAGTATAAGTTCAACGAAAGTGAATCTTCATTTTAAAGATGGTTCAACGATACCCAATCTGCTGATCGGAAATGGA

TTGGAGTTCTCATTAACAGAAAAGAAGCTATCTAAATCCATGGCTTCTTTGATCAGATCTGGAGAATACACCATAACAGA

AGGGGACTTAGACGACAACCCCGGTGAGAATAGACTAGTCTATAGTCTAAAGGAACCAGACAGTTTTAAGGCTAAACGTG

GGAACCCCGTACAAAAGATCTTAAGGGTTCTGGAAGATACGAACTATTGAGATTTTATTGGGGTCATTTAATAGTTAAAT

GACTGTCATGCACCTATCCGTCAGCAAAGCGAACGTCAGAGATGGATAGCCAATACGGGGCAAGATAACTGAGGAAGCCA

ATGCCAAGAGTAACTTCTTGGATACCGGAAATATCCACATCACTCGGAATATTTCTGGGCTGCTATTCGAATATAGTAGA

CTGCAGAATTCAGTAAGCCTTATCAAAGTTTTGCCTATTCAATTTGGTAGAAGATACTCATCCCAGGACCTAGAACAAAG

CTCTGAGAATGAGACCTTAGTCATAGATAGGAAGGAGGCTTCGACAGACGGATACCAGAGGTTTGACCCGAATAAAGGTA

AAGATATGGTTGAAGAAAACAAATTGAACTGGTAAGACTAGCCGAACAATACGGATTATATGACAACAGAGTCTATGACA

GGCAACTTATATTAATTAGATCTTTAAATTTCCGAGAATACACAGCTTGAACTATCATAAATAAACCGGGGGTCCCAAAC

TCCCGGAATAGATAGGGAAAGTGTGAAACCGGAGAATAAAGAAGAAATGTTTCAATCCATAGTGAGATTCCTTCGGGATC

TTACATATCACCCTAACAAATACCAACCTTCCGCCATTAAGAGAGTGTGAATACCCAAACCGGGGAAAGATGAGAAGAGA

CCGTTAGGTATCCCAACCATCAAAGACAGAAGTGCACAAGCTTTAATAAATATGGTATTATATCCTTTAGTAGAGATGAC

CAGCGACCCGAACAGCTATGGTTTCCGTAACAACCGGGATTGTAAGTTCGCAATAGCAGCCTTGAGAGGTAAACTAAAAT

CCAGCGATTTAACCAAGATAAGAGATGGTATGAGAAGAAGATGTGAGAGAAGCAACCCAGGGCAATATCACAAAACTAAC

CAAGAGAAGTGGATACTGGACGCAGACATTAAAGGATTTTTCGATAATATAAACCATGAATGACTAATACAGAATTTATT

CTTACATCCTAAGATTAAAGGAATAGTTCAGAAATGGCTGAAAGCTAGAATCTTTGACAAGGATAAATATACGGACCCCT

AACAGGTACCCCACAAGGTGGGATCATATCCCCTACATTGGCCAATTTCACACTAAATGGTCTAGAAGAGAAAGTATTAA

AGTCAATATACGCCATCACCAAGTCAAGAGAACAAAGAAAAACCGTTAAATTAAGAGATGGAAGAGCGGAAAGGTTATGC

ATGACGGTATCAGTAGTAAGGTATGCTGATGACTTCATAGTCCTAGCTAGAAGCAAGAACATAATAAATAAATATGTTAA

CCCCGCCATAACCGAATTCCTGAGGGAGAGAGGATTGGGACTCTCACCACAGAAAACTAAAACATTTTCTTTAGCCCAAA

AGAACACACAACTAGACTTCTTAGGGTATACCTTTAAGTACCAAGACAAATGGGGCGCGAAGAGAACCATGGTATATGGA

AGGGATTCTAGAAAGAAGGTTATAGCACTATACCCAAATAGAGTTAAGGTGACTAACTTCATCAATAAACTGAAAGAGAT

AGTTAAATCTTCTAAGAACCTCTCGGCTATGGAATTAATCTCAAAATTAAATCCAATTATAAGAGGATGAGCGCAATACT

ACAATATGGAGAATAGCTCAGATACCGGTCAAGAGCGCGAAATGCCCTTTATAATATCATGTGAGATTGAATGAGAGAGA

AACACCCGACTCTGGGTAAAAAGTTAGCTAAGATGTATTTTCTAACGACAAAGAAGAACATAGGTGAAATCGATGCTGAC

TCGCCCAACCCGGGGATAAAGGAGTAAACACGAGTTTGGAGAGGGAAAGAAAGTGGAATATGAGAAGTTAAAA
