# Supplementary material for: Rediscovering an old foe: Optimised molecular methods for DNA extraction and sequencing applications for fungarium specimens of powdery mildew (Erysiphales)
Source: PLoS One. 2020 May 13;15(5):e0232535. doi: 10.1371/journal.pone.0232535 (PMC7219758; doi:10.1371/journal.pone.0232535)
Supplement: S4 File — (DOCX) [file pone.0232535.s004.docx]

Supplementary Information 4

Apple Powdery mildew Podosphaera leucotricha rRNA genome

>lcl|tig00002255 len=15111 reads=1438 covStat=1.00 gappedBases=no class=contig suggestRepeat=no suggestCircular=no

CCGCTACAGTAGATCTGACGCTGTAGCGAGTAGGCAGGCGTGGAGGTCAGTGACGAAGCCTTGGGGTGACCCGGGTAGAA

CGGCCTCTAGTGCAGATCTTGGTGGTAGTAGCAAATACTCAAATGAGAACTTTGAGGACTGAAGTGGGGAAAGGTTCCGT

GTGAACAGCAGTTGGACACGGGTTAGTCGATCCTAAGCCATAGGGAAACTCCGTTTTAAAGTGCGCACTTGTGCGTCGCC

CGGCGAAAGGGAAGCCGGTTAACATTCCGGCACCTGGATGTGGATTCTCCACGGCAACGTAACTGAAAGCGAAGACGACG

GCGGGGCCCTGGGAAGAGTTCTCTTTTCTTCTTAACGGCCCGTCACCCTGAAATCGGTTTGTCCGGAGCTAGGGTTTAAT

GGCCGGTAGAGTCCACACCTTTGTGGGATCCGGTGCGCCCCGACGTCCCTTGAAAATTCGCTGGAAGGAATAGTTTTCAC

GCCAGGTCGTACTCATAACCGCAGCAGGTCTCCAAGGTGAAAAGCCTCTAGTTGATAGAACAATGTAGATAAGGGAAGTC

GGCAAAATAGATCCGTAACTTCGGGAAAAGGATTGGCTCTAAGGGTTGGGTACGTTGGGCCTTGGGTGGAAGCACCGGGA

GCAGGTCGGCACTAGCCTTTACGGGCCGGCGCCTTCCAGCACCTGGTTGTGGACGCCCTTGGCAGGCTTCGGCCGTCCGG

CGTACGCTTAACAACCAACTTAGAACTGGTACGGACAAGGGGAATCTGACTGTCTAATTAAAACATAGCATTGCGATGGC

CAGAAAGTGGTGTTGACGCAATGTGATTTCTGCCCAGTGCTCTGAATGTCAAAGTGAAGAAATTCAACCAAGCGCGGGTA

AACGGCGGGAGTAACTATGACTCTCTTAAGGTAGCCAAATGCCTCGTCATCTAATTAGTGACGCGCATGAATGGATTAAC

GAGATTCCCACTGTCCCTATCTACTATCTAGCGAAACCACAGCCAAGGGAACGGGCTTGGCAGAATCAGCGGGGAAAGAA

GACCCTGTTGAGCTTGACTCTAGTTTGACATTGTGAAAAGACATAGGGTGTAGAATAGGTGGGAGCTTCGGCGCCGGTGA

AATACCACTACCCTTATCGTTTTTACTTAATCAATTAAGCGGAACTGGGCTTCATCGCCCATTTTCTAGCGTTAAGGTCC

TTCGCGGGCCGATCCGGGTTGATGACATTGTCAGGTGGGGAGTTTGGCTGGGGCGGCACATCTGTTAAACCATAACGCAG

GTGTCCTAAGGGGACTCATGGAGAACAGAAATCTCCAGTAGAGCAAAAGGGCAAAAGTCCCCTTGATTTTGATTTTCAGT

GTGAATACAAACCATGAAAGTGTGGCCTATCGATCCTTTAGTCCCTCGAAATTTGAGGCTAGAGGTGCCAGAAAAGTTAC

CACAGGGATAACTGGCTTGTGGCAGCCAAGCGTTCATAGCGACGTTGCTTTTGATCCTTCGATGTCGGCTCTTCCTATCA

TACCGAAGCAGAATTCGGTAAGCGTTGGATTGTTCACCCACTAATAGGGAACGTGAGCTGGGTTTAGACCGTCGTGAGAC

AGGTTAGTTTTACCCTACTGATGAATGTCGTCCCAATGGTAATACCGCTTAGTACGAGAGGAACCGCGGTTTCAGATAAT

TGGTTTTGCGGCTGTCCGACCGGGCAGTGCCGCGAAGCTACCATCTGCTGGATTATGGCTGAACGCCTCTAAGTCAGAAT

CCATGCCAGAAAGGGACGATTTCCCCACATCGTAGTCGGATACGAATAGGCCTTTGGCCCTGAACCTTAGCAGGTTGGCG

ACCGGCTCCGGGTAGAAGTCCGGGGTCGAGCTGACGAATTGCAATTTCACAATGCGTAGGGATAAATCCTTTGCAGACGA

CTTAGTTGTGCAACCGGGTCGTGTAAGCAGTCGAGTAGCCTTGTTGTTACGAGCTGCTGAGCGTAAGCCCGTCGTTGCCT

AGATTTGTCTTTGACCTCCCCATTGATTTAACTGGCCTAGTGCCGGTTGTTGAAGGAAGGCGGGTGTTTTAACTGCTCGG

TTTAACCGCCGGGTGGTTTGATTCTCCTAGCCCGACGGCGGTGACAGGGTCACCACAGAAGTACTTGGTCGTTTCTAGAG

GCGGCGGTGGTGGTGCTGCTGCTGCGGTGCTGGCTGCAGAATATATCGGTGGTGTGGGTTGGTGGCTGCAAGGGTTTCTG

TCTGGCAGCTGCATTCCTCTGGGCTGGGACGGACAGGGTCACCCGCAGTCCCTTGTGCATCTCACTGCGCCGGACGAAGA

TCAGGGTCACCCGCCGCCTGTCGCTCGCCTTGCTGGCCGTTCCTAGCCTTTGCATCGCGCAGCCGAACAGGTTTCGACGC

CGTGGAACGCCCTTAGTGCGAGGGCTATGGTGTATTACGGTGCTCGGTCGAGGTGTCTAGATTGCCTGGGCCGCGATCCG

ACGCGAACTGGAGAGCAGGGTCACCCTTGGCCGGCCACCAGAGGACAGGGTCACACATGGCCGGCCACCAGAGGACAGGG

TCACGGGCGGCCGCCGCGGACAGGGTCACACGTGGCCGGCCACCCTGGGTCAGGGTCACAGGTGGCCGCCGCGGACAGGG

TCACACATGGCCGGCCACTCTGGATCAGGGTCACACATGGCCGGCCGGCCAAGGACAGGGTCACCCATGGCCACCTTGGA

CAGGGTCACCGCCGGCCGGCCAGCGCGCGGTCGGCCAACCACGCAGGCGGCCAGTGCCAGGGTCCCCTTCTCCAGGCACA

CCTCGACCGCCACCCCCGGTGCCACCCACTGCCCCTGGCTGTACAGACCACCTCCAGGGACCCACGGGACCTCACCCTCC

AAATTTCAGAGACCCTCGCCAGGCGGTGCTCTGGGGGTCGGCGATGACCGGCGCTGAGGGCAATAGCGAATCCCATACAA

AATCGGTCGCGTAGGACATGGAATTCCGTGCACGAACCGCTGTACGGCGGATGTCAGCACACGCAAGTTCGGAGGGTATA

TGGAGAGGGATGGTTGGCATGCAAGTCGGTCTCAGGGTTTCAACCAAGCGTTGTGGCCCTTTAACCCTTGCCGTCGCCCC

TGTTCCCGTGGGGTCTGAGGGTTTAATCTTCTAACGGCGGTGGTTCAACCGCGCCGGTGAAACTTCCGTCCGAGAGACAG

CGATTATAGGCGCATCTGTCGGTAGAGTTTAACCGGGGTGTTTAACGGTCTGTGGTGAATGGCAAGGTGGGCTCCGCCGA

ACAAAGGCGTACCCCCACCGGTCTTTCCACCAGTCCGCAGGCGTACCCGGCGCGAGGAGGCAGCGATTATAGGCGCACCT

CTTCCGTCCGGCGTGTCTGACCGCCACCGGCGAGGGCGGTAGAGGTAATGCGTTGCAAGACACCCCTACCTGCTCATTGT

CCGGTAAGCGCCGTACCGGCCGACCGCCACCTTGGGGTCCGGCGGCTTGGATTGATCCCACAGCAGTAGTGGGACCATAG

AAGAGGGTCGCCAGGCCTCTGGTGTGGGGTTTCCCTCCAGAGGCAGGCCGCCGGCGCAACAGTTGACCGGAGGTTGGGGA

CAGGGTCACCCGCGGTCGCTTTGGCCCGTGGCGGCGCGGTTTTGGGTTTTCCCTCTTCTCCAAGGCGCGGGGAGACTGAC

TACCAAAGAAGACGGCGCCGGACAGGTGAGAACGGCCGGAGGCGGCGGTGGCGTGCAACTTGCGGGCGCCGTAGGGCAGG

GTCACCCCTCGAAAATGTGGCCGGCCGTCCCAGGACAGGGTGCCCCTCGTTTGGCCGGCCGCAGGGGACAGGGTCACCTC

CCGAGTGGCCGCCAGGGTCACCTTTCGGGTCTGGCCATGGCCGGCGGCCGGCGGAGGACAGGGTCACCTAAAATGTTGGC

CGGCGGACCGGTGGCAGGGTCACCCCGAGGCTTGTGAGCCCCTGCGAGCCAGACGTCTTCCAGGTAGGCAGCTGAGGCGG

TTCGGGAGGTCTAGGGAGCCCGGGGAGCCGTGTGCTTGTCTCGCTGCGGTGGTTCACCTACGGACGGCCGCCGGCGGATC

GGGCGGCGGCGGCGGCGGCGGCGGCGAGGTCAGTTTAGGGTCTATGGCACTTCCGGCCGGCGTAGCGGGGCCTCTAGACG

GTAGAGAGGCTTTATATGACCGGCCATTGACTCTATATGTCAGGGGTAGGGATGGGTGGTGATCTGCTGCAAGGACTCAC

CTGACCATTCTGCTTTTCAAAATGTCATTTACACCAAAACAACTTCGGTCTATACACGGCGACAGGAGGGTCTGGCACTC

CATACCAGTGCTCTAGGGGTAGTGTAGCGTGGCCGTAAGAGGCCCCAAGCGTCGGTTTGACCAACCATGCCGTCGCCGCC

GCGGTATGTCGCCGCCAACTTTGGGACAGGGTCATACCCCAACTTGACTTCTCCAGGCACCCCTCGGCCACCACCCACGG

AGCCACCTACTGCCCCTGGCTGTACAGACCACCTCCAGGGACCCACGGGACCTCACCCTCCAAATTTCAGAGACCCTCGC

CAGGCGGTGCTCTGGGGGTCGGCGATGACCGGCGCTGAGGGCAATAGCGAATCCCATACAAAATCGGTCGCGTAGGACAT

GGAATTCCGTGCACGAACCGCTGTACGGCGGACGTCAGCATATACAAGTTCGGAGGGTATATGAAGGGGAAAGGCAGTGG

CCGAGGCAGTTGAAGCGAACCAACTAACAATTGGCGCCGCTCCTGCTTATGAGCCCGCCCGACCTCAATCGCGGCCTTCC

GCGTCGGCAAAGCCGTCTACCGTGGGCCGGCCGTGTATCACTACTGCTTCGCGCTGGTGCGACCGGTCCTTGAAGGTGGC

TGCCGCGGGTGTCTTCTTCGGAATACACCTGTGACCGAAGTCCGTTTAATCTTGATTAGGCGGCCATGTCGGAGTCTGTG

GATGTTTGACCGTTGGTTAACACTATCCTAAGACACAATCGGGGCACGCCCTAGCAGGTAACCGTCTCACGACGTGACTT

GCGAAAGGTAACCTCCACTTGGGGTACGCATGCCATAGAACGTAACAACCTCACGGTTGAAGCGATCCTACGGCGGACCG

TCACCCTGGGGAATTCAGGTGGGAAAACTGGATGGGATTGTCCGGCGCGGGTCCAGGTCTAACGACCGCTCCCACGCAGG

TGATCCCGGGCCTATCCATTTCGATGGAGAAAGCCCCTTCCGTTTAACAACTCTTGCCAAACCCTCCGGGGCGCGGTTCA

CCCGGTTCATCGGGAGGCGTGAAAGCGACTCCCCTGTCCCGGCTAAGATAGTTACCTGGTTGATTCTGCCAGTAGTCATA

TGCTTGTCTCAAAGATTAAGCCATGCATGTCTAAGTATAAGCAACTATACGGTGAAACTGCGAATGGCTCATTAAATCAG

TTATCGTTTATTTGATAGTACCTTACTACTTGGATAACCGTGGTAATTCTAGAGCTAATACATGCTAAAACCCCAACTTC

GGAAGGTGTATTTATTAGATAAAACCAACGCCCTTCGGGGCTCCTTGGTGATTCATAATAACTAAACGAATCGCATGGCC

TTGCGCCGGCGATGGTTCATTCAAATTTCTGCCCTATCAACTTTCGATGGTAGGATAGTGGCCTACCATGGTATCAACGG

GTAACGGGGAATTAGGGTTCTATTCCGGAGAGGGAGCCTGAGAAACGGCTACCACATCCAAGGAAGGCAGCAGGCGCGCA

AATTACCCAATCCCGACACGGGGAGGTAGTGACAATAAATACTGATACAGGGCTCTTTTGGGTCTTGTAATTGGAATGAG

TACAATTTAAATCCCTTAACGAGGAACAATTGGAGGGCAAGTCTGGTGCCAGCAGCCGCGGTAATTCCAGCCCAATAGCG

TATATTAAAGTTGTTGCAGTTAAAAGCTCGTAGTTGAACCTTGGGCCTGGCTGGCCGGTCCGCCTCACCGCGTGTACTGG

TCCGGCCGGGCCTTTCCTTCTGGGAGCCGCATGCCCTTCACTGGGCGTGTCGGGGAACCAGGACTTTACTTTGAAAAATT

AGAGTGTTCAAAGCAGGCCTTTGCTCGAATACATTAGCATGGAATAATAGAATAGGACGTGCGGTTCTATTTTGTTGGTT

TCTAGGACCGCCGTAATGATTAATAGGGATAGTCGGGGCATCAGTATTCAATTGTCAGAGGTGAAATTCTTGGATTTATT

GAAGACTAACTACTGCGAAAGCATTTGCCAAGGATGTTTTCATTAATCAGTGAACGAAAGTTAGGGGATCGAAGACGATC

AGATACCGTCGTAGTCTTAACCATAAACTATGCCGACTAGGATCGGGCGATGTTATCATTTTGACTCGCTCGGCACCTTA

CGAGAAATCAAAGTCTTTGGGTTCTGGGGAGTATGGTCGCAAGGCTGAAACTTAAAGAAATTGACGGAAGGGCACCACCA

GGCGTGGAGCCTGCGGCTTAATTTGACTCAACACGGGGAAACTCACCAGGTCCAGACACAATAAGGATTGACAGATTGAG

AGCTCTTTCTTGATTTTGTGGGTGGTGGTGCATGGCCGTTCTTAGTTGGTGGAGTGATTTGTCTGCTTAATTGCGATAAC

GAACGAGACCTTAACCTGCTAAATAGCCCGGCCCGCTTTGGCGGGTCGCCGGCTTCTTAGAGGGACTATCGGCTCAAGCC

GATGGAAGTTTGAGGCAATAACAGGTCTGTGATGCCCTTAGATGTTCTGGGCCGCACGCGCGCTACACTGACAGAGCCAA

CGAGTTCATTTCCTTGCCCGGAAGGGTTGGGTAATCTTGTTAAACTCTGTCGTGCTGGGGATAGAGCATTGCAATTATTG

CTCTTCAACGAGGAATGCCTAGTAAGCGTACGTCATCAGCGTGCGTTGATTACGTCCCTGCCCTTTGTACACACCGCCCG

TCGCTACTACCGATTGAATGGCTGAGTGAGGCCTTCGGACTGGCCCAGGGAGGTCGGCAACGACCACCCAGGGCCGGAAA

GTTGGTCAAACTCCGTCATTTAGAGGAAGTAAAAGTCGTAACAAGGTTTCCGTAGGTGAACCTGCGGAAGGATCATTAAA

GAGTAAGGGTGCTCAGCGCCCGACCTCCAACCCTTTGTTGTTAAAACTACCTTGTTGCTTTGGCGGGACCGCTCGGTCTC

GAGCCGCTGGGGATTCGTCCCAGGCGAGCGCCCGCCAGAGTTAAACCAAACTCTTGTTATTTAACCGGTCGTCTGAGTTA

AAATTTTGAATAAATCAAAACTTTCAACAACGGATCTCTTGGTTCTCGCATCGATGAAGAACGCAGCGAAATGCGATAAG

TAATGTGAATTGCAGAATTCAGTGAATCATCGAATCTTTGAACGCACATTGCGCCCCTTGGTATTCCGAGGGGCATGCCT

GTTCGAGCGTCATTACACCACTCAAGCTATGCTTGGTATTGGGCGTCGTCCTTAGTTGGGCGCGCCTTAAAGACCTCGGC

GAGGCCACTCCGGCTTTAGGCGTAGTAGAATTTATTCGAACGTCTGTCAAAGGAGAGGAACTCTGCCGACTGAAACCTTT

ATTTTTCTAGGTTGACCTCGGATCAGGTAGGGATACCCGCTGAACTTAAGCATATCAATAAGCGGAGGAAAAGAAACCAA

CAGGGATTGCCCTAGTAACGGCGAGTGAAGCGGCAACAGCTCAAATTTGAAAGCTAGCCTTCGGGTTCGCATTGTAATTT

GTAGAGGATGATTTGGGGAAGCCGCCTGTCTAAGTTCCTTGGAACAGGACGTCATAGAGGGTGAGAATCCCGTATGTGAC

AGGAAATGGCACCCTATGTAAATCTCCTTCGACGAGTCGAGTTGTTTGGGAATGCAGCTCTAAATGGGAGGTAAATTTCT

TCTAAAGCTAAATATTGGCGAGAGACCGATAGCGCACAAGTAGAGTGATCGAAAGATGAAAAGCACTTTGGAAAGAGAGT

TAAAAGCACGTGAAATTGTTGAAAGGGAAGCGCTTGCAATCAGACTTGTTTAAACTGTTCGGCCGGTCTTCTGACCGGTT

TACTCAGTTTGGACAGGCCAGCATCAGTTTCGGCGGCCGGATAAAGGCTCTGGGAATGTGGCCTCCACTTCGGTGGAGGT

GTTATAGCCCAGGGTGTAATACGGCCAGCCGGGACTGAGGTCCGCGCTTCGGCTAGGATGCTGGCGTAATGGTTGTAAGC

GACCCGTCTTGAAACACGGACCAAGGAGTCTAACATCTATGCGAGTGTTCGGGTGTCAAACCCCTGCGCGTAATGAAAGT

GAACGGAGGTGGGAACCCGCAAGGGTGCACCATCGACCGATCCTGATGTCTTCGGATGGATTTGAGTAAGAGCATAGCTG

TTGGGACCCGAAAGATGGTGAACTATGCCTGAATAGGGTGAAGCCAGAGGAAACTCTGGTGGAGGCTCGCAGCGGTTCTG

ACGTGCAAATCGATCGTCAAATTTGGGTATAGGGGCGAAAGACTAATCGAACCATCTAGTAGCTGGTTCCTGCCGAAGTT

TCCCTCAGGATAGCAGTAACGTTTTCAGTTTTATGAGGTAAAGCGAATGATTAGAGGCCTTGGGGTTGAAACAACCTTAA

CCTATTCTCAAACTTTAAATATGTAAGAAGTCCTTGTTACTTAGTTGAACGTGGACATTTGAATGTATCGTTACTAGTGG

GCCATTTTGGTAAGCAGAACTGGCGATGCGGGATGAACCGAACGCGAGGTTAAGGTGCCGGAATACACGCTCATCAGACA

CCACAAAAGGTGTTAGTTCATCTAGACAGCAGGACGGTGGCCATGGAAGTCGGAATCCGCTAAGGAGTGTGTAACAACTC

ACCTGCCGAATGAACTAGCCCTGAAAATGGATGGCGCTCAAGCGTGTTACCCATACCTCGCCGCTACAGTAGATTCGACG

CTGTAGCGAGTAGGCAGGCGTGGAGGTCAGTGACGAAGCCTTGGGGTGACCCCGGGTAGAACGGCCTCTAGTGCAGATCT

TGGTGGTAGTAGCAAATACTCAAATGAGAACTTTGAGGACTGAAGTGGGGAAAGGTTCCGTGTGAACAGCAGTTGGACAC

GGGTTAGTCGATCCTAAGCCATAGGGAAACTCCGTTTTAAAGTGCGCACTTGTGCGTCGCCCGGCGAAAGGGAAGCCGGT

TAACATTCCGGCACCTGGATGTGGATTCTCCACGGCAACGTAACTGAAAGCGAAGACGACGGCGGGGCCCTGGGAAGAGT

TCTCTTTTCTTCTTAACGGCCCGTCACCCTGAAATCGGTTTGTCCGGAGCTAGGGTTTAATGGCCGGTAGAGTCCCACAC

CTTTGTGGGATCCGGTGCGCCCCGACGTCCCTTGAAAATTCGCTGGAAGGAATAGTTTTCACGCCAGGTCGTACTCATAA

CCGCAGCAGGTCTCCAAGGTGAAAAGCCTCTAGTTGATAGAACAATGTAGATAAGGGAAGTCGGCAAAATAGATCCGTAA

CTTCGGGAAAAGGATTGGCTCTAAGGGTTGGGTACGTTGGGCCTTGGGTGGAAGCACCGGGAGCAGGTCGGCACTAGCCT

TTACGGGCCGGCGCCTTCCAGCACCTGGTTGTGGACGCCCTTGGCAGGCTTCGGCCGTCCGGCGTACGCTTAACAACCAA

CTTAGAACTGGTACGGACAAGGGGAATCTGACTGTCTAATTAAAACATAGCATTGCGATGGCCAGAAAGTGGTGTTGACG

CAATGTGATTTCTGCCCAGTGCTCTGAATGTCAAAGTGAAGAAATTCAACCAAGCGCGGGTAAACGGCGGGAGTAACTAT

GACTCTCTTAAGGTAGCCAAATGCCTCGTCATCTAATTAGTGACGCGCATGAATGGATTAACGAGATTCCCACTGTCCCT

ATCTACTATCTAGCGAAACCACAGCCAAGGGAACGGGCTTGGCAGAATCAGCGGGGAAAGAAGACCCTGTTGAGCTTGAC

TCTAGTTTGACATTGTGAAAAGACATAGGGGTGTAGAATAGGTGGGAGCTTCGGCGCCGGTGAAATACCACTACCCTTAT

CGTTTTTACTTAATCAATTAAGCGGAACTGGGCTTCATCGCCCATTTTCTAGCGTTAAGGTCCTTCGCGGGCCGATCCGG

GTTGATGACATTGTCAGGTGGGGAGTTTGGCTGGGGCGGCACATCTGTTAAACCATAACGCAGGTGTCCTAAGGGGACTC

ATGGAGAACAGAAATCTCCAGTAGAGCAAAGGGCAAAAGTCCCCTTGATTTTGATTTTCAGTGTGAATACAAACCATGAA

AGTGTGGCCTATCGATCCTTTAGTCCCTCGAAATTTGAGGCTAGAGGTGCCAGAAAAGTTACCACAGGGATAACTGGCTT

GTGGCAGCCAAGCGTTCATAGCGACGTTGCTTTTGATCCTTCGATGTCGGCTCTTCCTATCATACCGAAGCAGAATTCGG

TAAGCGTTGGATTGTTCACCCACTAATAGGGAACGTGAGCTGGGTTTAGACCGTCGTGAGACAGGTTAGTTTTACCCTAC

TGATGAATGTCGTCCCAATGGTAATACCGCTTAGTACGAGAGGAACCGCGGTTTCAGATAATTGGTTTTGCGGCTGTCCG

ACCGGGCAGTGCCGCGAAGCTACCATCTGCTGGATTATGGCTGAACGCCTCTAAGTCAGAATCCATGCCAGAAAGGGACG

ATTTCCCCTACACATCGTAGTCGGATACGAATAGGCCTTTGGCCCTGAACCTTAGCAGGTTGGCGACCGGCTCCGGGTAG

AAGTCCGGGTCGAGCTGACGAATTGCAATTTCACAATGCGTAGGGATAAATCCTTTGCAGACGACTTAGTTGTGCAACCG

GGTCGTGTAAGCAGTCGAGTAGCCTTGTTGTTACGAGCTGCTGAGCGTAAGCCCGTCGTTGCCTAGATTTGTCTTTGACC

TCCCCATTGATTTAACTGGCCTAGTGCCGGTTGTTGAAGGAAGGCGGGTGTTTTAACTGCTCGGTTTAACCGCCGGGTGG

TTTGATTCTCCTCGCCCGACGGCGGTGACAGGGTCACTGCAGAAGTACTTGGTCGTTTCTAGAGGCGGCGGTGGTGGTGC

TGCTGCTGCGGTGCTGGCTGCAGAATATATCGGTGGTGTGGGTTGGTGGCTGCAAGGGTTTCTGTCTGGCAGCTGCATTC

CTCTGGGCTGGGACGGACAGGGTCACCCGCAGTCCCTTGTGCATCTCACTGCGCCGGACGAAGATCAGGGTCACCCGCCG

CCTGTTGCTCGCCTTGCTGGCCGTTCCTAGCCTTTGCATCGCGCAGCCGAACAGGTTTCGACGCCGTGGAACGCCCTTAG

TGCGAGGGCTATGGTGTATTACGGTGCTCGGTCGAGGTGTCTAGATTGCCTGGGCCGCGATCCGACGCGAACTGGAGAGC

AGGGTCACCCTTGGCCGGCCACCAGAGGACAGGGTCACACATGGCCGGCCACCAGAGGACAGGGTCACGGGCGGCCGCCG

CGGACAGGGTCACACGTGGCCGGCCACCCTGGGTCAGGGTCACAGGTGGCCGCCGCGGACAGGGTCACACATGGCCGGCC

ACTCTGGATCAGGGTCACACATGGCCGGCCGGCCAAGGACAGGGTCACCCATGGCCACCTTGGACAGGGTCACCGCCGGC

CGGCCAGCGCGCGTCGGCCAACCACGCAGGCGGCCAGTGCCAGGGTCCCCTCTCCAGGCACACCTCGACCGCCACCCCCG

GTGCCACCCACTGCCCCTGGCTGTACAGACCACCTCCAGGGACCCACGGGACCTCACCCTCAAATTTCAGAGACCCTCGC

CAGGCGGTGCTCTGGGGGTCGGCGATGACCGGCGCTGAGGGCAATAGCGAATCCCATACAAAATCGGTCGCGTAGGACAT

GGAATTCCGTGCACGAACCGCTGTACGGCGGATGTCAGCACACGCAAGTTCGGAGGGTATATGGAGAGGGATGGTTGGCA

TGCAAGTCGGTCTCAGGGTTTCAACCAAGCGTTGTGGCCCTTTAACCCTTGCCGTCGCCCCCTGTTCCCGTGGGGTCTGA

GGGTTTAATCTTCTAACGGCGGTGGTTCAACCGCGCCGGTGAAACTTCCGTCCGAGACAGCGATTATAGGCGCATCTGTC

GGTAGAGTTTAACCGGGGTGTTTAACGGTCTGTGGTGAATGGCAAGGTGGGCTCCGCCGAACAAAGGCGTACCCCCACCG

GTCTTTCCACCAGTCCGCAGGCGTACCCGGCGCGAGGAGGCAGCGATTATAGGCGCACCTCTTCCGTCCGGCGTGTCTGA

CCGCCACCGGCGAGGGCGGTAGAGGTAATGCGTTGCAAGACACCCCTACCTGCTCATTGTCCGGTAAGCGCCGTACCGGC

CGACCGCCACCTTGGGGTCCGGCGGCTTGGATTGATCCCACAGCAGTAGTGGGACCATAGAAGAGGTCGCCAGGCCTCTG

GTGTGGGGTTTTCCCCTCCAGAGGCAGGCCGCCGGCGCAACAGTTGACCGGAGGTTGGGGACAGGGTCACCCGCGGTCGC

TTTGGCCCGTGGCGGCGCGGTTTTGGGTTTTCCCCTTCTCCAAGGCGCGGGGAGACTGACTACCAAAGAAGACGGCGCCG

GACAGGTGAGAACGGCCGGAGGCGGCGGTGGCGTGCAACTTGCGGGCGCCGTAGGGCAGGGTCACCCCTCGAAAATGGCC

GGCCGTCCCAGGACAGGGTGCCCCTCGTTTGGCCGGCCGCAGGGGACAGGGTCACCTCGAGTGGCCGCCAGGGTCACCTT

TCGGGGTCTGGCCATGGCCGGCGGCCGGCGGAGGACAGGGTCACCTAAAATGTTGGCCGGCGGACCGGTGGCAGGGTCAC

CCCGAGGCTTGTGAGCCCCTGCGAGCCAGACGTCTTCCAGGTAGGCAGCTGAGGCGGTTCGGGAGGTCTAGGGAGCCCGG

GGAGCCGTGTGCTTGTCTCTCGCTGCGGTGGTTCACCTACGGACGGCCGCCGGCGGATCGGGCGGCGGCGGCGGCGGCGG

CGGCGAGGTCAGTTTAGGGTCTATGGCACTTCCGGCCGGCGTAGCGGGGCCTCTAGACGGTAGAGAGGCTTTATATGACC

GGCCATTGACTCTATATGTCAGGGGTAGGGATGGGTGGTGATCTGCAAGGACTCACCTGACCATTCTGCTTTTCAAAATG

TCATTTACACCAAAACAACTTCGGTCTATACACGGCGACAGGAGGGTCTGGCACTCCATACCAGTGCTCTAGGGGTAGTG

TAGCGTGGCCGTAGAGGCCCCAAGCGTCGGTTTGACCAACCATGCCGTCGCCGCCGCGGTATGTCGCCGCCAACTTTGGG

ACAGGGTCATACCCCCACGACCCCCTCTCCAGGCACACCTCGGCCACCACCCCCGGAGCCACCTACTGCCCCTGGCTGTA

CAGACCACCTCCAGGGACCCACGGGACCTCACCCTCAAATTTCAGAGACCCTCGCCAGGCGGTGCTCTGGGGGTCGGCGA

TGACCGGCGCTGAGGGCAATAGCGAATCCCATACAAAATCGGTCGCGTAGGACATGGAATTCCGTGCACGAACCGCTGTA

CGGCGGACGTCAGCATATACAAGTTCGGAGGGTATATGAAGGAAAGGTGGCCGAGGCAGTTGAAGCGAACCAACTAACAA

TTGGCGCCGCTCCTGCTTATGAGCCCGCCCGACCTCAATCGCGGCCTTCCGCGTCGGCAAAGCCGTCTACCGTGGGCCGG

CCGTGTATCACTACTGCTTCGCGCTGGTGCGACCGGTCCTTGAAGGTGGCTGCCGCGGGTGTCTTCTTCGGAATACACCT

GTGACCGAAGTCCGTTTAATCTTGATTAGGCGGCCATGTCGGAGTCTGTGGATGTTTGACCGTTGGTTAACACTATCCTA

AGACACAATCGGGGCACGCCCTAGCAGGTAACCGTCTCACGACGTGACTTGCGAAAGGTAACCTCCACTTGGGGTACGCA

TGCCATAGAACGTAACAACCTCACGGTTGAAGCGATCCTACGGCGGACCGTCACCCTGGGGAATTCAGGTGGGAAAACTG

GATGGGATTGTCCGGCGCGGGTCCAGGTCTAACGACCGCTACGCAGGTGATCCCGGGCCTATCCATTTCGATGGAGAAAG

CCCCTTCCGTTTAACAACTCTTGCCAAACCCTCCGGGGCGCGGTTCACCCGGTTCATCGGGAGGCGTGAAAGCGACTCCC

CTGTCCCGGGCTAAGATAGTTACCTGGTTGATTCTGCCAGTAGTCATATGCTTGTCTCAAAGATTAAGCCATGCATGTCT

AAGTATAAGCAACTATACGGTGAAACTGCGAATGGCTCATTAAATCAGTTATCGTTTATTTGATAGTACCTTACTACTTG

GATAACCGTGGTAATTCTAGAGCTAATACATGCTAAAACCCCAACTTCGGAAGGGGTGTATTTATTAGATAAAACCAACG

CCCTTCGGGGCTCCTTGGTGATTCATAATAACTAAACGAATCGCATGGCCTTGCGCCGGCGATGGTTCATTCAAATTTCT

GCCCTATCAACTTTCGATGGTAGGATAGTGGCCTACCATGGTATCAACGGGTAACGGGGAATTAGGGTTCTATTCCGGAG

AGGGAGCCTGAGAAACGGCTACCACATCCAAGGAAGGCAGCAGGCGCGCAAATTACCCAATCCCGACACGGGGAGGTAGT

GACAATAAATACTGATACAGGGCTCTTTTGGGTCTTGTAATTGGAATGAGTACAATTTAAATCCTTAACGAGGAACAATT

GGAGGGCAAGTCTGGTGCCAGCAGCCGCGGTAATTCCAGCTCAATAGCGTATATTAAAGTTGTTGCAGTTAAAAGCTCGT

AGTTGAACCTTGGGCCTGGCTGGCCGGTCCGCCTCACCGCGTGTACTGGTCCGGCCGGGCCTTTCCTTCTGGGGAGCCGC

ATGCCCTTCACTGGGCGTGTCGGGGAACCAGGACTTTACTTTGAAAAATTAGAGTGTTCAAAGCAGGCCTTTGCTCGAAT

ACATTAGCATGGAATAATAGAATAGGACGTGCGGTTCTATTTTGTTGGTTTCTAGGACCGCCGTAATGATT
